# 1,2‐Carboboration of Arylallenes by In Situ Generated Alkenylboranes for the Synthesis of 1,4‐Dienes

**DOI:** 10.1002/chem.202200470

**Published:** 2022-04-21

**Authors:** Arthur Averdunk, Max Hasenbeck, Tizian Müller, Jonathan Becker, Urs Gellrich

**Affiliations:** ^1^ Institut für Organische Chemie Justus-Liebig-Universität Gießen Heinrich-Buff-Ring 17 35392 Gießen Germany; ^2^ Institut für Anorganische und Analytische Chemie Justus-Liebig-Universität Gießen Heinrich-Buff-Ring 17 35392 Gießen Germany

**Keywords:** 1,2-carboboration, 1,4-dienes, boranes, density functional theory, Lewis acids

## Abstract

We herein report a novel method for the coupling of unactivated alkynes and arylallenes, which relies on an unprecedented and regioselective 1,2‐carboboration of the allene by an alkenylborane. The alkenylborane is conveniently prepared *in situ* by hydroboration of an alkyne with Piers’ borane, *i. e*., HB(C_6_F_5_)_2_. The boryl‐substituted 1,4‐dienes that are formed by this carboboration are well‐suited for a subsequent Suzuki‐Miyaura coupling with aryl iodides. This allowed us to develop a three‐step, one‐pot protocol for the synthesis of aryl‐substituted 1,4‐dienes. The generality of the reaction was demonstrated by the synthesis of twenty dienes with modular variations of all three reaction partners. The mechanism of the new 1,2‐carboboration was investigated using dispersion corrected double‐hybrid DFT computations that allowed us to rationalize the chemo‐ and regioselectivity of this key step.

## Graphical abstract

**A chemo‐ and regioselective** 1,2‐carboboration of arylallenes by alkenylboranes, formed *in situ* by hydroboration of alkynes with Piers’ borane (HB(C_6_F_5_)_2_) is reported. This novel 1,2‐carboboration and a subsequent Suzuki‐Miyaura coupling were used for the synthesis of twenty new aryl substituted 1,4‐dienes.

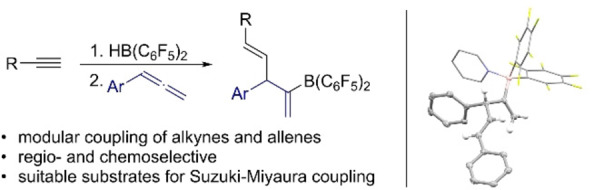

## Introduction

**Scheme 1:**
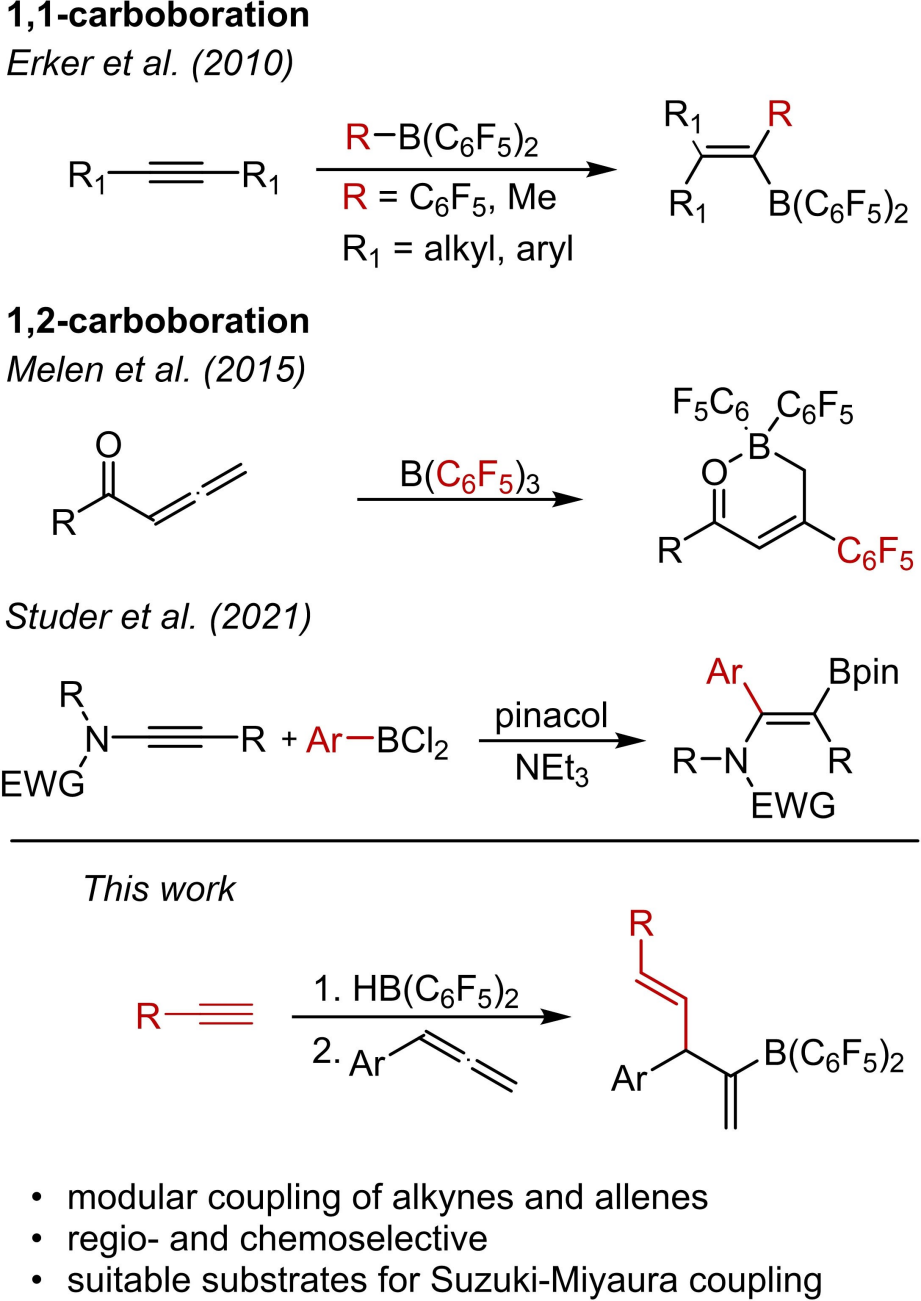
The context of this work: Recent examples of carboborations and the consecutive hydroboration‐1,2‐carboboration sequence reported herein.

Carboborations are a powerful and atom economic synthetic method because they simultaneously form a C−C bond and install a valuable boryl unit into a molecular framework by adding an organoborane to a double or triple bond. In recent years, uncatalyzed direct carboborations by Lewis acidic boranes have received increasing attention.^[1]^ A seminal contribution to this field is the 1,1‐carboboration of alkynes by the Lewis acidic tris(perfluorophenyl)borane (BCF) that was independently discovered by Erker and Berke.[^2^, ^3^] The groups of Hashmi and Stephan reported the cyclopropanation and the formation of allyl boranes by 1,1‐carboboration of enynes and propargylic esters, respectively.[^2e^, ^2j^] We recently reported that the reaction of phenylallene with BCF leads to the formation of a C_6_F_5_‐substituted indene via a cyclisation, 1,1‐carboboration, retro‐hydroboration sequence.^[2k]^ In contrast, there are still only a few reports on uncatalyzed 1,2‐carboborations.^[4]^ Melen and co‐workers described the 1,2‐addition of B(C_6_F_5_)_3_ to the terminal double bond of allenylketones (Scheme 1).^[4g]^ In 2021, Studer *et al*. reported the 1,2‐carboboration of alkyne‐substituted sulphonamides with dichloroarylboranes. The dichloroborane moieties were subsequently transformed *in situ* to their respective pinacol derivatives (Scheme 1).^[4b]^

We became now interested in devising a protocol that allows the selective group transfer of an easily interchangeable substituent at the boron to a non‐activated substrate via a carboboration. A fast and convenient way to synthesize electron‐poor boranes is the hydroboration of alkynes by the very active hydroboration reagent HB(C_6_F_5_)_2_, also known as Piers’ borane.^[5]^ Since allenes are reactive towards strong boron‐based Lewis acids, we aimed to elucidate whether the reaction of electron‐poor alkenylboranes, synthesized by hydroboration of alkynes, could lead to a selective transfer of the vinyl group to an allene in a carboboration reaction.[^4g^, ^6^, ^7^]

## Results and Discussion

**Scheme 2:**
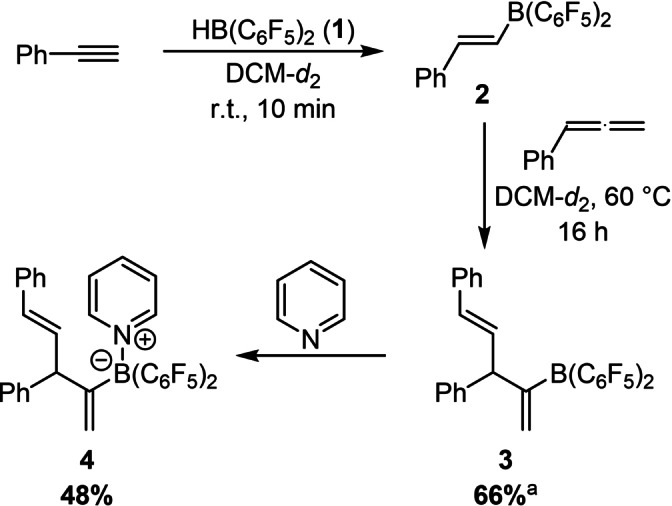
Formation of the 1,4‐diene **3** upon 1,2‐carboboration of phenylallene by the alkenylborane **2** and isolation of the respective pyridine adduct **4**. a) Yield determined by ^1^H NMR with trimethoxybenzene as internal standard.

We commenced our attempts by preparing alkenylborane **2** by hydroboration of phenylacetylene with Piers’ borane **1** in deuterated dichloromethane (Scheme 2).^[5]^ The corresponding ^1^H NMR shows complete conversion to alkenylborane **2** after 10 min at r.t. Next, phenylallene was added to the reaction mixture.

**Figure 1:**
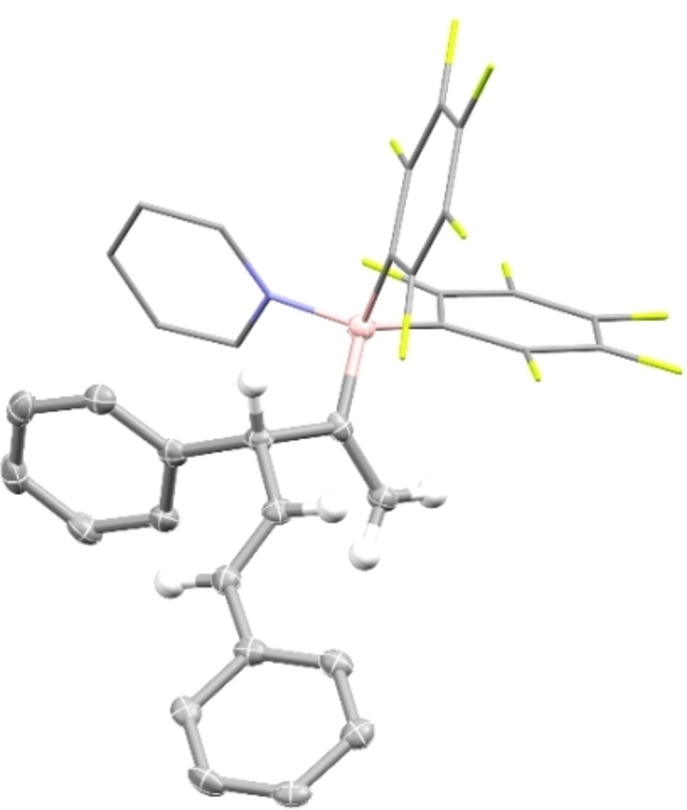
Molecular structure of 4 derived from SCXRD (50 % probability ellipsoids, all hydrogens attached to phenyl rings are omitted and pyridine and C6F5 rings are shown in stick representation for clarity).

**Figure 2:**
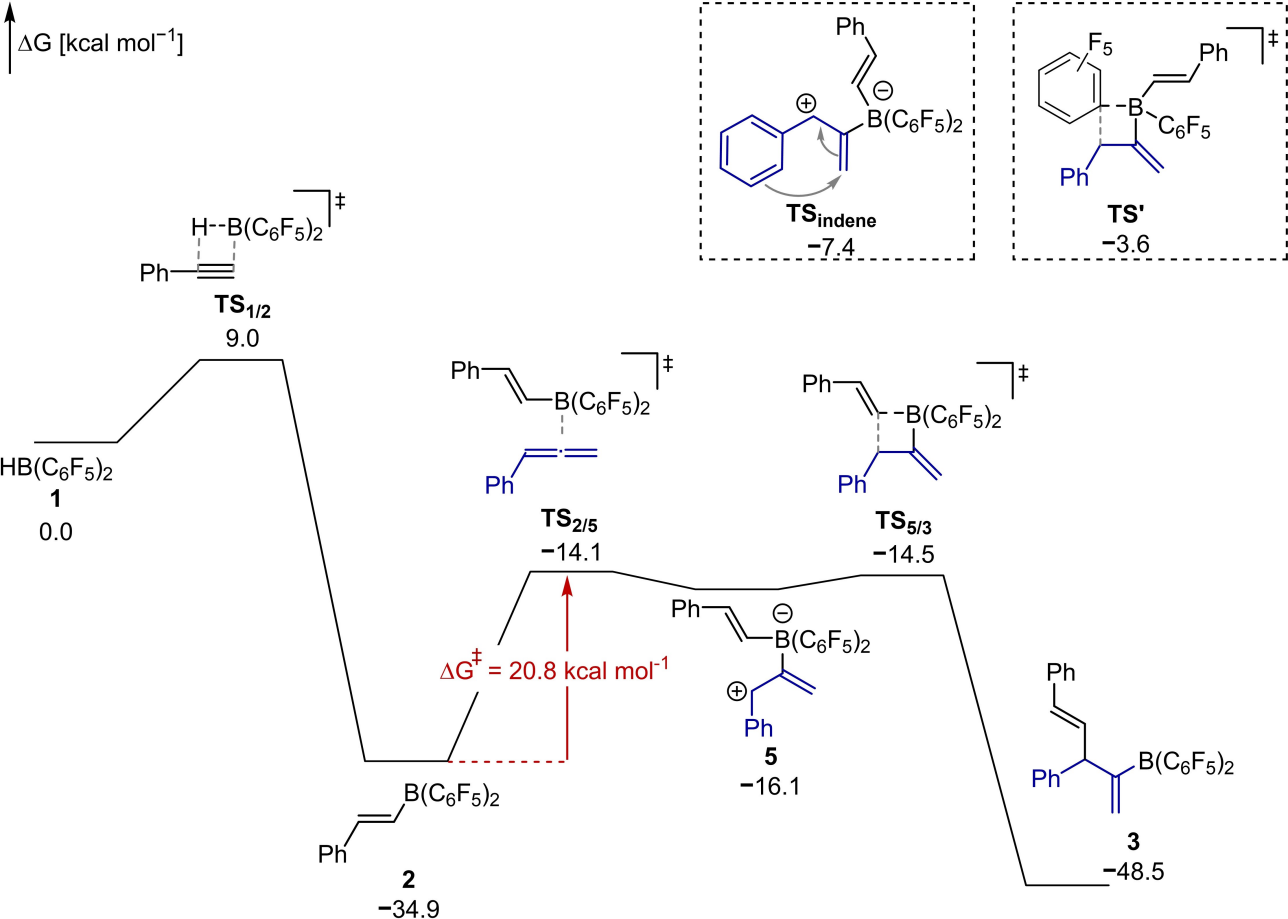
Gibbs free energies for the 1,2‐carboboration of phenylallene by the alkenylborane **2** computed at PCM(DCM)‐revDSD‐PBEP86‐D4/def2‐QZVPP//PCM(DCM)‐PBEh‐3c.

Upon heating to 60 °C in a sealed NMR tube, the formation of the 1,4‐diene **3**, which is the product of a formal 1,2‐carboboration to the internal double bond of the allene, was observed. While we have recently proposed that the Piers’ borane mediated tetramerization of alkynes commences with a carboboration of an alkyne by an alkenylborane, the carboboration of an allene by an alkenylborane is, to the best of our knowledge, unprecedented.^[4f]^ After 16 h, the yield of **3** was determined by ^1^H NMR to be 66 %. The reaction was repeated on a preparative scale in 1,2‐dichloroethane at 80 °C and the diene **3** was isolated as pyridine adduct **4** in 48 % yield by crystallization (Scheme 2). The structural assignment of **4** is further supported by single‐crystal X‐ray diffraction (SCXRD, Figure 1). With these promising initial experimental results in hand, we addressed the mechanism and the observed regioselectivity of the 1,2‐carboboration by DFT computations at the PCM(DCM)‐revDSD‐PBEP86‐D4/def2‐QZVPP//PCM(DCM)‐PBEh‐3c level of theory (Figure 2).^[8]^ The dispersion corrected, spin‐component scaled double hybrid functional revDSD‐PBEP86‐D4 was recently shown to be one of the most accurate DFT functionals by benchmark computations against the GMTKN55 database.^[8b]^ Since zwitterionic structures are likely to be part of the mechanism, the structures were optimized with an implicit solvent model for dichloromethane. According to the computations, the exergonic hydroboration of phenylacetylene by Piers’ borane **1** via **TS_1/2_** requires a Gibbs free activation energy of 9.0 kcal mol^−1^. The coordination of phenylallene to the alkenylborane **TS_2/5_** is computed to be rate determining.

Furthermore, this step dictates the regioselectivity of the carboboration. The central carbon of the allene binds to the borane so that in the zwitterion **5** the positive charge is resonance stabilized and in a benzylic and allylic position.[^2k^, ^6b^] The computed barrier of 20.8 kcal mol^−1^ for the formation of the zwitterion **5** is in favourable agreement with the mild reaction conditions. Attempts to locate minima on the potential energy surface for the two other zwitterionic regioisomers of the coordination of the allene to the borane lead to the direct dissociation of the respective structures. A virtually barrierless migration of the alkenyl substituent yields than the 1,4‐diene **3** in an exergonic reaction. In contrast, the transfer of a pentafluorophenyl ring via **TS’** has an activation barrier of 12.5 kcal mol^−1^ (see inset Figure 2). Thus, the computations agree with the experimentally observed chemoselectivity. We further considered the formation of an indene ring system by an intramolecular Friedel‐Crafts type reaction.[^2k^, ^6b^] Again, the respective transition state **TS_indene_** is kinetically disfavoured by 7.1 kcal mol^−1^ (see inset Figure 2).

**Scheme 3:**
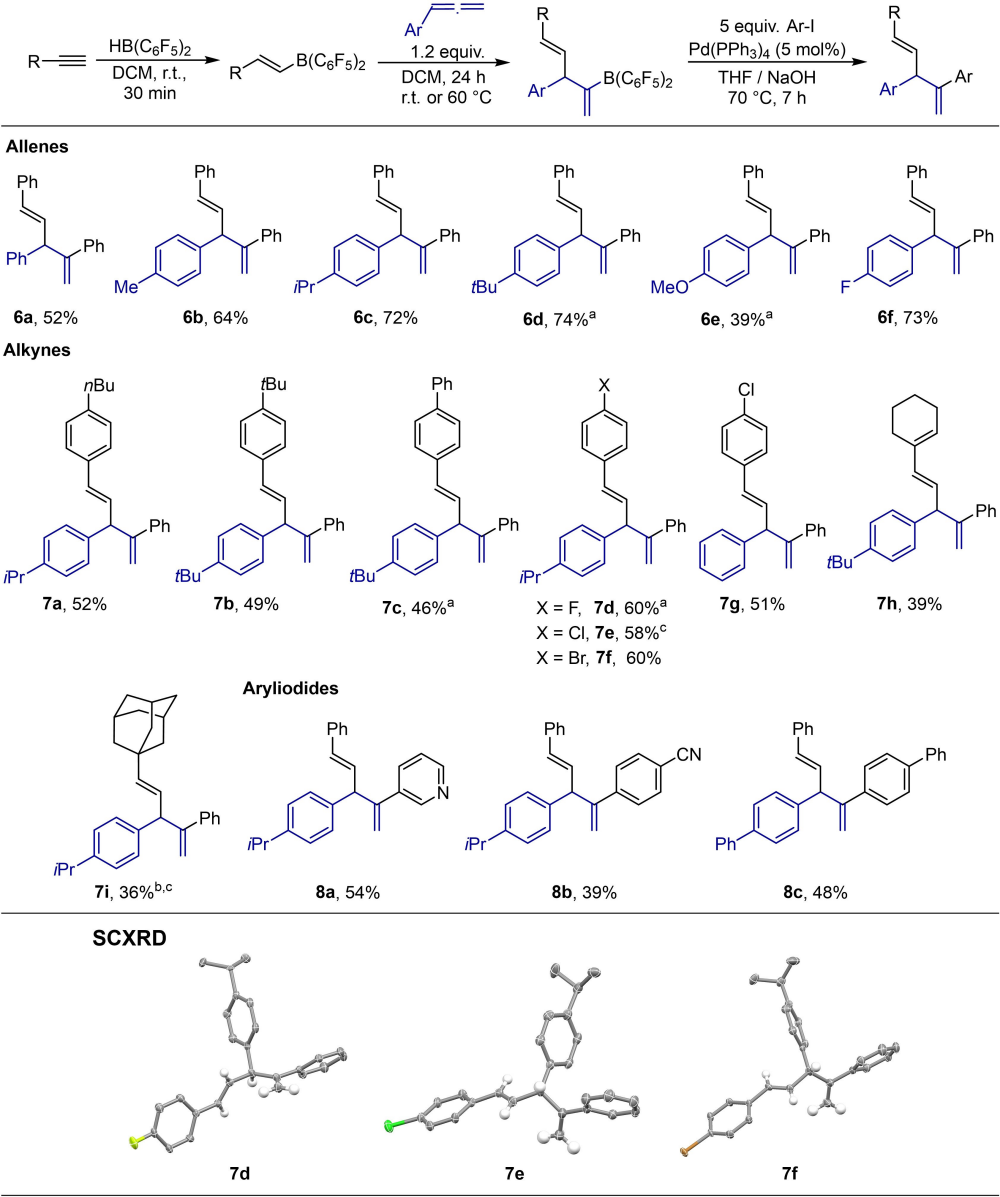
Scope of the consecutive hydroboration, carboboration, and Suzuki‐Miyaura coupling and molecular structures derived from SCXRD (50 % probability ellipsoids, for clarity only the hydrogens of the 1,4‐diene core are shown) a) The carboboration step was done at r.t. b) The reaction time for the carboboration was 3 days c) Products contained ca. 5 % impurities after column chromatography and two distillations.

Erker and co‐workers showed that B(C_6_F_5_)_2_ groups are suitable coupling partners in Suzuki‐Miyaura[^9^, ^10^] cross‐coupling reactions.[^2a^, ^2c^, ^6a^, ^11^] Therefore, we probed if it is possible to develop a one‐pot procedure for a 1,2‐carboboration of allenes by alkenylboranes and a consecutive Suzuki‐Miyaura coupling. Indeed, the reaction of **3**, formed *in situ*, with phenyliodide under basic conditions lead to the formation of the diene **6 a** in 52 % yield (Scheme 3). Notably, the reaction sequence did not require the isolation of **3**, only the solvent was removed before the Suzuki‐Miyaura coupling. This three‐step one‐pot method with the 1,2‐carboboration as key step offers a potentially broad scope. As aforementioned, a variety of alkenylboranes is accessible by hydroboration of different alkynes with Piers’ borane **1**.^[5]^ Furthermore, by adding different allenes the substitution pattern of the boryl substituted 1,4‐dienes can be easily altered. Finally, these key intermediates can be cross‐coupled with different organoiodides to access various 1,4‐dienes. 1,4‐ or skipped dienes are of synthetic interest due to their prevalence in several natural products.[^12^, ^13^] Therefore, we explored the scope of this reaction sequence (Scheme 3). First, we screened different allenes. Besides phenylallene, the corresponding *para*‐substituted methyl, *iso*‐propyl, and *tert*‐butyl phenylallenes (**6 b**–**6 d**) are suitable reaction partners. With 64 to 74 %, the yields for these substrates are better than for the unsubstituted phenylallene. Additionally, the 1,2‐carboboration of *para*‐*t*Bu‐phenylallene proceeds at r.t. while the reaction with phenylallene requires elevated temperatures. These results support the proposed mechanism: Electron donating groups stabilize the (partial) positive charge which is present in the zwitterionic intermediate and the respective transition state.^[14]^ While alcohols themselves are problematic substrates because of the strong Lewis acidic character of the boron moiety, protected alcohols like methyl aryl ethers are tolerated, as it was demonstrated by the synthesis of **6 e** in 39 % yield.^[15]^ The carboboration and coupling of *para*‐fluorophenylallene gives the respective diene **6 f** in 73 % yield. We then tested different alkynes. The reaction with alkyl‐ and phenyl‐substituted phenylacetylenes gives the dienes **7 a**–**7 c** in moderate yields. Furthermore, alkynes with electron withdrawing groups like fluorine, chlorine, and bromine can also be used as coupling partners with yields between 51–60 % (**7 d**–**7 g**). To elucidate whether aliphatic alkynes are suitable substrates for the carboboration, we reacted ethynyl‐cyclohexene and adamantylacetylene with Piers’ borane and *p*‐*t*Bu‐phenylallene and *p*‐*i*Pr‐phenylallene, respectively. Subsequent coupling with phenyliodide gave **7 h** and **7 i** in 39 % and 36 % yield. The synthesis of **7 h** demonstrates that double bonds are tolerated during the carboboration. Additionally, several organoiodides were tested. The reaction with 3‐iodopyridine as an example for a heterocycle gave diene **8 a** in 54 % yield. The reaction with 4‐iodobenzonitrile and biphenyliodide gave the respective products **8 b** and **8 c** in 39 % and 48 % yield, respectively.

**Scheme 4:**
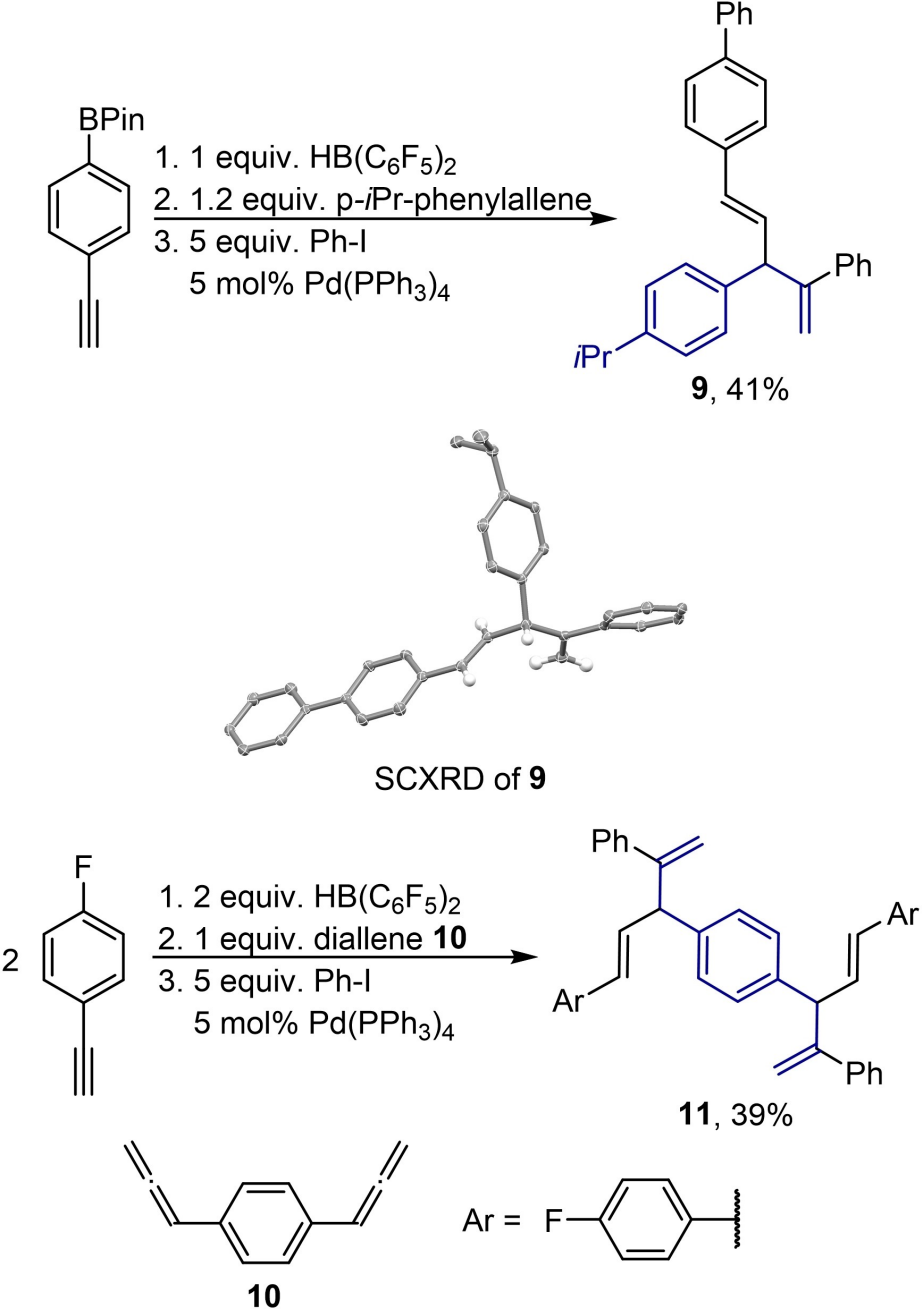
Special substrates of the hydroboration, carboboration, and Suzuki‐Miyaura coupling sequence and the molecular structure of diene **9** derived from SCXRD (50 % probability ellipsoids, for clarity only the hydrogens of the 1,4‐diene core are shown).

The reaction of commercially available *para*‐Bpin substituted phenylacetylene with *p*‐*i*Pr‐phenylallene under the standard conditions led to the replacement of both boron groups upon coupling with phenyliodide. The respective product **9** with four aromatic rings was isolated in 41 % yield (Scheme 4). The hydroboration of two equivalents of *para*‐F‐phenylacetylene, the addition of one equivalent of diallene **10**, and a subsequent Suzuki‐Miyaura coupling yielded the phenyl bridged bis‐1,4‐diene **11** in 39 % (Scheme 4). Products **9** and **11** demonstrate that complex molecular frameworks can be synthesized from non‐ or low‐functionalised starting materials using the protocol presented herein. The structure of the dienes **7 d**, **7 e**, **7 f**, and **9** was additionally supported by SCXRD (Schemes 3 and 4). Regarding the limitations of this method, we observed that the internal alkynes 3‐hexyne and diphenylacetylene are readily hydroborated by **1** but the resulting alkenylboranes did not react with phenylallene. We further found that alkynes with Lewis basic sides such as ethynyl phenylmethyl ether and 2‐ethynylthiophene are not suitable for this reaction sequence. Furthermore, the carboboration of cyclohexylallene with **2** was not successful. This is probably because of the lacking stabilization of the positive charge in the rate‐determining transition state and the zwitterionic intermediate.

## Conclusion

In summary, we report a novel and regioselective 1,2‐carboboration as the key step of a three‐step one‐pot protocol for the synthesis of aryl‐substituted 1,4‐dienes from alkynes, allenes, and organoiodides. This unprecedented 1,2‐carboboration transfers a vinyl group of an *in situ* generated alkenylborane to the benzylic position of the aryl allene. All three components of the reaction sequence that forms two C−C bonds can be modified. Therefore, a variety of aryl‐substituted 1,4‐dienes is accessible by this modular approach. The usefulness of this protocol was demonstrated by the synthesis of twenty different dienes. We expect this finding to stimulate the development of new applications of carboboration reactions for organic synthesis.

## Crystal structures

Deposition Number(s) 2150152 (for 4 at 100 K), 2150153 (for 7e at 100 K), 2150154 (for 7d at 100 K), 2150155 (for 9 at 100 K), and 2150156 (for 7 f at 100 K) contain(s) the supplementary crystallographic data for this paper. These data are provided free of charge by the joint Cambridge Crystallographic Data Centre and Fachinformationszentrum Karlsruhe Access Structures service.

## Conflict of interest

The authors declare no conflict of interest.

1

## Supporting information

As a service to our authors and readers, this journal provides supporting information supplied by the authors. Such materials are peer reviewed and may be re‐organized for online delivery, but are not copy‐edited or typeset. Technical support issues arising from supporting information (other than missing files) should be addressed to the authors.

Supporting Information

## Data Availability

The data that support the findings of this study are available in the supplementary material of this article.
