# Supplementary material for: 1,2‐Carboboration of Arylallenes by In Situ Generated Alkenylboranes for the Synthesis of 1,4‐Dienes
Source: Chemistry. 2022 Apr 21;28(32):e202200470. doi: 10.1002/chem.202200470 (PMC9325554; doi:10.1002/chem.202200470)
Supplement: Supplementary file 1 — Supporting Information [file CHEM-28-0-s001.pdf]

Chemistry–A European Journal

Supporting Information

1,2-Carboboration of Arylallenes by In Situ Generated Alkenylboranes for the Synthesis of 1,4-Dienes

Arthur Averdunk, Max Hasenbeck, Tizian Müller, Jonathan Becker, and Urs Gellrich*

1 Table of contents

2 General Specifications 4

3 Synthesis of alkynes 4

4 Synthesis of monoallenes 5

4.1 Wittig reaction 5

4.2 Doering-LaFlamme reaction 5

5 Synthesis of diallene **10** 6

5.1 Wittig reaction 6

5.2 Doering-LaFlamme reaction 6

5.2.1 Cyclopropantion of *para*-vinylstyrene 7

5.2.2 Synthesis of 1,4-(bis-propa-1,2-diene)-benzol **10** 13

6 Initial NMR experiment of bis(pentafluorophenyl)((*E*)-2-phenylethenyl)borane **2** with phenylallene 16

7 Synthesis and characterization of (*E*)-(3,5-diphenylpenta-1,4-dien-2-yl)borane **3** 22

7.1 Additional NMR spectra of (*E*)-(3,5-diphenylpenta-1,4-dien-2-yl)borane 23

8 Synthesis and characterization of (*E*)-(3,5-diphenylpenta-1,4-dien-2-yl)borane pyridine adduct **4** 31

8.1 Additional NMR spectra of (*E*)-(3,5-diphenylpenta-1,4-dien-2-yl)borane pyridine adduct **4** 32

9 Synthesis and characterization of 1,4-diene products 39

9.1 General procedure 39

9.2 Purification and characterization of 1,4-diene products 39

9.3 Additional NMR spectra of 1,4-diene products 59

9.3.1 1,4-diene **6a** 59

9.3.2 1,4-diene **6b** 66

9.3.3 1,4-diene **6c** 73

9.3.4 1,4-diene **6d** 80

9.3.5 1,4-diene **6e** 87

9.3.6 1,4-diene **6f** 94

9.3.7 1,4-diene **7a** 102

9.3.8 1,4-diene **7b** 109

9.3.9 1,4-diene **7c** 116

9.3.10 1,4-diene **7d** 123

9.3.11 1,4-diene **7e** 131

9.3.12 1,4-diene **7f** 138

9.3.13 1,4-diene **7g** 145

9.3.14 1,4-diene **7h** 152
9.3.15 1,4-diene **7i** 159
9.3.16 1,4-diene **8a** 166
9.3.17 1,4-diene **8b** 173
9.3.18 1,4-diene **8c** 180
9.3.19 1,4-diene **9** 187
9.3.20 Bis-1,4-diene **11** 194
10 Single crystal X-ray diffraction 205
10.1 Pyridine adduct **4** 205
10.2 1,4-diene **7d** 213
10.3 1,4-diene **7e** 220
10.4 1,4-diene **7f** 228
10.5 1,4-diene **9** 236
11 Comparison between computed and experimental NMR shifts of 1,4-diene **3** 243
12 Computational details 243
12.1 NMR computations 244
12.2 Cartesian coordinates and energies. 245
13 Reference 267

2 General Specifications

All manipulations with air and moisture sensitive compounds were carried out under a nitrogen atmosphere using standard Schlenk and glovebox techniques (nitrogen glovebox, mBraun Uni Lab plus).

$B(C_6F_5)_3$ was synthesized from boron trifluoride etherate according to literature procedures.[1]

Piers' borane $HB(C_6F_5)_2$ was synthesized from $B(C_6F_5)_3$ according to literature procedure.[2]

Iodobenzene, 3-iodopyridine, 4-iodobenzonitril, and 4-iodobiphenyl were purchased commercially.

Pyridine was purchased commercially, distilled over CaH_2 under inert conditions, and stored in the glovebox over molecular sieves.

All dry, non-deuterated solvents were if commercially available purchased by Acros Organics or Sigma Aldrich in a sealed bottle with a septum and stored (except benzene) over molecular sieves.

Deuterated solvents were distilled under inert conditions or freeze pump thawed three times and kept in the glovebox over 4 Å molecular sieves.

NMR spectra were recorded on Bruker Avance II 200 MHz, Bruker Avance III HD 400 MHz, Bruker Avance II 400 MHz, Bruker Avance III HD 600 MHz, and Avance Neo 700 MHz spectrometers. ^{1}H and ^{13}C NMR chemical shifts are referenced to residual solvent resonance peaks or tetramethylsilane.

Mass spectra were recorded on an ESI-MS-Bruker Mikro-TOF mass spectrometer. For ESI measurements in the positive ion detection mode the capillary current was set to 4500 V with the end plate offset of –500 V. For APCI measurements in the positive ion detection mode the capillary current was set to 1500 V with the end plate offset of -500 V.

The HPLC chromatogram was recorded using a chiralpak IA column with a 97:3 *n*-hexane/*iso*-propanol mixture, a flow of 1 mL/min, and a UV detector at 270 nm.

3 Synthesis of alkynes

The alkynes *p*-*i*Pr-phenylacetylene and *p*-F-phenylacetylene were synthesized using the Corey-Fuchs reaction starting from the corresponding aldehydes analogously to literature known procedures (Scheme SI 1).[3] The spectral data are identical to the literature.[4]

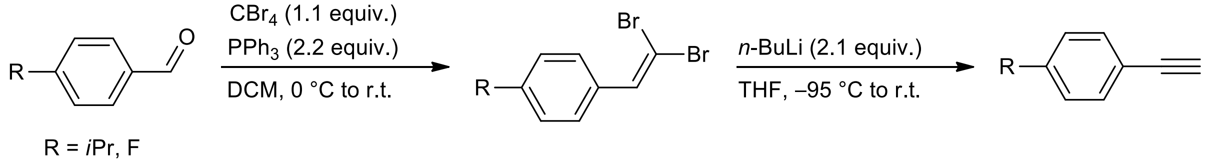

Scheme SI 1: General approach for the synthesis of *para*-*iso*-propyl- and *para*-fluorophenylacetylene from the corresponding aldehydes *via* a Corey-Fuchs reaction.

1-Adamantylacetylene was synthesized starting from 1-bromoadamantane in a two-step procedure (Scheme SI 2).[5]

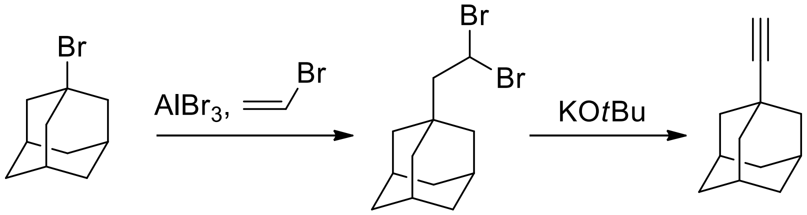

Scheme SI 2: Synthesis of 1-adamantylacetylene from 1-bromoadamantane.

Phenylacetylene, *p*-Ph-phenylacetylene, *p*-Cl-phenylacetylene, *p*-BPin-phenylacetylene, *p*-Br-phenylacetylene, *p*-*t*Bu-phenylacetylene, *p*-*n*bu-phenylacetylene, and *p*-Me-phenylacetylene were purchased commercially.

4 Synthesis of monoallenes

4.1 Wittig reaction

The olefins para-iso-propylstyrene, *p*ara-phenylstyrene and *para*-fluorostyrene were synthesized using a Wittig reaction starting from the corresponding aldehydes analogously to literature known procedures (Scheme SI 3).[6] The spectral data of the styrene derivatives are identical to the literature.[6,7]

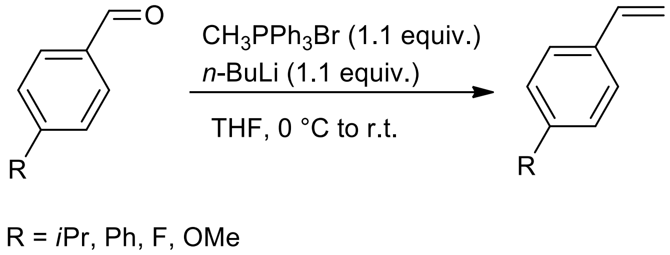

Scheme SI 3: General approach for the synthesis of *para-iso*-propyl-, *para*-phenyl-, *para*-fluoro-, and *para*-methoxystyrene from the corresponding aldehydes *via* a Wittig reaction

4.2 Doering-LaFlamme reaction

Phenylallene, *para*-methylphenylallene, *para-iso*-propylphenylallene, *para-tert*butylphenylallene, *para*-phenylphenylallene, *para*-fluorophenylallene and *p*ara-methoxyphenylallene were synthesized according to reported procedures using the Doering-LaFlamme reaction starting from the corresponding olefins (Scheme SI 4).[8] The spectral data of the styrene derivatives are identical with the literature.[8b,9] Styrene, *para*-methylstyrene, *para-tert*-butylstyrene were purchased commercially.

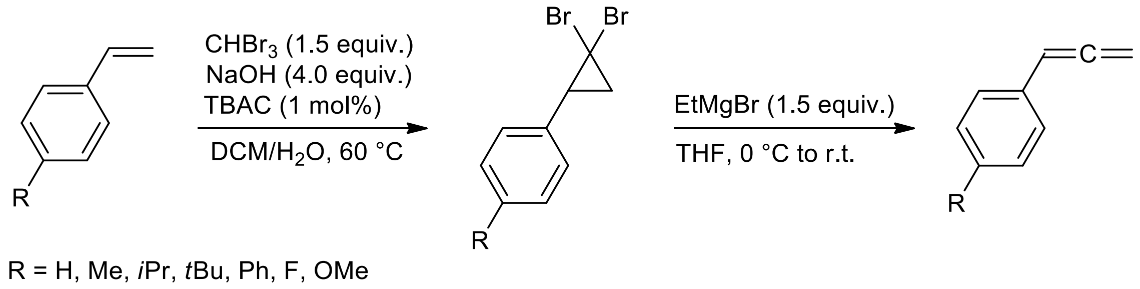

Scheme SI 4: General approach for the synthesis of phenylallene, *para-iso*-propyl-, *para*-phenyl-, *para*-fluoro-, *para*-methoxystyrene, *para-tert*-butyl-, and *para*-methylphenylallene from the corresponding styrene derivatives via a Doering-LaFlamme reaction.

5 Synthesis of diallene 10

5.1 Wittig reaction

para-Vinylstyrene was synthesized using a Wittig reaction starting from the corresponding dialdehyde analogously to literature known procedures (Scheme SI 5).[6] The spectral data are identical to the literature.[10]

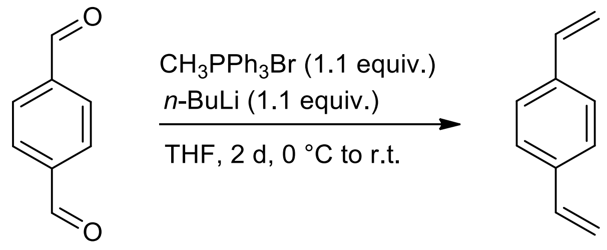

Scheme SI 5: Synthesis of *para*-vinylstyrene from the corresponding aldehyde via a Wittig reaction.

5.2 Doering-LaFlamme reaction

1,4-(bis-propa-1,2-diene)-benzene was synthesized using the Doering-LaFlamme reaction starting from *para*-vinylstyrene (Scheme SI 6).[8] The spectral data are identical to the literature.[11]

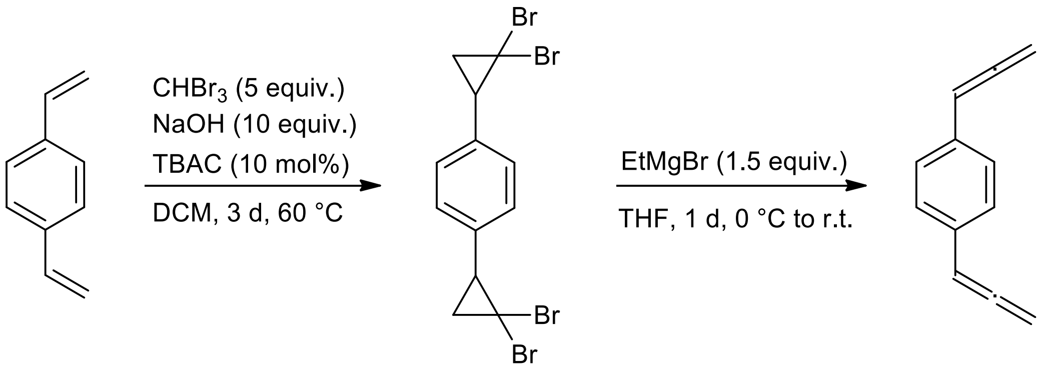

Scheme SI 6: Synthesis of 1,4-(bis-propa-1,2-diene)-benzene starting from *para*-vinylstyrene via a Doering-LaFlamme reaction.

5.2.1 Cyclopropantion of *para*-vinylstyrene

In a three-necked flask equipped with a reflux condenser and a dropping funnel, *para*-vinylstyrene (1.4 g, 10.8 mmol, 1.0 equiv.), bromoform (13.6 g, 53.8 mmol, 5.0 equiv.) and triethylbenzylammonium chloride (0.24 g, 1.075 mmol, 0.10 equiv.) were stirred in DCM (50 mL) vigorously at 0 °C (ice bath). Under a nitrogen atmosphere, aqueous NaOH (10 ml, 11 M) was added dropwise. After completed addition, the reaction mixture was heated to 60 °C for 3 d. The mixture was diluted with water, extracted three times with DCM, and dried over $MgSO_4$. The solvent was removed under reduced pressure and the crude product was purified by silica gel column chromatography (*n*-hexane) to yield the cyclopropanation product 1,4-(bis-(2,2-dibromocyclopropyl))-benzene as yellowish oil (3.89 g, 8.21 mmol, 76 %).

Remark: The product was isolated as a mixture of diastereomers.

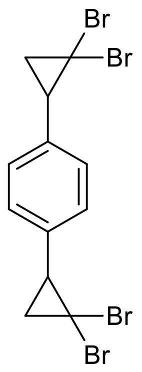

^{1}H NMR (400 MHz, chloroform-*d*) δ 7.25 (s, 4H, Ar-*H*), 2.95 (ddd, *J* = 10.5, 8.3, 1.5 Hz, 1H, C*H*), 2.15 (ddd, *J* = 10.5, 7.8, 0.8 Hz, 1H, CH_2), 2.02 (td, *J* = 8.0, 2.5 Hz, 1H, CH_2).

^{13}C NMR (101 MHz, chloroform-*d*) δ 135.54 (Ar-C_q), 128.96 (d, *J* = 1.5 Hz, Ar-*C*), 35.81 (d, *J* = 1.5 Hz, *C*H), 28.39 (d, *J* = 3.6 Hz, *C*Br_2), 27.50 (*C*H_2).

5.2.1.1 Additional NMR spectra of 1,4-(bis-(2,2-dibromocyclopropyl))-benzene

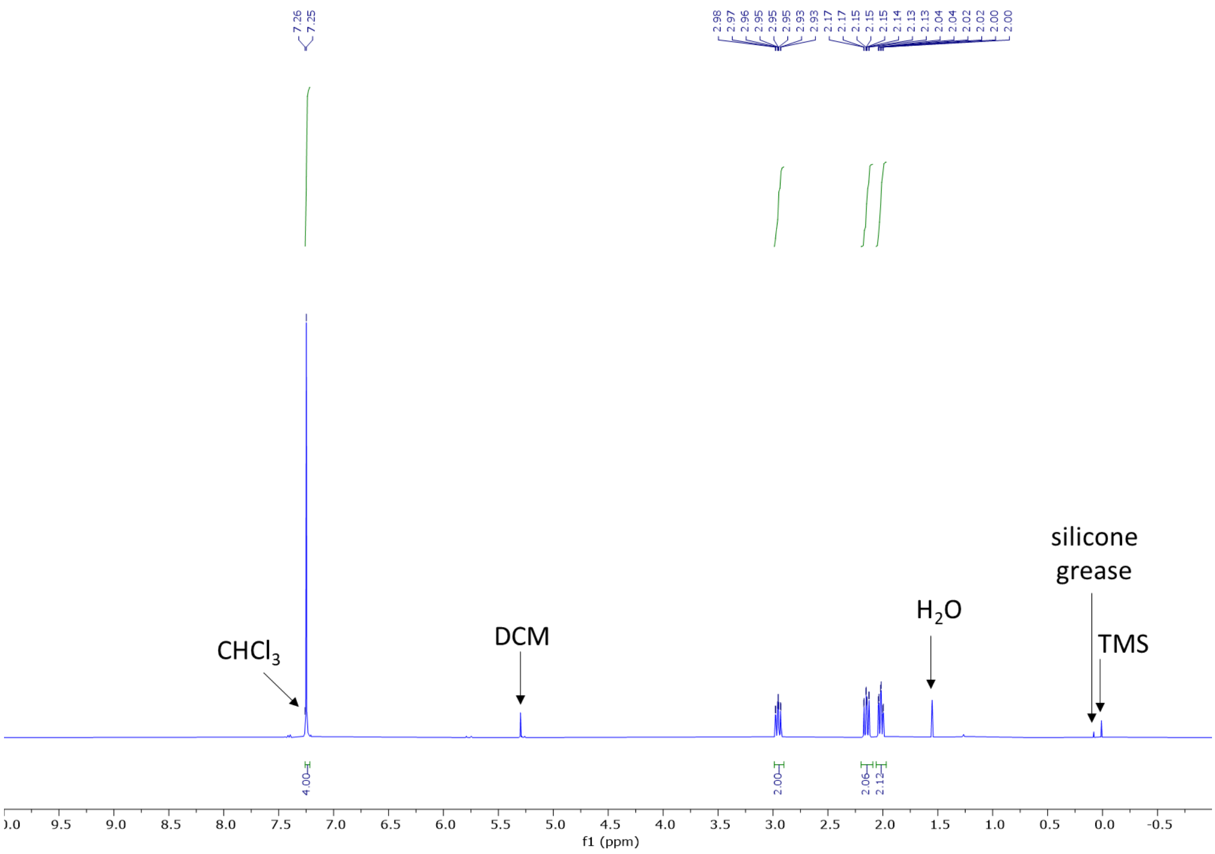

Figure SI 1: ^{1}H NMR spectrum of 1,4-(bis-(2,2-dibromocyclopropyl))-benzene (400 MHz, chloroform-*d*).

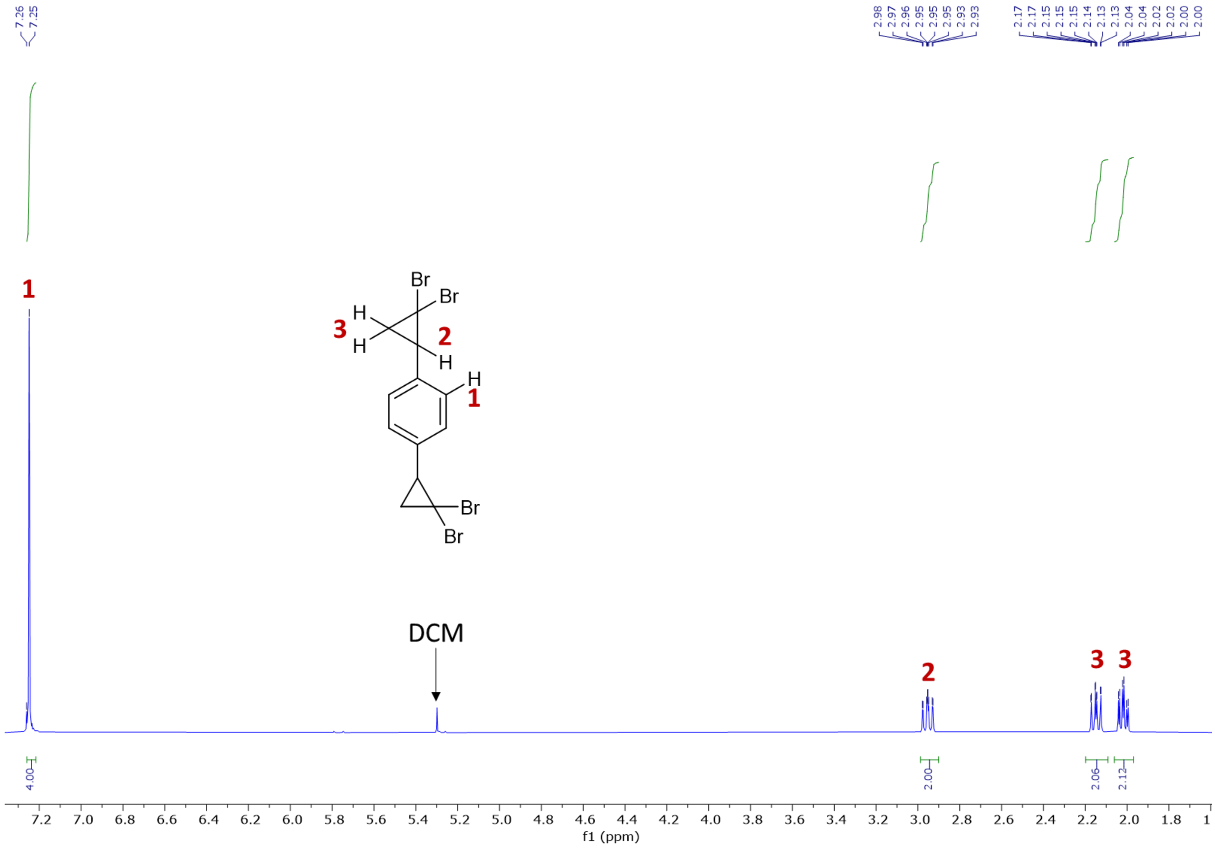

Figure SI 2: Excerpt of the ^{1}H NMR spectrum of 1,4-(bis-(2,2-dibromocyclopropyl))-benzene (400 MHz, chloroform-*d*).

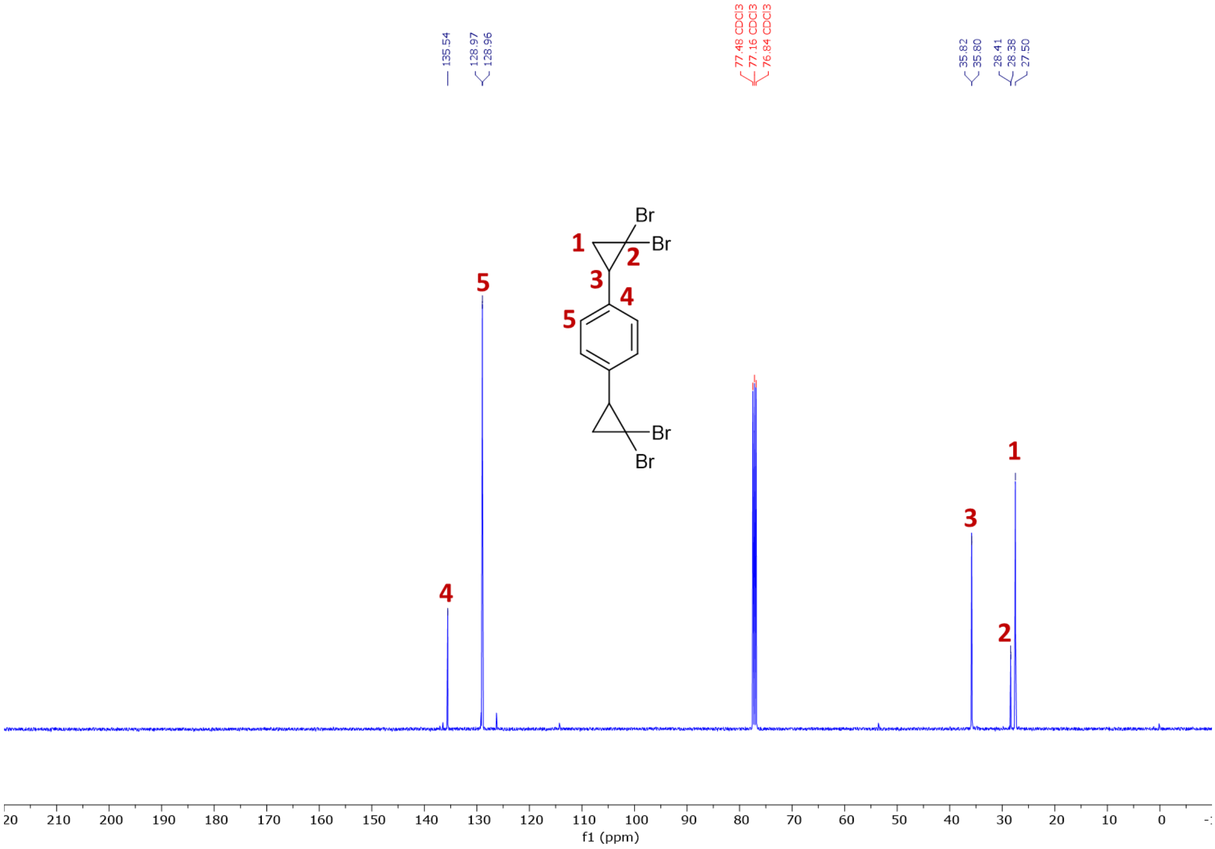

Figure SI 3: ^{13}C NMR spectrum of 1,4-(bis-(2,2-dibromocyclopropyl))-benzene (101 MHz, chloroform-*d*).

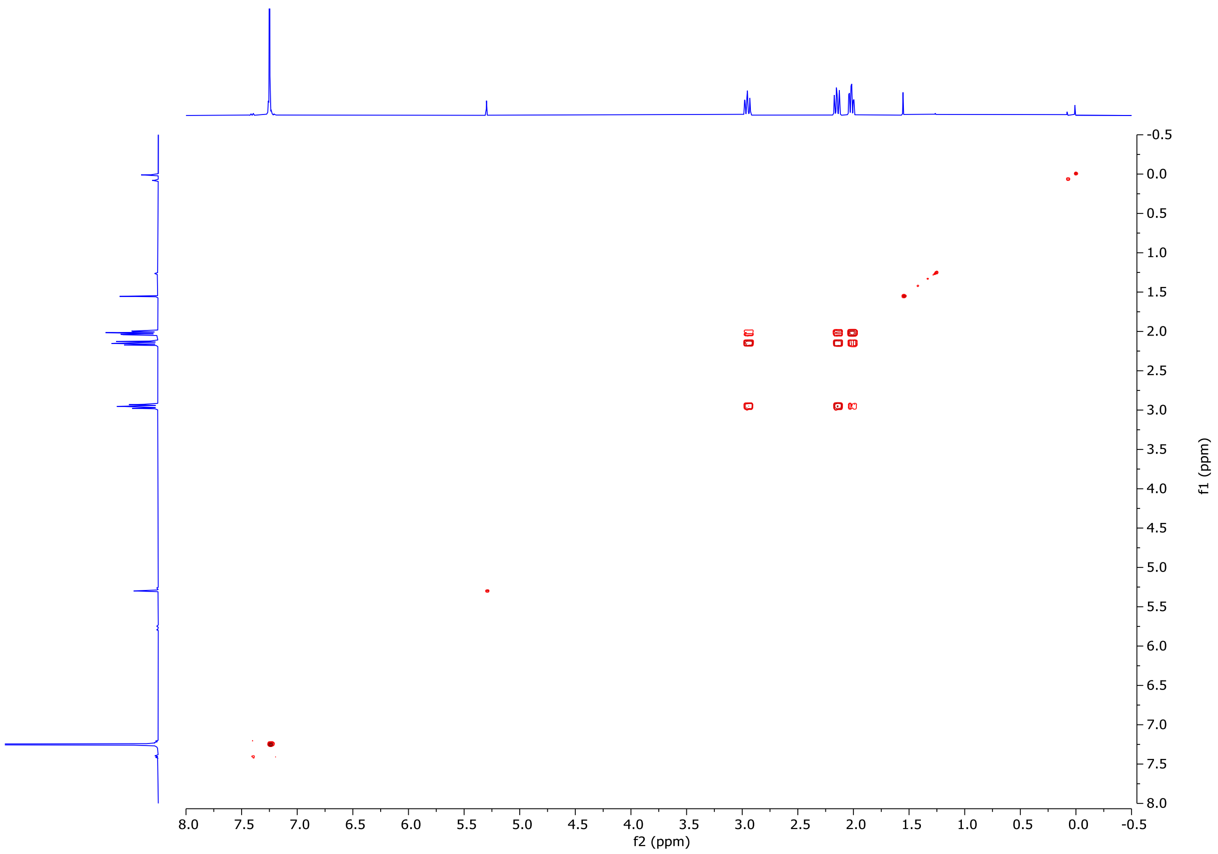

Figure SI 4: HH COSY NMR spectrum of 1,4-(bis-(2,2-dibromocyclopropyl))-benzene (400 MHz, chloroform-*d*).

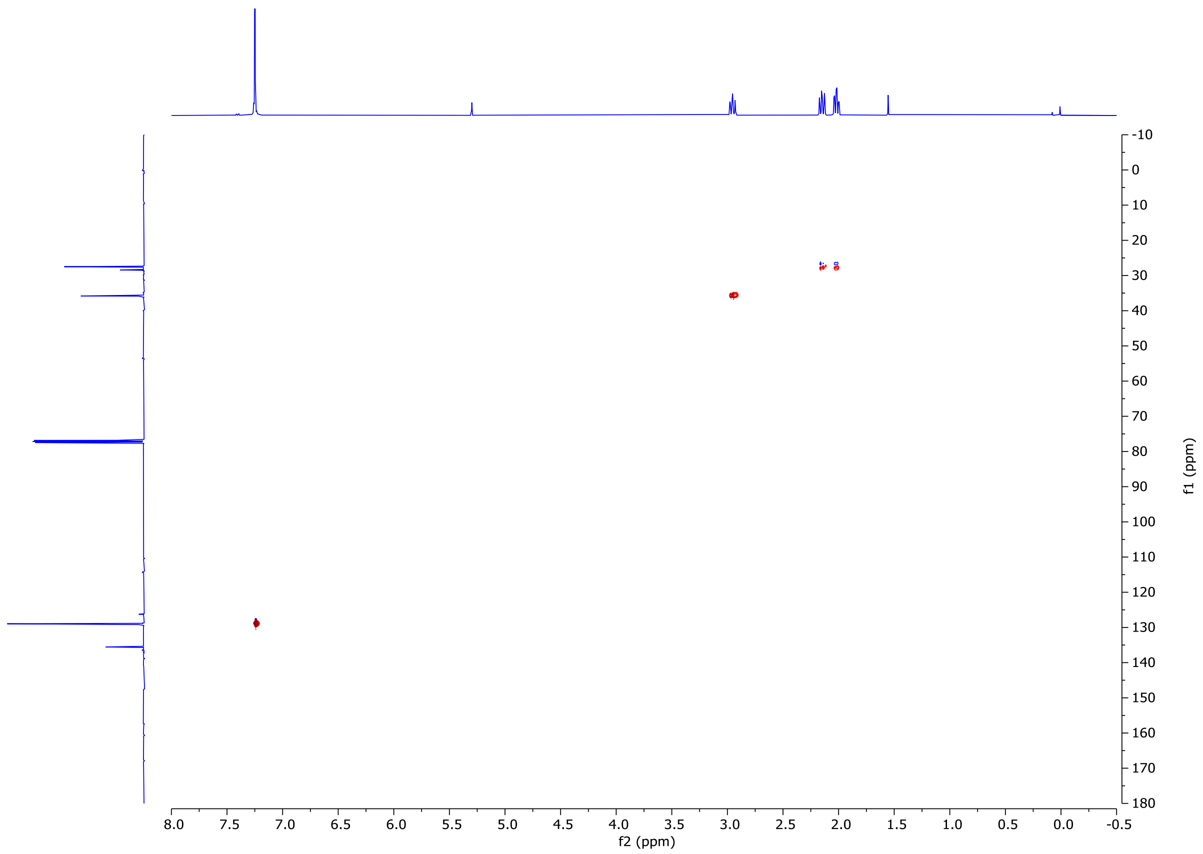

Figure SI 5: $^{1}H^{13}C$ HSQC NMR spectrum of 1,4-(bis-(2,2-dibromocyclopropyl))-benzene (100 MHz, chloroform-*d*).

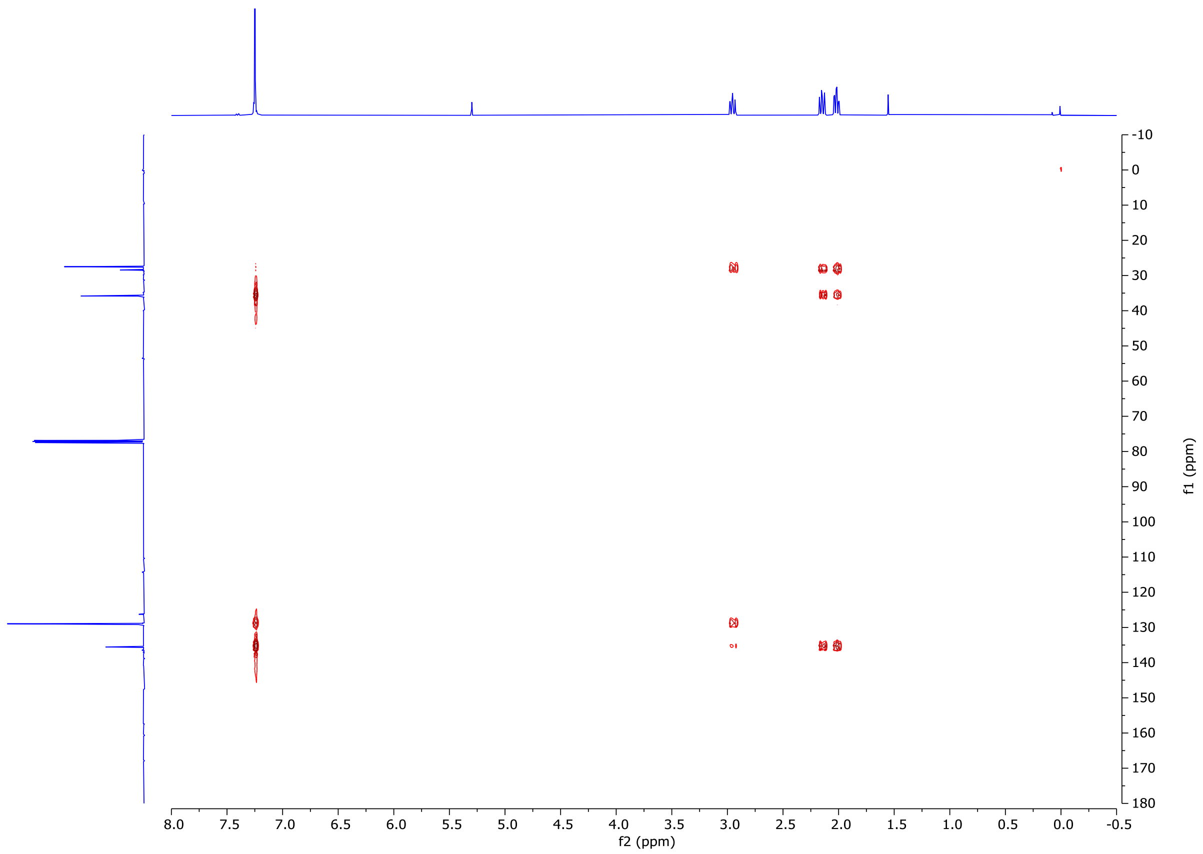

Figure SI 6: $^{1}H^{13}C$ HMBC NMR spectrum of 1,4-(bis-(2,2-dibromocyclopropyl))-benzene (100 MHz, chloroform-*d*).

5.2.2 Synthesis of 1,4-(bis-propa-1,2-diene)-benzol 10

In a flame-dried three-necked-flask equipped with a refluxing condenser and a dropping funnel, a solution of 1,4-(bis-(2,2-dibromocyclopropyl))-benzene (3.9 g, 8.2 mmol,1.0 equiv.) in dry THF (50 mL) was stirred vigorously. Under a nitrogen atmosphere, a solution of MeMgBr (3 M in THF, 41.1 mmol, 5.0 equiv.) was added dropwise at 0 °C (ice bath). The reaction mixture was warmed to room temperature and stirred for 1 d. The mixture was quenched carefully with water at 0 °C, extracted three times with *n*-pentane and dried over $MgSO_4$. The solvent was removed under reduced pressure and the crude product was purified by silica gel column chromatography (*n*-hexane). Inside the glovebox, the product is crystallized from a saturated solution in DCM at room temperature giving the product 1,4-(bis-(2,2-dibromocyclopropyl))-benzene **10** as yellow crystals (0.30 g, 1.95 mmol, 24 %).

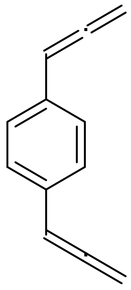

^{1}H NMR (400 MHz, chloroform-*d*) δ 7.24 (s, 4H, Ar-*H*), 6.15 (t, *J* = 6.7 Hz, 1H, C*H*), 5.15 (d, *J* = 6.7 Hz, 2H, C*H*$_2$).

^{13}C NMR (101 MHz, chloroform-*d*) δ 210.08 (CH*C*CH$_2$), 132.67 (Ar-*C*$_q$), 127.10 (Ar-*C*), 93.94 (*C*H), 79.02 (*C*H$_2$).

5.2.2.1 *Additional NMR spectra of diallene 10*

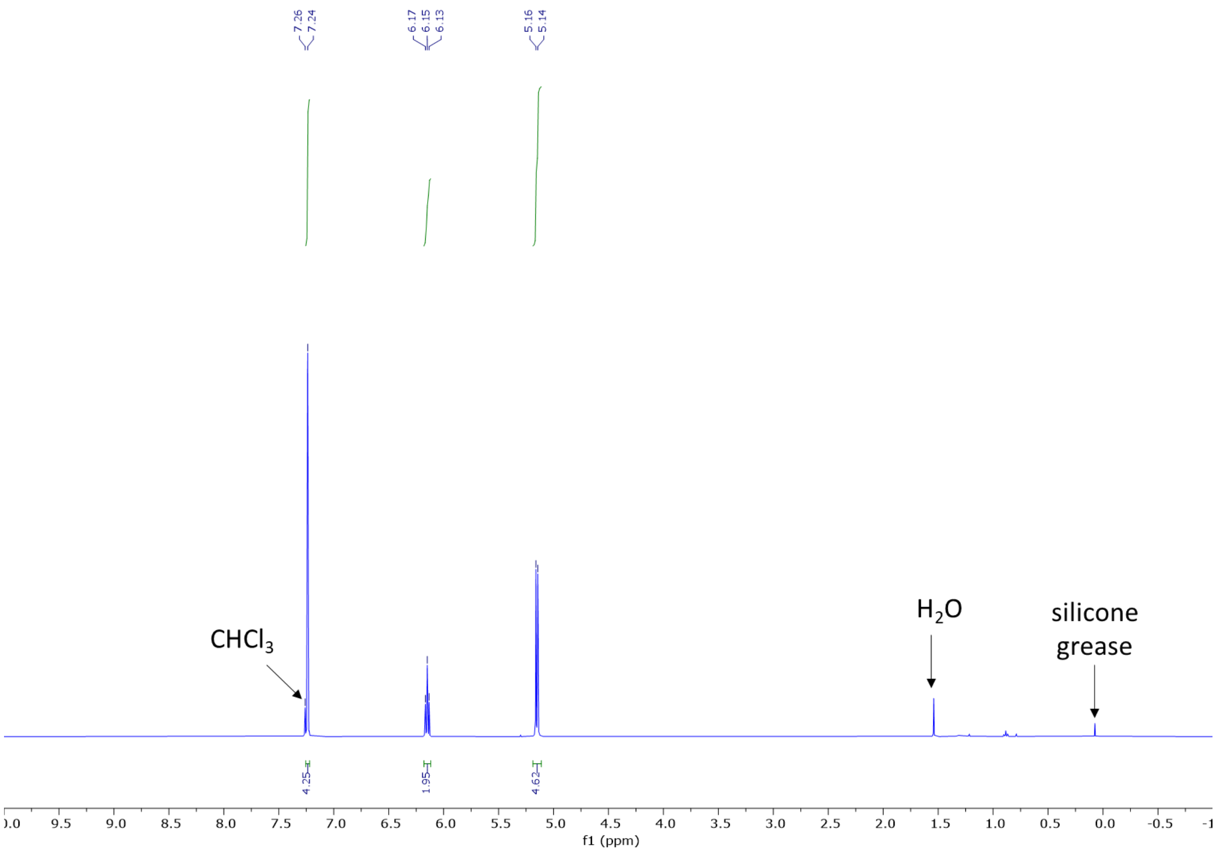

Figure SI 7: ^{1}H NMR spectrum of 1,4-(bis-(2,2-dibromocyclopropyl))-benzene **10** (400 MHz, chloroform-*d*).

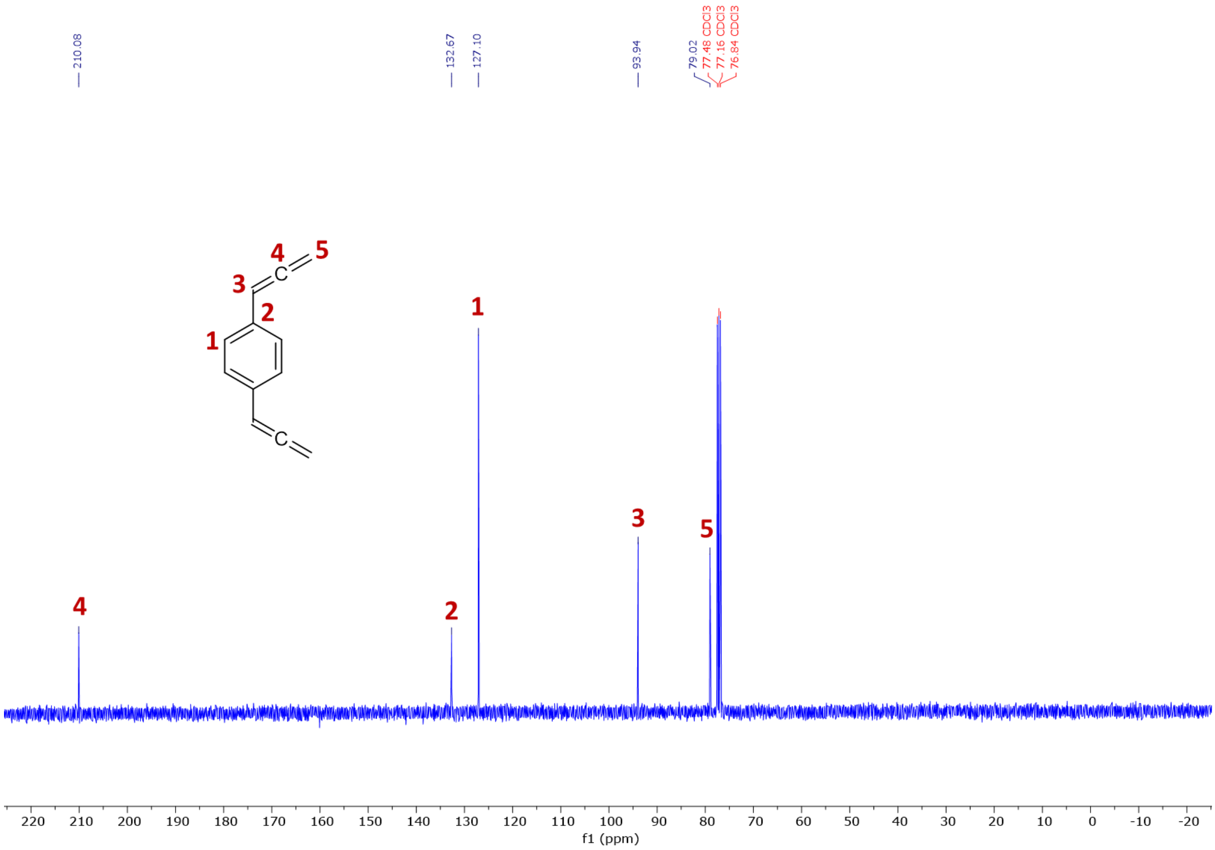

Figure SI 8: ^{13}C NMR spectrum of 1,4-(bis-(2,2-dibromocyclopropyl))-benzene **10** (101 MHz, chloroform-*d*).

6 Initial NMR experiment of bis(pentafluorophenyl)((*E*)-2-phenylethenyl)borane 2 with phenylallene

Inside the glovebox, phenylacetylene (5.5 µL, 0.05 mmol, 1.0 equiv.) and Piers' borane (17.3 mg, 0.05 mmol, 1.0 equiv.) were dissolved in DCM-d_2 (0.4 mL) in an NMR tube with J Young valve and left at room temperature for about 10 min. Afterwards, a ^{1}H and ^{11}B NMR spectrum were measured. The ^{1}H and ^{11}B NMR spectra show that all phenylacetylene had been hydroborated to the literature known bis(pentafluorophenyl)-((*E*)-2-phenylethenyl)borane **2** (Figure SI 9, Figure SI 10).[12]

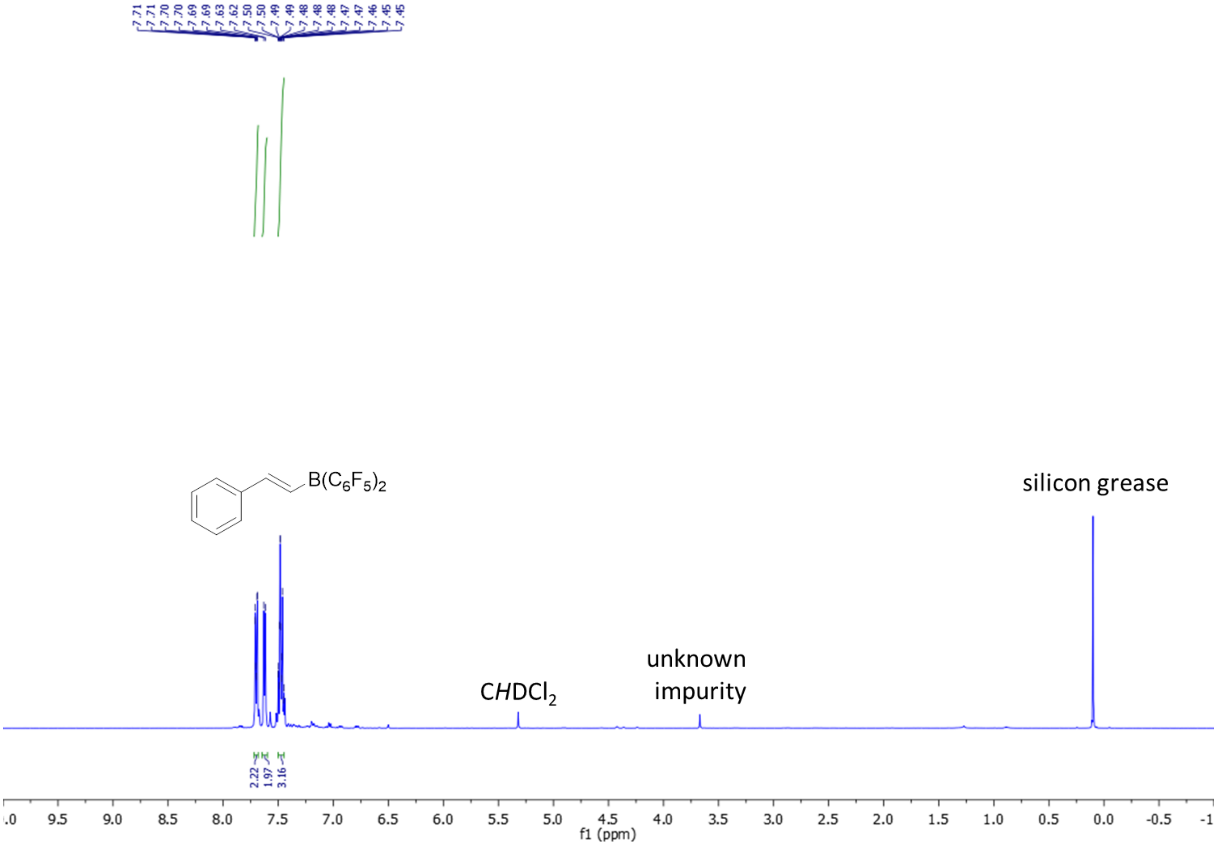

Figure SI 9: ^{1}H NMR spectrum of the hydroboration product of phenylacetylene and Piers' borane, bis(pentafluorophenyl)-((*E*)-2-phenylethenyl)borane **2** (400 MHz, DCM-d_2).

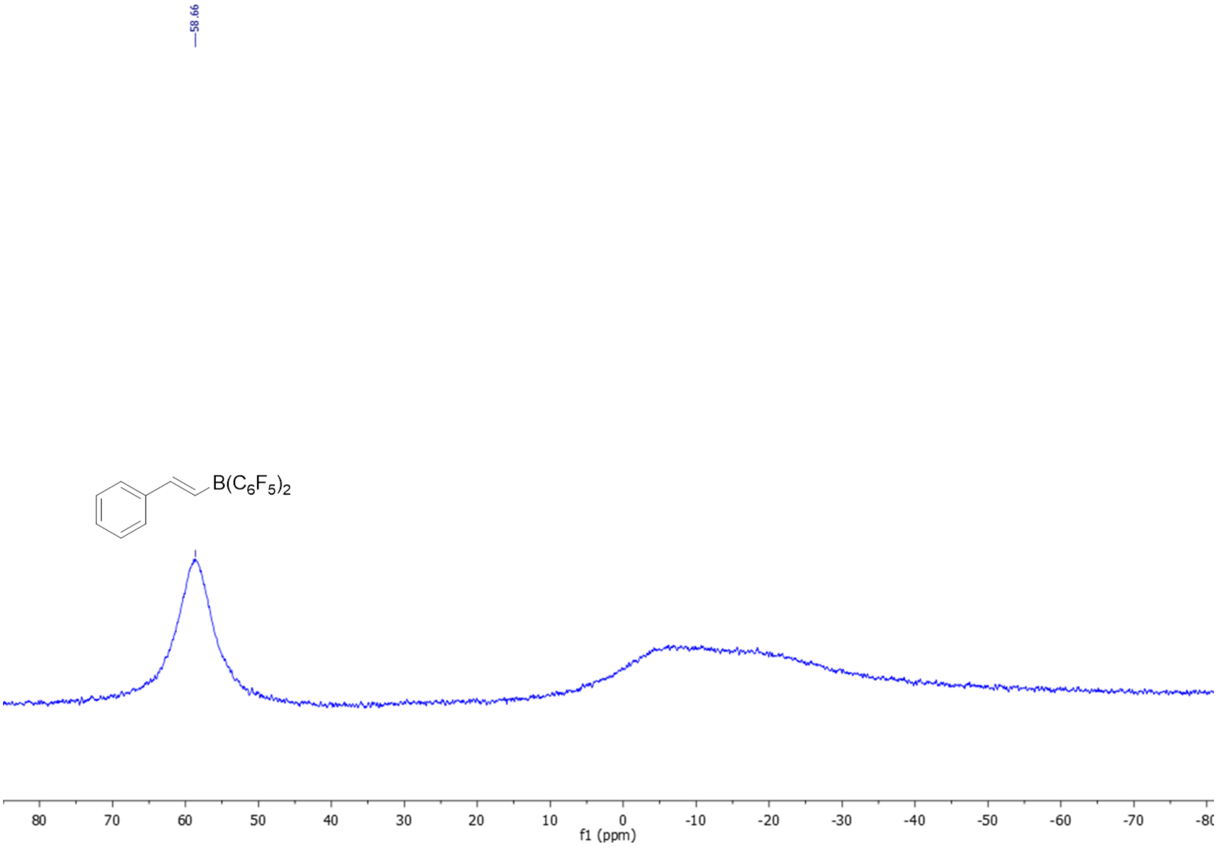

Figure SI 10: ^{11}B NMR spectrum of the hydroboration product of phenylacetylene and Piers' borane, bis(pentafluorophenyl)((*E*)-2-phenylethenyl)borane) **2** (128 MHz, DCM-d_2).

The NMR tube was brought into the glovebox and phenylallene (6.4 µL, 0.05 mmol, 1.0 equiv.) was added. The tube was taken out of the glovebox and was heated to 60 °C in an oil bath for 16 h.

Afterwards ^{1}H and ^{11}B NMR spectra were measured. The spectra show that most of the phenylallene disappeared and one new main set of signals appeared which can be assigned to the 1,2-carboboration product of the bis(pentafluorophenyl)-((*E*)-2-phenylethenyl)borane and phenylallene, (*E*)-(3,5-diphenylpenta-1,4-dien-2-yl)borane **3** (Figure SI 11, Figure SI 12) (For a complete NMR characterization see chapter 7).

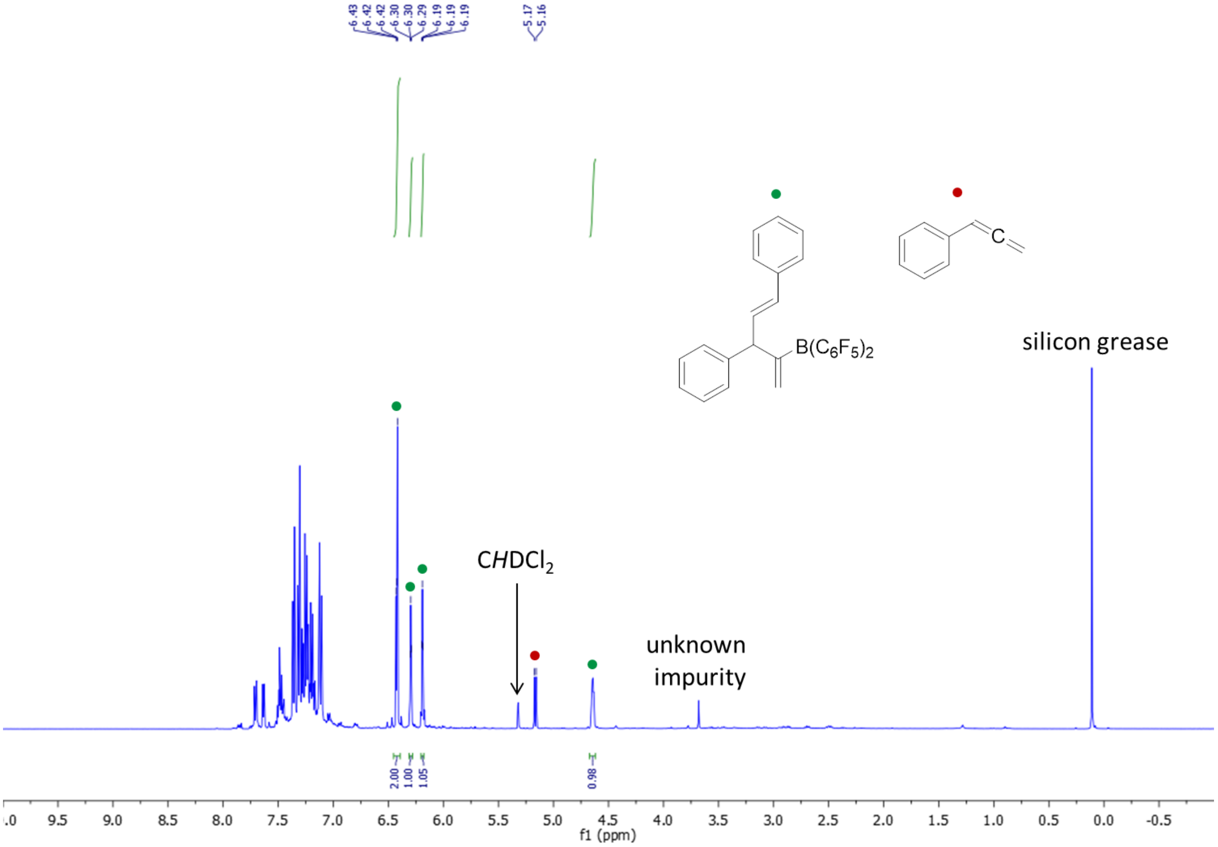

Figure SI 11: ^{1}H NMR spectrum of the 1,2,-carboboration product **3** of bis(pentafluorophenyl)-((*E*)-2-phenylethenyl)borane with phenylallene, (*E*)-(3,5-diphenylpenta-1,4-dien-2-yl)borane (400 MHz, DCM-d_2).

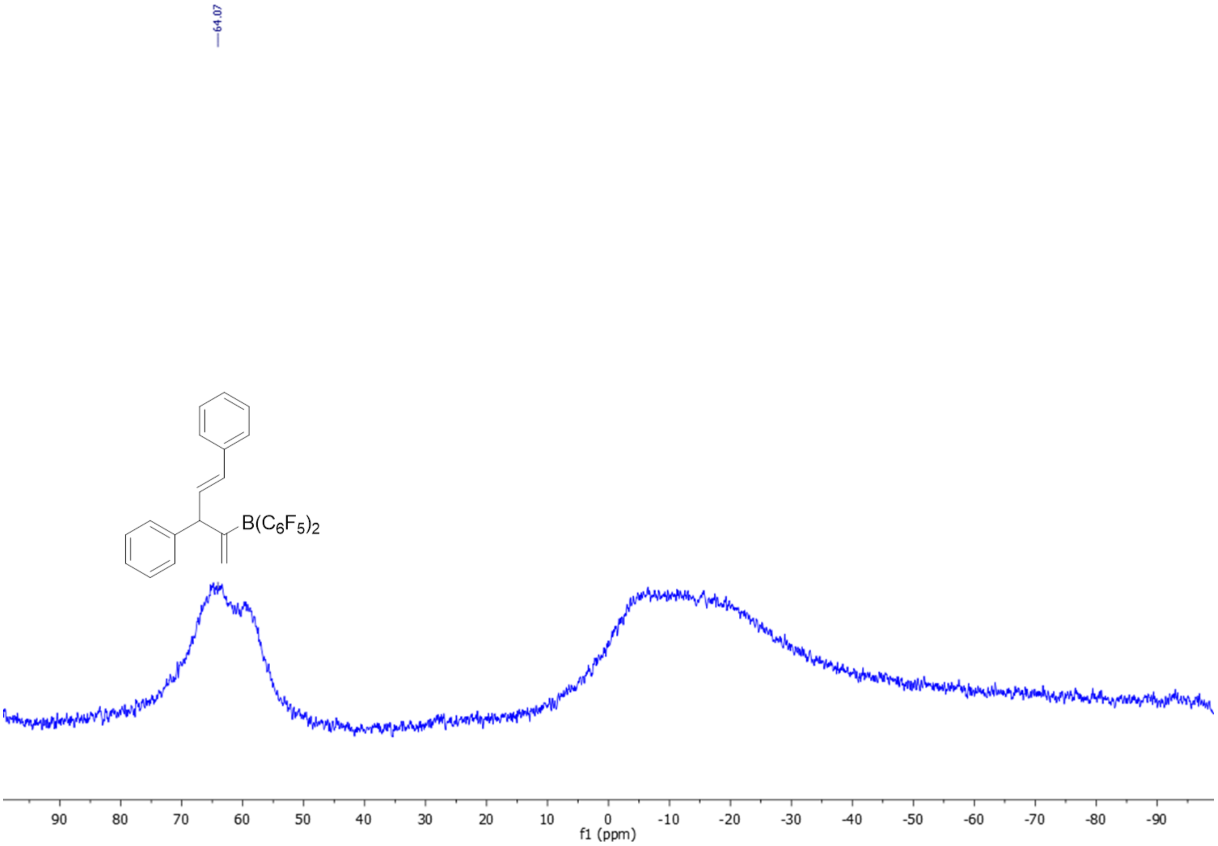

Figure SI 12: ^{11}B NMR spectrum of the 1,2,-carboboration product **3** of bis(pentafluorophenyl)((*E*)-2-phenylethenyl)borane with phenylallene, (*E*)-(3,5-diphenylpenta-1,4-dien-2-yl)borane (128 MHz, DCM-d_2).

For the *in situ* quantification of the diene **3**, 1,3,5-trimethoxybenzene (0.03 mmol, 50 µL of a 0.6 M stock solution in benzene-d_6) was added to the reaction mixture inside the glovebox. The ^{1}H NMR spectrum showed that the *in situ* yield of the new diene is 69 % (Figure SI 13). The experiment was reproduced under identical reaction conditions. The yield of diene **3** of the second run is 63 % (Figure SI 14). The average yield of **3** of both experiments is 66 %.

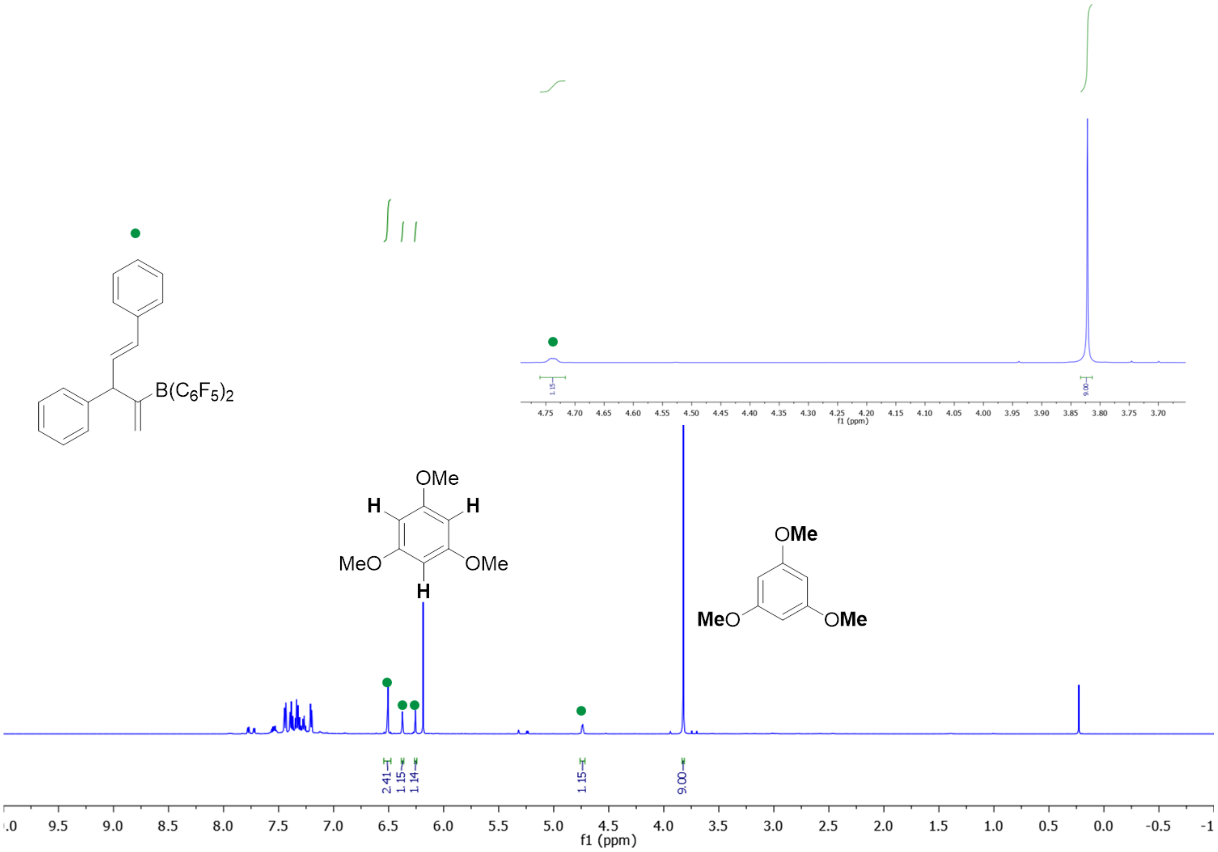

Figure SI 13: ^{1}H NMR spectrum of the 1,2-carboboration product of bis(pentafluorophenyl)((*E*)-2-phenylethenyl)borane **2** with phenylallene, (*E*)-(3,5-diphenylpenta-1,4-dien-2-yl)borane **3**, with 1,3,5-trimethoxybenzene (0.03 mmol, 50 µL of a 0.6 M stock solution in benzene-d_6) for *in situ* quantification (600 MHz, DCM-d_2).

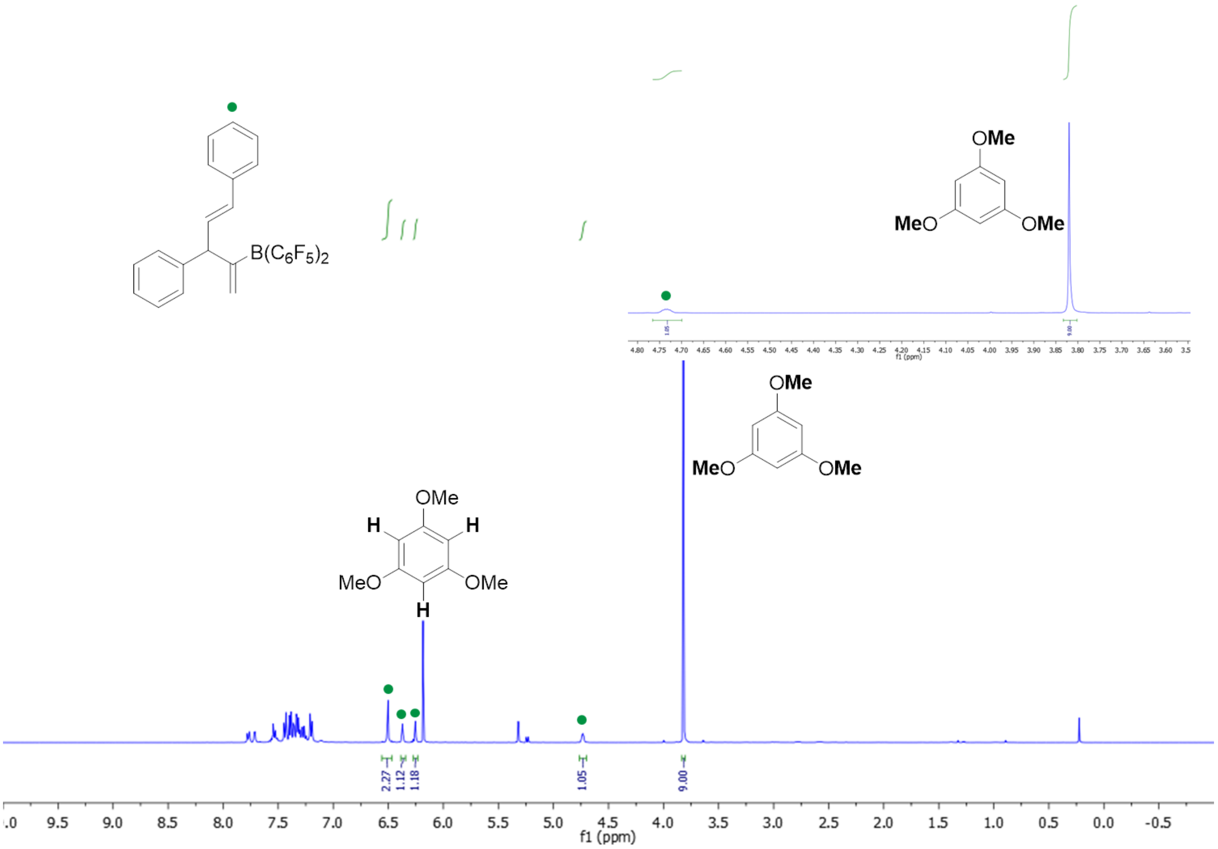

Figure SI 14: ^{1}H NMR spectrum of the reproduction experiment of the 1,2-carboboration product of bis(pentafluorophenyl)((*E*)-2-phenylethenyl)borane **2** with phenylallene, (*E*)-(3,5-diphenylpenta-1,4-dien-2-yl)borane **3**, with 1,3,5-trimethoxybenzene (0.03 mmol, 50 µL of a 0.6 M stock solution in benzene-d_6) for *in situ* quantification (400 MHz, DCM-d_2).

7 Synthesis and characterization of (*E*)-(3,5-diphenylpenta-1,4-dien-2-yl)borane 3

Inside the glovebox, phenylacetylene (47.7 µL, 0.434 mmol, 1.0 equiv.) and Piers' borane (150 mg, 0.434 mmol, 1.0 equiv.) were suspended in 1,2-dichloroethane (3 mL) in a 50 mL Schlenk tube with J Young valve. After 10 min at room temperature phenylallene (55.4 µL, 0.434 mmol, 1.0 equiv.) was added. The tube was closed, taken out of the glovebox, and placed into a, oil bath at 70 °C overnight. The solvent was evaporated at room temperature under oil pump vacuum under inert conditions. The tube was taken into the glovebox, extracted three times with *n*-pentane (2 mL) into a 10 mL glass vial. After the solvent was evaporated at room temperature, a part of the sample (about 20-30 mg) was dissolved in toluene-d_8 and transferred into an NMR tube with J Young valve. The reaction mixture with (*E*)-(3,5-diphenylpenta-1,4-dien-2-yl)borane **3** as main product was fully characterized by NMR spectroscopy.

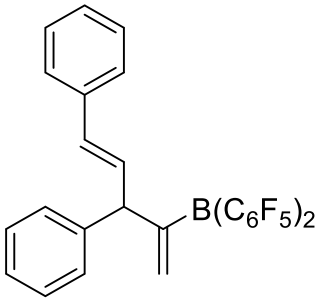

^{1}H NMR (600 MHz, toluene-d_8) δ 7.15 – 7.12 (m, 2H, Ar-*H*), 7.06 (dd, *J* = 8.5, 6.7 Hz, 2H, Ar-*H*), 7.03 – 6.99 (m, 2H, Ar-*H*), 6.98 – 6.96 (m, 3H, Ar-*H*), 6.95 – 6.92 (m, 1H, Ar-*H*), 6.27 (d, *J* = 5.8 Hz, 2H, *H*C=C*H*), 6.08 (t, *J* = 2.0 Hz, 1H, C_{sp2}-H_2), 5.87 (t, *J* = 1.9 Hz, 1H, C_{sp2}-H_2).

Remark: Both olefinic hydrogens at the *E*-configurated double bond show the same chemical shift in toluene-d_8 and DCM-d_2 (see chapter 6) in the ^{1}H NMR spectra leading to a higher order spectrum. The NMR shifts were also investigated computationally (see chapter 11).

^{13}C NMR (151 MHz, toluene-d_8) δ 159.29 (br, C_{sp2}-B), 142.87 (Ar-C_q), 141.23 ($C_{sp2}H_2$), 137.16 (Ar-C_q), 132.85 (H*C*=CH-C_{bn}H), 131.31 (HC=*C*H-C_{bn}H), 129.17 (Ar-*C*), 128.85 (Ar-*C*), 128.55 (Ar-*C*), 127.94 (Ar-*C*), 127.19 (Ar-*C*), 126.68 (Ar-*C*), 54.48 (C_{bn}-H).

Remark: In ^{13}C{^{1}H} and ^{13}C APT edited NMR spectrum the carbons bound to fluorine are broad and barely visible and could therefore not be assigned.

^{19}F NMR (377 MHz, toluene-d_8) δ -129.98 – -130.15 (m, 4F, o-*F*), -148.20 – -148.42 (m, 2F. p-*F*), -160.95 – -161.18 (m, 4F, m-*F*).

^{11}B NMR (128 MHz, toluene-d_8) δ 65.21.

7.1 Additional NMR spectra of (*E*)-(3,5-diphenylpenta-1,4-dien-2-yl)borane

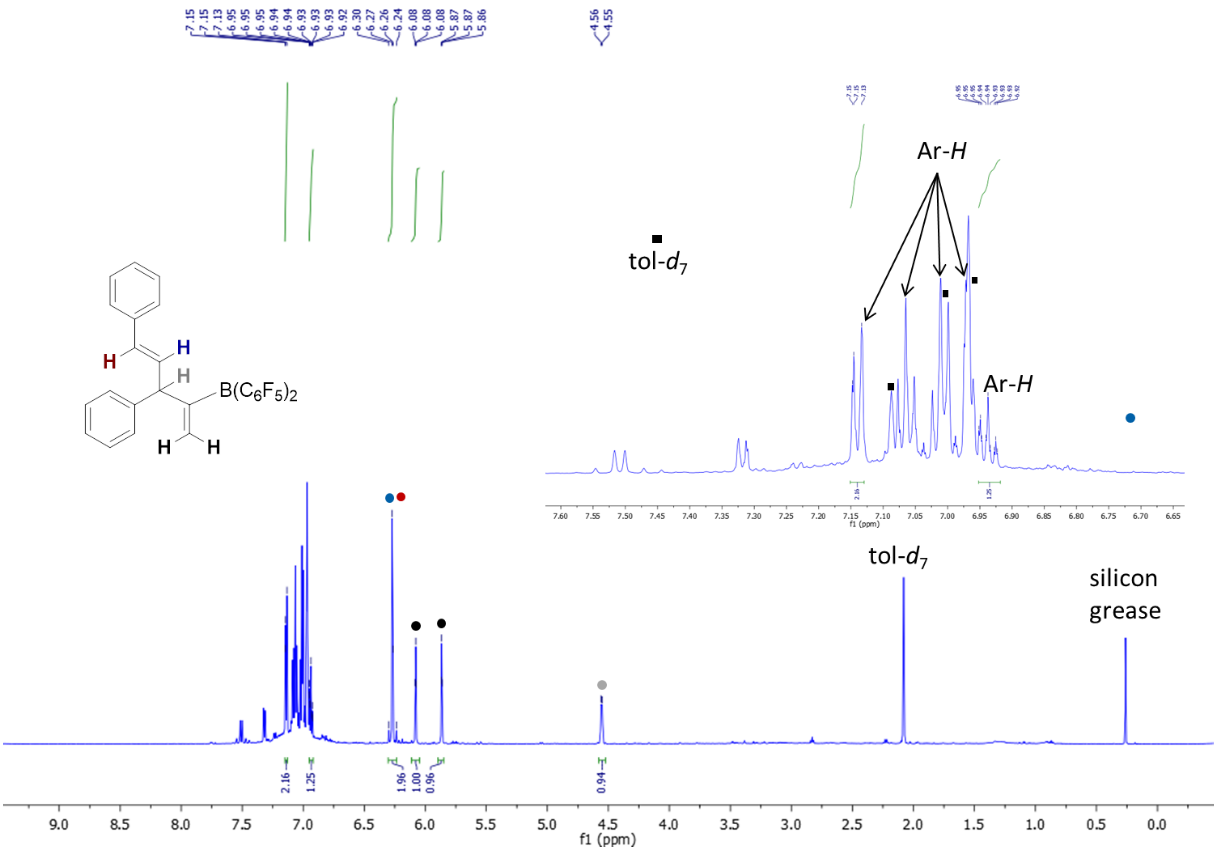

Figure SI 15: ^{1}H NMR spectrum of the reaction mixture of vinylborane **2** and phenylallene with the addition product **3** as main product (400 MHz, toluene-d_8).

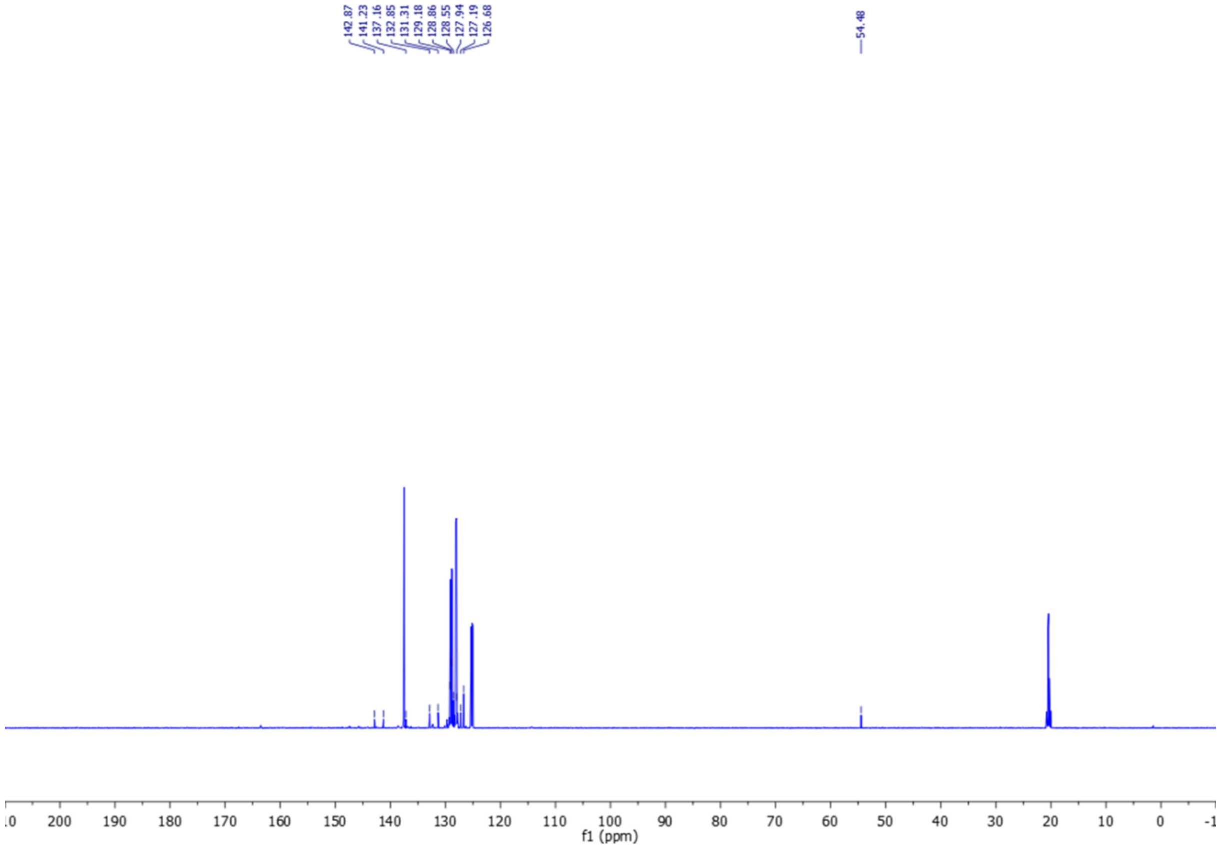

Figure SI 16: ^{13}C NMR spectrum of the reaction mixture of vinylborane **2** and phenylallene with the addition product **3** as main product (101 MHz, toluene-d_8).

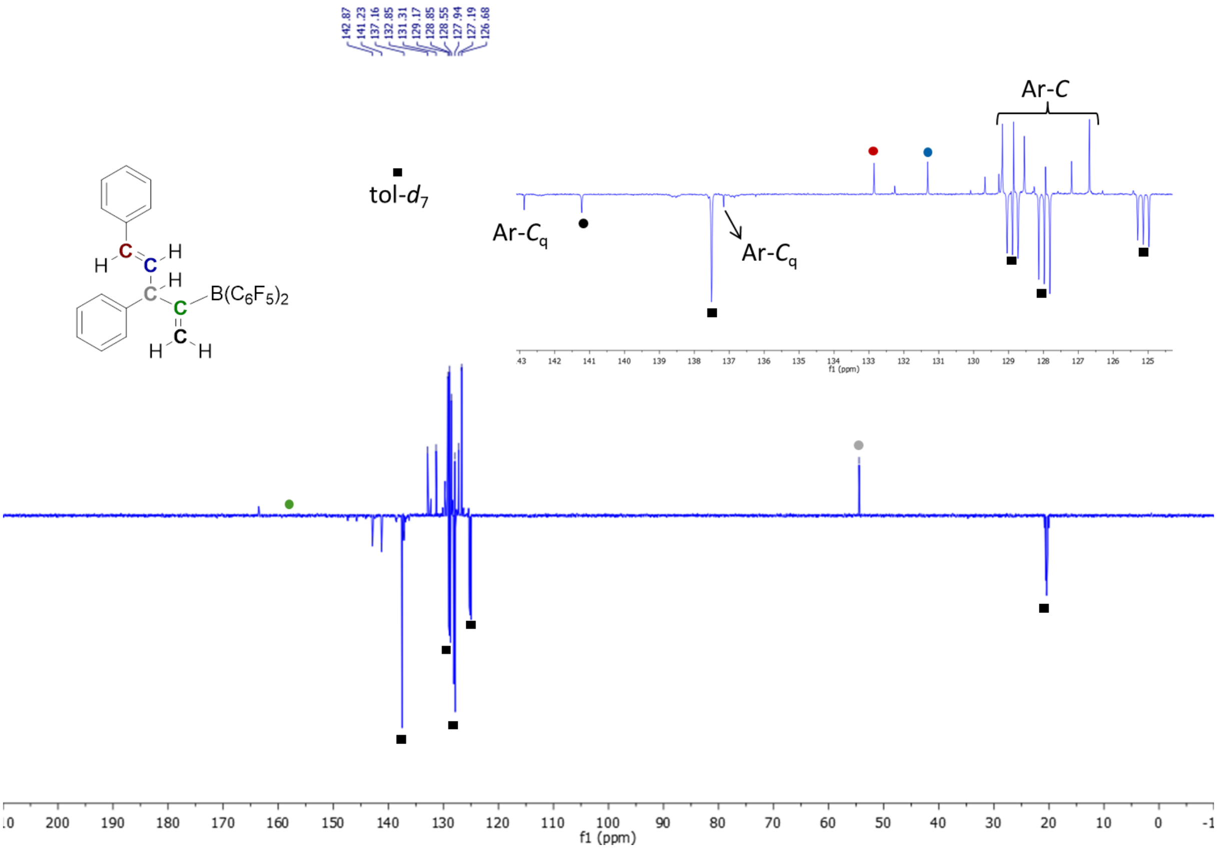

Figure SI 17: APT edited ^{13}C NMR spectrum of the reaction mixture of vinylborane **2** and phenylallene with the addition product **3** as main product (101 MHz, toluene-d_8).

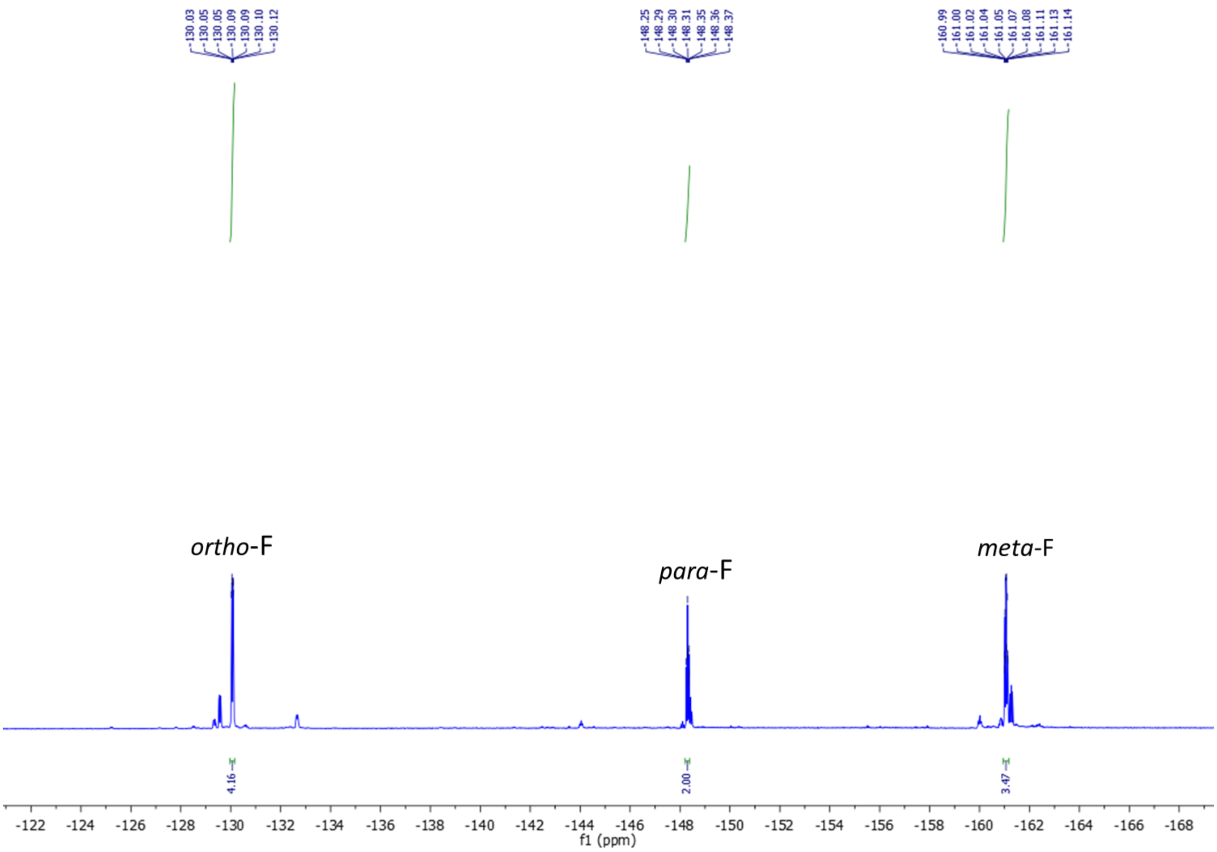

Figure SI 18: ^{19}F NMR spectrum of the reaction mixture of vinylborane **2** and phenylallene with the addition product **3** as main product (377 MHz, toluene-d_8).

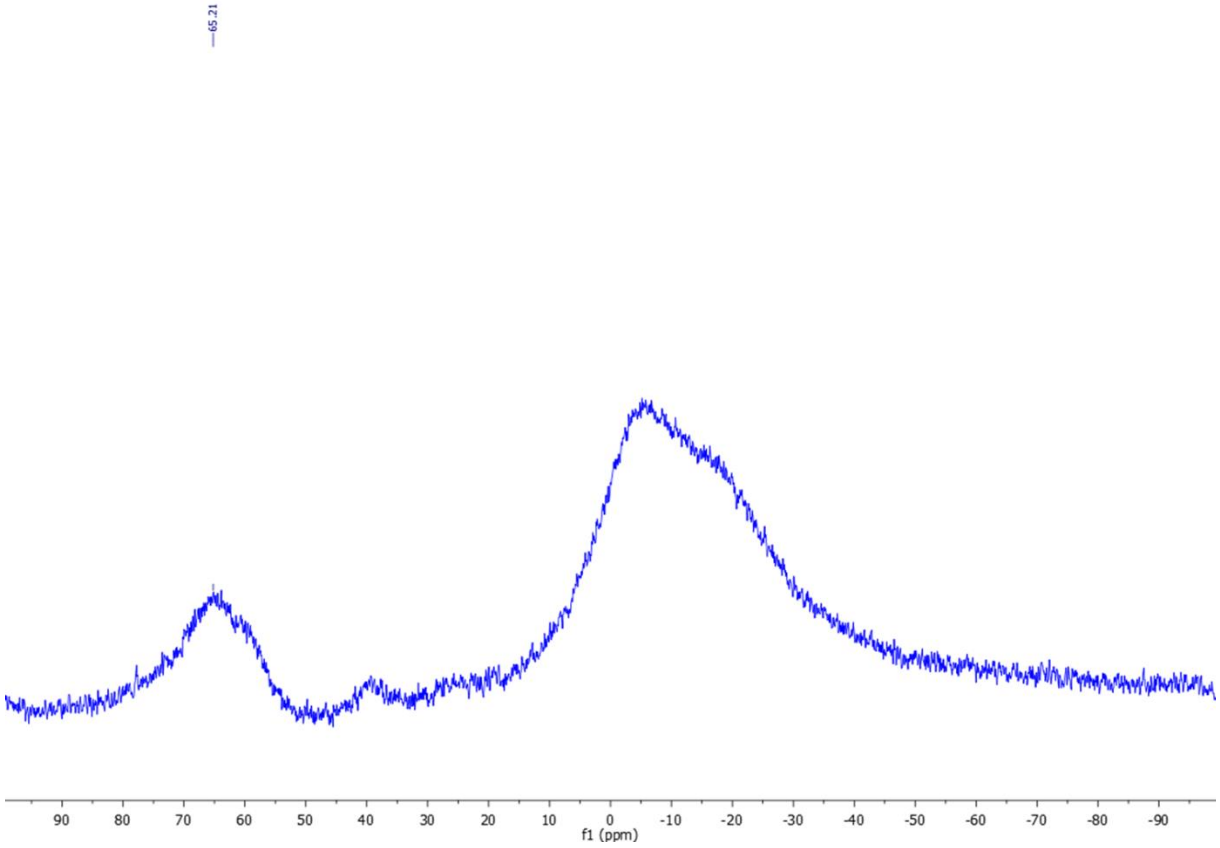

Figure SI 19: ^{11}B NMR spectrum of the reaction mixture of vinylborane **2** and phenylallene with the addition product **3** as main product (128 MHz, toluene-d_8).

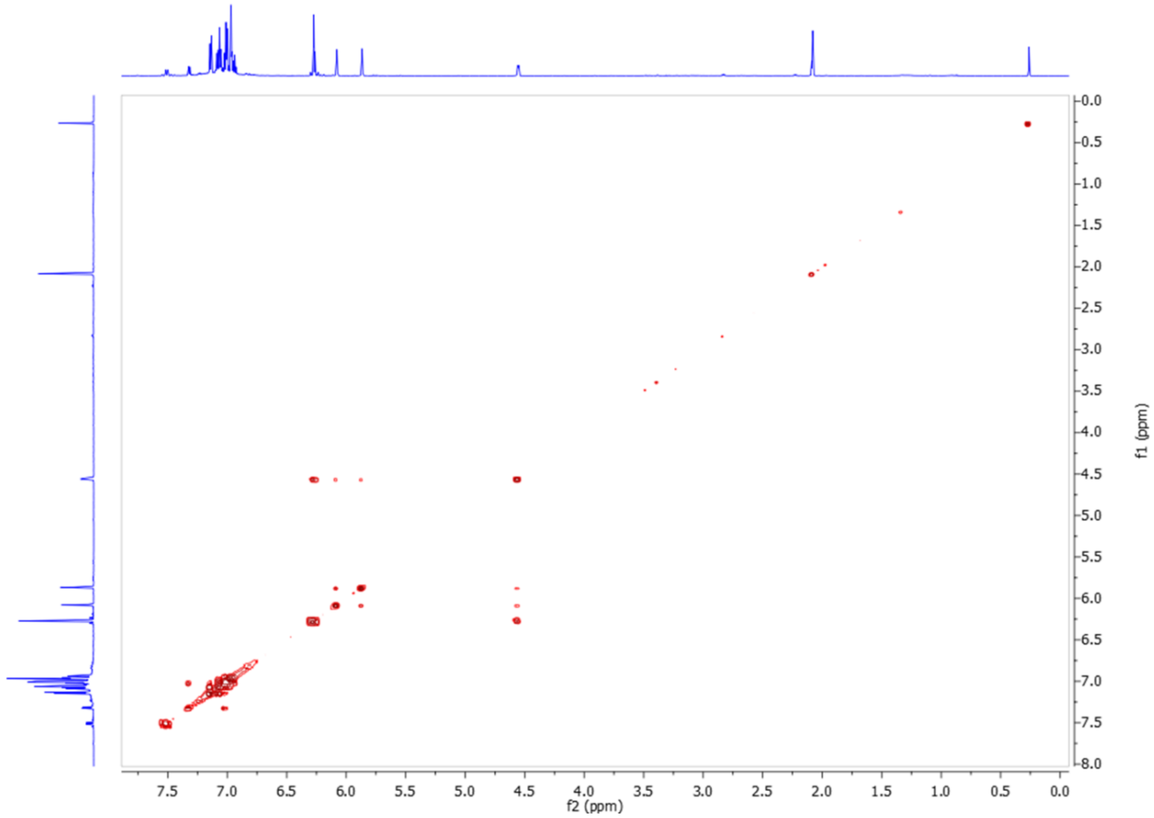

Figure SI 20: HH COSY NMR spectrum of the reaction mixture of vinylborane **2** and phenylallene with the addition product **3** as main product (101 MHz, toluene-d_8).

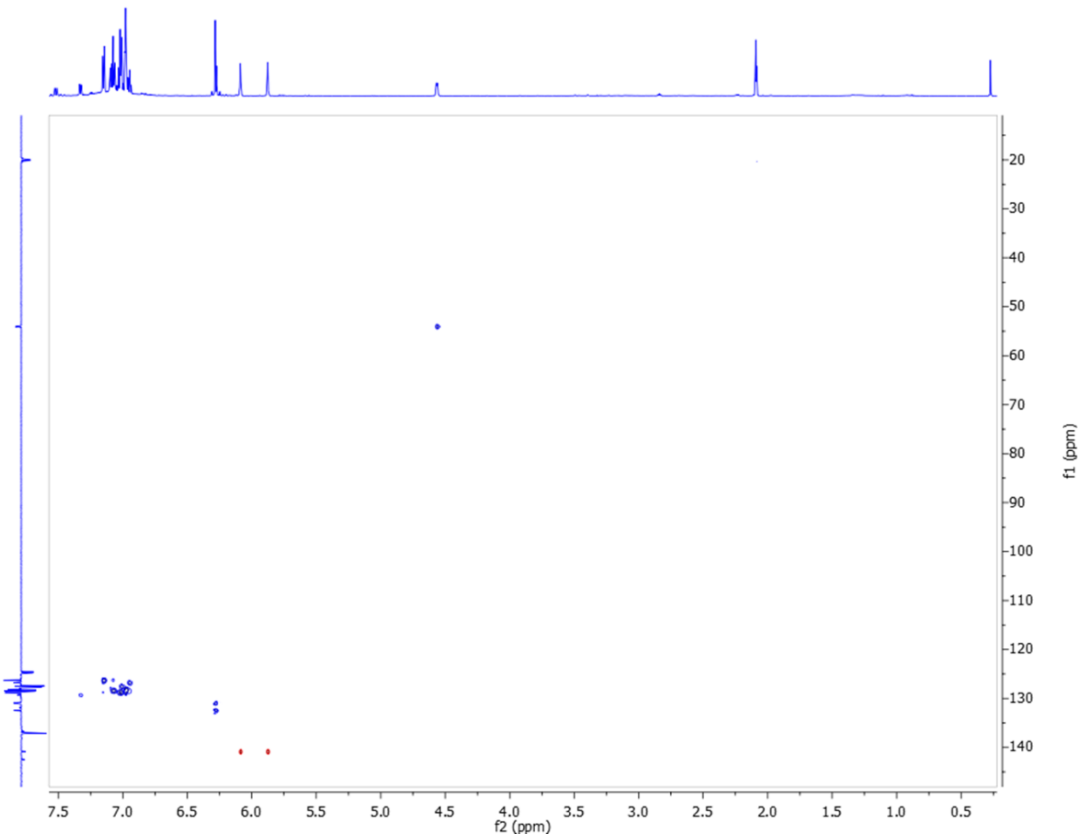

Figure SI 21: $^{1}H^{13}C$ HSQC NMR spectrum of the reaction mixture of vinylborane **2** and phenylallene with the addition product **3** as main product (101 MHz, toluene-d_8).

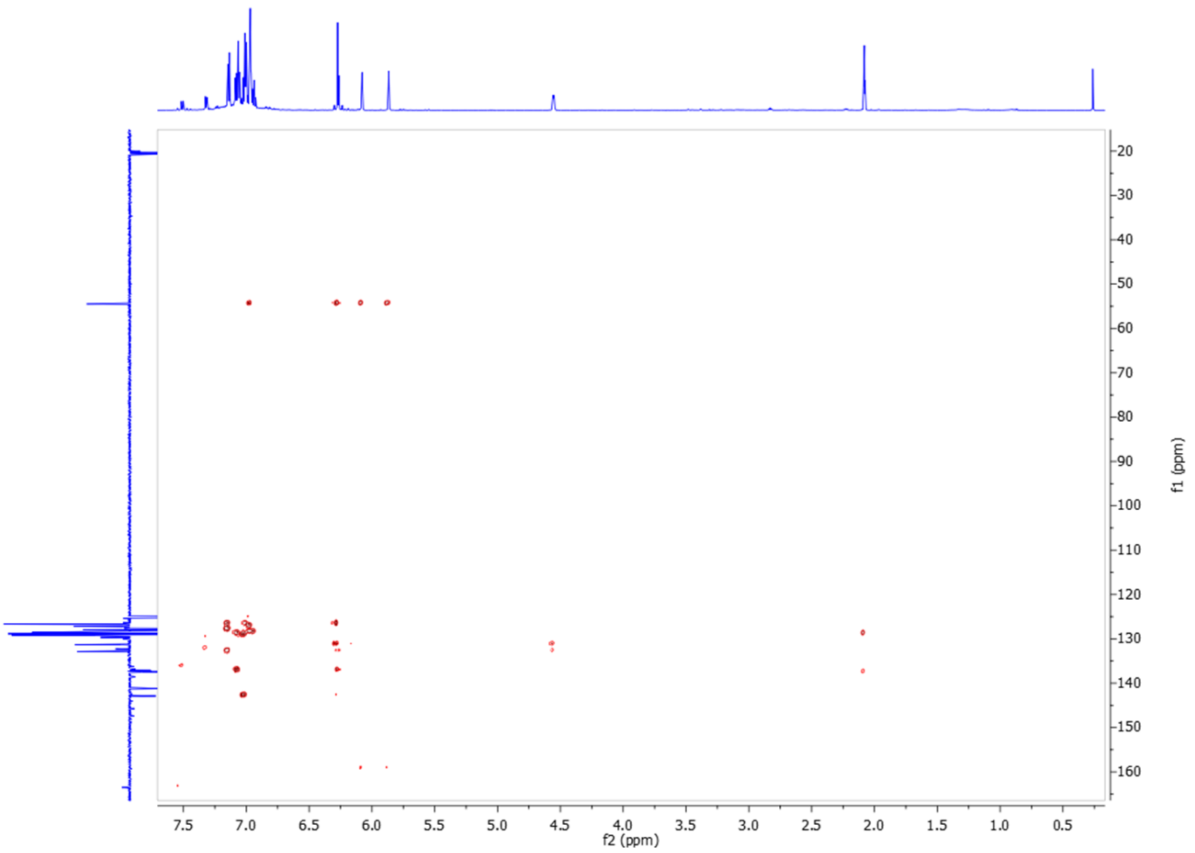

Figure SI 22: $^{1}H^{13}C$ HMBC NMR spectrum of the reaction mixture of vinylborane **2** and phenylallene with the addition product **3** as main product (101 MHz, toluene-d_8).

8 Synthesis and characterization of (*E*)-(3,5-diphenylpenta-1,4-dien-2-yl)borane pyridine adduct 4

Inside the glovebox, phenylacetylene (47.7 µL, 0.434 mmol, 1.0 equiv.) and Piers' borane (150.0 mg, 0.434 mmol, 1.0 equiv.) were suspended in dry 1,2-dichloroethane (5 mL) in a Schlenk tube with J Young valve. After 10 min at room temperature phenylallene (55.4 µL, 0.434 mmol, 1.0 equiv.) was added. The tube was closed, taken out of the glovebox, and heated to 80 °C for 17 h in an oil bath. The tube was cooled to room temperature, taken into the glovebox, and pyridine (35.0 µL, 0.434 mmol, 1.0 equiv.) was added. The tube was taken out of the glovebox and the solvent was evaporated under high vacuum at room temperature. The residue was dissolved in DCM (5 mL), filtered through a Whatman filter, layered with *n*-hexane (45 mL), and stored for about 40 h at -26 °C. The product **4** was obtained as colourless crystals (133.7 mg, 0.208 mmol, 48 %).

Crystals obtained this way were suitable for SCXRD analysis (see chapter 10.1).

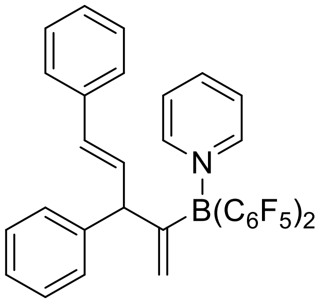

^{1}H NMR (400 MHz, chloroform-*d*) δ 8.68 (d, *J* = 5.9 Hz, 2H, *ortho*-Py), 8.03 (tt, *J* = 7.6, 1.5 Hz, 1H, *para*-Py), 7.57 (dd, *J* = 7.7, 6.8 Hz, 2H, *meta*-Py), 7.30 – 7.22 (m, 4H, Ar-*H*), 7.15 (ddt, *J* = 8.5, 5.9, 2.0 Hz, 1H, Ar-*H*), 7.10 – 7.03 (m, 3H, Ar-*H*), 6.99 (dd, *J* = 7.6, 1.9 Hz, 2H, Ar-*H*), 6.65 (dd, *J* = 16.0, 5.7 Hz, 1H, HC=C*H*-C_{bn}H), 5.75-5.66 (m, 2H, *H*C=CH, $C_{sp2}H_2$), 5.24 (s, 1H, $C_{sp2}H_2$), 4.30 (d, *J* = 5.7 Hz, 1H, $C_{bn}H$).

^{13}C NMR (101 MHz, chloroform-*d*) δ 155.69 (C_q-B) 147.18 (*ortho*-Py), 142.23 (Ar-C_q), 141.76 (*para*-Py), 137.95 (Ar-C_q), 136.72 (HC=*C*H-C_{bn}H), 129.72 (H*C*=CH-C_{bn}H), 128.50 (Ar-*C*), 128.13 (Ar-*C*), 127.60 (Ar-*C*), 126.91 (Ar-*C*), 126.26 (Ar-*C*), 125.84 (Ar-*C*), 125.52 (*meta*-Py), 120.37 ($C_{sp2}H_2$), 53.83 (C_{bn}).

Remark: In ^{13}C{^{1}H} NMR spectrum the carbons bound to fluorine are not visible. Additionally, the quaternary carbon bound directly to the boron is also not visible because of the signal broadening but the chemical shift can be identified using the ^{1}H^{13}C HMBC spectrum.

^{19}F NMR (377 MHz, chloroform-*d*) δ -128.82 (d, *J* = 23.6 Hz, 2F, *ortho*-F), -131.53 (d, *J* = 23.7 Hz, 2F, *ortho*-F), -157.35 (t, *J* = 20.4 Hz, 1F, *para*-F), -157.80 (t, *J* = 20.4 Hz, 1F, *para*-F), -163.25 (dddd, *J* = 34.5, 23.7, 19.9, 8.7 Hz, 4F, *meta*-F).

^{11}B NMR (128 MHz, chloroform-*d*) δ -0.63.

HRMS (APCI) m/z [M+H$^+$] calc. for $C_{34}H_{20}BF_{10}NNa^+$: 666.1421; found [M+Na$^+$]: 666.1422

8.1 Additional NMR spectra of (*E*)-(3,5-diphenylpenta-1,4-dien-2-yl)borane pyridine adduct 4

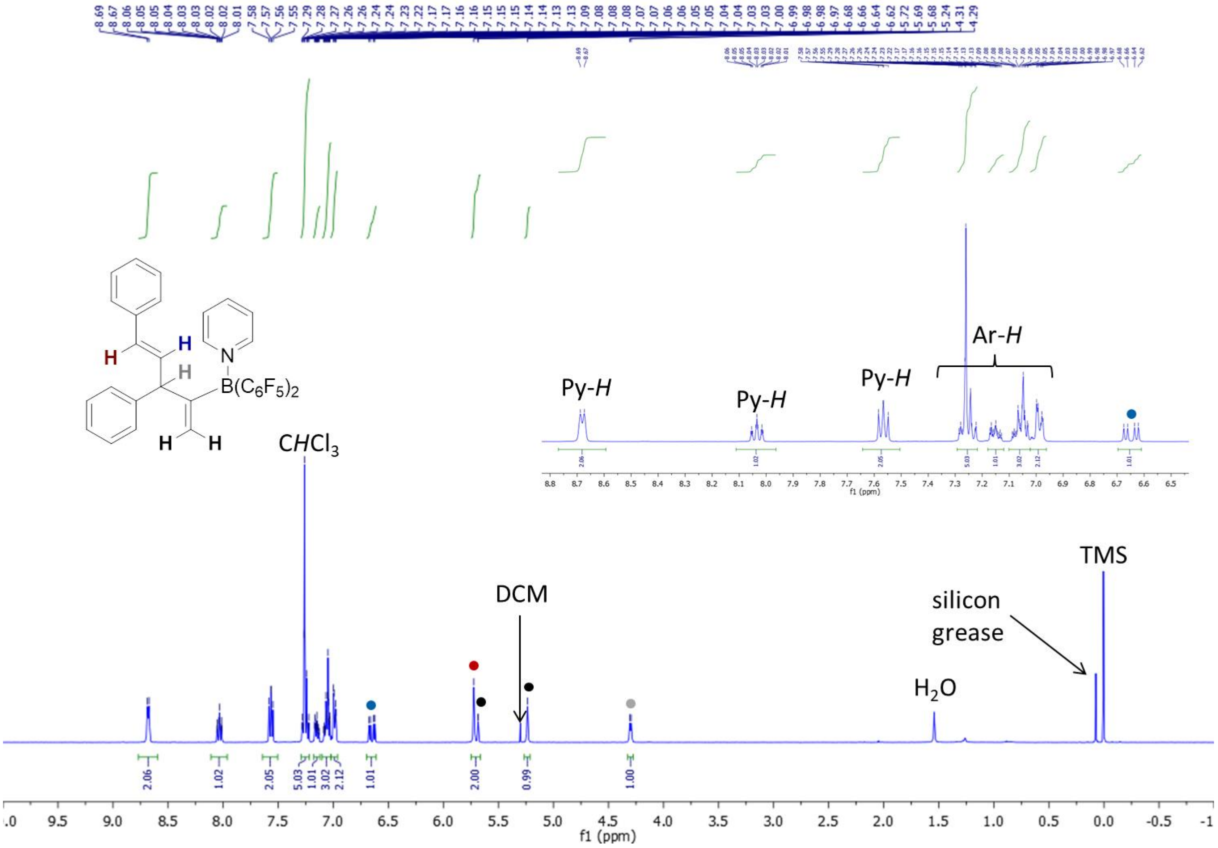

Figure SI 23: ^{1}H NMR spectrum of the addition product of bis(pentafluorophenyl)((*E*)-2-phenylethenyl)borane **2** and phenylallene as pyridine adduct **4** with low field excerpt (400 MHz, chloroform-*d*).

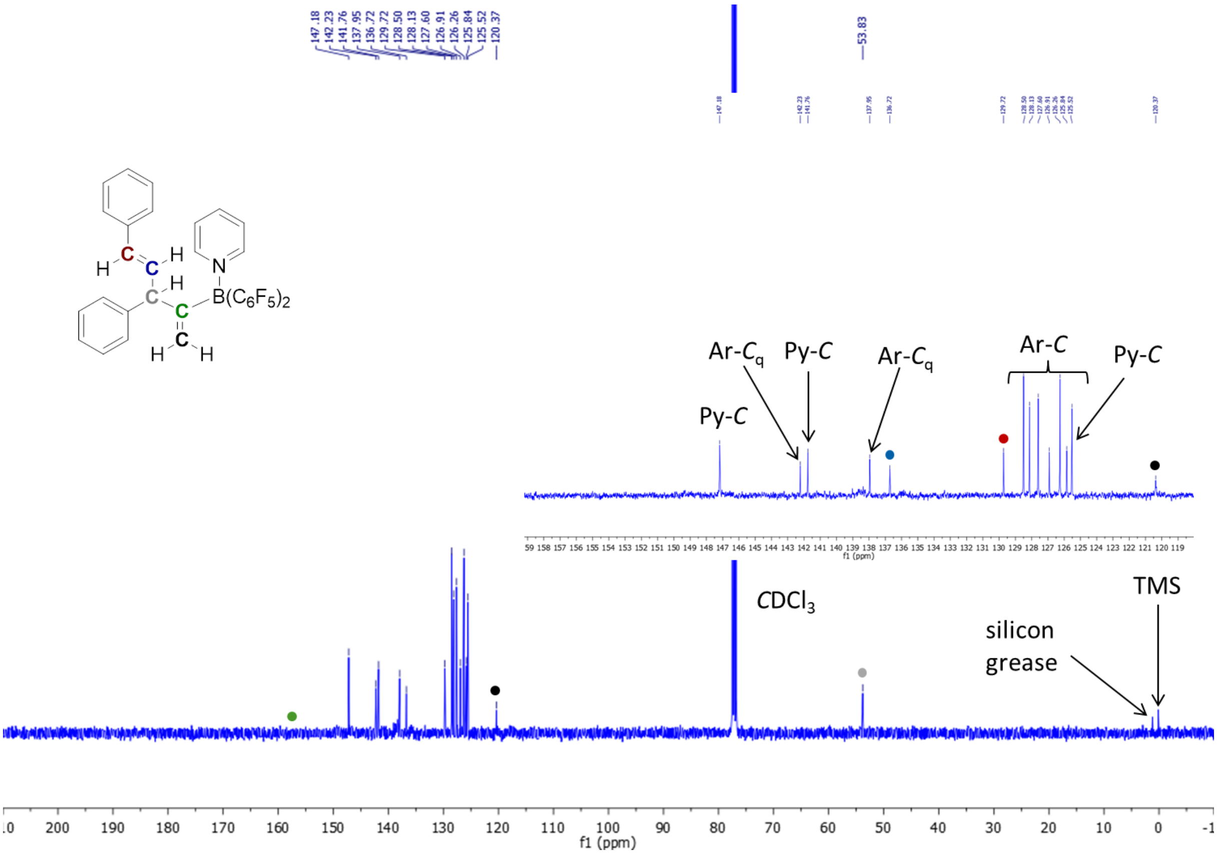

Figure SI 24: $^{13}C\{^{1}H\}$ NMR spectrum of the addition product of bis(pentafluorophenyl)((*E*)-2-phenylethenyl)borane **2** and phenylallene as pyridine adduct **4** with low field excerpt (151 MHz, chloroform-*d*).

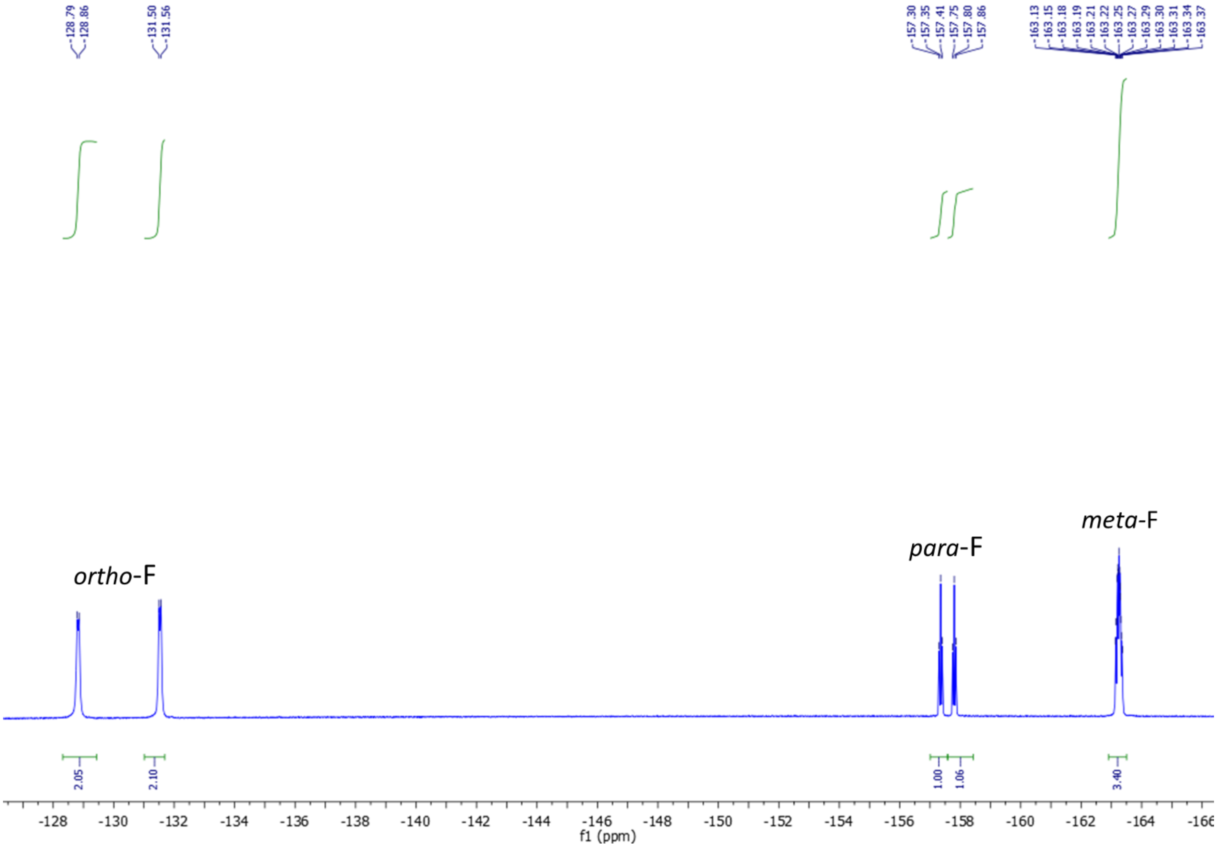

Figure SI 25: ^{19}F NMR spectrum of the addition product of bis(pentafluorophenyl)((*E*)-2-phenylethenyl)borane **2** and phenylallene as pyridine adduct **4** (377 MHz, chloroform-*d*).

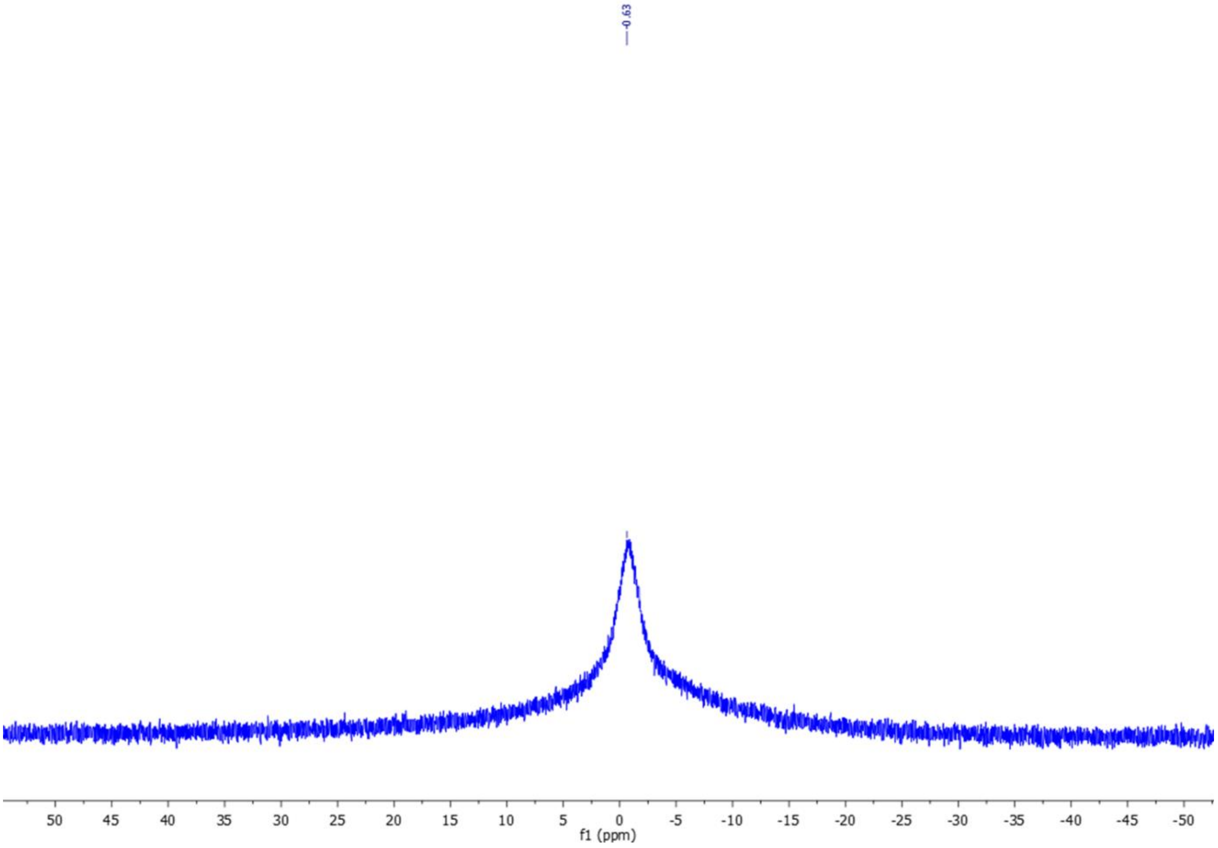

Figure SI 26: ^{11}B NMR spectrum of the addition product of bis(pentafluorophenyl)((*E*)-2-phenylethenyl)borane **2** and phenylallene as pyridine adduct **4** (128 MHz, chloroform-*d*).

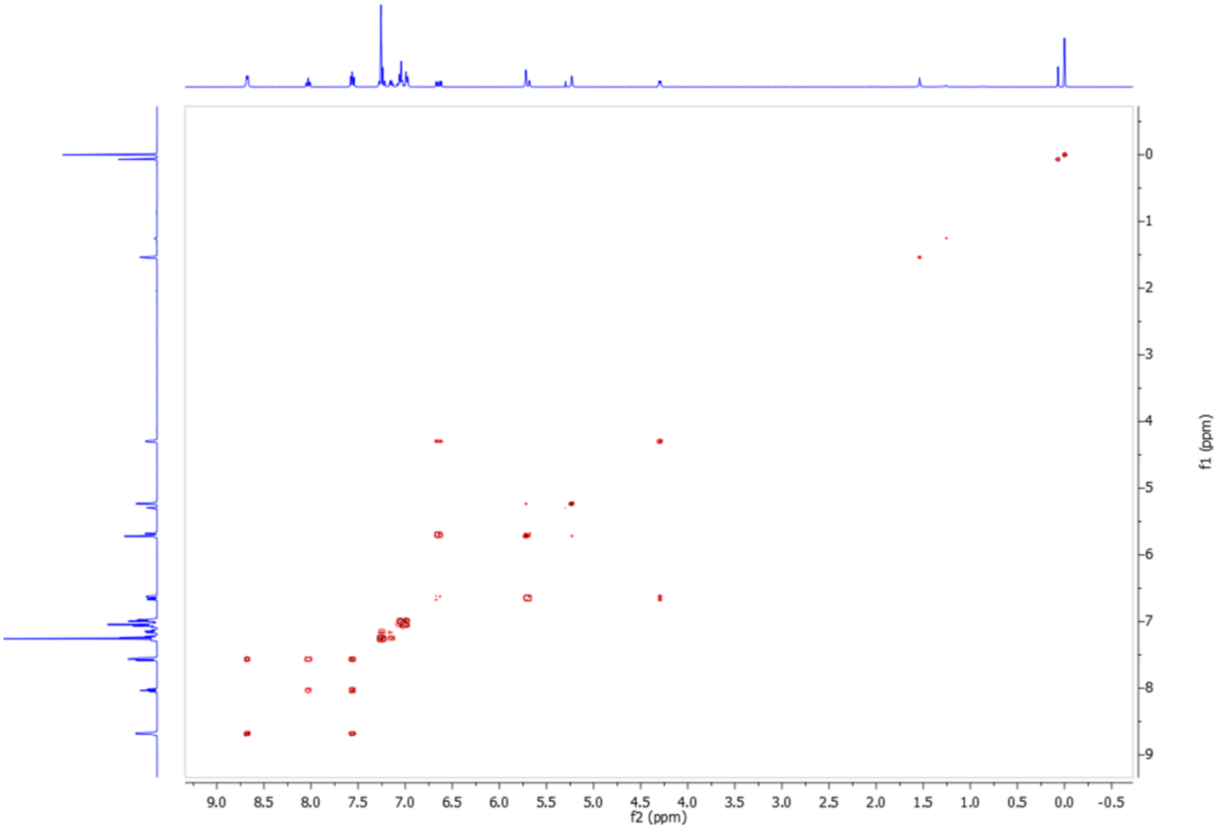

Figure SI 27: HH COSY NMR spectrum of the addition product of bis(pentafluorophenyl)((*E*)-2-phenylethenyl)borane **2** and phenylallene as pyridine adduct **4** (128 MHz, chloroform-*d*).

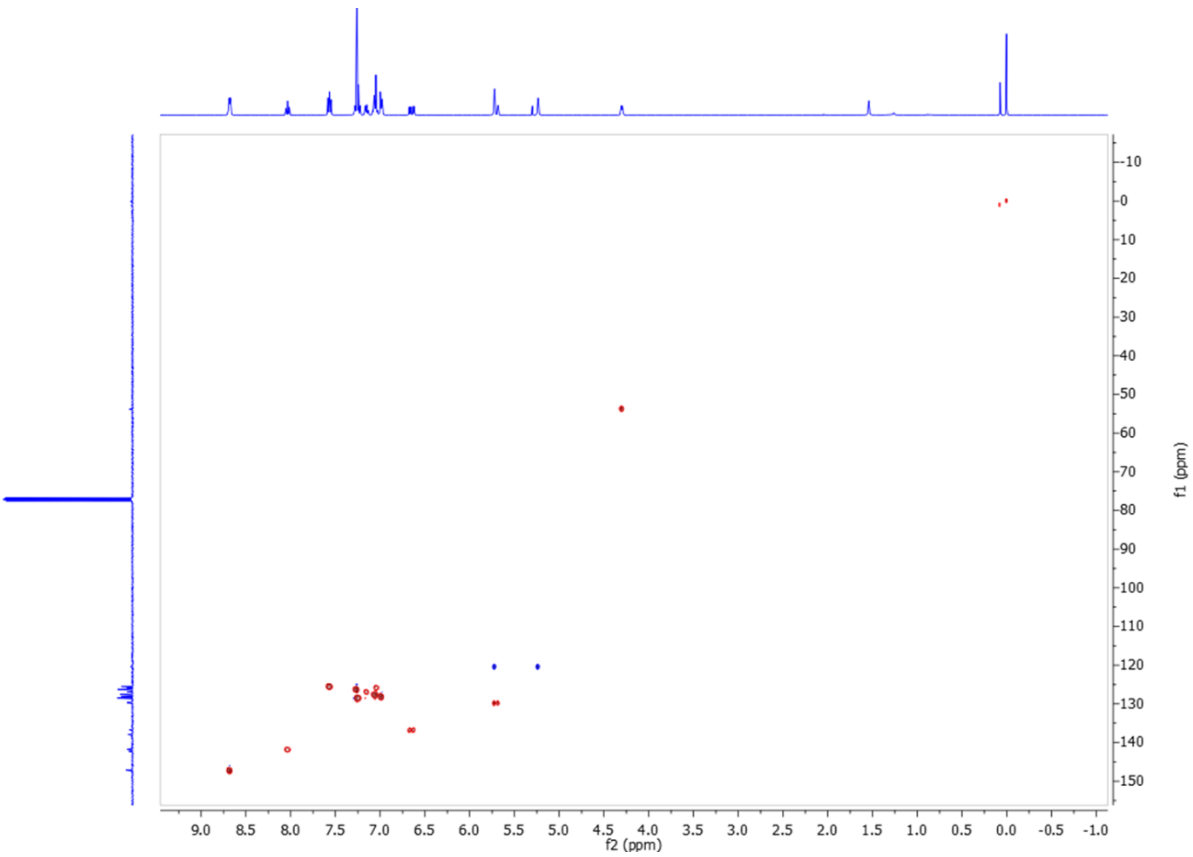

Figure SI 28: $^{1}H^{13}C$ HSQC NMR spectrum of the addition product of bis(pentafluorophenyl)((*E*)-2-phenylethenyl)borane **2** and phenylallene as pyridine adduct **4** (101 MHz, chloroform-*d*).

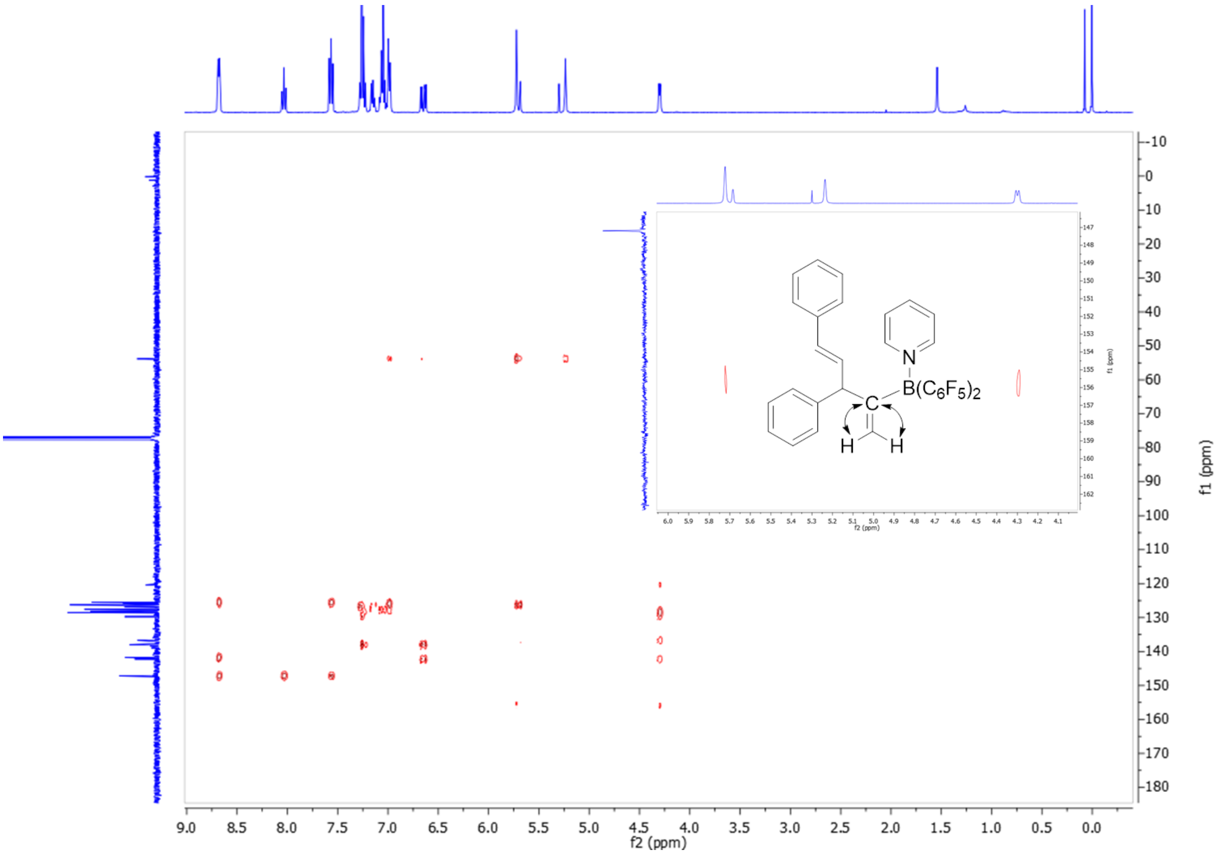

Figure SI 29: $^{1}H^{13}C$ HMBC NMR spectrum of the addition product of bis(pentafluorophenyl)((*E*)-2-phenylethenyl)borane **2** and phenylallene as pyridine adduct **4** with low field excerpt showing the ^{2}J coupling between the terminal olefinic hydrogen and the quaternary carbon bound to the boron (101 MHz, chloroform-*d*).

9 Synthesis and characterization of 1,4-diene products

9.1 General procedure

Inside the glovebox Piers' borane (138.4 mg, 0.40 mmol, 1.0 equiv.) and the terminal alkyne (0.40 mmol, 1.0 equiv) were suspended in dry DCM (3.5 mL) in a Schlenk tube with J Young valve and stirred for 30 min at room temperature. The allene (0.48 mmol, 1.2 equiv.) was added, the tube was closed, and stirred for the specified time at the specified temperature. The solvent was evaporated under inert conditions at oil-pump vacuum. The tube was taken into the glovebox. The residue was dissolved in dry THF (20 mL) and $Pd(PPh_3)_4$ (23.1 mg, 0.02 mmol, 0.05 equiv.) was added. The tube was closed, taken out of the glovebox, degassed aqueous NaOH (3 M, 6 mL), and the specified organoiodide (2.0 mmol, 5.0 equiv.) were added under inert conditions. The tube was closed and heated for 7 h to 70 °C in an oil bath. The reaction mixture was cooled to room temperature and transferred to a separation funnel. Water was added and the aqueous phase was extracted three times with *n*-hexane. The combined organic phases were dried over Na_2SO_4, filtered and the solvent was evaporated under reduced pressure.

9.2 Purification and characterization of 1,4-diene products

(*E*)-1,3,4-triphenyl-penta-1,4-diene **6a** was synthesized according to the general procedure with phenylacetylene (43.9 µL, 0.40 mmol, 1.0 equiv.), phenylallene (61.3 µL, 0.48 mmol, 1.2 equiv.) and iodobenzene (223.8 µL, 2.0 mmol, 5.0 equiv.). The carboboration was performed at 60 °C. The crude product was purified by flash column chromatography (*n*-hexane/EtOAc, 199:1) and subsequent distillation in a Kugelrohr apparatus (140 °C, 0.1-0.3 mbar). The diene **6a** was isolated as colorless oil (61.2 mg, 0.206 mmol, 52 %).

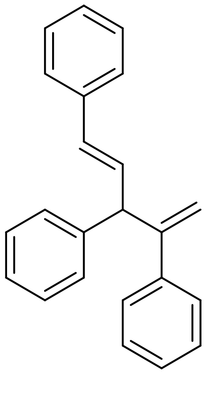

rac

^{1}H NMR (400 MHz, chloroform-*d*) δ 7.42 – 7.15 (m, 15H, Ar-*H*), 6.56 (dd, *J* = 15.9, 7.2 Hz, 1H, HC=C*H*-C_{bn}), 6.31 (d, *J* = 16.0 Hz, 1H, *H*C=CH-CH_{bn}), 5.60 (s, 1H, $C_{gem,sp2}H$), 5.20 (s, 1H, $C_{gem,sp2}H$), 4.79 (d, *J* = 7.2 Hz, 1H, H_{bn}).

^{13}C NMR (101 MHz, chloroform-*d*) δ 150.25 ($C_{q,sp2}$), 141.97 (Ar-C_q), 141.72 (Ar-C_q), 137.50 (Ar-C_q), 132.36 (H*C*=CH-C_{bn}), 131.40 (HC=*C*H-C_{bn}), 128.86 (Ar-*C*), 128.63 (Ar-*C*), 128.61 (Ar-*C*), 128.32 (Ar-*C*), 127.49 (Ar-C_{para}), 127.40 (Ar-C_{para}), 126.74 (Ar-*C*), 126.66 (Ar-C_{para}), 126.44 (Ar-*C*), 115.80 ($C_{sp2}H_2$), 53.49 (C_{bn})

HRMS (APCI) m/z [M+H$^+$] calc. $C_{23}H_{20}$+H$^+$: 297.1638; found: 297.1640.

(*E*)-1,4-diphenyl-3-(*para*-methylphenyl)-penta-1,4-diene **6b** was synthesized according to the general procedure with phenylacetylene (43.9 µL, 0.40 mmol, 1.0 equiv.), *para*-methyl-phenylallene (62.5 mg, 0.48 mmol, 1.2 equiv.) and iodobenzene (223.8 µL, 2.0 mmol, 5.0 equiv.). The carboboration was performed at 60 °C. The crude product was purified by flash column chromatography (*n*-hexane/EtOAc, 199:1) and subsequent distillation in in a Kugelrohr apparatus (140 - 150°C, 0.1-0.2 mbar). The diene **6b** was isolated as colorless oil (78.9 mg, 0.254 mmol, 64 %).

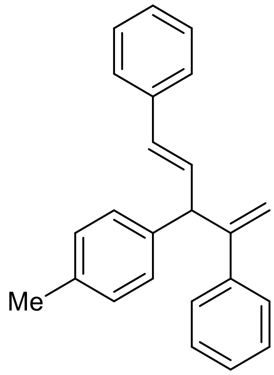

rac

^{1}H NMR (400 MHz, chloroform-*d*) δ 7.40 – 7.37 (m, 2H, Ar-*H*), 7.36 – 7.32 (m, 2H, Ar-*H*), 7.31 – 7.17 (m, 8H, Ar-*H*), 7.10 (d, *J* = 7.9 Hz, 1H, Ar-*H*) 6.55 (dd, *J* = 15.9, 7.2 Hz, 1H, HC=C*H*-C_{bn}), 6.31 (d, *J* = 15.9 Hz, 1H, *H*C=CH-CH_{bn}), 5.59 (s, 1H, $C_{gem,sp2}$*H*), 5.19 (s, 1H, $C_{gem,sp2}$*H*), 4.75 (d, *J* = 7.1 Hz, 1H, H_{bn}), 2.31 (s, 3H, C*H*$_3$).

^{13}C NMR (101 MHz, chloroform-*d*) δ 150.38 ($C_{q,sp2}$), 141.82 (Ar-C_q), 138.91 (Ar-C_q), 137.58 (Ar-C_q), 136.16 (Ar-C_q), 132.60 (H*C*=CH-C_{bn}), 131.18 (HC=*C*H-C_{bn}), 129.33 (Ar-*C*), 128.72 (Ar-*C*), 128.61 (Ar-*C*), 128.31 (Ar-*C*), 127.45 (Ar-*C*), 127.33 (Ar-*C*), 126.74 (Ar-*C*), 126.44 (Ar-*C*), 115.63 ($C_{sp2}H_2$), 53.49 (C_{bn}), 21.19 (*C*H_3).

HRMS (APCI) m/z [M+H$^+$] calc. $C_{24}H_{22}$+H$^+$: 311.1795; found: 311.1793.

(*E*)-1,4-diphenyl-3-(*para*-*iso*-propylphenyl)-penta-1,4-diene **6c** was synthesized according to the general procedure with phenylacetylene (43.9 µL, 0.40 mmol, 1.0 equiv.), *para*-isopropylphenylallene (76.0 mg, 0.48 mmol, 1.2 equiv.) and iodobenzene (223.8 µL, 2.0 mmol, 5.0 equiv.). The carboboration was performed at 60 °C. The crude product was purified by flash column chromatography (*n*-hexane/EtOAc, 200:1) and subsequently distillation in a Kugelrohr apparatus (140 - 160°C, 0.4 mbar). The diene **6c** was isolated as colorless oil (97.6 mg, 0.289 mmol, 72 %).

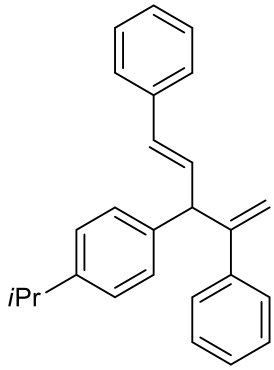

rac

^{1}H NMR (400 MHz, chloroform-*d*) δ 7.42 – 7.38 (m, 2H, Ar-*H*), 7.35 – 7.31 (m, 2H, Ar-*H*), 7.28 – 7.12 (m, 10H, Ar-*H*), 6.54 (dd, *J* = 15.9, 7.3 Hz, 1H, HC=C*H*-C_{bn}), 6.33 (d, *J* = 15.9 Hz, 1H, *H*C=CH-CH_{bn}), 5.58 (s, 1H, $C_{gem,sp2}H$), 5.17 (s, 1H, $C_{gem,sp2}H$), 4.75 (d, *J* = 7.3 Hz, 1H, H_{bn}), 2.86 (hept, *J* = 6.9 Hz, 1H, C*H*(CH_3)$_2$), 1.22 (d, *J* = 6.9 Hz, 6H, CH(CH_3)$_2$).

^{13}C NMR (101 MHz, chloroform-*d*) δ 150.39 ($C_{q,sp2}$), 147.10 (Ar-C_q), 141.86 (Ar-C_q), 139.31 (Ar-C_q), 137.58 (Ar-C_q), 132.58 (H*C*=CH-C_{bn}), 131.14 (HC=*C*H-C_{bn}), 128.67 (Ar-*C*), 128.60 (Ar-*C*), 128.31 (Ar-*C*), 127.44 (Ar-*C*), 127.31 (Ar-*C*), 126.73 (Ar-*C*), 126.64 (Ar-*C*), 126.43 (Ar-*C*), 115.69 ($C_{sp2}H_2$), 53.11 (C_{bn}), 33.79 (*C*H(CH_3)$_2$), 21.19 (CH(*C*H_3)$_2$).

HRMS (APCI) m/z [M+H$^+$] calc. $C_{26}H_{26}$+H$^+$: 339.2108; found: 339.2107.

(*E*)-1,4-diphenyl-3-(*para-tert*-butylphenyl)-penta-1,4-diene **6d** was synthesized according to the general procedure with phenylacetylene (43.9 µL, 0.40 mmol, 1.0 equiv.), *para-tert*-butylphenylallene (61.3 µL, 0.48 mmol, 1.2 equiv.) and iodobenzene (223.8 µL, 2.0 mmol, 5.0 equiv.). The carboboration was performed at room temperature. The crude product was purified by flash column chromatography (*n*-hexane/EtOAc, 200:1) and subsequent Kugelrohr distillation (0.085 mbar, 150 °C). The diene **6d** was isolated as a colorless oil (104.1 mg, 0.296 mmol, 74 %).

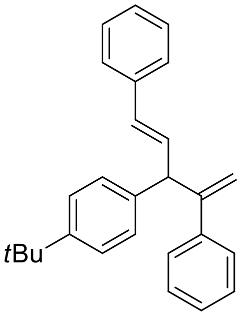

rac

^{1}H NMR (400 MHz, chloroform-*d*) δ 7.37 – 7.33 (m, 2H, Ar-*H*), 7.29 – 7.23 (m, 4H, Ar-*H*), 7.22 – 7.08 (m, 8H, Ar-*H*), 6.49 (dd, *J* = 15.9, 7.3 Hz, 1H, HC=C*H*-CH$_{bn}$), 6.28 (dd, *J* = 15.9, 1.2 Hz, 1H, *H*C=CH-CH$_{bn}$), 5.53 (d, *J* = 1.1 Hz, 1H, C$_{gem,sp2}$*H*$_2$), 5.12 (t, *J* = 1.1 Hz, 1H, C$_{gem,sp2}$*H*$_2$), 4.70 (d, *J* = 7.3 Hz, 1H, *H*$_{bn}$), 1.24 (s, 9H, C(C*H*$_3$)$_3$).

^{13}C NMR (101 MHz, CDCl$_3$) δ 150.33 (CH$_2$*C*$_{sp2}$), 149.36 (Ar-*C*$_q$), 141.85 (Ar-*C*$_q$), 138.94 (Ar-*C*$_q$), 137.56 (Ar-*C*$_q$), 132.54 (CH*C*$_{sp2}$-*H*), 131.12 (Ph*C*$_{sp2}$-*H*), 128.59 (Ar-*C*), 128.39 (Ar-*C*), 128.31 (Ar-*C*), 127.44 (Ar-*C*), 127.30 (Ar-*C*), 126.71 (Ar-*C*), 126.42 (Ar-*C*), 125.48 (Ar-*C*), 115.72 (*C*$_{sp2}$-H$_2$), 52.98 (*C*$_{sp3}$H), 34.52 (*C*(CH$_3$)$_3$), 31.53 (C(*C*H$_3$)$_3$)

HRMS (ESI) m/z [M+H+] calc. for $C_{27}H_{29}^+$: m/z: 353.2264; found: 353.2261.

(*E*)-1,4-diphenyl-3-(*para*-methoxyphenyl)-penta-1,4-diene **6e** was synthesized according to the general procedure with phenylacetylene (43.9 µL, 0.40 mmol, 1.0 equiv.), *p*-methoxy-phenylallene (61.3 µL, 0.48 mmol, 1.2 equiv.) and iodobenzene (223.8 µL, 2.0 mmol, 5.0 equiv.). The carboboration was performed at room temperature. The crude product was purified by flash column chromatography (*n*-hexane/EtOAc, 200:3). The diene **6e** was isolated as a colorless oil (51.4 mg, 0.157 mmol, 39 %).

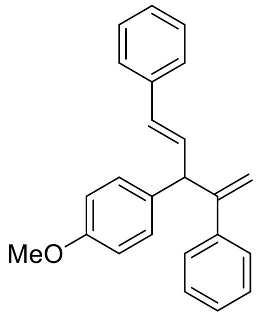

rac

^{1}H NMR (400 MHz, chloroform-*d*) δ 7.46 – 7.36 (m, 4H, Ar-*H*), 7.35 – 7.19 (m, 8H, Ar-*H*), 6.90 – 6.85 (m, 2H, Ar-*H*), 6.59 (dd, *J* = 15.9, 7.0 Hz, 1H, HC=C*H*-CH_{bn}), 6.33 (dd, *J* = 15.9, 1.3 Hz, 1H, *H*C=CH-CH_{bn}), 5.62 (d, *J* = 1.1 Hz, 1H, $C_{gem,sp2}H$), 5.23 (t, *J* = 1.2 Hz, 1H, $C_{gem,sp2}H$), 4.78 (d, *J* = 7.0 Hz, 1H, H_{bn}), 3.81 (s, 3H, OCH_3).

^{13}C NMR (101 MHz, chloroform-*d*) δ 158.35 (Ar-C_q), 150.47 ($C_{q,sp2}$), 141.77 (Ar-C_q), 137.55 (Ar-C_q), 133.91 (Ar-C_q), 132.70 (HC=*C*H-CH_{bn}), 131.14 (H*C*=CH-CH_{bn}), 129.81 (Ar-*C*), 128.61 (Ar-*C*), 128.30 (Ar-*C*), 127.45 (Ar-*C*), 127.33 (Ar-*C*), 126.72 (Ar-*C*), 126.41 (Ar-*C*), 115.52 ($C_{sp2}H_2$), 114.00 (Ar-*C*), 55.32 (OCH_3), 52.62 (C_{bn}).

HRMS (ESI) m/z [M+H+] calc. for $C_{24}H_{23}O^+$: m/z: 327.1744; found: 327.1747.

(*E*)-1,4-diphenyl-2-(*para*-fluorophenyl)-penta-1,4-diene **6f** was synthesized according to the general procedure with phenylacetylene (43.9 µL, 0.40 mmol, 1.0 equiv.), *para*-fluorophenylallene (57.7 mg, 0.48 mmol, 1.2 equiv.) and iodobenzene (223.8 µL, 2.0 mmol, 5.0 equiv.). The carboboration was performed at room temperature. The crude product was purified by flash column chromatography (*n*-hexane/EtOAc, 400:1) and subsequent distillation in a Kugelrohr apparatus (150 °C, 0.09 mbar). The diene **6f** was isolated as colorless oil (91.4 mg, 0.291 mmol, 73 %).

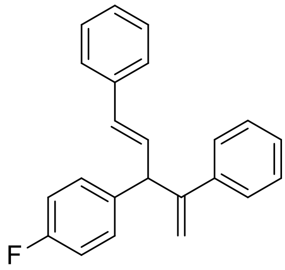

rac

^{1}H NMR (400 MHz, chloroform-*d*) δ 7.38 – 7.31 (m, 4H, Ar-*H*), 7.30 – 7.16 (m, 11H, Ar-*H*), 7.00 – 6.92 (m, 2H, Ar-*H*), 6.52 (dd, *J* = 15.9, 7.0 Hz, 1H, HC=C*H*-CH$_{bn}$), 6.27 (d, *J* = 15.9 Hz, 1H, *H*C=CH-CH$_{bn}$), 5.59 (s, 1H, C$_{gem,sp2}$*H*), 5.19 (s, 1H, C$_{gem,sp2}$*H*), 4.76 (d, *J* = 7.0 Hz, 1H, *H*$_{bn}$).

^{13}C NMR (101 MHz, chloroform-*d*) δ 161.72 (d, *J* = 244.7 Hz, *C*-F), 150.15 (*C*$_{q,sp2}$), 141.46 (Ar-*C*$_{q}$), 137.52 (d, *J* = 3.2 Hz, Ar-*C*$_{q}$), 137.31 (Ar-*C*$_{q}$), 132.14 (HC=*C*H-C$_{bn}$), 131.56 (H*C*=CH-C$_{bn}$), 130.29 (d, *J* = 7.9 Hz, Ar-*C*), 128.67 (Ar-*C*), 128.37 (Ar-*C*), 127.60 (Ar-*C*), 127.51 (Ar-*C*), 126.69 (Ar-*C*), 126.43 (Ar-*C*), 115.84 (*C*$_{sp2}$H$_2$), 115.42 (d, *J* = 21.2 Hz, Ar-*C*), 52.64 (*C*$_{bn}$).

HRMS (APCI) m/z [M+H$^+$] calc. $C_{23}H_{19}F+H^+$: 315.1544; found: 315.1545.

(*E*)-1-(*para*-*n*-butylphenyl)-3-(*para*-*iso*propylphenyl)-3-phenyl-penta-1,4-diene **7a** was synthesized according to the general procedure with *para*-*n*-butylphenylacetylene (63.3 mg, 0.40 mmol, 1.0 equiv.), *para*-*iso*-propylphenylallene (76.0 mg, 0.48 mmol, 1.2 equiv.) and iodobenzene (223.8 µL, 2.0 mmol, 5.0 equiv.). The carboboration was performed at 60 °C. The crude product was purified by flash column chromatography (*n*-hexane/EtOAc, 199:1) and subsequent distillation in a Kugelrohr apparatus (130-165 °C, 0.03 – 0.10 mbar). The diene **7a** was isolated as colorless oil (81.5 mg, 0.207 mmol, 52 %).

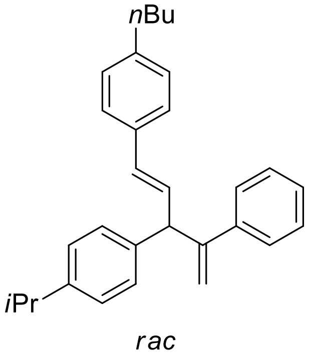

^{1}H NMR (400 MHz, chloroform-*d*) δ 7.41 – 7.37 (m, 2H, Ar-*H*), 7.27 – 7.17 (m, 7H, Ar-*H*), 7.13 (d, *J* = 8.1 Hz, 2H, Ar-*H*), 7.08 (d, *J* = 8.1 Hz, 2H, Ar-*H*), 6.49 (dd, *J* = 15.9, 7.3 Hz, 1H, HC=C*H*-CH_{bn}), 6.30 (d, *J* = 15.9 Hz, 1H, *H*C=CH-CH_{bn}), 5.57 (d, *J* = 0.9 Hz, 1H, $C_{gem,sp2}$*H*), 5.16 (s, 1H, $C_{gem,sp2}$*H*), 4.74 (d, *J* = 7.3 Hz, 1H, H_{bn}), 2.86 (hept, *J* = 6.8 Hz, 1H, C*H*$(CH_3)_2$), 2.56 (t, 2H, CH_2), 1.61 – 1.51 (m, 2H, CH_2), 1.33 (dq, *J* = 14.5, 7.3 Hz, 2H, CH_2), 1.22 (d, *J* = 6.9 Hz, 6H, $CH(CH_3)_2$), 0.90 (t, *J* = 7.3 Hz, 3H, CH_3).

^{13}C NMR (101 MHz, chloroform-*d*) δ 150.50 ($C_{q,sp2}$), 147.03 (Ar-C_q), 142.18 (Ar-C_q), 141.92 (Ar-C_q), 139.49 (Ar-C_q), 135.02 (Ar-C_q), 131.56 (HC=*C*H-C_{bn}), 131.02 (H*C*=CH-C_{bn}), 128.67 (2C, Ar-*C*), 128.29 (Ar-*C*), 127.40 (Ar-*C*), 126.73 (Ar-*C*), 126.60 (Ar-*C*), 126.33 (Ar-*C*), 115.67 ($C_{sp2}H_2$), 53.11 (C_{bn}), 35.48 (CH_2), 33.79 ($CH(CH_3)_2$), 33.76 (CH_2), 24.14 ($CH(CH_3)_2$), 22.46 (CH_2), 14.09 (CH_3).

HRMS (APCI) m/z [M+H$^+$] calc. $C_{30}H_{34}$+H$^+$: 395.2734; found: 395.2733.

(*E*)-1,3-bis(*para-tert*-butylphenyl)-4-phenyl-penta-1,4-diene **7b** was synthesized according to the general procedure with *para-tert*-butylphenylacetylene (63.3 mg, 0.40 mmol, 1.0 equiv.), *para-tert*butylphenylallene (82.7 mg, 0.48 mmol, 1.2 equiv.) and iodobenzene (223.8 µL, 2.0 mmol, 5.0 equiv.). The carboboration was performed at 60 °C. The crude product was purified by flash column chromatography (*n*-hexane/EtOAc, 400:1) and was subsequently distilled twice in a Kugelrohr apparatus (145 – 150 °C, 0.01 - 0.10 mbar). The diene **7b** was isolated as colorless oil (79.6 mg, 0.195 mmol, 49 %).

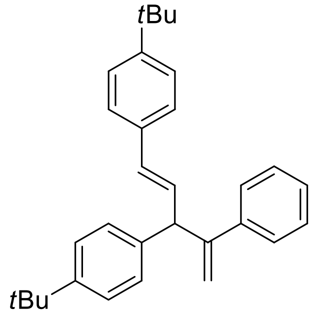

rac

^{1}H NMR (400 MHz, chloroform-*d*) δ 7.49 – 7.45 (m, 2H, Ar-*H*), 7.41 – 7.23 (m, 11H, Ar-*H*), 6.57 (dd, *J* = 15.9, 7.3 Hz, 1H, HC=C*H*-CH_{bn}), 6.39 (d, *J* = 16.4 Hz, 1H, *H*C=CH-CH_{bn}), 5.64 (s, 1H, $C_{gem,sp2}H$), 5.21 (s, 1H, $C_{gem,sp2}H$), 4.81 (d, *J* = 7.3 Hz, 1H, H_{bn}), 1.36 (s, 18H, 2xC(CH_3)$_3$).

^{13}C NMR (101 MHz, chloroform-*d*) δ 150.46 ($C_{q,sp2}$ or Ar-C_q), 150.39 ($C_{q,sp2}$ or Ar-C_q), 149.32 (Ar-C_q), 141.94 (Ar-C_q), 139.15 (Ar-C_q), 134.83 (Ar-C_q), 131.72 (HC=*C*H-C_{bn}), 130.88 (H*C*=CH-C_{bn}), 128.41 (Ar-*C*), 128.30 (Ar-*C*), 127.41 (Ar-*C*), 126.73 (Ar-*C*), 126.15 (Ar-*C*), 125.52 (Ar-*C*), 125.44 (Ar-*C*), 115.73 ($C_{sp2}H_2$), 52.99 (C_{bn}), 34.66 (*C*(CH_3)$_3$), 34.53 (*C*(CH_3)$_3$), 31.54 (C(*C*H_3)$_3$), 31.45 (C(*C*H_3)$_3$).

HRMS (APCI) m/z [M+H$^+$] calc. $C_{31}H_{36}$+H$^+$: 409.2890; found: 409.2891.

(*E*)-1-(4-biphenyl)-3-(*para-tert*-butylphenyl)-4-phenyl-penta-1,4-diene **7c** was synthesized according to the general procedure with 4-biphenylacetylene (71.3 mg, 0.40 mmol, 1.0 equiv.), *para-tert*-butylphenylallene (82.7 mg, 0.48 mmol, 1.2 equiv.) and iodobenzene (223.8 µL, 2.0 mmol, 5.0 equiv.). The carboboration was performed at room temperature. The crude product was purified by flash column chromatography (*n*-hexane/EtOAc, 400:1) and subsequent crystallization of a concentrated solution of DCM layered with *n*-hexane at 3 °C for several days. The diene **7c** was isolated as colorless solid (79.4 mg, 0.185 mmol, 46 %).

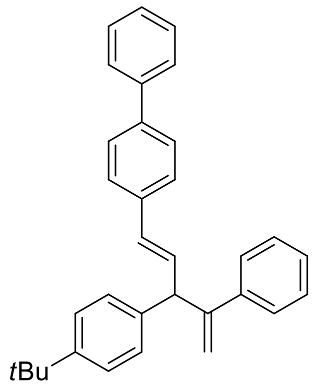

rac

^{1}H NMR (400 MHz, chloroform-*d*) δ 7.63 – 7.59 (m, 2H, Ar-*H*), 7.57 – 7.53 (m, 2H, Ar-*H*), 7.48 – 7.42 (m, 6H, Ar-*H*), 7.38 – 7.22 (m, 8H, Ar-*H*), 6.63 (dd, *J* = 15.9, 7.3 Hz, 1H, HC=C*H*-CH$_{bn}$), 6.41 (d, *J* = 15.9 Hz, 1H, *H*C=CH-CH$_{bn}$), 5.64 (d, *J* = 0.8 Hz, 1H, C$_{gem,sp2}$*H*), 5.22 (s, 1H, C$_{gem,sp2}$*H*), 4.81 (d, *J* = 7.3 Hz, 1H, *H*$_{bn}$), 1.34 (s, 9H, C(C*H*$_3$)$_3$).

^{13}C NMR (101 MHz, Chloroform-*d*) δ 150.33 (*C*$_{q,sp2}$), 149.43 (Ar-*C*$_q$), 141.86 (Ar-*C*$_q$), 140.93 (Ar-*C*$_q$), 140.10 (Ar-*C*$_q$), 138.91 (Ar-*C*$_q$), 136.64 (Ar-*C*$_q$), 132.73 (HC=*C*H-C$_{bn}$), 130.67 (H*C*=CH-C$_{bn}$), 128.90 (Ar-*C*), 128.40 (Ar-*C*), 128.34 (Ar-*C*), 127.47 (Ar-*C*), 127.35 (Ar-*C*), 127.31 (Ar-*C*), 127.05 (Ar-*C*), 126.85 (Ar-*C*), 126.73 (Ar-*C*), 125.51 (Ar-*C*), 115.78 (*C*$_{sp2}$H$_2$), 53.04 (*C*$_{bn}$), 34.55 (*C*(CH$_3$)$_3$), 31.54 (C(*C*H$_3$)$_3$).

HRMS (APCI) m/z [M+H$^+$] calc. $C_{33}H_{32}+H^+$: 429.2577; found: 429.2576.

(*E*)-1-(*para*-fluorophenyl)-3-(*para*-*iso*-propylphenylallene)-4-phenyl-penta-1,4-diene **7d** was synthesized according to the general procedure with *para*-fluorophenylacetylene (48.0 mg, 0.40 mmol, 1.0 equiv.), *para*-*iso*-propylphenylallene (76.0 mg, 0.48 mmol, 1.2 equiv.) and iodobenzene (223.8 µL, 2.0 mmol, 5.0 equiv.). The carboboration was performed at room temperature. The crude product was purified by flash column chromatography (*n*-hexane/EtOAc, 400:1) and subsequent distillation in a Kugelrohr apparatus (140 °C, 0.03 mbar). The diene **7d** was isolated as colorless oil (85.6 mg, 0.240 mmol, 60 %).

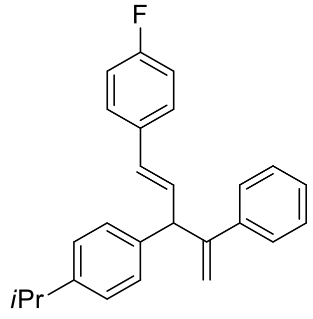

rac

^{1}H NMR (400 MHz, chloroform-*d*) δ 7.41 – 7.36 (m, 2H, Ar-*H*), 7.31 – 7.18 (m, 7H, Ar-*H*), 7.15 (d, *J* = 8.2 Hz, 2H, Ar-*H*), 6.99 – 6.92 (m, 2H, Ar-*H*), 6.45 (dd, *J* = 15.9, 7.2 Hz, 1H, HC=C*H*-CH_{bn}), 6.27 (d, *J* = 15.9 Hz, 1H, *H*C=CH-CH_{bn}), 5.59 (d, *J* = 0.8 Hz, 1H, $C_{gem,sp2}H$), 5.16 (s, 1H, $C_{gem,sp2}H$), 4.74 (d, *J* = 7.2 Hz, 1H, H_{bn}), 2.87 (hept, *J* = 6.9 Hz, 1H, C*H*$(CH_3)_2$), 1.22 (d, *J* = 6.9 Hz, 6H, CH$(CH_3)_2$).

^{13}C NMR (101 MHz, chloroform-*d*) δ 162.23 (d, *J* = 246.2 Hz, *C*-F), 150.34 ($C_{q,sp2}$), 147.18 (Ar-C_q), 141.80 (Ar-C_q), 139.18 (Ar-C_q), 133.73 (d, *J* = 3.3 Hz, (Ar-C_q)), 132.37 (d, *J* = 2.2 Hz, HC=*C*H-C_{bn}), 129.95 (H*C*=CH-C_{bn}), 128.64 (Ar-*C*), 128.33 (Ar-*C*), 127.87 (d, *J* = 7.9 Hz, Ar-*C*), 127.48 (Ar-*C*), 126.71 (Ar-*C*), 126.68 (Ar-*C*), 115.68 ($C_{sp2}H_2$), 115.45 (d, *J* = 21.5 Hz, Ar-*C*), 53.05 (C_{bn}), 33.79 (*C*H$(CH_3)_2$), 24.12 (CH$(CH_3)_2$).

HRMS (APCI) m/z [M+H$^+$] calc. $C_{26}H_{25}F+H^+$:357.2013; found: 357.2015.

(*E*)-1-(*para*-chlorophenyl)-3-(*para*-*iso*-propylphenyl)-4-phenyl-penta-1,4-diene **7e** was synthesized according to the general procedure with *para*-chlorophenylacetylene (54.8 mg, 0.40 mmol, 1.0 equiv.), *para*-*iso*-propylphenylallene (76.0 mg, 0.48 mmol, 1.2 equiv.) and iodobenzene (223.8 µL, 2.0 mmol, 5.0 equiv.). The carboboration was performed at 60 °C. The crude product was purified by flash column chromatography (*n*-hexane/EtOAc, 199:1) and was subsequently distilled twice in a Kugelrohr apparatus (120 - 160 °C, 0.003 – 0.10 mbar). The diene **7e** was isolated as yellowish oil (86.5 mg, 0.232 mmol, 58 %).

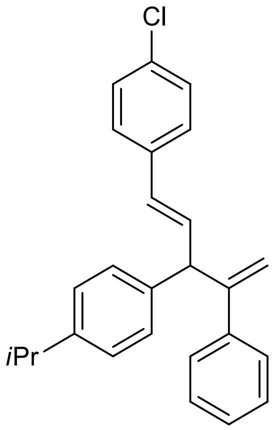

rac

^{1}H NMR (400 MHz, chloroform-*d*) δ 7.41 – 7.37 (m, 2H, Ar-*H*), 7.30 – 7.19 (m, 9H, Ar-*H*), 7.16 (d, *J* = 8.2 Hz, 2H, Ar-*H*), 6.53 (dd, *J* = 15.9, 7.3 Hz, 1H, HC=C*H*-C_{bn}), 6.27 (dd, *J* = 15.9, 1.0 Hz, 1H, *H*C=CH-C_{bn}), 5.60 (d, *J* = 0.9 Hz, 1H, $C_{gem,sp2}H$), 5.16 (s, 1H, $C_{gem,sp2}H$), 4.75 (d, *J* = 7.3 Hz, 1H, H_{bn}), 2.88 (hept, *J* = 6.9 Hz, 1H, C*H*(CH_3)$_2$), 1.24 (d, *J* = 6.9 Hz, 6H, CH(CH_3)$_2$).

^{13}C NMR (101 MHz, chloroform-*d*) δ 150.23 ($C_{q,sp2}$), 147.25 (Ar-C_q), 141.76 (Ar-C_q), 139.00 (Ar-C_q), 136.08 (Ar-C_q), 133.34 (HC=*C*H-C_{bn}), 132.90 (Ar-C_q), 129.94 (H*C*=CH-C_{bn}), 128.73 (Ar-*C*), 128.64 (Ar-*C*), 128.34 (Ar-*C*), 127.64 (Ar-*C*), 127.51 (Ar-*C*), 126.71 (2xAr-*C*), 115.74 ($C_{sp2}H_2$), 53.07 (C_{bn}), 33.80 (*C*H(CH_3)$_2$), 24.12 (CH(CH_3)$_2$).

HRMS (APCI) m/z [M+H$^+$] calc. $C_{26}H_{25}Cl+H^+$: 373.1718; found: 373.1715.

(*E*)-1-(*para*-bromophenyl)-3-(*para*-*iso*-propylphenyl)-4-phenylpenta-1,4-diene **7f** was synthesized according to the general procedure with *para*-bromophenylacetylene (72.4 mg, 0.40 mmol, 1.0 equiv.), *para*-*iso*-propylphenylallene (76.0 mg, 0.48 mmol, 1.2 equiv.) and iodobenzene (223.8 µL, 2.0 mmol, 5.0 equiv.). The carboboration was performed at 60 °C. The crude product was purified by flash column chromatography (*n*-hexane/EtOAc, 99:1) and subsequent distillation in a Kugelrohr apparatus (120 – 150 °C, 0.004 mbar). The diene **7f** was isolated as colorless oil (99.9 mg, 0.239 mmol, 60 %).

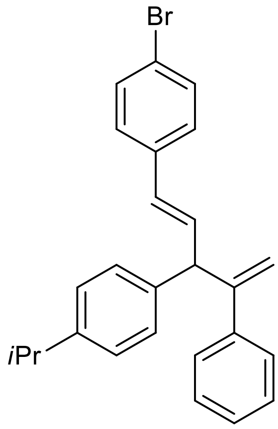

rac

^{1}H NMR (400 MHz, chloroform-*d*) δ 7.44 (ddd, *J* = 8.3, 4.7, 1.9 Hz, 4H, Ar-*H*), 7.34 – 7.19 (m, 9H, Ar-*H*), 6.59 (dd, *J* = 15.9, 7.3 Hz, 1H, HC=C*H*-C_{bn}), 6.30 (d, *J* = 15.8 Hz, 1H, *H*C=CH-C_{bn}), 5.65 (d, *J* =0.8 Hz, 1H, $C_{gem,sp2}$*H*), 5.21 (s, 1H, $C_{gem,sp2}$*H*), 4.79 (d, *J* = 7.2 Hz, 1H, H_{bn}), 2.92 (hept, *J* = 6.9 Hz, 1H, C*H*(CH_3)$_2$), 1.28 (d, *J* = 6.9 Hz, 1H, C*H*(CH_3)$_2$).

^{13}C NMR (101 MHz, chloroform-*d*) δ 150.16 ($C_{q,sp2}$), 147.23 (Ar-C_q), 141.71 (Ar-C_q), 138.93 (Ar-C_q), 136.49 (Ar-C_q), 133.47 (HC=*C*H-C_{bn}), 131.66 (Ar-*C*), 129.98 (H*C*=CH-C_{bn}), 128.64 (Ar-*C*), 128.33 (Ar-*C*), 127.96 (Ar-*C*), 127.50 (Ar-*C*), 126.70 (Ar-*C*), 126.69 (Ar-*C*), 121.00 (Ar-C_q), 115.73 ($C_{sp2}H_2$), 53.05 (C_{bn}), 33.78 (*C*H(CH_3)$_2$), 24.12 (CH(CH_3)$_2$).

HRMS (APCI) m/z [M+H$^+$] calc. $C_{26}H_{25}Br+H^+$: 417.1213; found: 417.1215.

(*E*)-1-(*para*-chlorophenyl)-3,4-diphenyl-penta-1,4-diene **7g** was synthesized according to the general procedure with *para*-chlorophenylacetylene (54.6 mg, 0.40 mmol, 1.0 equiv.), phenylallene (61.3 µL, 0.48 mmol, 1.2 equiv.) and iodobenzene (223.8 µL, 2.0 mmol, 5.0 equiv.). The carboboration was performed at 60 °C. The crude product was purified by flash column chromatography (*n*-hexane/EtOAc, 199:1) and subsequent distillation in a Kugelrohr apparatus (140 °C, 0.1 mbar). The diene **7g** was isolated as colorless oil (67.7 mg, 0.205 mmol, 51 %).

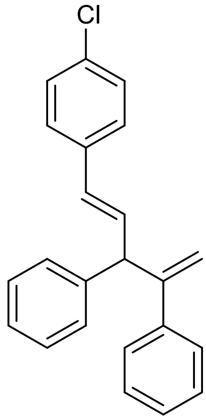

rac

^{1}H NMR (400 MHz, chloroform-*d*) δ 7.38 – 7.34 (m, 2H, Ar-*H*), 7.29 (d, *J* = 4.4 Hz, 2H, Ar-*H*), 7.27 – 7.17 (m, 8H), 6.53 (dd, *J* = 15.9, 7.2 Hz, 1H, HC=C*H*-C_{bn}), 6.24 (dd, *J* = 15.9, 1.2 Hz, 1H, *H*C=CH-C_{bn}), 5.60 (d, *J* = 0.8 Hz, 1H, $C_{gem,sp2}$*H*), 5.18 (s, 1H, $C_{gem,sp2}$*H*), 4.77 (d, *J* = 7.1 Hz, 1H, H_{bn}).

^{13}C NMR (101 MHz, chloroform-*d*) δ 150.07 ($C_{q,sp2}$), 141.67 (Ar-C_q), 141.59 (Ar-C_q), 135.98 (Ar-C_q), 133.11 (HC=*C*H-C_{bn}), 132.98 (Ar-C_q), 130.22 (H*C*=CH-C_{bn}), 128.84 (Ar-*C*), 128.76 (Ar-*C*), 128.67 (Ar-*C*), 128.35 (Ar-*C*), 127.64 (Ar-*C*), 127.56 (Ar-*C*), 126.77 (Ar-*C*), 126.71 (Ar-*C*), 115.83 ($C_{sp2}H_2$), 53.44 (C_{bn}).

HRMS (APCI) m/z [M+H$^+$] calc. $C_{23}H_{19}Cl+H^+$: 331.1248; found: 331.1248.

(*E*)-1-(cyclohex-1-en-1-yl)-3-(*para-tert*-butylphenyl)-4-phenyl-penta-1,4-diene **7h** was synthesized according to the general procedure with 1-ethinyl-cyclohex-1-ene (42.5 mg, 0.40 mmol, 1.0 equiv.), *para-tert*-butyl-phenylallene (82.7 mg, 0.48 mmol, 1.2 equiv.) and iodobenzene (223.8 µL, 2.0 mmol, 5.0 equiv.). The carboboration was performed at 60 °C. The crude product was purified by flash column chromatography (*n*-hexane/EtOAc, 400:1) and subsequent distillation in a Kugelrohr apparatus (125 - 150°C, 0.008-0.010 mbar). The diene **7h** was isolated as colorless oil (55.2 mg, 0.155 mmol, 39 %).

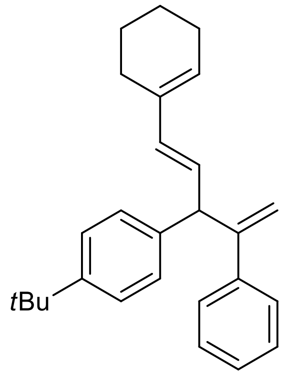

rac

^{1}H NMR (400 MHz, Chloroform-*d*) δ 7.40 – 7.34 (m, 2H, Ar-*H*), 7.30 – 7.15 (m, 7H, Ar-*H*), 5.99 (d, *J* = 15.7 Hz, 1H, *H*C=CH-CH$_{bn}$), 5.85 (dd, *J* = 15.7, 7.3 Hz, 1H, HC=C*H*-CH$_{bn}$), 5.63 (t, *J* = 3.7 Hz, 1H, C$_{sp2}$*H*), 5.53 (d, *J* = 1.0 Hz, 1H, C$_{gem,sp2}$*H*), 5.12 (s, 1H, C$_{gem,sp2}$*H*), 4.61 (d, *J* = 7.3 Hz, 1H, *H*$_{bn}$), 2.17 – 2.05 (m, 4H, C*H*$_2$), 1.68 – 1.52 (m, 4H, C*H*$_2$), 1.28 (s, 1H, C(C*H*$_3$)$_3$).

^{13}C NMR (101 MHz, Chloroform-*d*) δ 150.84 (*C*$_{q,sp2}$), 149.09 (Ar-*C*$_q$), 142.10 (Ar-*C*$_q$), 139.64 (Ar-*C*$_q$), 135.62 (*C*$_{q,sp2}$), 134.67 (H*C*=CH-C$_{bn}$), 128.71 (*C*$_{sp2}$H), 128.38 (HC=*C*H-C$_{bn}$), 128.25 (Ar-*C*), 128.23 (Ar-*C*), 127.30 (Ar-*C*), 126.73 (Ar-*C*), 125.37 (Ar-*C*), 115.33 (*C*$_{sp2}$H$_2$), 52.96 (*C*$_{bn}$), 34.50 (*C*(CH$_3$)$_3$), 31.54 (C(*C*H$_3$)$_3$), 25.95 (*C*H$_2$), 24.87 (*C*H$_2$), 22.71 (*C*H$_2$), 22.66 (*C*H$_2$).

HRMS (APCI) m/z [M+H$^+$] calc. $C_{27}H_{32}$+H$^+$: 357.2577; found: 357.2576.

(*E*)-1-adamantyl-3-(*para*-*iso*-propylphenyl)-3-phenyl-penta-1,4-diene **7i** was synthesized according to the general procedure with adamantylacetylene (64.1 mg, 0.40 mmol, 1.0 equiv.), *para*-*iso*-propylphenylallene (76.0 mg, 0.48 mmol, 1.2 equiv.) and iodobenzene (223.8 µL, 2.0 mmol, 5.0 equiv.). The carboboration was performed at 60 °C for 3 days. The crude product was purified by flash column chromatography (*n*-hexane/EtOAc, 500:1) and was subsequently distilled twice in a Kugelrohr apparatus (150 °C, 0.03 mbar). The diene **7i** was isolated as colorless oil (57.1 mg, 0.144 mmol, 36 %).

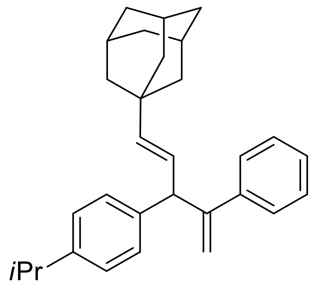

rac

^{1}H NMR (600 MHz, chloroform-*d*) δ 7.40 – 7.37 (m, 2H, Ar-*H*), 7.27 (td, *J* = 6.7, 6.2, 1.5 Hz, 2H, Ar-*H*), 7.24 – 7.20 (m, 1H, Ar-*H*), 7.19 (d, *J* = 8.2 Hz, 2H, Ar-*H*), 7.15 (d, *J* = 8.2 Hz, 2H, Ar-*H*), 5.61 (dd, *J* = 15.7, 7.6 Hz, 1H, HC=C*H*-CH_{bn}), 5.51 (s, 1H, $C_{gem,sp2}H$), 5.34 (d, *J* = 15.7 Hz, 1H, *H*C=CH-CH_{bn}), 5.07 (s, 1H, $C_{gem,sp2}H$), 4.54 (d, *J* = 7.6 Hz, 1H, H_{bn}), 2.89 (hept, *J* = 6.9 Hz, 1H, C*H*$(CH_3)_2$), 1.98 (s, 3H, H_{Ad}), 1.73 (d, *J* = 12.1 Hz, 3H, H_{Ad}), 1.66 (d, *J* = 11.3 Hz, 3H, H_{Ad}), 1.58 (d, *J* = 2.4 Hz, 6H, H_{Ad}), 1.26 (d, *J* = 6.9 Hz, 6H, $CH(CH_3)_2$).

^{13}C NMR (151 MHz, chloroform-*d*) δ 151.42 ($C_{q,sp2}$), 146.61 (Ar-C_q), 143.50 (H*C*=CH-C_{bn}), 142.26 (Ar-C_q), 140.34 (Ar-C_q), 128.54 (Ar-*C*), 128.12 (Ar-*C*), 127.18 (Ar-*C*), 126.91 (HC=*C*H-C_{bn}), 126.81 (Ar-*C*), 126.38 (Ar-*C*), 115.11 ($C_{sp2}H_2$), 52.90 (C_{bn}), 42.49 ($C_{Ad}H_2$), 37.06 ($C_{Ad}H_2$), 34.96 ($C_{q,Ad}$), 33.75 (*C*H$(CH_3)_2$, 28.65 (C_{Ad}H), 24.15 (*C*H$(CH_3)_2$.

HRMS (APCI) m/z [M+H$^+$] calc. $C_{30}H_{36}$+H$^+$: 397.2890; found: 397.2888.

(E)-1-phenyl-3-(*para*-*iso*-propylphenyl)-4-(3-pyridyl)-penta-1,4-diene **8a** was synthesized according to the general procedure with phenylacetylene (40.9 µL, 0.40 mmol, 1.0 equiv.), *para*-*iso*-propylphenylallene (76.0 mg, 0.48 mmol, 1.2 equiv.) and 3-iodopyridin (410.0 mg, 2.0 mmol, 5.0 equiv.). The carboboration was performed at 60 °C. The crude product was purified by flash column chromatography (*n*-hexane/EtOAc, 9:1) and subsequent distillation in a Kugelrohr apparatus (120 - 150°C, 0.007 mbar). The diene **8a** was isolated as colorless oil (72.7 mg, 0.214 mmol, 54 %).

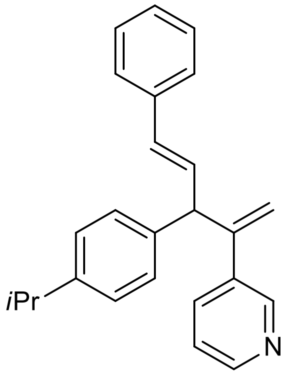

rac

^{1}H NMR (400 MHz, chloroform-*d*) δ 8.67 (d, J = 1.3 Hz, 1H, Ar_{Py}-H), 8.46 (d, J = 3.7 Hz, 1H, Ar_{Py}-H), 7.65 (dt, J = 7.9, 1.8 Hz, 1H, Ar_{Py}-H), 7.36 (d, J = 7.3 Hz, 2H, Ar-H), 7.30 (t, J = 7.5 Hz, 2H, Ar-H), 7.20 (ddd, J = 18.6, 7.9, 3.3 Hz, 6H, Ar-H and Ar_{Py}-H), 6.54 (dd, J = 15.9, 7.3 Hz, 1H, HC=CH-C_{bn}), 6.36 (d, J = 15.9 Hz, 1H, HC=CH-C_{bn}), 5.63 (s, 1H, $C_{gem,sp2}H$), 5.32 (s, 1H, $C_{gem,sp2}H$), 4.73 (d, J = 7.3 Hz, 1H, H_{bn}), 2.89 (hept, J = 6.9 Hz, 1H, CH(CH_3)$_2$), 1.24 (d, J = 6.9 Hz, 6H, CH(CH_3)$_2$).

^{13}C NMR (101 MHz, chloroform-*d*) δ 148.46 (Ar_{Py}-C), 148.11 (Ar_{Py}-C), 147.55 ($C_{q,sp2}$), 147.43 (Ar-C_q), 138.36 (Ar-C_q), 137.26 (Ar-C_q), 137.20 (Ar-C_q), 133.98 (Ar_{Py}-C), 131.66 (C_{sp2}H), 131.60 (C_{sp2}H), 128.61 (Ar-C), 128.58 (Ar-C), 127.49 (Ar-C), 126.80 (Ar-C), 126.42 (Ar-C), 123.11 (Ar_{Py}-C), 117.12 ($C_{sp2}H_2$), 53.04 (C_{bn}), 33.76 (CH(CH_3)$_2$), 24.07 (CH(CH_3)$_2$).

HRMS (ESI) m/z [M+H$^+$] calc. $C_{25}H_{25}N+H^+$: 340.2060; found: 340.2034.

(*E*)-1-phenyl-3-(*para*-*iso*-propylphenyl)-4-(*para*-cyanophenyl)-penta-1,4-diene **8b** was synthesized according to the general procedure with phenylacetylene (40.9 µL, 0.40 mmol, 1.0 equiv.), *para*-*iso*-propylphenylallene (76.0 mg, 0.48 mmol, 1.2 equiv.) and iodobenzene (458.0 mg, 2.0 mmol, 5.0 equiv.). The carboboration was performed at 60 °C. The crude product was purified by flash column chromatography (*n*-hexane/EtOAc, 49:1), subsequent distillation in a Kugelrohr apparatus (125 - 175°C, 0.006 mbar) and crystallization from *n*-hexane at -23 °C for several days. The diene **8b** was isolated as colorless solid (57.0 mg, 0.157 mmol, 39 %).

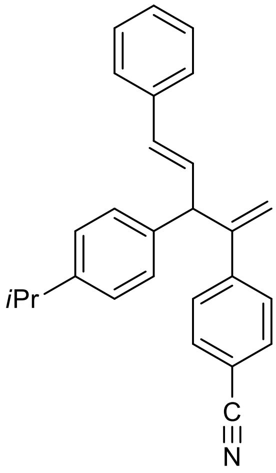

rac

^{1}H NMR (400 MHz, chloroform-*d*) δ 7.57 – 7.53 (m, 2H, Ar-*H*), 7.51 – 7.46 (m, 2H, Ar-*H*), 7.38 – 7.28 (m, 4H, Ar-*H*), 7.25 – 7.16 (m, 5H, Ar-*H*), 6.52 (dd, *J* = 15.9, 7.3 Hz, 1H, HC=C*H*-C_{bn}), 6.35 (d, *J* = 16.0 Hz, 1H, *H*C=CH-C_{bn}), 5.68 (s, 1H, $C_{gem,sp2}H$), 5.36 (s, 1H, $C_{gem,sp2}H$), 4.74 (d, *J* = 7.2 Hz, 1H, H_{bn}), 2.90 (hept, *J* = 6.9 Hz, 1H, C*H*(CH)$_3$)$_2$), 1.25 (d, *J* = 6.9 Hz, 6H, CH(C*H*)$_3$)$_2$).

^{13}C NMR (101 MHz, chloroform-*d*) δ 149.13 ($C_{q,sp2}$), 147.54 (Ar-C_q), 146.29 (Ar-C_q), 138.34 (Ar-C_q), 137.19 (Ar-C_q), 132.17 (Ar-*C*), 131.72 (H*C*=CH-C_{bn}), 131.55 (HC=*C*H-C_{bn}), 128.66 (Ar-*C*), 128.53 (Ar-*C*), 127.57 (Ar-*C*), 127.40 (Ar-*C*), 126.85 (Ar-*C*), 126.43 (Ar-*C*), 119.00 (*C*N), 118.21 ($C_{sp2}H_2$), 110.99 (Ar-C_q), 52.84 (C_{bn}), 33.77 (*C*H(CH$_3$)$_2$), 24.08 (CH(*C*H$_3$)$_2$).

HRMS (ESI) m/z [M+H$^+$] calc. $C_{27}H_{25}N+Na^+$: 386.1879; found: 386.1874.

(*E*)-1-phenyl-3,4-di-(4-biphenyl)-penta-1,4-diene **8c** was synthesized according to the general procedure with phenylacetylene (40.9 µL, 0.40 mmol, 1.0 equiv.), *para*-phenyl-phenylallene (92.3 mg, 0.48 mmol, 1.2 equiv.) and 4-iodobiphenyl (560.2 mg, 2.0 mmol, 5.0 equiv.). The carboboration was performed at 60 °C. The crude product was purified by flash column chromatography (*n*-hexane/EtOAc, 400:1) and subsequent crystallization of a concentrated solution in DCM layered with *n*-hexane at 3 °C for several days. The diene **8c** was isolated as colorless solid (86.0 mg, 0.192 mmol, 48 %).

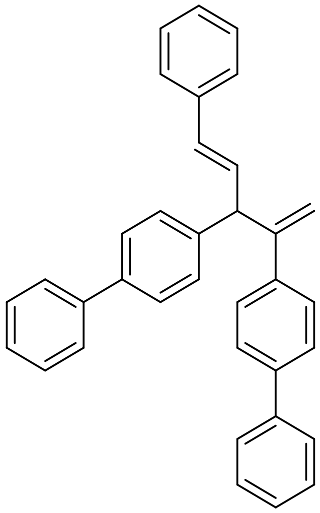

rac

^{1}H NMR (700 MHz, chloroform-*d*) δ 7.63 – 7.61 (m, 2H, Ar-*H*), 7.60 – 7.57 (m, 4H,Ar-*H*), 7.55 – 7.52 (m, 4H, Ar-*H*), 7.46 – 7.42 (m, 6H, Ar-*H*), 7.41 (d, *J* = 7.4 Hz, 2H, Ar-*H*), 7.36 – 7.31 (m, 4H, Ar-*H*), 7.24 (t, *J* = 7.4 Hz, 1H, Ar-*H*), 6.64 (dd, *J* = 15.9, 7.2 Hz, 1H, HC=C*H*-C_{bn}), 6.41 (d, *J* = 15.9 Hz, 1H, *H*C=CH-C_{bn}), 5.74 (s, 1H, $C_{gem,sp2}$*H*), 5.31 (s, 1H, $C_{gem,sp2}$*H*), 4.91 (d, *J* = 7.2 Hz, 1H, H_{bn}).

^{13}C NMR (176 MHz, chloroform-*d*) δ 149.56 ($C_{q,sp2}$), 141.07 (Ar-C_q), 140.97 (Ar-C_q), 140.76 (Ar-C_q), 140.47 (Ar-C_q), 140.31 (Ar-C_q), 139.53 (Ar-C_q), 137.45 (Ar-C_q), 132.22 (HC=*C*H-C_{bn}), 131.54 (H*C*=CH-C_{bn}), 129.27 (Ar-*C*), 128.88 (Ar-*C*), 128.86 (Ar-*C*), 128.66 (Ar-*C*), 127.46 (Ar-*C*), 127.40 (Ar-*C*), 127.38 (Ar-*C*), 127.28 (Ar-*C*), 127.14 (Ar-*C*), 127.10 (Ar-*C*), 127.08 (Ar-*C*), 127.07 (Ar-*C*), 126.48 (Ar-*C*), 115.89 ($C_{sp2}H_2$), 53.02 (C_{bn}).

HRMS (APCI) m/z [M+H$^+$] calc. $C_{35}H_{28}$+H$^+$: 449.2264; found: 449.2263.

(E)-1-(4-biphenyl)-3-(*para-iso*-propylphenyl)-3-phenyl-penta-1,4-diene **9** was synthesized according to the general procedure with *para*-pinacolboranephenylacetylene (91.2 mg, 0.40 mmol, 1.0 equiv.), *para-iso*-propylphenylallene (76.0 mg, 0.48 mmol, 1.2 equiv.) and iodobenzene (223.8 µL, 2.0 mmol, 5.0 equiv.). The carboboration was performed at 60 °C. The crude product was purified by flash column chromatography (*n*-hexane/EtOAc, gradient 399:1 – 99:1) and subsequent crystallization of a concentrated solution of DCM layered with *n*-hexane at 3 °C for two days. The diene was isolated as colorless solid (67.4 mg, 0.163 mmol, 41 %).

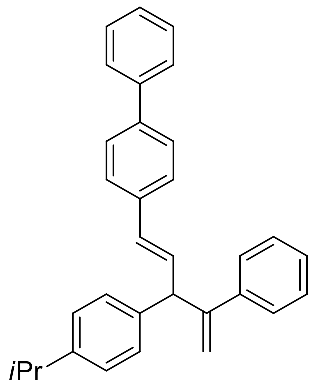

rac

^{1}H NMR (400 MHz, chloroform-*d*) δ 7.62 – 7.58 (m, 2H, Ar-*H*), 7.55 (d, J = 8.3 Hz, 2H, Ar-*H*), 7.47 – 7.42 (m, 6H, Ar-*H*), 7.38 – 7.32 (m, 1H, Ar-*H*), 7.32 – 7.22 (m, 5H, Ar-*H*), 7.21 – 7.16 (m, 2H, Ar-*H*), 6.62 (dd, J = 15.9, 7.3 Hz, 1H, HC=C*H*-CH$_{bn}$), 6.40 (d, J = 15.8 Hz, 1H, *H*C=CH-CH$_{bn}$), 5.63 (s, 1H, C$_{gem,sp2}$*H*), 5.22 (s, 1H, C$_{gem,sp2}$*H*), 4.81 (d, J = 7.3 Hz, 1H, *H*$_{bn}$), 2.91 (hept, J = 6.9 Hz, 1H, C*H*(CH$_3$)$_2$), 1.26 (d, J = 6.9 Hz, 6H, CH(C*H*$_3$)$_2$).

^{13}C NMR (101 MHz, chloroform-*d*) δ 150.38 (*C*$_{q,sp2}$), 147.15 (Ar-*C*$_q$), 141.86 (Ar-*C*$_q$), 140.93 (Ar-*C*$_q$), 140.11 (Ar-*C*$_q$), 139.28 (Ar-*C*$_q$), 136.64 (Ar-*C*$_q$), 132.75 (HC=*C*H-C$_{bn}$), 130.69 (H*C*=CH-C$_{bn}$), 128.90 (Ar-*C*), 128.69 (Ar-*C*), 128.33 (Ar-*C*), 127.46 (Ar-*C*), 127.35 (Ar-*C*), 127.31 (Ar-*C*), 127.05 (Ar-*C*), 126.85 (Ar-*C*), 126.74 (Ar-*C*), 126.66 (Ar-*C*), 115.75 (*C*$_{sp2}$H$_2$), 53.16 (*C*$_{bn}$), 33.80 (*C*H(CH$_3$)$_2$), 24.14 (*C*H(CH$_3$)$_2$).

HRMS (APCI) m/z [M+H$^+$] calc. $C_{32}H_{30}$+H$^+$: 415.2421; found: 415.2423.

Inside the glovebox Piers' borane (311.3 mg, 0.90 mmol, 2.25 equiv.) and *para*-fluorophenylacetylene (108.1 mg, 0.90 mmol, 2.25 equiv.) were dissolved in dry DCM (7.0 mL) in a Schlenk tube with J Young valve and stirred for 30 min at room temperature. Diallene **10** (61.7 mg, 0.40 mmol, 1.00 equiv.) was added, the tube was taken out of the glovebox and heated to 60 °C for 24 h. The solvent was evaporated under oil-pump vacuum under inert conditions. Inside the glovebox the residue was transferred to a pressure tube, dissolved in dry THF (40 mL) and $Pd(PPh_3)_4$ (46.2 mg, 0.04 mmol, 0.10 equiv.) was added. Under N_2 flow degassed aqueous NaOH (3 M, 12 mL) and iodobenzene (447.6 µL, 4.0 mmol, 10.0 equiv) were added and the reaction mixture was stirred under inert conditions for 7 h at 70 °C. The reaction mixture was transferred to a separation funnel and extracted with *n*-hexane three times. The combined organic phases were dried over Na_2SO_4, filtered and the solvent was evaporated under reduced pressure. The crude product was purified by column chromatography (*n*-hexane/EtOAc 500:1) and subsequent crystallization of a saturated DCM solution layered with *n*-hexane at 3 °C for several days. The product 1,4-bis((*E*)-1-(4-fluorophenyl)-4-phenylpenta-1,4-dien-3-yl)benzene **11** was isolated as a colorless solid (85.9 mg, 0.156 mmol, 39 %).

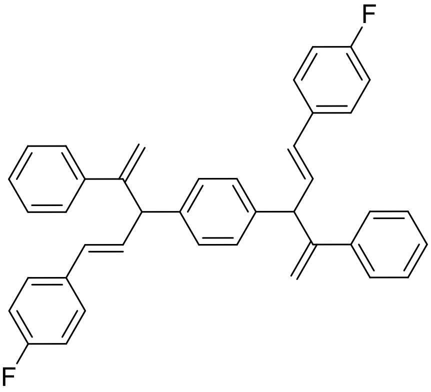

Remark: The product was isolated as a racemic mixture of the (*S*,*S*) and (*R*,*R*) enantiomers and the *meso* product in a ratio of about 1:1 (see chromatogram Figure SI 175). Most signals of both diastereomers in the ^{1}H and ^{13}C NMR spectra overlap completely. If not otherwise stated (see footnote) the overlapping signals are listed.

^{1}H NMR (700 MHz, chloroform-*d*) δ 7.35 – 7.33 (m, 8H, Ar-*H*), 7.29 (dd, *J* = 8.1, 5.6 Hz, 8H, Ar-*H*), 7.23 (q, *J* = 7.2, 6.6 Hz, 20H, Ar-*H*), 6.97 (t, *J* = 8.6 Hz, 8H, Ar-*H*), 6.45 (dd, *J* = 15.9, 7.2 Hz, 2H, HC=C*H*-CH_{bn})[a], 6.45 (dd, *J* = 15.9, 7.2 Hz, 2H, HC=C*H*-CH_{bn})[a], 6.24 (d, *J* = 15.9 Hz, 2H, *H*C=CH-CH_{bn})[a], 6.23 (d, *J* = 15.9 Hz, 2H, *H*C=CH-CH_{bn})[a], 5.57 (s, 2H, $C_{gem,sp2}H$)[a], 5.56 (s, 2H, $C_{gem,sp2}H$)[a], 5.16 (s, 2H, $C_{gem,sp2}H$)[a], 5.15 (s, 2H, $C_{gem,sp2}H$)[a], 4.73 (d, *J* = 7.2 Hz, 4H, H_{bn}).

^{13}C NMR (176 MHz, chloroform-*d*) δ 162.26 (d, *J* = 246.3 Hz, 4C, *C*F), 150.32 (2C, $C_{q,sp2}$)[a], 150.27 (2C, $C_{q,sp2}$)[a], 141.69 (2C, Ar-C_q)[a], 141.67 (2C, Ar-C_q)[a], 140.13 (2C, Ar-C_q)[a], 140.11 (2C, Ar-C_q)[a], 133.62 (d, *J* = 3.2 Hz, 4C, Ar-C_q), 132.04 (d, *J* = 1.9 Hz, 2C, HC=*C*H-C_{bn})[a], 132.01 (d, *J* = 1.9 Hz, 2C, HC=*C*H-C_{bn})[a], 130.23 (2C, H*C*=CH-C_{bn})[a], 130.20 (2C, H*C*=CH-C_{bn})[a], 128.94 (8C, Ar-*C*), 128.28 (8C, Ar-*C*), 127.87 (d, *J* = 8.0 Hz, 8C, Ar-*C*), 127.47 (4C, Ar-*C*), 126.79 (4C, Ar-*C*)[a], 126.78 (4C, Ar-*C*)[a], 115.69 (2C, $C_{sp2}H_2$)[a], 115.68 (2C, $C_{sp2}H_2$)[a], 115.49 (d, *J* = 21.5 Hz, 8C, Ar-*C*), 53.16 (4C, C_{bn}).

Footnote a: separated signals belonging to one of the two possible diastereomers.

HRMS (APCI) m/z [M+H$^+$] calc. $C_{32}H_{30}$+H$^+$: 551.2545; found: 551.2544.

9.3 Additional NMR spectra of 1,4-diene products

9.3.1 1,4-diene 6a

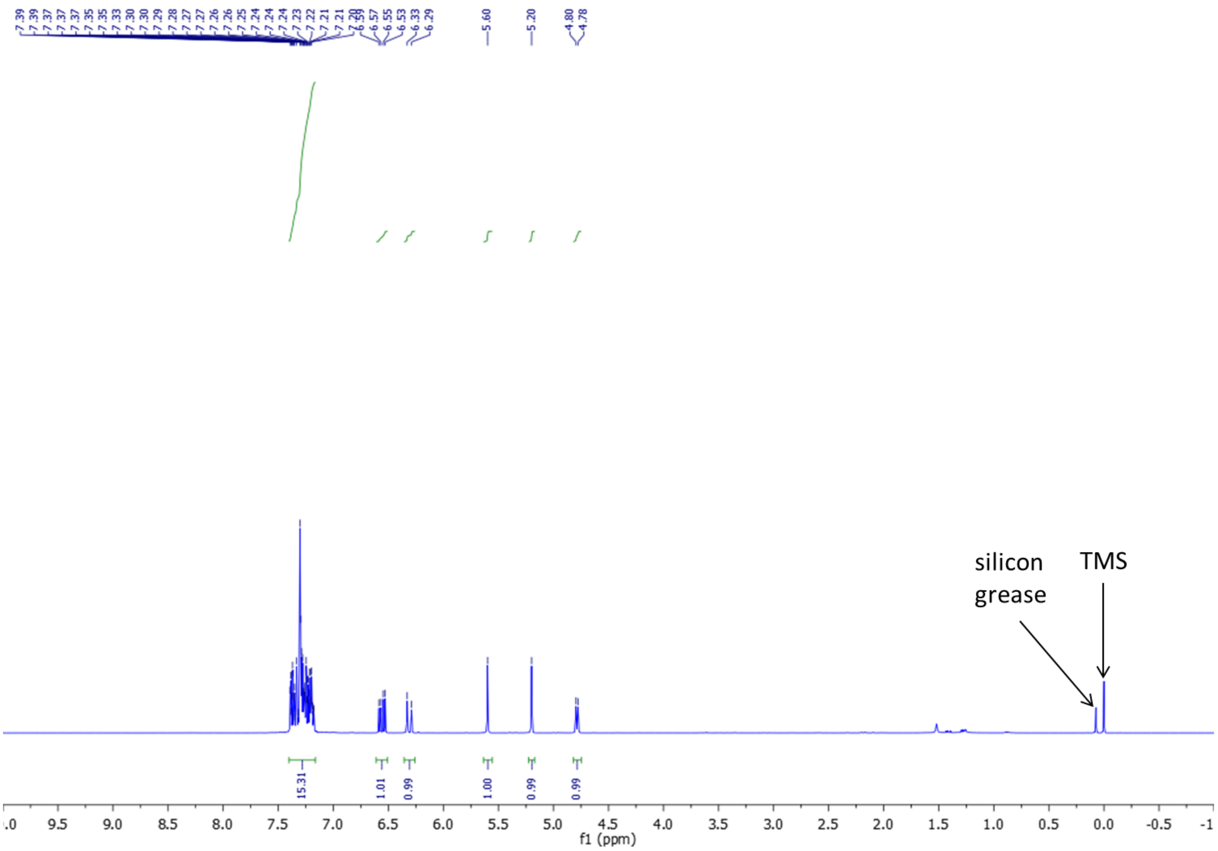

Figure SI 30: ^{1}H NMR spectrum of (*E*)-1,3,4-triphenyl-penta-1,4-diene **6a** (400 MHz, chloroform-*d*).

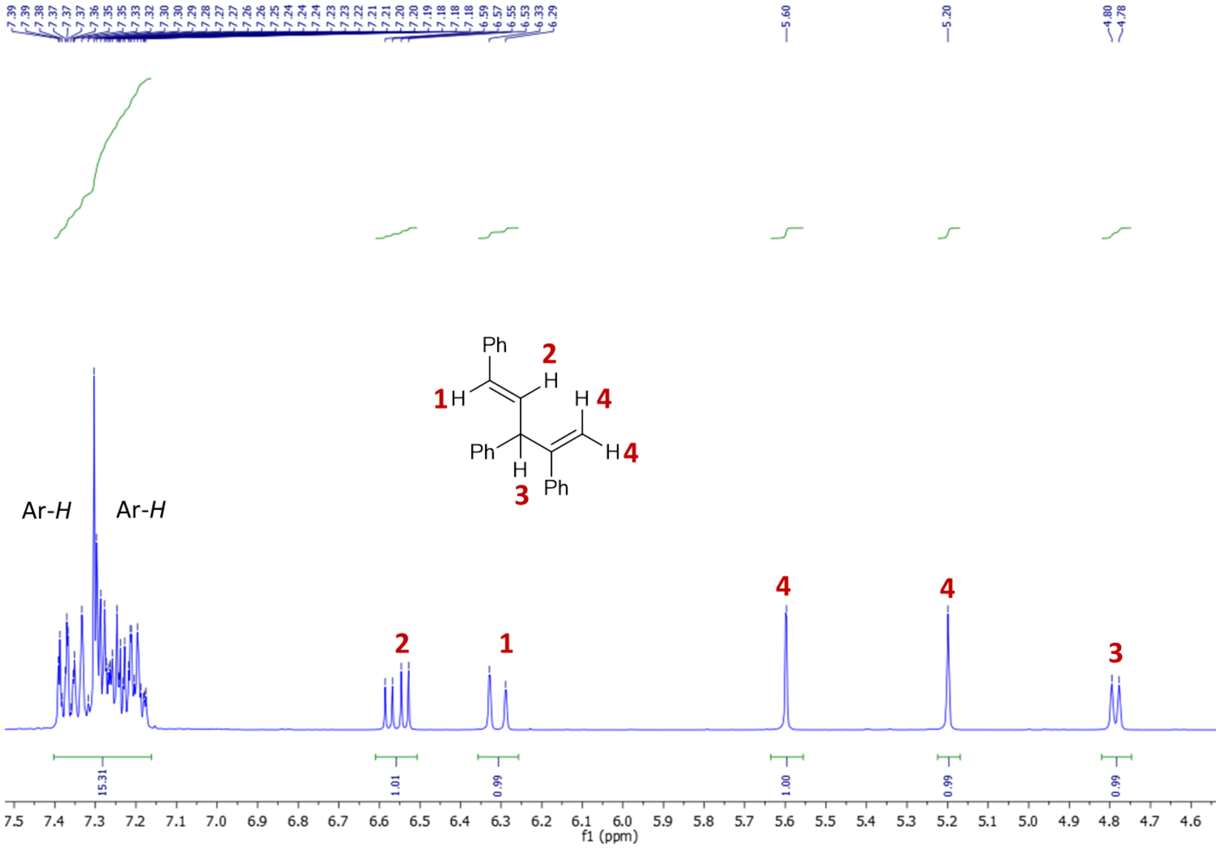

Figure SI 31: Excerpt of ^{1}H NMR spectrum of (*E*)-1,3,4-triphenyl-penta-1,4-diene **6a** (400 MHz, chloroform-*d*).

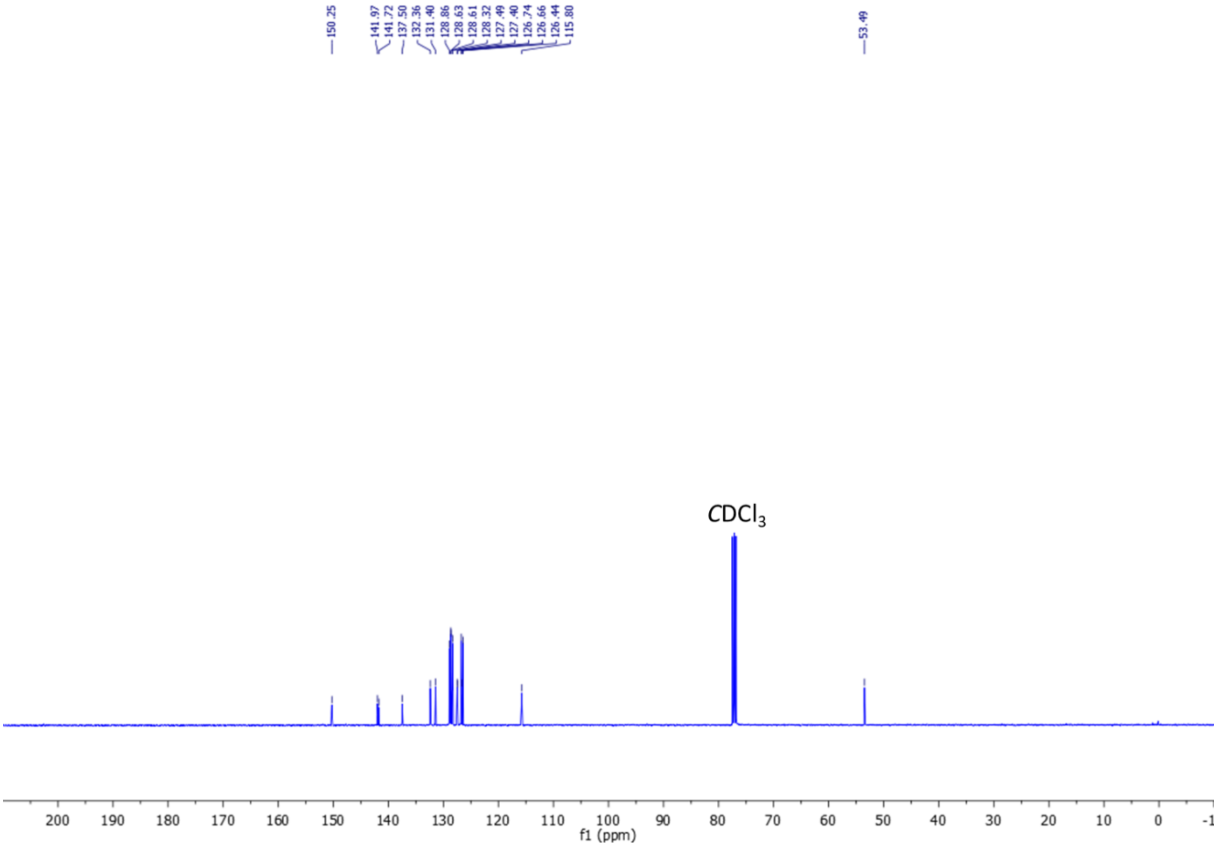

Figure SI 32: ^{13}C NMR spectrum of (*E*)-1,3,4-triphenyl-penta-1,4-diene **6a** (101 MHz, chloroform-*d*).

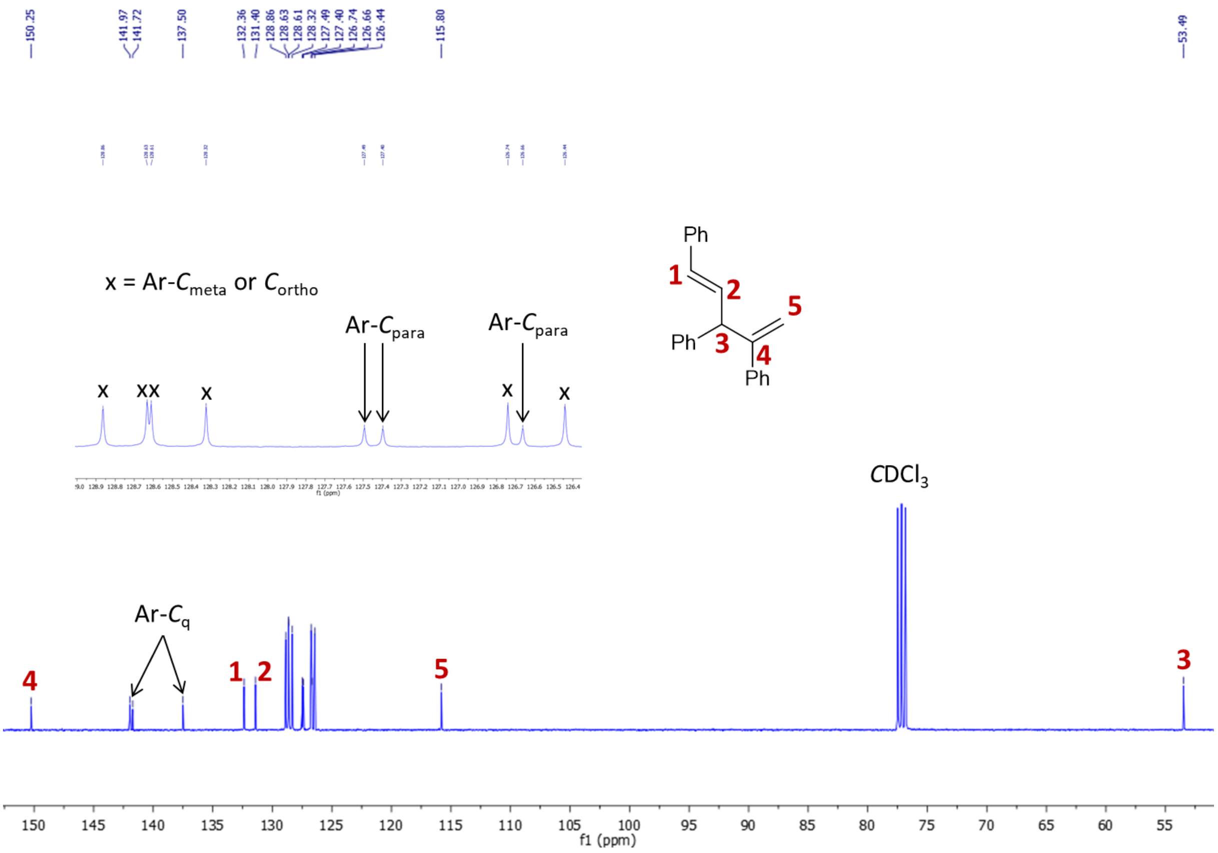

Figure SI 33: Excerpt of the ^{13}C NMR spectrum of (*E*)-1,3,4-triphenyl-penta-1,4-diene **6a** (101 MHz, chloroform-*d*).

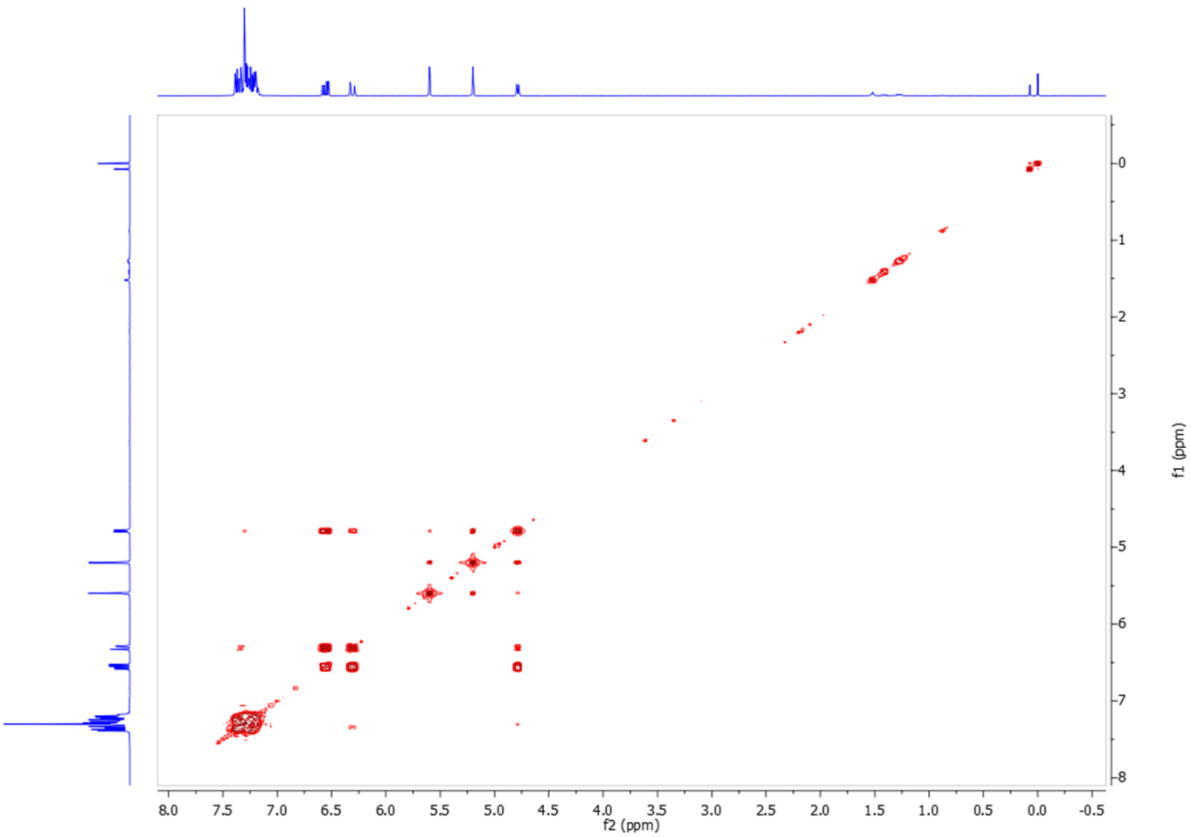

Figure SI 34: HH COSY NMR spectrum of (*E*)-1,3,4-triphenyl-penta-1,4-diene **6a** (400 MHz, chloroform-*d*).

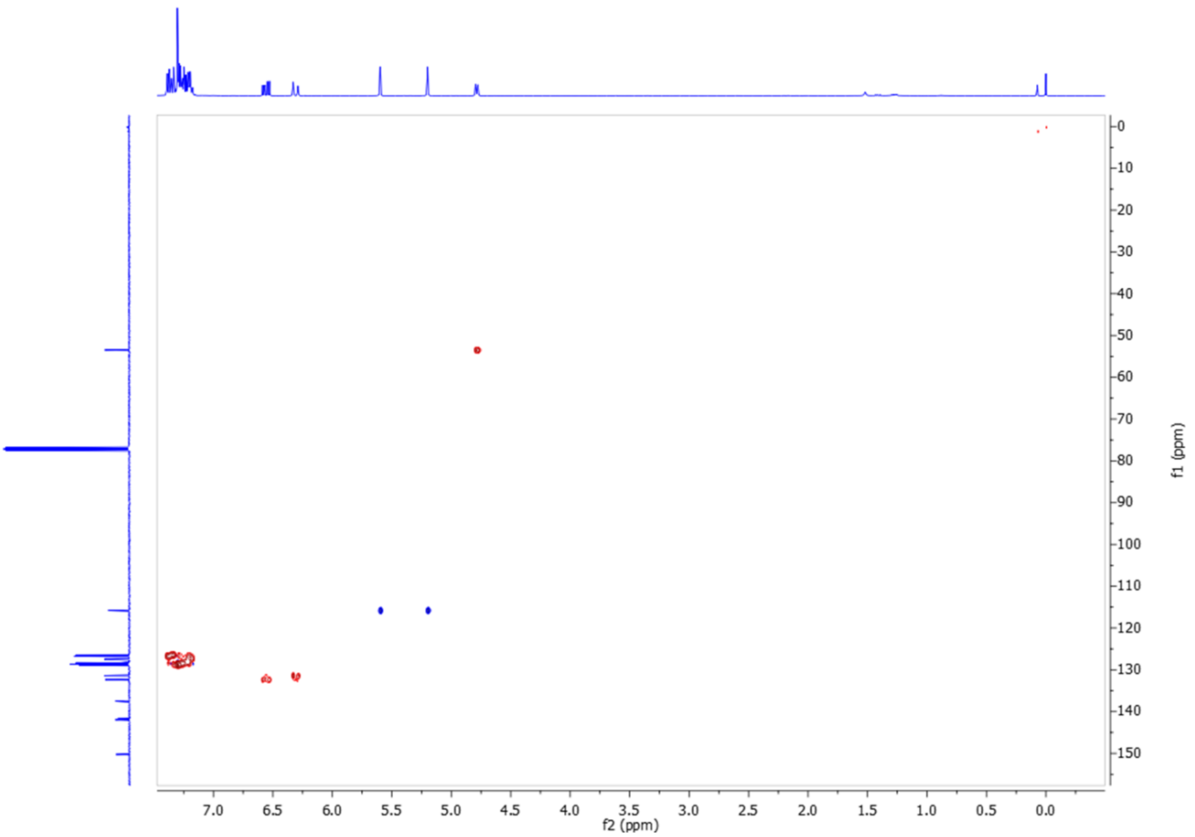

Figure SI 35: HSQC NMR spectrum of (*E*)-1,3,4-triphenyl-penta-1,4-diene **6a** (101 MHz, chloroform-*d*).

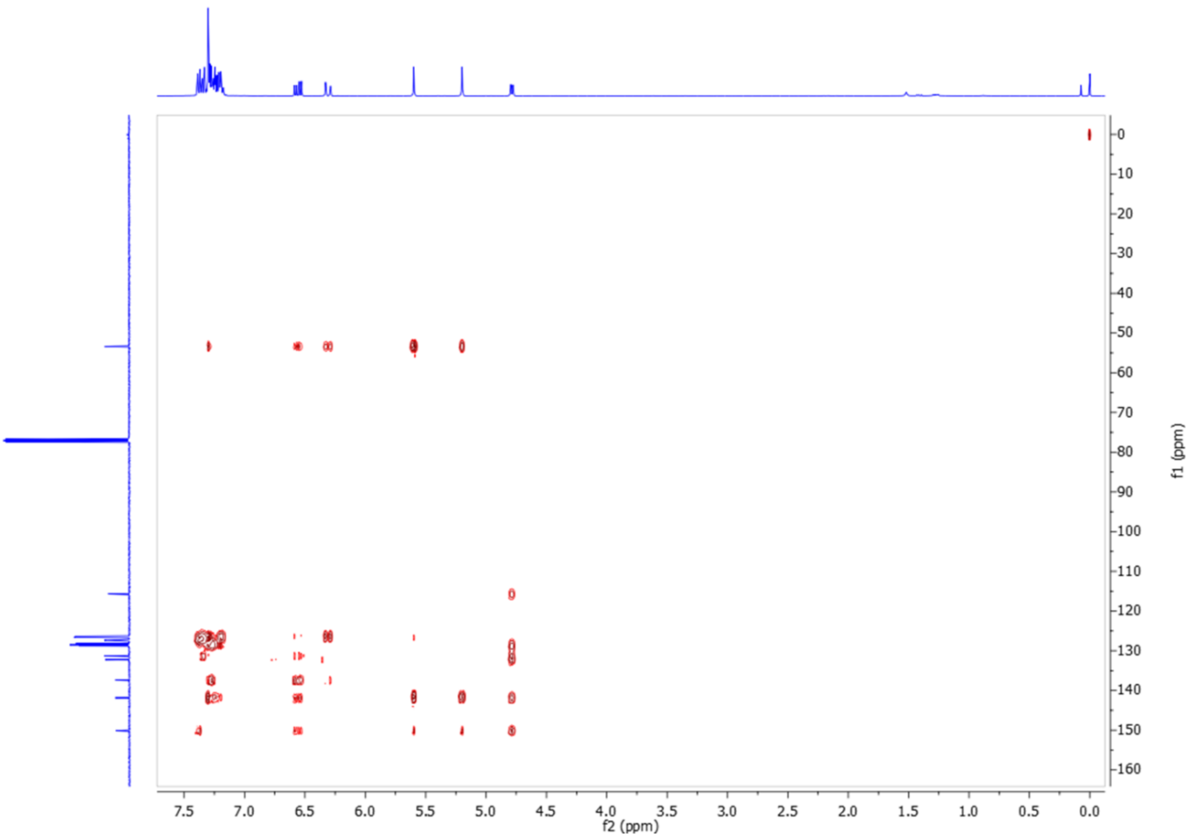

Figure SI 36: HMBC NMR spectrum of (*E*)-1,3,4-triphenyl-penta-1,4-diene **6a** (101 MHz, chloroform-*d*).

9.3.2 1,4-diene 6b

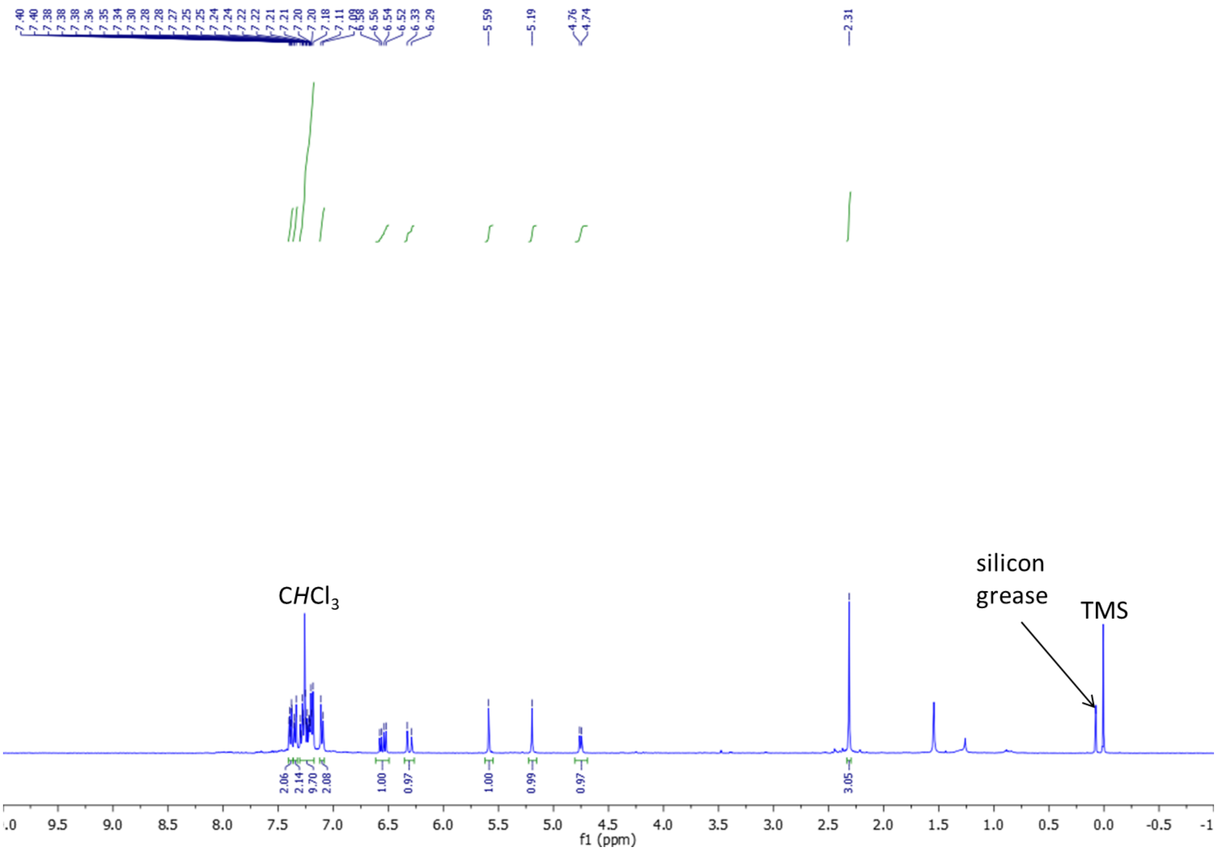

Figure SI 37: ^{1}H NMR spectrum of (*E*)-1,4-diphenyl-3-(*para*-methylphenyl)-penta-1,4-diene **6b** (400 MHz, chloroform-*d*).

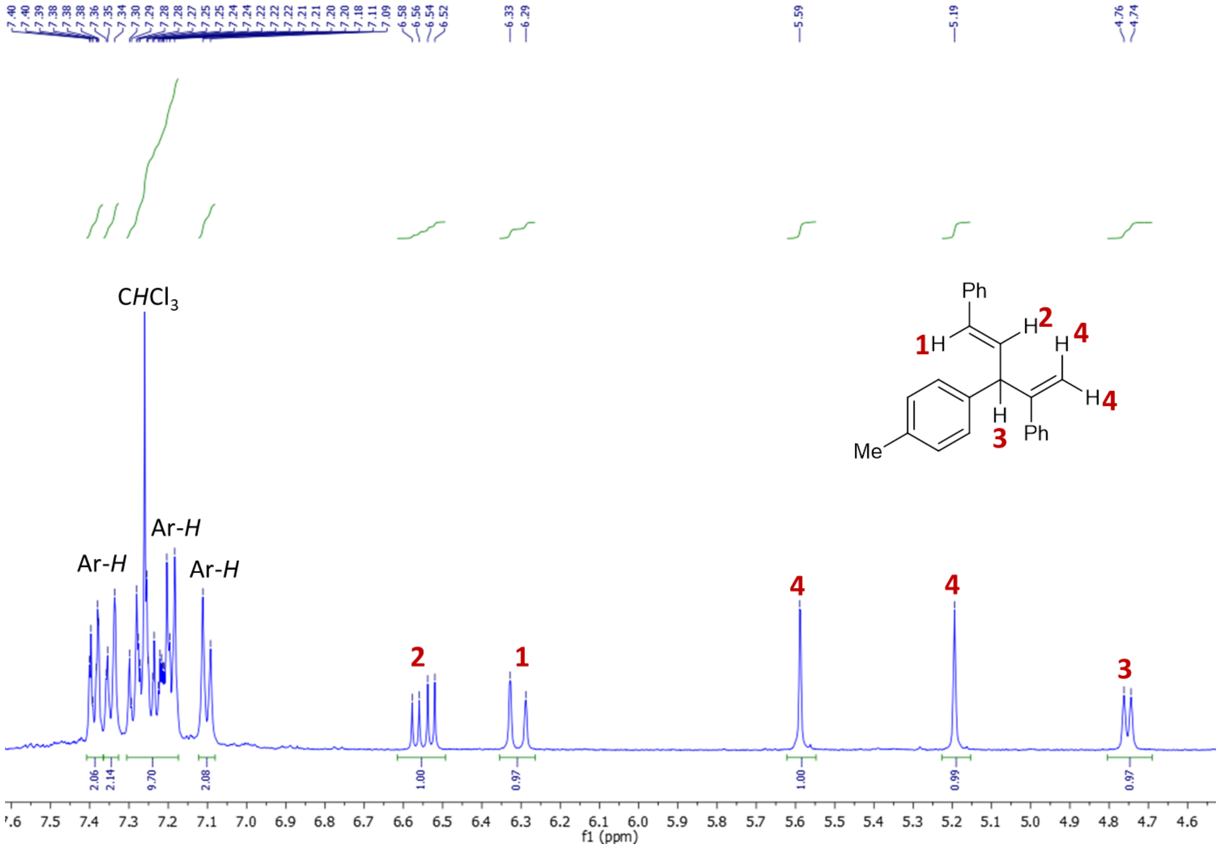

Figure SI 38: Excerpt of ^{1}H NMR spectrum of (*E*)-1,4-diphenyl-3-(*para*-methylphenyl)-penta-1,4-diene **6b** (400 MHz, chloroform-*d*).

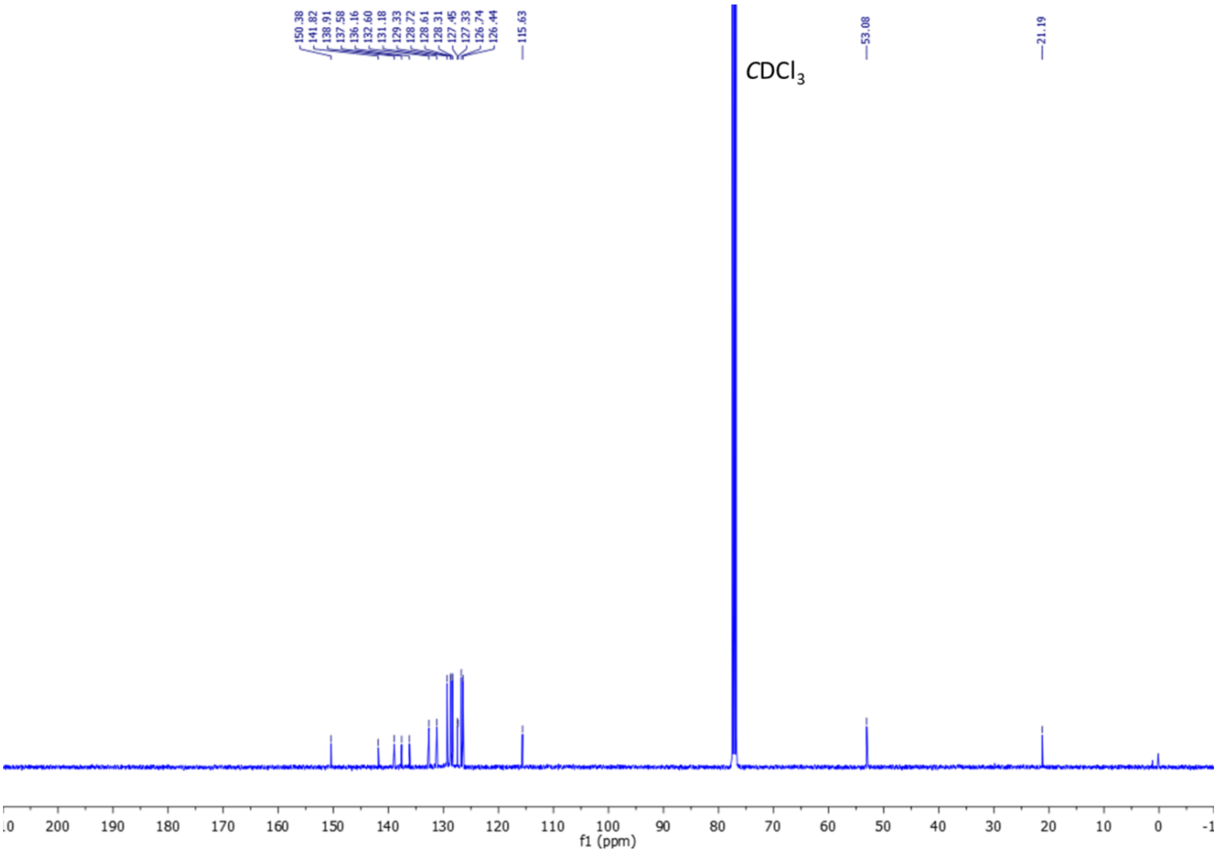

Figure SI 39: ^{13}C NMR spectrum of (*E*)-1,4-diphenyl-3-(*para*-methylphenyl)-penta-1,4-diene **6b** (101 MHz, chloroform-*d*).

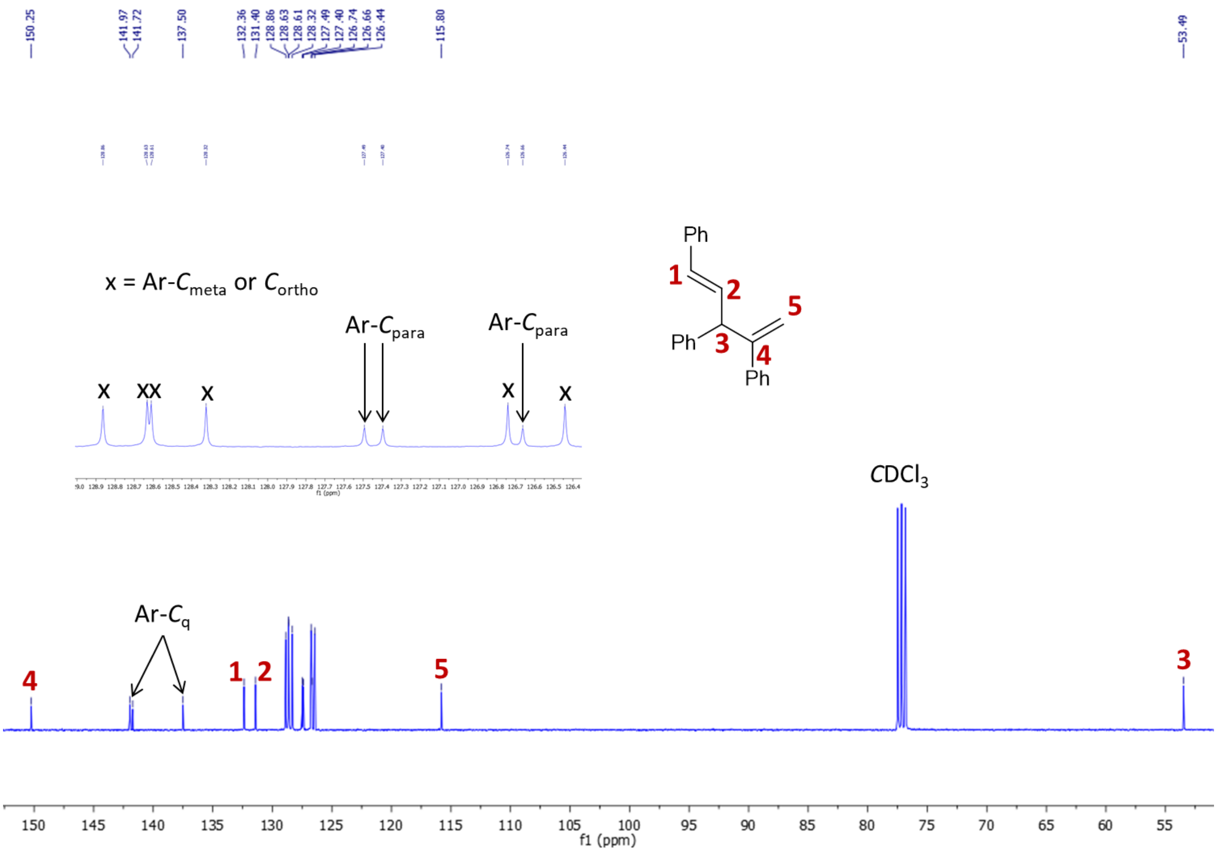

Figure SI 40: Excerpt of the ^{13}C NMR spectrum of (*E*)-1,4-diphenyl-3-(*para*-methylphenyl)-penta-1,4-diene **6b** (101 MHz, chloroform-*d*).

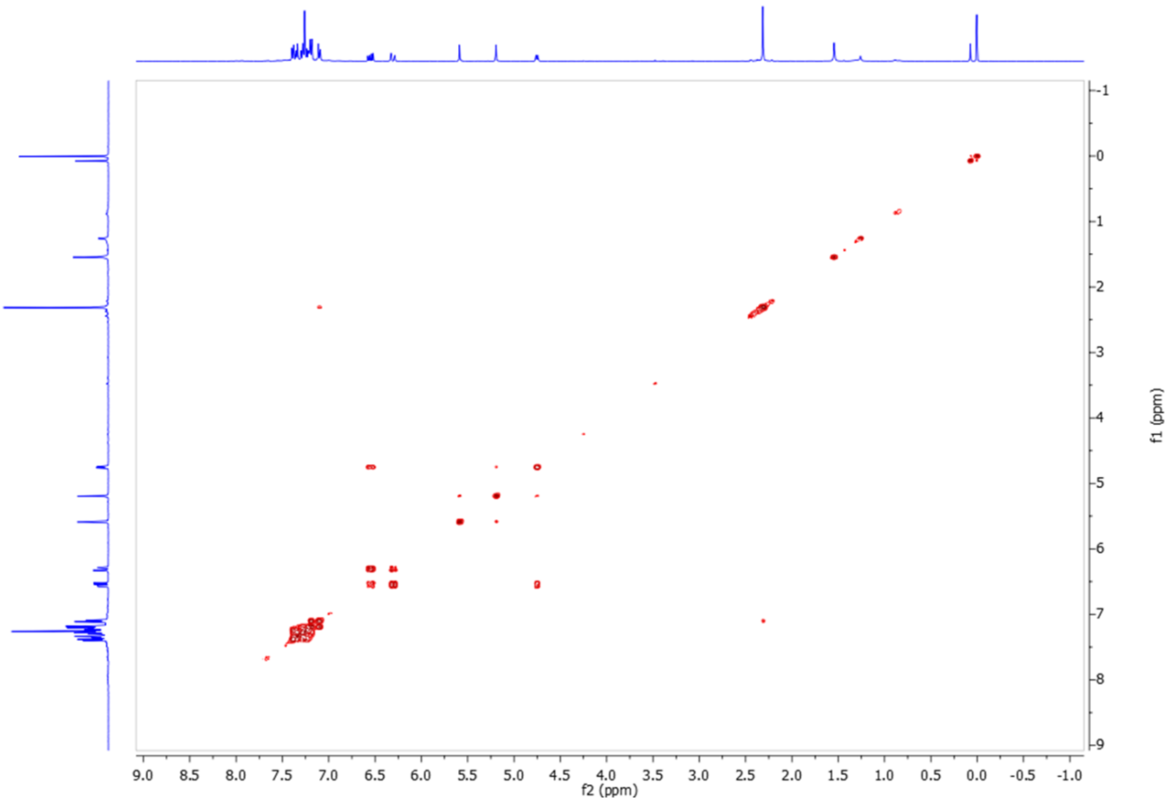

Figure SI 41: HH COSY NMR spectrum of (*E*)-1,4-diphenyl-3-(*para*-methylphenyl)-penta-1,4-diene **6b** (400 MHz, chloroform-*d*).

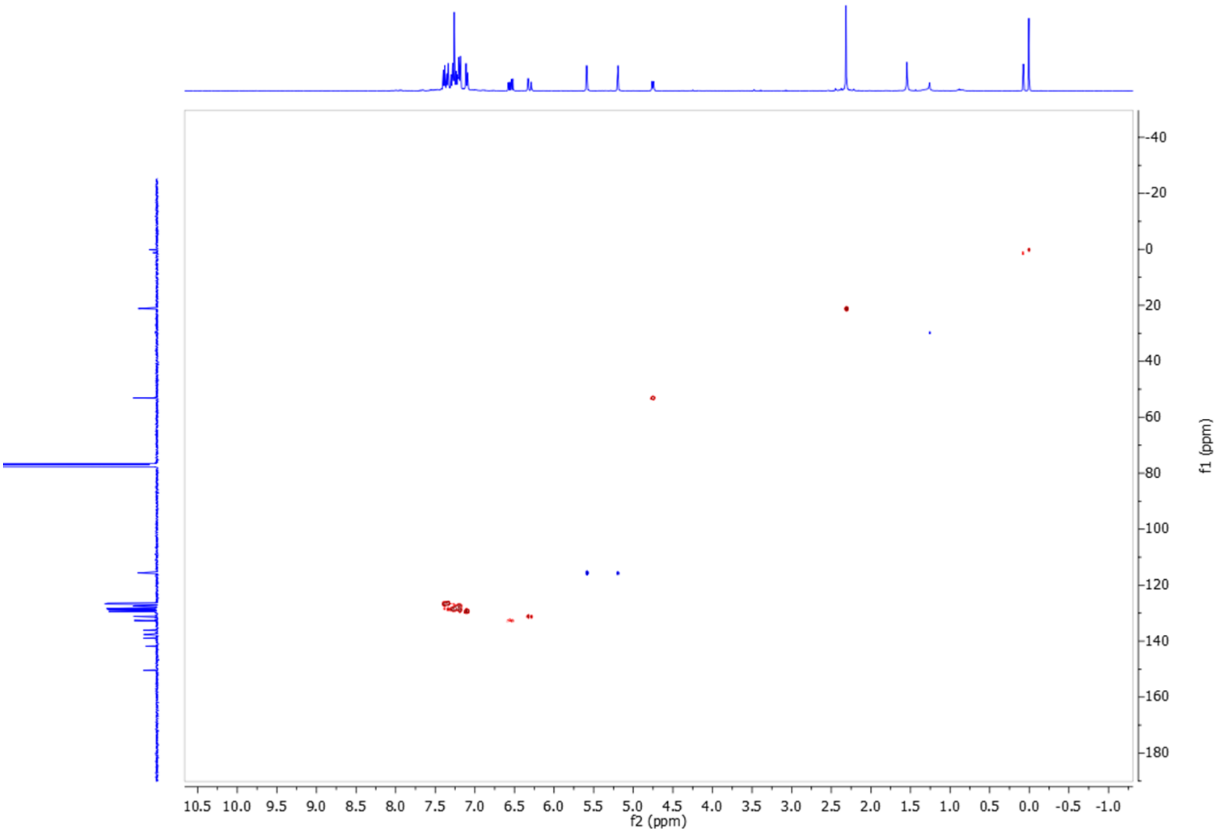

Figure SI 42: HSQC NMR spectrum of (*E*)-1,4-diphenyl-3-(*para*-methylphenyl)-penta-1,4-diene **6b** (101 MHz, chloroform-*d*).

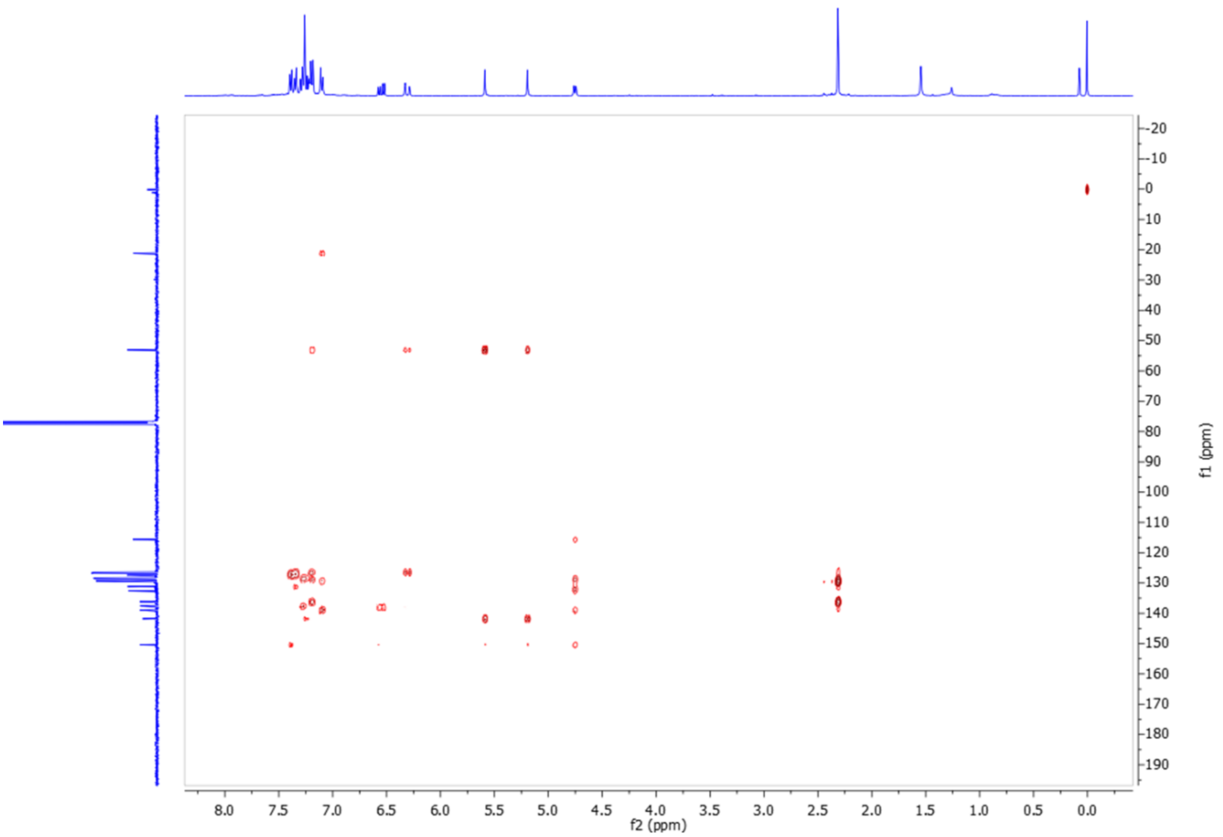

Figure SI 43: HMBC NMR spectrum of (*E*)-1,4-diphenyl-3-(*para*-methylphenyl)-penta-1,4-diene **6b** (101 MHz, chloroform-*d*).

9.3.3 1,4-diene 6c

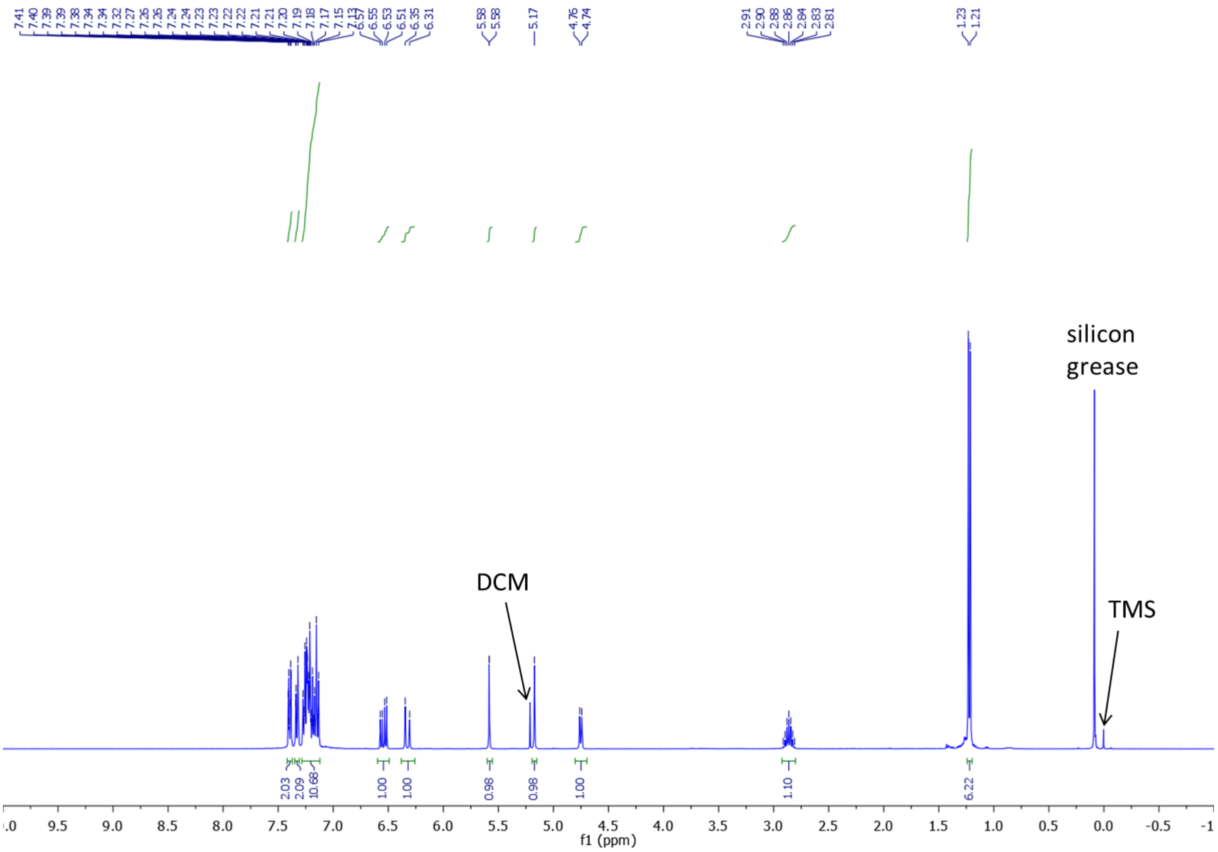

Figure SI 44: ^{1}H NMR spectrum of-(*E*)-1,4-diphenyl-3-(*para*-*iso*-propylphenyl)-penta-1,4-diene **6c** (400 MHz, chloroform-*d*).

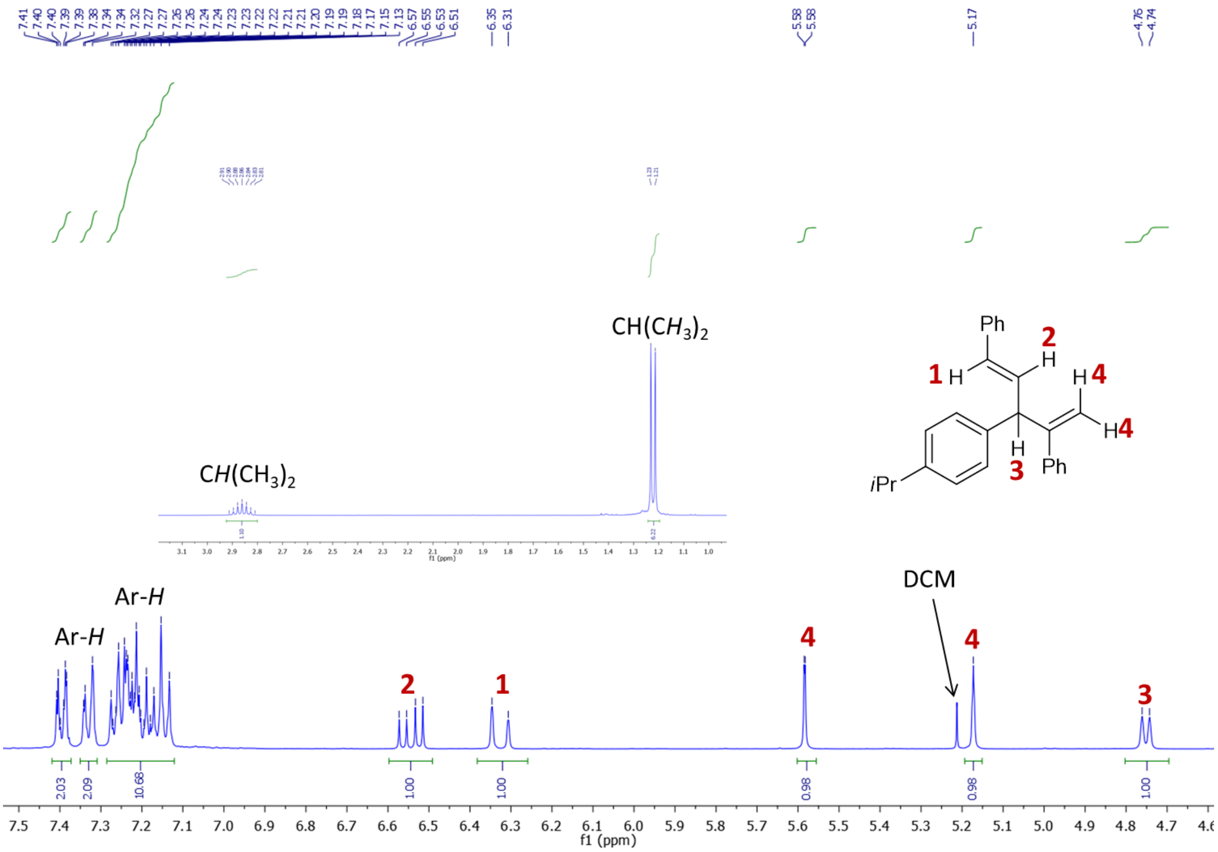

Figure SI 45: Excerpt of ^{1}H NMR spectrum of (*E*)-1,4-diphenyl-3-(*para*-*iso*-propylphenyl)-penta-1,4-diene **6c** (400 MHz, chloroform-*d*).

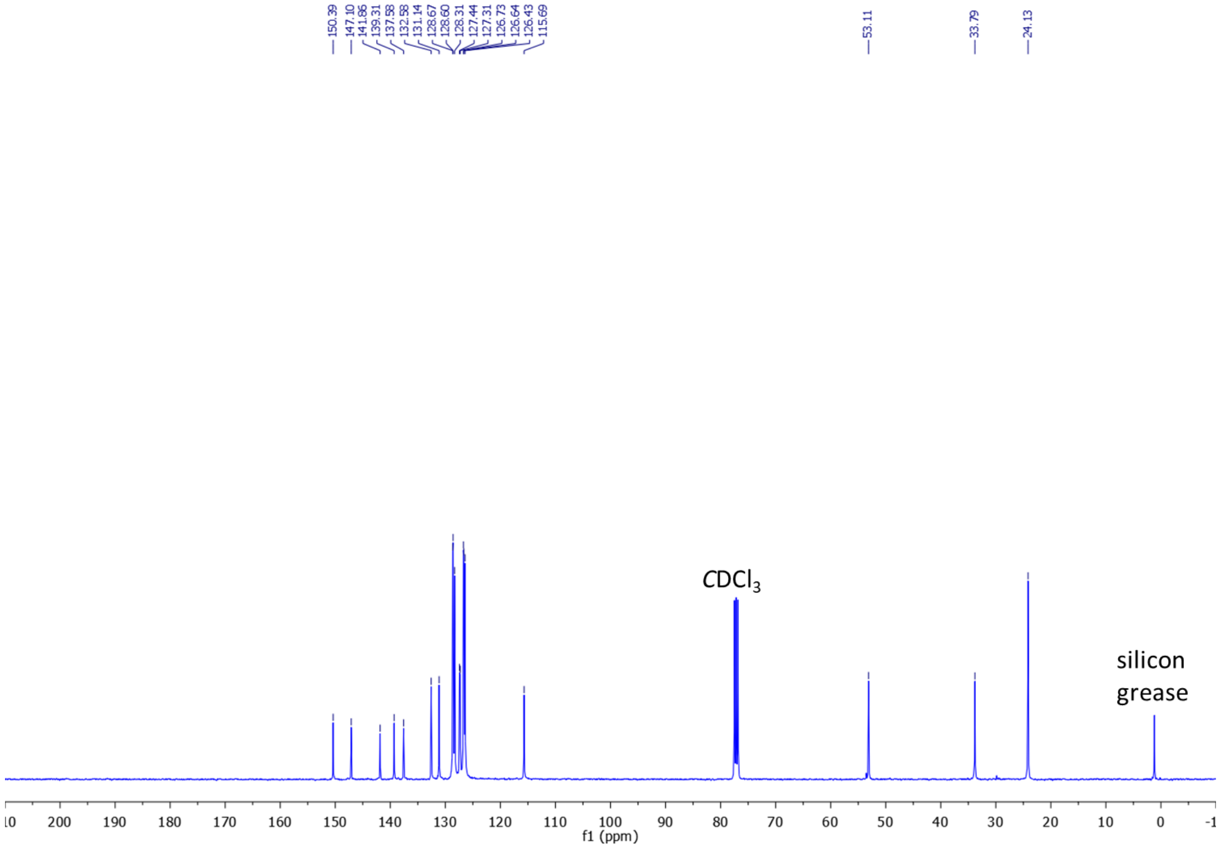

Figure SI 46: ^{13}C NMR spectrum of (*E*)-1,4-diphenyl-3-(*para*-*iso*-propylphenyl)-penta-1,4-diene **6c** (101 MHz, chloroform-*d*).

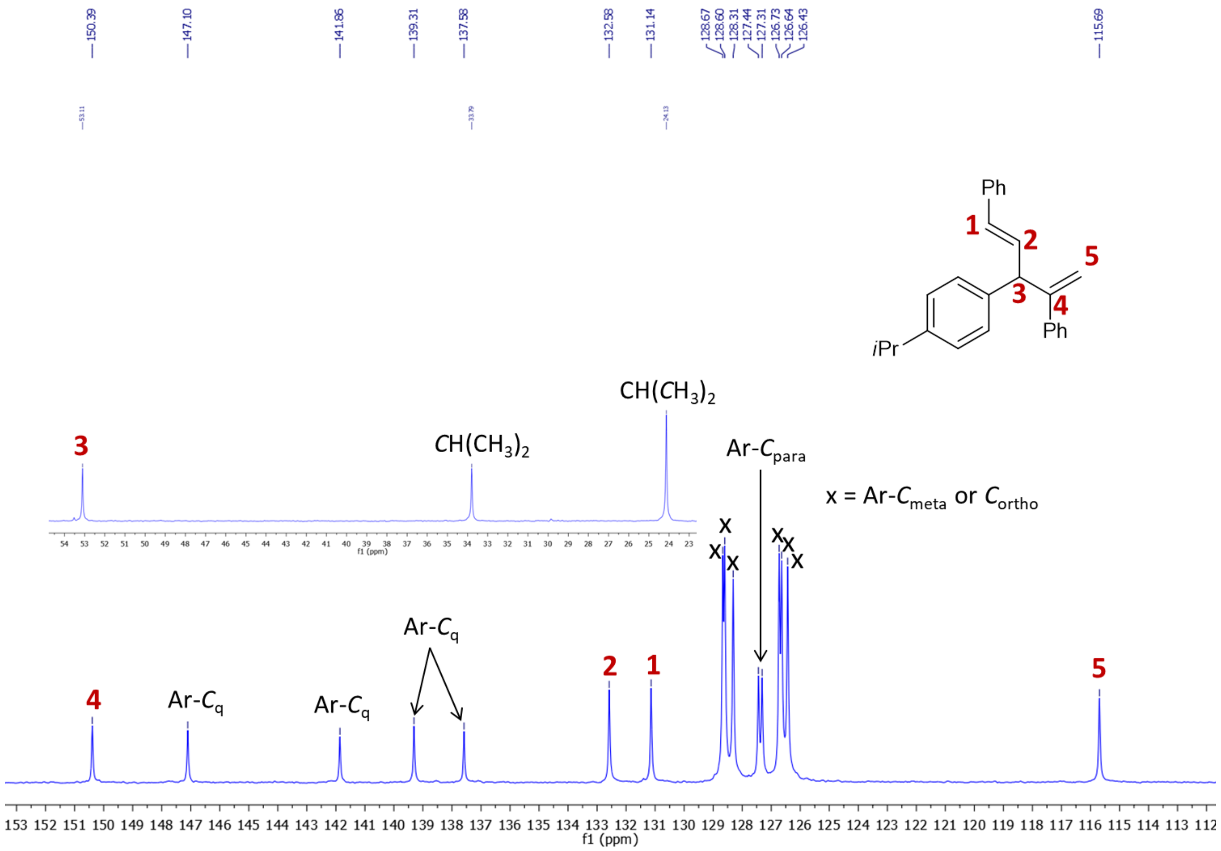

Figure SI 47: Excerpt of the ^{13}C NMR spectrum of (*E*)-1,4-diphenyl-3-(*para*-*iso*-propylphenyl)-penta-1,4-diene **6c** (101 MHz, chloroform-*d*).

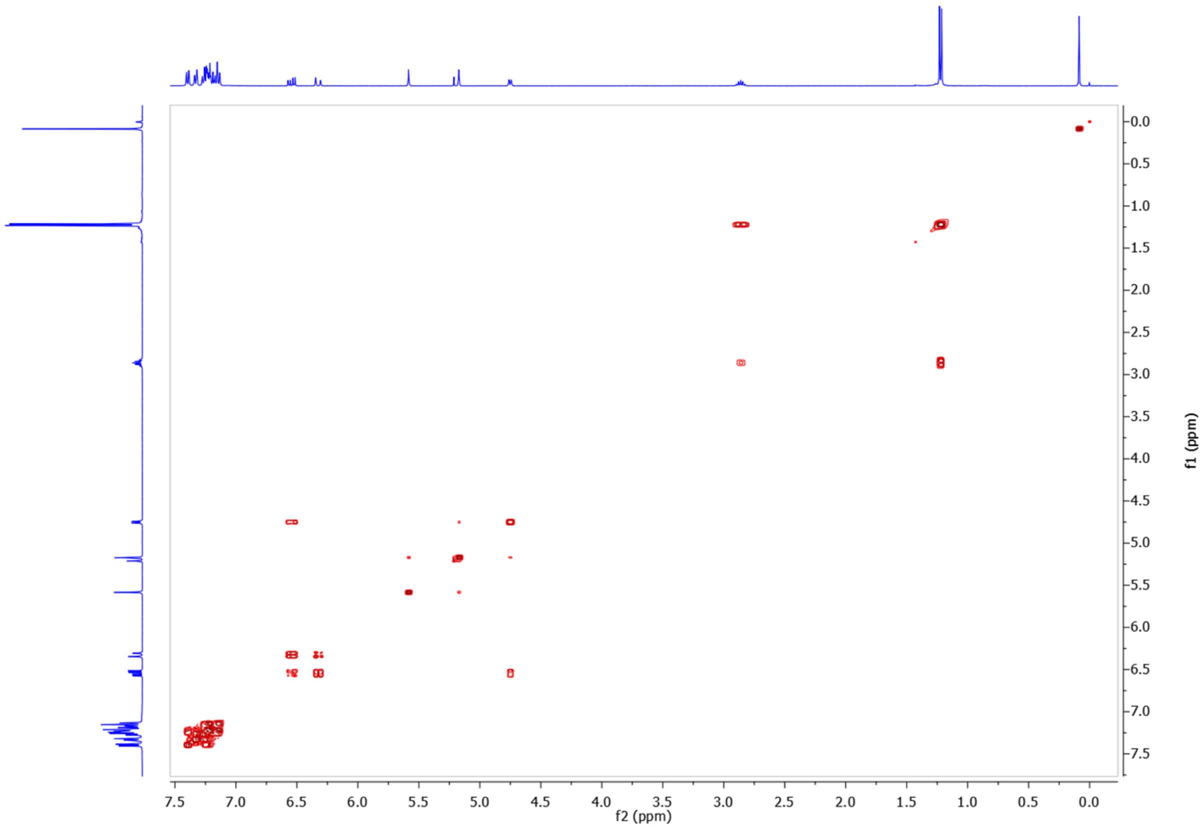

Figure SI 48: HH COSY NMR spectrum of (*E*)-1,4-diphenyl-3-(*para*-*iso*-propylphenyl)-penta-1,4-diene **6c** (400 MHz, chloroform-*d*).

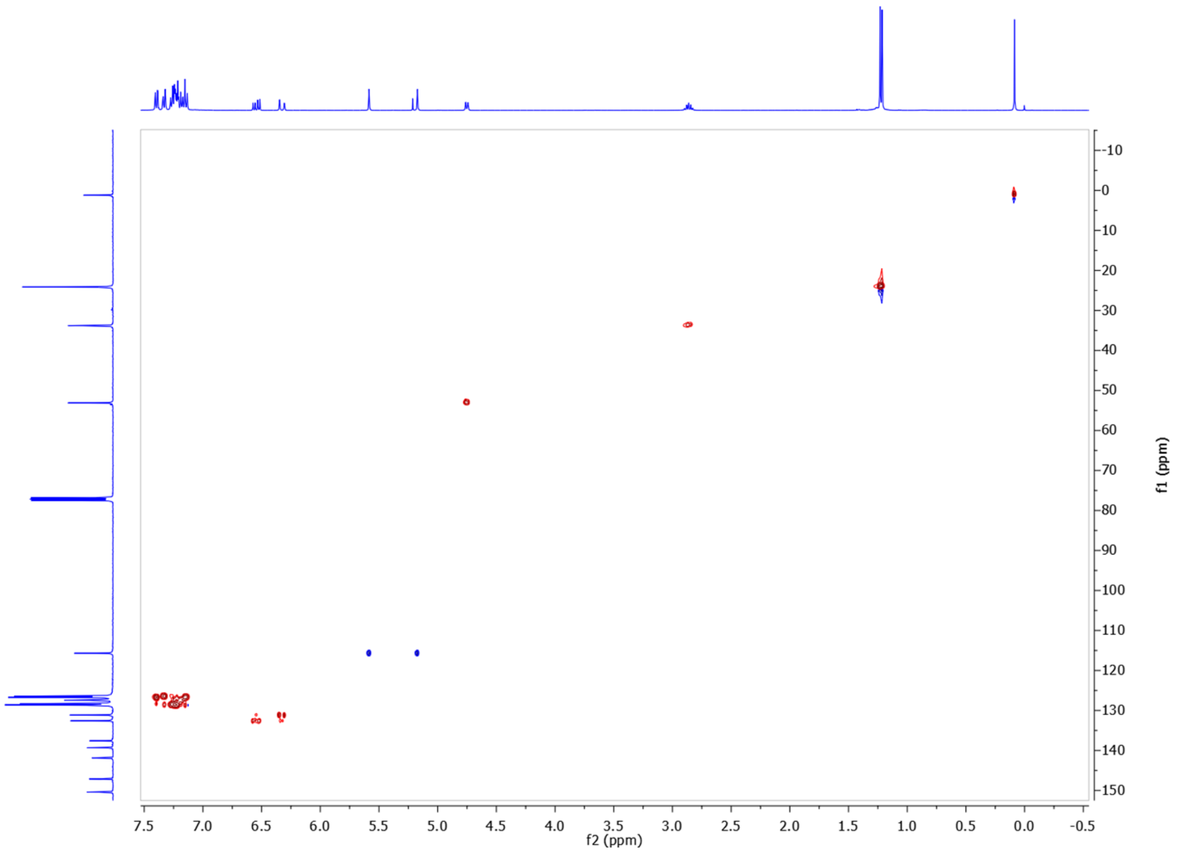

Figure SI 49: HSQC NMR spectrum of (*E*)-1,4-diphenyl-3-(*para*-*iso*-propylphenyl)-penta-1,4-diene **6c** (101 MHz, chloroform-*d*).

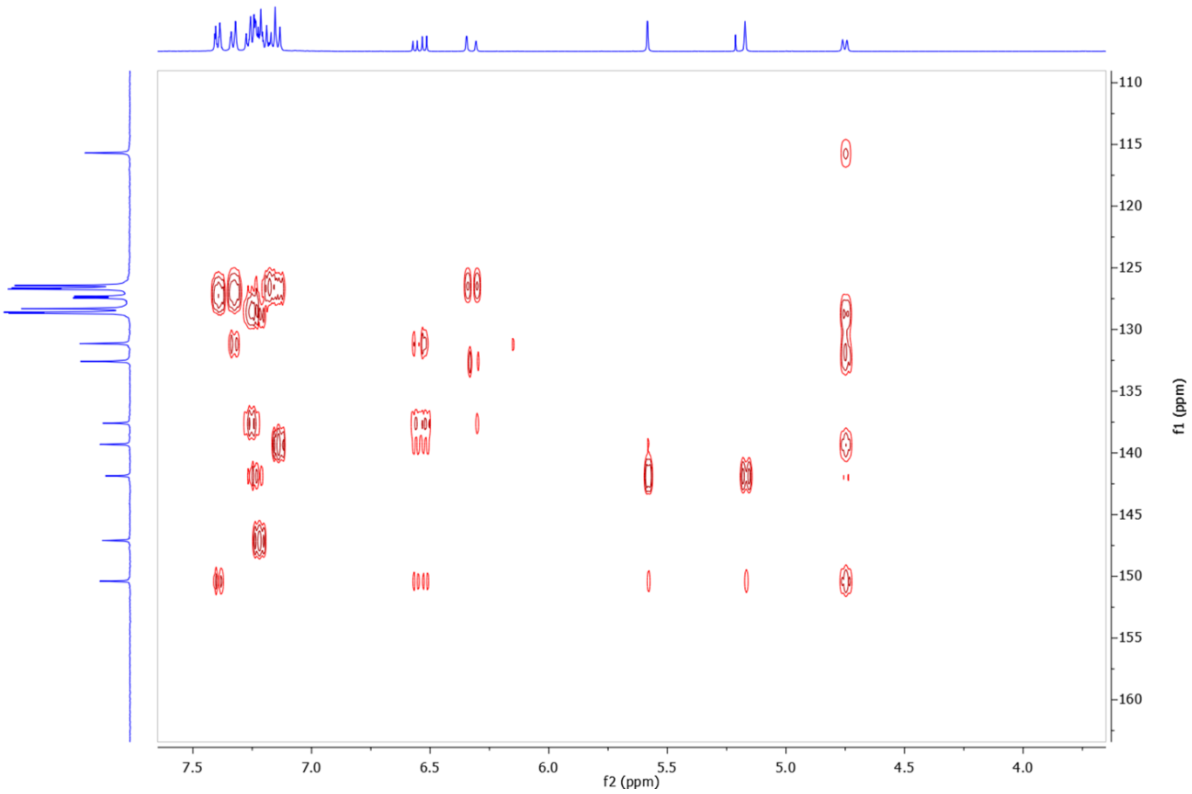

Figure SI 50: HMBC NMR spectrum of (*E*)-1,4-diphenyl-3-(*para*-*iso*-propylphenyl)-penta-1,4-diene **6c** (101 MHz, chloroform-*d*).

9.3.4 1,4-diene 6d

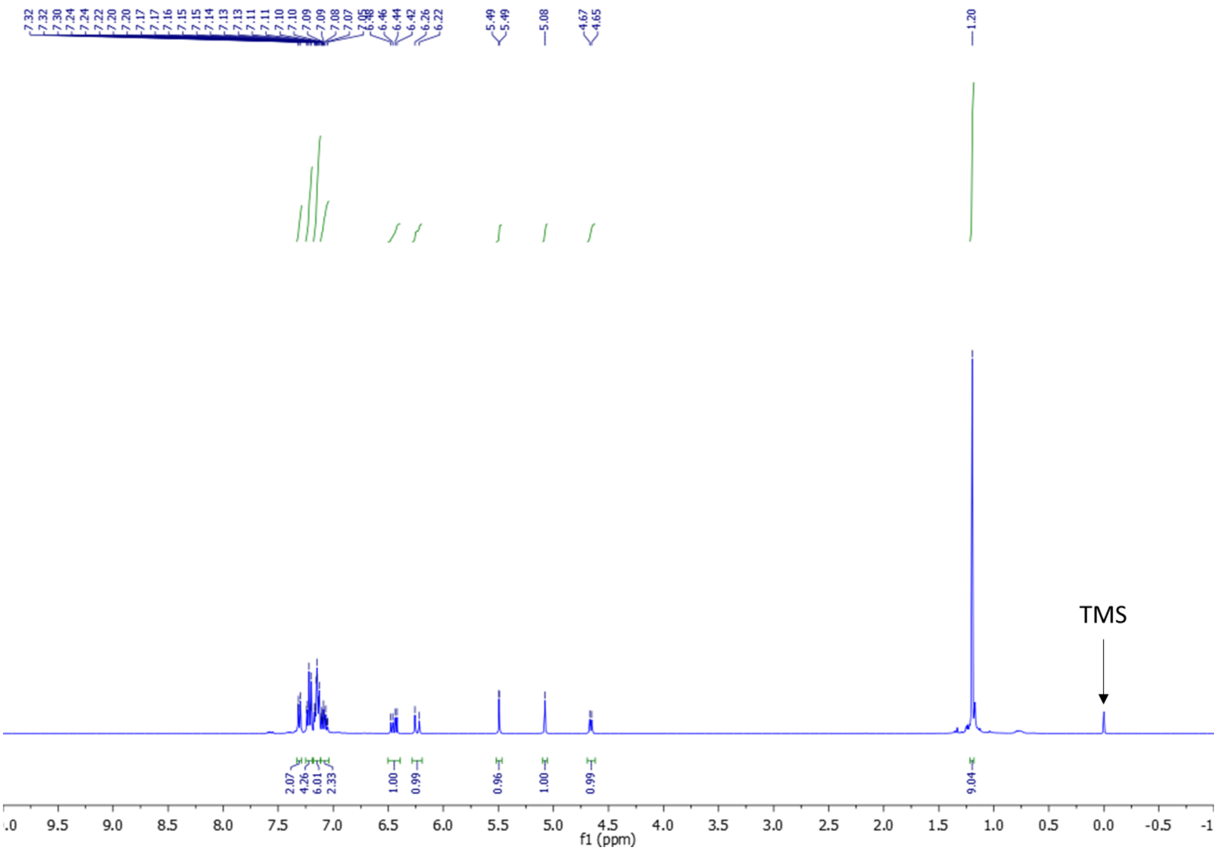

Figure SI 51: ^{1}H NMR spectrum of (*E*)-1,4-diphenyl-3-(*para*-*tert*-butylphenyl)-penta-1,4-diene **6d** (400 MHz, chloroform-*d*).

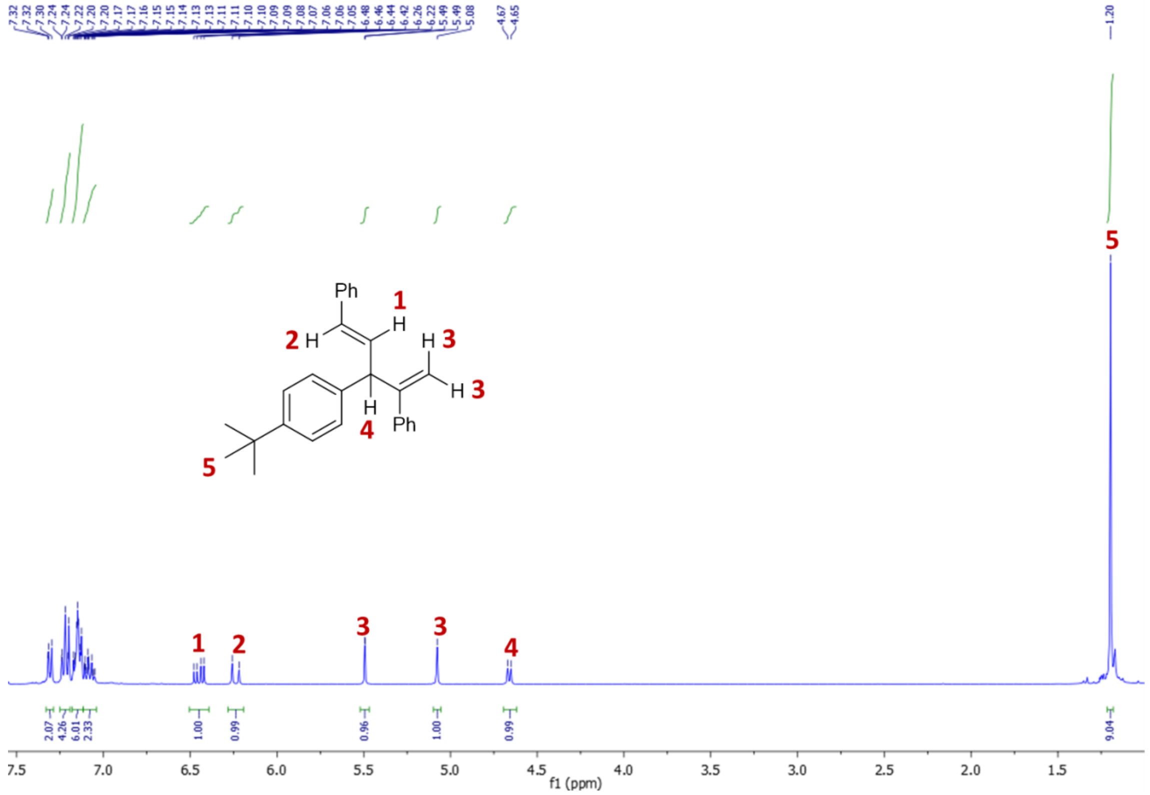

Figure SI 52: Excerpt of the ^{1}H NMR spectrum of (*E*)-1,4-diphenyl-3-(*para*-*tert*-butylphenyl)-penta-1,4-diene **6d** (400 MHz, chloroform-*d*).

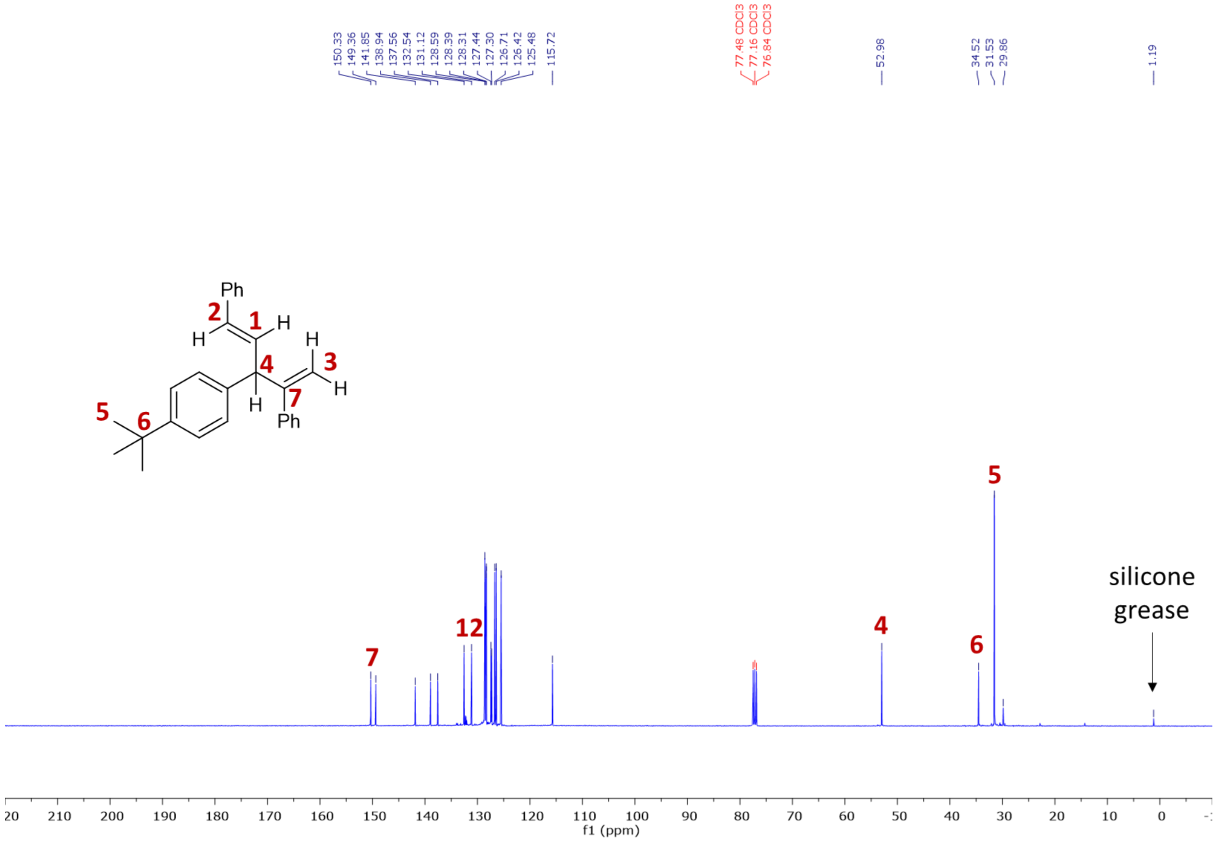

Figure SI 53: ^{13}C NMR spectrum of (*E*)-1,4-diphenyl-3-(*para*-*tert*-butylphenyl)-penta-1,4-diene **6d** (101 MHz, chloroform-*d*).

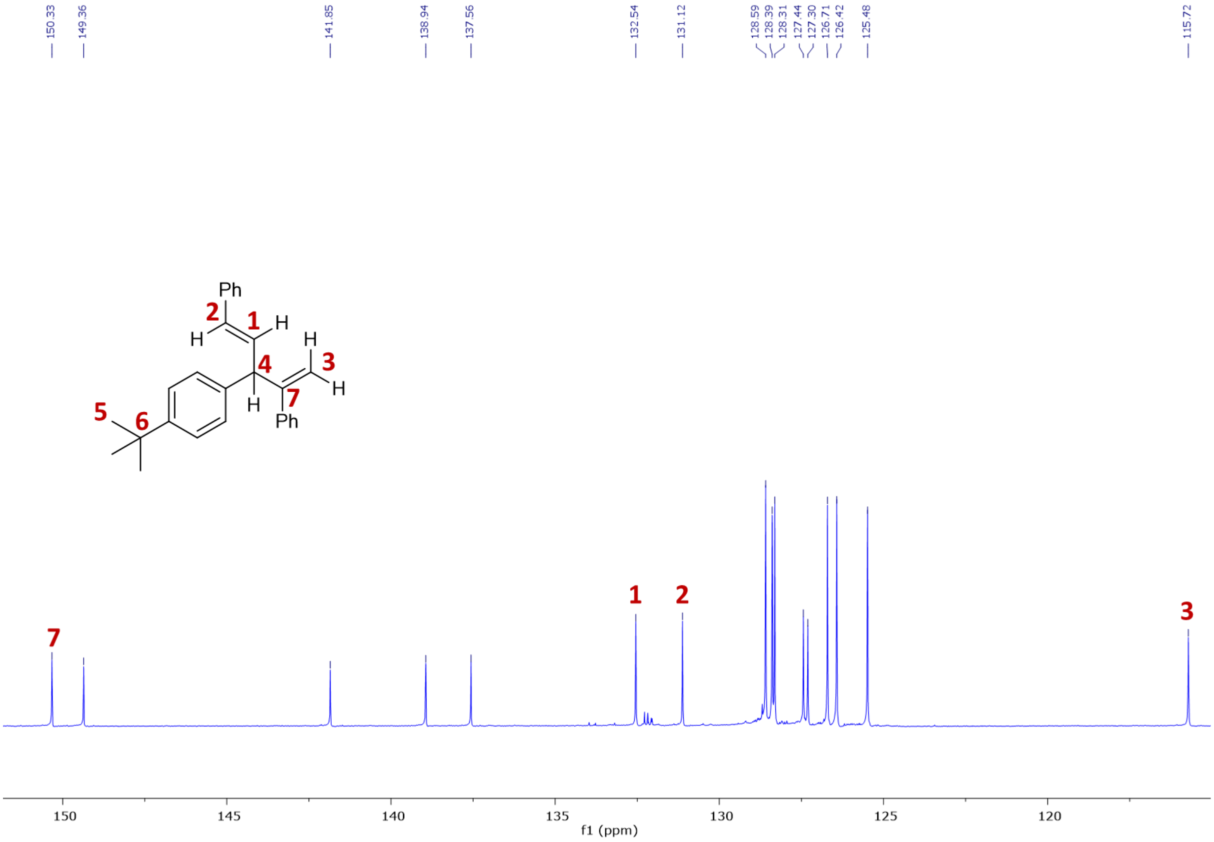

Figure SI 54: Excerpt of the ^{13}C NMR spectrum of (*E*)-1,4-diphenyl-3-(*para*-*tert*-butylphenyl)-penta-1,4-diene **6d** (101 MHz, chloroform-*d*).

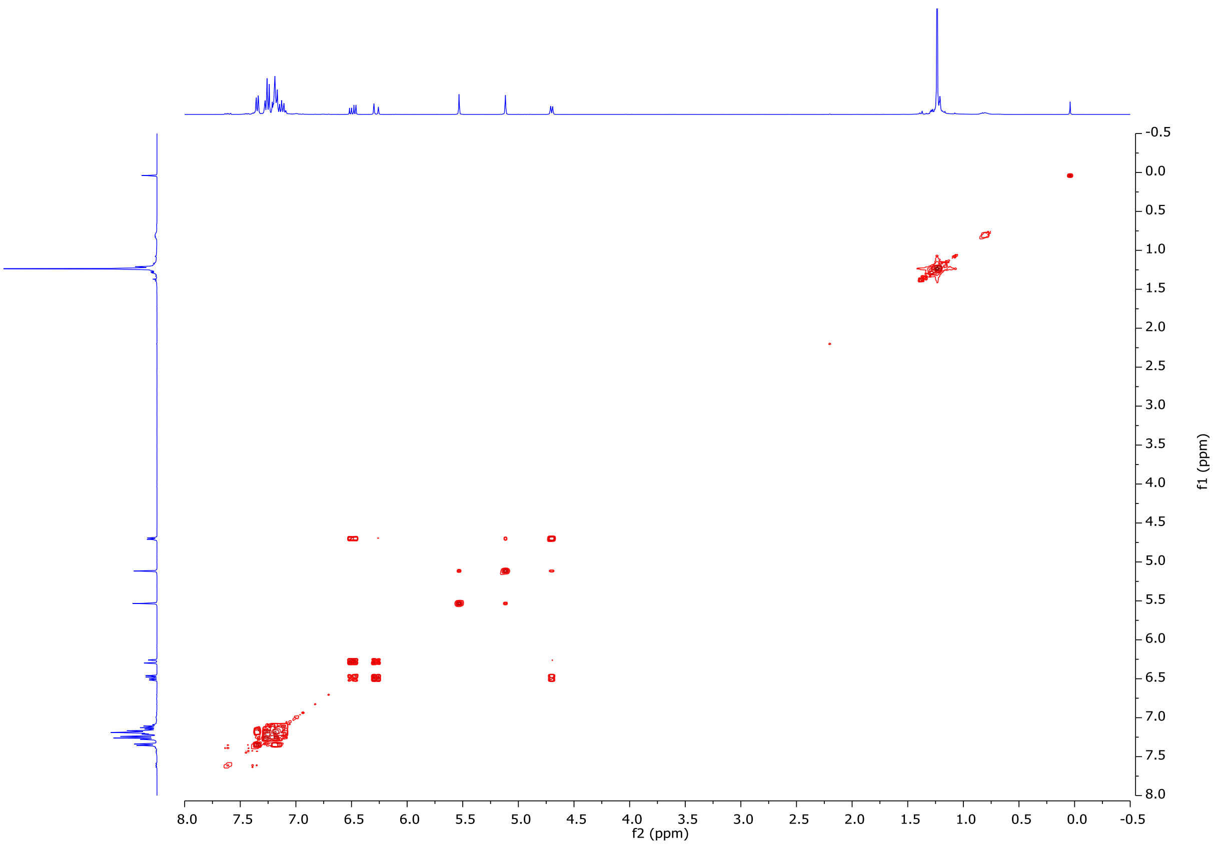

Figure SI 55: HH COSY NMR spectrum of (*E*)-1,4-diphenyl-3-(*para*-*tert*-butylphenyl)-penta-1,4-diene **6d** (400 MHz, chloroform-*d*).

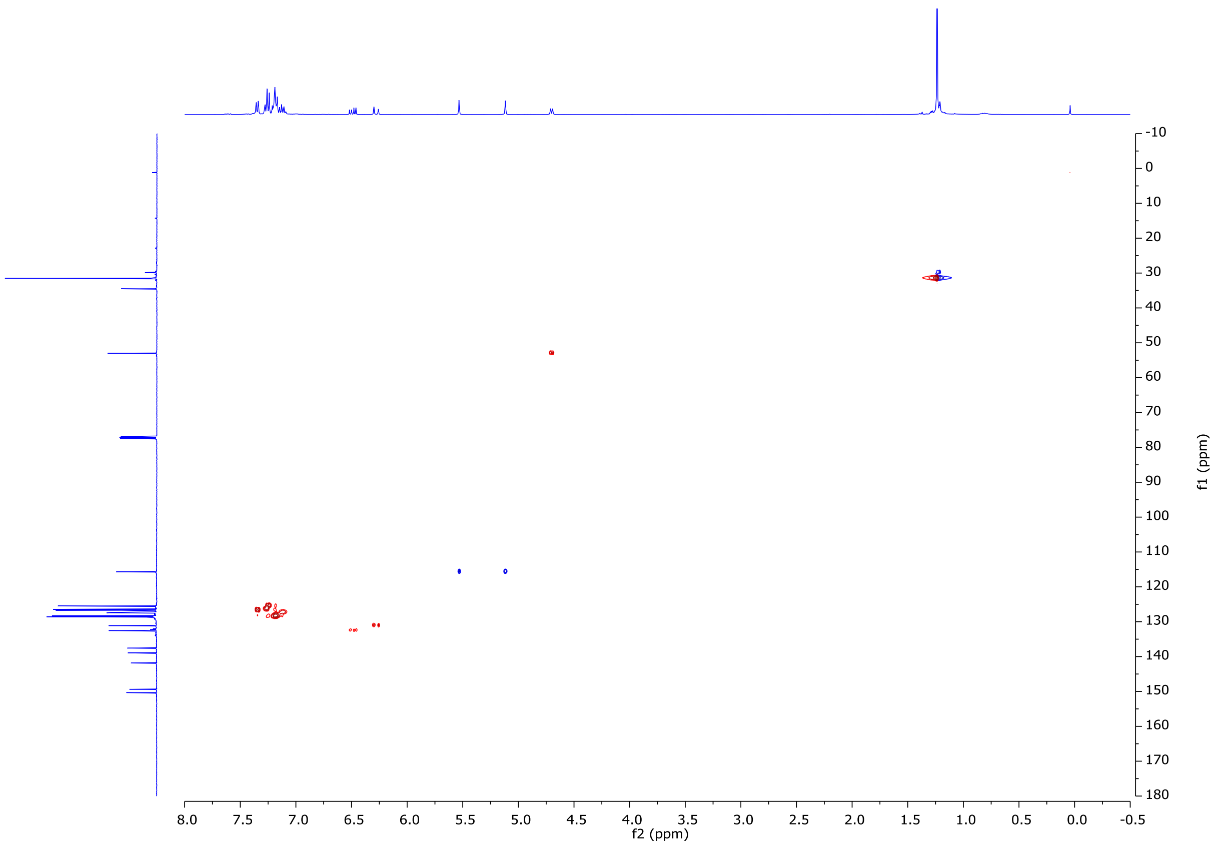

Figure SI 56: $^{1}H^{13}C$ HSQC NMR spectrum of (*E*)-1,4-diphenyl-3-(*para*-*tert*-butylphenyl)-penta-1,4-diene **6d** (400 MHz, chloroform-*d*).

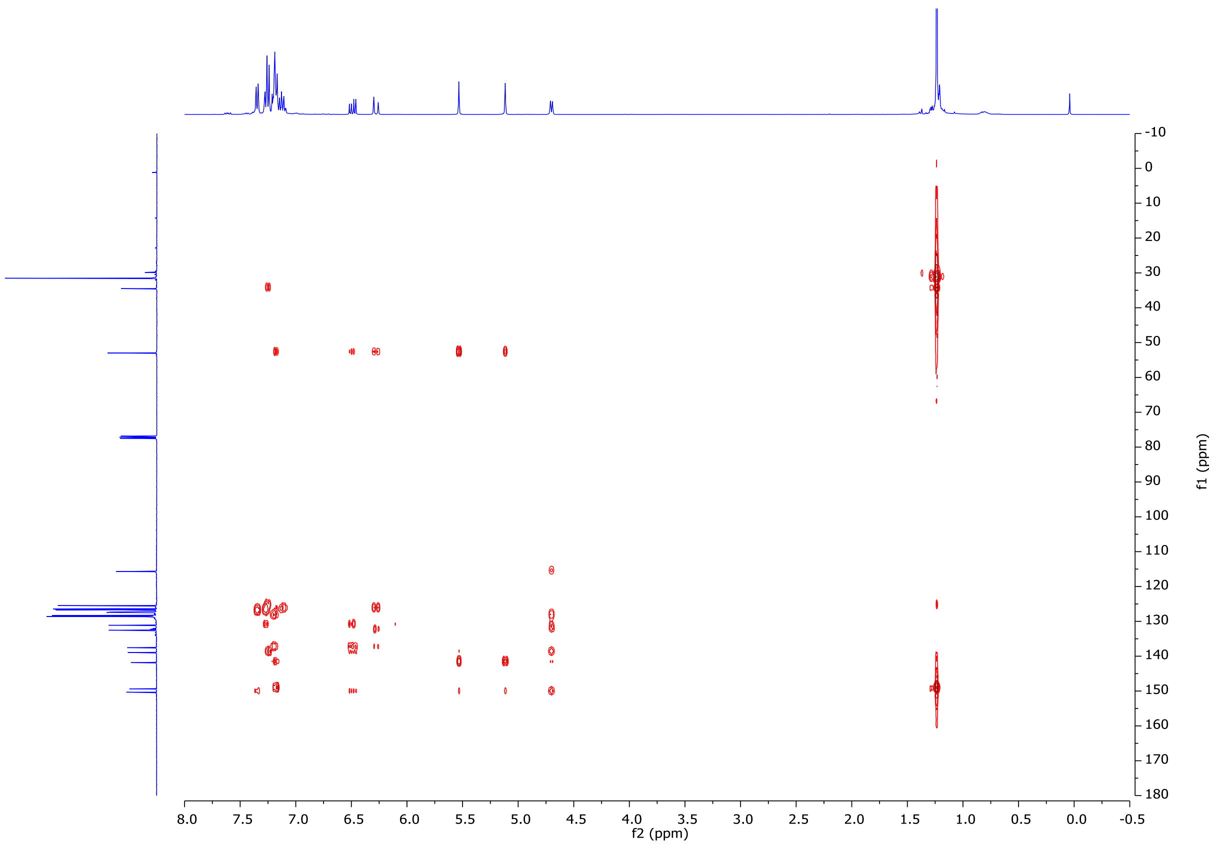

Figure SI 57: $^{1}H^{13}C$ HMBC NMR spectrum of (*E*)-1,4-diphenyl-3-(*para*-*tert*-butylphenyl)-penta-1,4-diene **6d** (400 MHz, chloroform-*d*).

9.3.5 1,4-diene 6e

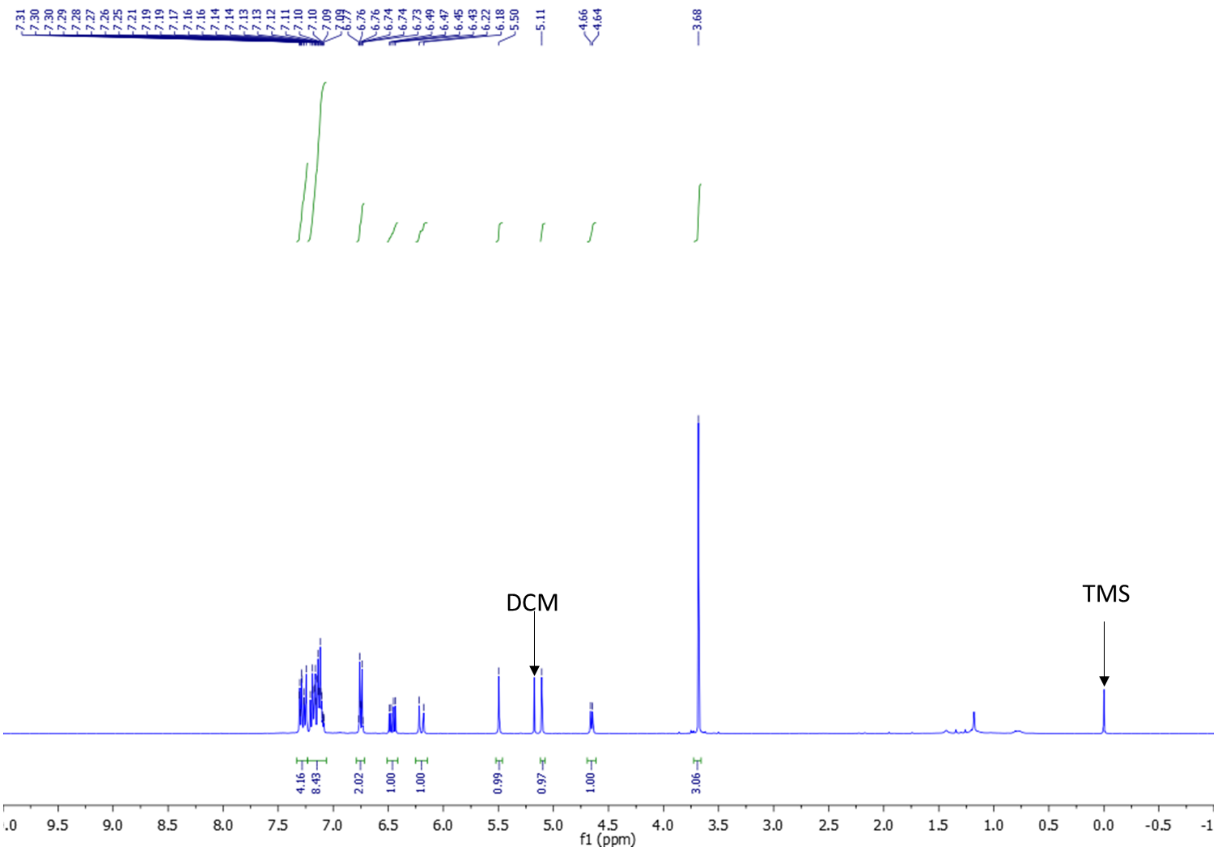

Figure SI 58: ^{1}H NMR spectrum of (*E*)-1,4-diphenyl-3-(*para*-methoxyphenyl)-penta-1,4-diene **6e** (400 MHz, chloroform-*d*).

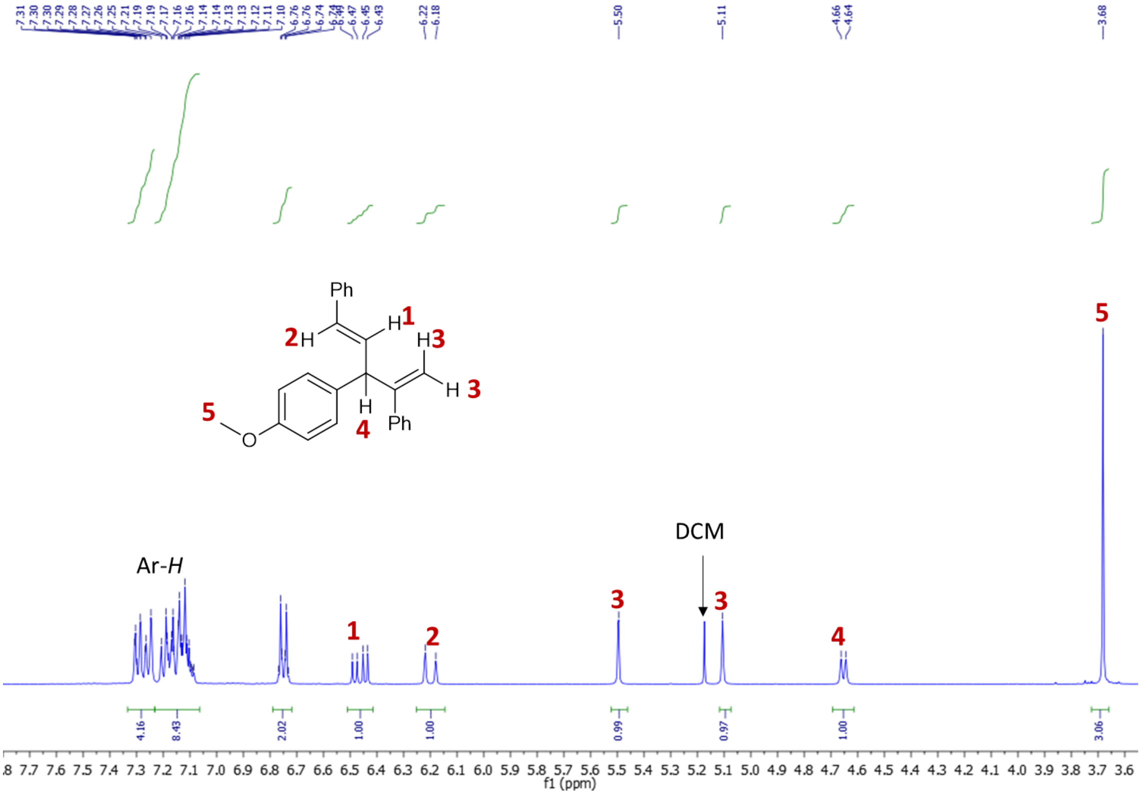

Figure SI 59: Excerpt of the ^1H NMR spectrum of (*E*)-1,4-diphenyl-3-(*para*-methoxyphenyl)-penta-1,4-diene **6e** (400 MHz, chloroform-*d*).

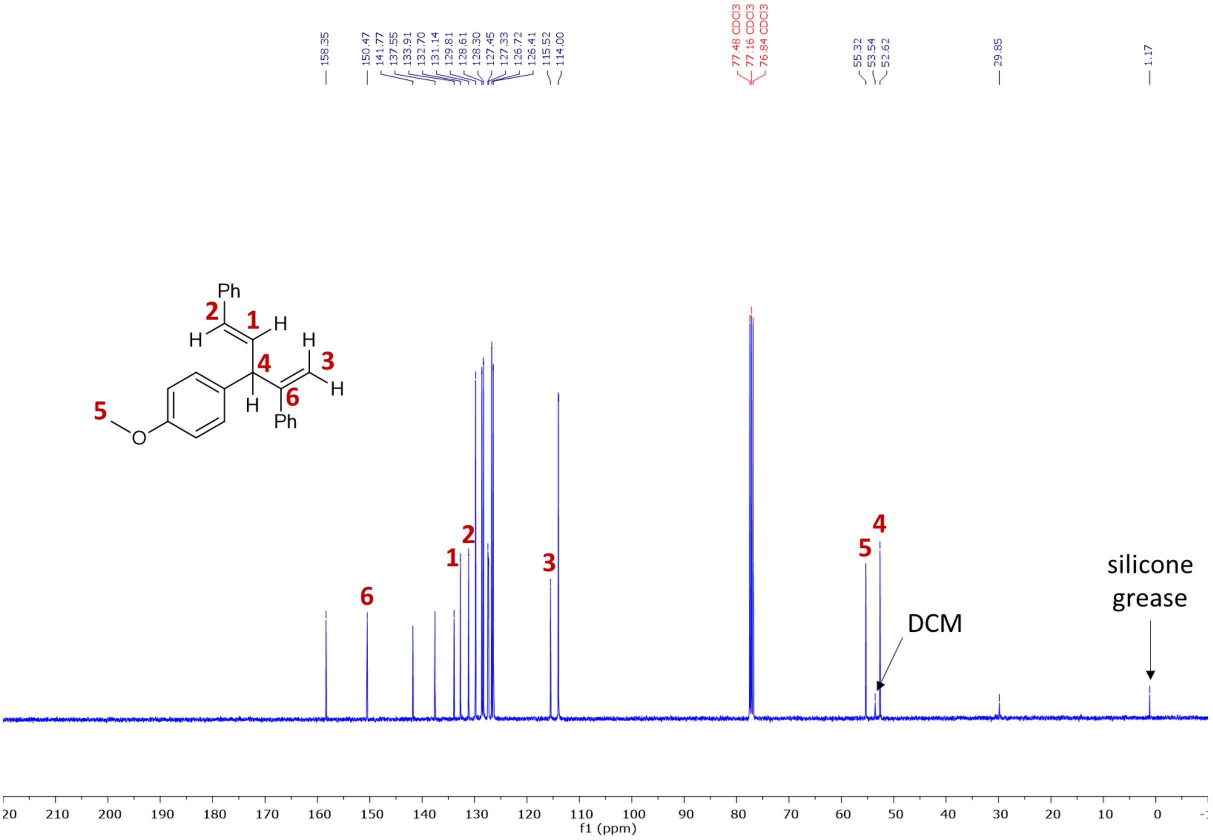

Figure SI 60: ^{13}C NMR spectrum of (*E*)-1,4-diphenyl-3-(*para*-methoxyphenyl)-penta-1,4-diene **6e** (101 MHz, chloroform-*d*).

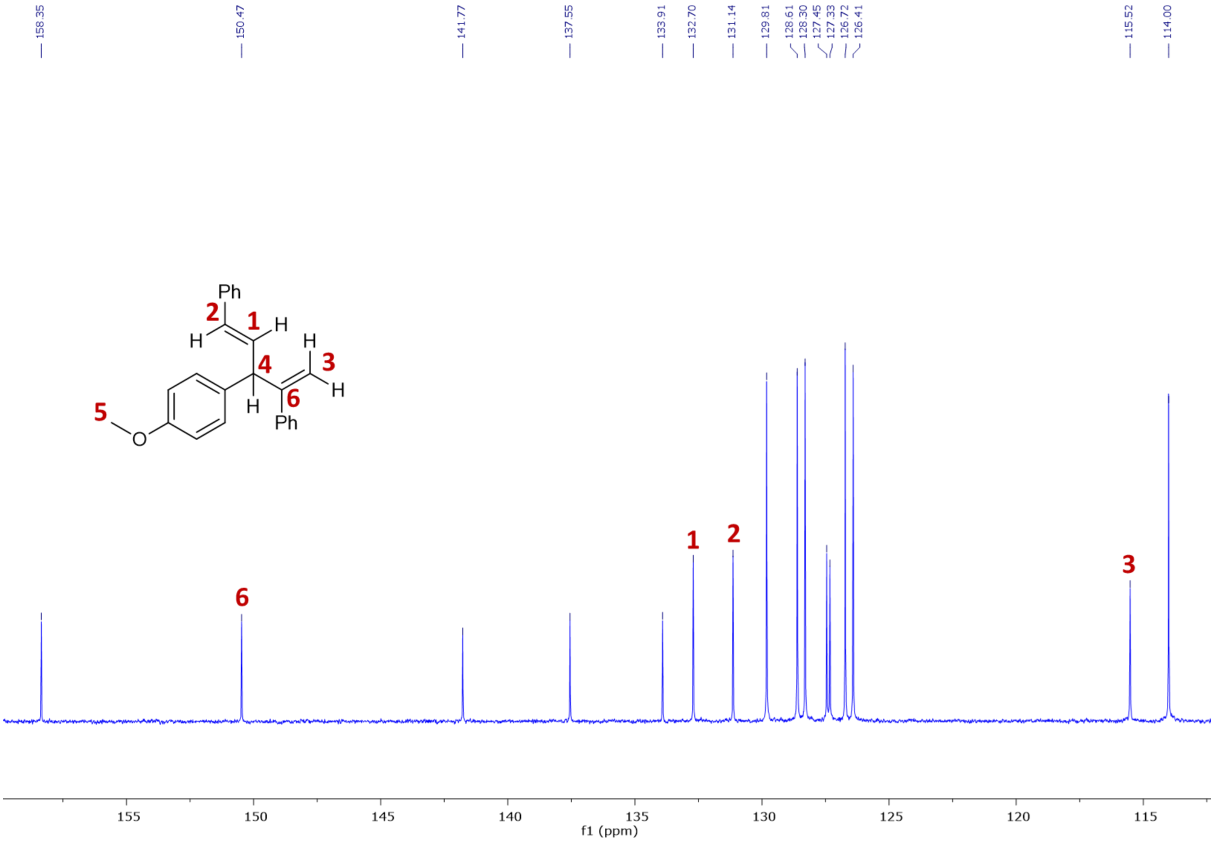

Figure SI 61: Excerpt of the ^{13}C NMR spectrum of (*E*)-1,4-diphenyl-3-(*para*-methoxyphenyl)-penta-1,4-diene **6e** (101 MHz, chloroform-*d*).

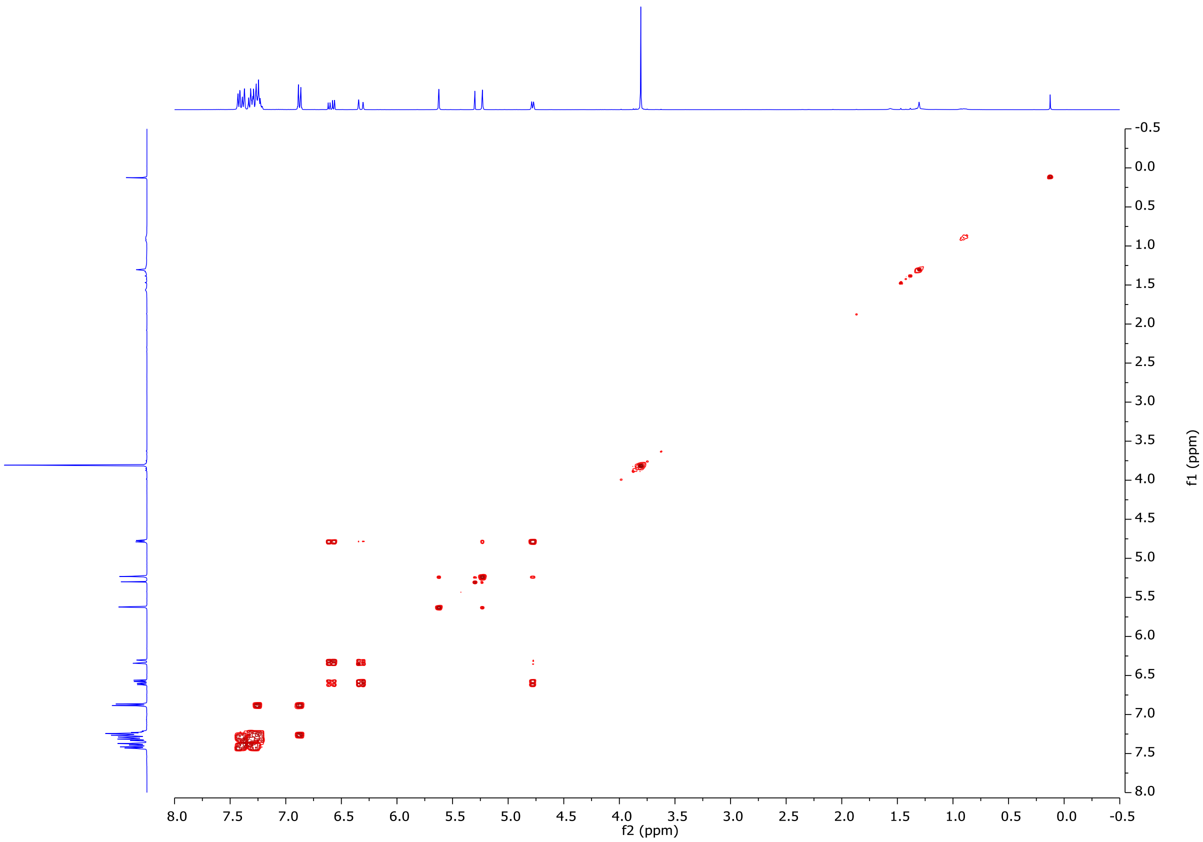

Figure SI 62: HH COSY NMR spectrum of (*E*)-1,4-diphenyl-3-(*para*-methoxyphenyl)-penta-1,4-diene **6e** (400 MHz, chloroform-*d*).

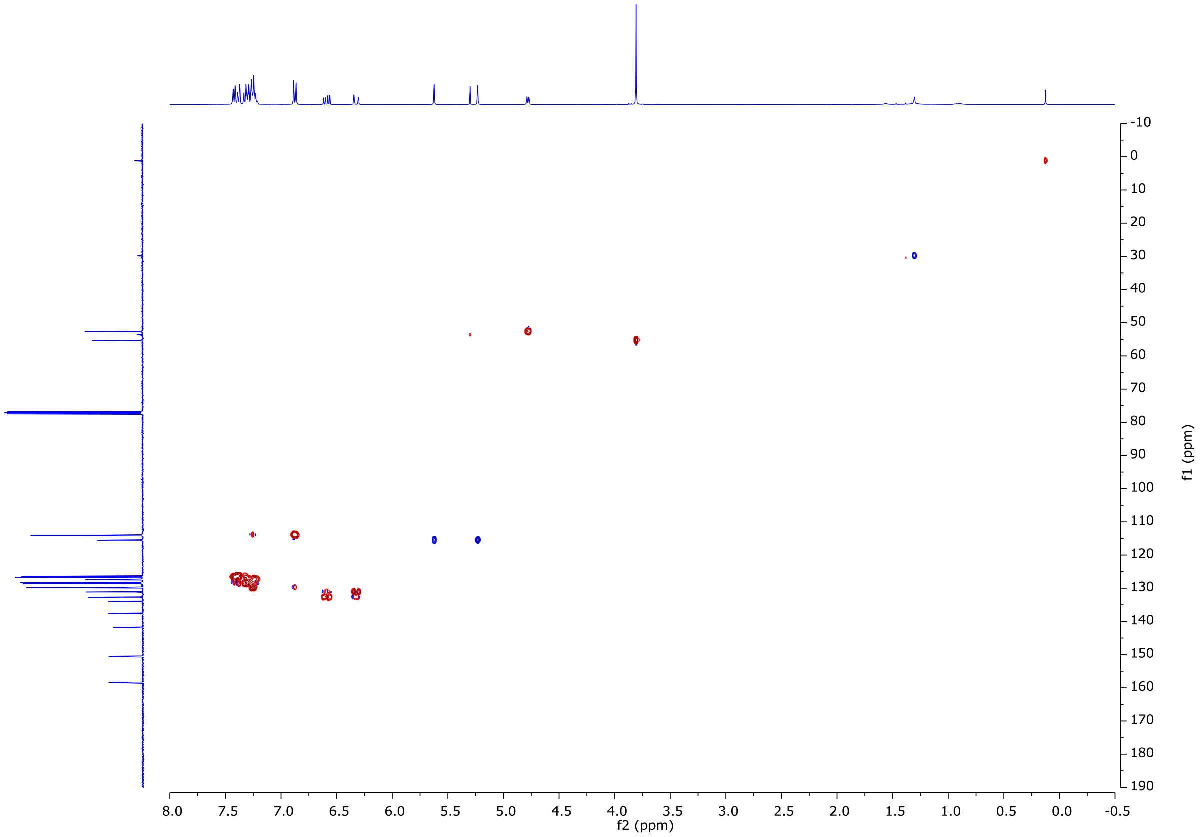

Figure SI 63: $^{1}H^{13}C$ HSQC NMR spectrum of (*E*)-1,4-diphenyl-3-(*para*-methoxyphenyl)-penta-1,4-diene **6e** (101 MHz, chloroform-*d*).

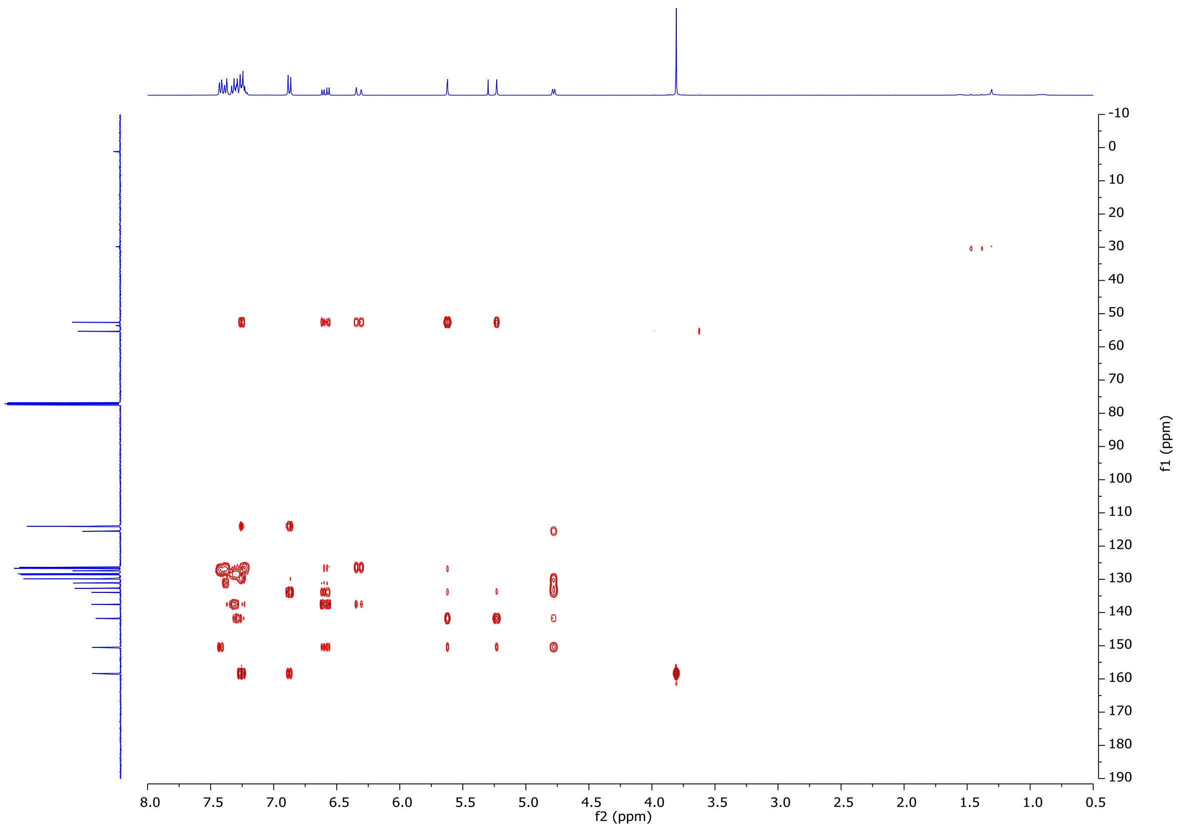

Figure SI 64: $^{1}H^{13}C$ HMBC NMR spectrum of (*E*)-1,4-diphenyl-3-(*para*-methoxyphenyl)-penta-1,4-diene **6e** (101 MHz, chloroform-*d*).

9.3.6 1,4-diene 6f

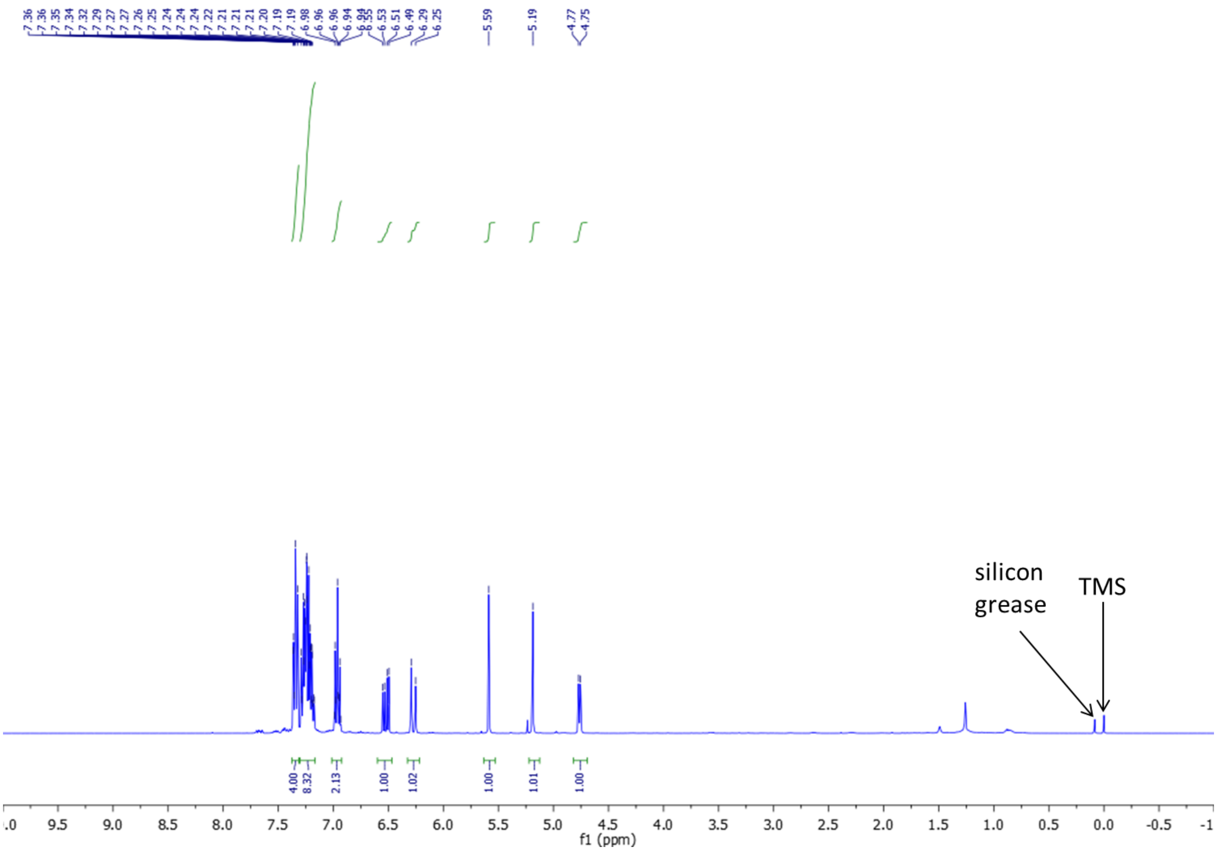

Figure SI 65: ^{1}H NMR spectrum of (*E*)-1,4-diphenyl-2-(*para*-fluorophenyl)-penta-1,4-diene **6f** (400 MHz, chloroform-*d*).

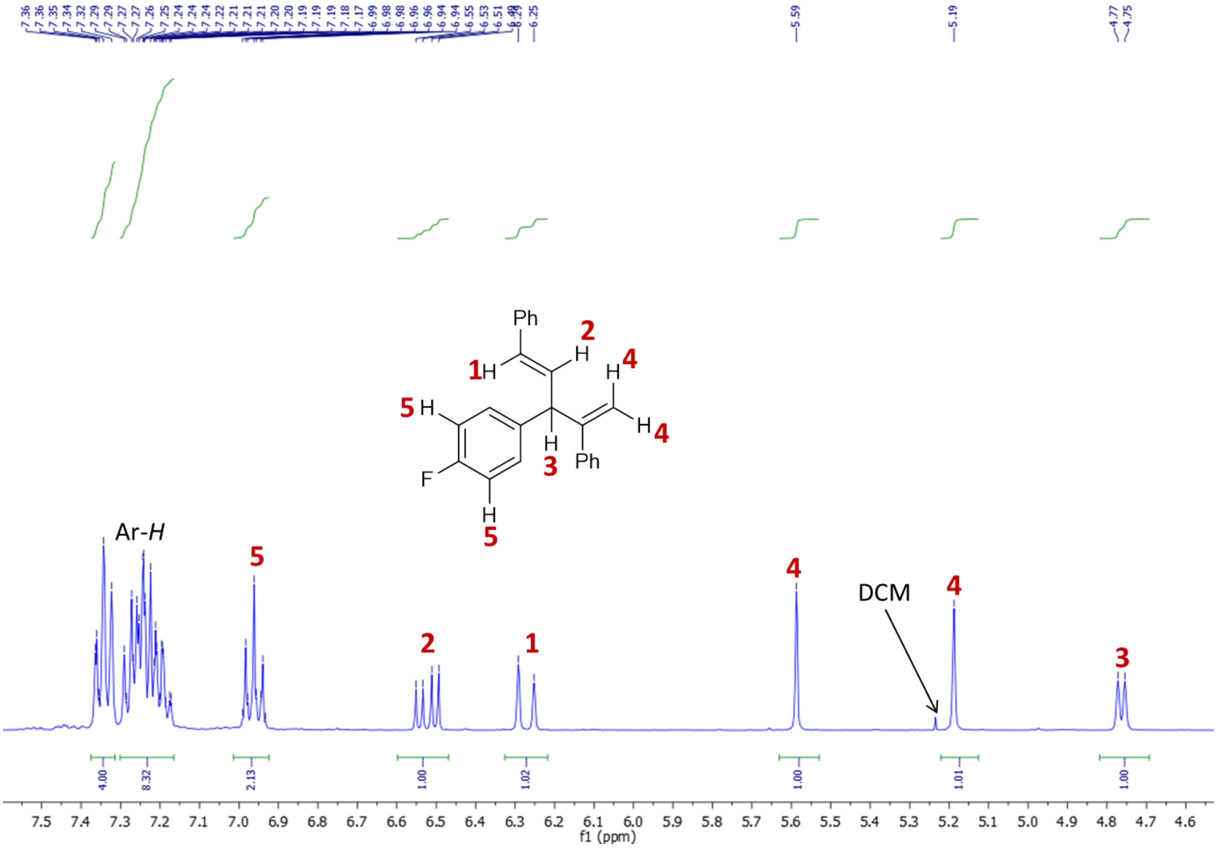

Figure SI 66: Excerpt of ^{1}H NMR spectrum of (*E*)-1,4-diphenyl-2-(*para*-fluorophenyl)-penta-1,4-diene **6f** (400 MHz, chloroform-*d*).

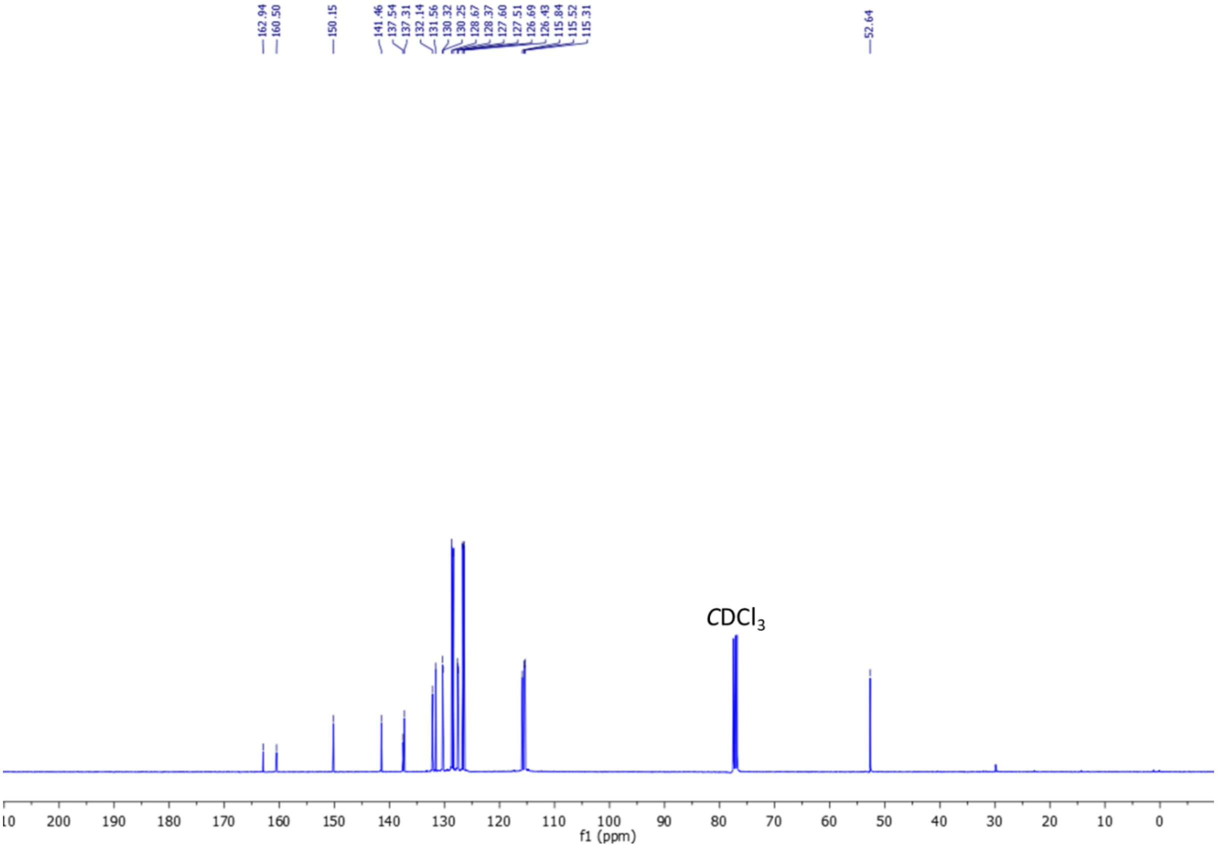

Figure SI 67: ^{13}C NMR spectrum of (*E*)-1,4-diphenyl-2-(*para*-fluorophenyl)-penta-1,4-diene **6f** (101 MHz, chloroform-*d*).

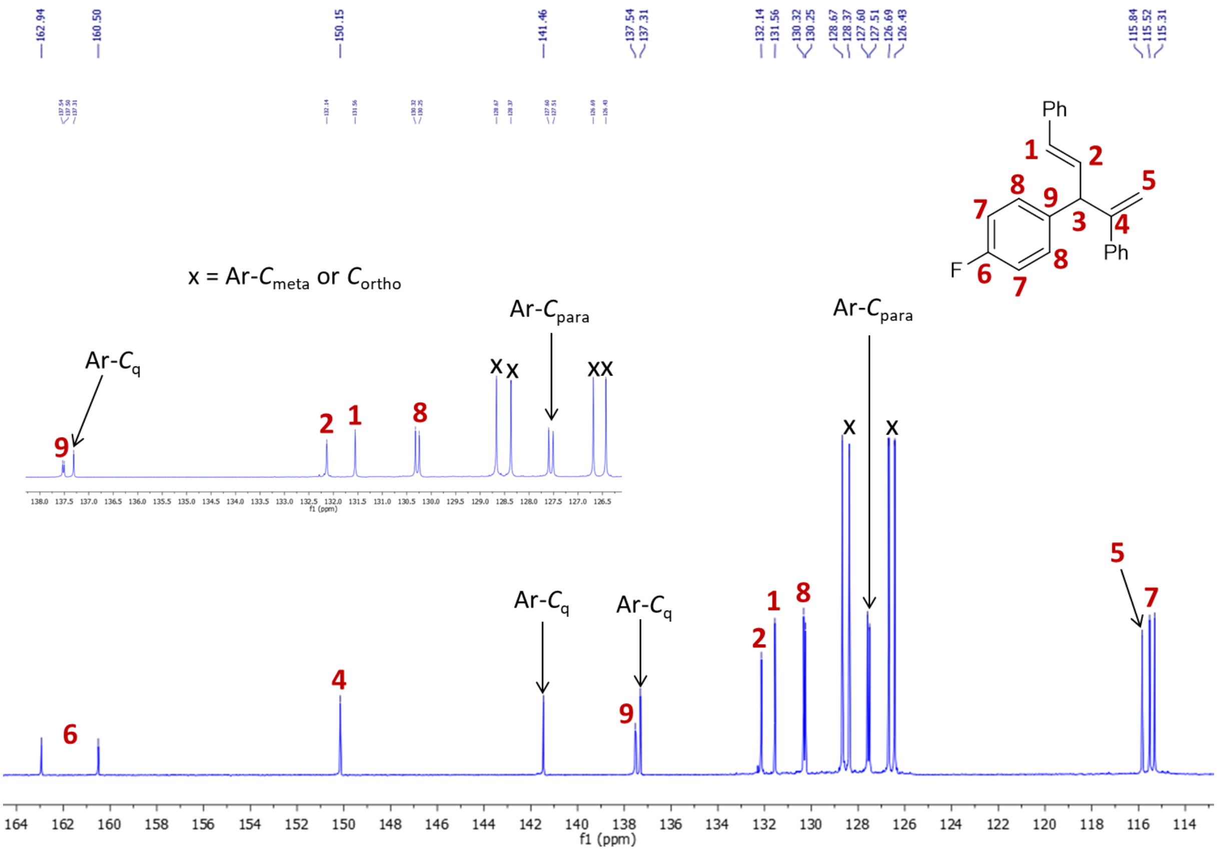

Figure SI 68: Excerpt of the ^{13}C NMR spectrum (*E*)-1,4-diphenyl-2-(*para*-fluorophenyl)-penta-1,4-diene **6f** (101 MHz, chloroform-*d*).

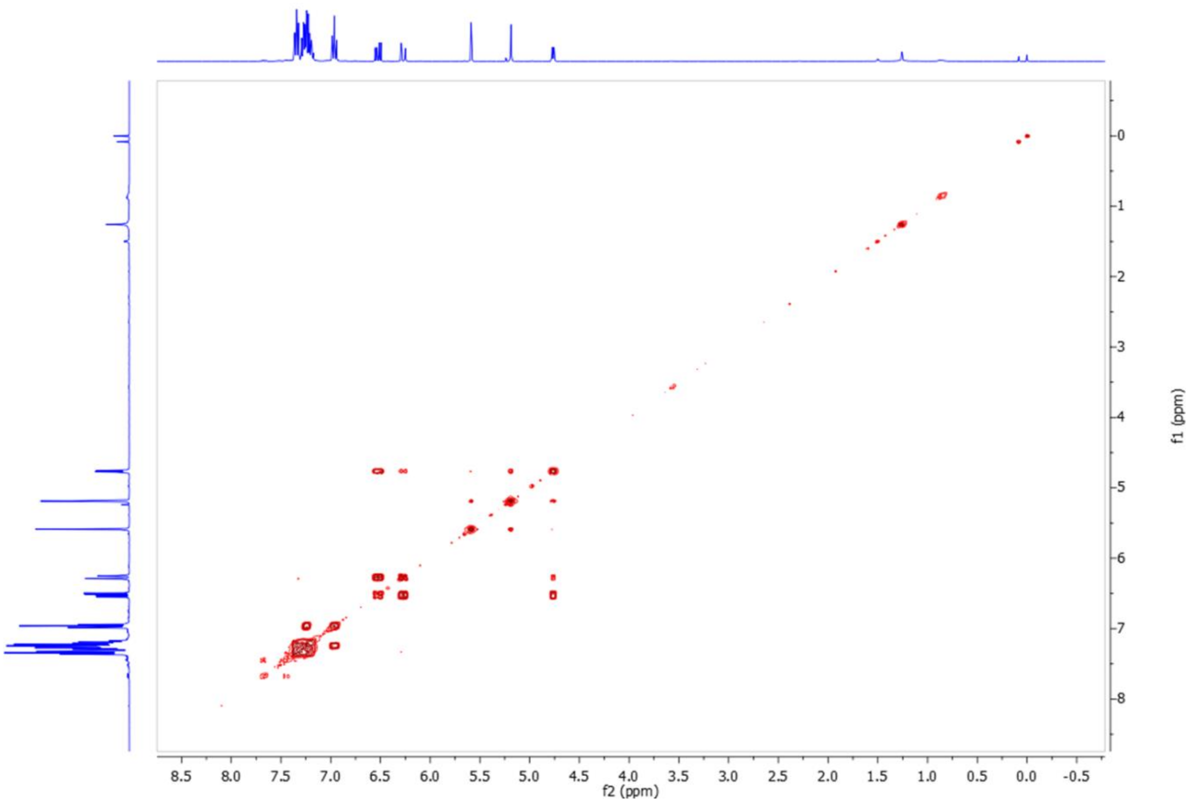

Figure SI 69: HH COSY NMR spectrum of (*E*)-1,4-diphenyl-2-(*para*-fluorophenyl)-penta-1,4-diene **6f** (400 MHz, chloroform-*d*).

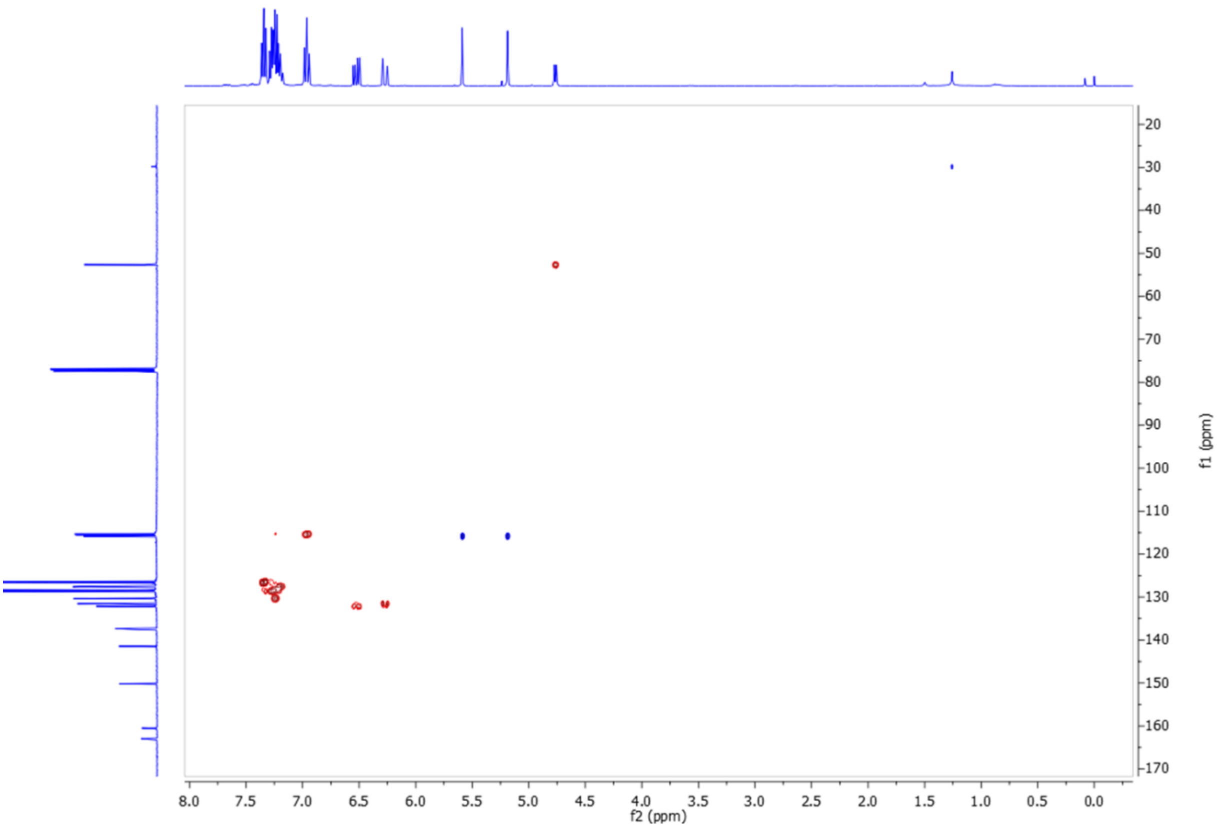

Figure SI 70: HSQC NMR spectrum of (*E*)-1,4-diphenyl-2-(*para*-fluorophenyl)-penta-1,4-diene **6f** (101 MHz, chloroform-*d*).

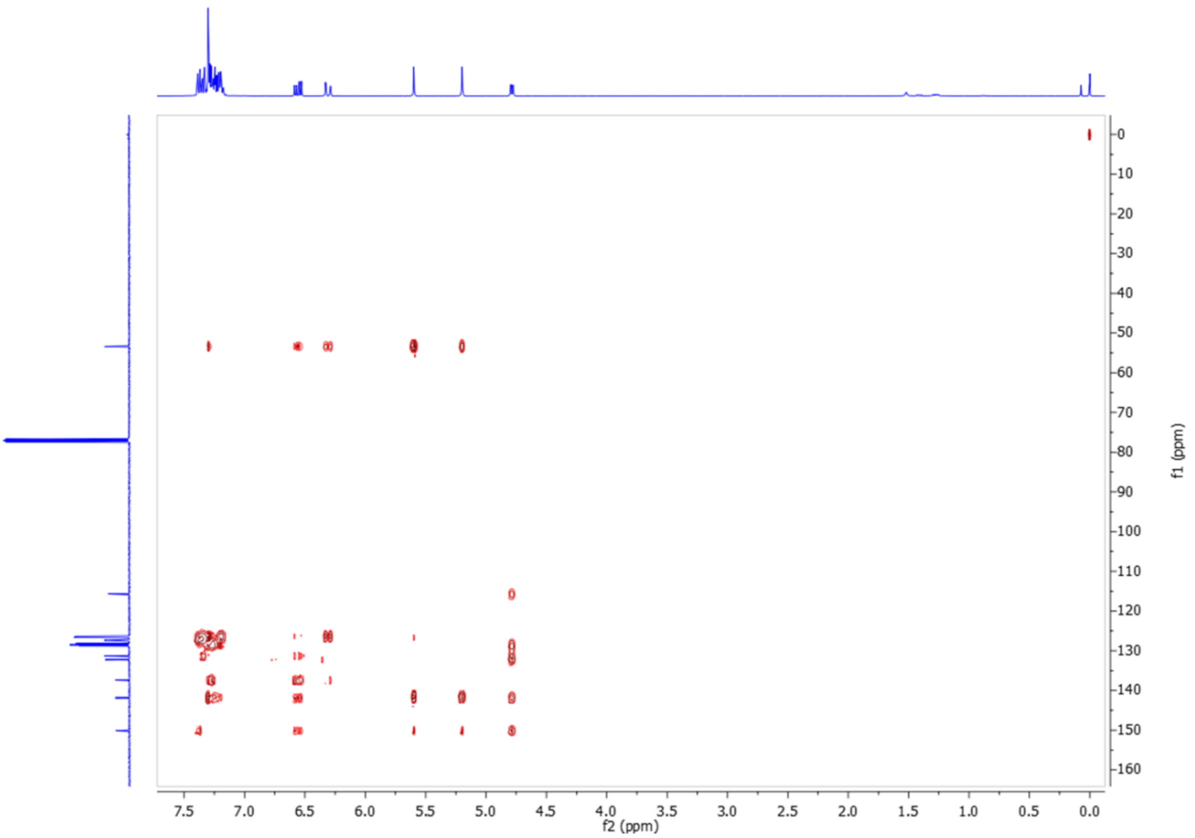

Figure SI 71: HMBC NMR spectrum of (*E*)-1,4-diphenyl-2-(*para*-fluorophenyl)-penta-1,4-diene **6f** (101 MHz, chloroform-*d*).

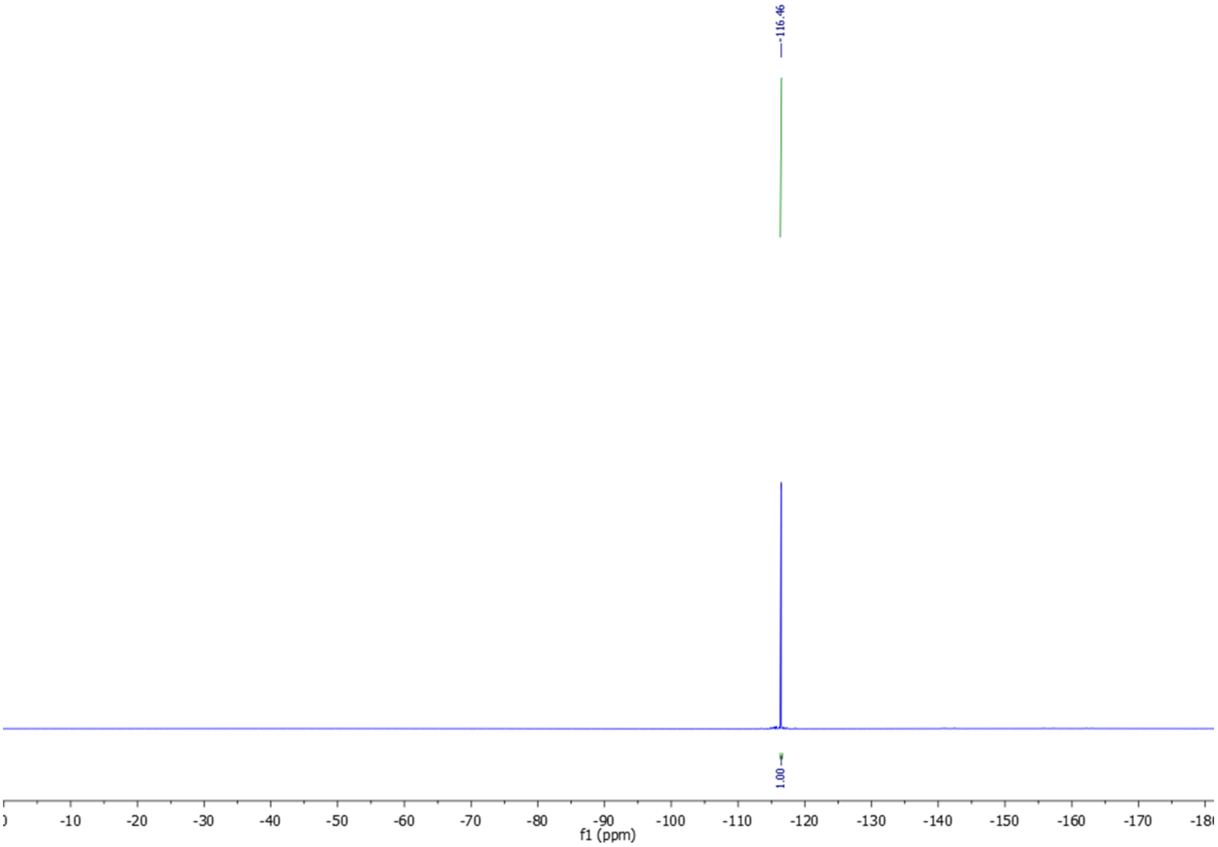

Figure SI 72: ^{19}F NMR spectrum of (*E*)-1,4-diphenyl-2-(*para*-fluorophenyl)-penta-1,4-diene **6f** (377 MHz, chloroform-*d*).

9.3.7 1,4-diene 7a

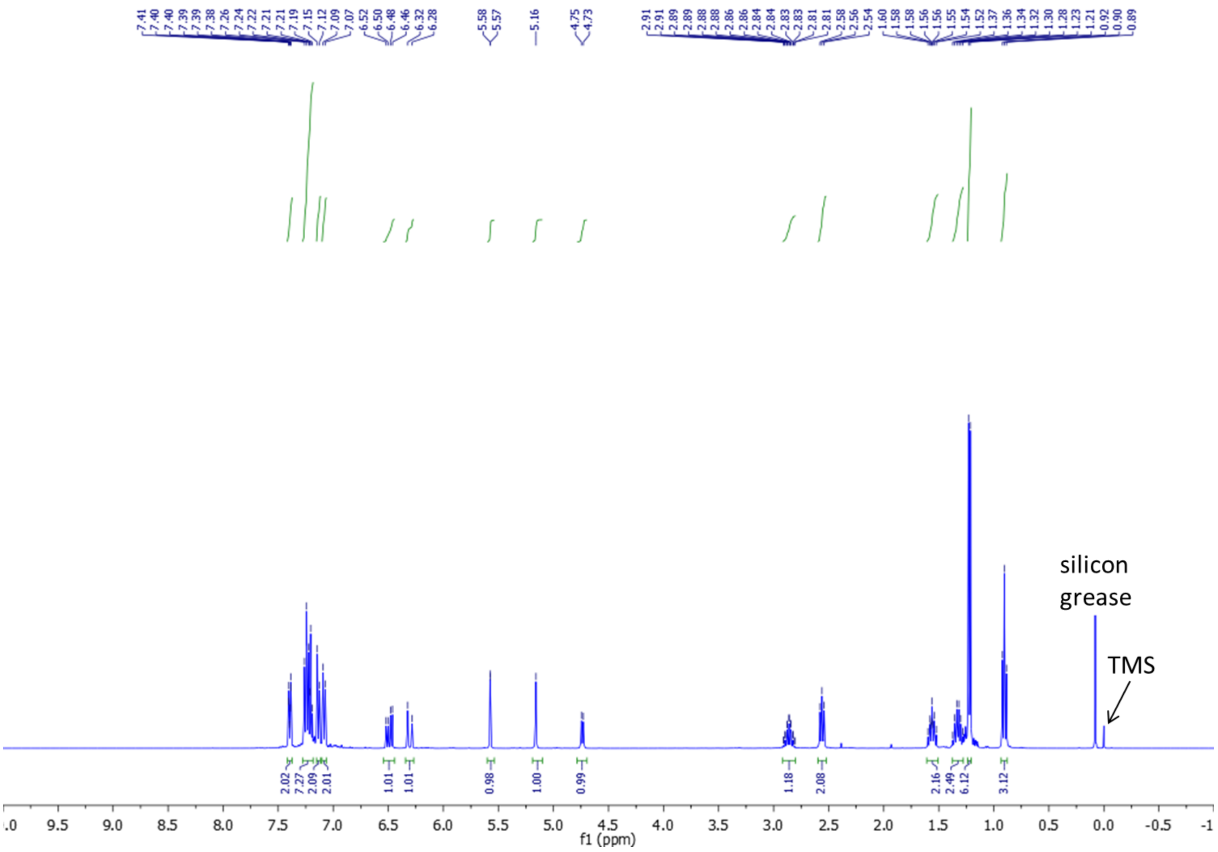

Figure SI 73: ^{1}H NMR spectrum of (*E*)-1-(*para*-*n*-butylphenyl)-3-(*para*-*iso*propylphenyl)-3-phenyl-penta-1,4-diene diene **7a** (400 MHz, chloroform-*d*).

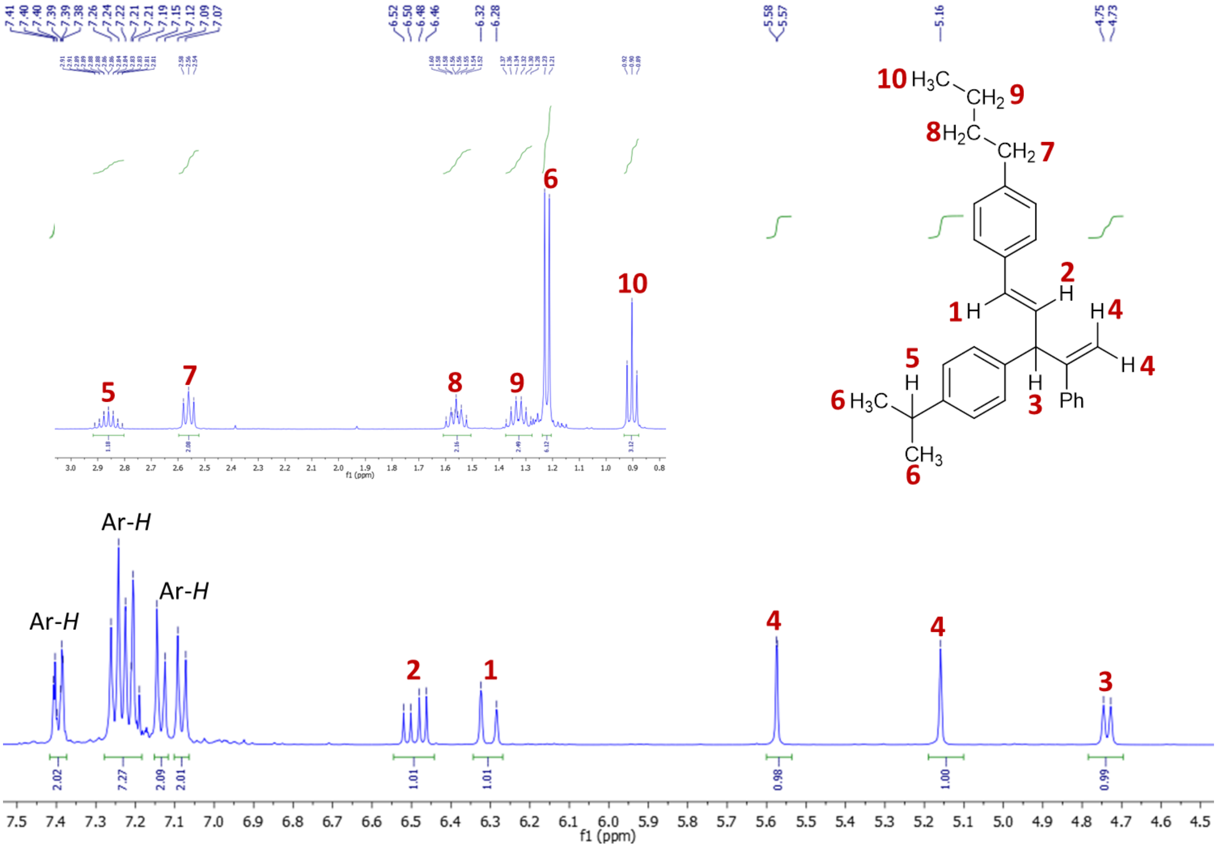

Figure SI 74: Excerpt of ^{1}H NMR spectrum of (*E*)-1-(*para*-*n*-butylphenyl)-3-(*para*-*iso*propylphenyl)-3-phenyl-penta-1,4-diene **7a** (400 MHz, chloroform-*d*).

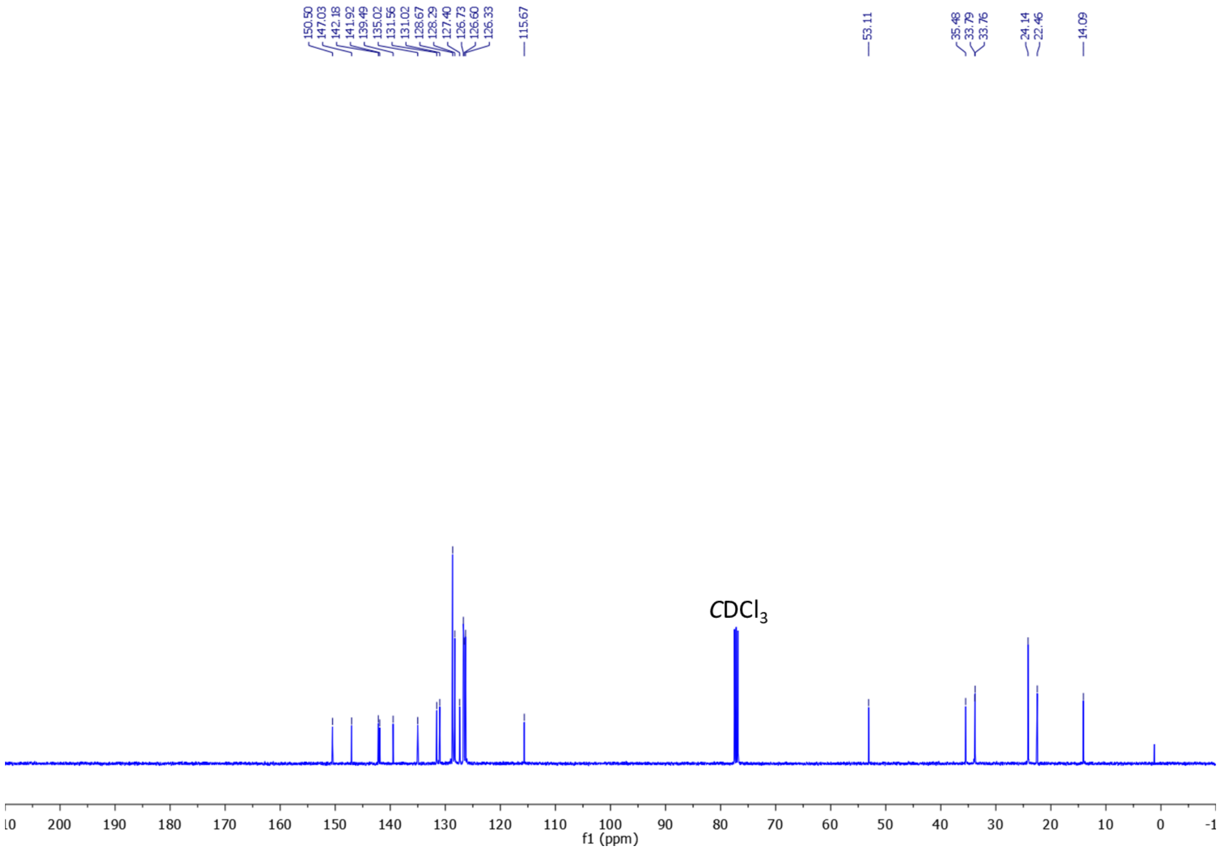

Figure SI 75: ^{13}C NMR spectrum of (*E*)-1-(*para*-*n*-butylphenyl)-3-(*para*-*iso*propylphenyl)-3-phenyl-penta-1,4-diene **7a** (101 MHz, chloroform-*d*).

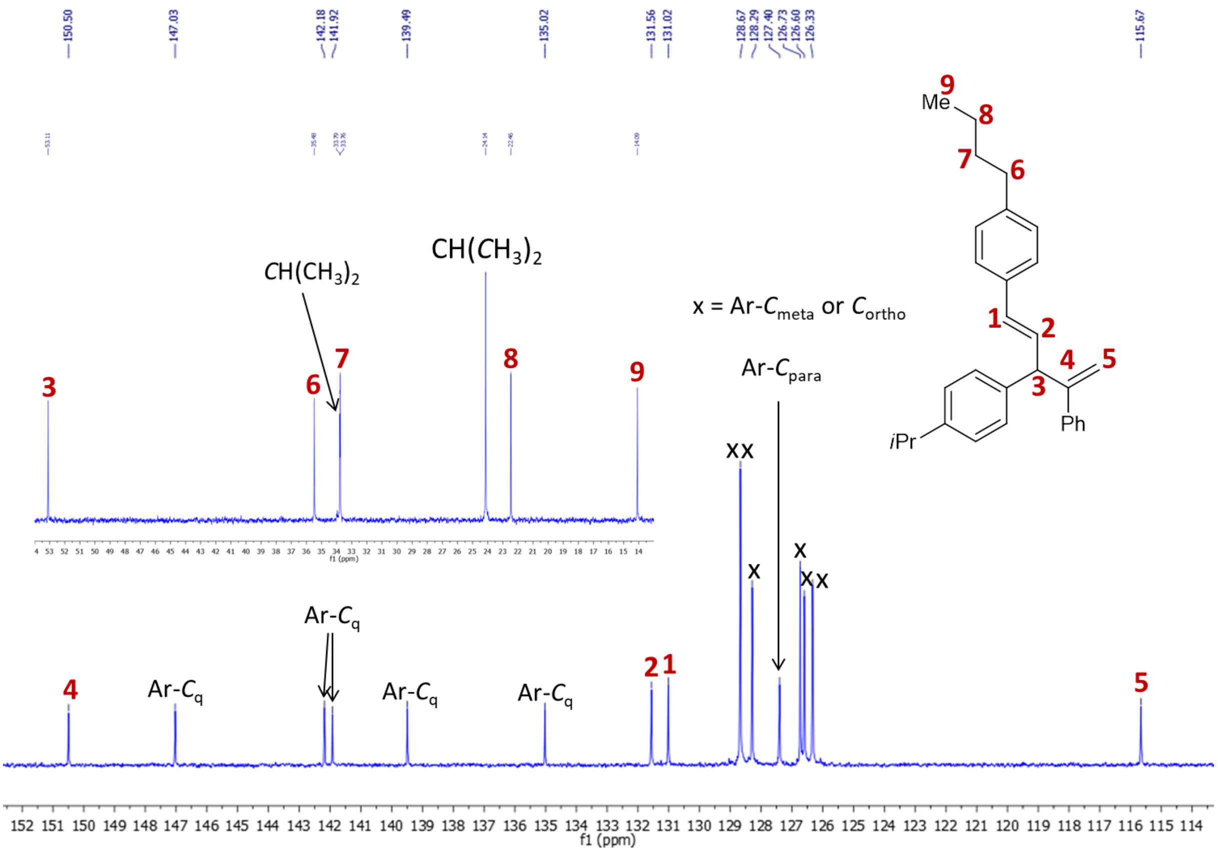

Figure SI 76: Excerpt of the ^{13}C NMR spectrum of (*E*)-1-(*para*-*n*-butylphenyl)-3-(*para*-*iso*propylphenyl)-3-phenyl-penta-1,4-diene **7a** (101 MHz, chloroform-*d*).

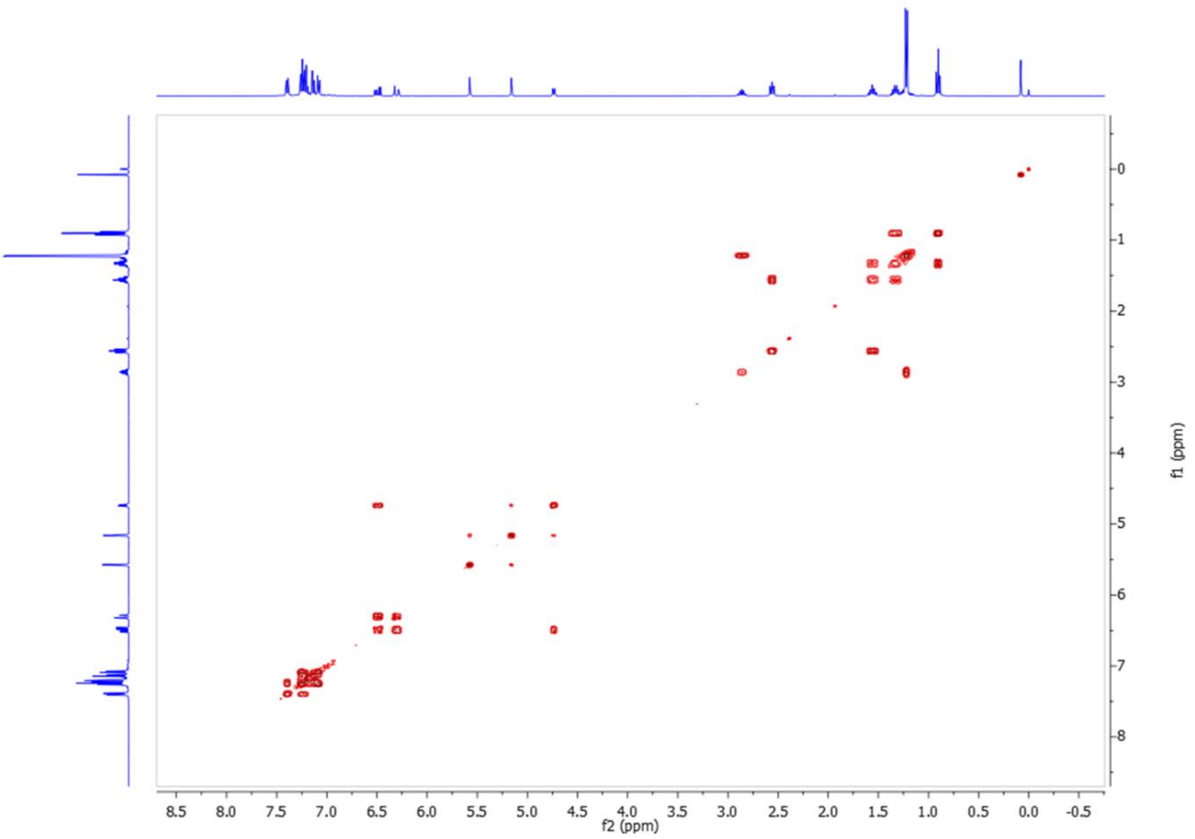

Figure SI 77: HH COSY NMR spectrum of (*E*)-1-(*para*-*n*-butylphenyl)-3-(*para*-*iso*propylphenyl)-3-phenyl-penta-1,4-diene **7a** (400 MHz, chloroform-*d*).

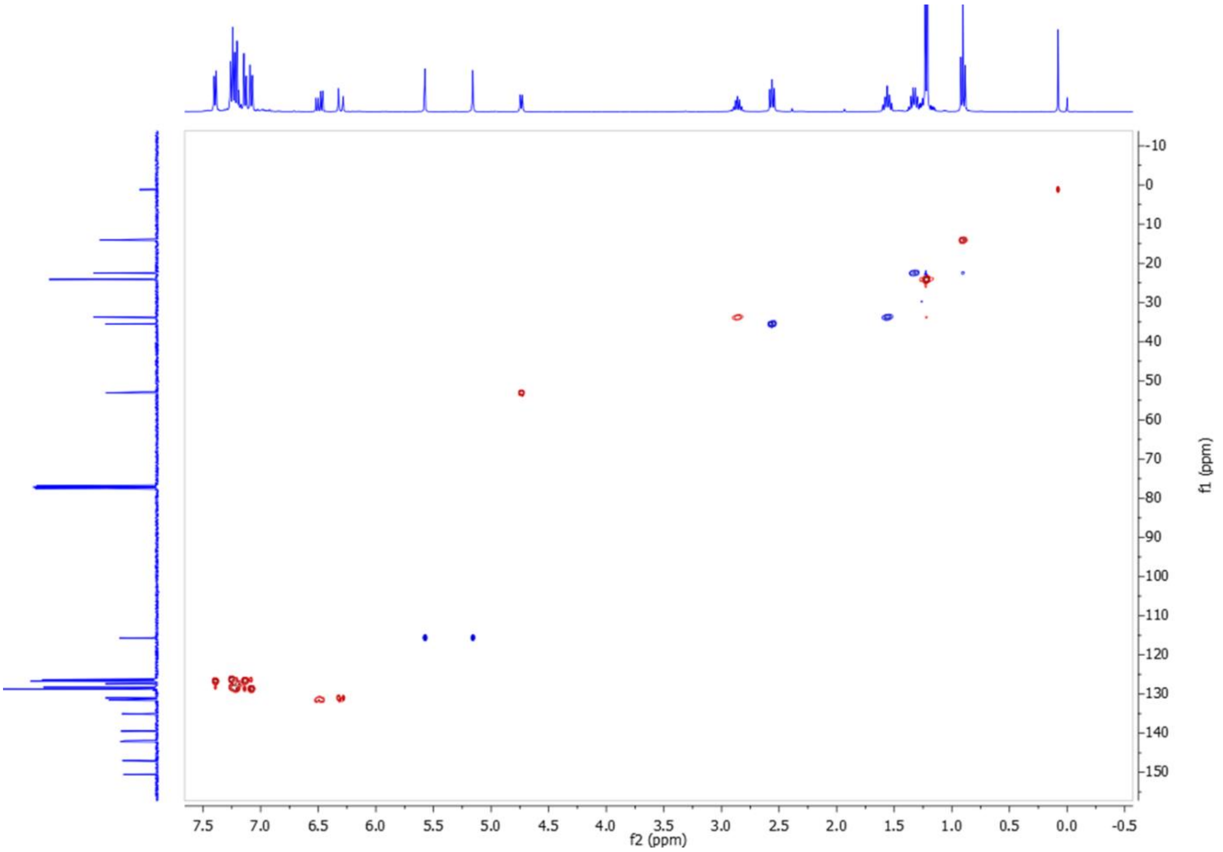

Figure SI 78: HSQC NMR spectrum of (*E*)-1-(*para*-*n*-butylphenyl)-3-(*para*-*iso*propylphenyl)-3-phenyl-penta-1,4-diene **7a** (101 MHz, chloroform-*d*).

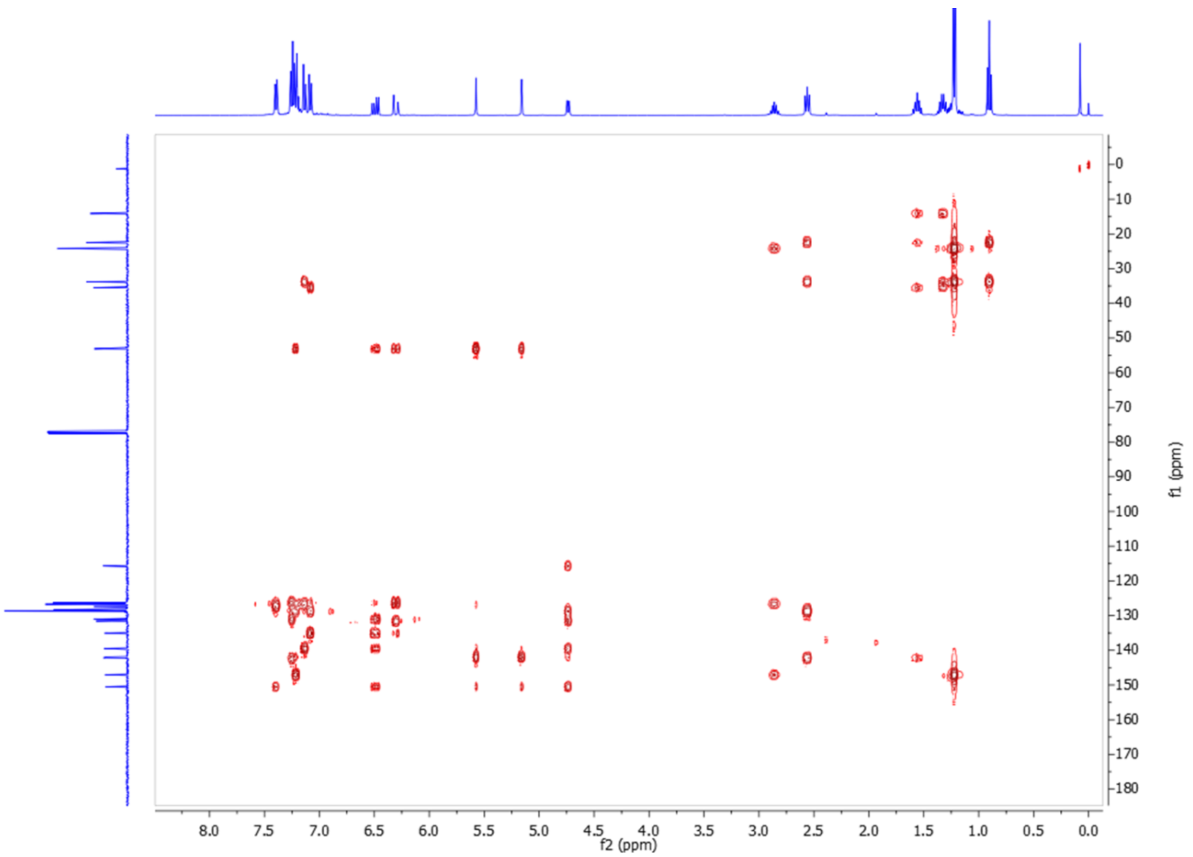

Figure SI 79: HMBC NMR spectrum of (*E*)-1-(*para*-*n*-butylphenyl)-3-(*para*-*iso*propylphenyl)-3-phenyl-penta-1,4-diene **7a** (101 MHz, chloroform-*d*).

9.3.8 1,4-diene 7b

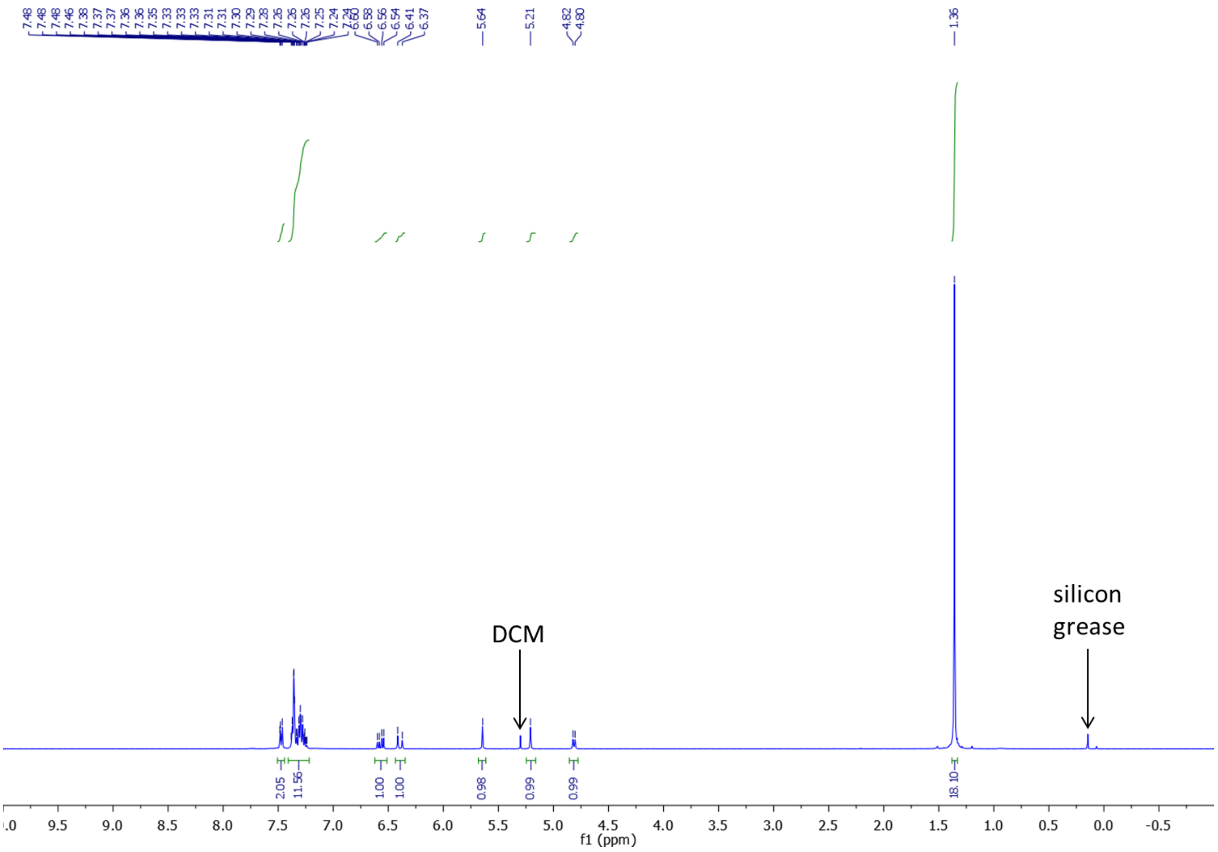

Figure SI 80: ^{1}H NMR spectrum of (*E*)-1,3-bis(*para*-*tert*-butylphenyl)-4-phenyl-penta-1,4-diene **7b** (400 MHz, chloroform-*d*).

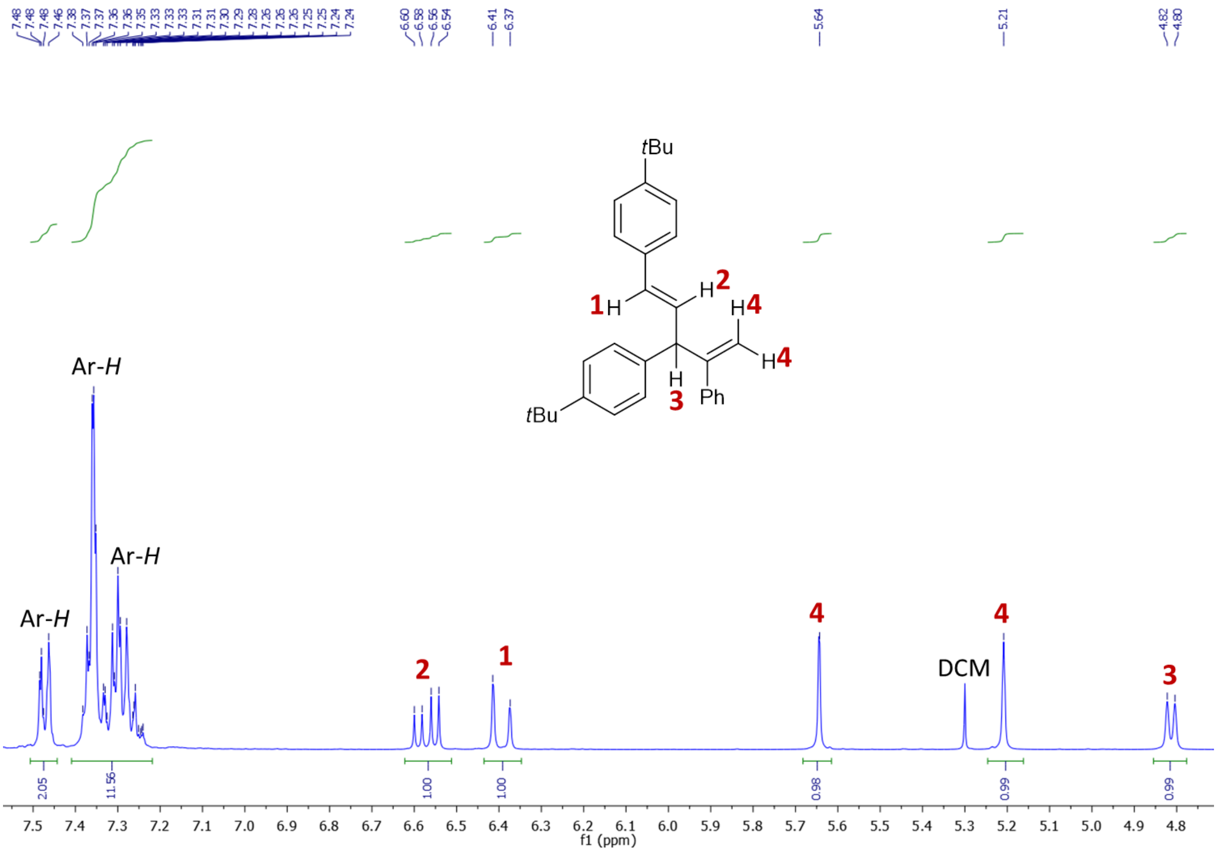

Figure SI 81: Excerpt of ^{1}H NMR spectrum of (*E*)-1,3-bis(*para*-*tert*-butylphenyl)-4-phenyl-penta-1,4-diene **7b** (400 MHz, chloroform-*d*).

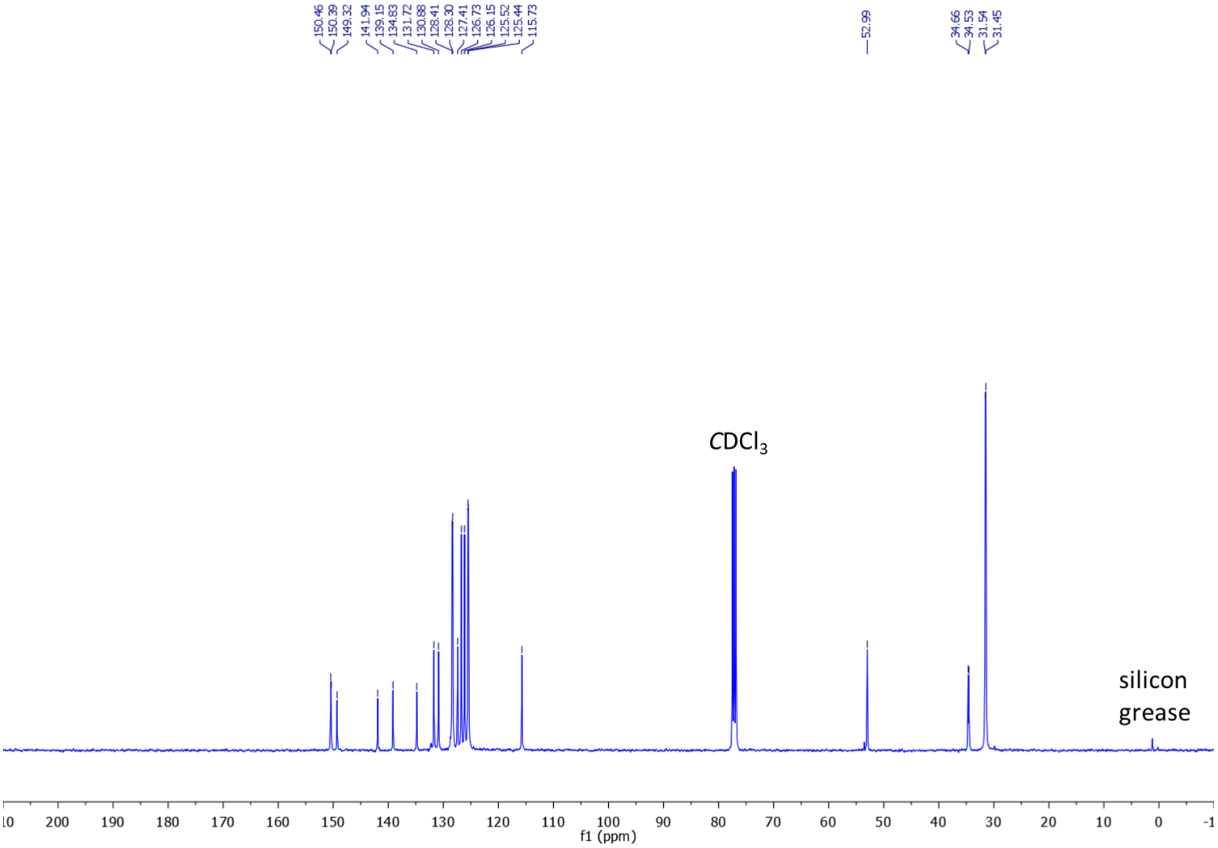

Figure SI 82: ^{13}C NMR spectrum of (*E*)-1,3-bis(*para*-*tert*-butylphenyl)-4-phenyl-penta-1,4-diene **7b** (101 MHz, chloroform-*d*).

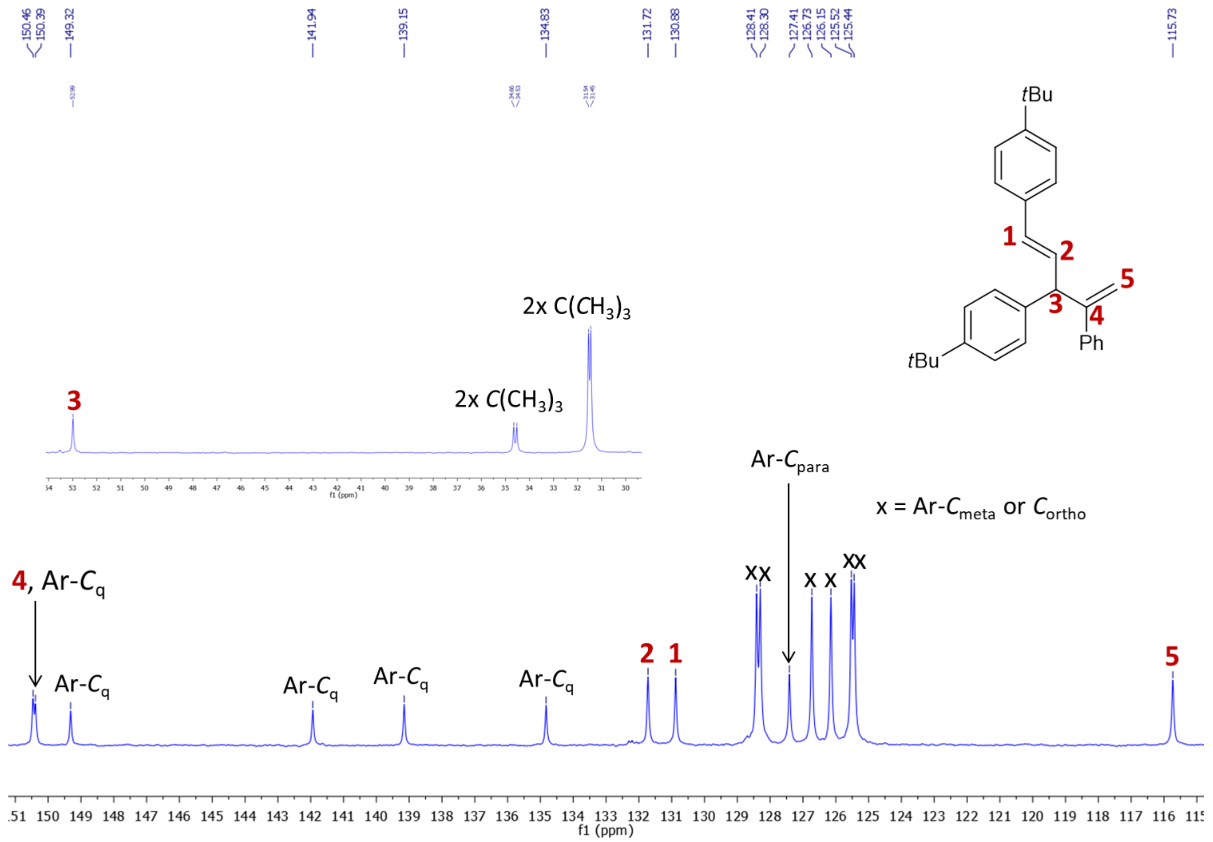

Figure SI 83: Excerpt of the ^{13}C NMR spectrum of (*E*)-1,3-bis(*para*-*tert*-butylphenyl)-4-phenyl-penta-1,4-diene **7b** (101 MHz, chloroform-*d*).

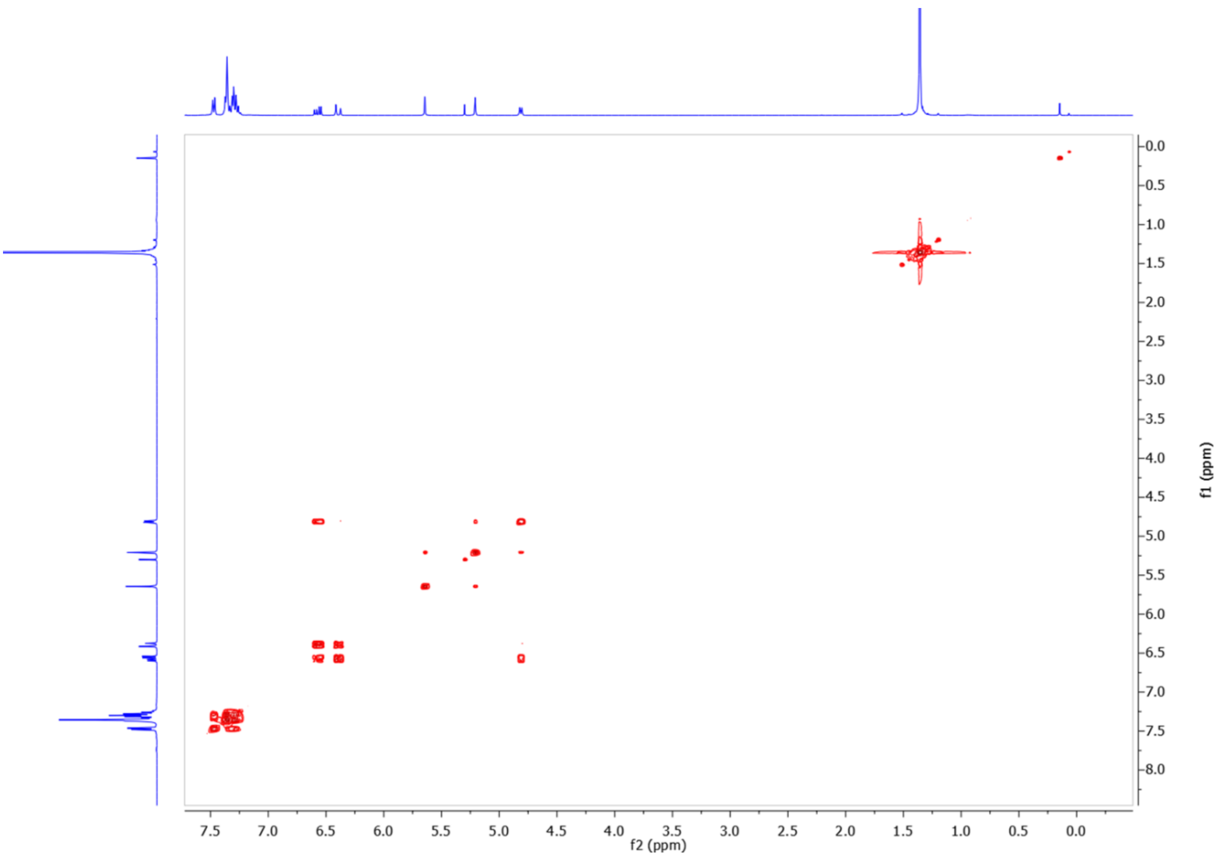

Figure SI 84: HH COSY NMR spectrum of (*E*)-1,3-bis(*para*-*tert*-butylphenyl)-4-phenyl-penta-1,4-diene **7b** (400 MHz, chloroform-*d*).

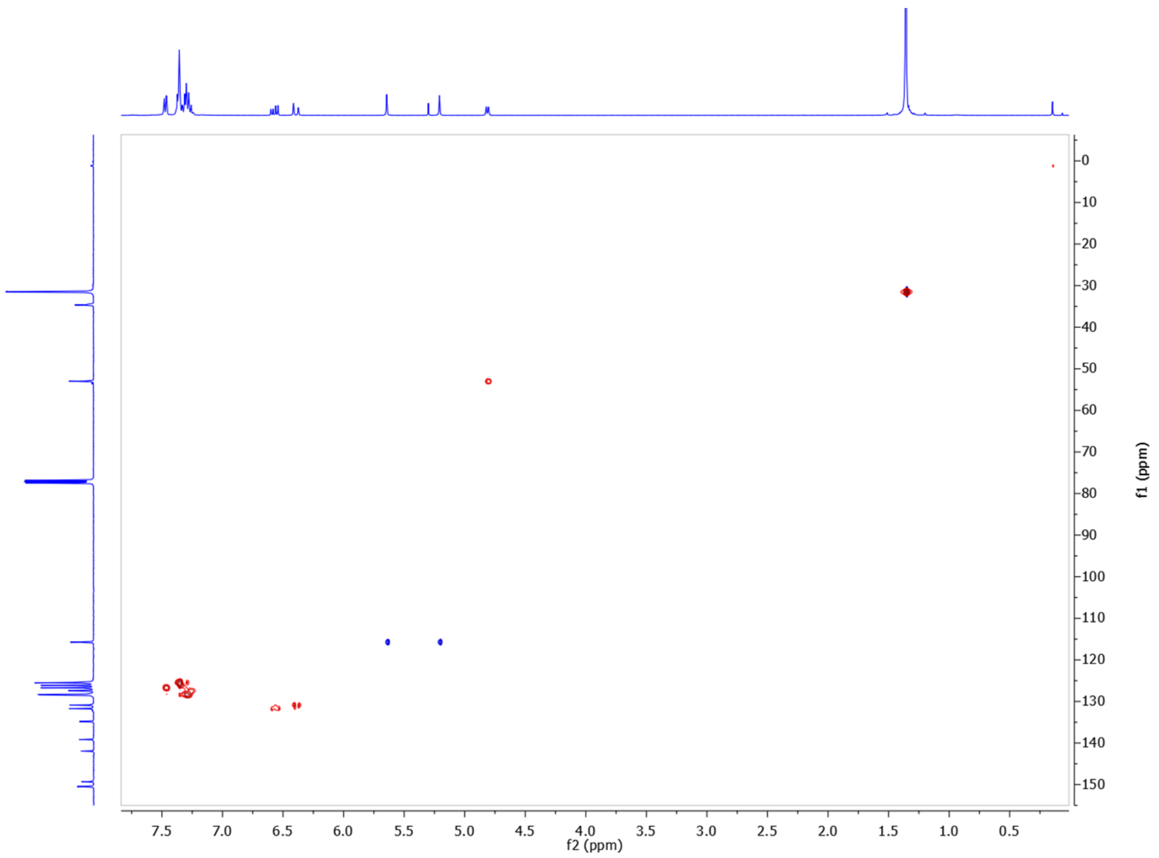

Figure SI 85: HSQC NMR spectrum of (*E*)-1,3-bis(*para*-*tert*-butylphenyl)-4-phenyl-penta-1,4-diene **7b** (101 MHz, chloroform-*d*).

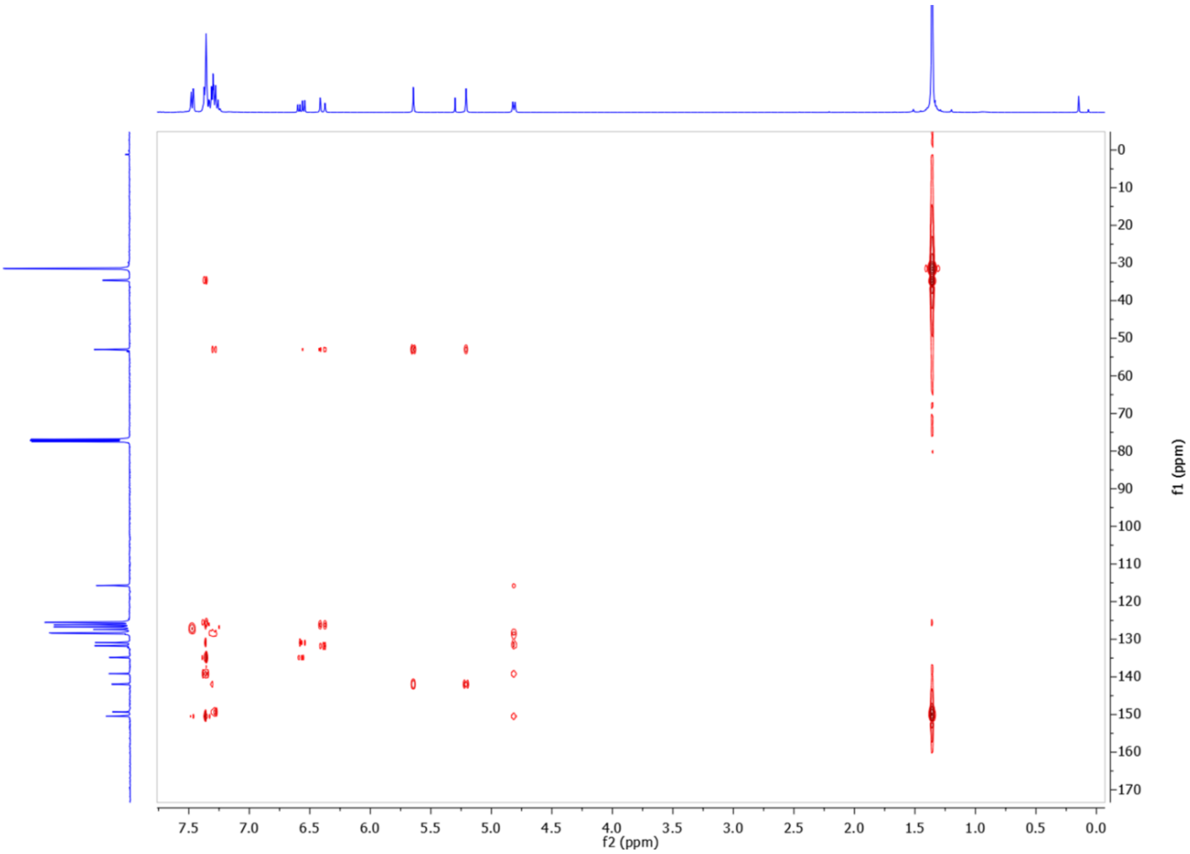

Figure SI 86: HMBC NMR spectrum of (*E*)-1,3-bis(*para*-*tert*-butylphenyl)-4-phenyl-penta-1,4-diene **7b** (101 MHz, chloroform-*d*).

9.3.9 1,4-diene 7c

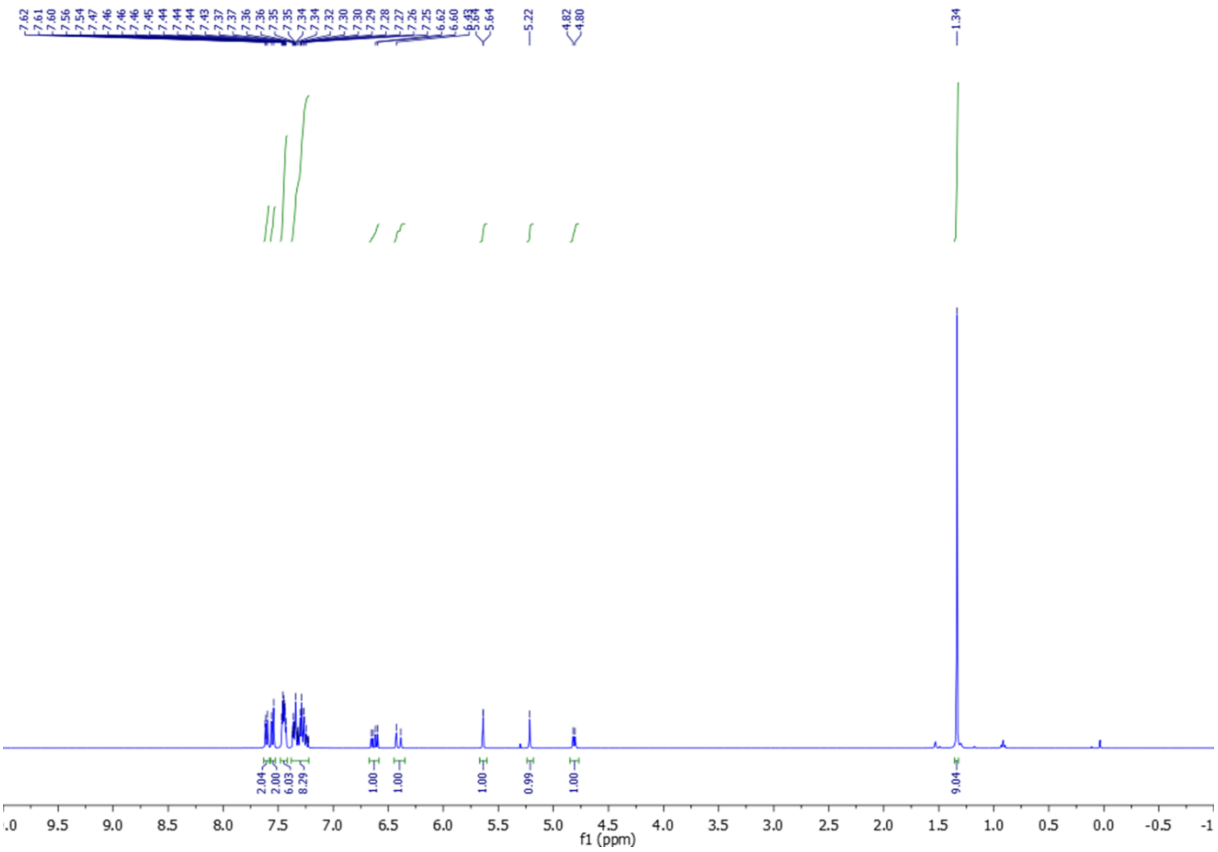

Figure SI 87: ^{1}H NMR spectrum of (*E*)-1-(4-biphenyl)-3-(*para*-*tert*-butylphenyl)-4-phenyl-penta-1,4-diene **7c** (400 MHz, chloroform-*d*).

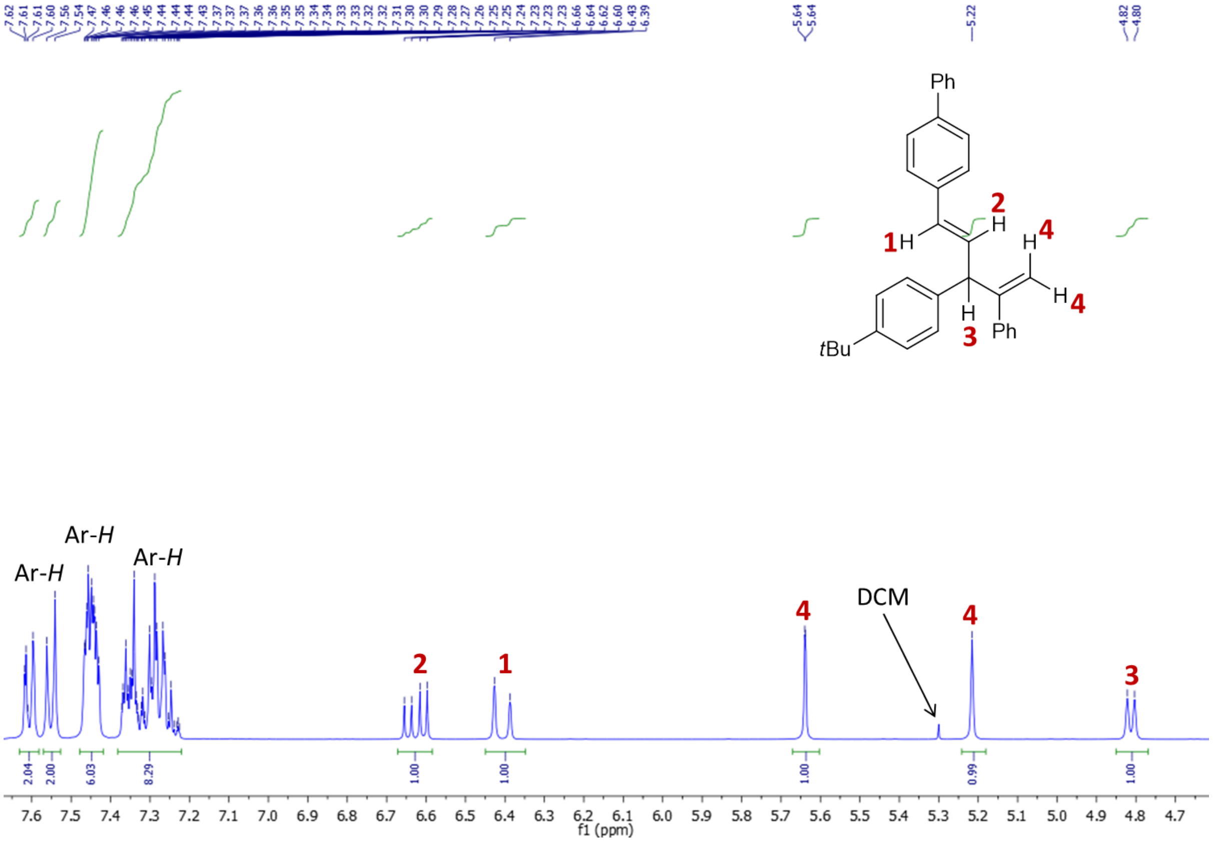

Figure SI 88: Excerpt of ^{1}H NMR spectrum of (*E*)-1-(4-biphenyl)-3-(*para*-*tert*-butylphenyl)-4-phenyl-penta-1,4-diene **7c** (400 MHz, chloroform-*d*).

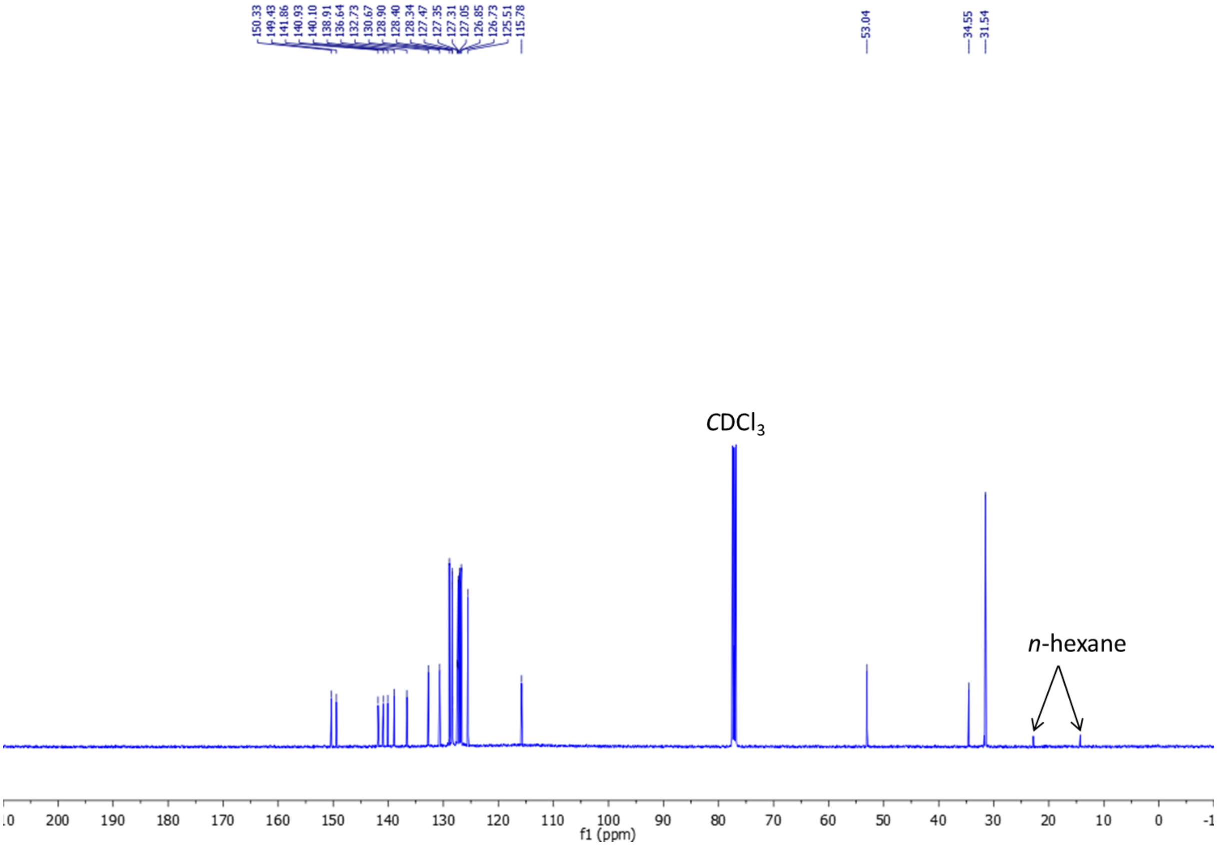

Figure SI 89: ^{13}C NMR spectrum of (*E*)-1-(4-biphenyl)-3-(*para*-*tert*-butylphenyl)-4-phenyl-penta-1,4-diene **7c** (101 MHz, chloroform-*d*).

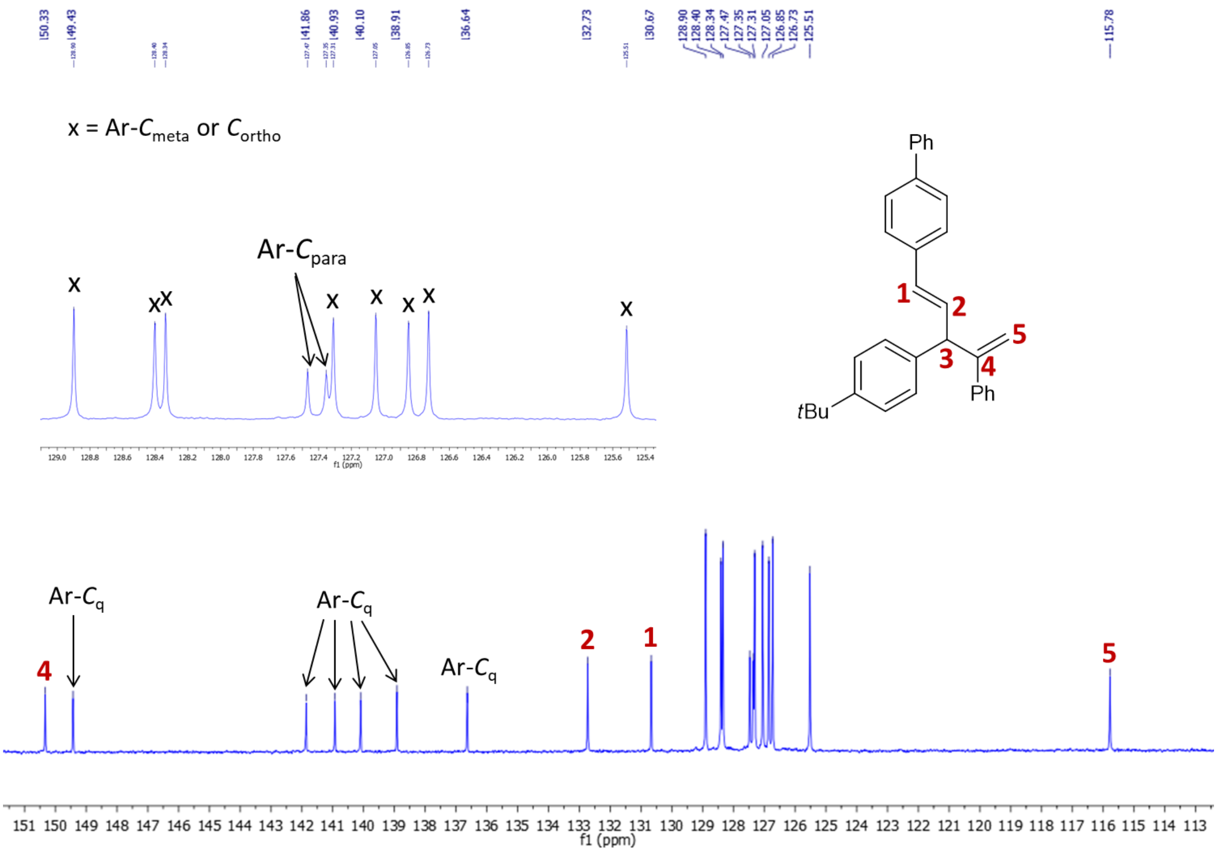

Figure SI 90: Excerpt of the ^{13}C NMR spectrum of (*E*)-1-(4-biphenyl)-3-(*para*-*tert*-butylphenyl)-4-phenyl-penta-1,4-diene **7c** (101 MHz, chloroform-*d*).

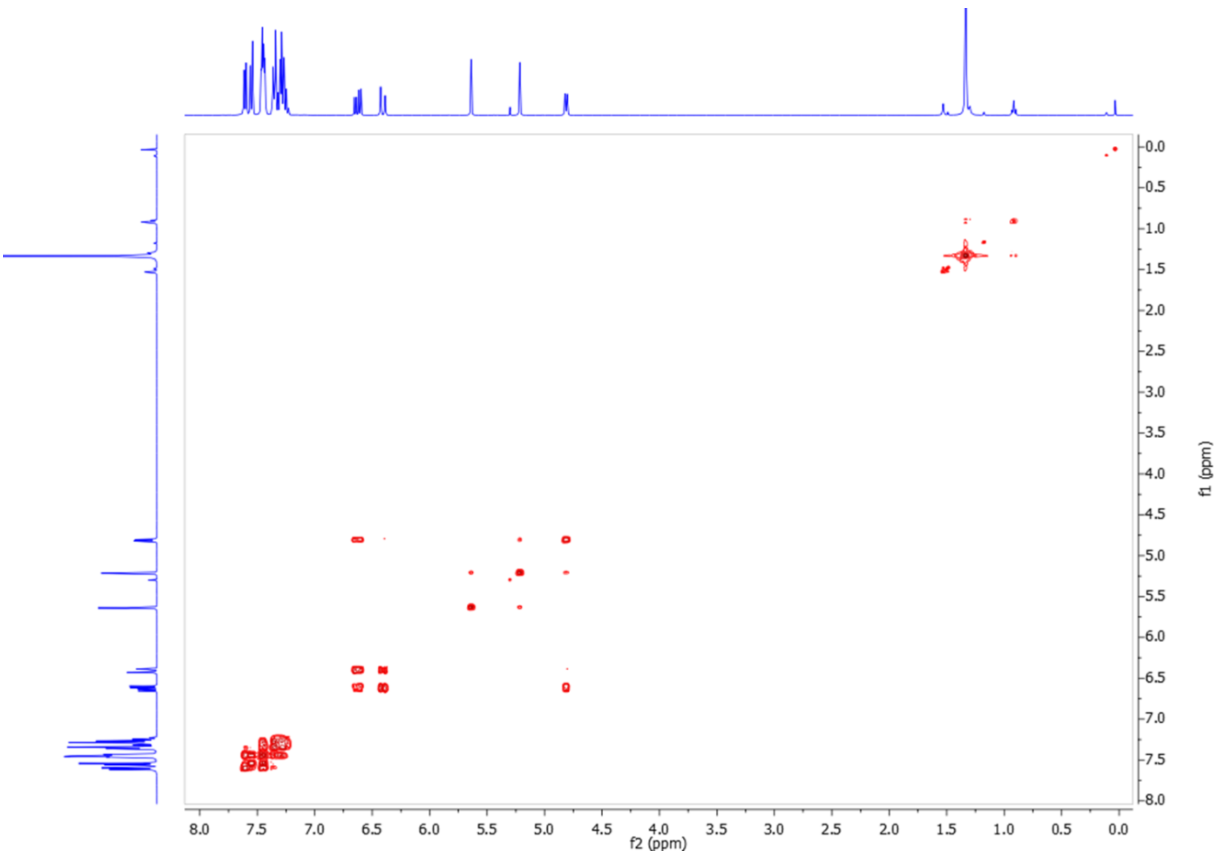

Figure SI 91: HH COSY NMR spectrum of (*E*)-1-(4-biphenyl)-3-(*para*-*tert*-butylphenyl)-4-phenyl-penta-1,4-diene **7c** (400 MHz, chloroform-*d*).

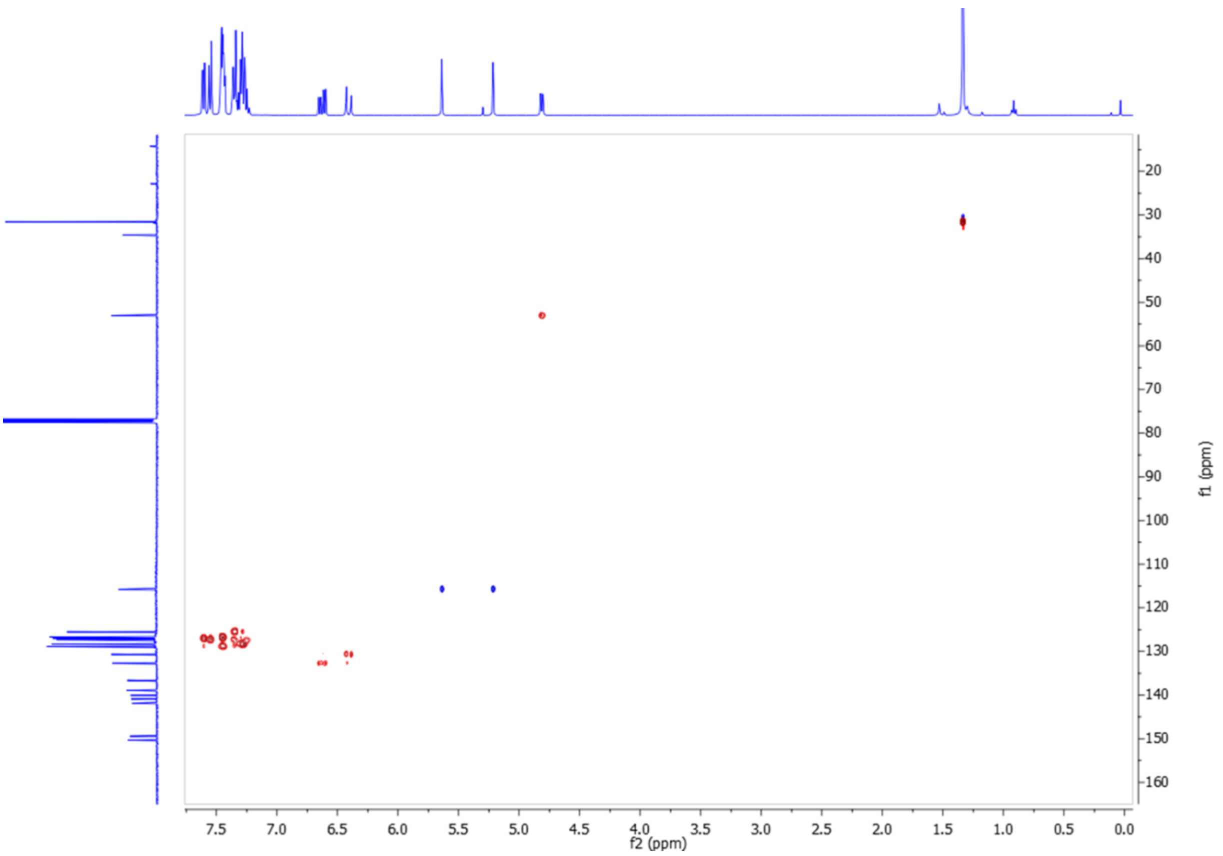

Figure SI 92: HSQC NMR spectrum of (*E*)-1-(4-biphenyl)-3-(*para-tert*-butylphenyl)-4-phenyl-penta-1,4-diene **7c** (101 MHz, chloroform-*d*).

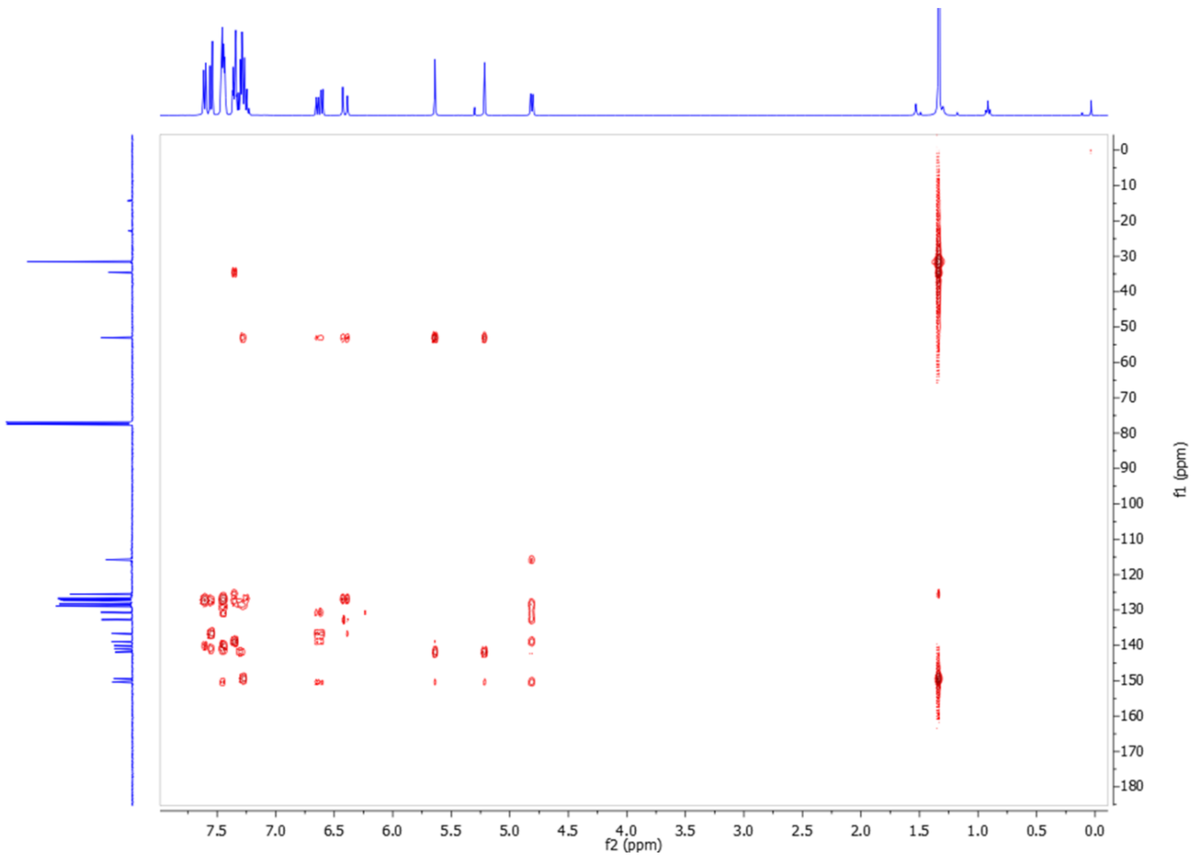

Figure SI 93: HMBC NMR spectrum of (*E*)-1-(4-biphenyl)-3-(*para*-*tert*-butylphenyl)-4-phenyl-penta-1,4-diene **7c** (101 MHz, chloroform-*d*).

9.3.10 1,4-diene 7d

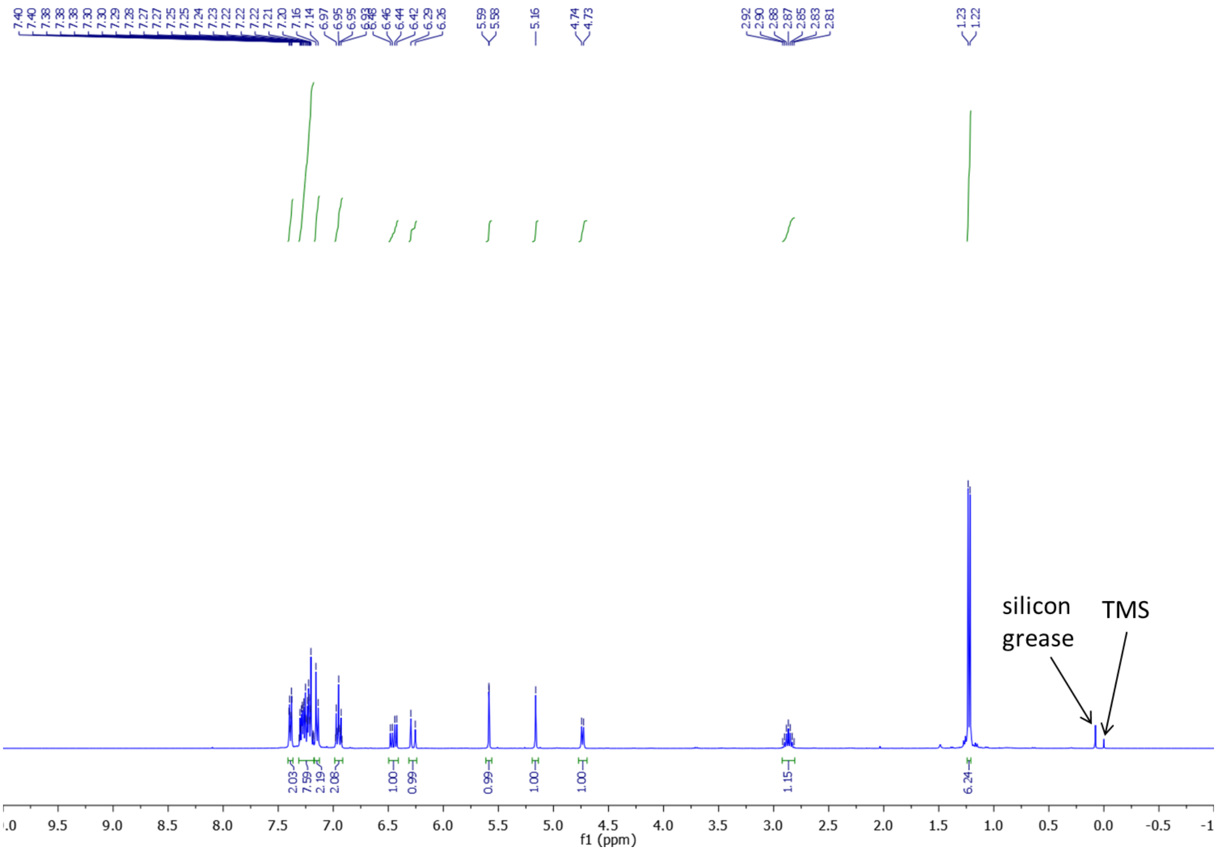

Figure SI 94: ^{1}H NMR spectrum of (*E*)-1-(*para*-fluorophenyl)-3-(*para*-*iso*-propylphenylallene)-4-phenyl-penta-1,4-diene **7d** (400 MHz, chloroform-*d*).

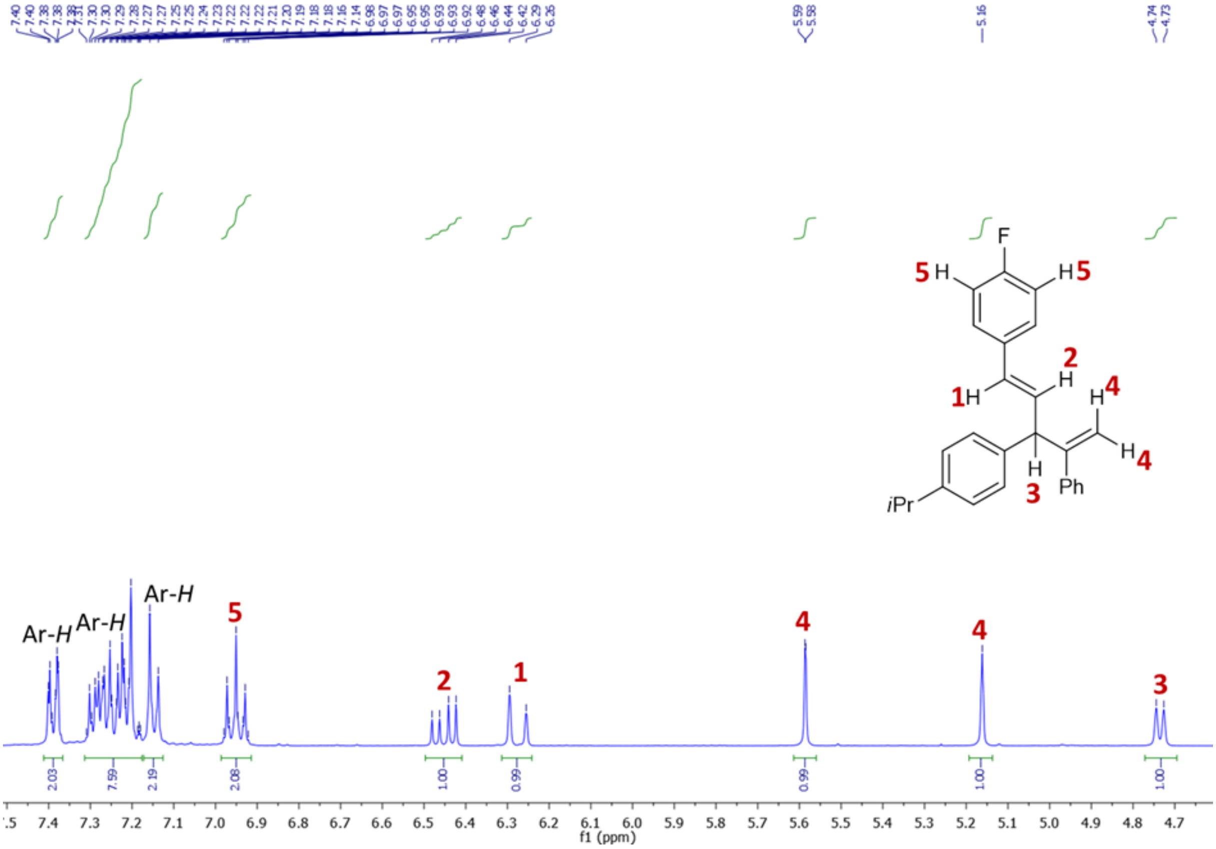

Figure SI 95: Excerpt of ^{1}H NMR spectrum of (*E*)-1-(*para*-fluorophenyl)-3-(*para*-*iso*-propylphenylallene)-4-phenyl-penta-1,4-diene **7d** (400 MHz, chloroform-*d*).

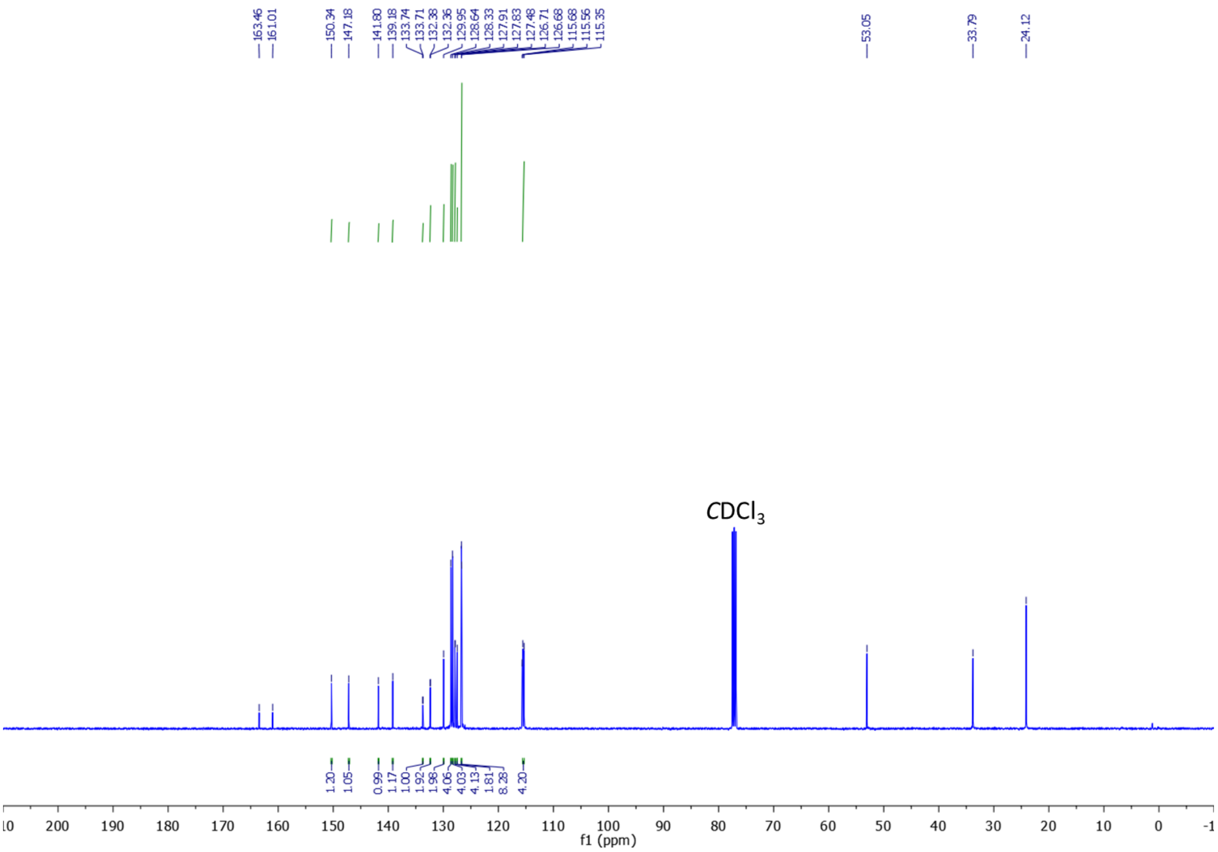

Figure SI 96: ^{13}C NMR spectrum of (*E*)-1-(*para*-fluorophenyl)-3-(*para*-*iso*-propylphenylallene)-4-phenyl-penta-1,4-diene **7d** (101 MHz, chloroform-*d*).

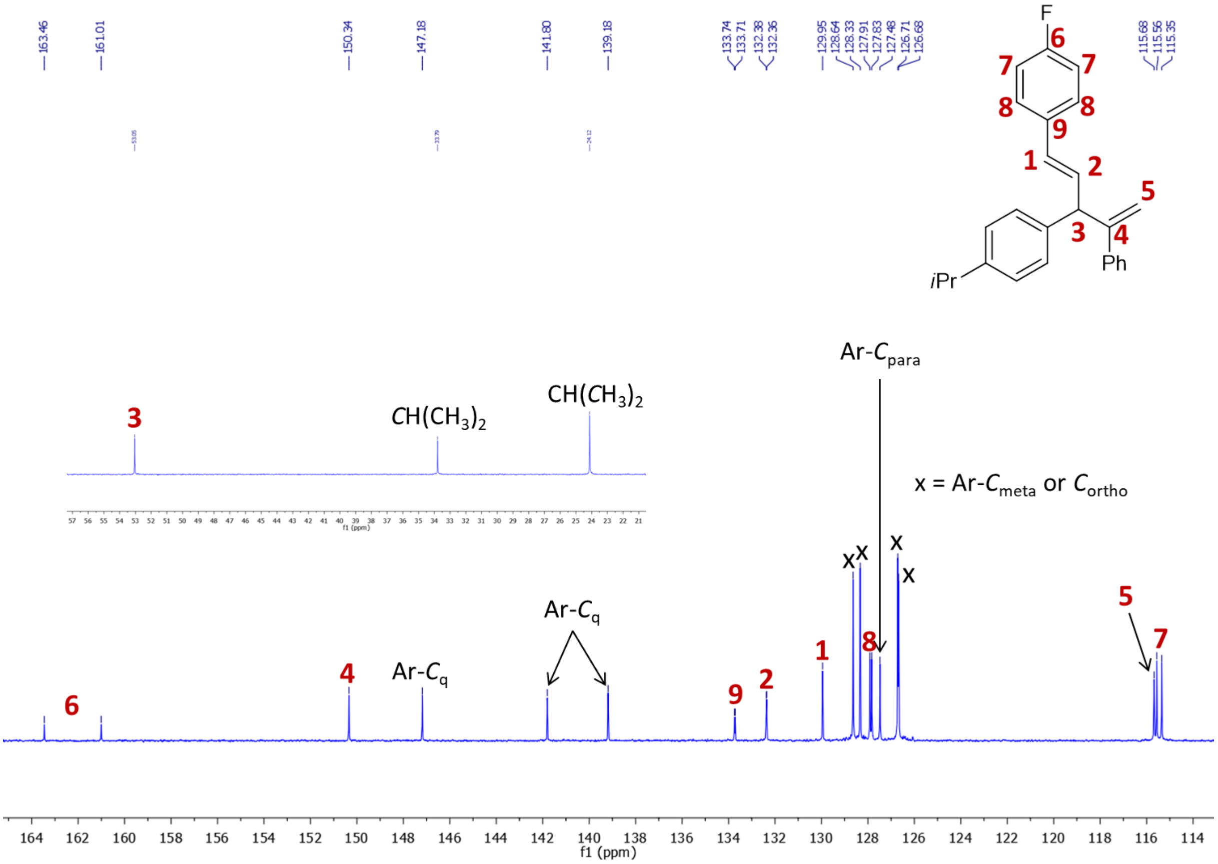

Figure SI 97: Excerpt of the ^{13}C NMR spectrum of (*E*)-1-(*para*-fluorophenyl)-3-(*para*-*iso*-propylphenylallene)-4-phenyl-penta-1,4-diene **7d** (101 MHz, chloroform-*d*).

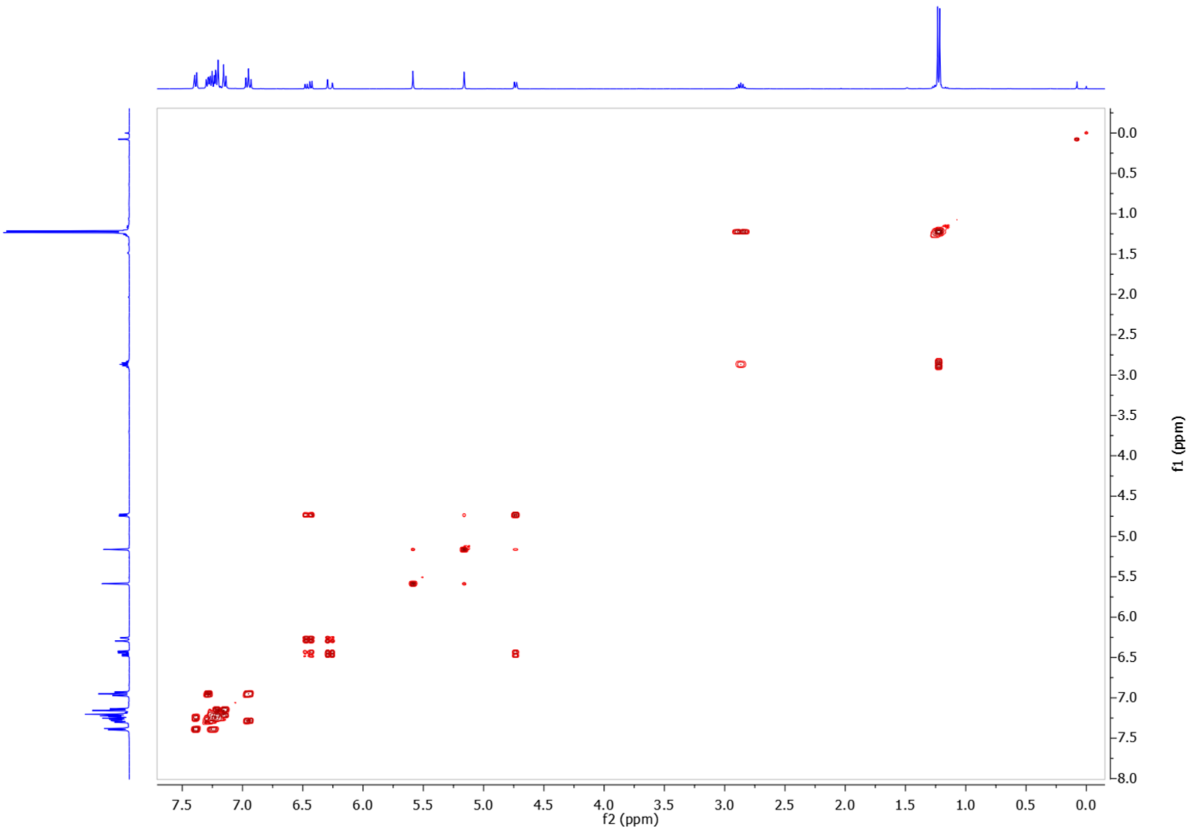

Figure SI 98: HH COSY NMR spectrum of (*E*)-1-(*para*-fluorophenyl)-3-(*para*-*iso*-propylphenylallene)-4-phenyl-penta-1,4-diene **7d** (400 MHz, chloroform-*d*).

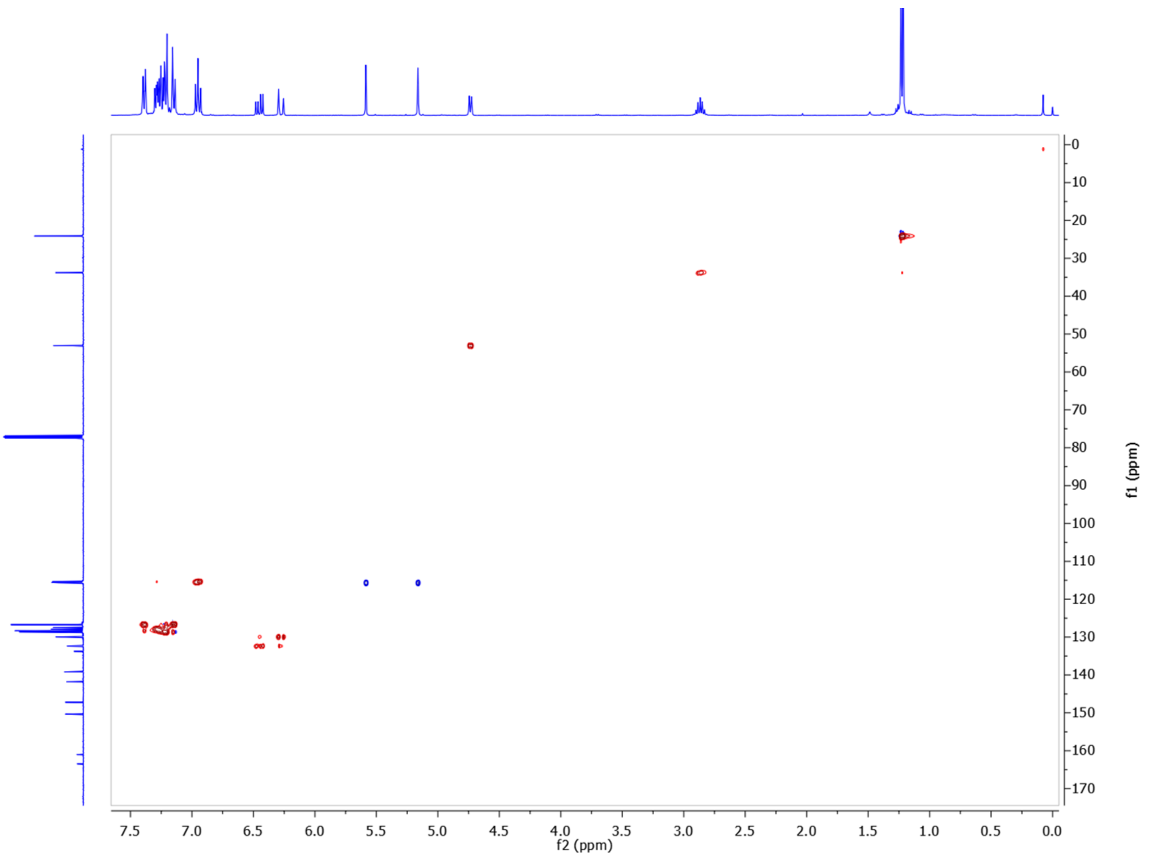

Figure SI 99: HSQC NMR spectrum of (*E*)-1-(*para*-fluorophenyl)-3-(*para*-*iso*-propylphenylallene)-4-phenyl-penta-1,4-diene diene **7d** (101 MHz, chloroform-*d*).

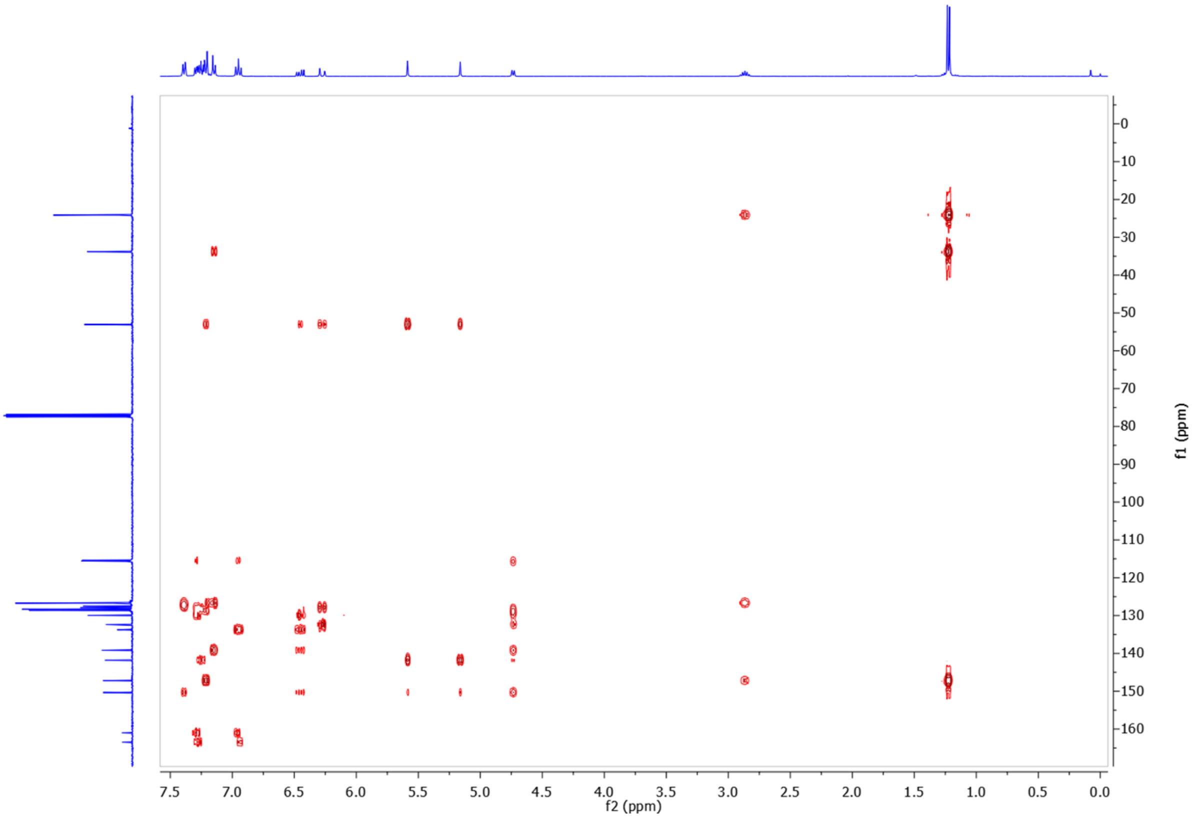

Figure SI 100: HMBC NMR spectrum of (*E*)-1-(*para*-fluorophenyl)-3-(*para*-*iso*-propylphenylallene)-4-phenyl-penta-1,4-diene **7d** (101 MHz, chloroform-*d*).

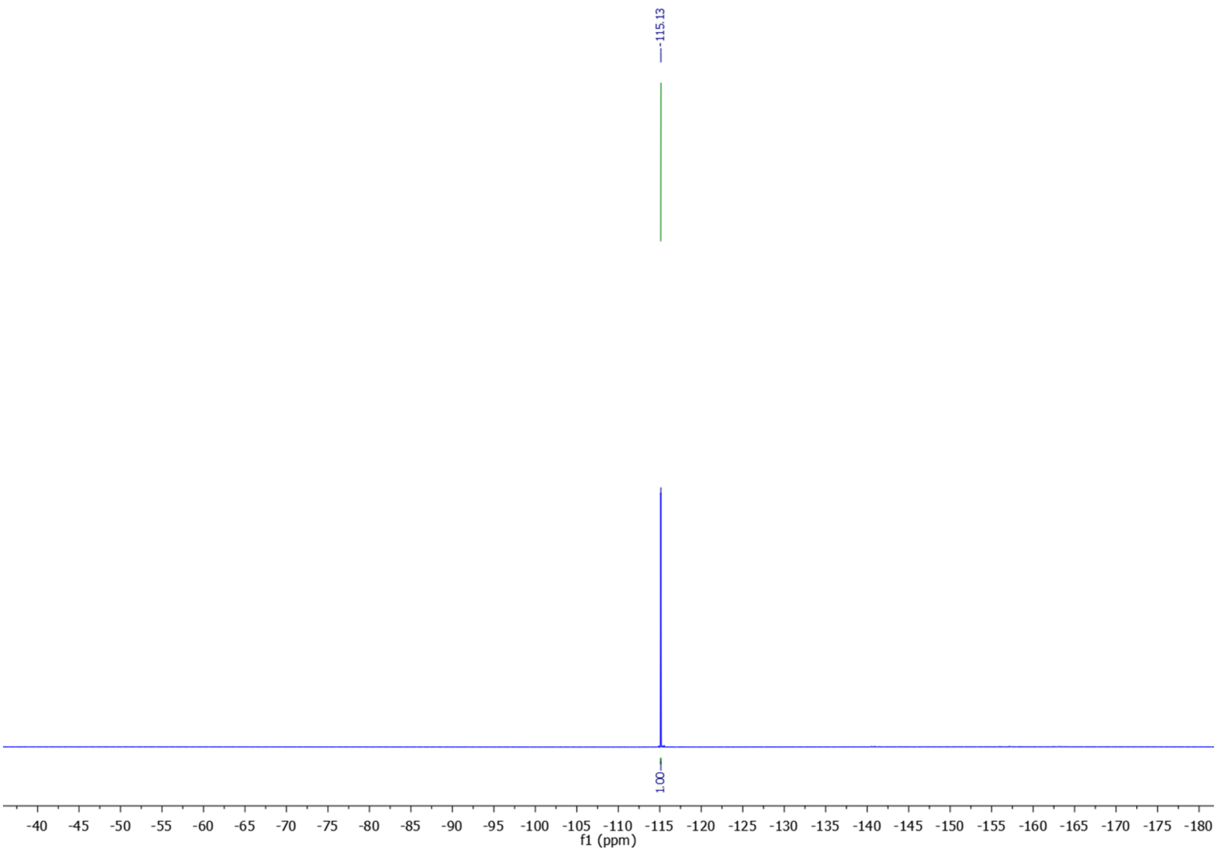

Figure SI 101: ^{19}F NMR spectrum of (*E*)-1-(*para*-fluorophenyl)-3-(*para*-*iso*-propylphenylallene)-4-phenyl-penta-1,4-diene **7d** (377 MHz, chloroform-*d*).

9.3.11 1,4-diene 7e

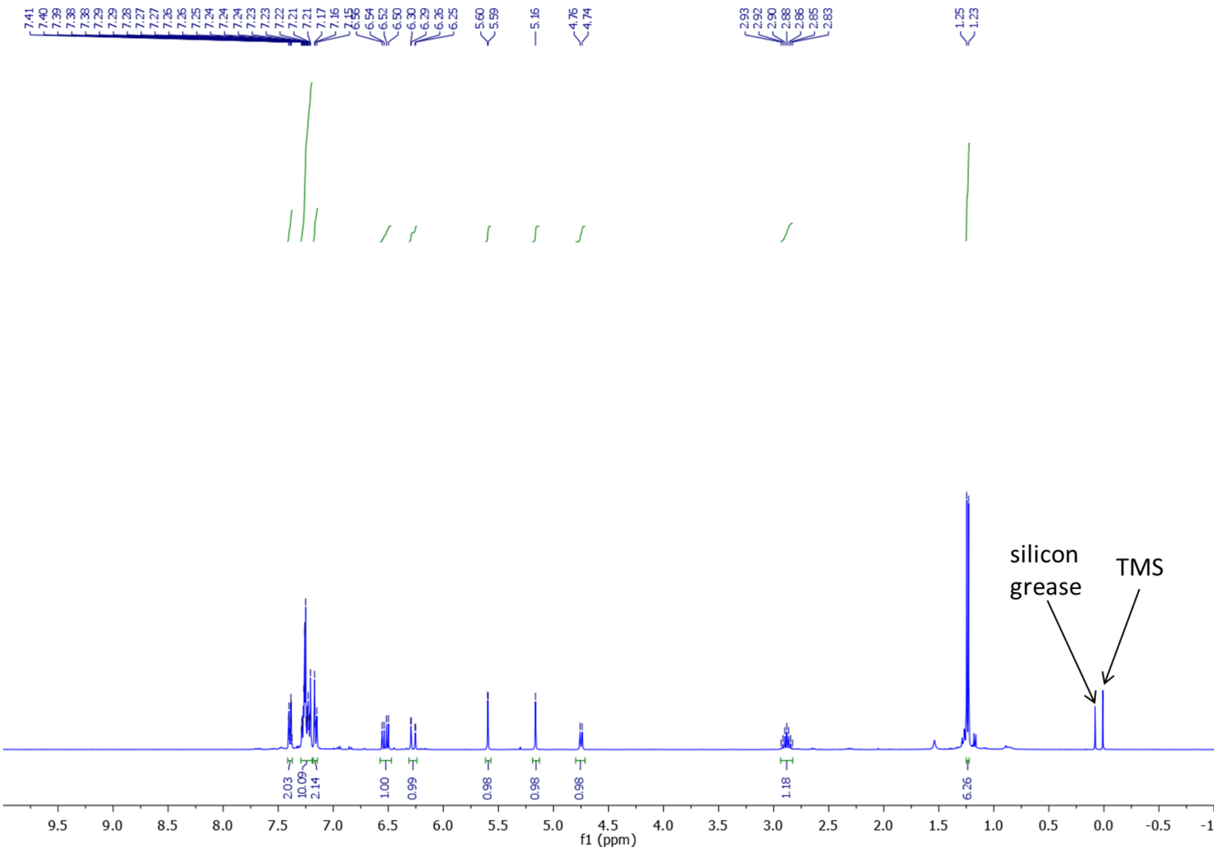

Figure SI 102: ^{1}H NMR spectrum of (*E*)-1-(*para*-chlorophenyl)-3-(*para*-*iso*-propylphenyl)-4-phenyl-penta-1,4-diene **7e** (400 MHz, chloroform-*d*).

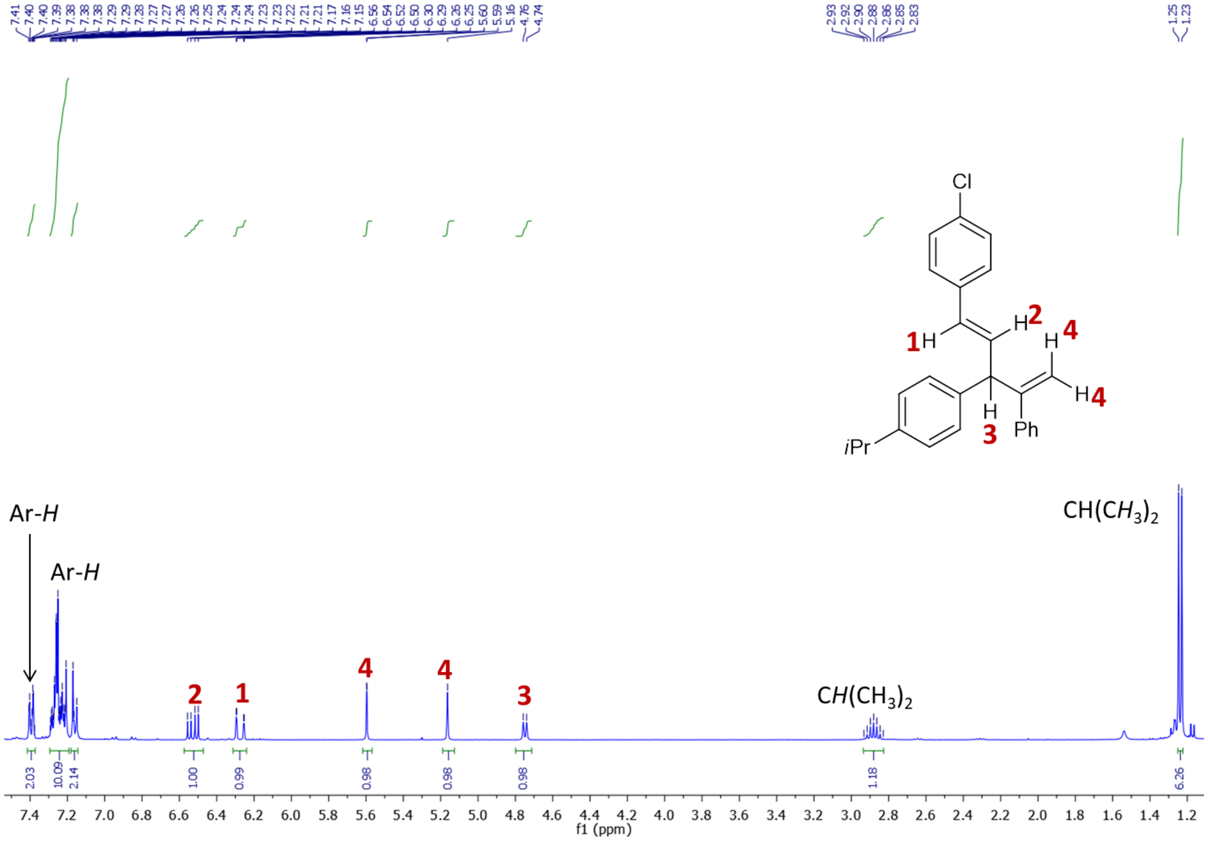

Figure SI 103: Excerpt of ^{1}H NMR spectrum of (*E*)-1-(*para*-chlorophenyl)-3-(*para*-*iso*-propylphenyl)-4-phenyl-penta-1,4-diene **7e** (400 MHz, chloroform-*d*).

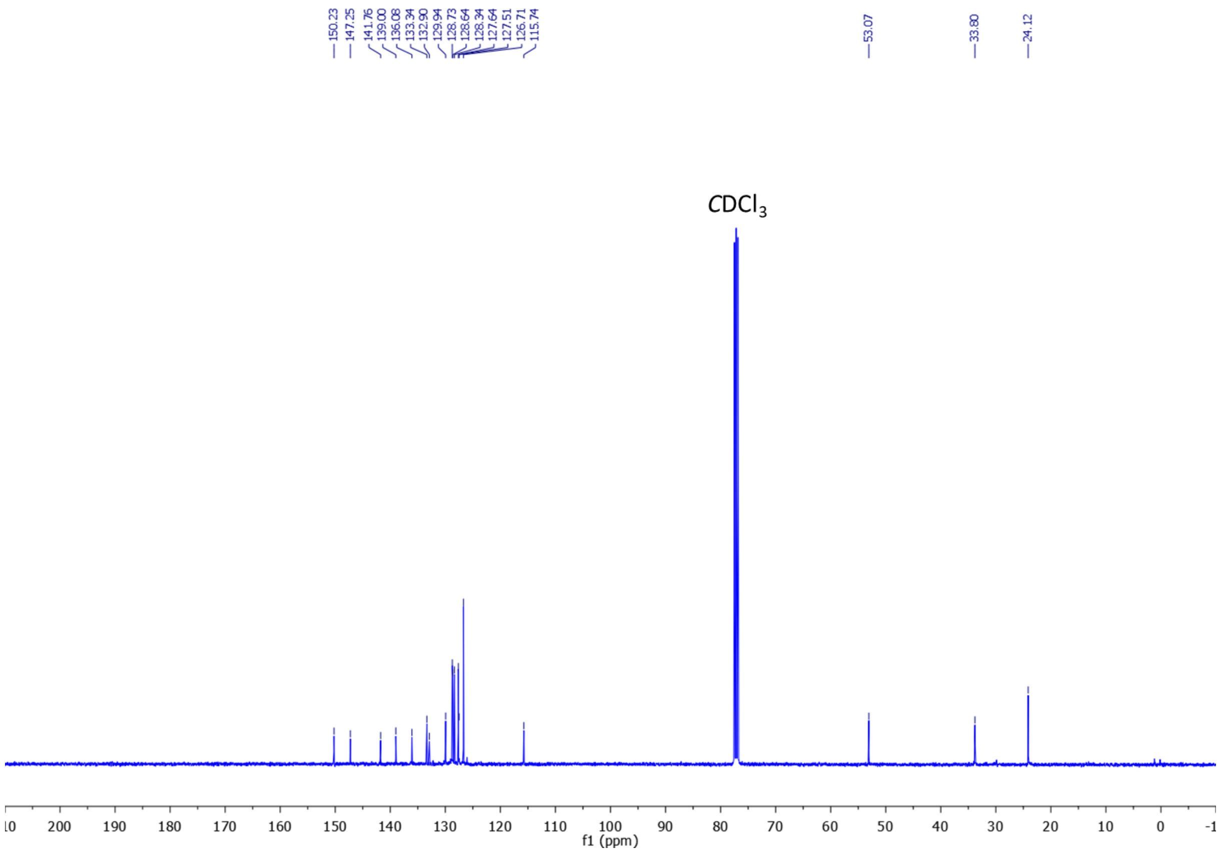

Figure SI 104: ^{13}C NMR spectrum of (*E*)-1-(*para*-chlorophenyl)-3-(*para*-*iso*-propylphenyl)-4-phenyl-penta-1,4-diene **7e** (101 MHz, chloroform-*d*).

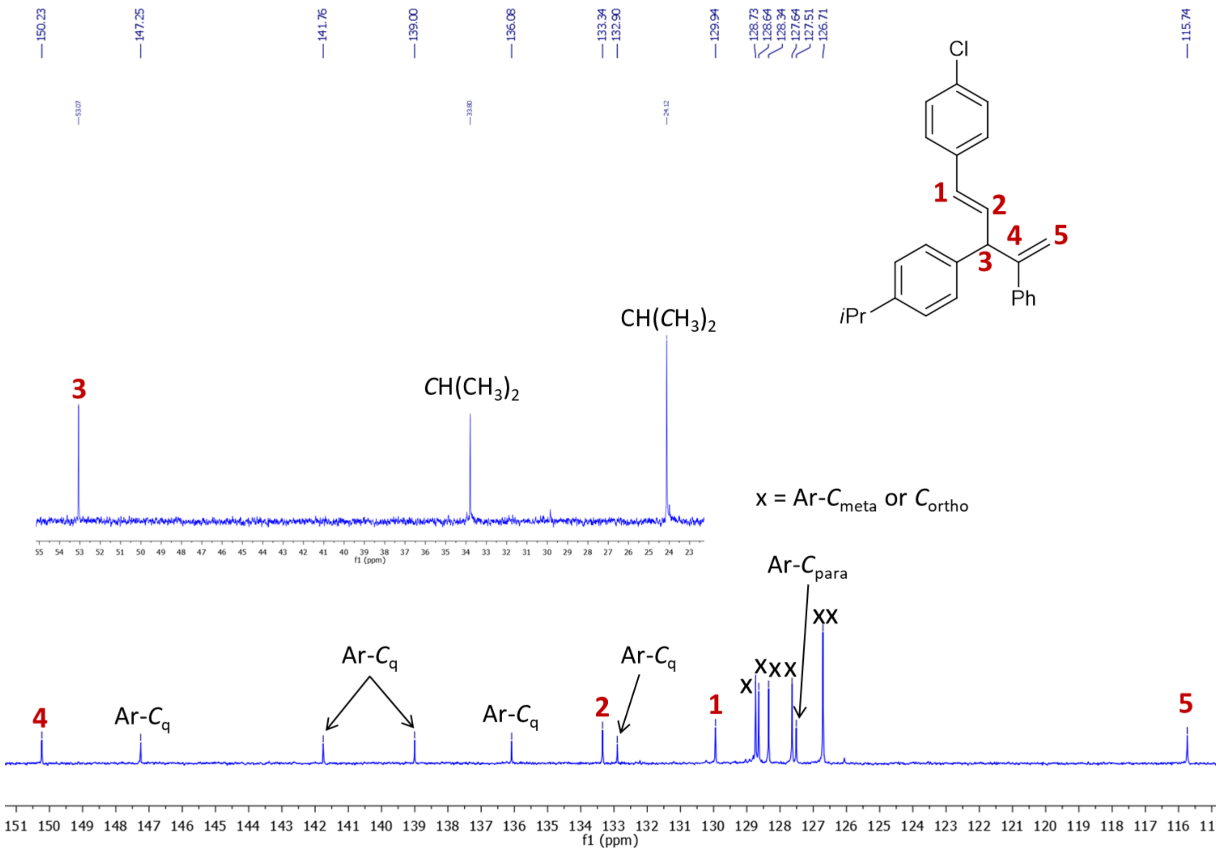

Figure SI 105: Excerpt of the ^{13}C NMR spectrum of (*E*)-1-(*para*-chlorophenyl)-3-(*para*-*iso*-propylphenyl)-4-phenyl-penta-1,4-diene **7e** (101 MHz, chloroform-*d*).

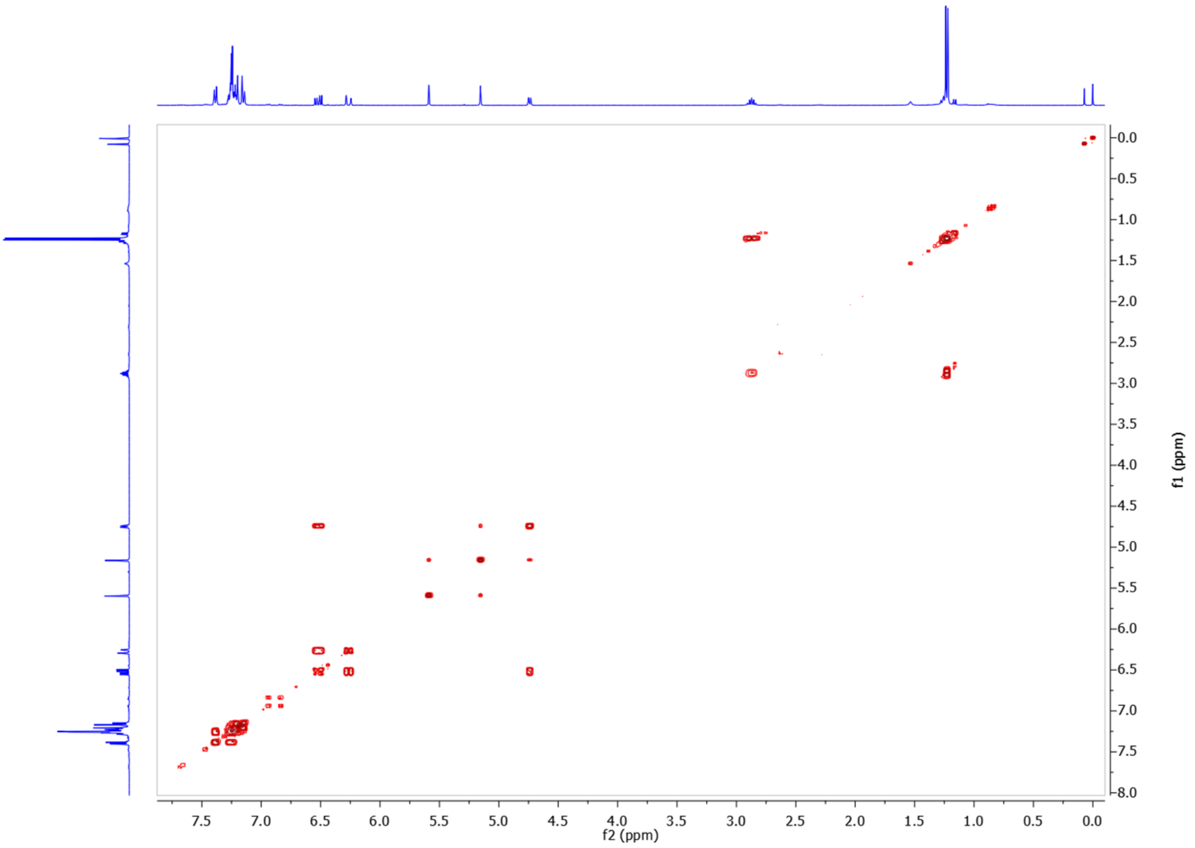

Figure SI 106: HH COSY NMR spectrum of (*E*)-1-(*para*-chlorophenyl)-3-(*para*-*iso*-propylphenyl)-4-phenyl-penta-1,4-diene **7e** (400 MHz, chloroform-*d*).

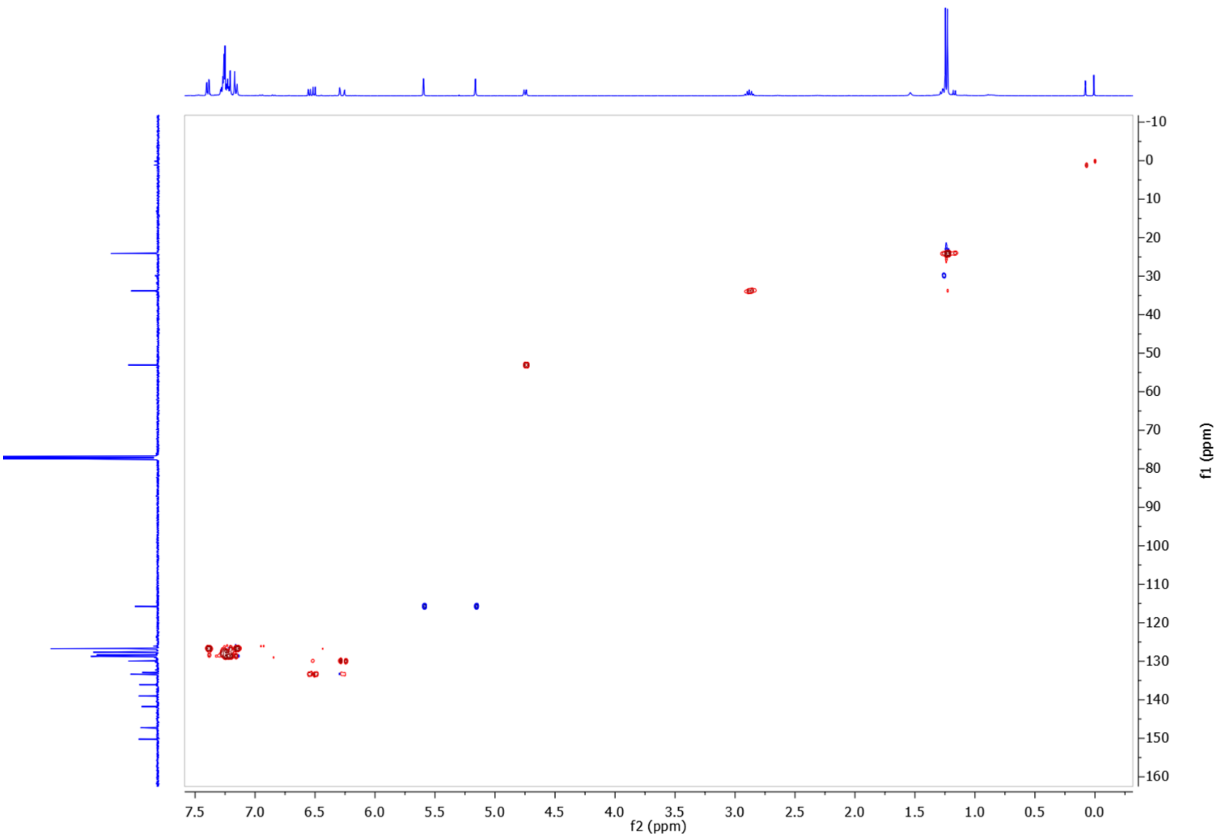

Figure SI 107: HSQC NMR spectrum of (*E*)-1-(*para*-chlorophenyl)-3-(*para*-*iso*-propylphenyl)-4-phenyl-penta-1,4-diene **7e** (101 MHz, chloroform-*d*).

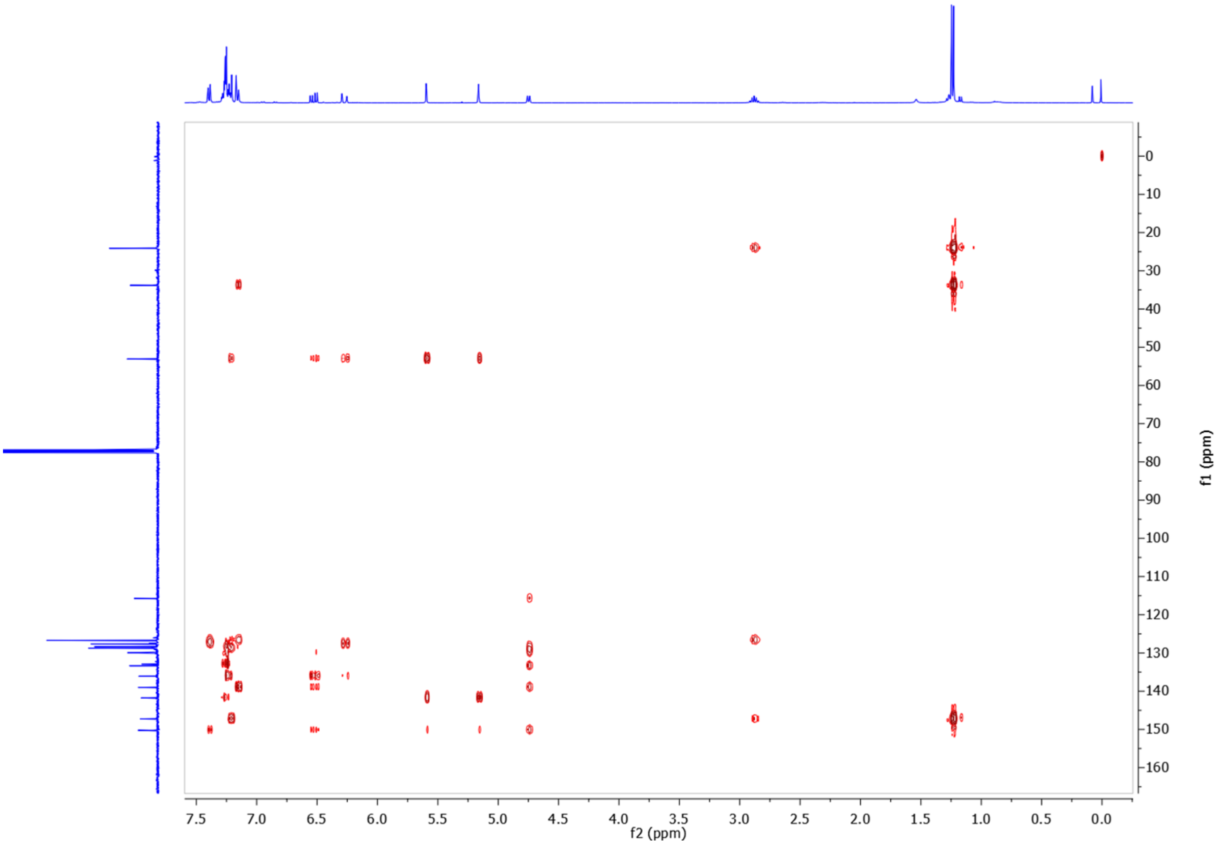

Figure SI 108: HMBC NMR spectrum of (*E*)-1-(*para*-chlorophenyl)-3-(*para*-*iso*-propylphenyl)-4-phenyl-penta-1,4-diene **7e** (101 MHz, chloroform-*d*).

9.3.12 1,4-diene 7f

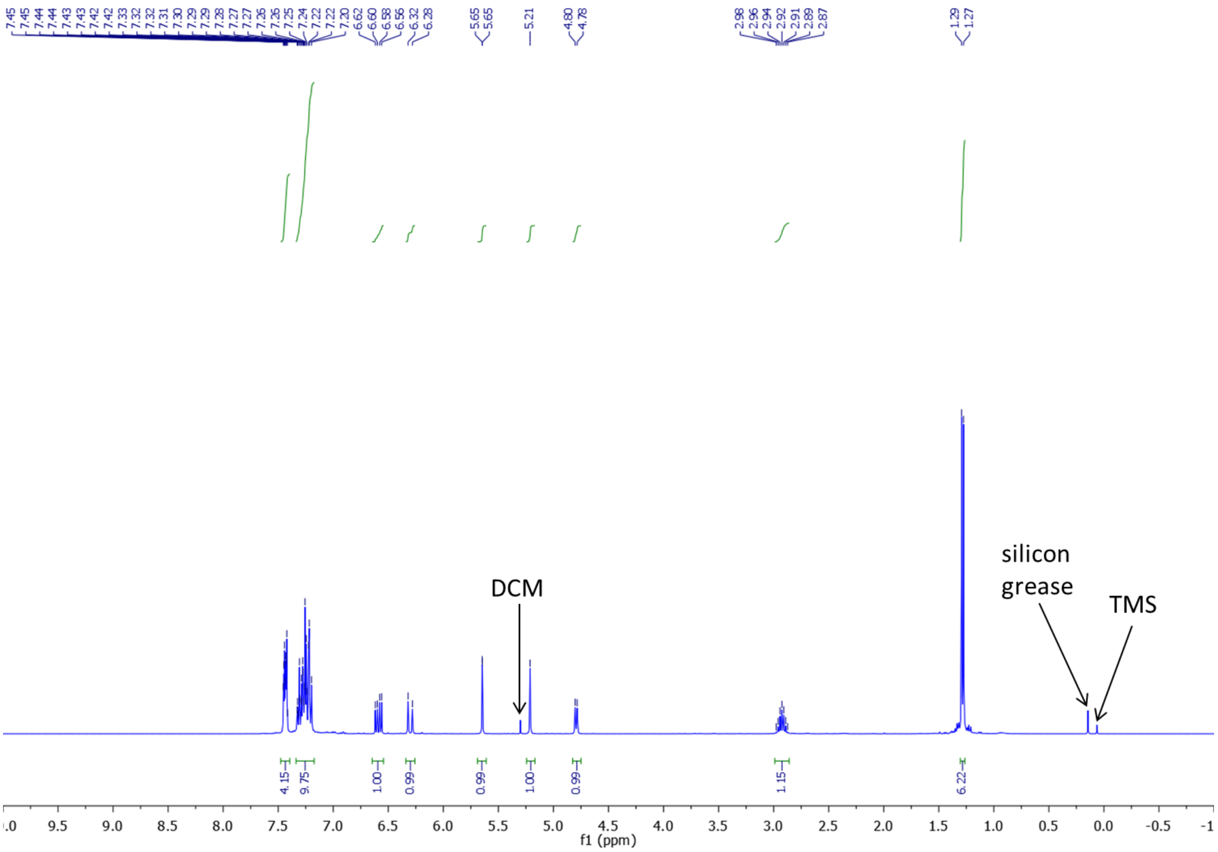

Figure SI 109: ^{1}H NMR spectrum of (*E*)-1-(*para*-bromophenyl)-3-(*para*-*iso*-propylphenyl)-4-phenyl-penta-1,4-diene **7f** (400 MHz, chloroform-*d*).

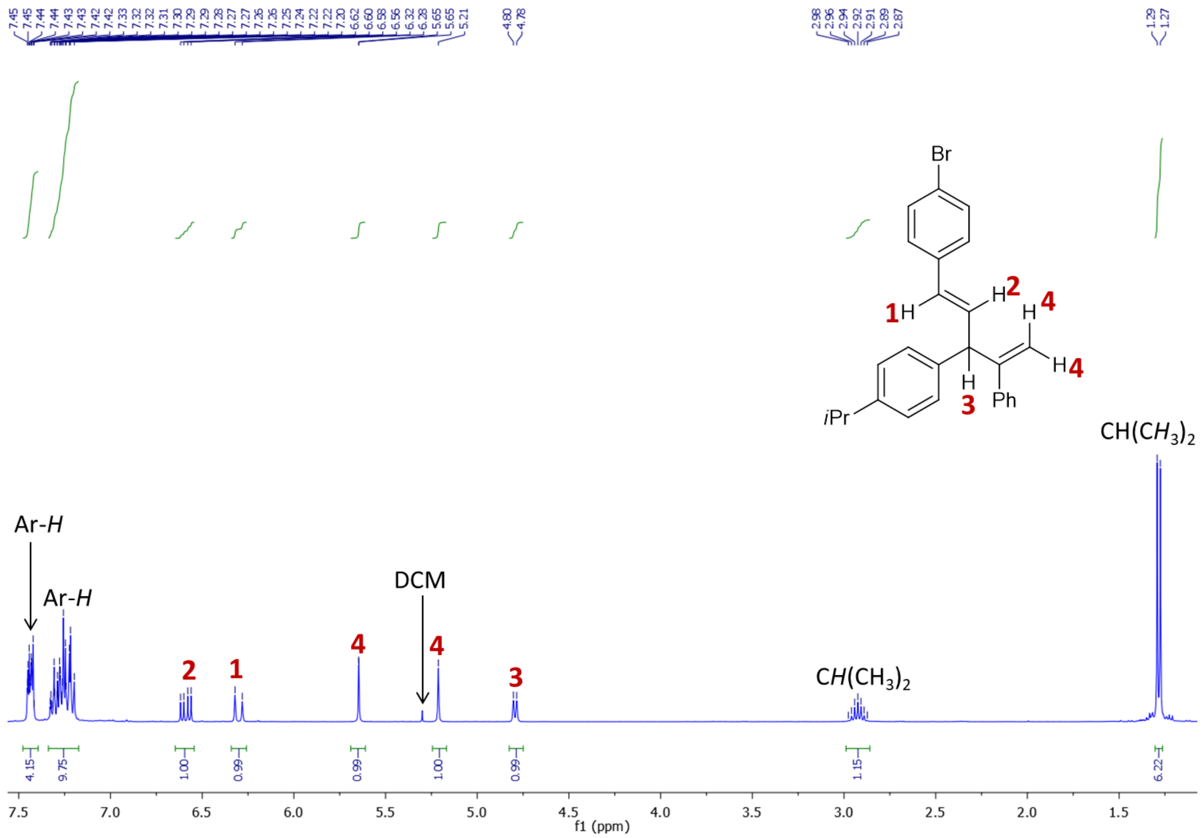

Figure SI 110: Excerpt of ^{1}H NMR spectrum of (*E*)-1-(*para*-bromophenyl)-3-(*para*-*iso*-propylphenyl)-4-phenyl-penta-1,4-diene **7f** (400 MHz, chloroform-*d*).

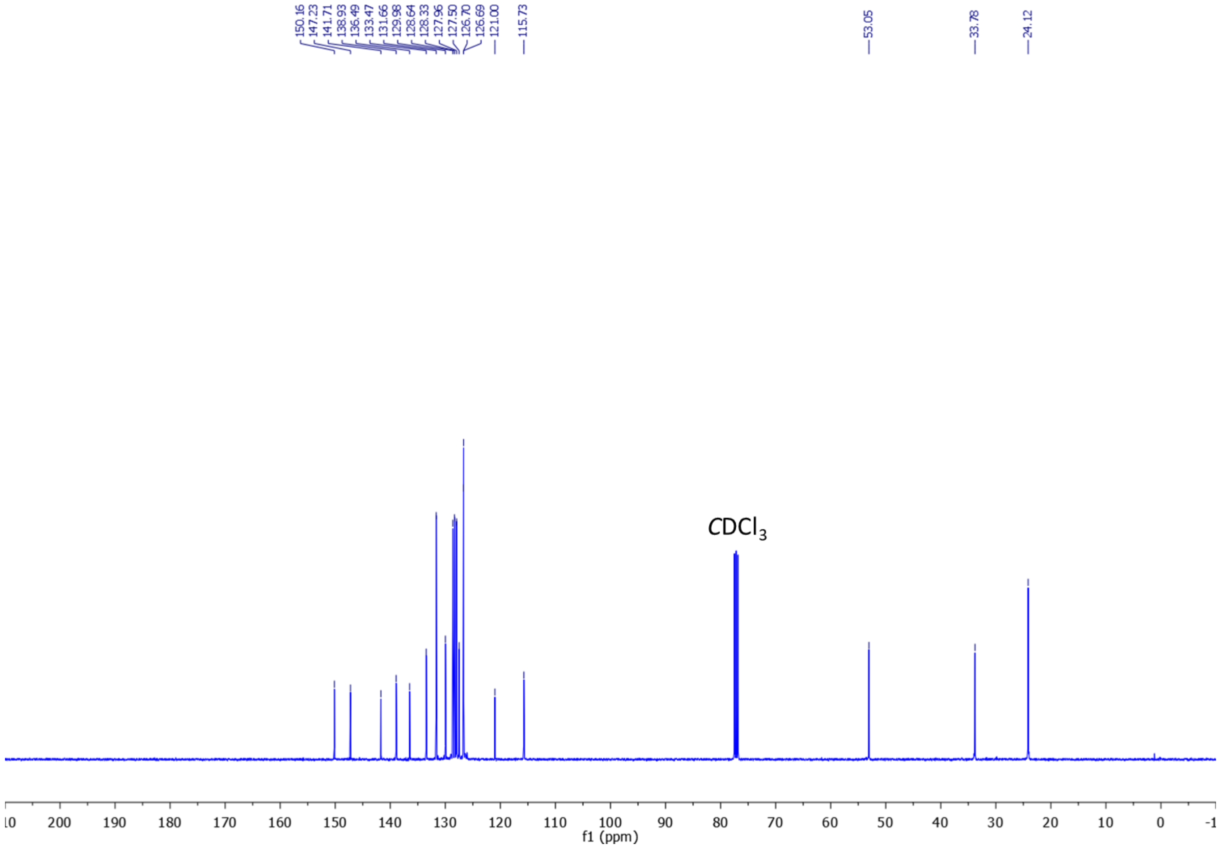

Figure SI 111: ^{13}C NMR spectrum of (*E*)-1-(*para*-bromophenyl)-3-(*para*-*iso*-propylphenyl)-4-phenyl-penta-1,4-diene **7f** (101 MHz, chloroform-*d*).

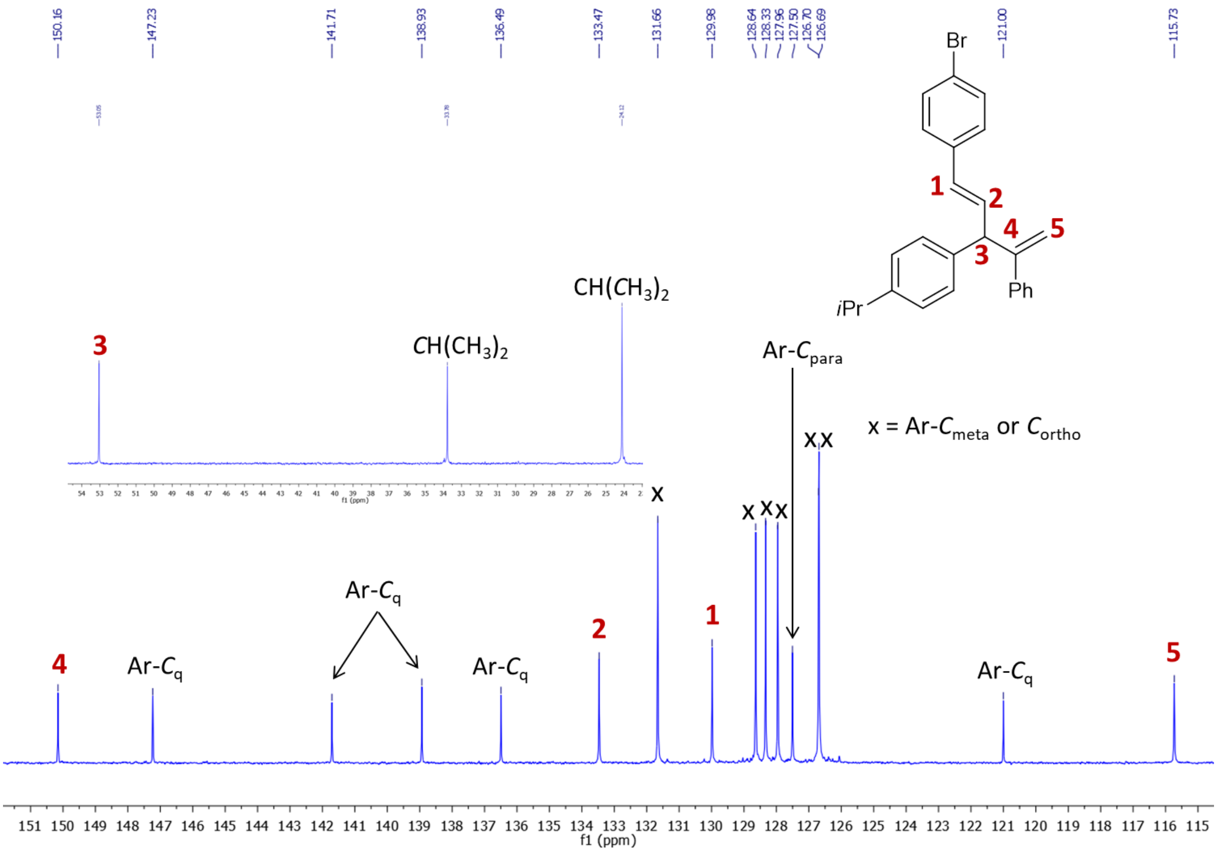

Figure SI 112: Excerpt of the ^{13}C NMR spectrum of (*E*)-1-(*para*-bromophenyl)-3-(*para*-*iso*-propylphenyl)-4-phenyl-penta-1,4-diene **7f** (101 MHz, chloroform-*d*).

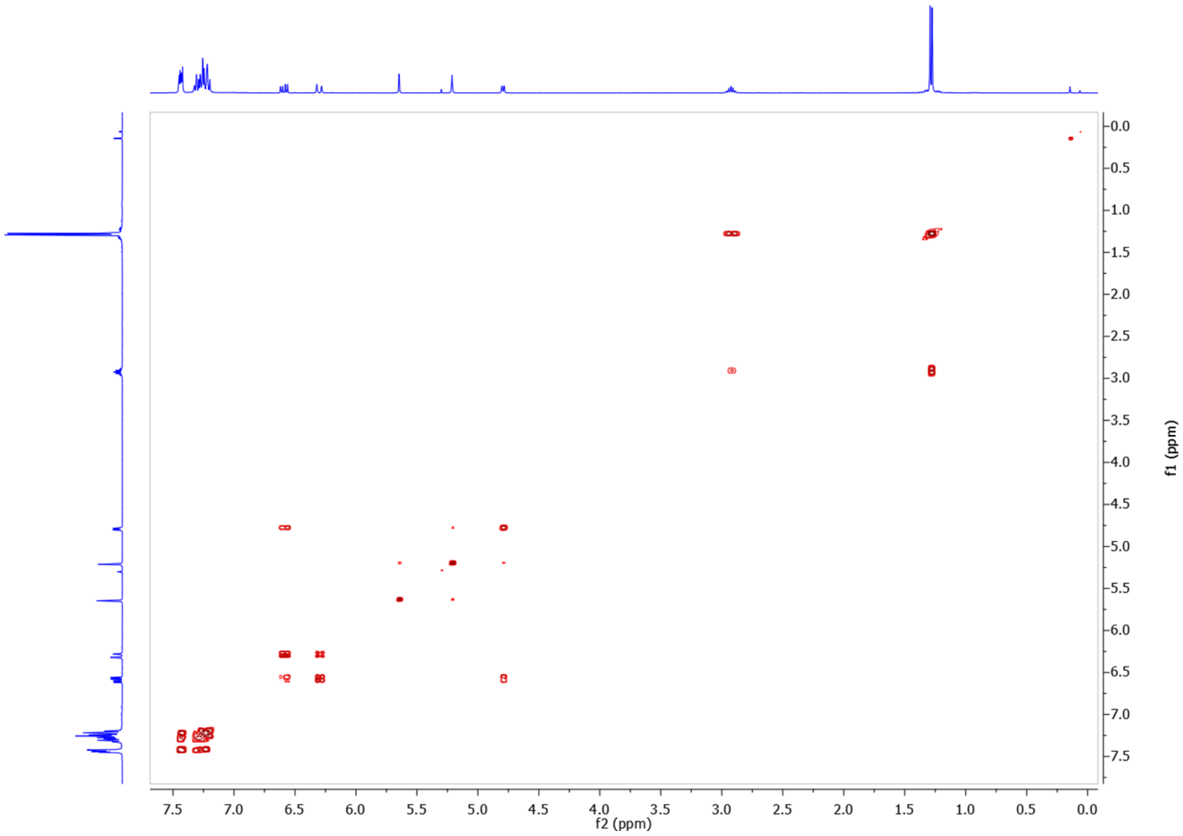

Figure SI 113: HH COSY NMR spectrum of (*E*)-1-(*para*-bromophenyl)-3-(*para*-*iso*-propylphenyl)-4-phenyl-penta-1,4-diene **7f** (400 MHz, chloroform-*d*).

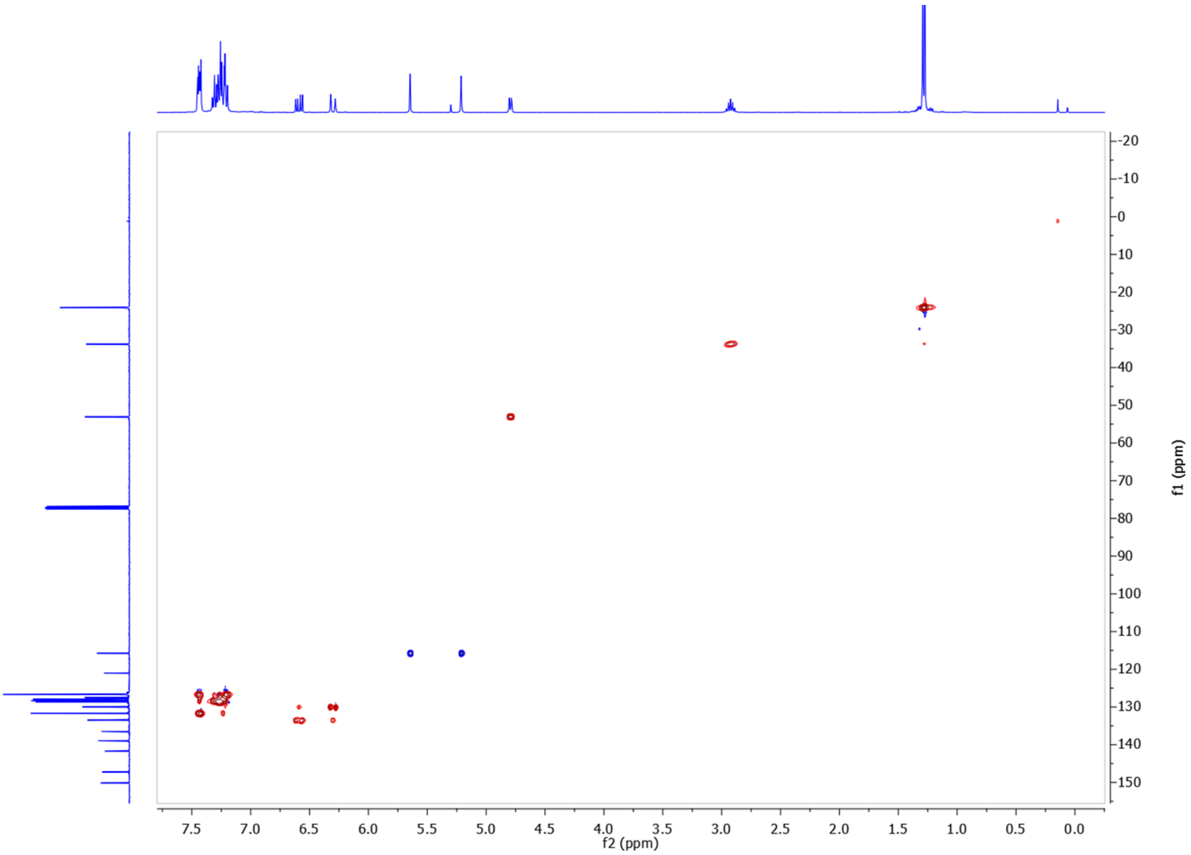

Figure SI 114: HSQC NMR spectrum of (*E*)-1-(*para*-bromophenyl)-3-(*para*-*iso*-propylphenyl)-4-phenyl-penta-1,4-diene **7f** (101 MHz, chloroform-*d*).

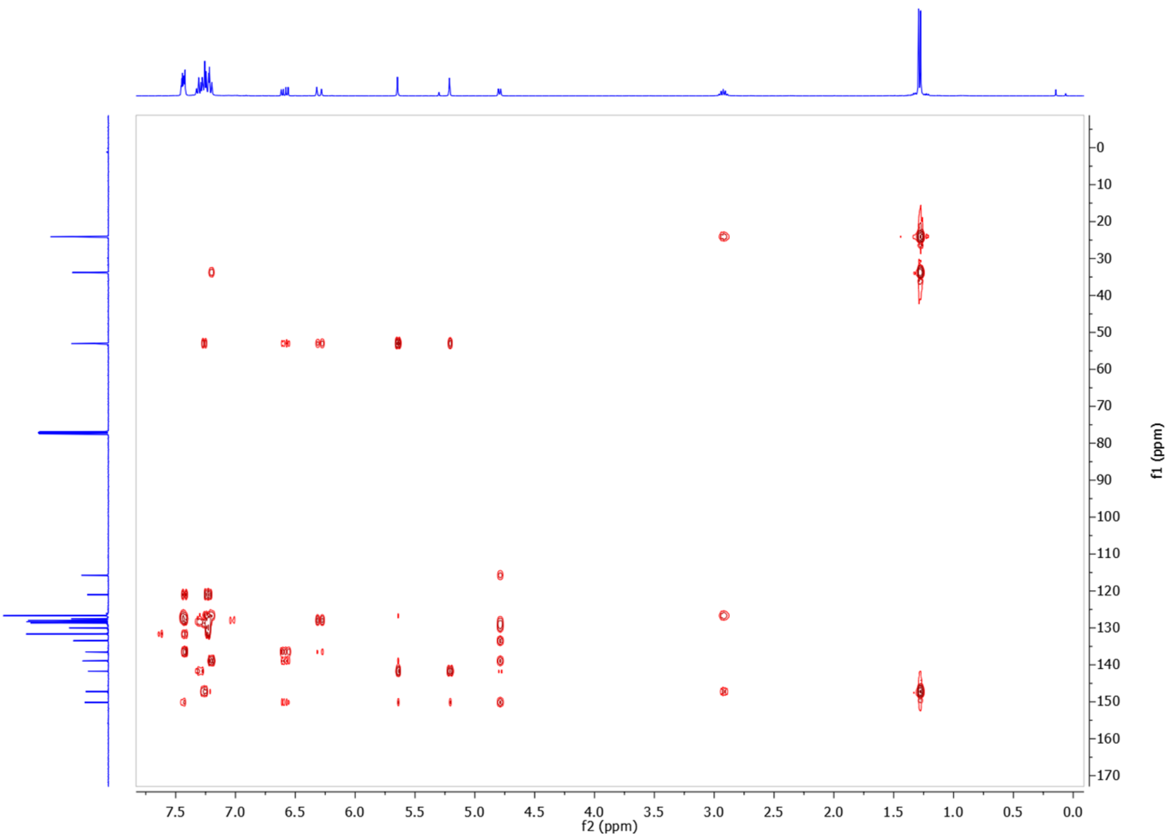

Figure SI 115: HMBC NMR spectrum of (*E*)-1-(*para*-bromophenyl)-3-(*para*-*iso*-propylphenyl)-4-phenyl-penta-1,4-diene **7f** (101 MHz, chloroform-*d*).

9.3.13 1,4-diene 7g

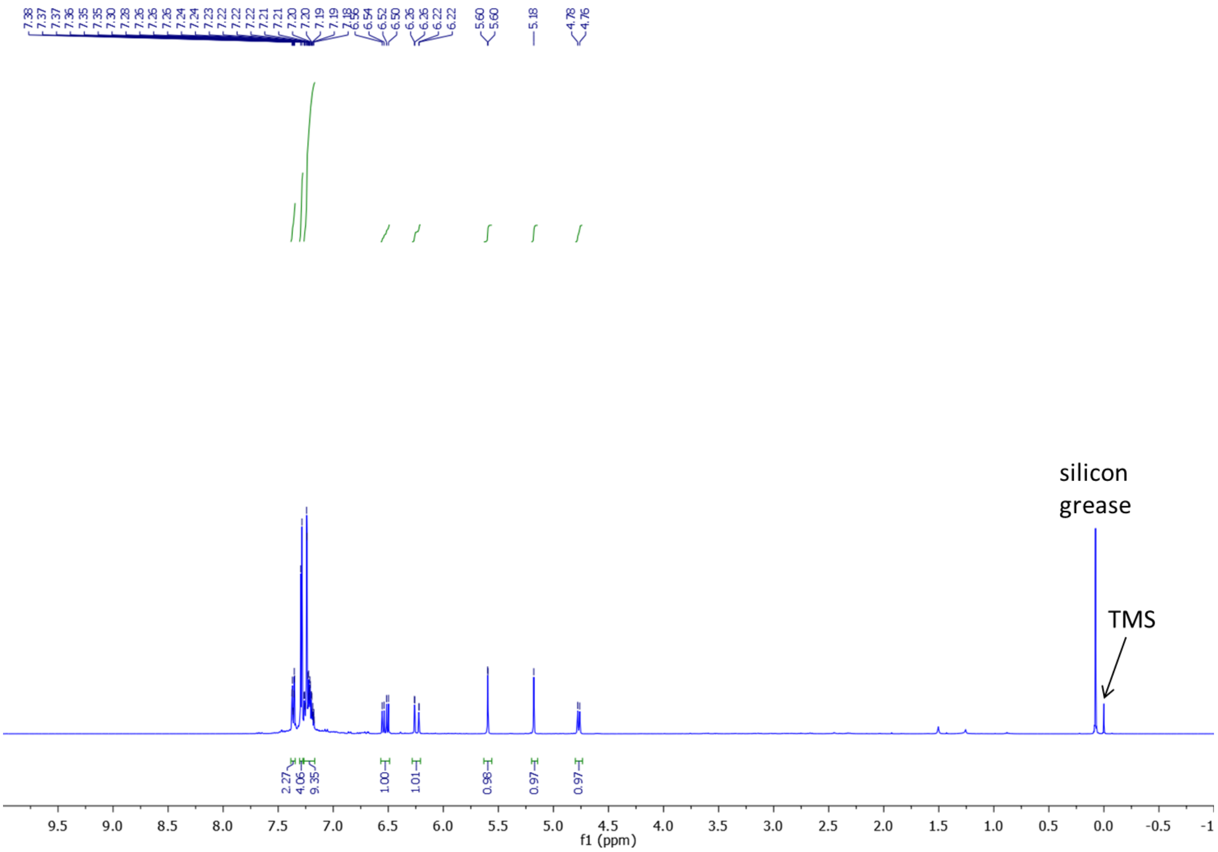

Figure SI 116: ^{1}H NMR spectrum of (*E*)-1-(*para*-chlorophenyl)-3,4-diphenyl-penta-1,4-diene **7g** (400 MHz, chloroform-*d*).

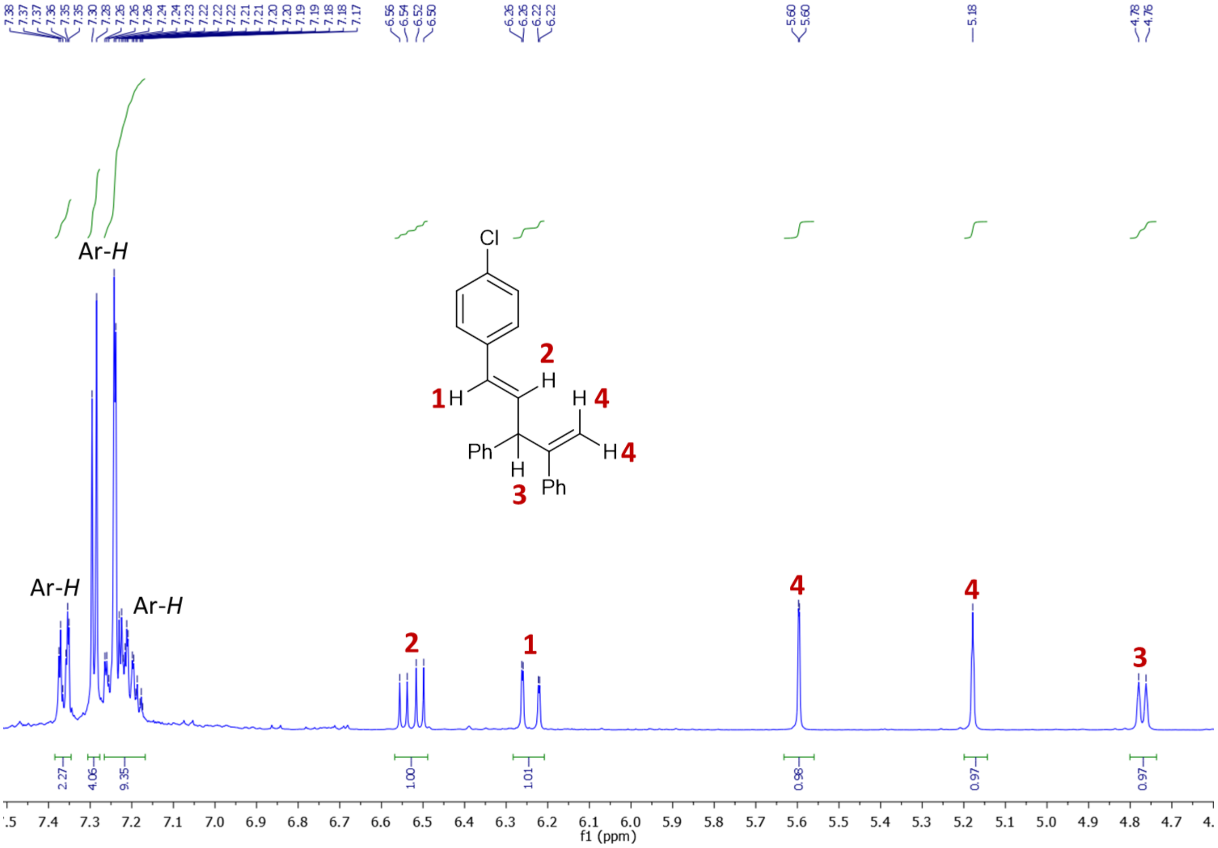

Figure SI 117: Excerpt of ^{1}H NMR spectrum of (*E*)-1-(*para*-chlorophenyl)-3,4-diphenyl-penta-1,4-diene **7g** (400 MHz, chloroform-*d*).

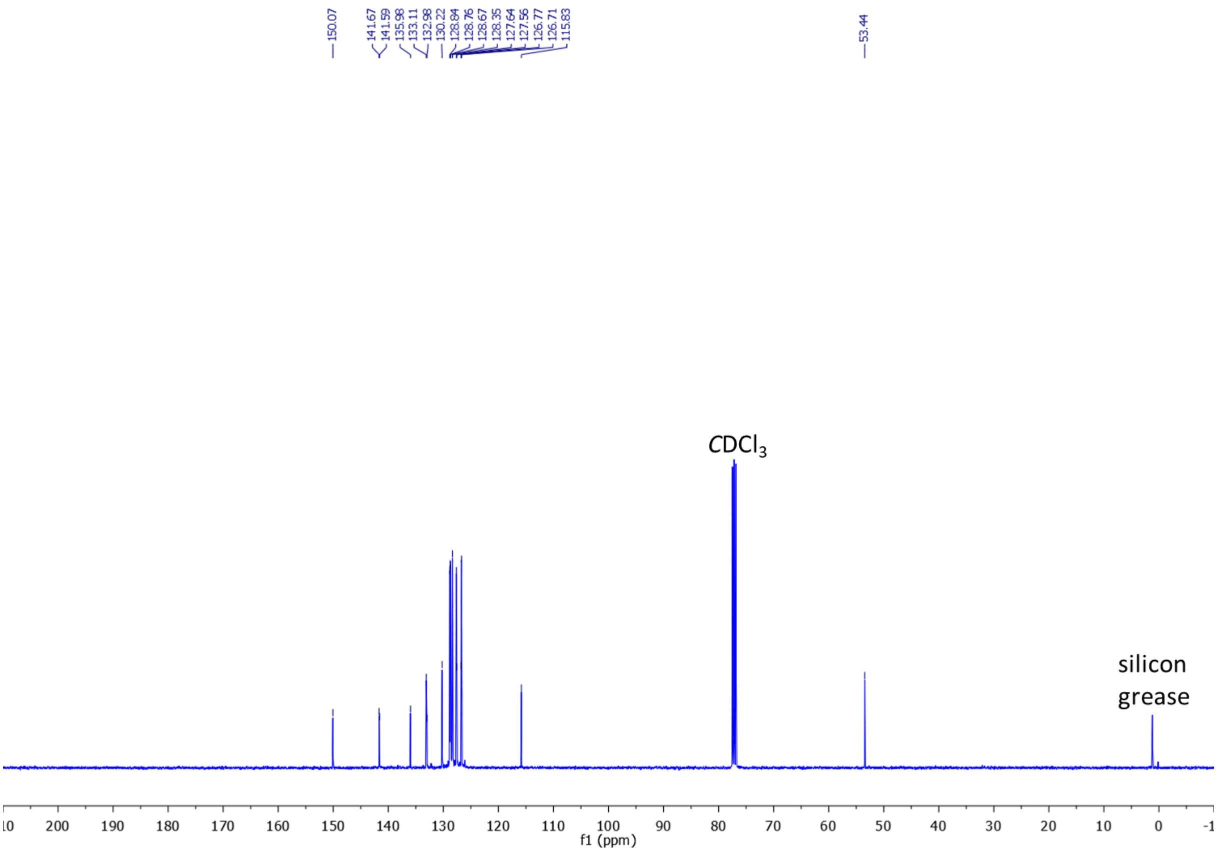

Figure SI 118: ^{13}C NMR spectrum of (*E*)-1-(*para*-chlorophenyl)-3,4-diphenyl-penta-1,4-diene **7g** (101 MHz, chloroform-*d*).

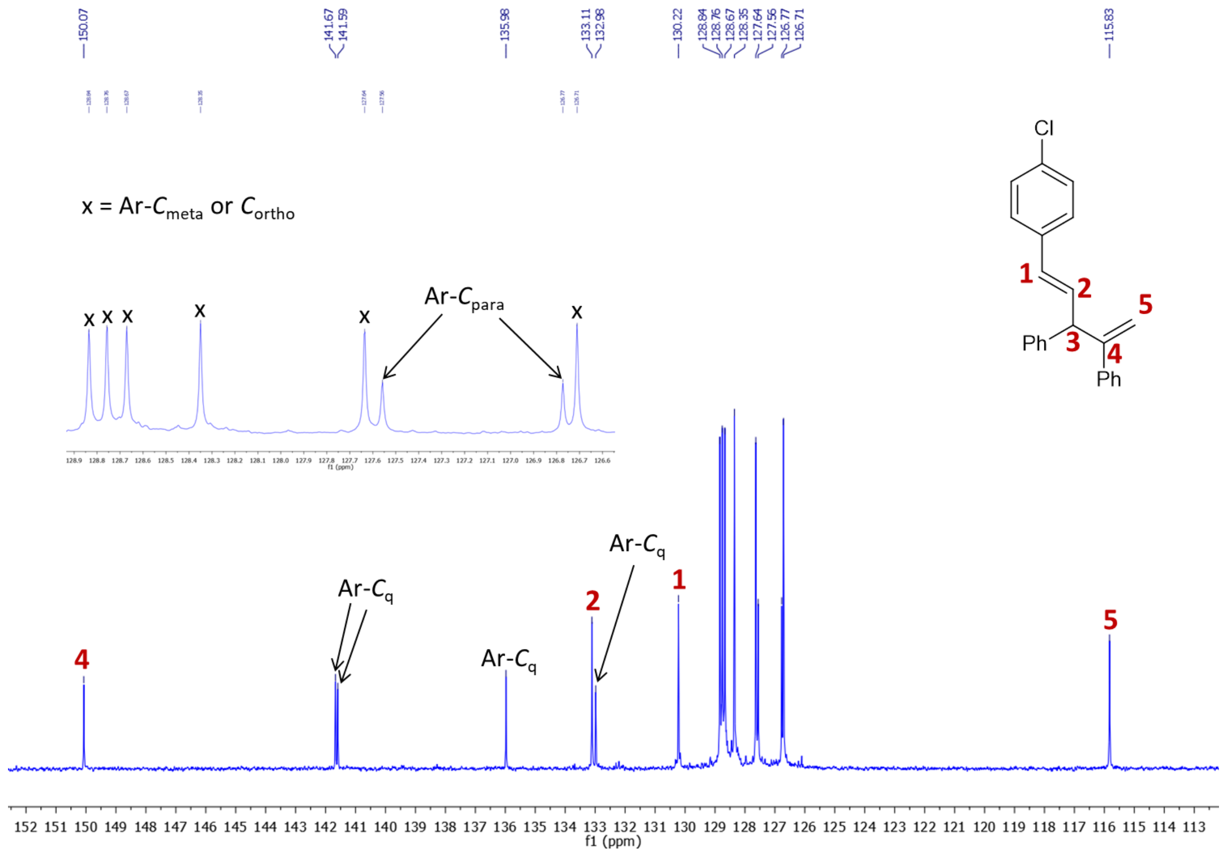

Figure SI 119: Excerpt of the ^{13}C NMR spectrum of (*E*)-1-(*para*-chlorophenyl)-3,4-diphenyl-penta-1,4-diene **7g** (101 MHz, chloroform-*d*).

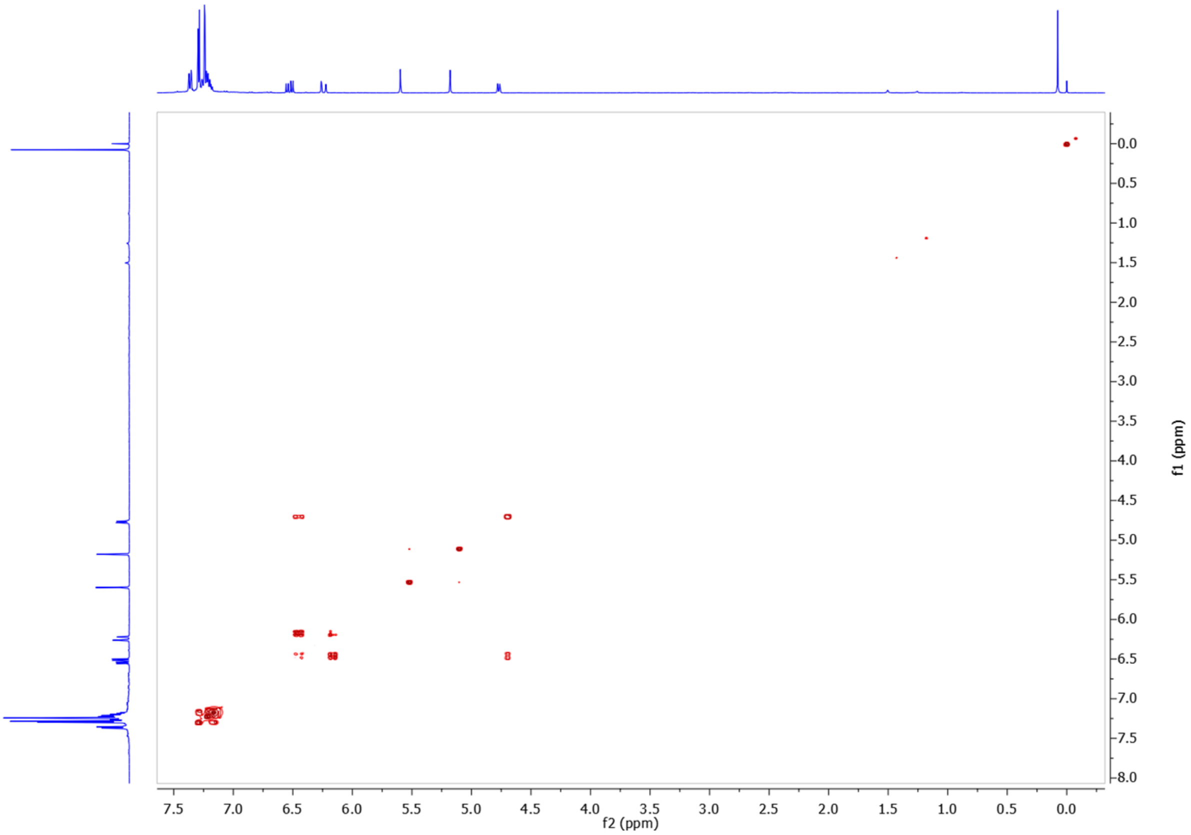

Figure SI 120: HH COSY NMR spectrum of (*E*)-1-(*para*-chlorophenyl)-3,4-diphenyl-penta-1,4-diene **7g** (400 MHz, chloroform-*d*).

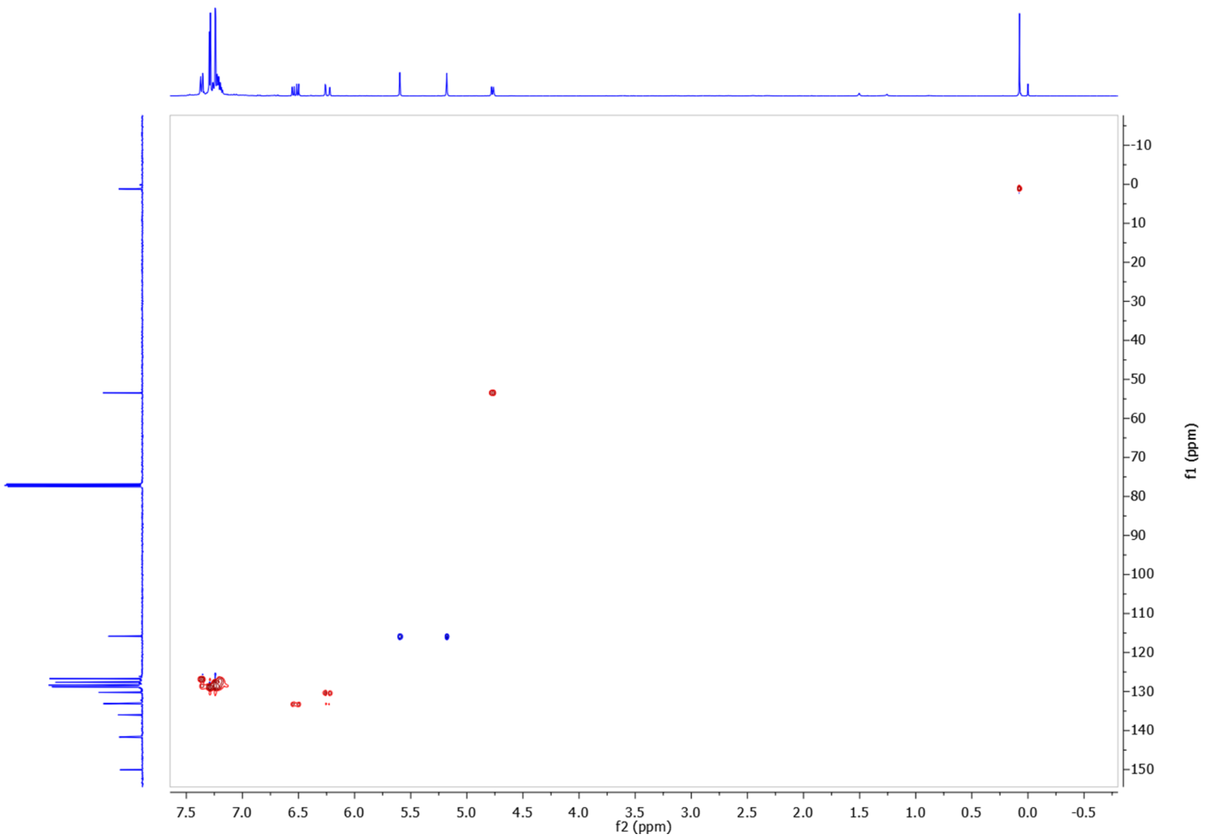

Figure SI 121: HSQC NMR spectrum of (*E*)-1-(*para*-chlorophenyl)-3,4-diphenyl-penta-1,4-diene **7g** (101 MHz, chloroform-*d*).

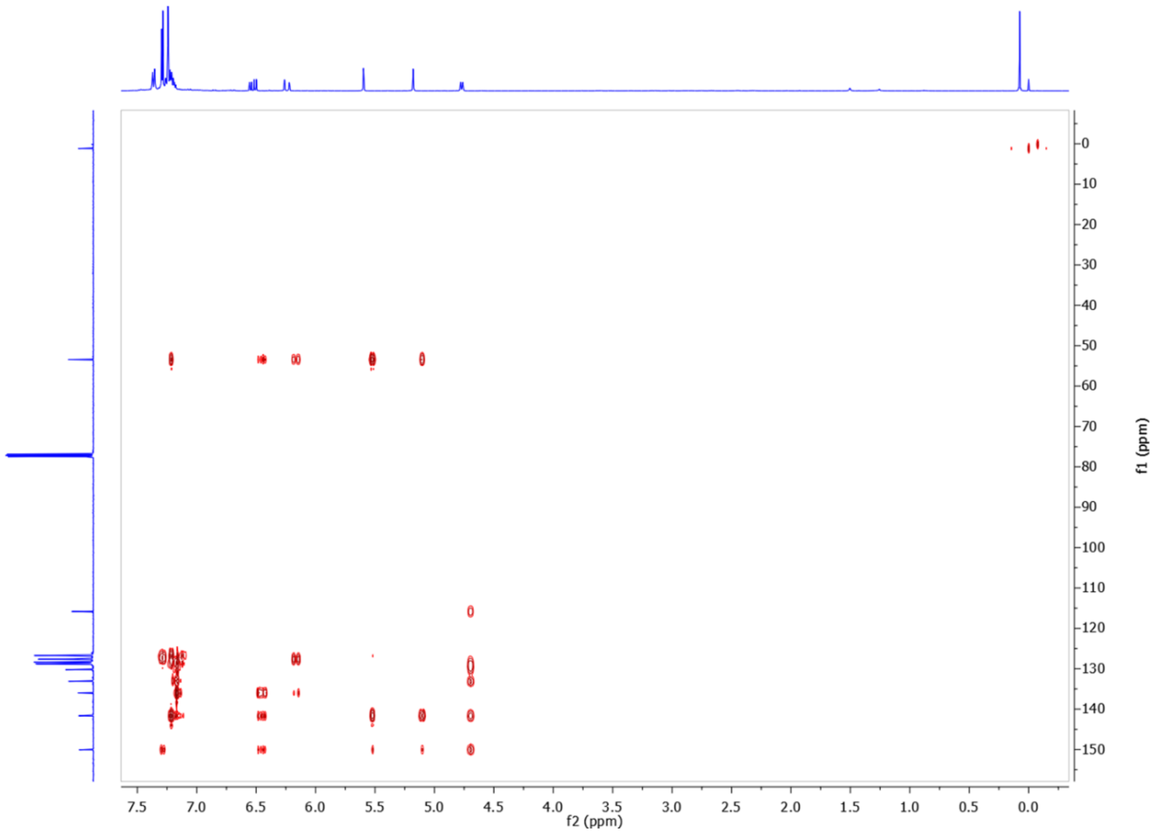

Figure SI 122: HMBC NMR spectrum of (*E*)-1-(*para*-chlorophenyl)-3,4-diphenyl-penta-1,4-diene **7g** (101 MHz, chloroform-*d*).

9.3.14 1,4-diene 7h

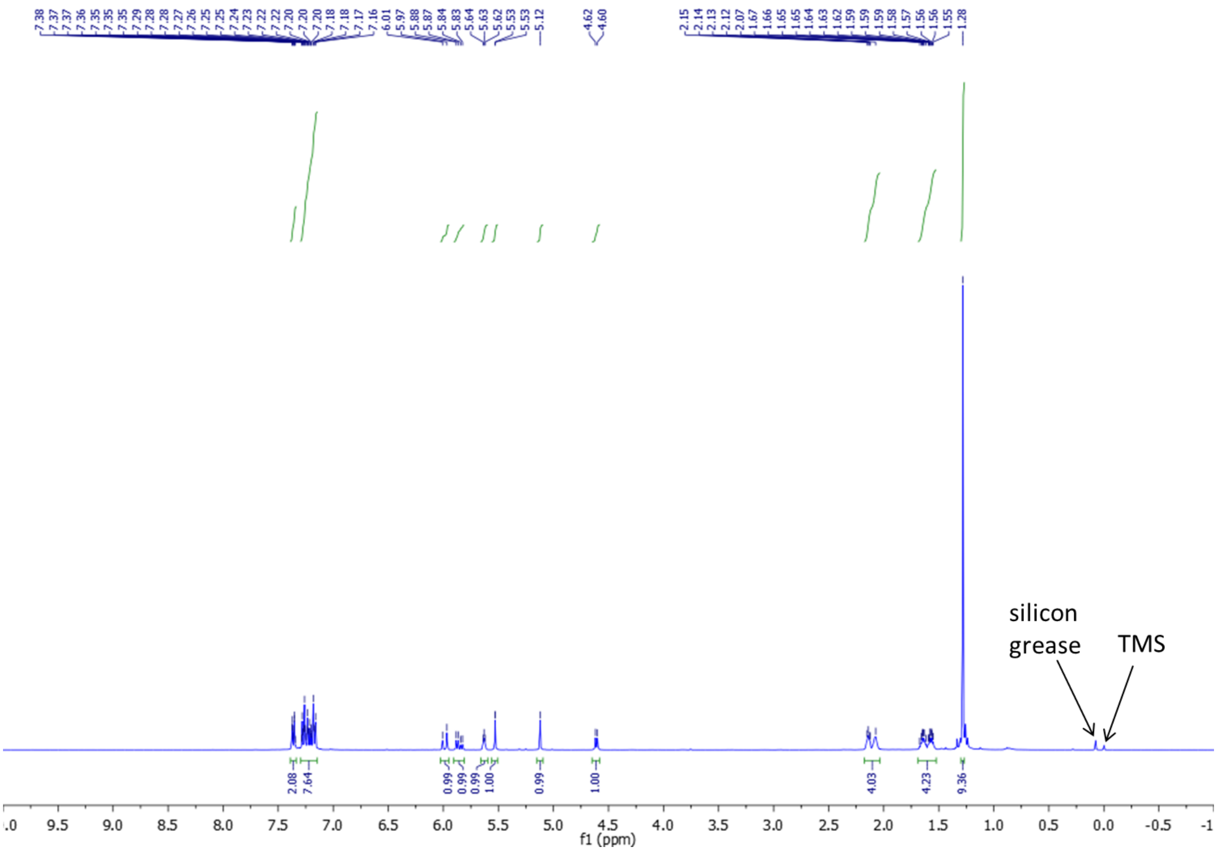

Figure SI 123: ^{1}H NMR spectrum of (*E*)-1-(cyclohex-1-en-1-yl)-3-(*para*-*tert*-butylphenyl)-4-phenyl-penta-1,4-diene **7h** (400 MHz, chloroform-*d*).

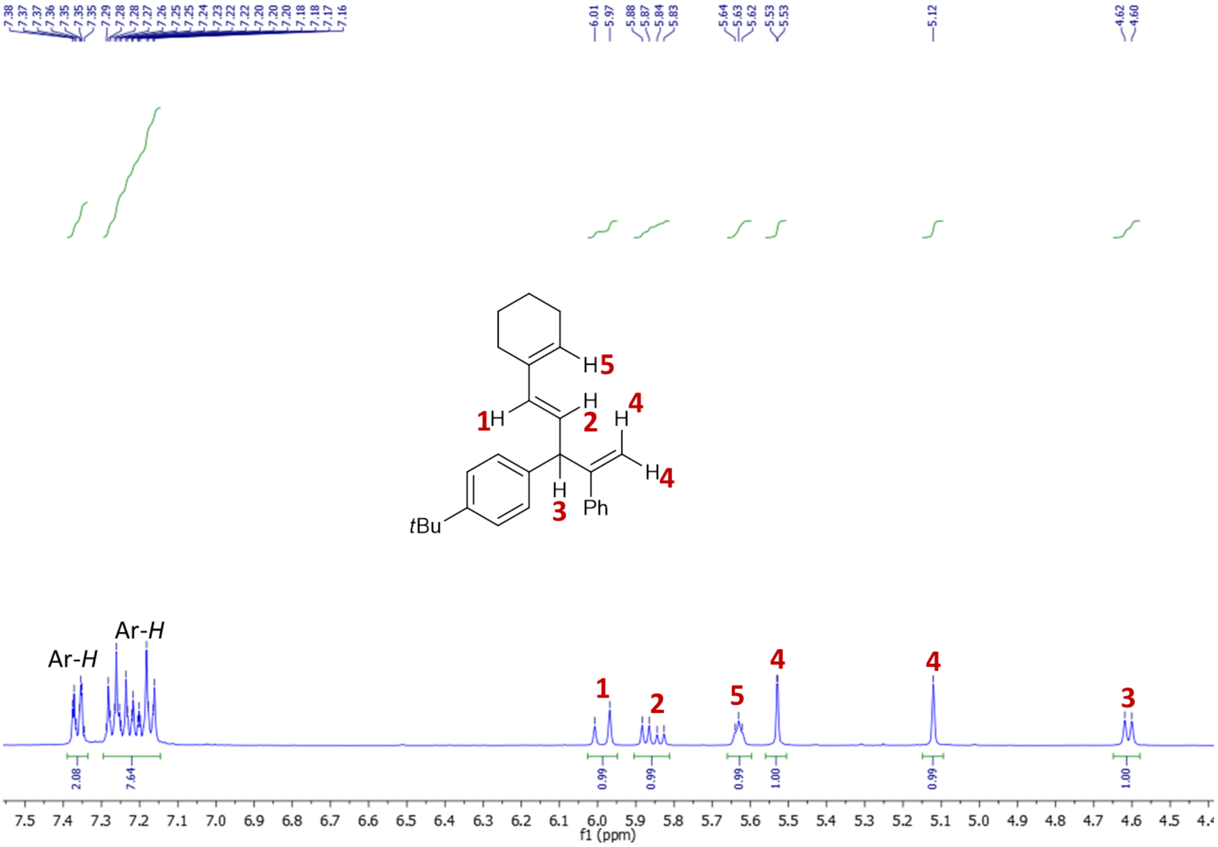

Figure SI 124: Excerpt of ^{1}H NMR spectrum of (*E*)-1-(cyclohex-1-en-1-yl)-3-(*para*-*tert*-butylphenyl)-4-phenyl-penta-1,4-diene **7h** (400 MHz, chloroform-*d*).

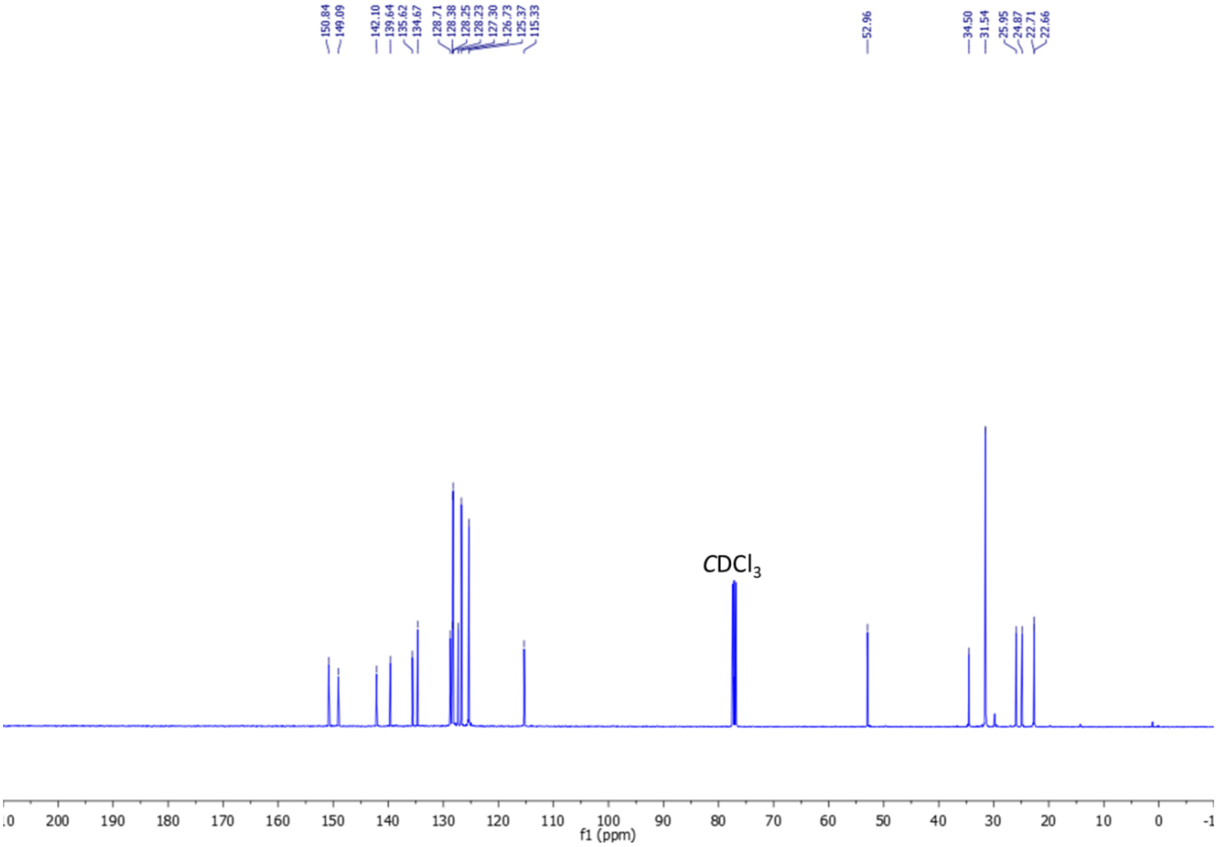

Figure SI 125: ^{13}C NMR spectrum of (*E*)-1-(cyclohex-1-en-1-yl)-3-(*para*-*tert*-butylphenyl)-4-phenyl-penta-1,4-diene **7h** (101 MHz, chloroform-*d*).

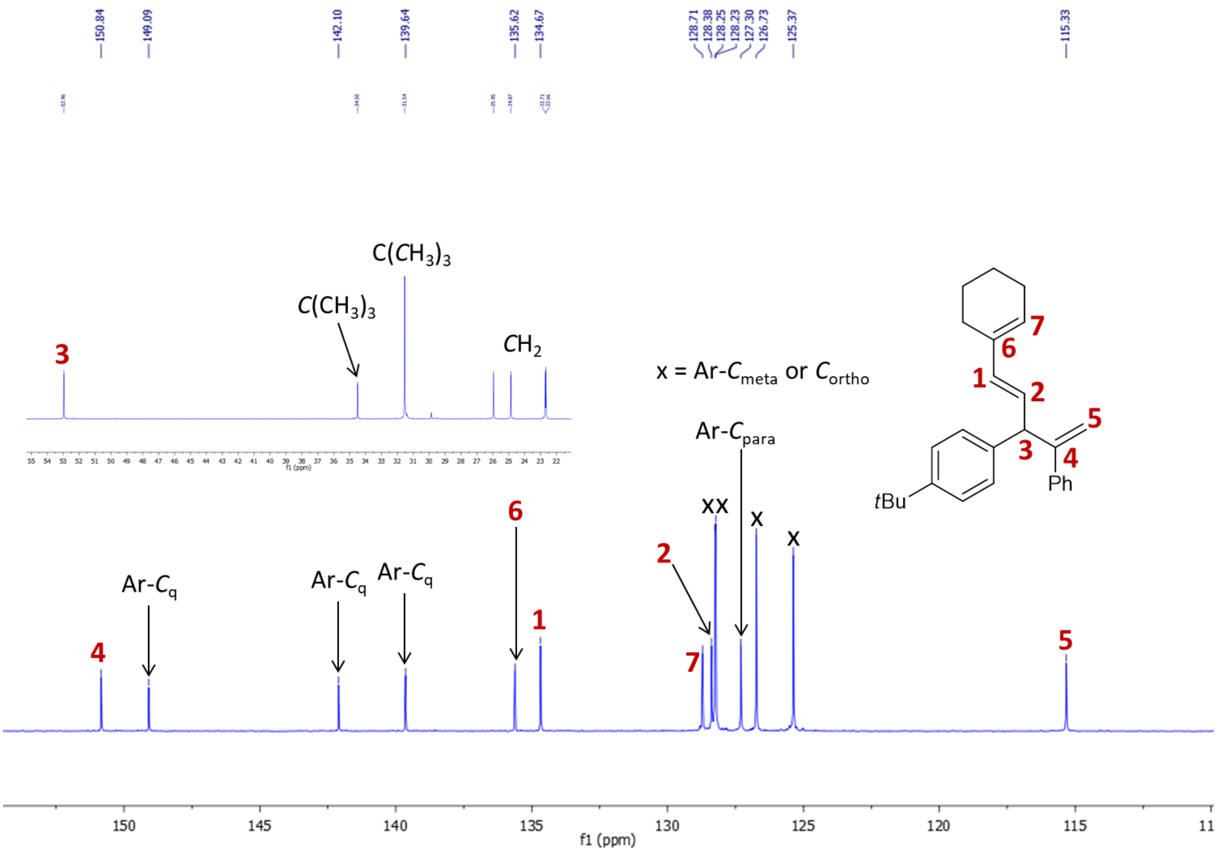

Figure SI 126: Excerpt of the ^{13}C NMR spectrum of (*E*)-1-(cyclohex-1-en-1-yl)-3-(*para*-*tert*-butylphenyl)-4-phenyl-penta-1,4-diene **7h** (101 MHz, chloroform-*d*).

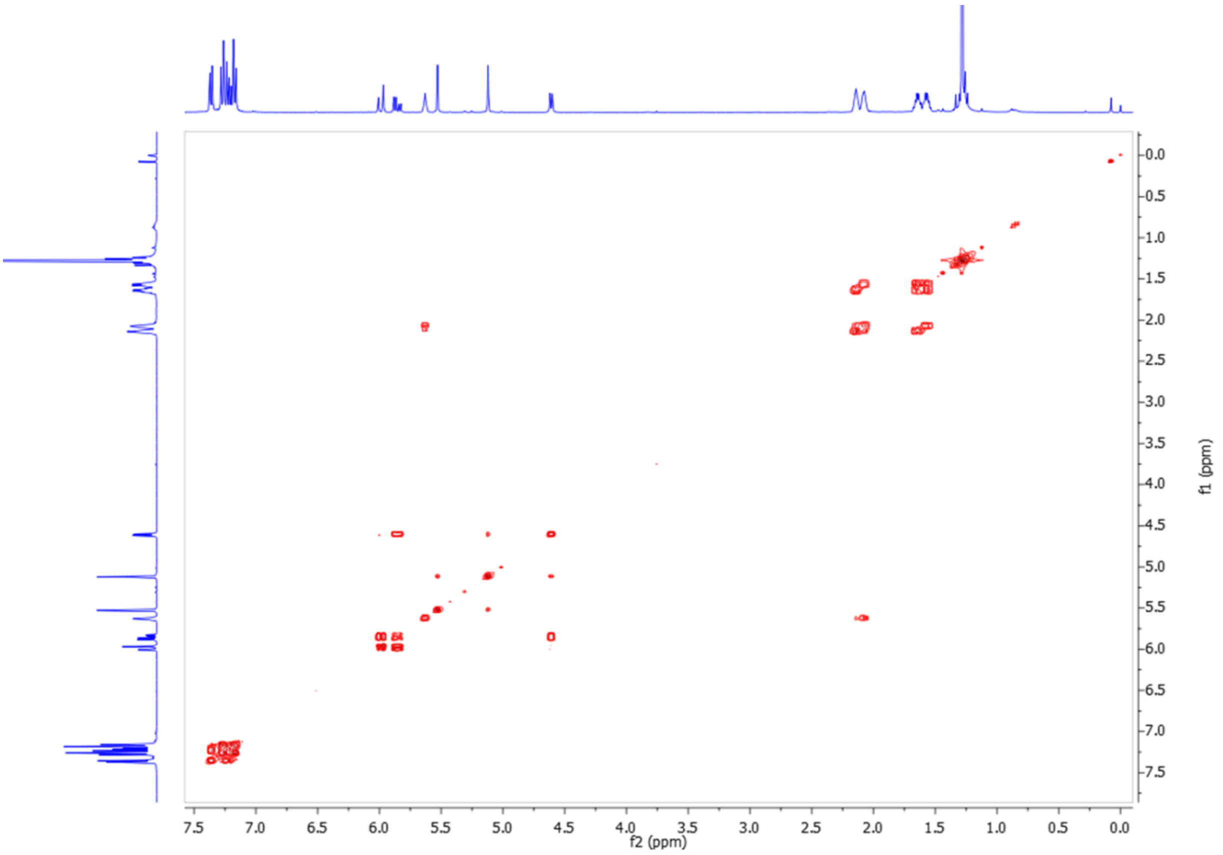

Figure SI 127: HH COSY NMR spectrum of (*E*)-1-(cyclohex-1-en-1-yl)-3-(*para*-*tert*-butylphenyl)-4-phenyl-penta-1,4-diene **7h** (400 MHz, chloroform-*d*).

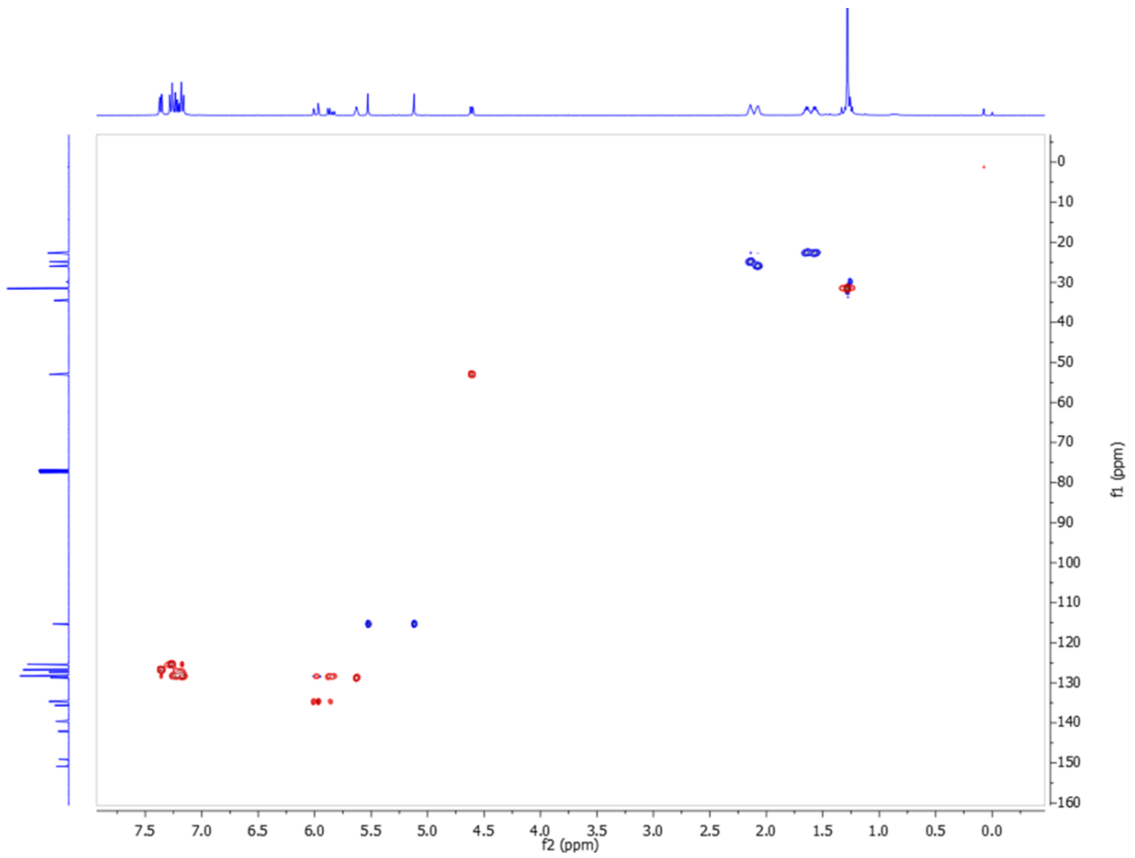

Figure SI 128: HSQC NMR spectrum of (*E*)-1-(cyclohex-1-en-1-yl)-3-(*para*-*tert*-butylphenyl)-4-phenyl-penta-1,4-diene **7h** (101 MHz, chloroform-*d*).

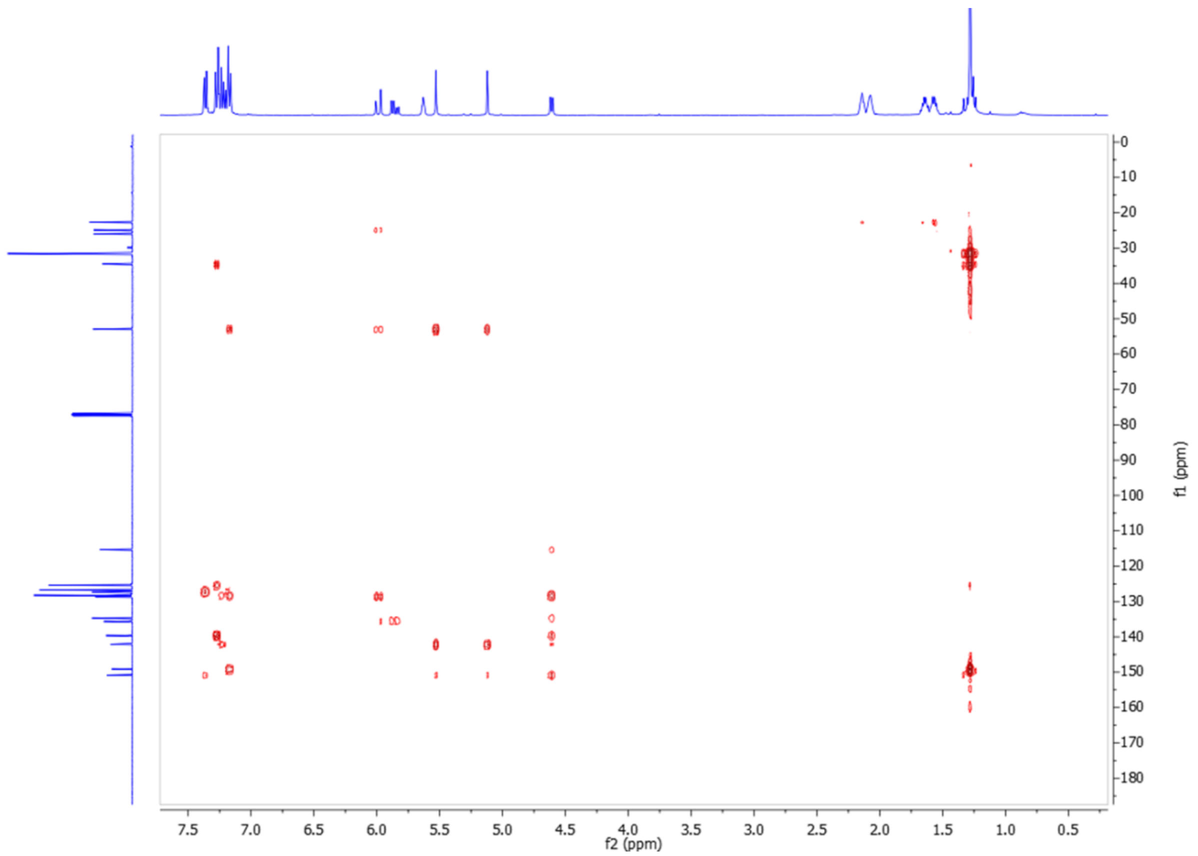

Figure SI 129: HMBC NMR spectrum of (*E*)-1-(cyclohex-1-en-1-yl)-3-(*para*-*tert*-butylphenyl)-4-phenyl-penta-1,4-diene **7h** (101 MHz, chloroform-*d*).

9.3.15 1,4-diene 7i

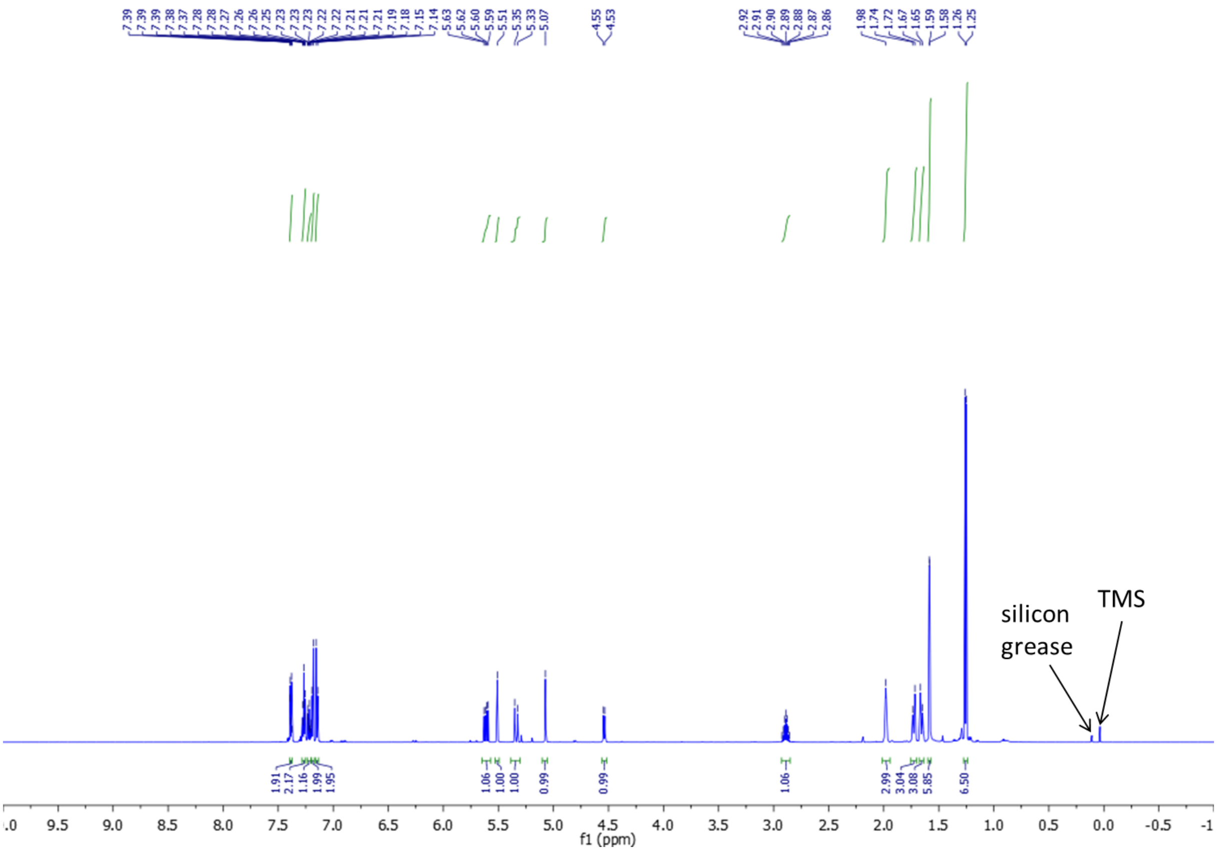

Figure SI 130: ^{1}H NMR spectrum of (*E*)-1-adamantyl-3-(*para*-*iso*-propylphenyl)-3-phenyl-penta-1,4-diene **7i** (600 MHz, chloroform-*d*).

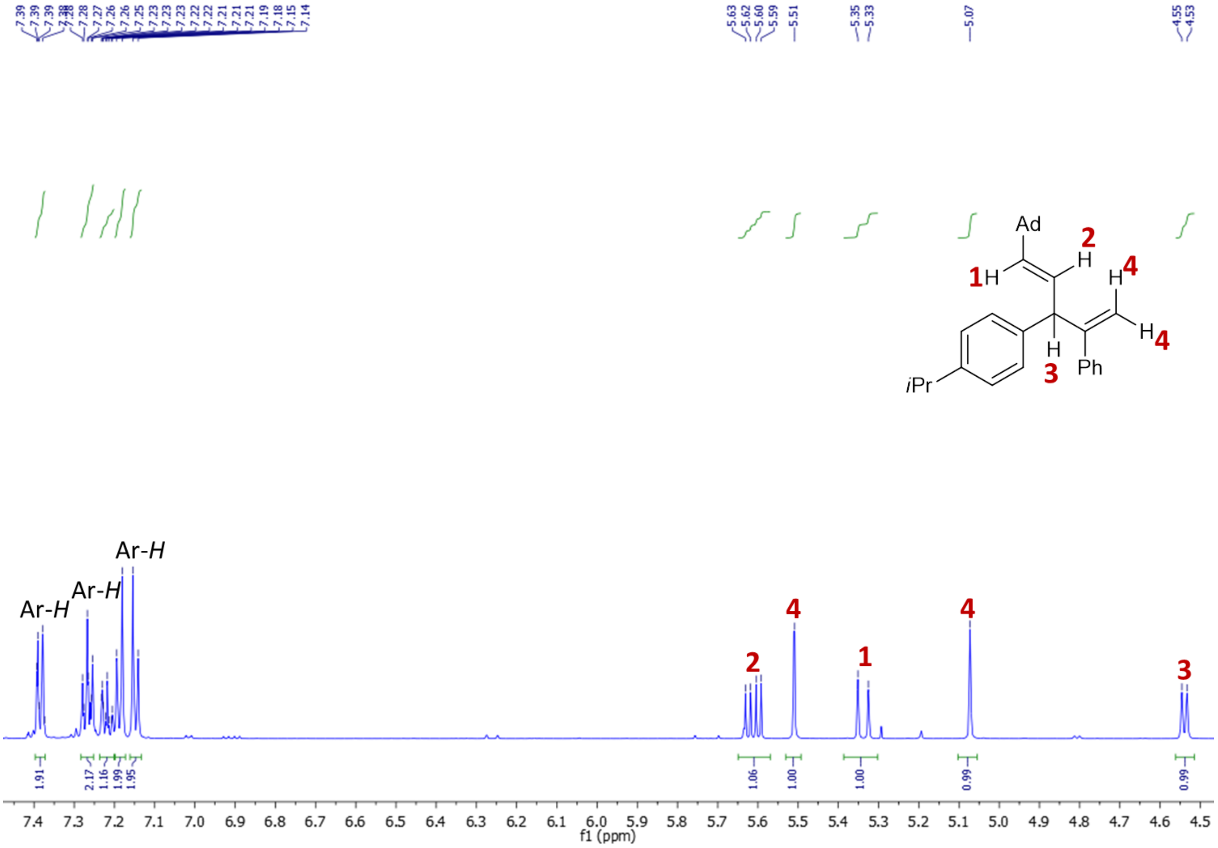

Figure SI 131: Excerpt of ^{1}H NMR spectrum of (*E*)-1-adamantyl-3-(*para*-*iso*-propylphenyl)-3-phenyl-penta-1,4-diene **7i** (600 MHz, chloroform-*d*).

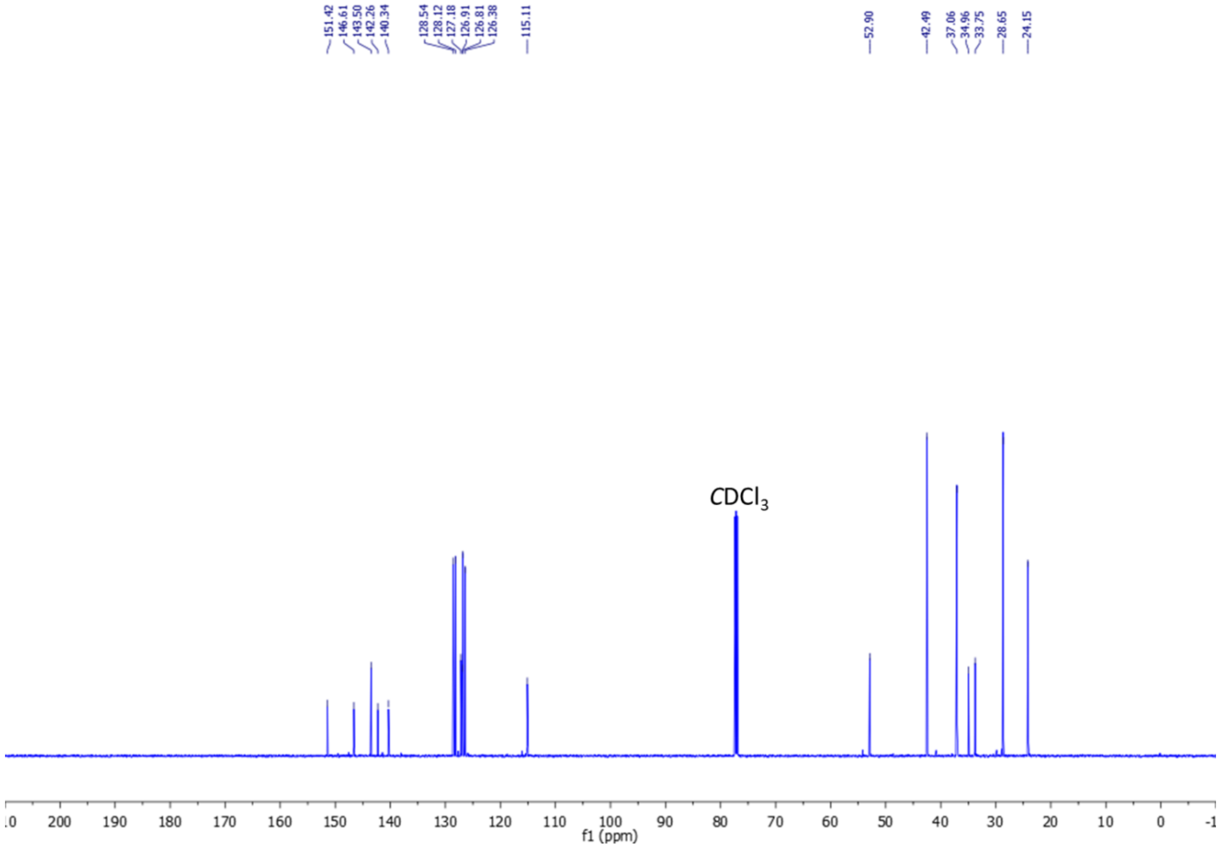

Figure SI 132: ^{13}C NMR spectrum of (*E*)-1-adamantyl-3-(*para*-*iso*-propylphenyl)-3-phenyl-penta-1,4-diene **7i** (151 MHz, chloroform-*d*).

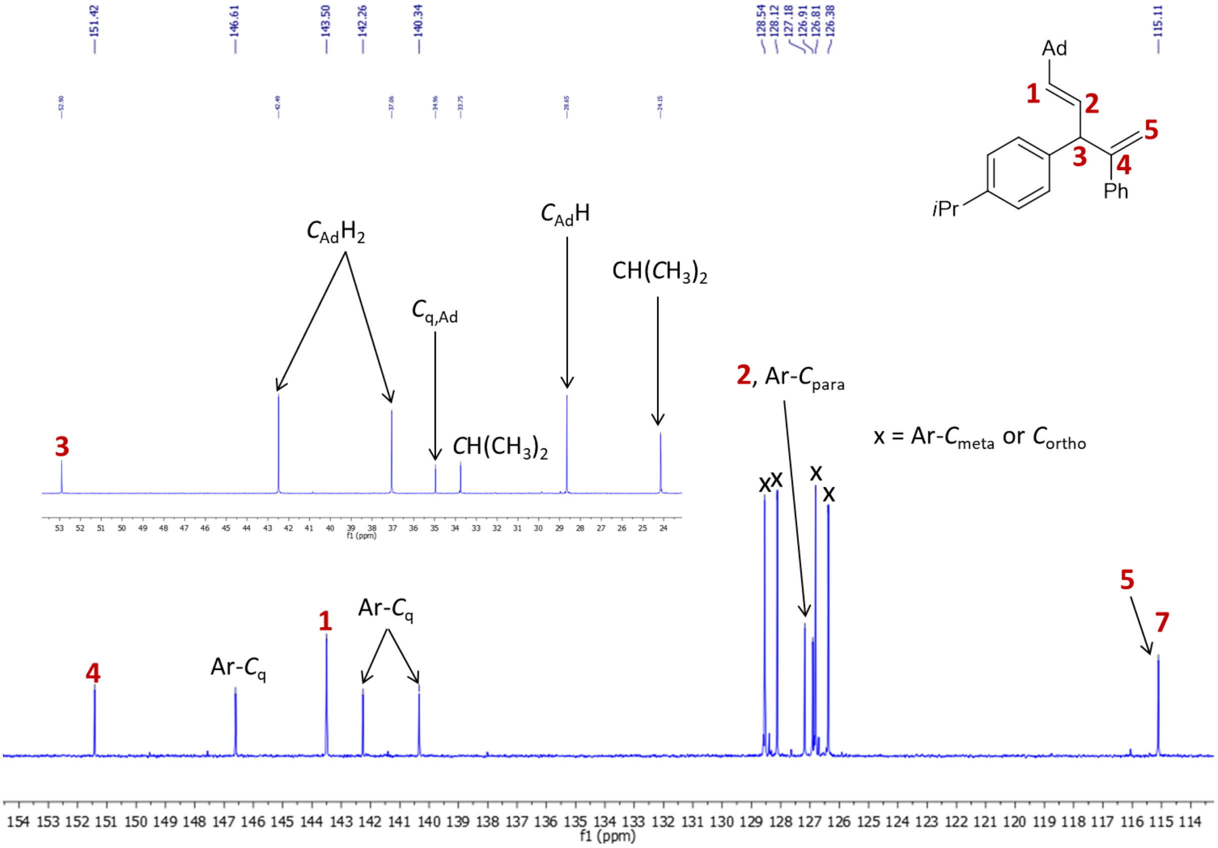

Figure SI 133: Excerpt of the ^{13}C NMR spectrum of (*E*)-1-adamantyl-3-(*para*-*iso*-propylphenyl)-3-phenyl-penta-1,4-diene **7i** (151 MHz, chloroform-*d*).

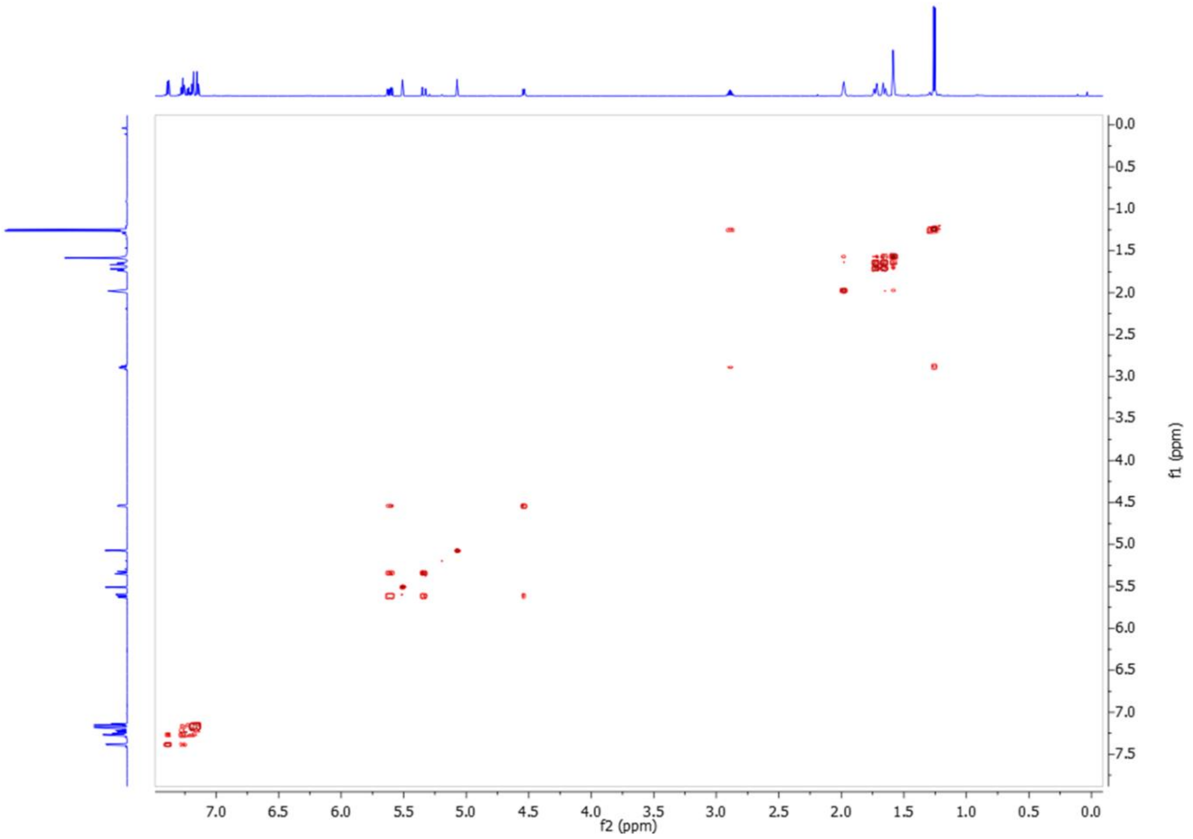

Figure SI 134: HH COSY NMR spectrum of (*E*)-1-adamantyl-3-(*para*-*iso*-propylphenyl)-3-phenyl-penta-1,4-diene **7i** (600 MHz, chloroform-*d*).

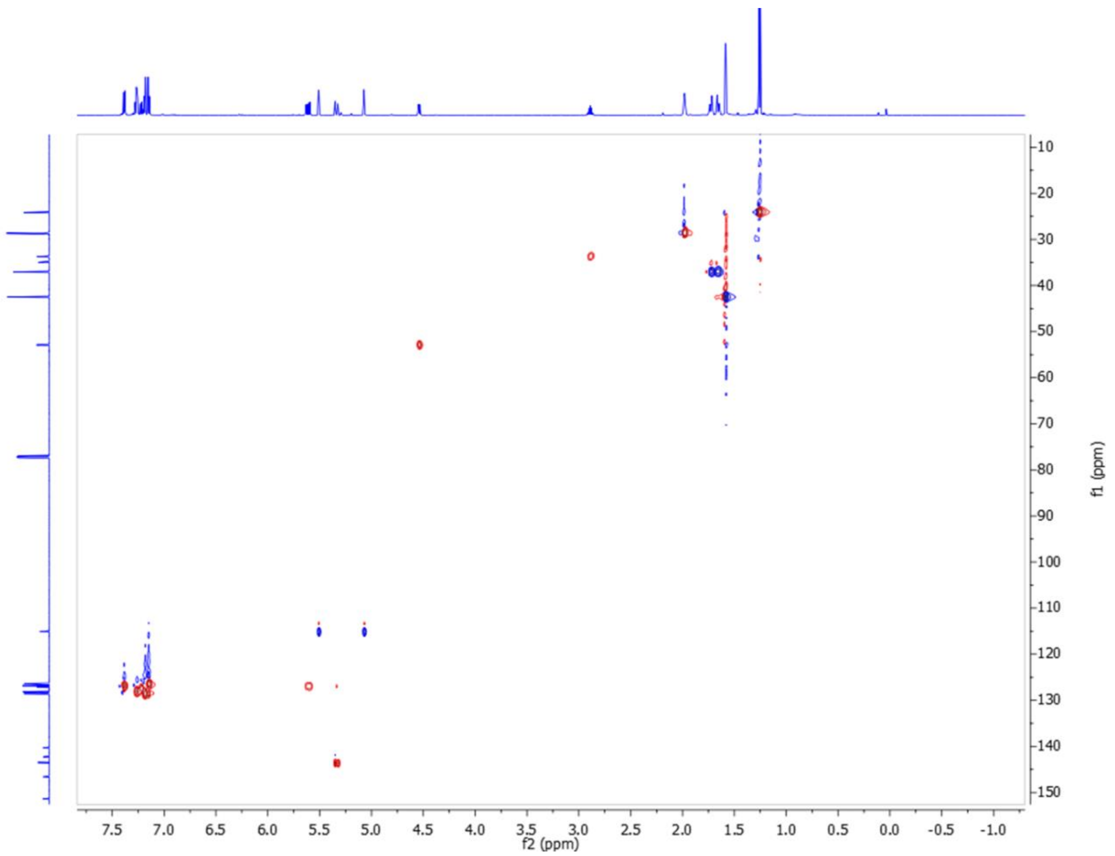

Figure SI 135: HSQC NMR spectrum of (*E*)-1-adamantyl-3-(*para*-*iso*-propylphenyl)-3-phenyl-penta-1,4-diene **7i** (151 MHz, chloroform-*d*).

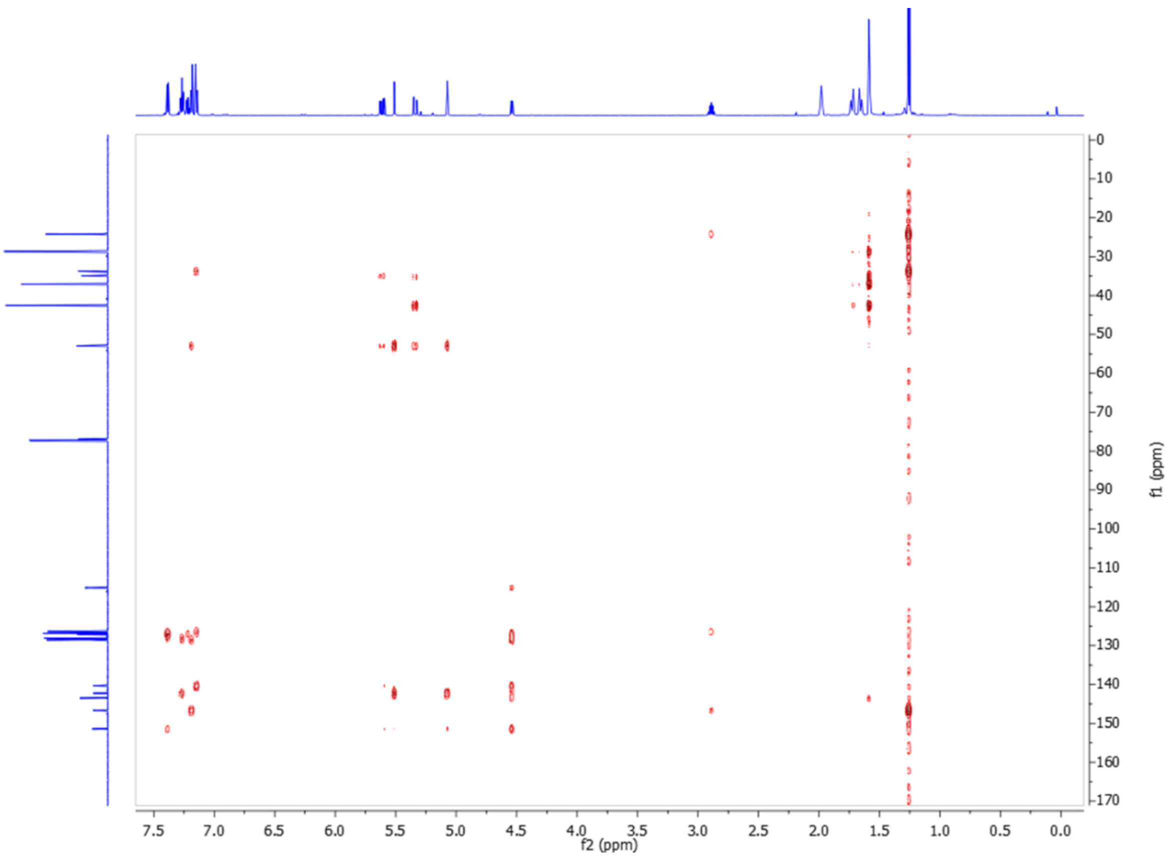

Figure SI 136: HMBC NMR spectrum of (*E*)-1-adamantyl-3-(*para*-*iso*-propylphenyl)-3-phenyl-penta-1,4-diene **7i** (151 MHz, chloroform-*d*).

9.3.16 1,4-diene 8a

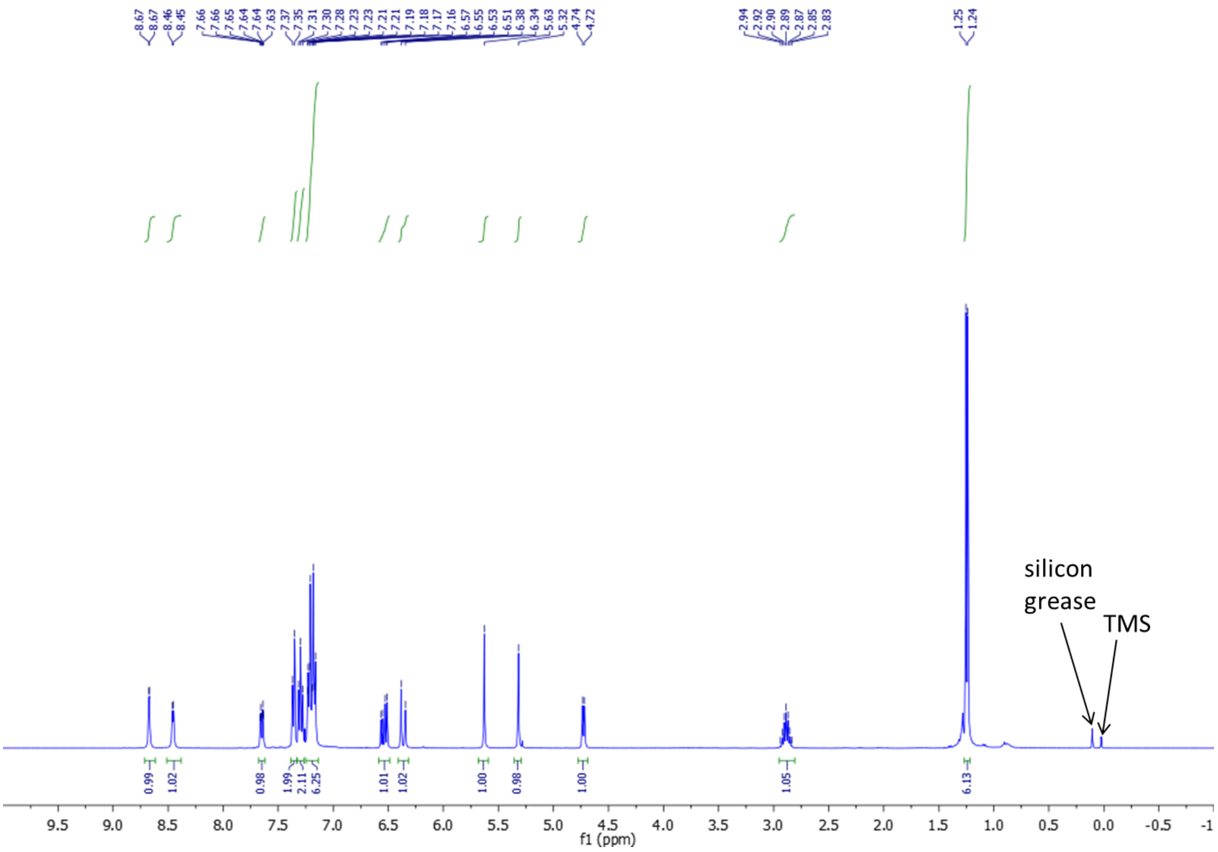

Figure SI 137: ^{1}H NMR spectrum of (*E*)-1-phenyl-3-(*para*-*iso*-propylphenyl)-4-(3-pyridyl)-penta-1,4-diene **8a** (400 MHz, chloroform-*d*).

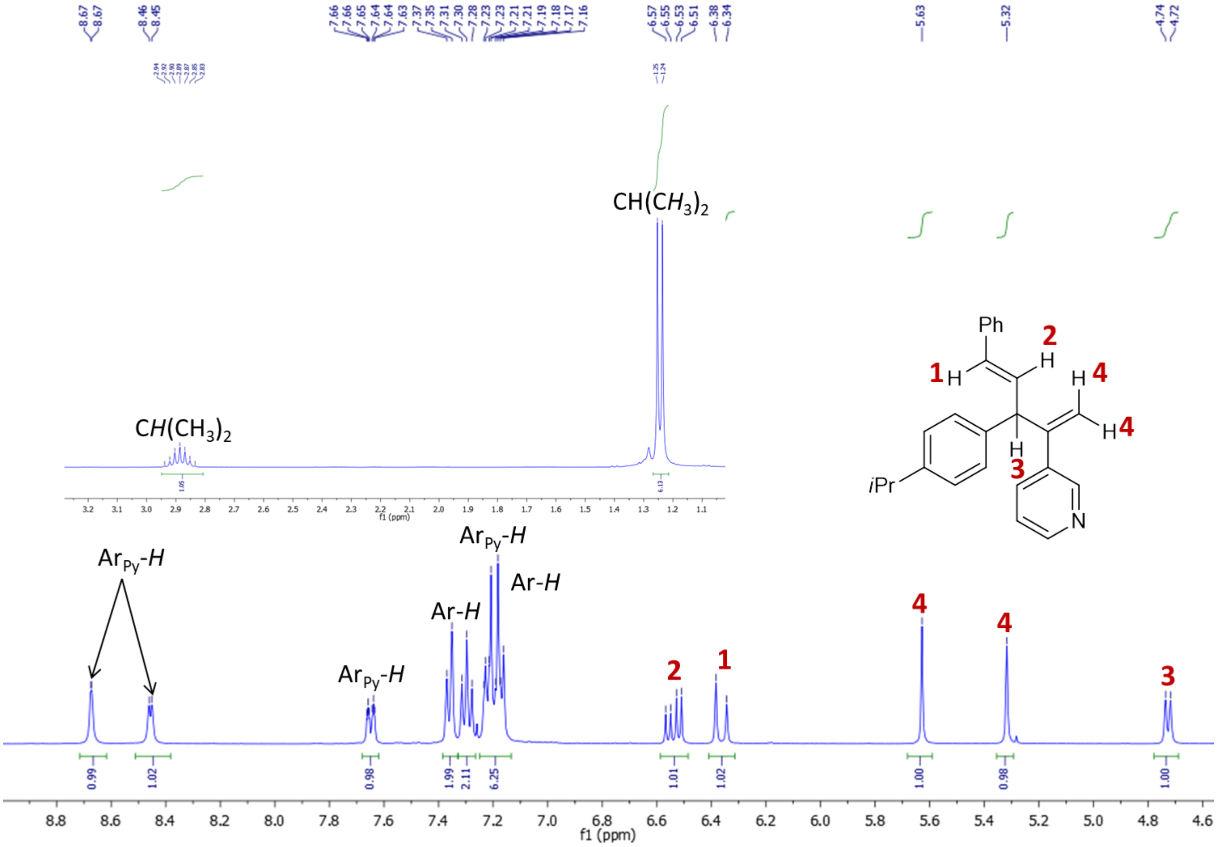

Figure SI 138: Excerpt of ^{1}H NMR spectrum of (*E*)-1-phenyl-3-(*para*-*iso*-propylphenyl)-4-(3-pyridyl)-penta-1,4-diene **8a** (400 MHz, chloroform-*d*).

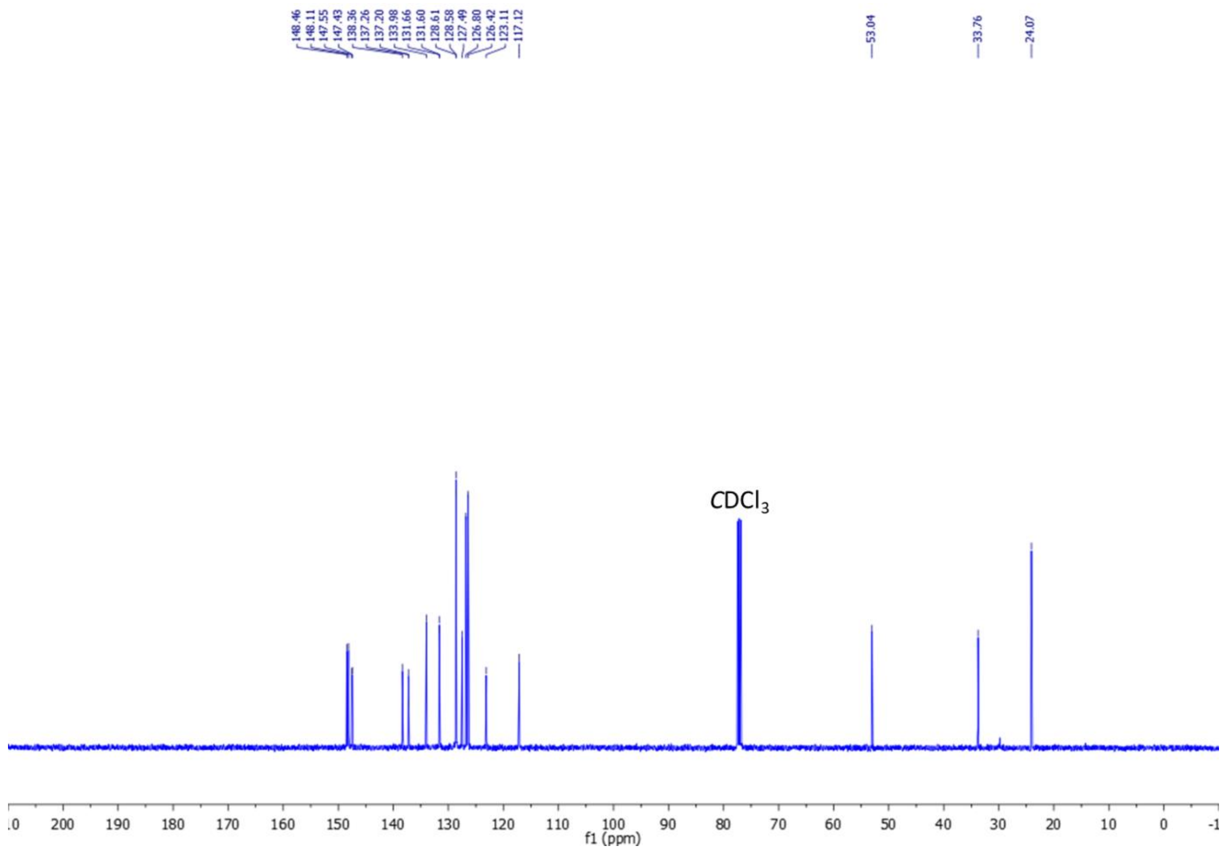

Figure SI 139: ^{13}C NMR spectrum of (*E*)-1-phenyl-3-(*para*-*iso*-propylphenyl)-4-(3-pyridyl)-penta-1,4-diene **8a** (101 MHz, chloroform-*d*).

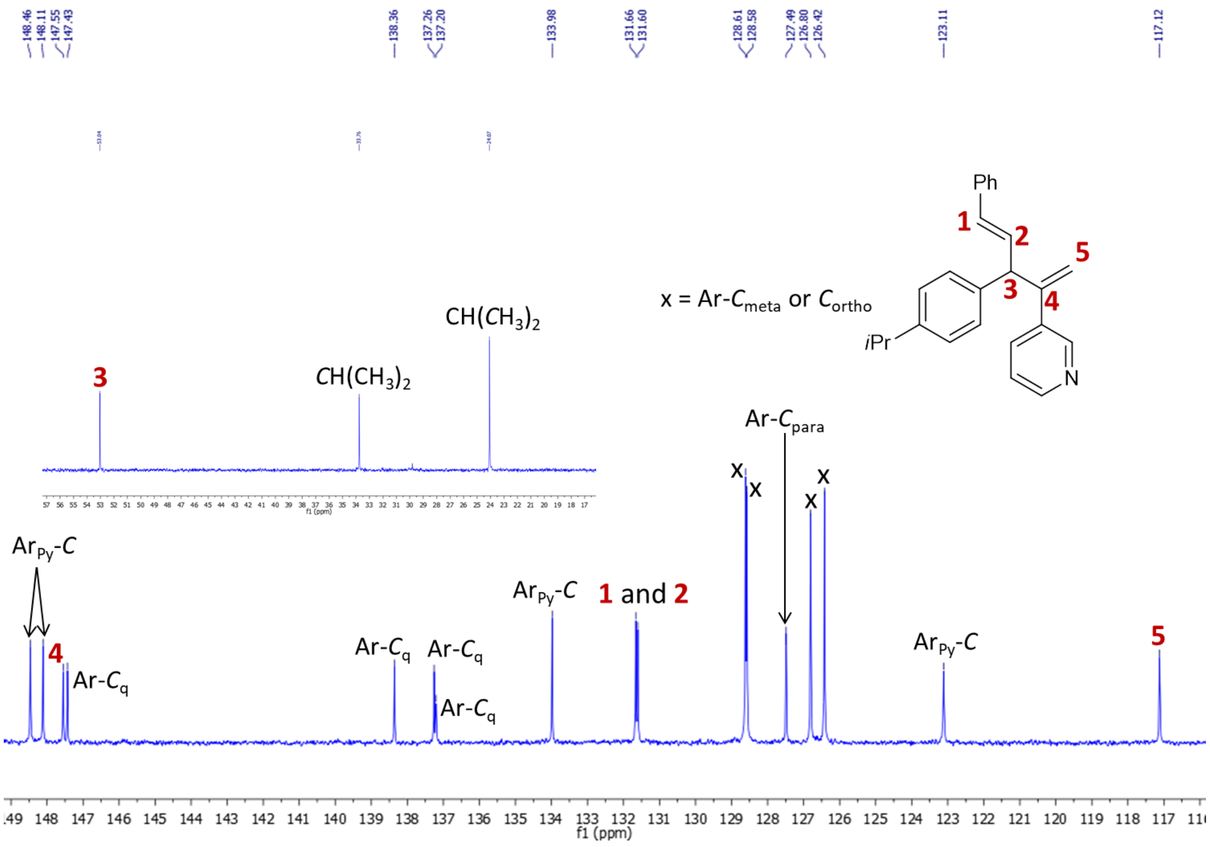

Figure SI 140: Excerpt of the ^{13}C NMR spectrum of (*E*)-1-phenyl-3-(*para*-*iso*-propylphenyl)-4-(3-pyridyl)-penta-1,4-diene **8a** (101 MHz, chloroform-*d*).

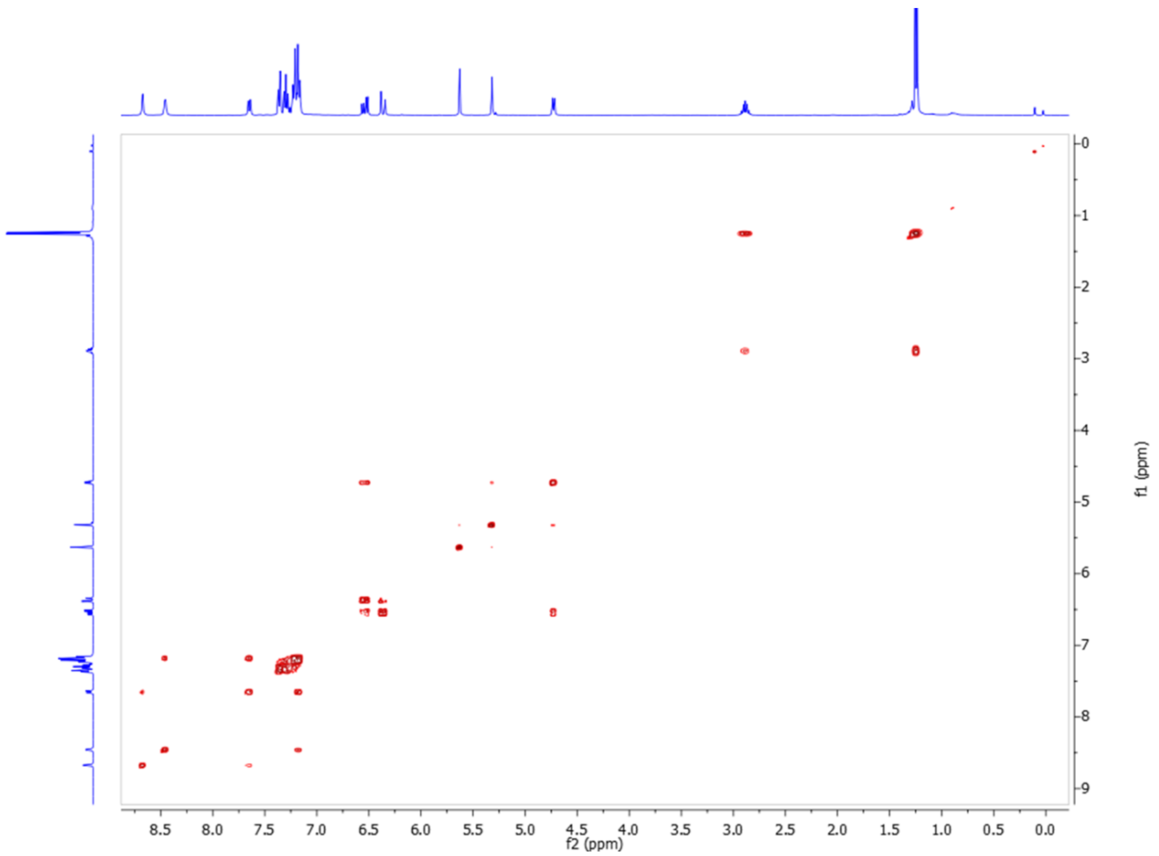

Figure SI 141: HH COSY NMR spectrum of (*E*)-1-phenyl-3-(*para*-*iso*-propylphenyl)-4-(3-pyridyl)-penta-1,4-diene **8a** (400 MHz, chloroform-*d*).

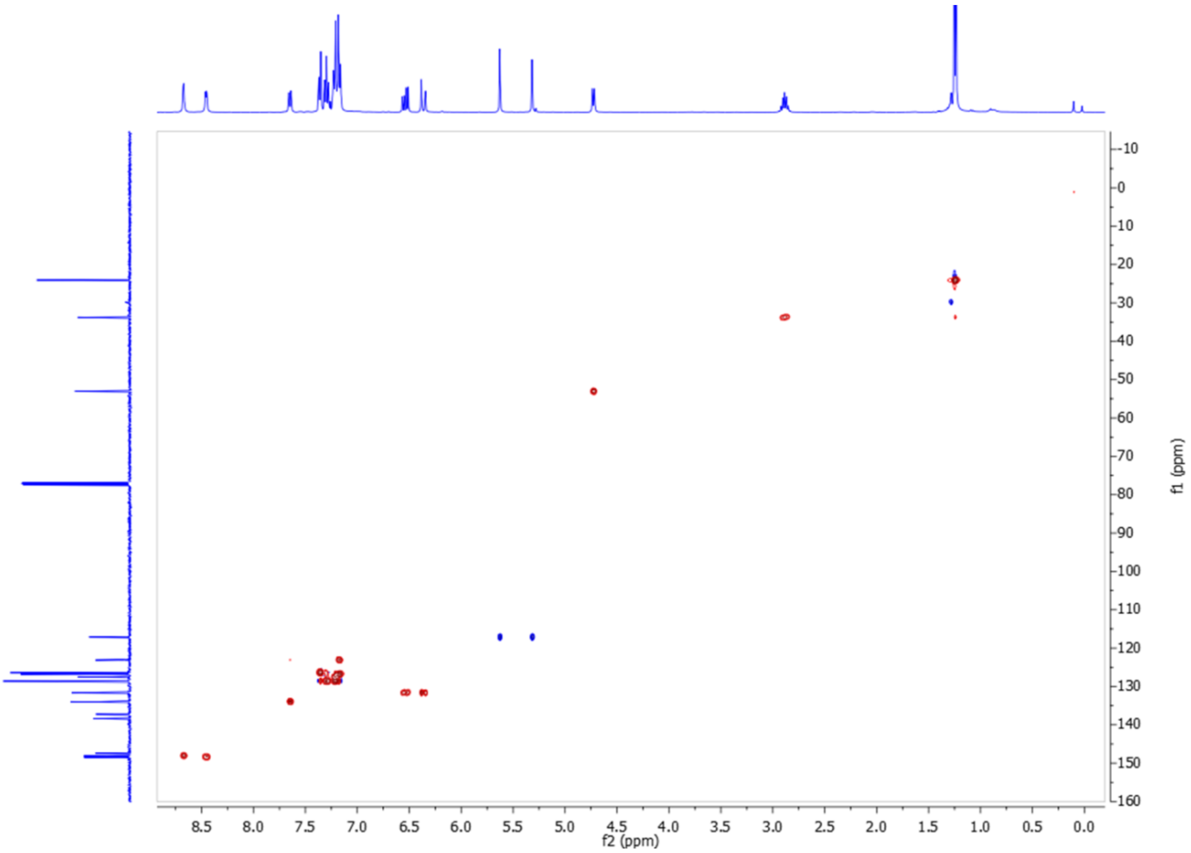

Figure SI 142: HSQC NMR spectrum of (*E*)-1-phenyl-3-(*para*-*iso*-propylphenyl)-4-(3-pyridyl)-penta-1,4-diene **8a** (101 MHz, chloroform-*d*).

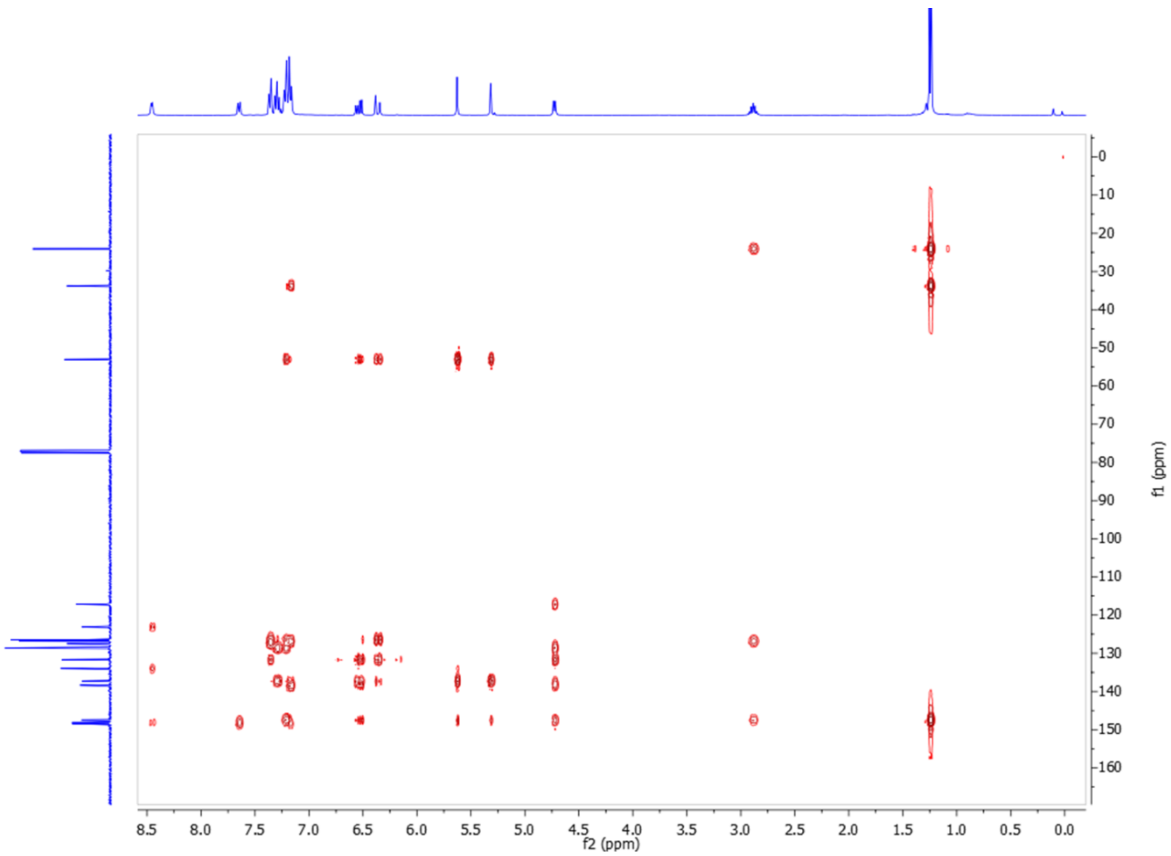

Figure SI 143: HMBC NMR spectrum of (*E*)-1-phenyl-3-(*para*-*iso*-propylphenyl)-4-(3-pyridyl)-penta-1,4-diene **8a** (101 MHz, chloroform-*d*).

9.3.17 1,4-diene 8b

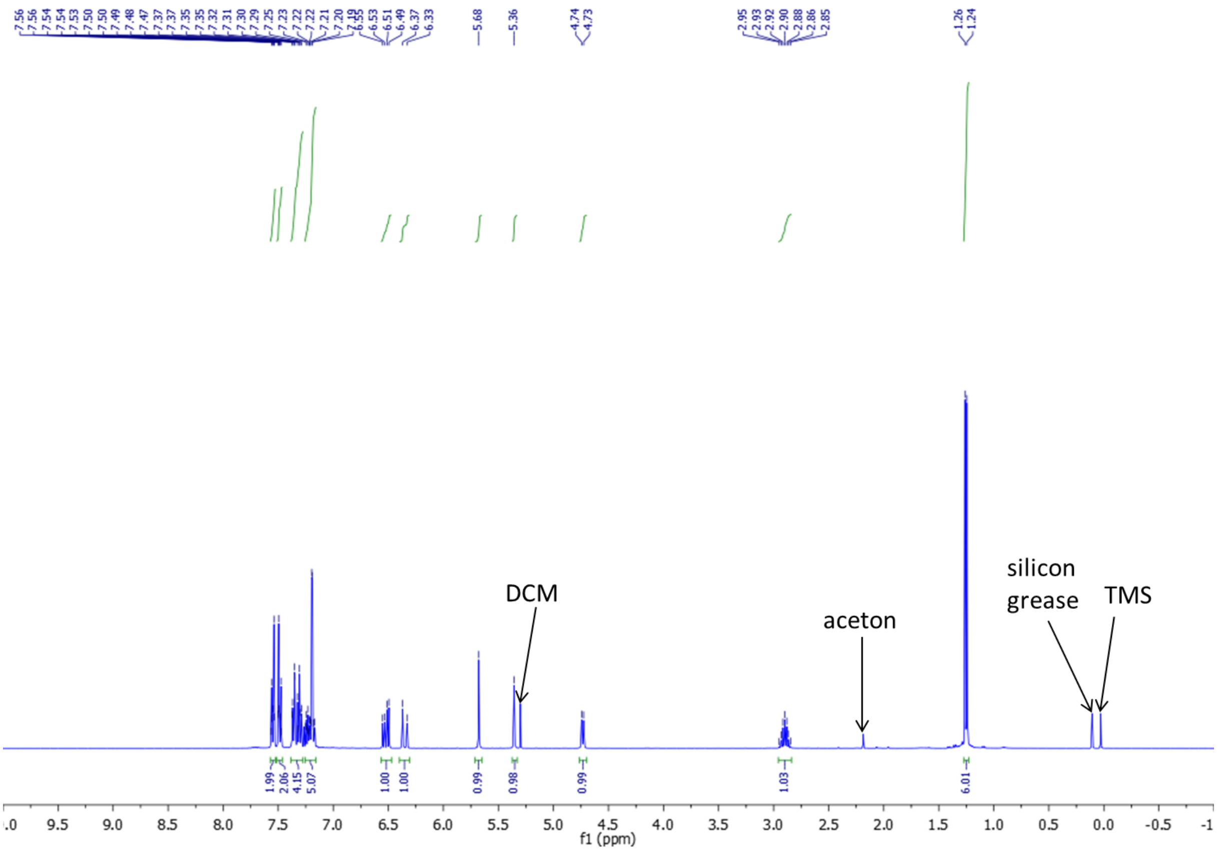

Figure SI 144: ^{1}H NMR spectrum of (*E*)-1-phenyl-3-(*para*-*iso*-propylphenyl)-4-(*para*-cyanophenyl)-penta-1,4 **8b** (400 MHz, chloroform-*d*).

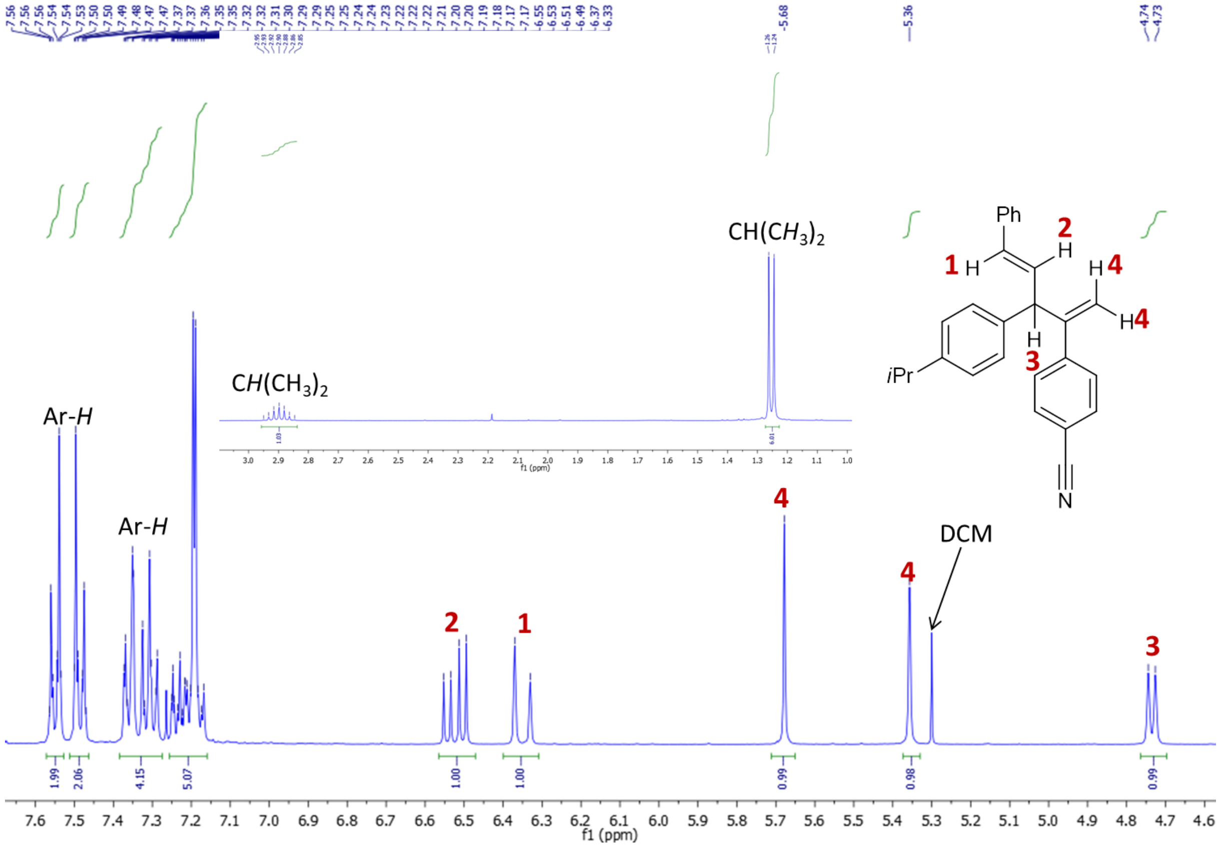

Figure SI 145: Excerpt of ^{1}H NMR spectrum of (E)-1-phenyl-3-(*para*-*iso*-propylphenyl)-4-(*para*-cyanophenyl)-penta-1,4-diene **8b** (400 MHz, chloroform-d).

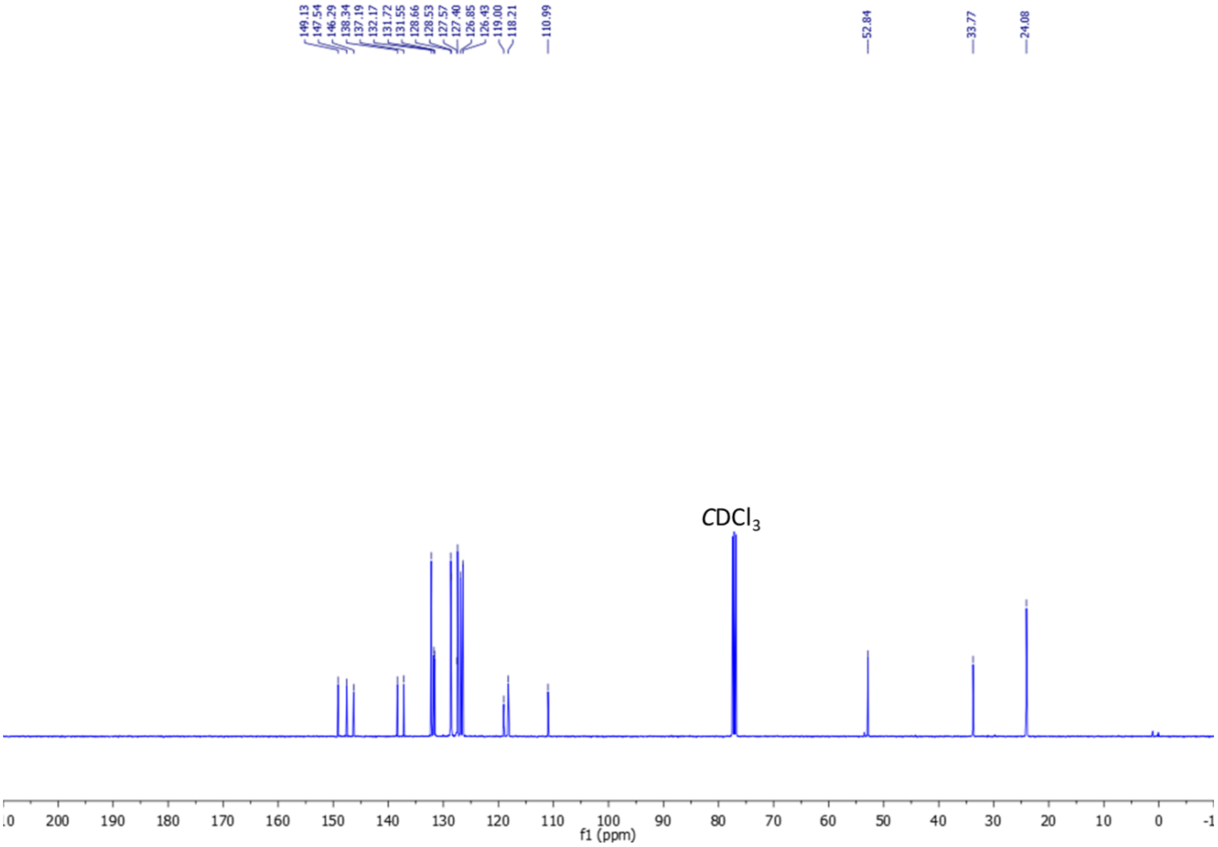

Figure SI 146: ^{13}C NMR spectrum of (*E*)-1-phenyl-3-(*para*-*iso*-propylphenyl)-4-(*para*-cyanophenyl)-penta-1,4-diene **8b** (101 MHz, chloroform-*d*).

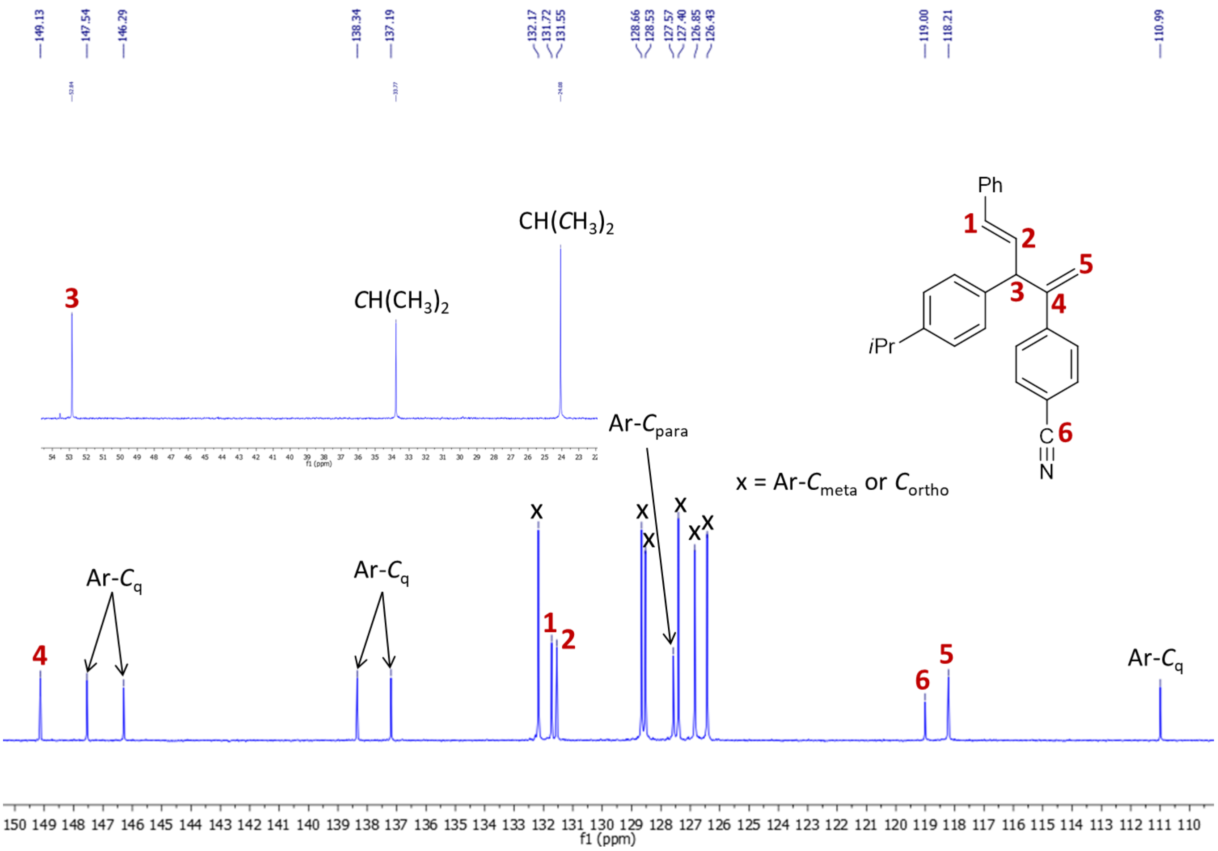

Figure SI 147: Excerpt of the ^{13}C NMR spectrum of (*E*)-1-phenyl-3-(*para*-*iso*-propylphenyl)-4-(*para*-cyanophenyl)-penta-1,4-diene **8b** (101 MHz, chloroform-*d*).

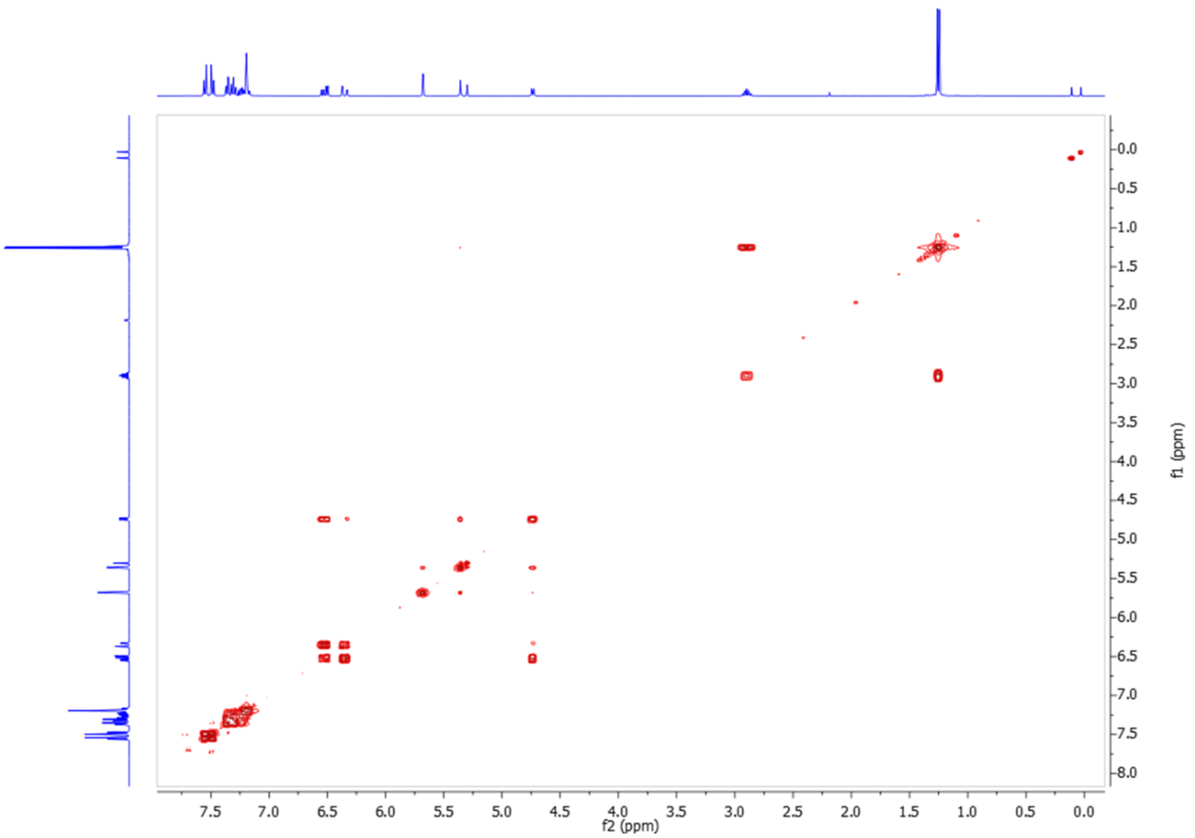

Figure SI 148: HH COSY NMR spectrum of (*E*)-1-phenyl-3-(*para*-*iso*-propylphenyl)-4-(*para*-cyanophenyl)-penta-1,4-diene **8b** (400 MHz, chloroform-*d*).

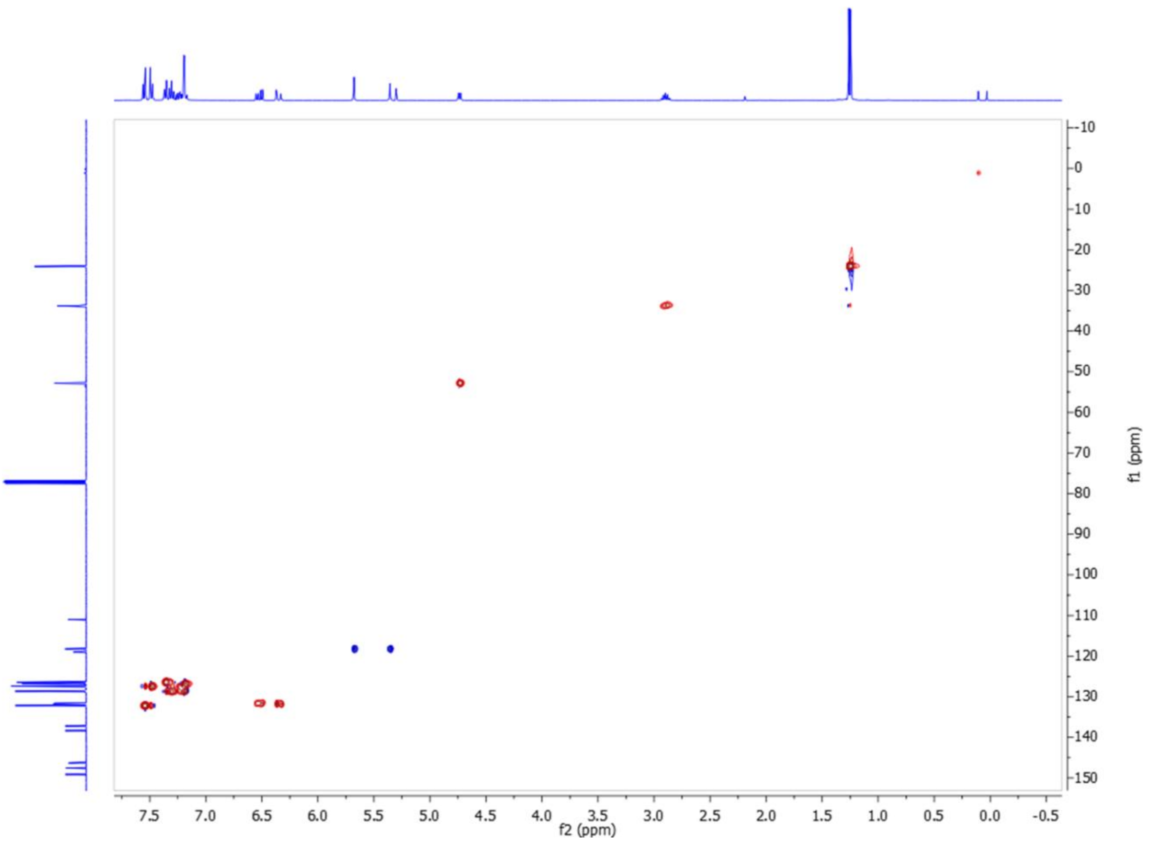

Figure SI 149: HSQC NMR spectrum of (*E*)-1-phenyl-3-(*para*-*iso*-propylphenyl)-4-(*para*-cyanophenyl)-penta-1,4-diene **8b** (101 MHz, chloroform-*d*).

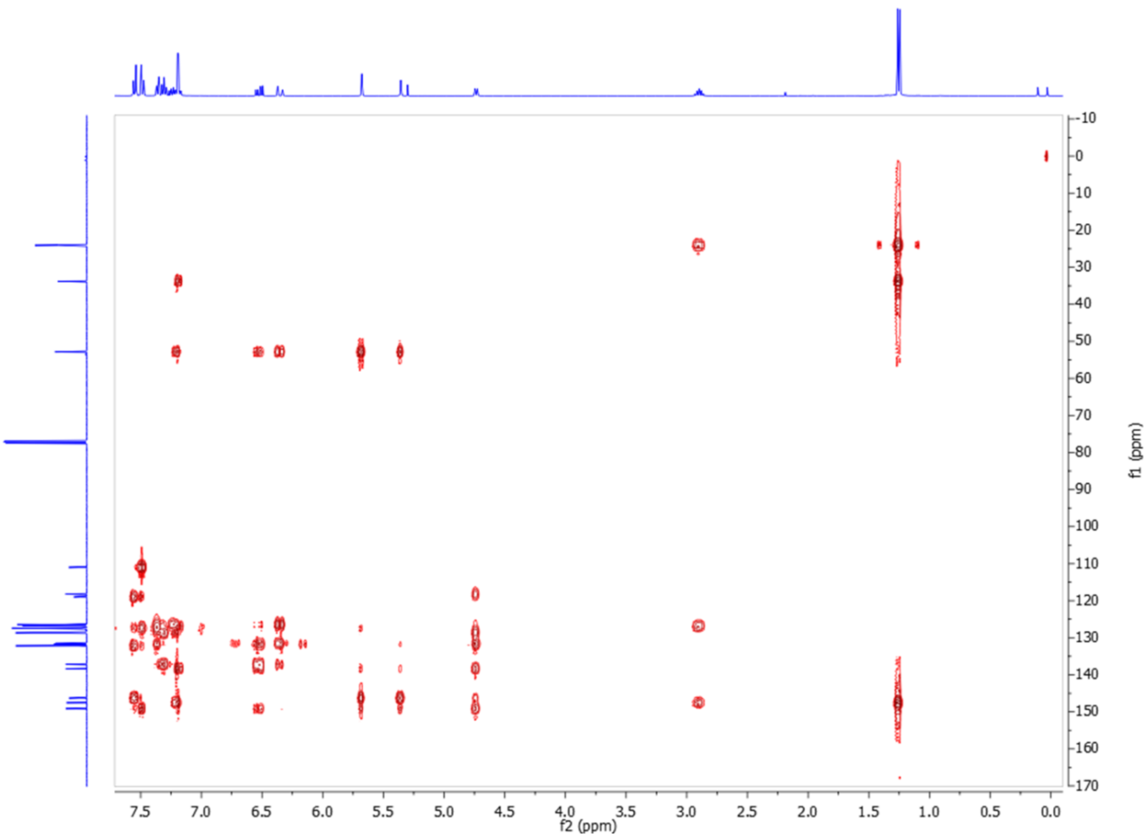

Figure SI 150: HMBC NMR spectrum of (*E*)-1-phenyl-3-(*para*-*iso*-propylphenyl)-4-(*para*-cyanophenyl)-penta-1,4-diene **8b** (101 MHz, chloroform-*d*).

9.3.18 1,4-diene 8c

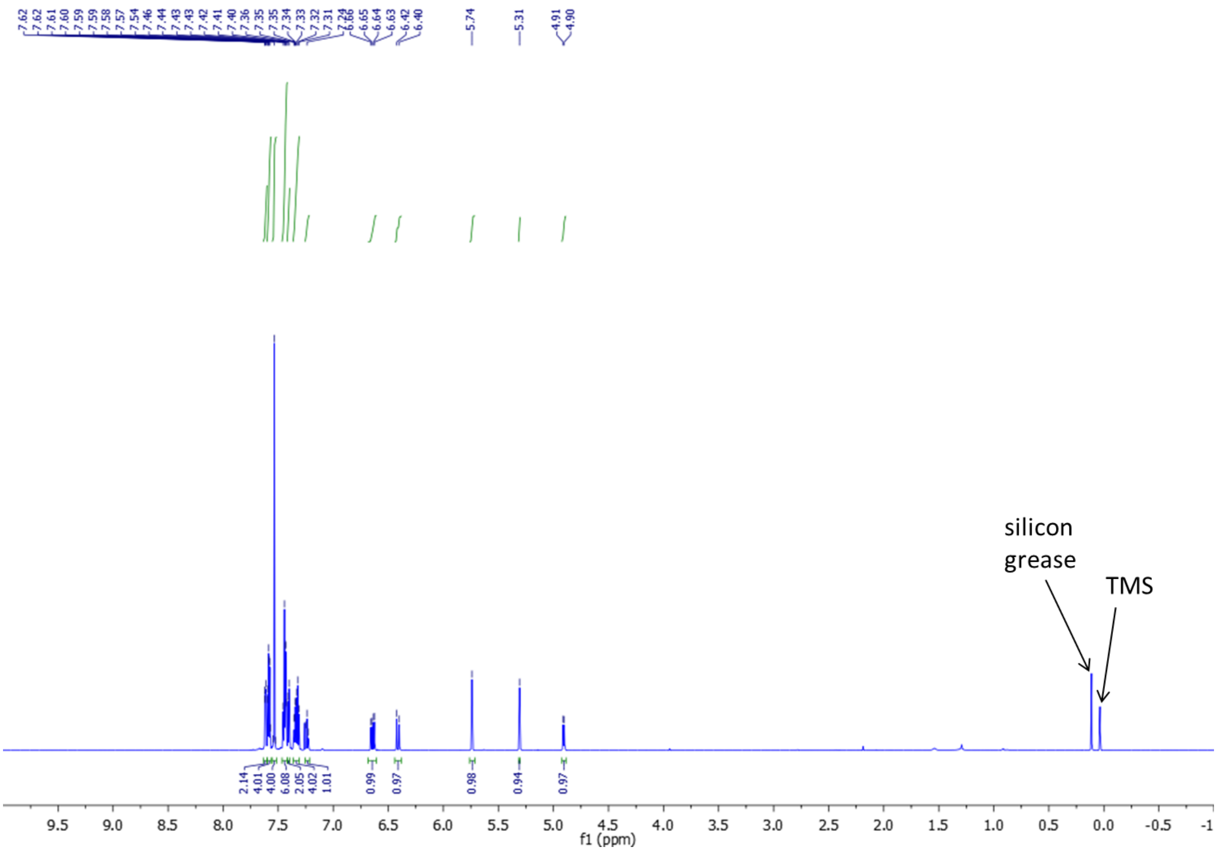

Figure SI 151: ^{1}H NMR spectrum of (*E*)-1-phenyl-3,4-di-(4-biphenyl)-penta-1,4-diene **8c** (700 MHz, chloroform-*d*).

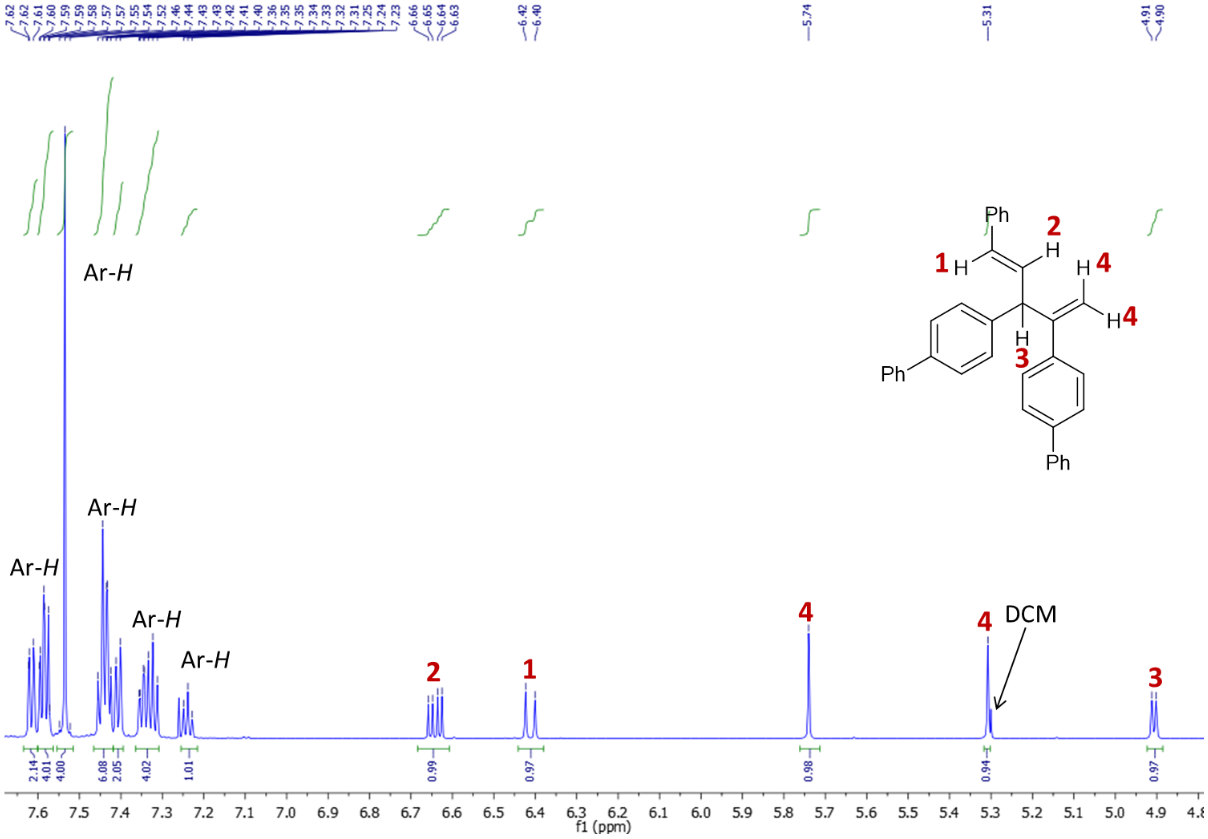

Figure SI 152: Excerpt of ^{1}H NMR spectrum of (*E*)-1-phenyl-3,4-di-(4-biphenyl)-penta-1,4-diene **8c** (700 MHz, chloroform-*d*).

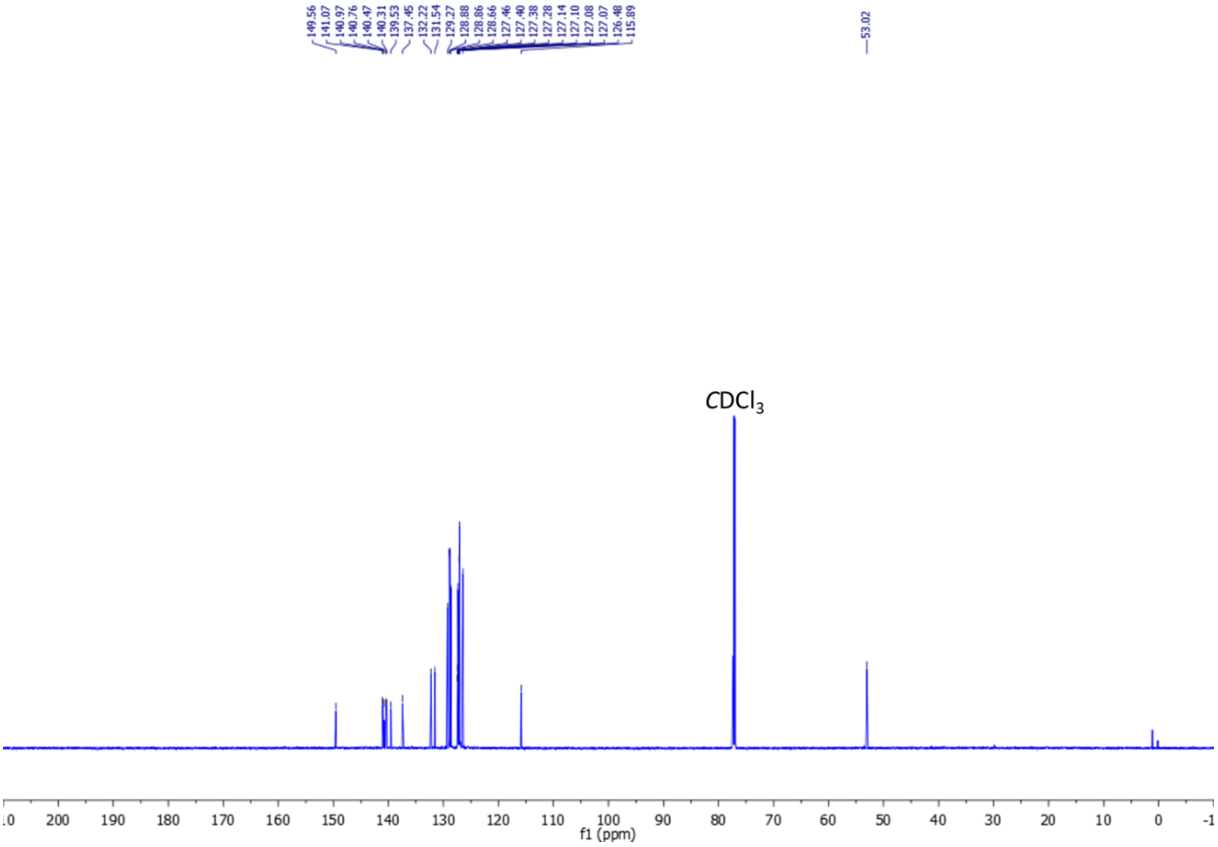

Figure SI 153: ^{13}C NMR spectrum of (*E*)-1-phenyl-3,4-di-(4-biphenyl)-penta-1,4-diene **8c** (176 MHz, chloroform-*d*).

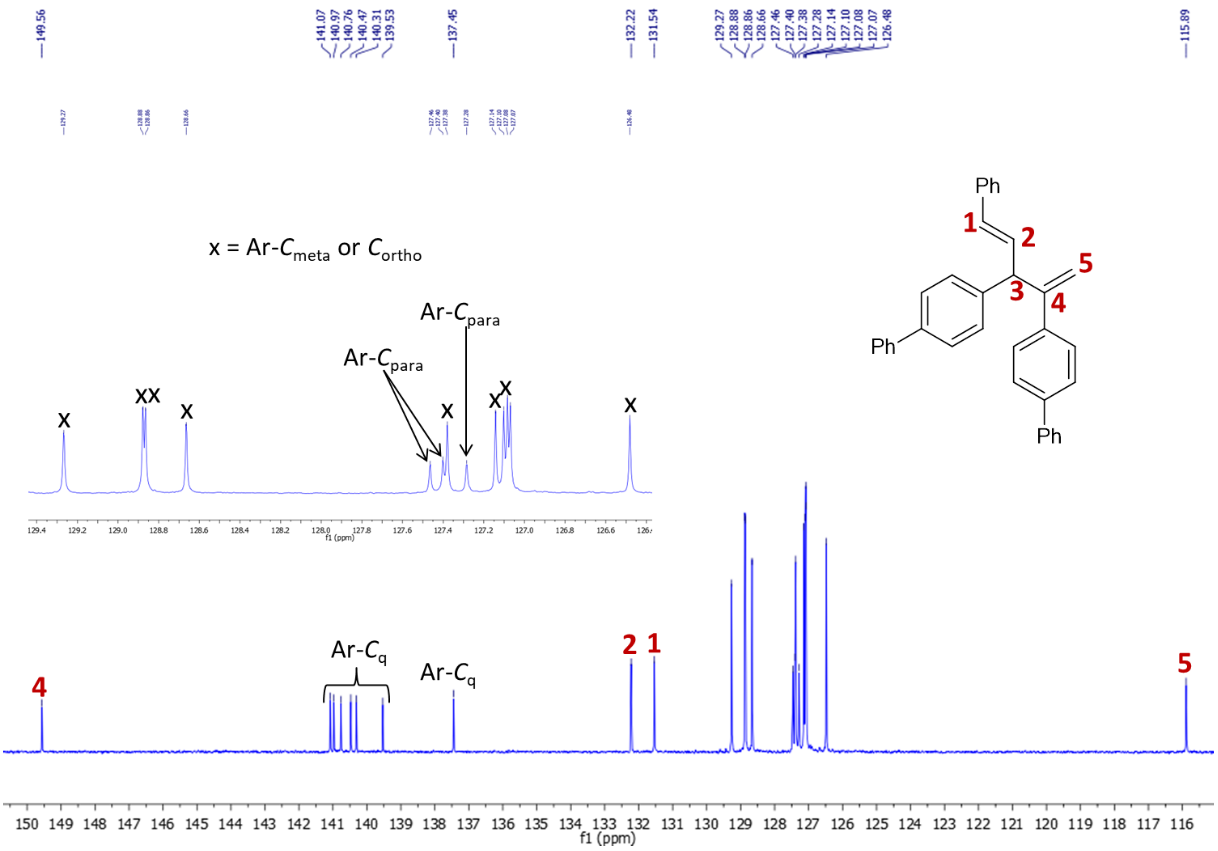

Figure SI 154: Excerpt of the ^{13}C NMR spectrum of (*E*)-1-phenyl-3,4-di-(4-biphenyl)-penta-1,4-diene **8c** (176 MHz, chloroform-*d*).

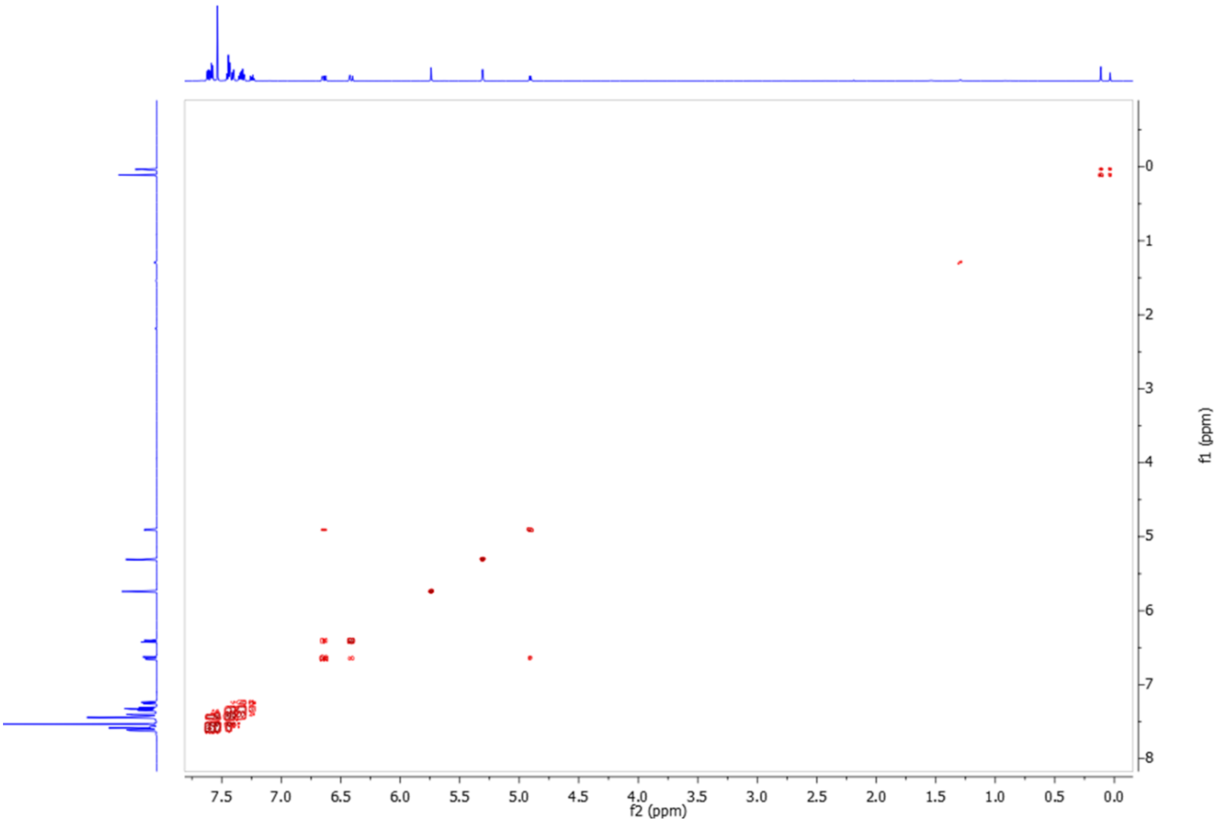

Figure SI 155: HH COSY NMR spectrum of (*E*)-1-phenyl-3,4-di-(4-biphenyl)-penta-1,4-diene **8c** (700 MHz, chloroform-*d*).

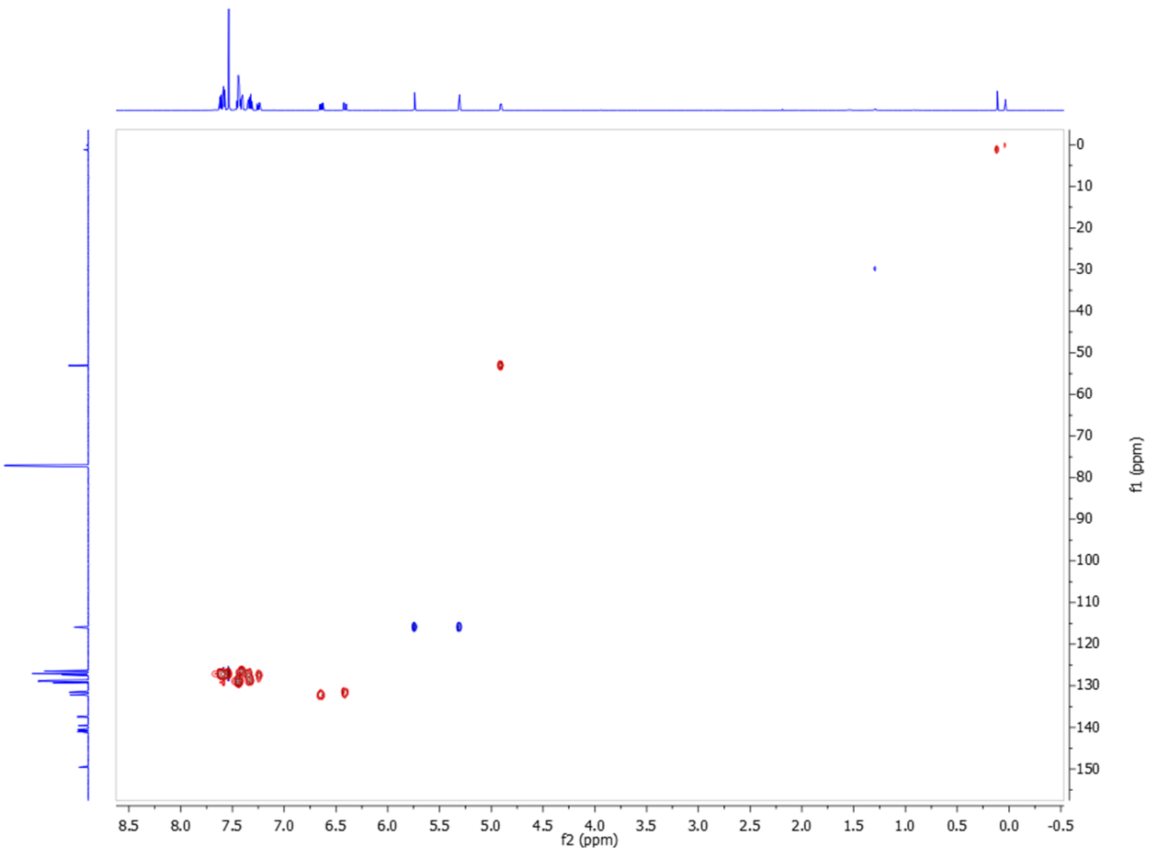

Figure SI 156: HSQC NMR spectrum of (*E*)-1-phenyl-3,4-di-(4-biphenyl)-penta-1,4-diene **8c** (176 MHz, chloroform-*d*).

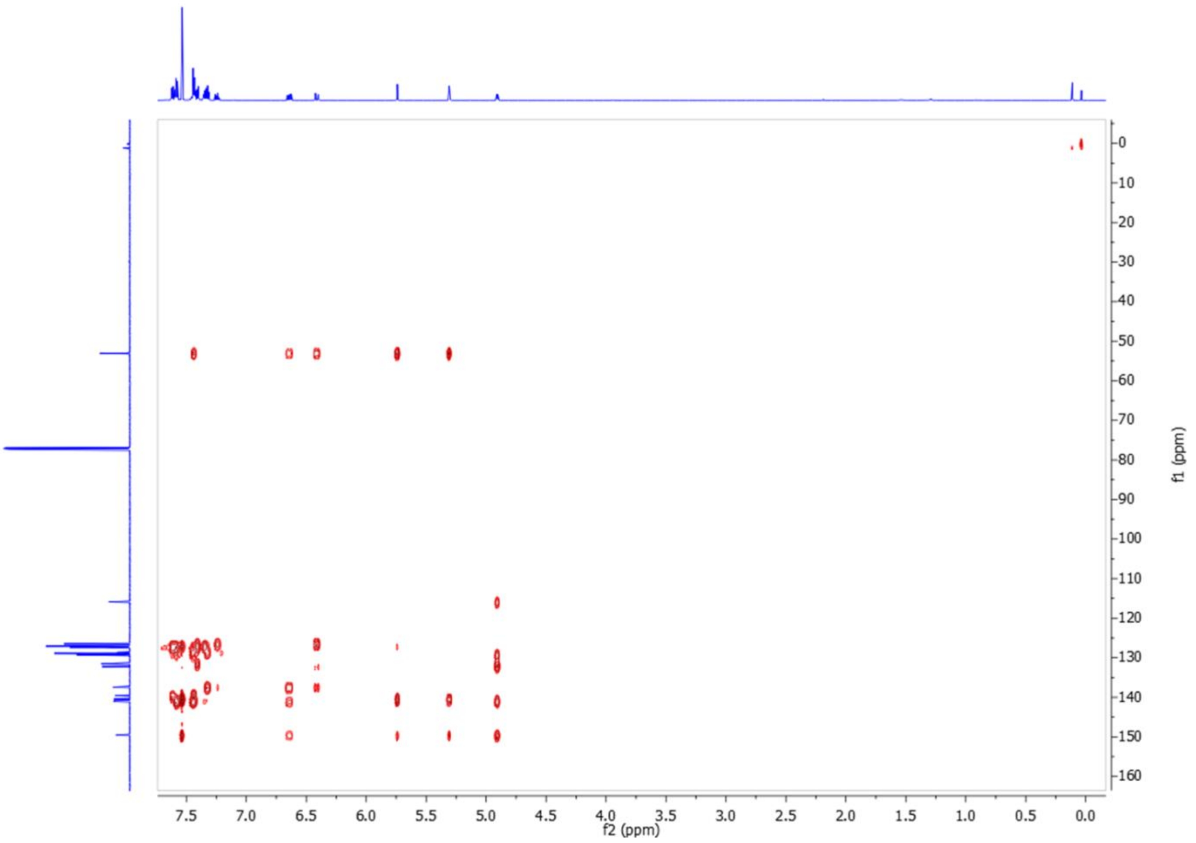

Figure SI 157: HMBC NMR spectrum of (*E*)-1-phenyl-3,4-di-(4-biphenyl)-penta-1,4-diene **8c** (176 MHz, chloroform-*d*).

9.3.19 1,4-diene 9

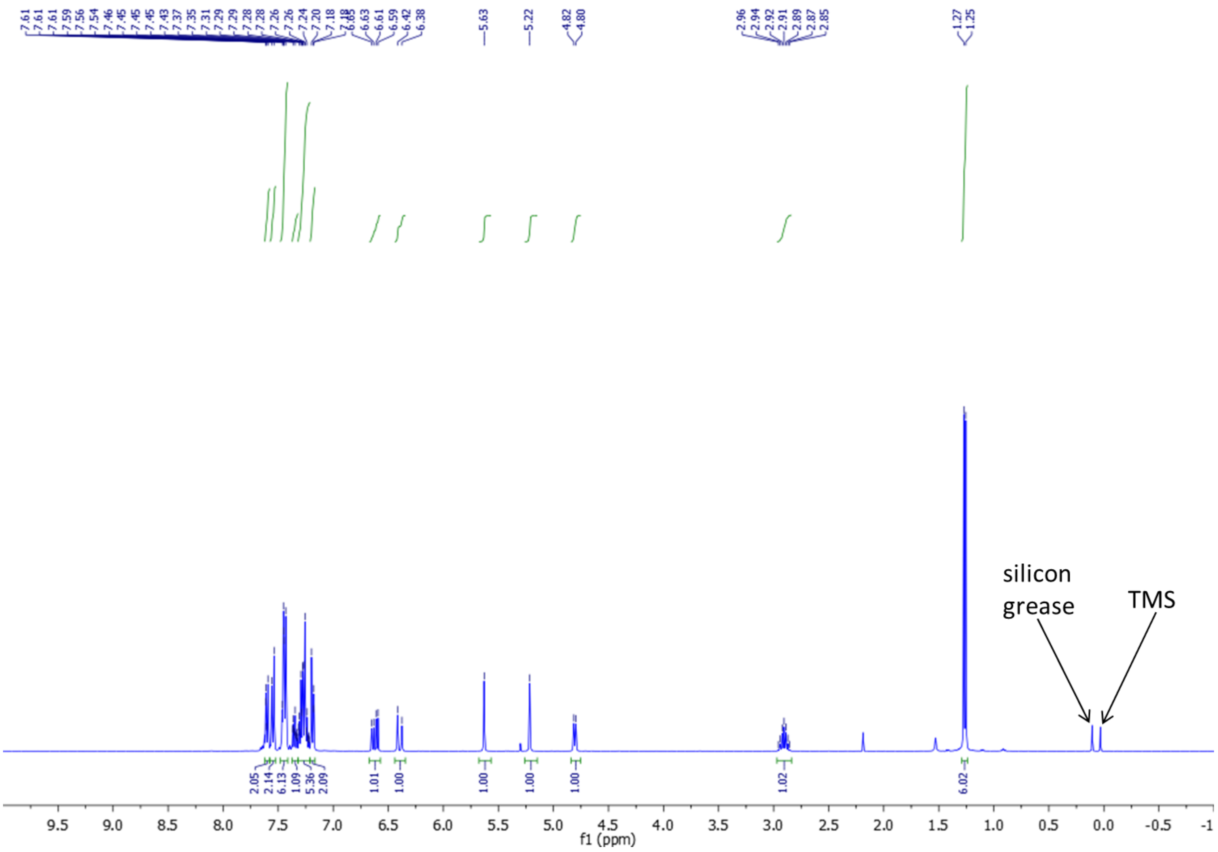

Figure SI 158: ^{1}H NMR spectrum of (*E*)-1-(4-biphenyl)-3-(*para*-*iso*-propylphenyl)-3-phenyl-penta-1,4-diene **9** (400 MHz, chloroform-*d*).

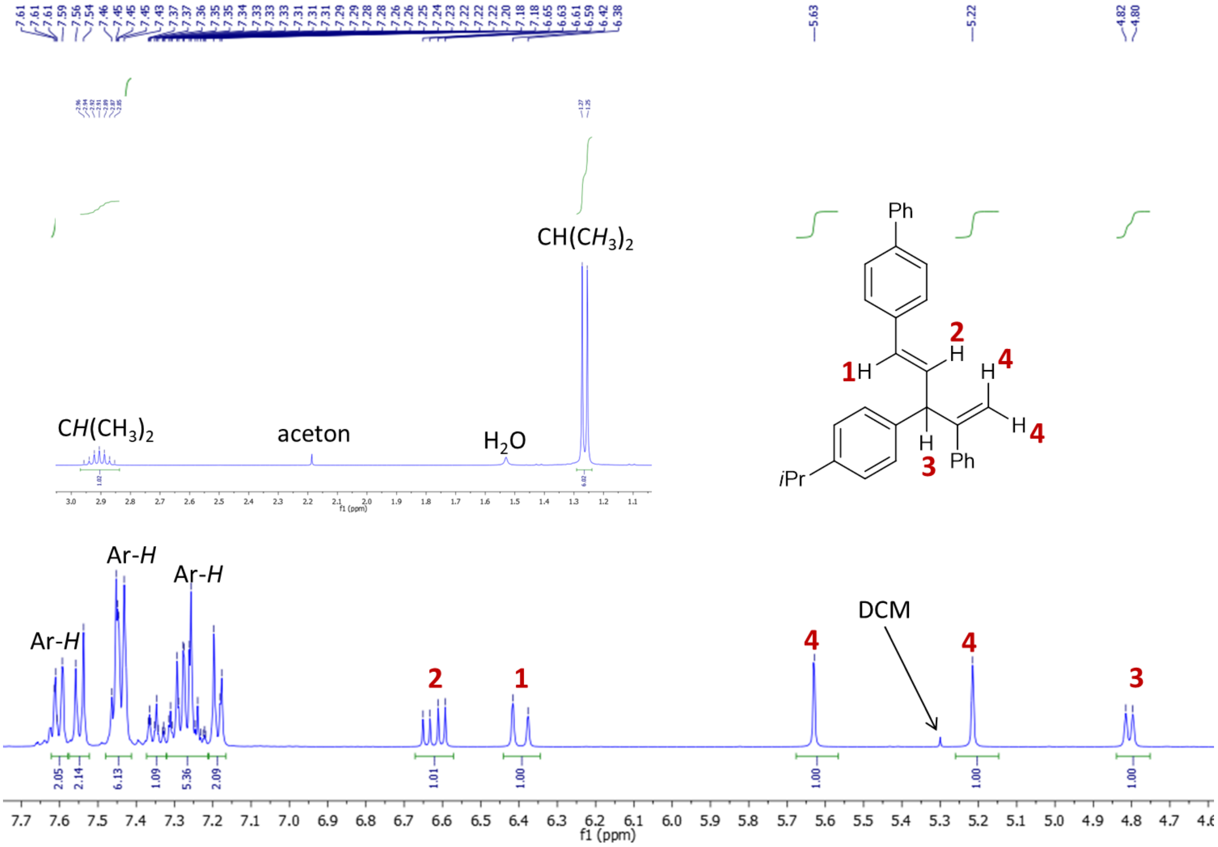

Figure SI 159: Excerpt of ^{1}H NMR spectrum of (*E*)-1-(4-biphenyl)-3-(*para*-*iso*-propylphenyl)-3-phenyl-penta-1,4-diene **9** (400 MHz, chloroform-*d*).

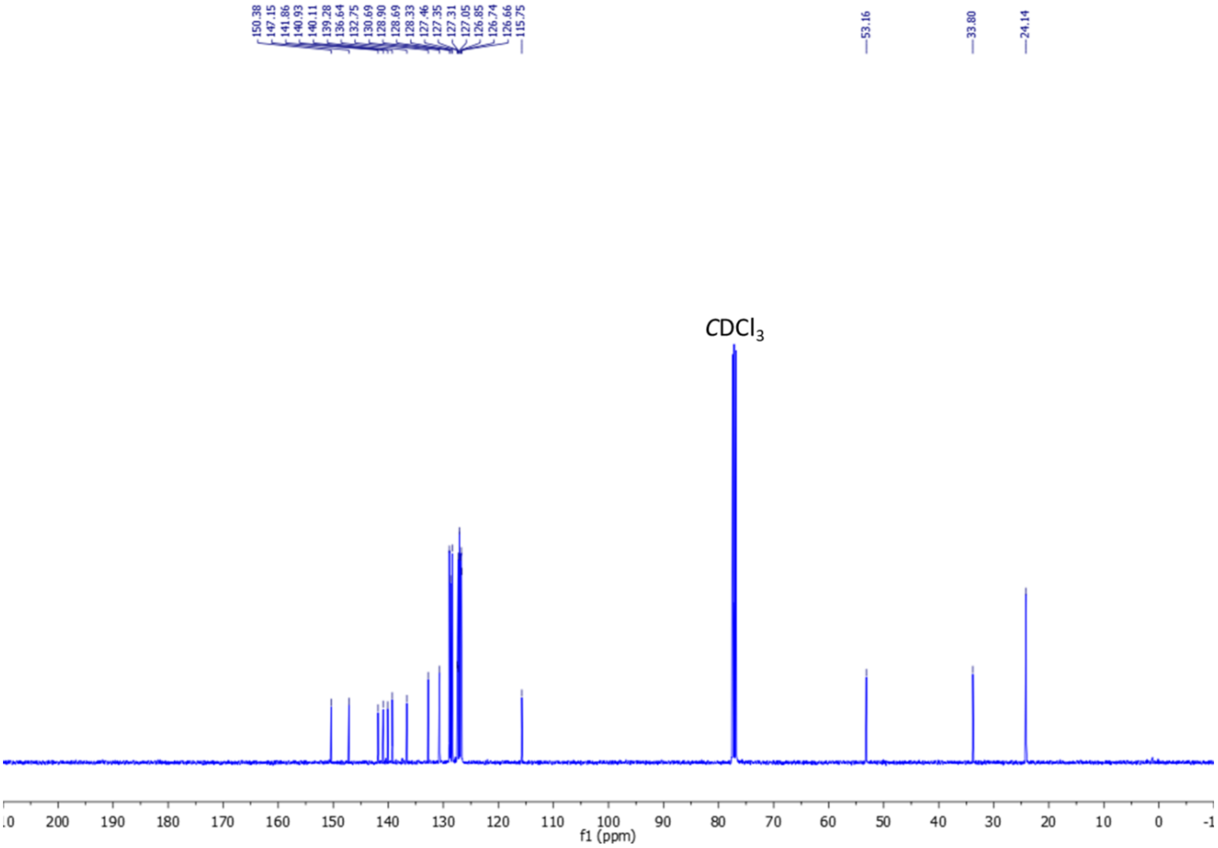

Figure SI 160: ^{13}C NMR spectrum of (*E*)-1-(4-biphenyl)-3-(*para*-*iso*-propylphenyl)-3-phenyl-penta-1,4-diene **9** (101 MHz, chloroform-*d*).

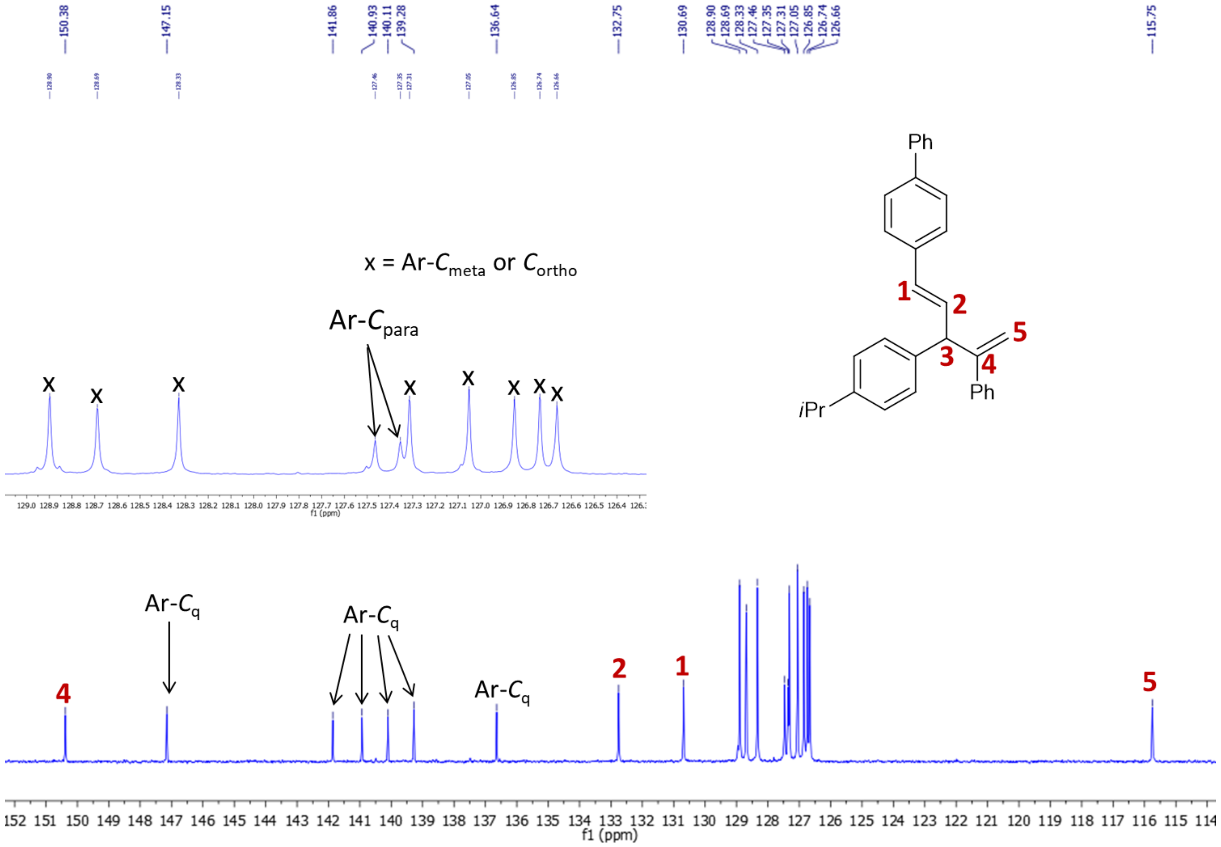

Figure SI 161: Excerpt of the ^{13}C NMR spectrum of (*E*)-1-(4-biphenyl)-3-(*para*-*iso*-propylphenyl)-3-phenyl-penta-1,4-diene **9** (101 MHz, chloroform-*d*).

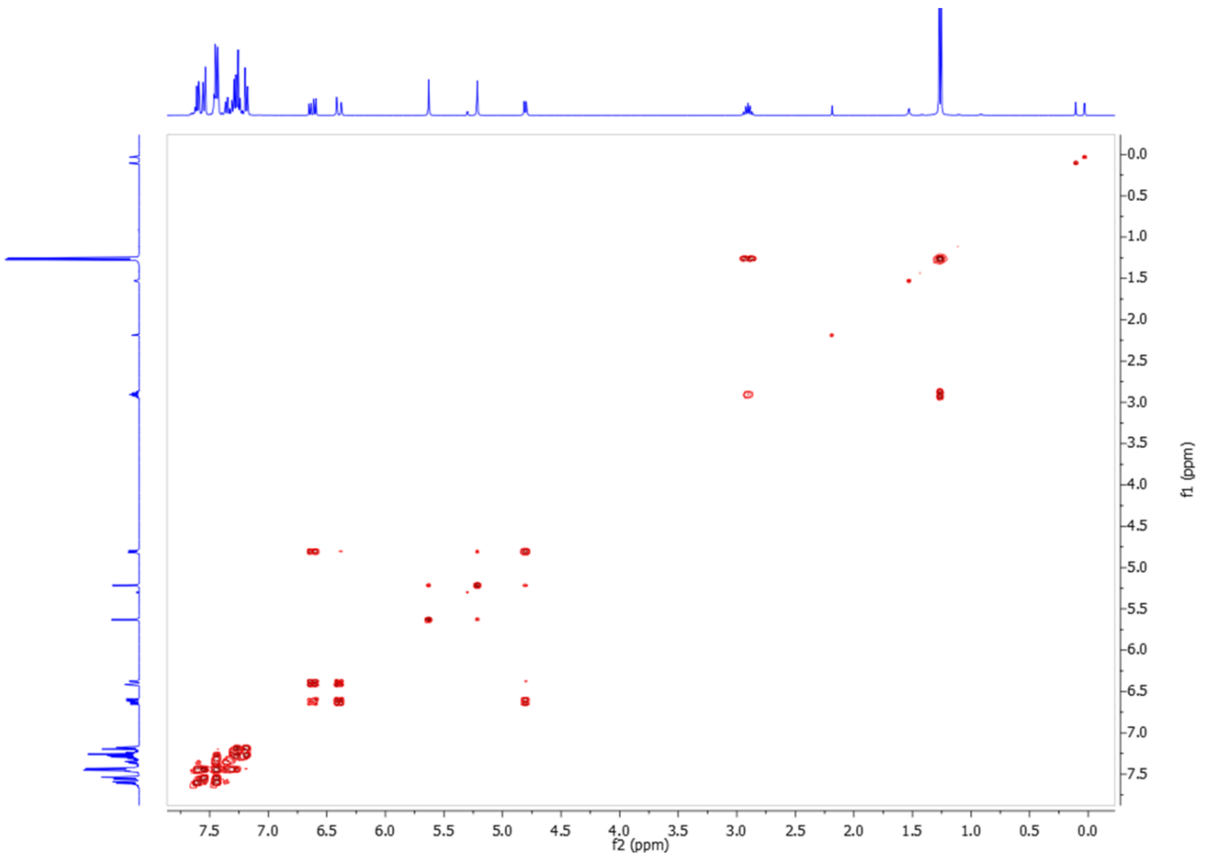

Figure SI 162: HH COSY NMR spectrum of (*E*)-1-(4-biphenyl)-3-(*para*-*iso*-propylphenyl)-3-phenyl-penta-1,4-diene **9** (400 MHz, chloroform-*d*).

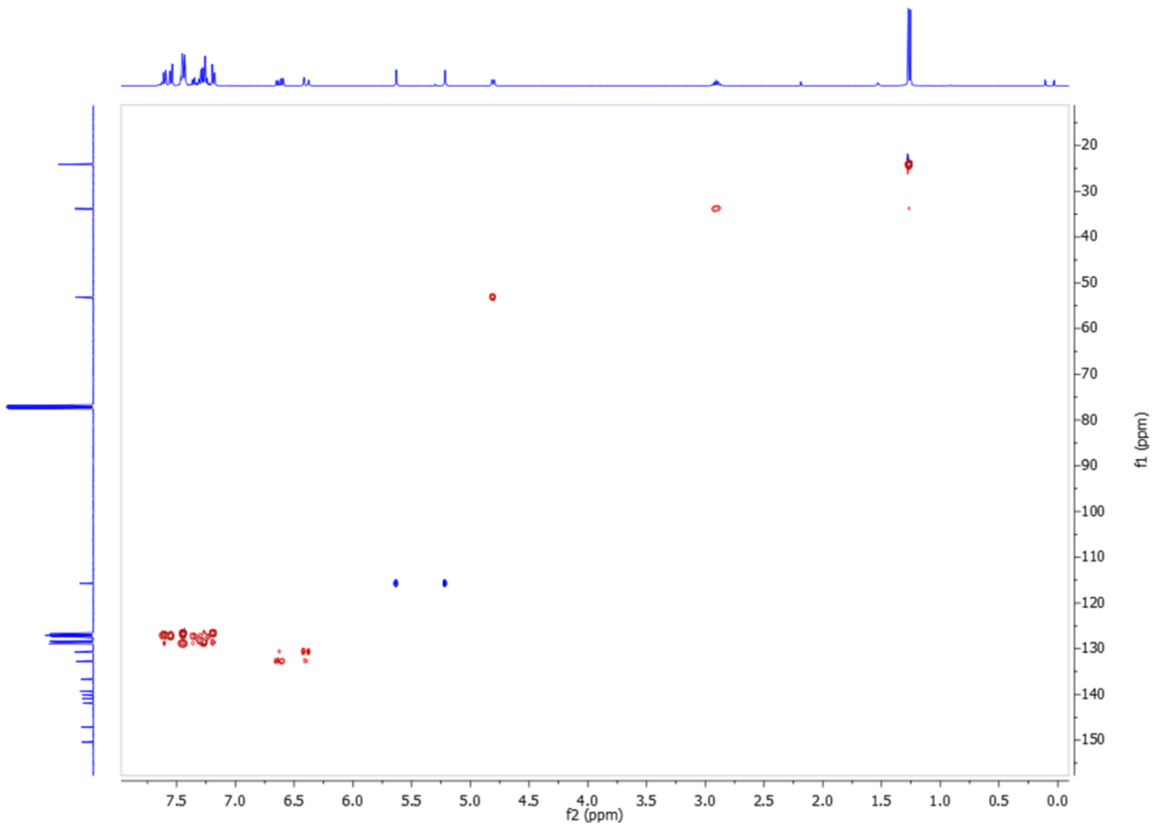

Figure SI 163: HSQC NMR spectrum of (*E*)-1-(4-biphenyl)-3-(*para*-*iso*-propylphenyl)-3-phenyl-penta-1,4-diene **9** (101 MHz, chloroform-*d*).

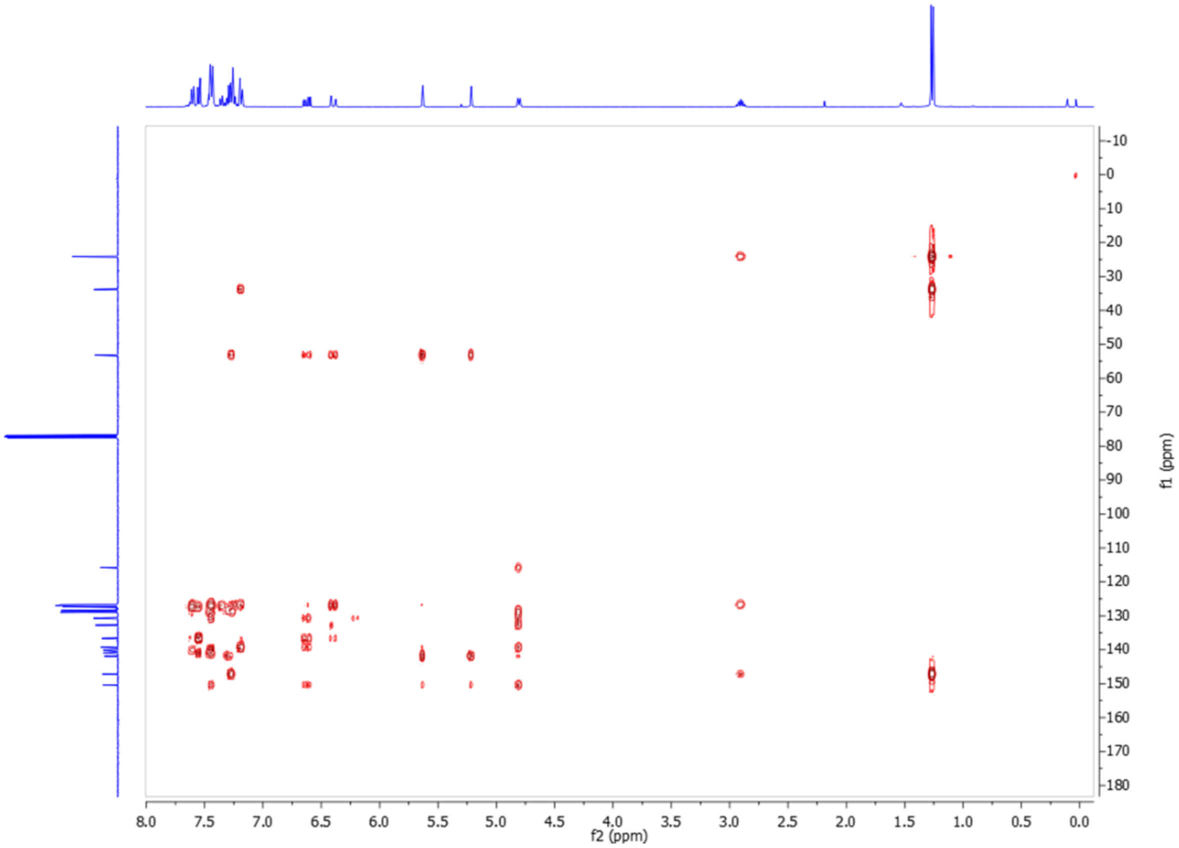

Figure SI 164: HMBC NMR spectrum of (*E*)-1-(4-biphenyl)-3-(*para*-*iso*-propylphenyl)-3-phenyl-penta-1,4-diene **9** (101 MHz, chloroform-*d*).

9.3.20 Bis-1,4-diene 11

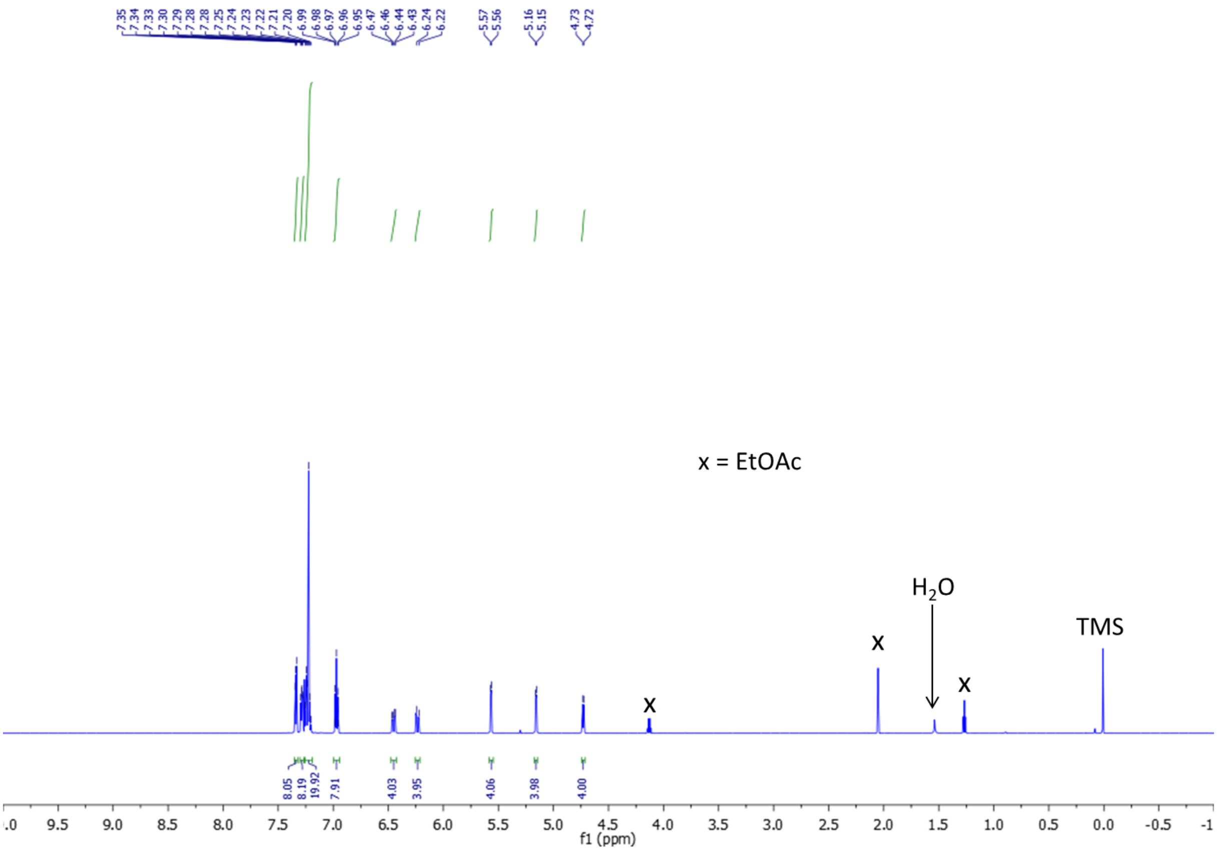

Figure SI 165: ^{1}H NMR spectrum of the two diastereomers of 1,4-bis((*E*)-1-(4-fluorophenyl)-4-phenyl-penta-1,4-dien-3-yl)benzene **11** (700 MHz, chloroform-*d*).

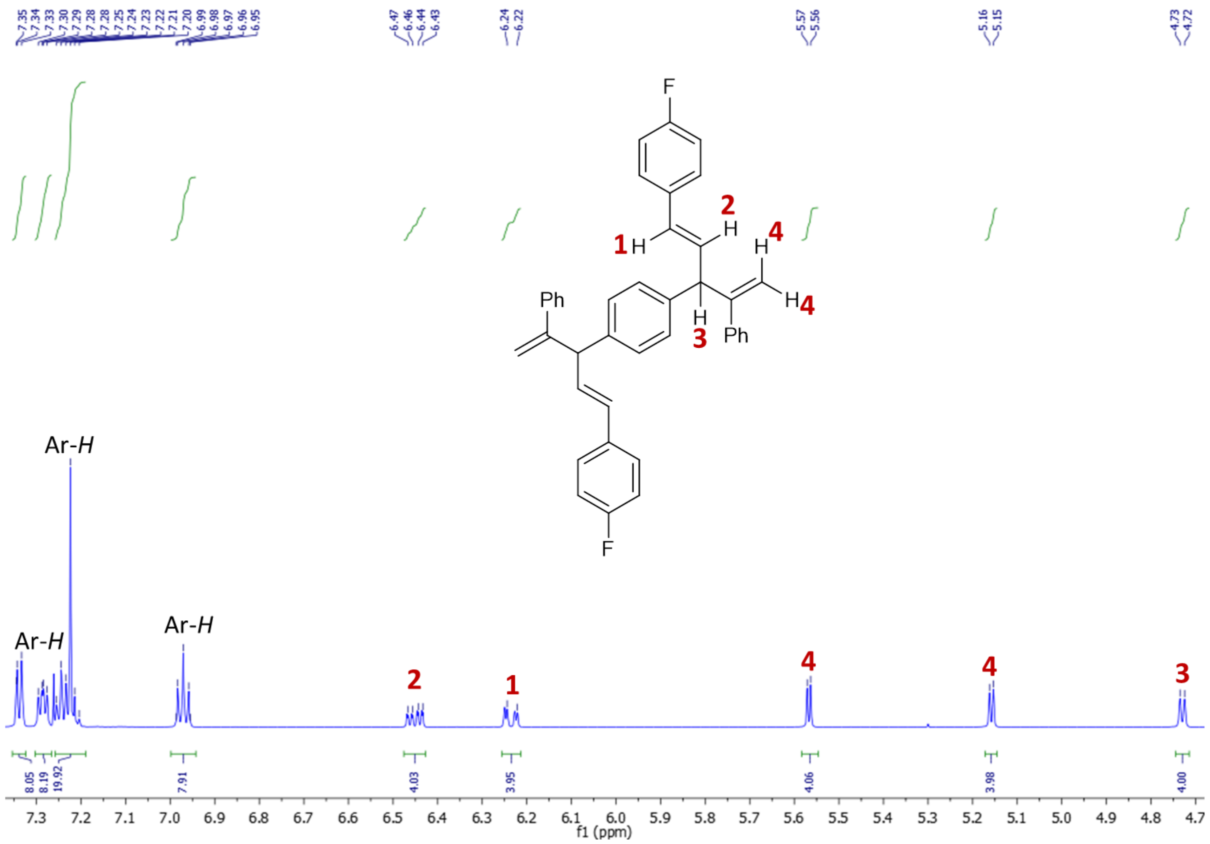

Figure SI 166: Excerpt of ^{1}H NMR spectrum of the two diastereomers of 1,4-bis((*E*)-1-(4-fluorophenyl)-4-phenyl-penta-1,4-dien-3-yl)benzene **11** (700 MHz, chloroform-*d*).

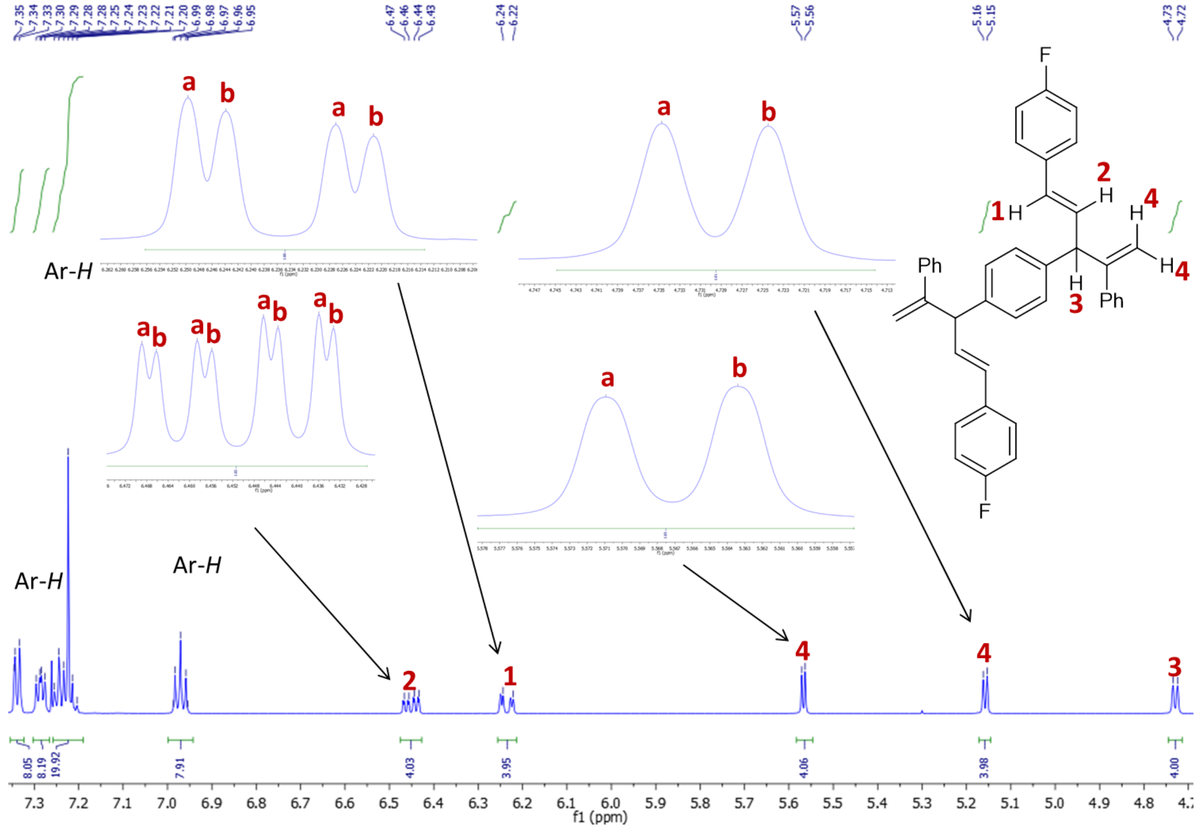

Figure SI 167: Excerpt of the ^{1}H NMR spectrum of the two diastereomers of 1,4-bis((*E*)-1-(4-fluorophenyl)-4-phenyl-penta-1,4-dien-3-yl)benzene **11** with insets of separated signals for both diastereomers (red a and b) (700 MHz, chloroform-*d*).

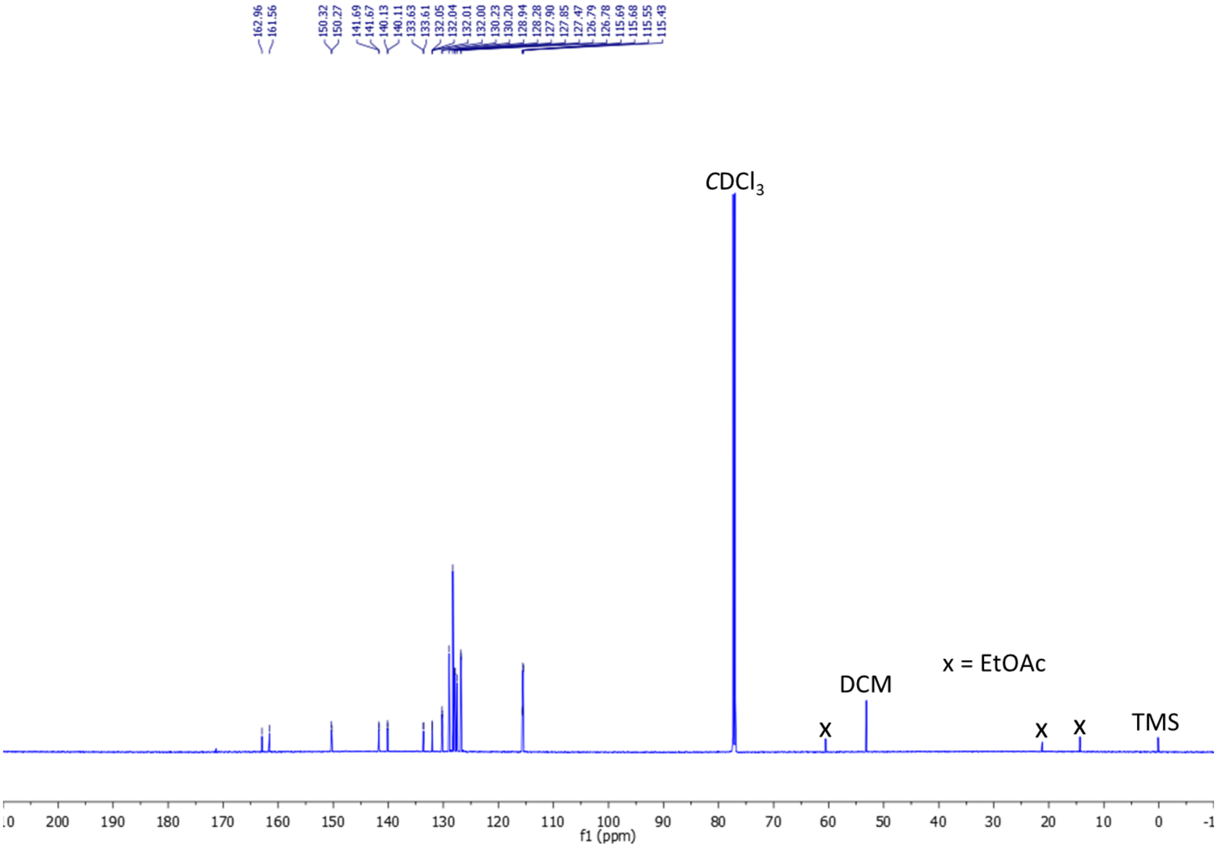

Figure SI 168: ^{13}C NMR spectrum of the two diastereomers of 1,4-bis((*E*)-1-(4-fluorophenyl)-4-phenyl-penta-1,4-dien-3-yl)benzene **11** (176 MHz, chloroform-*d*).

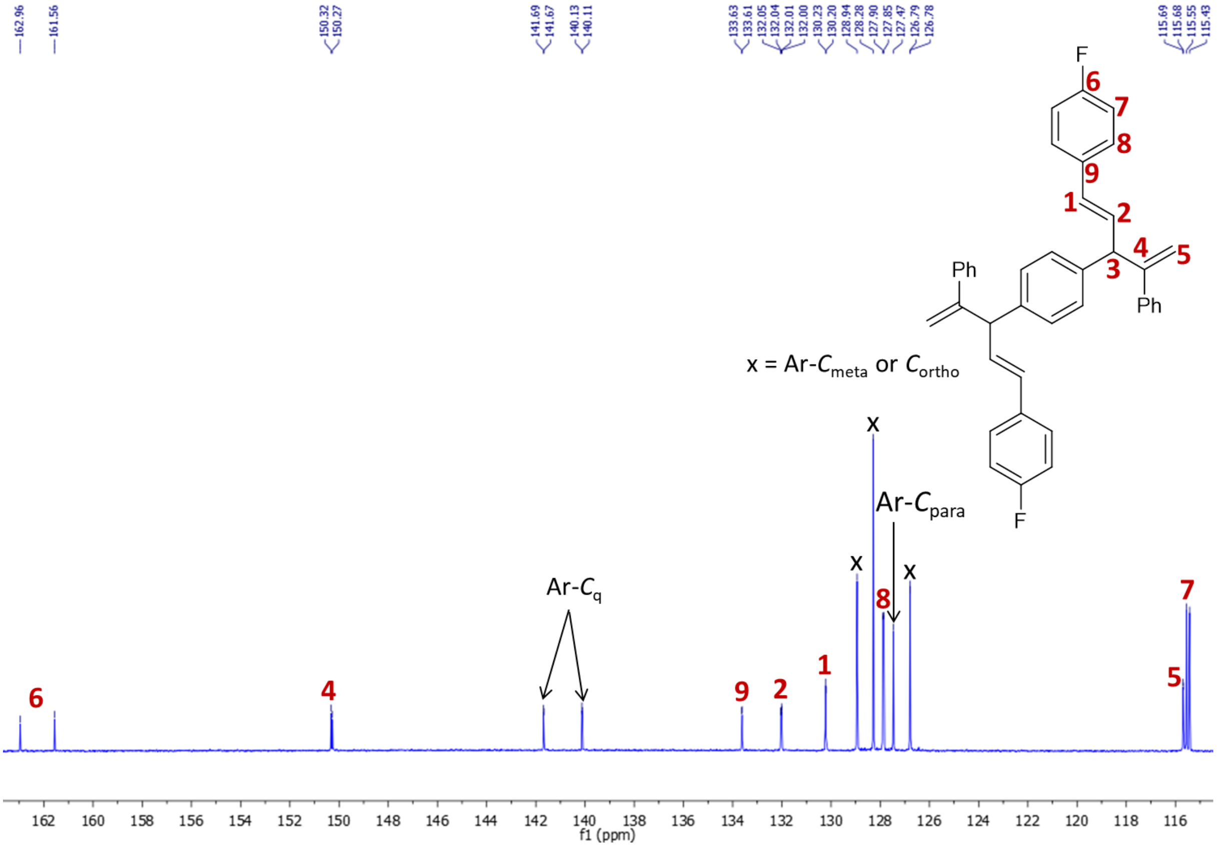

Figure SI 169: Excerpt of the ^{13}C NMR spectrum of the two diastereomers of 1,4-bis((*E*)-1-(4-fluorophenyl)-4-phenyl-penta-1,4-dien-3-yl)benzene **11** (176 MHz, chloroform-*d*).

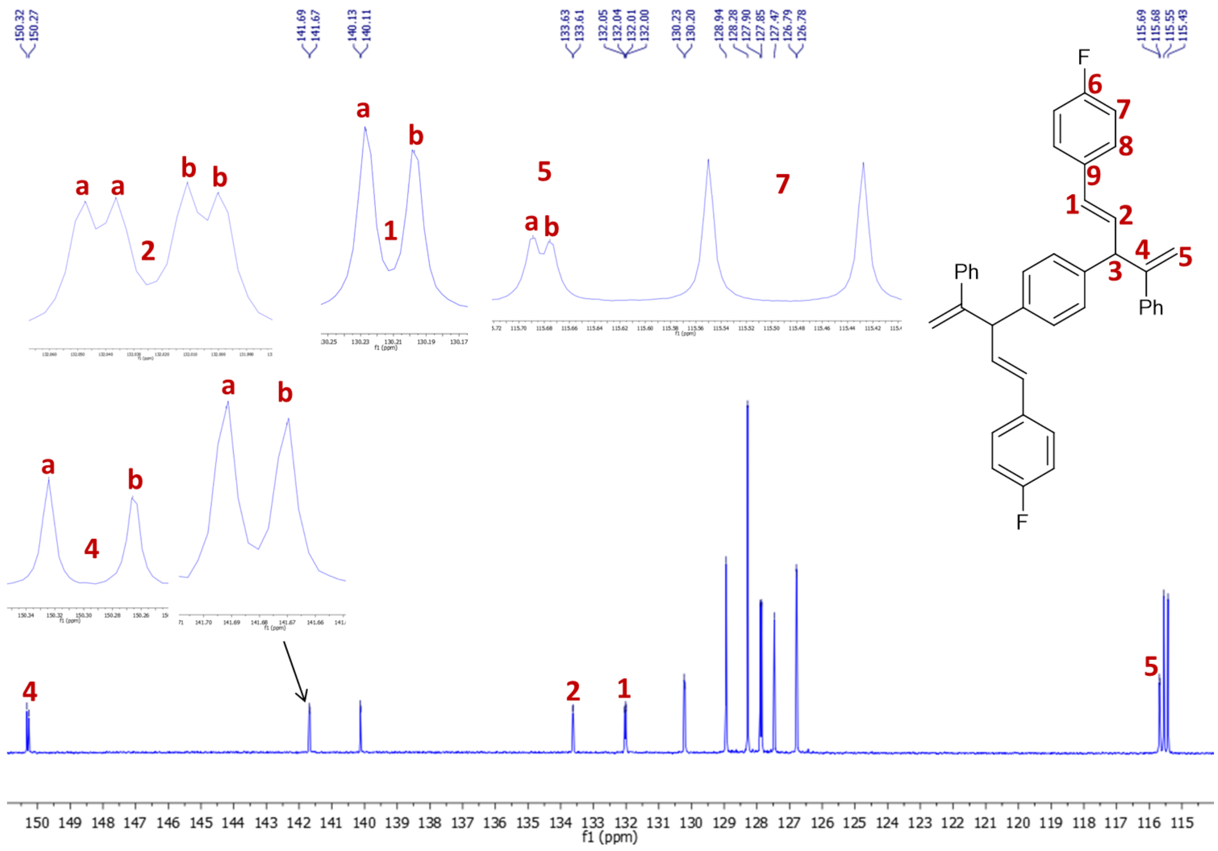

Figure SI 170: Excerpt of the ^{13}C NMR spectrum of the two diastereomers of 1,4-bis((*E*)-1-(4-fluorophenyl)-4-phenyl-penta-1,4-dien-3-yl)benzene **11** with insets of separated signals for both diastereomers (red a and b) (176 MHz, chloroform-*d*).

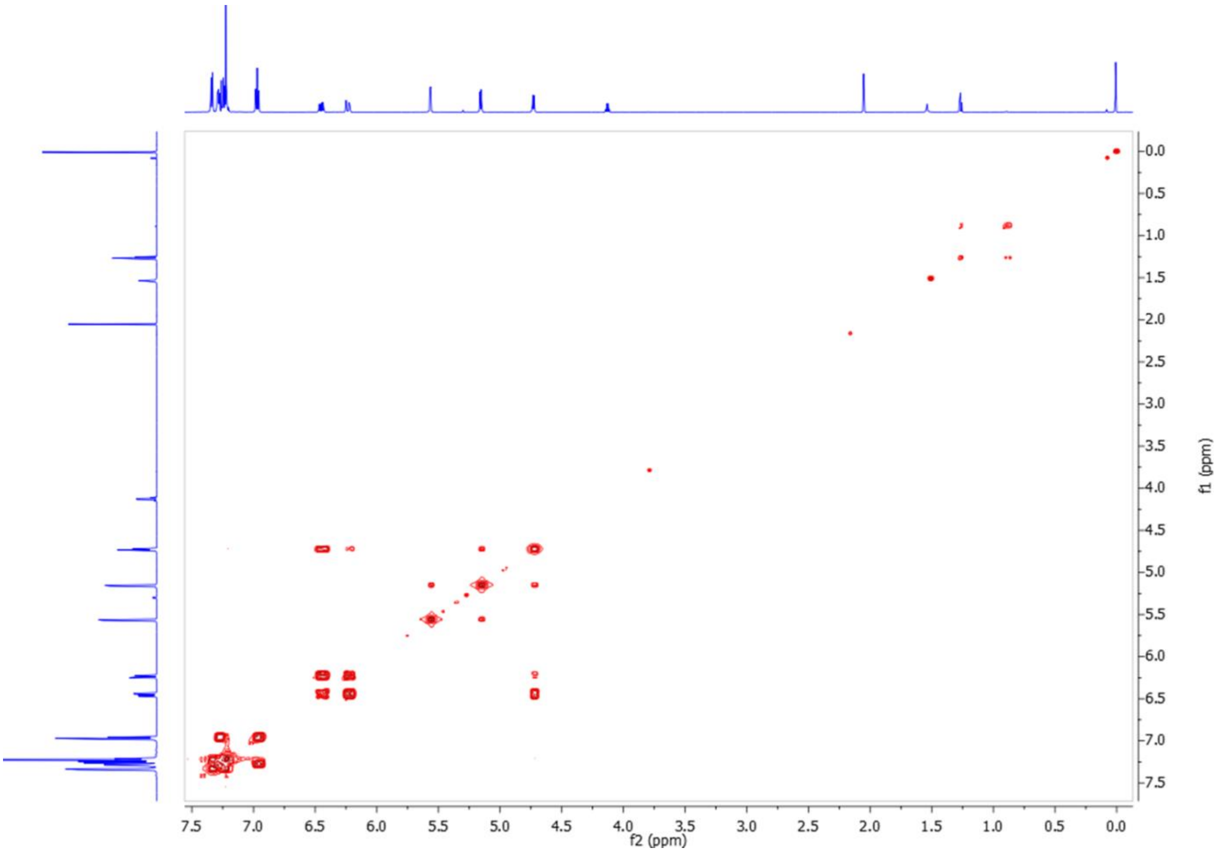

Figure SI 171: HH COSY NMR spectrum of the two diastereomers of 1,4-bis((*E*)-1-(4-fluorophenyl)-4-phenyl-penta-1,4-dien-3-yl)benzene **11** (700 MHz, chloroform-*d*).

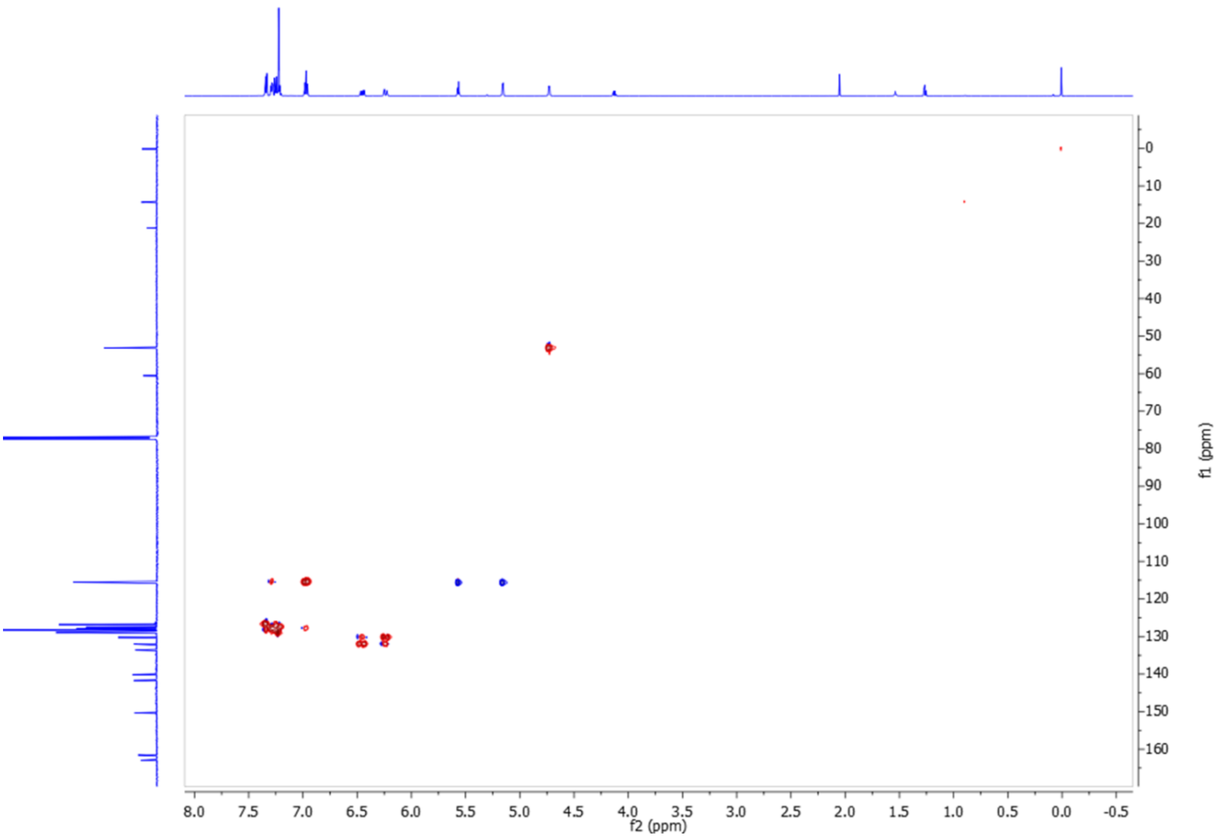

Figure SI 172: HSQC NMR spectrum of the two diastereomers of 1,4-bis((*E*)-1-(4-fluorophenyl)-4-phenyl-penta-1,4-dien-3-yl)benzene **11** (176 MHz, chloroform-*d*).

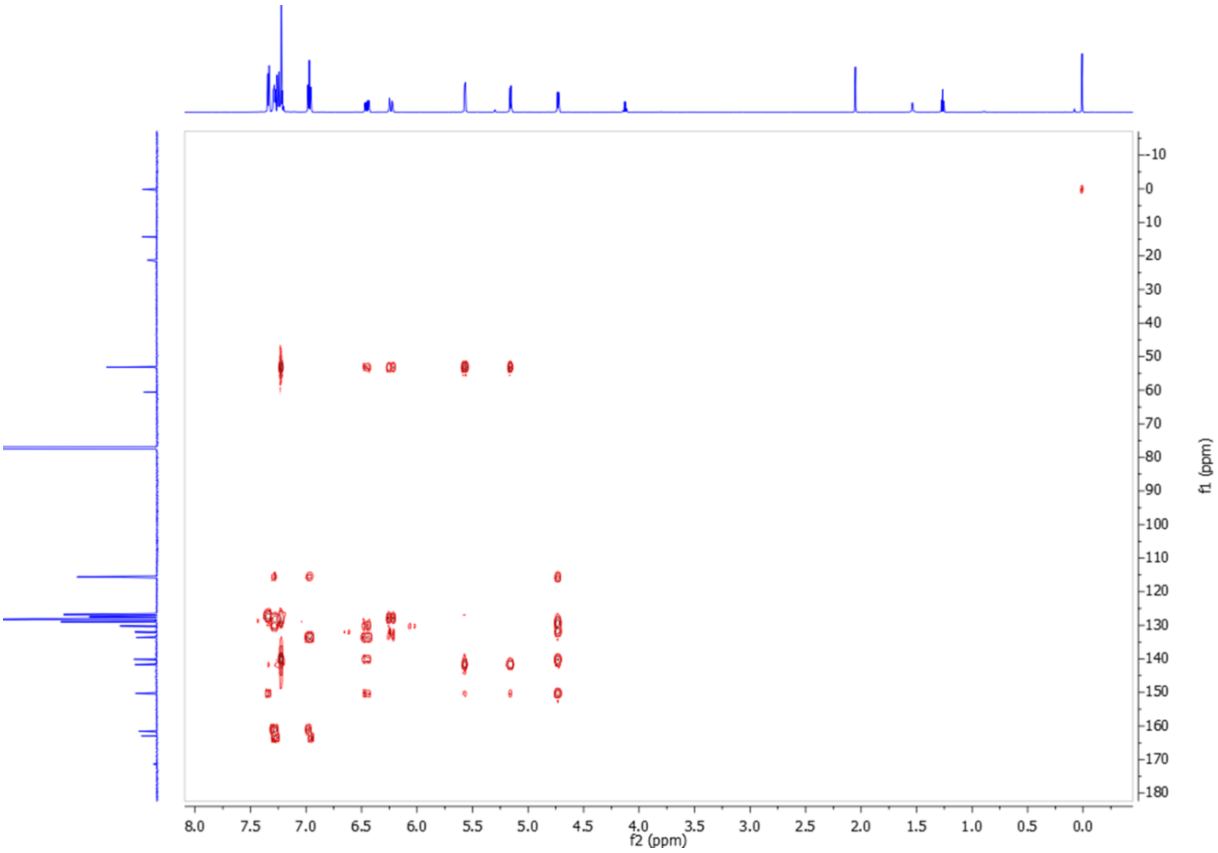

Figure SI 173: HMBC NMR spectrum of the two diastereomers of 1,4-bis((*E*)-1-(4-fluorophenyl)-4-phenyl-penta-1,4-dien-3-yl)benzene **11** (176 MHz, chloroform-*d*).

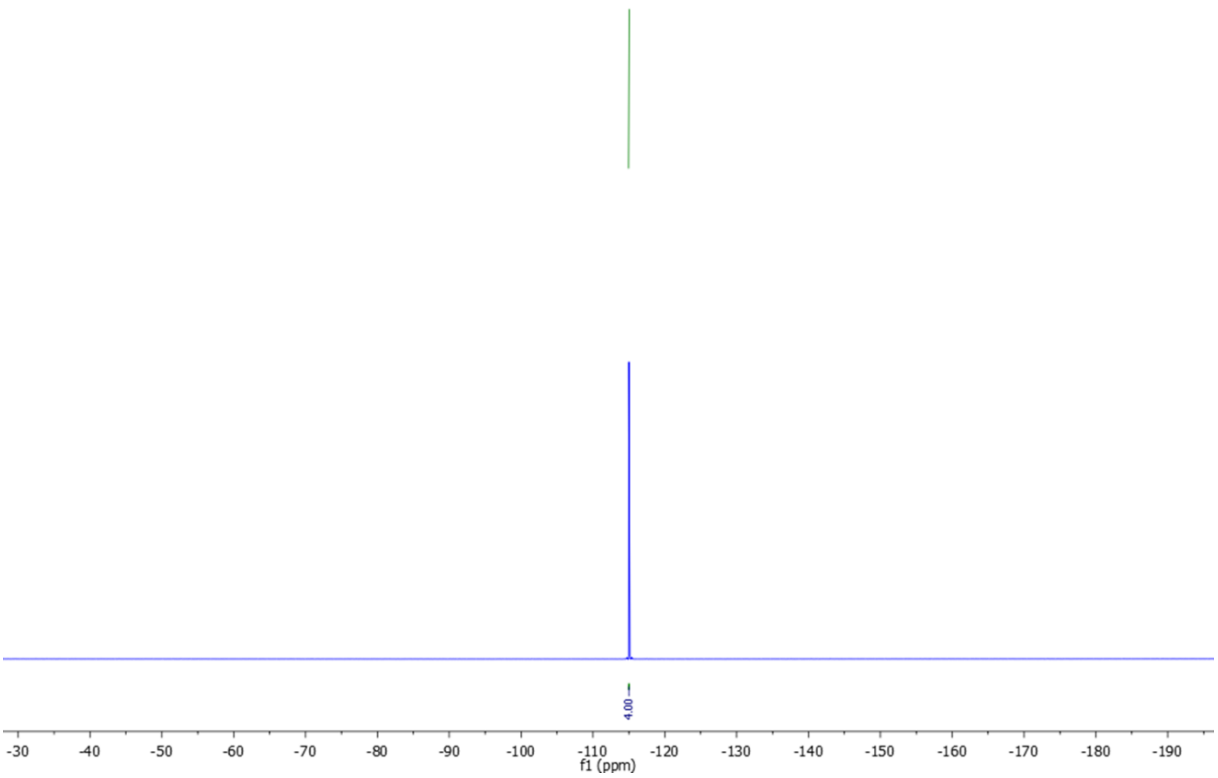

Figure SI 174: ^{19}F NMR spectrum of the two diastereomers of 1,4-bis((*E*)-1-(4-fluorophenyl)-4-phenyl-penta-1,4-dien-3-yl)benzene **11** (377 MHz, chloroform-*d*).

11 AA-FP06-068

Chiralpak IA | 1ml/min | 97% n-Hexan, 3% IPA

Sample Name:	**AA-FP06-068**	*Injection Volume:*	**2,5**
Vial Number:	**11**	*Channel:*	**UV_VIS_2**
Sample Type:	**unknown**	*Wavelength:*	**270**
Control Program:	**M06_F10_A97_B00_C03**	*Bandwidth:*	**1**
Quantif. Method:	**default**	*Dilution Factor:*	**1,0000**
Recording Time:	**7.2.2022 9:24**	*Sample Weight:*	**1,0000**
Run Time (min):	**15,93**	*Sample Amount:*	**1,0000**

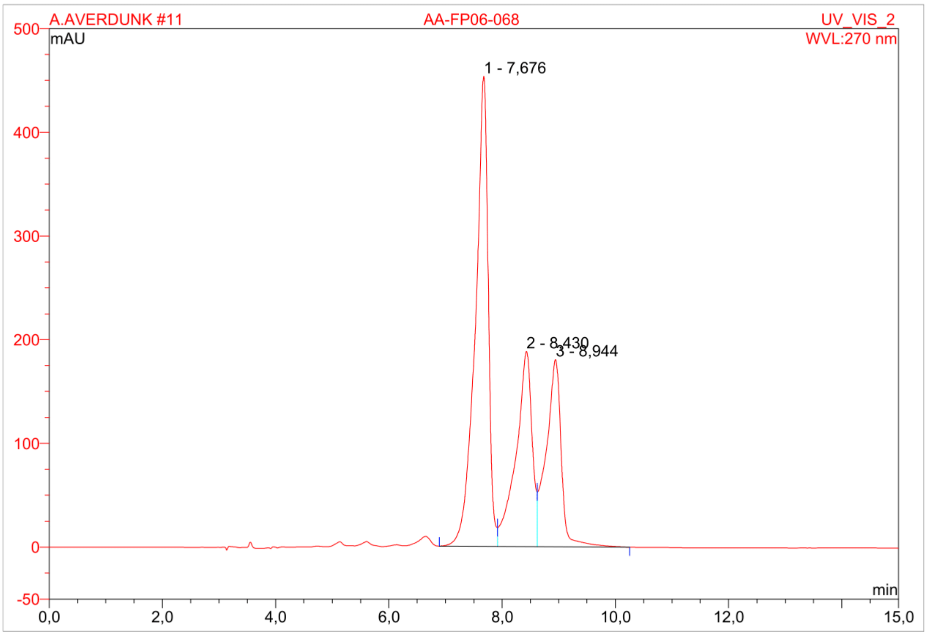

No.	Ret.Time min	Peak Name	Height mAU	Area mAU*min	Rel.Area %	Amount	Type
1	7,68	n.a.	453,053	119,227	50,26	n.a.	BM
2	8,43	n.a.	188,017	62,166	26,21	n.a.	M
3	8,94	n.a.	180,332	55,829	23,53	n.a.	MB
Total:			821,402	237,222	100,00	0,000	

default/Integration

Chromeleon (c) Dionex 1996-2001
Version 6.80 SR15 Build 4656 (243203)

Figure SI 175: Chromatogram after chiral HPLC of **11**.

10 Single crystal X-ray diffraction

Diffraction data for all structures were collected at low temperatures (100K) using ϕ- and ω-scans on a BRUKER D8 Venture system equipped with dual IμS microfocus sources, a PHOTON100 detector and an OXFORD CRYOSYSTEMS 700 low temperature system. Mo-K_α radiation with wavelength 0.71073 Å, Cu-K_α radiation with wavelength 1.54178 Å and a collimating Quazar multilayer mirror were used. Semi-empirical absorption correction from equivalents was applied using SADABS-2016/2.[13] The structures were solved by direct methods using SHELXT2015.[14] and refinement was performed against F^2 on all data by full-matrix least squares using SHELXL2019/1.[15] All non-hydrogen atoms were refined anisotropically and C-H hydrogen atoms were positioned at geometrically calculated positions and refined using a riding model. The isotropic displacement parameters of all hydrogen atoms were fixed to 1.2x or 1.5x (CH_3 hydrogen atoms) the U_{eq} value of the atoms they are linked to. The crystallographic data have been deposited with the Cambridge Crystallographic Data Centre as CCDC No. 2150152 - 2150156 and can be obtained free of charge (https://www.ccdc.cam.ac.uk/structures/).

10.1 Pyridine adduct 4

The crystal structure of **4** was solved in the monoclinic space group $P2_1/n$ and refined as non-merohedral twin. The twin ratio was refined and converged to 0.534(2). The asymmetric unit contains one full molecule of **4**.

Table SI 1: Crystal data and structure refinement for pyridine adduct **4**.

CCDC No	2150152	
Empirical formula	C_{34} H_{20} B F_{10} N	
Formula weight	643.32	
Temperature	100(2) K	
Wavelength	1.54178 Å	
Crystal system	Monoclinic	
Space group	$P2_1/n$	
Unit cell dimensions	a = 9.7735(6) Å	α= 90°.
	b = 9.6307(6) Å	β= 91.559(3)°.
	c = 30.0478(17) Å	γ = 90°.
Volume	2827.2(3) Å^3	
Z	4	
Density (calculated)	1.511 Mg/m^3	
Absorption coefficient	1.169 mm^{-1}	
F(000)	1304	
Crystal size	0.448 x 0.158 x 0.068 mm^3	
Theta range for data collection	2.942 to 70.057°.	
Index ranges	-11 ≤ h ≤ 11, 0 ≤ k ≤ 11, 0 ≤ l ≤ 36	

Reflections collected	5417
Independent reflections	5417 (merged data)
Completeness to theta = 67.679°	87.5 %
Absorption correction	Semi-empirical from equivalents
Refinement method	Full-matrix least-squares on F^2
Data / restraints / parameters	5417 / 0 / 416
Goodness-of-fit on F^2	1.174
Final R indices [I>2σ(I)]	R1 = 0.0678, wR2 = 0.1973
R indices (all data)	R1 = 0.0742, wR2 = 0.2025
Largest diff. peak and hole	0.475 and -0.347 e.Å^{-3}

Table SI 2: Atomic coordinates (x 10^4) and equivalent isotropic displacement parameters (Å^{2}x 10^3) For pyridine adduct **4**. U(eq) is defined as one third of the trace of the orthogonalized U^{ij} tensor.

	x	y	z	U(eq)
F(31)	8275(3)	1743(3)	1329(1)	22(1)
F(32)	9915(3)	531(3)	1933(1)	26(1)
F(33)	11016(3)	2021(3)	2626(1)	27(1)
F(34)	10380(4)	4769(3)	2703(1)	28(1)
F(35)	8646(3)	5963(3)	2141(1)	25(1)
F(41)	5819(3)	4499(3)	2113(1)	24(1)
F(42)	3660(4)	2933(4)	2310(1)	30(1)
F(43)	2556(3)	1158(4)	1696(1)	29(1)
F(44)	3657(3)	973(3)	877(1)	28(1)
F(45)	5793(3)	2503(3)	670(1)	23(1)
N(21)	6992(4)	6159(5)	1377(1)	16(1)
C(1)	7905(5)	4484(5)	798(2)	18(1)
C(2)	9127(5)	3997(6)	703(2)	20(1)
C(3)	7017(6)	5135(5)	414(2)	18(1)
C(4)	7277(6)	6685(6)	357(2)	20(1)
C(5)	8589(6)	7201(6)	305(2)	23(1)
C(6)	8797(7)	8628(6)	258(2)	27(1)
C(7)	7716(7)	9537(6)	272(2)	31(1)
C(8)	6403(7)	9045(6)	322(2)	28(1)
C(9)	6192(6)	7614(6)	360(2)	23(1)
C(10)	7193(5)	4316(6)	-12(2)	20(1)
C(11)	7483(5)	4822(6)	-411(2)	21(1)

C(12)	7737(5)	3965(6)	-810(2)	21(1)
C(13)	7946(5)	2543(6)	-791(2)	22(1)
C(14)	8223(5)	1778(6)	-1170(2)	24(1)
C(15)	8281(5)	2445(7)	-1584(2)	24(1)
C(16)	8059(6)	3855(7)	-1606(2)	26(1)
C(17)	7799(5)	4617(6)	-1227(2)	21(1)
C(21)	8048(6)	7045(6)	1347(2)	18(1)
C(22)	7890(6)	8456(6)	1394(2)	21(1)
C(23)	6607(6)	8980(6)	1466(2)	24(1)
C(24)	5513(6)	8088(6)	1486(2)	22(1)
C(25)	5736(6)	6684(5)	1437(2)	18(1)
C(31)	8413(5)	3945(5)	1682(2)	18(1)
C(32)	8781(5)	2554(6)	1663(2)	19(1)
C(33)	9634(5)	1891(5)	1972(2)	18(1)
C(34)	10178(5)	2628(6)	2327(2)	22(1)
C(35)	9846(6)	4005(6)	2362(2)	21(1)
C(36)	8952(6)	4626(6)	2054(2)	20(1)
C(41)	5906(5)	3626(5)	1377(2)	18(1)
C(42)	5299(5)	3639(5)	1792(2)	18(1)
C(43)	4205(5)	2847(6)	1908(2)	22(1)
C(44)	3633(5)	1936(5)	1597(2)	19(1)
C(45)	4194(5)	1859(5)	1182(2)	20(1)
C(46)	5298(5)	2683(5)	1080(2)	18(1)
B(1)	7302(6)	4525(6)	1300(2)	17(1)

Table SI 3: Bond lengths [Å] and angles [°] for pyridine adduct **4**.

F(31)-C(32)	1.355(6)	F(45)-C(46)	1.347(6)
F(32)-C(33)	1.344(6)	N(21)-C(25)	1.343(7)
F(33)-C(34)	1.334(6)	N(21)-C(21)	1.344(7)
F(34)-C(35)	1.354(6)	N(21)-B(1)	1.621(7)
F(35)-C(36)	1.349(6)	C(1)-C(2)	1.322(8)
F(41)-C(42)	1.361(6)	C(1)-C(3)	1.555(7)
F(42)-C(43)	1.337(6)	C(1)-B(1)	1.634(7)
F(43)-C(44)	1.332(6)	C(2)-H(2A)	0.9500
F(44)-C(45)	1.348(6)	C(2)-H(2B)	0.9500

C(3)-C(10)	1.520(7)	C(31)-C(32)	1.389(8)
C(3)-C(4)	1.525(7)	C(31)-B(1)	1.657(8)
C(3)-H(3)	1.0000	C(32)-C(33)	1.385(7)
C(4)-C(5)	1.388(8)	C(33)-C(34)	1.378(7)
C(4)-C(9)	1.388(8)	C(34)-C(35)	1.370(8)
C(5)-C(6)	1.397(8)	C(35)-C(36)	1.390(7)
C(5)-H(5)	0.9500	C(41)-C(42)	1.395(7)
C(6)-C(7)	1.374(9)	C(41)-C(46)	1.394(7)
C(6)-H(6)	0.9500	C(41)-B(1)	1.638(8)
C(7)-C(8)	1.381(9)	C(42)-C(43)	1.366(8)
C(7)-H(7)	0.9500	C(43)-C(44)	1.388(8)
C(8)-C(9)	1.398(8)	C(44)-C(45)	1.378(7)
C(8)-H(8)	0.9500	C(45)-C(46)	1.381(7)
C(9)-H(9)	0.9500		
C(10)-C(11)	1.330(8)	C(25)-N(21)-C(21)	118.4(4)
C(10)-H(10)	0.9500	C(25)-N(21)-B(1)	124.0(4)
C(11)-C(12)	1.483(7)	C(21)-N(21)-B(1)	117.4(4)
C(11)-H(11)	0.9500	C(2)-C(1)-C(3)	118.3(5)
C(12)-C(13)	1.386(8)	C(2)-C(1)-B(1)	123.9(5)
C(12)-C(17)	1.403(7)	C(3)-C(1)-B(1)	117.8(4)
C(13)-C(14)	1.388(8)	C(1)-C(2)-H(2A)	120.0
C(13)-H(13)	0.9500	C(1)-C(2)-H(2B)	120.0
C(14)-C(15)	1.402(8)	H(2A)-C(2)-H(2B)	120.0
C(14)-H(14)	0.9500	C(10)-C(3)-C(4)	112.9(4)
C(15)-C(16)	1.377(9)	C(10)-C(3)-C(1)	110.0(4)
C(15)-H(15)	0.9500	C(4)-C(3)-C(1)	112.8(4)
C(16)-C(17)	1.385(8)	C(10)-C(3)-H(3)	106.9
C(16)-H(16)	0.9500	C(4)-C(3)-H(3)	106.9
C(17)-H(17)	0.9500	C(1)-C(3)-H(3)	106.9
C(21)-C(22)	1.374(8)	C(5)-C(4)-C(9)	118.6(5)
C(21)-H(21)	0.9500	C(5)-C(4)-C(3)	121.4(5)
C(22)-C(23)	1.375(8)	C(9)-C(4)-C(3)	120.0(5)
C(22)-H(22)	0.9500	C(4)-C(5)-C(6)	120.1(5)
C(23)-C(24)	1.375(8)	C(4)-C(5)-H(5)	120.0
C(23)-H(23)	0.9500	C(6)-C(5)-H(5)	120.0
C(24)-C(25)	1.378(8)	C(7)-C(6)-C(5)	120.6(6)
C(24)-H(24)	0.9500	C(7)-C(6)-H(6)	119.7
C(25)-H(25)	0.9500	C(5)-C(6)-H(6)	119.7
C(31)-C(36)	1.386(7)	C(6)-C(7)-C(8)	120.2(5)

C(6)-C(7)-H(7)	119.9	C(24)-C(23)-H(23)	120.3
C(8)-C(7)-H(7)	119.9	C(22)-C(23)-H(23)	120.3
C(7)-C(8)-C(9)	119.1(6)	C(23)-C(24)-C(25)	118.9(5)
C(7)-C(8)-H(8)	120.5	C(23)-C(24)-H(24)	120.6
C(9)-C(8)-H(8)	120.5	C(25)-C(24)-H(24)	120.6
C(4)-C(9)-C(8)	121.4(6)	N(21)-C(25)-C(24)	122.1(5)
C(4)-C(9)-H(9)	119.3	N(21)-C(25)-H(25)	118.9
C(8)-C(9)-H(9)	119.3	C(24)-C(25)-H(25)	118.9
C(11)-C(10)-C(3)	127.0(5)	C(36)-C(31)-C(32)	113.4(5)
C(11)-C(10)-H(10)	116.5	C(36)-C(31)-B(1)	128.8(5)
C(3)-C(10)-H(10)	116.5	C(32)-C(31)-B(1)	117.5(4)
C(10)-C(11)-C(12)	124.7(5)	F(31)-C(32)-C(33)	115.5(5)
C(10)-C(11)-H(11)	117.7	F(31)-C(32)-C(31)	119.8(4)
C(12)-C(11)-H(11)	117.7	C(33)-C(32)-C(31)	124.7(5)
C(13)-C(12)-C(17)	118.0(5)	F(32)-C(33)-C(34)	119.6(5)
C(13)-C(12)-C(11)	123.0(5)	F(32)-C(33)-C(32)	120.8(5)
C(17)-C(12)-C(11)	119.1(5)	C(34)-C(33)-C(32)	119.6(5)
C(12)-C(13)-C(14)	121.5(5)	F(33)-C(34)-C(35)	121.0(5)
C(12)-C(13)-H(13)	119.3	F(33)-C(34)-C(33)	120.9(5)
C(14)-C(13)-H(13)	119.3	C(35)-C(34)-C(33)	118.1(5)
C(13)-C(14)-C(15)	119.9(5)	F(34)-C(35)-C(34)	119.8(5)
C(13)-C(14)-H(14)	120.1	F(34)-C(35)-C(36)	119.4(5)
C(15)-C(14)-H(14)	120.1	C(34)-C(35)-C(36)	120.8(5)
C(16)-C(15)-C(14)	119.0(5)	F(35)-C(36)-C(31)	121.7(5)
C(16)-C(15)-H(15)	120.5	F(35)-C(36)-C(35)	114.9(5)
C(14)-C(15)-H(15)	120.5	C(31)-C(36)-C(35)	123.4(5)
C(15)-C(16)-C(17)	121.0(5)	C(42)-C(41)-C(46)	113.2(5)
C(15)-C(16)-H(16)	119.5	C(42)-C(41)-B(1)	119.8(4)
C(17)-C(16)-H(16)	119.5	C(46)-C(41)-B(1)	126.5(5)
C(16)-C(17)-C(12)	120.7(5)	F(41)-C(42)-C(43)	115.9(5)
C(16)-C(17)-H(17)	119.6	F(41)-C(42)-C(41)	118.7(4)
C(12)-C(17)-H(17)	119.6	C(43)-C(42)-C(41)	125.3(5)
N(21)-C(21)-C(22)	122.1(5)	F(42)-C(43)-C(42)	122.0(5)
N(21)-C(21)-H(21)	118.9	F(42)-C(43)-C(44)	119.0(5)
C(22)-C(21)-H(21)	118.9	C(42)-C(43)-C(44)	118.9(5)
C(21)-C(22)-C(23)	119.0(5)	F(43)-C(44)-C(45)	120.6(5)
C(21)-C(22)-H(22)	120.5	F(43)-C(44)-C(43)	120.7(5)
C(23)-C(22)-H(22)	120.5	C(45)-C(44)-C(43)	118.7(5)
C(24)-C(23)-C(22)	119.4(5)	F(44)-C(45)-C(46)	120.3(5)

F(44)-C(45)-C(44)	119.5(5)	N(21)-B(1)-C(41)	109.4(4)
C(46)-C(45)-C(44)	120.3(5)	C(1)-B(1)-C(41)	116.1(4)
F(45)-C(46)-C(45)	115.6(4)	N(21)-B(1)-C(31)	110.4(4)
F(45)-C(46)-C(41)	120.8(5)	C(1)-B(1)-C(31)	112.7(4)
C(45)-C(46)-C(41)	123.6(5)	C(41)-B(1)-C(31)	104.9(4)
N(21)-B(1)-C(1)	103.3(4)		

Table SI 4: Anisotropic displacement parameters ($Å^2 x 10^3$) for pyridine adduct **4**. The anisotropic displacement factor exponent takes the form: $-2p^2[h^2 a^{*2}U^{11} + ... + 2 h k a^* b^* U^{12}]$

	U^{11}	U^{22}	U^{33}	U^{23}	U^{13}	U^{12}
F(31)	25(2)	17(1)	24(2)	-3(1)	-6(1)	-4(1)
F(32)	28(2)	18(2)	30(2)	2(1)	-4(1)	2(1)
F(33)	25(2)	32(2)	25(2)	6(1)	-9(1)	-1(2)
F(34)	38(2)	28(2)	19(1)	-3(1)	-9(1)	-8(2)
F(35)	36(2)	16(1)	23(2)	-4(1)	-5(1)	-1(1)
F(41)	29(2)	25(2)	18(1)	-4(1)	2(1)	-8(2)
F(42)	33(2)	36(2)	22(2)	-2(1)	11(1)	-10(2)
F(43)	24(2)	31(2)	32(2)	0(1)	6(1)	-14(2)
F(44)	30(2)	27(2)	28(2)	-7(1)	2(1)	-14(2)
F(45)	25(2)	26(2)	20(1)	-4(1)	4(1)	-9(1)
N(21)	16(2)	17(2)	14(2)	2(2)	0(2)	-3(2)
C(1)	22(3)	14(2)	18(2)	0(2)	-2(2)	-6(2)
C(2)	19(3)	22(3)	18(2)	0(2)	1(2)	-3(2)
C(3)	15(3)	22(3)	18(2)	1(2)	2(2)	0(2)
C(4)	21(3)	26(3)	13(2)	1(2)	-1(2)	0(2)
C(5)	24(3)	24(3)	21(2)	1(2)	4(2)	0(2)
C(6)	30(3)	27(3)	25(3)	3(2)	4(2)	-9(3)
C(7)	46(4)	22(3)	25(3)	7(2)	-4(3)	1(3)
C(8)	34(3)	29(3)	21(3)	1(2)	-4(2)	12(3)
C(9)	20(3)	30(3)	19(2)	0(2)	-2(2)	-1(3)
C(10)	13(2)	24(3)	23(3)	-2(2)	1(2)	-3(2)
C(11)	16(3)	21(3)	26(3)	0(2)	0(2)	-1(2)
C(12)	15(3)	30(3)	17(2)	-2(2)	-2(2)	-4(2)
C(13)	20(3)	27(3)	20(2)	0(2)	0(2)	-6(2)
C(14)	16(3)	27(3)	28(3)	-5(2)	-2(2)	-2(2)

C(15)	14(3)	37(3)	22(2)	-6(2)	2(2)	-7(2)
C(16)	25(3)	40(3)	14(2)	6(2)	-1(2)	-8(3)
C(17)	13(3)	23(3)	26(3)	2(2)	-4(2)	-7(2)
C(21)	14(3)	24(3)	15(2)	2(2)	1(2)	-2(2)
C(22)	17(3)	21(3)	24(3)	0(2)	-2(2)	-7(2)
C(23)	25(3)	20(3)	26(3)	2(2)	1(2)	0(2)
C(24)	18(3)	23(3)	26(3)	-2(2)	0(2)	1(2)
C(25)	15(3)	20(3)	19(2)	1(2)	-1(2)	0(2)
C(31)	15(3)	19(2)	19(2)	1(2)	1(2)	-5(2)
C(32)	19(3)	20(3)	17(2)	-2(2)	-2(2)	-7(2)
C(33)	12(3)	14(2)	26(2)	-1(2)	1(2)	-3(2)
C(34)	17(3)	29(3)	21(2)	4(2)	-5(2)	-1(2)
C(35)	21(3)	25(3)	17(2)	-1(2)	-1(2)	-9(2)
C(36)	21(3)	18(2)	21(2)	-1(2)	2(2)	0(2)
C(41)	15(3)	15(2)	23(2)	1(2)	-3(2)	-3(2)
C(42)	18(3)	15(2)	21(2)	-2(2)	-4(2)	-2(2)
C(43)	18(3)	24(3)	23(3)	1(2)	5(2)	1(2)
C(44)	13(2)	15(2)	29(3)	3(2)	1(2)	-3(2)
C(45)	18(3)	16(2)	26(3)	-2(2)	-6(2)	-4(2)
C(46)	16(3)	18(2)	21(2)	0(2)	0(2)	1(2)
B(1)	15(3)	17(3)	20(3)	-1(2)	1(2)	-4(2)

Table SI 5: Hydrogen coordinates (x 10^4) and isotropic displacement parameters (Å^{2}x 10^3) for pyridine adduct **4**.

	x	y	z	U(eq)
H(2A)	9442	4049	407	24
H(2B)	9691	3594	931	24
H(3)	6039	5030	498	22
H(5)	9347	6583	302	27
H(6)	9695	8973	217	33
H(7)	7873	10507	247	38
H(8)	5652	9670	331	34
H(9)	5288	7271	389	28
H(10)	7085	3338	7	24
H(11)	7531	5803	-442	25
H(13)	7899	2081	-513	27
H(14)	8373	806	-1149	28
H(15)	8471	1931	-1845	29
H(16)	8085	4313	-1886	31
H(17)	7660	5591	-1249	25
H(21)	8932	6685	1292	21
H(22)	8655	9059	1376	25
H(23)	6478	9951	1503	29
H(24)	4617	8433	1532	27
H(25)	4979	6068	1445	22

10.2 1,4-diene 7d

The crystal structure of **7d** was solved in the orthorhombic space group $P2_12_12_1$. The asymmetric unit contains one full molecule, which was found to be disordered at two positions. The disorder was refined using same distance restraints on 1,2 and 1,3 distances, similarity restraints on anisotropic displacement parameters, by setting two atoms with very similar coordinates to have the same anisotropic displacement parameters[16] and by using advanced rigid bond restraints.[17] The disorder ratios were allowed to refine and converged to 0.797(5) and 0.63(4). The absolute structure was confirmed with a Parsons parameter of 0.059(37).[18] Crystals suitable for SCXRD analysis were obtained by crystallization from a saturated DCM solution layered with *n*-hexane solution at -3 °C.

Table SI 6: Crystal data and structure refinement for 1,4-diene **7d**.

CCDC No	2150154	
Empirical formula	C_{26} H_{25} F	
Formula weight	356.46	
Temperature	100(2) K	
Wavelength	1.54178 Å	
Crystal system	Orthorhombic	
Space group	$P2_12_12_1$	
Unit cell dimensions	a = 9.6273(3) Å	α= 90°.
	b = 9.8026(3) Å	β= 90°.
	c = 21.0243(7) Å	γ = 90°.
Volume	1984.12(11) $Å^3$	
Z	4	
Density (calculated)	1.193 Mg/m^3	
Absorption coefficient	0.575 mm^{-1}	
F(000)	760	
Crystal size	0.365 x 0.239 x 0.123 mm^3	
Theta range for data collection	4.205 to 72.017°.	
Index ranges	-10 ≤ h ≤ 11, -12 ≤ k ≤ 12, -25 ≤ l ≤ 25	
Reflections collected	54873	
Independent reflections	3892 [R(int) = 0.0470]	
Completeness to theta = 67.679°	99.9 %	
Absorption correction	Semi-empirical from equivalents	
Refinement method	Full-matrix least-squares on F^2	
Data / restraints / parameters	3892 / 516 / 353	
Goodness-of-fit on F^2	1.067	
Final R indices [I>2σ(I)]	R1 = 0.0255, wR2 = 0.0627	
R indices (all data)	R1 = 0.0265, wR2 = 0.0634	
Absolute structure parameter	0.06(4)	
Extinction coefficient	0.0033(3)	

Largest diff. peak and hole 0.208 and -0.121 e.Å^{-3}

Table SI 7: Atomic coordinates (x 10^4) and equivalent isotropic displacement parameters (Å^{2}x 10^3) For 1,4-diene **7d**. U(eq) is defined as one third of the trace of the orthogonalized U^{ij} tensor.

	x	y	z	U(eq)
C(1)	4745(2)	4026(2)	8108(1)	25(1)
C(2)	3346(2)	3865(2)	8444(1)	24(1)
C(3)	3109(2)	2883(2)	8864(1)	31(1)
C(4)	2238(1)	4866(2)	8277(1)	23(1)
C(5)	2532(2)	6248(2)	8200(1)	27(1)
C(6)	1479(2)	7171(2)	8068(1)	31(1)
C(7)	117(2)	6731(2)	8004(1)	31(1)
C(8)	-192(2)	5363(2)	8074(1)	29(1)
C(9)	860(2)	4437(2)	8209(1)	25(1)
C(10)	4612(1)	3833(1)	7394(1)	21(1)
C(11)	5518(2)	4493(1)	6980(1)	23(1)
C(12)	5451(2)	4274(2)	6328(1)	25(1)
C(13)	4465(2)	3394(1)	6065(1)	23(1)
C(14)	3567(2)	2728(2)	6480(1)	24(1)
C(15)	3641(2)	2938(1)	7132(1)	24(1)
C(16)	4375(8)	3222(8)	5338(3)	20(2)
C(17)	5752(10)	2826(12)	5032(6)	34(2)
C(18)	3751(10)	4505(7)	5023(4)	28(2)
C(16A)	4327(17)	3127(18)	5386(7)	46(5)
C(17A)	5720(20)	2520(30)	5137(15)	58(5)
C(18A)	4010(30)	4442(18)	5045(9)	56(5)
F(1)	11207(7)	-61(7)	9553(4)	39(1)
C(19)	5827(2)	2970(2)	8324(1)	21(1)
C(20)	6855(2)	3285(2)	8718(1)	20(1)
C(21)	7972(3)	2361(3)	8932(2)	19(1)
C(22)	8319(4)	1170(4)	8603(2)	24(1)
C(23)	9390(8)	322(9)	8809(4)	30(1)
C(24)	10136(8)	720(8)	9338(3)	24(1)
C(25)	9813(5)	1865(5)	9688(2)	26(1)
C(26)	8728(3)	2693(3)	9476(2)	23(1)
F(1A)	11170(30)	190(30)	9649(15)	41(4)

C(19A)	5953(7)	3671(10)	8534(4)	20(2)
C(20A)	6721(7)	2620(7)	8364(3)	16(2)
C(21A)	7884(11)	1980(12)	8722(5)	14(2)
C(22A)	8484(19)	769(16)	8506(9)	26(3)
C(23A)	9620(30)	260(30)	8831(16)	28(4)
C(24A)	10010(40)	800(30)	9410(15)	24(1)
C(25A)	9485(18)	2018(19)	9604(9)	23(3)
C(26A)	8403(11)	2560(10)	9255(6)	15(2)

Table SI 8: Bond lengths [Å] and angles [°] for 1,4-diene **7d**.

C(1)-C(19A)	1.508(7)	C(12)-H(12)	0.9500
C(1)-C(10)	1.518(2)	C(13)-C(14)	1.391(2)
C(1)-C(2)	1.5286(19)	C(13)-C(16A)	1.456(13)
C(1)-C(19)	1.537(2)	C(13)-C(16)	1.539(6)
C(1)-H(1)	1.0000	C(14)-C(15)	1.389(2)
C(1)-H(1A)	1.0000	C(14)-H(14)	0.9500
C(2)-C(3)	1.326(2)	C(15)-H(15)	0.9500
C(2)-C(4)	1.491(2)	C(16)-C(17)	1.524(8)
C(3)-H(3A)	0.9500	C(16)-C(18)	1.544(7)
C(3)-H(3B)	0.9500	C(16)-H(16)	1.0000
C(4)-C(5)	1.394(2)	C(17)-H(17A)	0.9800
C(4)-C(9)	1.3989(19)	C(17)-H(17B)	0.9800
C(5)-C(6)	1.387(2)	C(17)-H(17C)	0.9800
C(5)-H(5)	0.9500	C(18)-H(18A)	0.9800
C(6)-C(7)	1.387(2)	C(18)-H(18B)	0.9800
C(6)-H(6)	0.9500	C(18)-H(18C)	0.9800
C(7)-C(8)	1.382(2)	C(16A)-C(18A)	1.507(16)
C(7)-H(7)	0.9500	C(16A)-C(17A)	1.555(16)
C(8)-C(9)	1.390(2)	C(16A)-H(16A)	1.0000
C(8)-H(8)	0.9500	C(17A)-H(17D)	0.9800
C(9)-H(9)	0.9500	C(17A)-H(17E)	0.9800
C(10)-C(11)	1.392(2)	C(17A)-H(17F)	0.9800
C(10)-C(15)	1.395(2)	C(18A)-H(18D)	0.9800
C(11)-C(12)	1.387(2)	C(18A)-H(18E)	0.9800
C(11)-H(11)	0.9500	C(18A)-H(18F)	0.9800
C(12)-C(13)	1.397(2)	F(1)-C(24)	1.361(5)

C(19)-C(20)	1.328(3)
C(19)-H(19)	0.9500
C(20)-C(21)	1.476(3)
C(20)-H(20)	0.9500
C(21)-C(26)	1.393(4)
C(21)-C(22)	1.398(4)
C(22)-C(23)	1.394(6)
C(22)-H(22)	0.9500
C(23)-C(24)	1.380(5)
C(23)-H(23)	0.9500
C(24)-C(25)	1.378(5)
C(25)-C(26)	1.396(4)
C(25)-H(25)	0.9500
C(26)-H(26)	0.9500
F(1A)-C(24A)	1.360(19)
C(19A)-C(20A)	1.318(12)
C(19A)-H(19A)	0.9500
C(20A)-C(21A)	1.488(12)
C(20A)-H(20A)	0.9500
C(21A)-C(26A)	1.353(12)
C(21A)-C(22A)	1.395(12)
C(22A)-C(23A)	1.378(19)
C(22A)-H(22A)	0.9500
C(23A)-C(24A)	1.38(2)
C(23A)-H(23A)	0.9500
C(24A)-C(25A)	1.359(19)
C(25A)-C(26A)	1.381(14)
C(25A)-H(25A)	0.9500
C(26A)-H(26A)	0.9500
C(19A)-C(1)-C(10)	128.5(3)
C(19A)-C(1)-C(2)	112.4(3)
C(10)-C(1)-C(2)	111.70(12)
C(10)-C(1)-C(19)	105.34(14)
C(2)-C(1)-C(19)	113.08(13)
C(10)-C(1)-H(1)	108.9
C(2)-C(1)-H(1)	108.9
C(19)-C(1)-H(1)	108.9
C(19A)-C(1)-H(1A)	99.0

C(10)-C(1)-H(1A)	99.0
C(2)-C(1)-H(1A)	99.0
C(3)-C(2)-C(4)	120.76(14)
C(3)-C(2)-C(1)	122.23(14)
C(4)-C(2)-C(1)	117.01(12)
C(2)-C(3)-H(3A)	120.0
C(2)-C(3)-H(3B)	120.0
H(3A)-C(3)-H(3B)	120.0
C(5)-C(4)-C(9)	118.22(14)
C(5)-C(4)-C(2)	121.44(13)
C(9)-C(4)-C(2)	120.32(13)
C(6)-C(5)-C(4)	120.58(14)
C(6)-C(5)-H(5)	119.7
C(4)-C(5)-H(5)	119.7
C(7)-C(6)-C(5)	120.52(15)
C(7)-C(6)-H(6)	119.7
C(5)-C(6)-H(6)	119.7
C(8)-C(7)-C(6)	119.68(15)
C(8)-C(7)-H(7)	120.2
C(6)-C(7)-H(7)	120.2
C(7)-C(8)-C(9)	119.94(14)
C(7)-C(8)-H(8)	120.0
C(9)-C(8)-H(8)	120.0
C(8)-C(9)-C(4)	121.05(14)
C(8)-C(9)-H(9)	119.5
C(4)-C(9)-H(9)	119.5
C(11)-C(10)-C(15)	117.68(13)
C(11)-C(10)-C(1)	120.57(13)
C(15)-C(10)-C(1)	121.65(13)
C(12)-C(11)-C(10)	121.13(13)
C(12)-C(11)-H(11)	119.4
C(10)-C(11)-H(11)	119.4
C(11)-C(12)-C(13)	121.26(14)
C(11)-C(12)-H(12)	119.4
C(13)-C(12)-H(12)	119.4
C(14)-C(13)-C(12)	117.55(14)
C(14)-C(13)-C(16A)	118.2(7)
C(12)-C(13)-C(16A)	124.2(7)
C(14)-C(13)-C(16)	122.4(3)

C(12)-C(13)-C(16)	120.0(3)	H(18D)-C(18A)-H(18E)	109.5
C(15)-C(14)-C(13)	121.23(14)	C(16A)-C(18A)-H(18F)	109.5
C(15)-C(14)-H(14)	119.4	H(18D)-C(18A)-H(18F)	109.5
C(13)-C(14)-H(14)	119.4	H(18E)-C(18A)-H(18F)	109.5
C(14)-C(15)-C(10)	121.15(14)	C(20)-C(19)-C(1)	122.2(2)
C(14)-C(15)-H(15)	119.4	C(20)-C(19)-H(19)	118.9
C(10)-C(15)-H(15)	119.4	C(1)-C(19)-H(19)	118.9
C(17)-C(16)-C(13)	113.4(6)	C(19)-C(20)-C(21)	126.2(2)
C(17)-C(16)-C(18)	111.4(6)	C(19)-C(20)-H(20)	116.9
C(13)-C(16)-C(18)	111.0(5)	C(21)-C(20)-H(20)	116.9
C(17)-C(16)-H(16)	106.9	C(26)-C(21)-C(22)	118.5(3)
C(13)-C(16)-H(16)	106.9	C(26)-C(21)-C(20)	119.2(3)
C(18)-C(16)-H(16)	106.9	C(22)-C(21)-C(20)	122.3(3)
C(16)-C(17)-H(17A)	109.5	C(23)-C(22)-C(21)	121.4(4)
C(16)-C(17)-H(17B)	109.5	C(23)-C(22)-H(22)	119.3
H(17A)-C(17)-H(17B)	109.5	C(21)-C(22)-H(22)	119.3
C(16)-C(17)-H(17C)	109.5	C(24)-C(23)-C(22)	117.9(5)
H(17A)-C(17)-H(17C)	109.5	C(24)-C(23)-H(23)	121.1
H(17B)-C(17)-H(17C)	109.5	C(22)-C(23)-H(23)	121.1
C(16)-C(18)-H(18A)	109.5	F(1)-C(24)-C(25)	116.9(5)
C(16)-C(18)-H(18B)	109.5	F(1)-C(24)-C(23)	120.2(5)
H(18A)-C(18)-H(18B)	109.5	C(25)-C(24)-C(23)	122.9(5)
C(16)-C(18)-H(18C)	109.5	C(24)-C(25)-C(26)	118.2(4)
H(18A)-C(18)-H(18C)	109.5	C(24)-C(25)-H(25)	120.9
H(18B)-C(18)-H(18C)	109.5	C(26)-C(25)-H(25)	120.9
C(13)-C(16A)-C(18A)	109.3(12)	C(21)-C(26)-C(25)	121.2(3)
C(13)-C(16A)-C(17A)	108.7(14)	C(21)-C(26)-H(26)	119.4
C(18A)-C(16A)-C(17A)	109.8(13)	C(25)-C(26)-H(26)	119.4
C(13)-C(16A)-H(16A)	109.7	C(20A)-C(19A)-C(1)	116.9(8)
C(18A)-C(16A)-H(16A)	109.7	C(20A)-C(19A)-H(19A)	121.6
C(17A)-C(16A)-H(16A)	109.7	C(1)-C(19A)-H(19A)	121.6
C(16A)-C(17A)-H(17D)	109.5	C(19A)-C(20A)-C(21A)	128.0(7)
C(16A)-C(17A)-H(17E)	109.5	C(19A)-C(20A)-H(20A)	116.0
H(17D)-C(17A)-H(17E)	109.5	C(21A)-C(20A)-H(20A)	116.0
C(16A)-C(17A)-H(17F)	109.5	C(26A)-C(21A)-C(22A)	118.3(11)
H(17D)-C(17A)-H(17F)	109.5	C(26A)-C(21A)-C(20A)	121.3(9)
H(17E)-C(17A)-H(17F)	109.5	C(22A)-C(21A)-C(20A)	120.4(11)
C(16A)-C(18A)-H(18D)	109.5	C(23A)-C(22A)-C(21A)	118.1(16)
C(16A)-C(18A)-H(18E)	109.5	C(23A)-C(22A)-H(22A)	120.9

C(21A)-C(22A)-H(22A)	120.9	C(24A)-C(25A)-C(26A)	117.4(15)
C(22A)-C(23A)-C(24A)	121(2)	C(24A)-C(25A)-H(25A)	121.3
C(22A)-C(23A)-H(23A)	119.4	C(26A)-C(25A)-H(25A)	121.3
C(24A)-C(23A)-H(23A)	119.4	C(21A)-C(26A)-C(25A)	123.8(11)
C(25A)-C(24A)-F(1A)	125(2)	C(21A)-C(26A)-H(26A)	118.1
C(25A)-C(24A)-C(23A)	120(2)	C(25A)-C(26A)-H(26A)	118.1
F(1A)-C(24A)-C(23A)	112.6(19)		

Table SI 9: Anisotropic displacement parameters ($Å^2 x 10^3$) for 1,4-diene **7d**. The anisotropic displacement factor exponent takes the form: $-2p^2[h^2 a^{*2}U^{11} + ... + 2 h k a^* b^* U^{12}]$

	U^{11}	U^{22}	U^{33}	U^{23}	U^{13}	U^{12}
C(1)	17(1)	34(1)	25(1)	7(1)	-2(1)	2(1)
C(2)	19(1)	32(1)	22(1)	3(1)	-1(1)	0(1)
C(3)	25(1)	36(1)	30(1)	7(1)	3(1)	3(1)
C(4)	19(1)	31(1)	18(1)	0(1)	1(1)	0(1)
C(5)	20(1)	32(1)	30(1)	1(1)	-1(1)	-2(1)
C(6)	27(1)	28(1)	38(1)	1(1)	1(1)	1(1)
C(7)	22(1)	36(1)	34(1)	-1(1)	0(1)	9(1)
C(8)	17(1)	40(1)	29(1)	-5(1)	0(1)	-1(1)
C(9)	21(1)	29(1)	24(1)	-3(1)	1(1)	-2(1)
C(10)	17(1)	18(1)	27(1)	2(1)	2(1)	6(1)
C(11)	22(1)	18(1)	30(1)	0(1)	1(1)	-3(1)
C(12)	24(1)	22(1)	29(1)	0(1)	6(1)	-4(1)
C(13)	20(1)	20(1)	30(1)	-3(1)	3(1)	3(1)
C(14)	17(1)	21(1)	36(1)	-3(1)	1(1)	-1(1)
C(15)	17(1)	20(1)	34(1)	4(1)	5(1)	1(1)
C(16)	20(3)	25(3)	13(2)	-7(2)	-2(2)	-6(2)
C(17)	23(2)	49(3)	31(3)	-16(2)	6(2)	4(2)
C(18)	28(2)	31(3)	24(2)	8(2)	0(2)	-3(2)
C(16A)	27(6)	43(6)	67(9)	-3(5)	21(5)	2(4)
C(17A)	40(5)	84(11)	50(9)	-32(7)	13(5)	-15(6)
C(18A)	59(9)	73(7)	36(5)	-17(5)	-7(5)	-4(5)
F(1)	24(1)	39(2)	53(2)	12(2)	-7(1)	11(1)
C(19)	20(1)	19(1)	23(1)	1(1)	-1(1)	1(1)
C(20)	20(1)	20(1)	21(1)	0(1)	1(1)	0(1)

C(21)	16(1)	21(2)	20(2)	0(1)	-1(1)	-2(1)
C(22)	23(2)	25(2)	26(2)	1(2)	-4(1)	2(1)
C(23)	26(3)	29(2)	34(2)	-1(1)	-3(2)	2(2)
C(24)	15(2)	28(1)	30(2)	9(1)	1(2)	5(1)
C(25)	16(2)	33(2)	28(2)	5(1)	-4(1)	3(1)
C(26)	20(1)	27(1)	22(2)	-2(1)	-4(1)	-1(1)
F(1A)	35(5)	41(9)	48(8)	9(6)	-18(5)	12(4)
C(19A)	15(4)	28(4)	18(3)	-3(3)	-3(3)	-5(3)
C(20A)	11(4)	22(4)	15(3)	0(3)	-4(2)	-2(3)
C(21A)	16(4)	13(5)	13(5)	-7(3)	0(3)	-2(3)
C(22A)	25(5)	18(6)	35(6)	-4(4)	-1(4)	2(4)
C(23A)	14(7)	32(7)	39(6)	12(4)	0(4)	8(4)
C(24A)	15(2)	28(1)	30(2)	9(1)	1(2)	5(1)
C(25A)	13(7)	26(5)	28(6)	-1(4)	-8(4)	6(4)
C(26A)	12(5)	19(4)	13(5)	-12(4)	-6(3)	6(3)

Table SI 10: Hydrogen coordinates (x 10^4) and isotropic displacement parameters (Å^{2}x 10 ^{3}) for 1,4-diene **7d**.

	x	y	z	U(eq)
H(1)	5113	4961	8195	31
H(1A)	4822	5043	8121	31
H(3A)	2225	2810	9062	37
H(3B)	3823	2251	8966	37
H(5)	3463	6561	8237	32
H(6)	1692	8112	8020	37
H(7)	-600	7367	7912	37
H(8)	-1123	5056	8030	35
H(9)	640	3497	8256	30
H(11)	6193	5103	7146	28
H(12)	6088	4730	6057	30
H(14)	2891	2119	6314	29
H(15)	3019	2464	7405	29
H(16)	3713	2455	5256	23
H(17A)	5608	2645	4579	52
H(17B)	6117	2005	5239	52

H(17C)	6418	3575	5082	52
H(18A)	3660	4352	4564	41
H(18B)	4362	5287	5099	41
H(18C)	2833	4689	5205	41
H(16A)	3557	2460	5313	55
H(17D)	5651	2370	4677	87
H(17E)	5904	1653	5351	87
H(17F)	6476	3162	5225	87
H(18D)	3952	4270	4586	84
H(18E)	4756	5102	5128	84
H(18F)	3128	4810	5198	84
H(19)	5758	2061	8169	25
H(20)	6874	4191	8878	24
H(22)	7814	934	8230	29
H(23)	9600	-503	8593	36
H(25)	10316	2086	10063	31
H(26)	8501	3497	9706	28
H(19A)	6150	4181	8908	24
H(20A)	6507	2223	7964	19
H(22A)	8122	305	8146	31
H(23A)	10133	-468	8653	34
H(25A)	9848	2477	9966	27
H(26A)	7998	3389	9398	18

10.3 1,4-diene 7e

The structure of **7e** was solved in the orthorhombic space group $P2_12_12_1$. The asymmetric unit contains one full molecule, which was found to be disordered at two positions. The disorder was refined using same distance restraints on 1,2 and 1,3 distances, similarity restraints on anisotropic displacement parameters, by setting atoms with very similar coordinates to have the same anisotropic displacement parameters and by using advanced rigid bond restraints. The disorder ratios were allowed to refine and converged to 0.542(7) and 0.63(5). The determination of the absolute structure could not reliably be done by determination of the Parsons parameter, as the crystal quality was not good enough. Bayesian statistics on Bijvoet pairs however indicate the correct assignment.[19] Crystals suitable for SCXRD analysis were obtained by crystallization from a saturated DCM solution at room temperature.

Table SI 11: Crystal data and structure refinement for 1,4-diene **7e.**

CCDC No	2150153	
Empirical formula	$C_{26} H_{25} Cl$	
Formula weight	372.91	
Temperature	100(2) K	
Wavelength	0.71073 Å	
Crystal system	Orthorhombic	
Space group	$P2_12_12_1$	
Unit cell dimensions	a = 9.8328(15) Å	α= 90°.
	b = 9.9308(15) Å	β= 90°.
	c = 20.994(3) Å	γ = 90°.
Volume	2050.0(5) $Å^3$	
Z	4	
Density (calculated)	1.208 Mg/m^3	
Absorption coefficient	0.194 mm^{-1}	
F(000)	792	
Crystal size	0.779 x 0.188 x 0.084 mm^3	
Theta range for data collection	1.940 to 27.475°.	
Index ranges	$-12 \le h \le 12$, $-12 \le k \le 12$, $-27 \le l \le 27$	
Reflections collected	29847	
Independent reflections	4700 [R(int) = 0.0578]	
Completeness to theta = 25.242°	100.0 %	
Absorption correction	Semi-empirical from equivalents	
Refinement method	Full-matrix least-squares on F^2	
Data / restraints / parameters	4700 / 734 / 346	
Goodness-of-fit on F^2	1.092	
Final R indices [I>2σ(I)]	R1 = 0.0394, wR2 = 0.0789	
R indices (all data)	R1 = 0.0550, wR2 = 0.0850	
Absolute structure parameter	0.20(2)	
Largest diff. peak and hole	0.169 and -0.216 e.$Å^{-3}$	

Table SI 12: Atomic coordinates (x 10^4) and equivalent isotropic displacement parameters ($Å^2$x 10^3) for 1,4-diene **7e**. U(eq) is defined as one third of the trace of the orthogonalized U^{ij} tensor.

	x	y	z	U(eq)
C(1)	4664(2)	5705(3)	8155(1)	32(1)
C(2)	3295(2)	5888(3)	8492(1)	30(1)
C(3)	3089(3)	6858(3)	8916(1)	38(1)
C(4)	2192(2)	4942(3)	8312(1)	27(1)
C(5)	861(2)	5403(2)	8231(1)	28(1)
C(6)	-179(2)	4524(3)	8079(1)	32(1)
C(7)	85(2)	3171(3)	8003(1)	36(1)
C(8)	1396(3)	2695(3)	8081(1)	37(1)
C(9)	2440(2)	3572(3)	8231(1)	34(1)
C(10)	4545(2)	6002(2)	7446(1)	24(1)
C(11)	3640(2)	6940(2)	7205(1)	27(1)
C(12)	3567(2)	7205(2)	6557(1)	28(1)
C(13)	4411(2)	6551(2)	6127(1)	26(1)
C(14)	5326(2)	5613(2)	6371(1)	29(1)
C(15)	5395(2)	5347(2)	7016(1)	26(1)
C(16)	4257(18)	6802(19)	5418(5)	32(1)
C(17)	5603(15)	7290(20)	5124(8)	46(3)
C(18)	3740(17)	5543(13)	5069(5)	39(2)
C(16A)	4430(30)	6870(30)	5422(8)	32(1)
C(17A)	5760(30)	7590(30)	5234(12)	42(4)
C(18A)	4200(40)	5610(20)	5024(11)	53(4)
Cl(1)	11354(9)	9880(11)	9602(5)	35(1)
C(19)	5846(5)	6196(6)	8533(2)	25(1)
C(20)	6585(5)	7214(4)	8332(2)	22(1)
C(21)	7743(5)	7846(7)	8677(3)	17(1)
C(22)	8244(7)	9067(7)	8447(3)	23(1)
C(23)	9333(15)	9698(15)	8738(8)	24(2)
C(24)	9940(40)	9100(30)	9250(20)	24(1)
C(25)	9452(10)	7898(12)	9500(6)	25(2)
C(26)	8356(6)	7271(6)	9203(3)	22(1)
Cl(1A)	11377(10)	10071(14)	9530(6)	41(2)
C(19A)	5715(6)	6864(7)	8355(3)	22(1)
C(20A)	6771(5)	6551(5)	8715(2)	24(2)
C(21A)	7886(7)	7464(7)	8919(4)	18(2)

C(22A)	8122(9)	8660(9)	8609(4)	25(2)
C(23A)	9200(20)	9490(20)	8796(11)	34(3)
C(24A)	10000(50)	9090(40)	9300(20)	24(1)
C(25A)	9783(13)	7896(15)	9612(7)	28(2)
C(26A)	8726(8)	7089(9)	9416(4)	26(2)

Table SI 13: Bond lengths [Å] and angles [°] for 1,4-diene **7e**.

C(1)-C(19)	1.489(5)	C(13)-C(16)	1.516(11)
C(1)-C(10)	1.521(3)	C(14)-C(15)	1.381(3)
C(1)-C(2)	1.532(3)	C(14)-H(14)	0.9500
C(1)-C(19A)	1.603(6)	C(15)-H(15)	0.9500
C(1)-H(1)	1.0000	C(16)-C(18)	1.535(10)
C(1)-H(1A)	1.0000	C(16)-C(17)	1.539(11)
C(2)-C(3)	1.327(3)	C(16)-H(16)	1.0000
C(2)-C(4)	1.484(3)	C(17)-H(17A)	0.9800
C(3)-H(3A)	0.9500	C(17)-H(17B)	0.9800
C(3)-H(3B)	0.9500	C(17)-H(17C)	0.9800
C(4)-C(9)	1.392(4)	C(18)-H(18A)	0.9800
C(4)-C(5)	1.396(3)	C(18)-H(18B)	0.9800
C(5)-C(6)	1.382(3)	C(18)-H(18C)	0.9800
C(5)-H(5)	0.9500	C(16A)-C(18A)	1.525(18)
C(6)-C(7)	1.378(4)	C(16A)-C(17A)	1.543(17)
C(6)-H(6)	0.9500	C(16A)-H(16A)	1.0000
C(7)-C(8)	1.383(4)	C(17A)-H(17D)	0.9800
C(7)-H(7)	0.9500	C(17A)-H(17E)	0.9800
C(8)-C(9)	1.383(4)	C(17A)-H(17F)	0.9800
C(8)-H(8)	0.9500	C(18A)-H(18D)	0.9800
C(9)-H(9)	0.9500	C(18A)-H(18E)	0.9800
C(10)-C(11)	1.384(3)	C(18A)-H(18F)	0.9800
C(10)-C(15)	1.392(3)	Cl(1)-C(24)	1.747(11)
C(11)-C(12)	1.387(3)	C(19)-C(20)	1.314(7)
C(11)-H(11)	0.9500	C(19)-H(19)	0.9500
C(12)-C(13)	1.388(3)	C(20)-C(21)	1.489(7)
C(12)-H(12)	0.9500	C(20)-H(20)	0.9500
C(13)-C(14)	1.393(3)	C(21)-C(26)	1.382(7)
C(13)-C(16A)	1.514(17)	C(21)-C(22)	1.395(7)

C(22)-C(23)	1.383(10)
C(22)-H(22)	0.9500
C(23)-C(24)	1.374(12)
C(23)-H(23)	0.9500
C(24)-C(25)	1.387(12)
C(25)-C(26)	1.392(9)
C(25)-H(25)	0.9500
C(26)-H(26)	0.9500
Cl(1A)-C(24A)	1.737(13)
C(19A)-C(20A)	1.321(8)
C(19A)-H(19A)	0.9500
C(20A)-C(21A)	1.486(8)
C(20A)-H(20A)	0.9500
C(21A)-C(22A)	1.374(8)
C(21A)-C(26A)	1.381(8)
C(22A)-C(23A)	1.403(13)
C(22A)-H(22A)	0.9500
C(23A)-C(24A)	1.367(14)
C(23A)-H(23A)	0.9500
C(24A)-C(25A)	1.378(14)
C(25A)-C(26A)	1.376(10)
C(25A)-H(25A)	0.9500
C(26A)-H(26A)	0.9500
C(19)-C(1)-C(10)	121.2(3)
C(19)-C(1)-C(2)	113.6(2)
C(10)-C(1)-C(2)	111.20(19)
C(10)-C(1)-C(19A)	99.6(3)
C(2)-C(1)-C(19A)	111.1(3)
C(19)-C(1)-H(1)	102.6
C(10)-C(1)-H(1)	102.6
C(2)-C(1)-H(1)	102.6
C(10)-C(1)-H(1A)	111.5
C(2)-C(1)-H(1A)	111.5
C(19A)-C(1)-H(1A)	111.5
C(3)-C(2)-C(4)	121.3(2)
C(3)-C(2)-C(1)	122.0(2)
C(4)-C(2)-C(1)	116.7(2)
C(2)-C(3)-H(3A)	120.0
C(2)-C(3)-H(3B)	120.0
H(3A)-C(3)-H(3B)	120.0
C(9)-C(4)-C(5)	118.0(2)
C(9)-C(4)-C(2)	121.4(2)
C(5)-C(4)-C(2)	120.6(2)
C(6)-C(5)-C(4)	121.0(2)
C(6)-C(5)-H(5)	119.5
C(4)-C(5)-H(5)	119.5
C(7)-C(6)-C(5)	120.2(2)
C(7)-C(6)-H(6)	119.9
C(5)-C(6)-H(6)	119.9
C(6)-C(7)-C(8)	119.7(2)
C(6)-C(7)-H(7)	120.1
C(8)-C(7)-H(7)	120.1
C(7)-C(8)-C(9)	120.2(2)
C(7)-C(8)-H(8)	119.9
C(9)-C(8)-H(8)	119.9
C(8)-C(9)-C(4)	120.9(2)
C(8)-C(9)-H(9)	119.6
C(4)-C(9)-H(9)	119.6
C(11)-C(10)-C(15)	117.6(2)
C(11)-C(10)-C(1)	122.6(2)
C(15)-C(10)-C(1)	119.8(2)
C(10)-C(11)-C(12)	121.4(2)
C(10)-C(11)-H(11)	119.3
C(12)-C(11)-H(11)	119.3
C(11)-C(12)-C(13)	121.2(2)
C(11)-C(12)-H(12)	119.4
C(13)-C(12)-H(12)	119.4
C(12)-C(13)-C(14)	117.4(2)
C(12)-C(13)-C(16A)	122.9(14)
C(14)-C(13)-C(16A)	119.7(14)
C(12)-C(13)-C(16)	120.1(8)
C(14)-C(13)-C(16)	122.4(8)
C(15)-C(14)-C(13)	121.4(2)
C(15)-C(14)-H(14)	119.3
C(13)-C(14)-H(14)	119.3
C(14)-C(15)-C(10)	121.2(2)
C(14)-C(15)-H(15)	119.4

C(10)-C(15)-H(15)	119.4	C(1)-C(19)-H(19)	119.6
C(13)-C(16)-C(18)	111.5(10)	C(19)-C(20)-C(21)	126.2(5)
C(13)-C(16)-C(17)	111.1(10)	C(19)-C(20)-H(20)	116.9
C(18)-C(16)-C(17)	110.5(10)	C(21)-C(20)-H(20)	116.9
C(13)-C(16)-H(16)	107.8	C(26)-C(21)-C(22)	118.8(5)
C(18)-C(16)-H(16)	107.8	C(26)-C(21)-C(20)	123.2(6)
C(17)-C(16)-H(16)	107.8	C(22)-C(21)-C(20)	117.9(6)
C(16)-C(17)-H(17A)	109.5	C(23)-C(22)-C(21)	121.0(7)
C(16)-C(17)-H(17B)	109.5	C(23)-C(22)-H(22)	119.5
H(17A)-C(17)-H(17B)	109.5	C(21)-C(22)-H(22)	119.5
C(16)-C(17)-H(17C)	109.5	C(24)-C(23)-C(22)	119.4(10)
H(17A)-C(17)-H(17C)	109.5	C(24)-C(23)-H(23)	120.3
H(17B)-C(17)-H(17C)	109.5	C(22)-C(23)-H(23)	120.3
C(16)-C(18)-H(18A)	109.5	C(23)-C(24)-C(25)	120.8(10)
C(16)-C(18)-H(18B)	109.5	C(23)-C(24)-Cl(1)	119.0(10)
H(18A)-C(18)-H(18B)	109.5	C(25)-C(24)-Cl(1)	120.2(10)
C(16)-C(18)-H(18C)	109.5	C(24)-C(25)-C(26)	119.3(8)
H(18A)-C(18)-H(18C)	109.5	C(24)-C(25)-H(25)	120.3
H(18B)-C(18)-H(18C)	109.5	C(26)-C(25)-H(25)	120.3
C(13)-C(16A)-C(18A)	111.1(18)	C(21)-C(26)-C(25)	120.6(6)
C(13)-C(16A)-C(17A)	110.8(17)	C(21)-C(26)-H(26)	119.7
C(18A)-C(16A)-C(17A)	111.1(17)	C(25)-C(26)-H(26)	119.7
C(13)-C(16A)-H(16A)	107.9	C(20A)-C(19A)-C(1)	119.2(6)
C(18A)-C(16A)-H(16A)	107.9	C(20A)-C(19A)-H(19A)	120.4
C(17A)-C(16A)-H(16A)	107.9	C(1)-C(19A)-H(19A)	120.4
C(16A)-C(17A)-H(17D)	109.5	C(19A)-C(20A)-C(21A)	127.0(6)
C(16A)-C(17A)-H(17E)	109.5	C(19A)-C(20A)-H(20A)	116.5
H(17D)-C(17A)-H(17E)	109.5	C(21A)-C(20A)-H(20A)	116.5
C(16A)-C(17A)-H(17F)	109.5	C(22A)-C(21A)-C(26A)	119.3(7)
H(17D)-C(17A)-H(17F)	109.5	C(22A)-C(21A)-C(20A)	121.0(7)
H(17E)-C(17A)-H(17F)	109.5	C(26A)-C(21A)-C(20A)	119.6(7)
C(16A)-C(18A)-H(18D)	109.5	C(21A)-C(22A)-C(23A)	120.3(9)
C(16A)-C(18A)-H(18E)	109.5	C(21A)-C(22A)-H(22A)	119.8
H(18D)-C(18A)-H(18E)	109.5	C(23A)-C(22A)-H(22A)	119.8
C(16A)-C(18A)-H(18F)	109.5	C(24A)-C(23A)-C(22A)	118.6(12)
H(18D)-C(18A)-H(18F)	109.5	C(24A)-C(23A)-H(23A)	120.7
H(18E)-C(18A)-H(18F)	109.5	C(22A)-C(23A)-H(23A)	120.7
C(20)-C(19)-C(1)	120.8(5)	C(23A)-C(24A)-C(25A)	122.0(12)
C(20)-C(19)-H(19)	119.6	C(23A)-C(24A)-Cl(1A)	120.1(12)

C(25A)-C(24A)-Cl(1A)	117.8(12)	C(25A)-C(26A)-C(21A)	121.4(8)
C(26A)-C(25A)-C(24A)	118.4(9)	C(25A)-C(26A)-H(26A)	119.3
C(26A)-C(25A)-H(25A)	120.8	C(21A)-C(26A)-H(26A)	119.3
C(24A)-C(25A)-H(25A)	120.8		

Table SI 14: Anisotropic displacement parameters (Å^{2}x 10^3) for 1,4-diene **7e**. The anisotropic displacement factor exponent takes the form: -2p^2[h^2 a*^{2}U^{11} + ... + 2 h k a* b* U^{12}]

	U^{11}	U^{22}	U^{33}	U^{23}	U^{13}	U^{12}
C(1)	18(1)	48(2)	30(1)	-9(1)	-2(1)	-4(1)
C(2)	19(1)	42(1)	28(1)	-2(1)	-1(1)	-3(1)
C(3)	27(1)	49(2)	37(1)	-9(1)	2(1)	-7(1)
C(4)	20(1)	36(1)	24(1)	-1(1)	0(1)	-2(1)
C(5)	25(1)	33(1)	28(1)	1(1)	1(1)	-1(1)
C(6)	19(1)	43(2)	36(1)	5(1)	0(1)	-2(1)
C(7)	26(1)	40(2)	42(1)	1(1)	1(1)	-10(1)
C(8)	33(1)	33(1)	46(2)	2(1)	1(1)	-1(1)
C(9)	24(1)	39(2)	38(1)	1(1)	-2(1)	3(1)
C(10)	19(1)	20(1)	32(1)	-3(1)	1(1)	-7(1)
C(11)	18(1)	24(1)	38(1)	-6(1)	6(1)	-2(1)
C(12)	17(1)	22(1)	44(1)	4(1)	4(1)	1(1)
C(13)	23(1)	22(1)	34(1)	3(1)	3(1)	-2(1)
C(14)	28(1)	25(1)	35(1)	0(1)	5(1)	7(1)
C(15)	24(1)	21(1)	35(1)	1(1)	0(1)	3(1)
C(16)	23(4)	37(2)	35(1)	9(1)	4(2)	3(2)
C(17)	22(3)	65(7)	50(5)	32(5)	-2(3)	6(4)
C(18)	46(5)	46(4)	25(3)	4(2)	3(3)	4(4)
C(16A)	23(4)	37(2)	35(1)	9(1)	4(2)	3(2)
C(17A)	30(6)	63(8)	34(7)	12(5)	12(5)	-4(5)
C(18A)	62(12)	60(6)	38(6)	2(4)	2(7)	-15(8)
Cl(1)	28(2)	35(3)	42(1)	-11(2)	-11(1)	-2(1)
C(19)	20(2)	26(3)	27(2)	4(2)	-4(2)	5(2)
C(20)	18(2)	24(2)	23(2)	0(2)	-1(2)	4(2)
C(21)	15(2)	14(3)	23(3)	4(2)	-1(2)	4(2)
C(22)	25(3)	16(3)	28(3)	7(2)	-6(2)	-1(2)
C(23)	19(3)	23(5)	30(4)	-1(3)	-3(3)	-5(3)

C(24)	17(3)	24(1)	29(4)	-7(2)	0(1)	-2(2)
C(25)	21(5)	30(3)	25(4)	2(3)	-6(3)	4(3)
C(26)	19(4)	21(3)	26(4)	10(3)	-4(2)	-1(3)
Cl(1A)	16(2)	36(3)	70(4)	-15(3)	-9(2)	-5(1)
C(19A)	14(3)	23(3)	30(3)	-3(2)	-3(2)	1(2)
C(20A)	16(2)	23(3)	32(3)	1(2)	-2(2)	-3(2)
C(21A)	18(3)	13(3)	24(4)	6(3)	-5(3)	3(2)
C(22A)	25(3)	25(5)	25(4)	11(3)	-7(3)	-1(3)
C(23A)	36(6)	22(5)	43(6)	8(4)	-2(4)	-8(3)
C(24A)	17(3)	24(1)	29(4)	-7(2)	0(1)	-2(2)
C(25A)	20(5)	35(4)	28(5)	1(3)	-7(3)	-4(4)
C(26A)	20(3)	29(4)	28(4)	5(3)	-9(3)	0(3)

Table SI 15: Hydrogen coordinates (x 10^4) and isotropic displacement parameters ($Å^2$x 10^3) for 1,4-diene **7e**.

	x	y	z	U(eq)
H(1)	4791	4706	8163	38
H(1A)	5050	4788	8233	38
H(3A)	2222	6958	9110	45
H(3B)	3808	7454	9025	45
H(5)	669	6334	8281	34
H(6)	-1080	4854	8027	39
H(7)	-630	2568	7897	43
H(8)	1580	1761	8032	45
H(9)	3339	3236	8279	40
H(11)	3057	7411	7488	32
H(12)	2928	7846	6405	33
H(14)	5915	5147	6088	35
H(15)	6033	4706	7169	32
H(16)	3567	7531	5360	38
H(17A)	6296	6588	5171	69
H(17B)	5904	8109	5343	69
H(17C)	5466	7485	4671	69
H(18A)	3663	5735	4613	59
H(18B)	2845	5291	5237	59
H(18C)	4380	4800	5135	59

H(16A)	3659	7507	5334	38
H(17D)	6535	6997	5332	63
H(17E)	5849	8427	5474	63
H(17F)	5752	7783	4777	63
H(18D)	4139	5856	4573	80
H(18E)	3355	5170	5157	80
H(18F)	4965	4989	5086	80
H(19)	6068	5769	8924	30
H(20)	6359	7581	7928	26
H(22)	7832	9470	8085	28
H(23)	9655	10538	8583	29
H(25)	9859	7507	9866	30
H(26)	8026	6438	9364	27
H(19A)	5580	7765	8217	27
H(20A)	6824	5645	8858	28
H(22A)	7549	8925	8267	30
H(23A)	9380	10313	8579	41
H(25A)	10349	7635	9957	34
H(26A)	8570	6257	9626	31

10.4 1,4-diene 7f

The structure of **7f** was solved in the orthorhombic space group $P2_12_12_1$ as racemic twin. The twin ratio was refined and converged to 0.24(1). The asymmetric unit contains one full molecule, which was found to be disordered at two positions. The disorder was refined using same distance restraints on 1,2 and 1,3 distances, similarity restraints on anisotropic displacement parameters, by setting two atoms with very similar coordinates to have the same anisotropic displacement parameters and by using advanced rigid bond restraints. The disorder ratios were allowed to refine and converged to 0.694(8) and 0.54(7). Crystals suitable for SCXRD analysis were obtained by crystallization from a saturated *n*-hexane solution at room temperature.

Table SI 16: Crystal data and structure refinement for 1,4-diene **7f**.

CCDC No	2150156
Empirical formula	$C_{26}H_{25}Br$
Formula weight	417.37
Temperature	100(2) K
Wavelength	0.71073 Å

Crystal system	Orthorhombic	
Space group	$P2_12_12_1$	
Unit cell dimensions	a = 9.9383(7) Å	α= 90°.
	b = 10.0009(8) Å	β= 90°.
	c = 21.0795(17) Å	γ = 90°.
Volume	2095.1(3) Å^3	
Z	4	
Density (calculated)	1.323 Mg/m^3	
Absorption coefficient	1.969 mm^{-1}	
F(000)	864	
Crystal size	0.569 x 0.406 x 0.239 mm^3	
Theta range for data collection	1.932 to 29.571°.	
Index ranges	-13<=h<=13, -13<=k<=13, -29<=l<=29	
Reflections collected	204139	
Independent reflections	5847 [R(int) = 0.0592]	
Completeness to theta = 25.242°	99.3 %	
Absorption correction	Semi-empirical from equivalents	
Refinement method	Full-matrix least-squares on F^2	
Data / restraints / parameters	5847 / 517 / 353	
Goodness-of-fit on F^2	1.143	
Final R indices [I>2σ(I)]	R1 = 0.0348, wR2 = 0.1016	
R indices (all data)	R1 = 0.0369, wR2 = 0.1030	
Absolute structure parameter	0.243(11)	
Largest diff. peak and hole	1.035 and -0.473 e.Å^{-3}	

Table SI 17: Atomic coordinates (x 10^4) and equivalent isotropic displacement parameters (Å^{2}x 10^3) for 1,4-diene **7f**. U(eq) is defined as one third of the trace of the orthogonalized U^{ij} tensor.

	x	y	z	U(eq)
C(1)	5358(3)	5570(4)	1833(2)	29(1)
C(2)	6690(3)	5759(4)	1488(2)	28(1)
C(3)	6878(4)	6721(4)	1057(2)	36(1)
C(4)	7805(3)	4846(3)	1673(1)	25(1)
C(5)	9103(3)	5340(3)	1757(1)	28(1)
C(6)	10156(3)	4486(4)	1918(2)	32(1)
C(7)	9921(4)	3135(4)	1997(2)	36(1)
C(8)	8628(4)	2624(4)	1912(2)	38(1)

C(9)	7583(3)	3475(4)	1753(2)	31(1)
C(10)	5477(3)	5913(3)	2535(1)	21(1)
C(11)	6362(3)	6875(3)	2764(1)	24(1)
C(12)	6434(3)	7164(3)	3408(2)	25(1)
C(13)	5624(3)	6507(3)	3846(2)	24(1)
C(14)	4733(3)	5548(3)	3614(2)	27(1)
C(15)	4660(3)	5261(3)	2973(1)	24(1)
C(16)	5680(20)	6870(20)	4542(5)	31(2)
C(17)	5990(40)	5642(19)	4944(10)	57(5)
C(18)	4360(20)	7530(30)	4753(11)	37(3)
C(16A)	5770(30)	6740(30)	4555(6)	31(2)
C(17A)	6370(20)	5520(30)	4895(11)	42(4)
C(18A)	4440(20)	7170(40)	4866(15)	44(5)
Br(1)	-1380(3)	9870(3)	407(1)	29(1)
C(19)	4189(4)	6126(6)	1476(2)	23(1)
C(20)	3458(4)	7137(4)	1685(2)	21(1)
C(21)	2304(4)	7762(5)	1356(3)	18(1)
C(22)	1667(6)	7188(5)	840(3)	23(1)
C(23)	583(7)	7798(7)	550(3)	25(1)
C(24)	137(19)	9024(15)	776(11)	20(2)
C(25)	740(10)	9610(10)	1298(5)	25(2)
C(26)	1839(5)	8983(6)	1578(3)	22(1)
Br(1A)	-1479(7)	10065(7)	523(4)	34(1)
C(19A)	4320(9)	6780(11)	1638(5)	20(2)
C(20A)	3239(8)	6465(10)	1297(5)	21(2)
C(21A)	2146(10)	7376(11)	1106(6)	18(2)
C(22A)	1260(13)	6987(12)	640(7)	27(2)
C(23A)	214(16)	7791(16)	442(8)	25(3)
C(24A)	10(40)	9000(40)	760(30)	23(4)
C(25A)	920(20)	9470(30)	1197(12)	27(4)
C(26A)	1961(12)	8602(14)	1391(7)	26(2)

Table SI 18: Bond lengths [Å] and angles [°] for 1,4-diene **7f**.

C(1)-C(19)	1.492(5)	C(17)-H(17B)	0.9800
C(1)-C(2)	1.522(4)	C(17)-H(17C)	0.9800
C(1)-C(10)	1.524(4)	C(18)-H(18A)	0.9800
C(1)-C(19A)	1.643(9)	C(18)-H(18B)	0.9800
C(1)-H(1)	1.0000	C(18)-H(18C)	0.9800
C(1)-H(1A)	1.0000	C(16A)-C(18A)	1.537(16)
C(2)-C(3)	1.337(5)	C(16A)-C(17A)	1.538(16)
C(2)-C(4)	1.488(4)	C(16A)-H(16A)	1.0000
C(3)-H(3A)	0.9500	C(17A)-H(17D)	0.9800
C(3)-H(3B)	0.9500	C(17A)-H(17E)	0.9800
C(4)-C(5)	1.394(4)	C(17A)-H(17F)	0.9800
C(4)-C(9)	1.398(5)	C(18A)-H(18D)	0.9800
C(5)-C(6)	1.393(5)	C(18A)-H(18E)	0.9800
C(5)-H(5)	0.9500	C(18A)-H(18F)	0.9800
C(6)-C(7)	1.381(6)	Br(1)-C(24)	1.896(7)
C(6)-H(6)	0.9500	C(19)-C(20)	1.320(6)
C(7)-C(8)	1.395(5)	C(19)-H(19)	0.9500
C(7)-H(7)	0.9500	C(20)-C(21)	1.479(6)
C(8)-C(9)	1.384(5)	C(20)-H(20)	0.9500
C(8)-H(8)	0.9500	C(21)-C(22)	1.383(8)
C(9)-H(9)	0.9500	C(21)-C(26)	1.386(7)
C(10)-C(11)	1.390(4)	C(22)-C(23)	1.382(8)
C(10)-C(15)	1.392(4)	C(22)-H(22)	0.9500
C(11)-C(12)	1.390(4)	C(23)-C(24)	1.388(8)
C(11)-H(11)	0.9500	C(23)-H(23)	0.9500
C(12)-C(13)	1.391(4)	C(24)-C(25)	1.383(9)
C(12)-H(12)	0.9500	C(25)-C(26)	1.391(7)
C(13)-C(14)	1.395(4)	C(25)-H(25)	0.9500
C(13)-C(16)	1.512(12)	C(26)-H(26)	0.9500
C(13)-C(16A)	1.519(13)	Br(1A)-C(24A)	1.892(16)
C(14)-C(15)	1.381(4)	C(19A)-C(20A)	1.330(12)
C(14)-H(14)	0.9500	C(19A)-H(19A)	0.9500
C(15)-H(15)	0.9500	C(20A)-C(21A)	1.473(12)
C(16)-C(17)	1.530(14)	C(20A)-H(20A)	0.9500
C(16)-C(18)	1.536(13)	C(21A)-C(22A)	1.375(16)
C(16)-H(16)	1.0000	C(21A)-C(26A)	1.378(15)
C(17)-H(17A)	0.9800	C(22A)-C(23A)	1.379(15)

C(22A)-H(22A)	0.9500
C(23A)-C(24A)	1.390(18)
C(23A)-H(23A)	0.9500
C(24A)-C(25A)	1.382(19)
C(25A)-C(26A)	1.408(17)
C(25A)-H(25A)	0.9500
C(26A)-H(26A)	0.9500
C(19)-C(1)-C(2)	113.0(3)
C(19)-C(1)-C(10)	117.8(3)
C(2)-C(1)-C(10)	111.6(3)
C(2)-C(1)-C(19A)	109.6(4)
C(10)-C(1)-C(19A)	97.2(4)
C(19)-C(1)-H(1)	104.3
C(2)-C(1)-H(1)	104.3
C(10)-C(1)-H(1)	104.3
C(2)-C(1)-H(1A)	112.5
C(10)-C(1)-H(1A)	112.5
C(19A)-C(1)-H(1A)	112.5
C(3)-C(2)-C(4)	121.1(3)
C(3)-C(2)-C(1)	122.4(3)
C(4)-C(2)-C(1)	116.5(3)
C(2)-C(3)-H(3A)	120.0
C(2)-C(3)-H(3B)	120.0
H(3A)-C(3)-H(3B)	120.0
C(5)-C(4)-C(9)	118.6(3)
C(5)-C(4)-C(2)	120.3(3)
C(9)-C(4)-C(2)	121.1(3)
C(6)-C(5)-C(4)	120.6(3)
C(6)-C(5)-H(5)	119.7
C(4)-C(5)-H(5)	119.7
C(7)-C(6)-C(5)	120.2(3)
C(7)-C(6)-H(6)	119.9
C(5)-C(6)-H(6)	119.9
C(6)-C(7)-C(8)	119.9(3)
C(6)-C(7)-H(7)	120.0
C(8)-C(7)-H(7)	120.0
C(9)-C(8)-C(7)	119.8(3)
C(9)-C(8)-H(8)	120.1
C(7)-C(8)-H(8)	120.1
C(8)-C(9)-C(4)	120.9(3)
C(8)-C(9)-H(9)	119.5
C(4)-C(9)-H(9)	119.5
C(11)-C(10)-C(15)	117.6(3)
C(11)-C(10)-C(1)	122.9(3)
C(15)-C(10)-C(1)	119.6(3)
C(12)-C(11)-C(10)	121.1(3)
C(12)-C(11)-H(11)	119.5
C(10)-C(11)-H(11)	119.5
C(11)-C(12)-C(13)	121.4(3)
C(11)-C(12)-H(12)	119.3
C(13)-C(12)-H(12)	119.3
C(12)-C(13)-C(14)	117.3(3)
C(12)-C(13)-C(16)	120.6(9)
C(14)-C(13)-C(16)	122.0(9)
C(12)-C(13)-C(16A)	121.7(11)
C(14)-C(13)-C(16A)	120.8(11)
C(15)-C(14)-C(13)	121.3(3)
C(15)-C(14)-H(14)	119.3
C(13)-C(14)-H(14)	119.3
C(14)-C(15)-C(10)	121.4(3)
C(14)-C(15)-H(15)	119.3
C(10)-C(15)-H(15)	119.3
C(13)-C(16)-C(17)	110.4(14)
C(13)-C(16)-C(18)	110.9(14)
C(17)-C(16)-C(18)	111.2(13)
C(13)-C(16)-H(16)	108.1
C(17)-C(16)-H(16)	108.1
C(18)-C(16)-H(16)	108.1
C(16)-C(17)-H(17A)	109.5
C(16)-C(17)-H(17B)	109.5
H(17A)-C(17)-H(17B)	109.5
C(16)-C(17)-H(17C)	109.5
H(17A)-C(17)-H(17C)	109.5
H(17B)-C(17)-H(17C)	109.5
C(16)-C(18)-H(18A)	109.5
C(16)-C(18)-H(18B)	109.5
H(18A)-C(18)-H(18B)	109.5

C(16)-C(18)-H(18C)	109.5
H(18A)-C(18)-H(18C)	109.5
H(18B)-C(18)-H(18C)	109.5
C(13)-C(16A)-C(18A)	112.3(18)
C(13)-C(16A)-C(17A)	112.0(17)
C(18A)-C(16A)-C(17A)	111.1(15)
C(13)-C(16A)-H(16A)	107.0
C(18A)-C(16A)-H(16A)	107.0
C(17A)-C(16A)-H(16A)	107.0
C(16A)-C(17A)-H(17D)	109.5
C(16A)-C(17A)-H(17E)	109.5
H(17D)-C(17A)-H(17E)	109.5
C(16A)-C(17A)-H(17F)	109.5
H(17D)-C(17A)-H(17F)	109.5
H(17E)-C(17A)-H(17F)	109.5
C(16A)-C(18A)-H(18D)	109.5
C(16A)-C(18A)-H(18E)	109.5
H(18D)-C(18A)-H(18E)	109.5
C(16A)-C(18A)-H(18F)	109.5
H(18D)-C(18A)-H(18F)	109.5
H(18E)-C(18A)-H(18F)	109.5
C(20)-C(19)-C(1)	123.1(4)
C(20)-C(19)-H(19)	118.5
C(1)-C(19)-H(19)	118.5
C(19)-C(20)-C(21)	126.5(4)
C(19)-C(20)-H(20)	116.8
C(21)-C(20)-H(20)	116.8
C(22)-C(21)-C(26)	118.6(4)
C(22)-C(21)-C(20)	123.2(5)
C(26)-C(21)-C(20)	118.2(5)
C(23)-C(22)-C(21)	121.5(5)
C(23)-C(22)-H(22)	119.3
C(21)-C(22)-H(22)	119.3
C(22)-C(23)-C(24)	119.1(6)
C(22)-C(23)-H(23)	120.4
C(24)-C(23)-H(23)	120.4
C(25)-C(24)-C(23)	120.6(7)
C(25)-C(24)-Br(1)	118.8(6)
C(23)-C(24)-Br(1)	120.5(6)
C(24)-C(25)-C(26)	119.1(7)
C(24)-C(25)-H(25)	120.5
C(26)-C(25)-H(25)	120.5
C(21)-C(26)-C(25)	121.1(5)
C(21)-C(26)-H(26)	119.5
C(25)-C(26)-H(26)	119.5
C(20A)-C(19A)-C(1)	117.9(9)
C(20A)-C(19A)-H(19A)	121.0
C(1)-C(19A)-H(19A)	121.0
C(19A)-C(20A)-C(21A)	126.6(10)
C(19A)-C(20A)-H(20A)	116.7
C(21A)-C(20A)-H(20A)	116.7
C(22A)-C(21A)-C(26A)	118.5(9)
C(22A)-C(21A)-C(20A)	119.5(11)
C(26A)-C(21A)-C(20A)	122.0(11)
C(21A)-C(22A)-C(23A)	122.3(11)
C(21A)-C(22A)-H(22A)	118.8
C(23A)-C(22A)-H(22A)	118.8
C(22A)-C(23A)-C(24A)	118.1(14)
C(22A)-C(23A)-H(23A)	121.0
C(24A)-C(23A)-H(23A)	121.0
C(25A)-C(24A)-C(23A)	121.5(16)
C(25A)-C(24A)-Br(1A)	119.7(14)
C(23A)-C(24A)-Br(1A)	118.6(13)
C(24A)-C(25A)-C(26A)	117.6(17)
C(24A)-C(25A)-H(25A)	121.2
C(26A)-C(25A)-H(25A)	121.2
C(21A)-C(26A)-C(25A)	121.4(12)
C(21A)-C(26A)-H(26A)	119.3
C(25A)-C(26A)-H(26A)	119.3

Table SI 19: Anisotropic displacement parameters ($Å^2 x 10^3$) for 1,4-diene **7f**. The anisotropic displacement factor exponent takes the form: $-2p^2[h^2 a^{*2}U^{11} + ... + 2 h k a^* b^* U^{12}]$

	U^{11}	U^{22}	U^{33}	U^{23}	U^{13}	U^{12}
C(1)	15(1)	45(2)	27(1)	5(1)	-4(1)	6(1)
C(2)	19(1)	41(2)	25(1)	1(1)	-2(1)	5(1)
C(3)	28(2)	46(2)	33(2)	10(2)	1(1)	6(1)
C(4)	19(1)	34(2)	22(1)	0(1)	-1(1)	5(1)
C(5)	22(1)	34(2)	26(1)	-2(1)	1(1)	2(1)
C(6)	16(1)	46(2)	34(2)	-3(1)	0(1)	6(1)
C(7)	25(2)	41(2)	42(2)	-2(2)	0(1)	12(1)
C(8)	32(2)	33(2)	50(2)	-2(1)	2(2)	5(2)
C(9)	22(1)	37(2)	34(2)	-4(1)	-1(1)	1(1)
C(10)	16(1)	20(1)	28(1)	2(1)	-1(1)	6(1)
C(11)	17(1)	22(1)	31(1)	3(1)	3(1)	1(1)
C(12)	17(1)	22(1)	37(2)	-3(1)	2(1)	-1(1)
C(13)	20(1)	24(1)	30(1)	-3(1)	-2(1)	-1(1)
C(14)	24(1)	27(1)	29(1)	2(1)	1(1)	-7(1)
C(15)	21(1)	20(1)	30(1)	1(1)	-1(1)	-2(1)
C(16)	28(3)	36(4)	28(2)	-8(2)	3(1)	-4(2)
C(17)	98(13)	48(5)	25(4)	3(3)	1(7)	21(7)
C(18)	30(4)	50(8)	31(6)	-19(5)	4(4)	-6(5)
C(16A)	28(3)	36(4)	28(2)	-8(2)	3(1)	-4(2)
C(17A)	43(7)	51(7)	32(6)	-4(5)	0(5)	-4(5)
C(18A)	28(5)	62(12)	43(9)	-17(8)	6(5)	-5(6)
Br(1)	20(1)	34(1)	33(1)	5(1)	-8(1)	7(1)
C(19)	19(2)	26(2)	23(2)	-3(2)	-5(2)	2(2)
C(20)	19(2)	23(2)	21(2)	2(1)	-5(1)	1(2)
C(21)	15(2)	19(2)	22(2)	-2(2)	-1(2)	3(2)
C(22)	20(2)	23(2)	25(3)	-5(2)	-4(2)	4(2)
C(23)	23(3)	29(2)	23(3)	-5(2)	-7(2)	1(2)
C(24)	12(4)	21(3)	26(3)	6(2)	-2(3)	5(3)
C(25)	18(3)	24(3)	31(3)	-3(2)	-4(3)	6(2)
C(26)	22(2)	21(2)	23(2)	-5(2)	-7(2)	5(2)
Br(1A)	17(1)	35(2)	50(3)	7(1)	-2(1)	7(1)
C(19A)	14(4)	15(4)	31(4)	-1(4)	-6(3)	4(3)
C(20A)	11(3)	23(4)	30(4)	-2(3)	-2(3)	0(3)
C(21A)	16(4)	21(4)	18(4)	-5(3)	-4(3)	2(3)

C(22A)	21(5)	28(5)	31(5)	-8(4)	-9(4)	4(4)
C(23A)	20(6)	32(5)	24(6)	3(4)	-3(4)	6(4)
C(24A)	16(7)	26(6)	27(7)	2(5)	1(5)	-1(5)
C(25A)	19(6)	26(6)	36(7)	-5(5)	2(5)	4(4)
C(26A)	22(4)	26(5)	29(5)	-13(4)	-6(4)	3(4)

Table SI 20: Hydrogen coordinates (x 10^4) and isotropic displacement parameters ($Å^2$x 10^3) for 1,4-diene **7f**.

	x	y	z	U(eq)
H(1)	5208	4581	1827	35
H(1A)	4961	4663	1761	35
H(3A)	7735	6831	864	43
H(3B)	6156	7296	945	43
H(5)	9272	6269	1704	33
H(6)	11038	4833	1974	38
H(7)	10640	2554	2109	43
H(8)	8465	1694	1962	46
H(9)	6703	3124	1698	38
H(11)	6926	7343	2476	28
H(12)	7051	7823	3552	30
H(14)	4164	5082	3901	32
H(15)	4040	4605	2829	29
H(16)	6420	7537	4602	37
H(17A)	5249	5003	4914	86
H(17B)	6113	5910	5388	86
H(17C)	6822	5222	4789	86
H(18A)	4238	8384	4528	55
H(18B)	4385	7702	5211	55
H(18C)	3599	6940	4656	55
H(16A)	6421	7491	4611	37
H(17D)	5795	4736	4825	63
H(17E)	6435	5697	5351	63
H(17F)	7274	5334	4726	63
H(18D)	4089	7962	4647	66
H(18E)	4601	7389	5313	66
H(18F)	3787	6442	4837	66

H(19)	3959	5734	1080	27
H(20)	3698	7498	2086	25
H(22)	1982	6355	682	28
H(23)	147	7383	200	30
H(25)	407	10430	1463	30
H(26)	2278	9397	1926	27
H(19A)	4487	7677	1764	24
H(20A)	3164	5561	1163	26
H(22A)	1372	6135	448	32
H(23A)	-354	7528	102	30
H(25A)	854	10350	1364	32
H(26A)	2544	8867	1725	31

10.5 1,4-diene 9

The crystal structure of **9** was solved in the monoclinic space group $P2_1/c$. The asymmetric unit contains one full molecule. Crystals suitable for SCXRD analysis were obtained by crystallization from a saturated *n*-hexane solution at room temperature.

Table SI 21: Crystal data and structure refinement for **9**.

CCDC No	2150155	
Empirical formula	C_{32} H_{30}	
Formula weight	414.56	
Temperature	100(2) K	
Wavelength	0.71073 Å	
Crystal system	Monoclinic	
Space group	$P2_1/c$	
Unit cell dimensions	a = 17.4804(17) Å	α= 90°.
	b = 5.6492(6) Å	β= 95.922(3)°.
	c = 23.189(2) Å	γ = 90°.
Volume	2277.7(4) Å^3	
Z	4	
Density (calculated)	1.209 Mg/m^3	
Absorption coefficient	0.068 mm^{-1}	
F(000)	888	
Crystal size	0.287 x 0.088 x 0.077 mm^3	
Theta range for data collection	1.766 to 27.876°.	

Index ranges	$-23 \le h \le 23$, $-7 \le k \le 7$, $-30 \le l \le 30$
Reflections collected	53956
Independent reflections	5435 [R(int) = 0.0668]
Completeness to theta = 25.242°	100.0 %
Absorption correction	Semi-empirical from equivalents
Refinement method	Full-matrix least-squares on F^2
Data / restraints / parameters	5435 / 0 / 292
Goodness-of-fit on F^2	1.011
Final R indices [I>2σ(I)]	R1 = 0.0513, wR2 = 0.1125
R indices (all data)	R1 = 0.0758, wR2 = 0.1259
Extinction coefficient	0.0102(10)
Largest diff. peak and hole	0.295 and -0.221 e.Å^{-3}

Table SI 22: Atomic coordinates (x 10^4) and equivalent isotropic displacement parameters (Å^{2}x 10^3) for 1,4-diene **9**. U(eq) is defined as one third of the trace of the orthogonalized U^{ij} tensor.

	x	y	z	U(eq)
C(1)	7085(1)	7343(3)	5206(1)	15(1)
C(2)	6949(1)	5563(3)	5679(1)	15(1)
C(3)	6322(1)	4213(3)	5636(1)	20(1)
C(4)	7511(1)	5507(3)	6211(1)	15(1)
C(5)	8028(1)	7359(3)	6349(1)	18(1)
C(6)	8529(1)	7311(3)	6855(1)	19(1)
C(7)	8530(1)	5407(3)	7231(1)	20(1)
C(8)	8024(1)	3537(3)	7100(1)	20(1)
C(9)	7526(1)	3584(3)	6595(1)	18(1)
C(10)	7856(1)	7038(3)	4950(1)	15(1)
C(11)	8295(1)	4987(3)	5016(1)	16(1)
C(12)	8942(1)	4701(3)	4716(1)	17(1)
C(13)	9165(1)	6430(3)	4342(1)	17(1)
C(14)	8732(1)	8514(3)	4289(1)	19(1)
C(15)	8092(1)	8810(3)	4590(1)	18(1)
C(16)	9822(1)	5959(3)	3975(1)	21(1)
C(17)	10320(1)	8128(3)	3894(1)	29(1)
C(18)	9496(1)	4940(4)	3385(1)	27(1)
C(19)	6468(1)	7257(3)	4699(1)	16(1)
C(20)	5969(1)	8981(3)	4549(1)	16(1)

C(21)	5399(1)	9002(3)	4036(1)	15(1)
C(22)	5363(1)	7225(3)	3614(1)	18(1)
C(23)	4825(1)	7304(3)	3132(1)	18(1)
C(24)	4296(1)	9172(3)	3042(1)	14(1)
C(25)	4334(1)	10943(3)	3465(1)	19(1)
C(26)	4872(1)	10853(3)	3949(1)	20(1)
C(27)	3726(1)	9239(3)	2518(1)	14(1)
C(28)	3674(1)	7387(3)	2114(1)	19(1)
C(29)	3143(1)	7444(3)	1626(1)	20(1)
C(30)	2652(1)	9358(3)	1525(1)	18(1)
C(31)	2696(1)	11215(3)	1916(1)	20(1)
C(32)	3225(1)	11154(3)	2409(1)	18(1)

Table SI 23: Bond lengths [Å] and angles [°] for **9**.

C(1)-C(19)	1.510(2)	C(12)-C(13)	1.389(2)
C(1)-C(2)	1.526(2)	C(12)-H(12)	0.9500
C(1)-C(10)	1.536(2)	C(13)-C(14)	1.397(2)
C(1)-H(1)	1.0000	C(13)-C(16)	1.521(2)
C(2)-C(3)	1.331(2)	C(14)-C(15)	1.388(2)
C(2)-C(4)	1.495(2)	C(14)-H(14)	0.9500
C(3)-H(3A)	0.9500	C(15)-H(15)	0.9500
C(3)-H(3B)	0.9500	C(16)-C(17)	1.526(2)
C(4)-C(5)	1.398(2)	C(16)-C(18)	1.539(2)
C(4)-C(9)	1.404(2)	C(16)-H(16)	1.0000
C(5)-C(6)	1.390(2)	C(17)-H(17A)	0.9800
C(5)-H(5)	0.9500	C(17)-H(17B)	0.9800
C(6)-C(7)	1.384(2)	C(17)-H(17C)	0.9800
C(6)-H(6)	0.9500	C(18)-H(18A)	0.9800
C(7)-C(8)	1.391(2)	C(18)-H(18B)	0.9800
C(7)-H(7)	0.9500	C(18)-H(18C)	0.9800
C(8)-C(9)	1.385(2)	C(19)-C(20)	1.329(2)
C(8)-H(8)	0.9500	C(19)-H(19)	0.9500
C(9)-H(9)	0.9500	C(20)-C(21)	1.473(2)
C(10)-C(11)	1.390(2)	C(20)-H(20)	0.9500
C(10)-C(15)	1.394(2)	C(21)-C(26)	1.394(2)
C(11)-C(12)	1.398(2)	C(21)-C(22)	1.398(2)
C(11)-H(11)	0.9500	C(22)-C(23)	1.385(2)

C(22)-H(22)	0.9500
C(23)-C(24)	1.404(2)
C(23)-H(23)	0.9500
C(24)-C(25)	1.398(2)
C(24)-C(27)	1.491(2)
C(25)-C(26)	1.389(2)
C(25)-H(25)	0.9500
C(26)-H(26)	0.9500
C(27)-C(32)	1.399(2)
C(27)-C(28)	1.401(2)
C(28)-C(29)	1.388(2)
C(28)-H(28)	0.9500
C(29)-C(30)	1.386(2)
C(29)-H(29)	0.9500
C(30)-C(31)	1.383(2)
C(30)-H(30)	0.9500
C(31)-C(32)	1.394(2)
C(31)-H(31)	0.9500
C(32)-H(32)	0.9500

C(19)-C(1)-C(2)	112.76(12)
C(19)-C(1)-C(10)	106.30(11)
C(2)-C(1)-C(10)	114.29(12)
C(19)-C(1)-H(1)	107.7
C(2)-C(1)-H(1)	107.7
C(10)-C(1)-H(1)	107.7
C(3)-C(2)-C(4)	121.10(14)
C(3)-C(2)-C(1)	120.77(13)
C(4)-C(2)-C(1)	117.98(13)
C(2)-C(3)-H(3A)	120.0
C(2)-C(3)-H(3B)	120.0
H(3A)-C(3)-H(3B)	120.0
C(5)-C(4)-C(9)	117.66(14)
C(5)-C(4)-C(2)	121.84(14)
C(9)-C(4)-C(2)	120.50(14)
C(6)-C(5)-C(4)	120.99(14)
C(6)-C(5)-H(5)	119.5
C(4)-C(5)-H(5)	119.5
C(7)-C(6)-C(5)	120.44(15)
C(7)-C(6)-H(6)	119.8
C(5)-C(6)-H(6)	119.8
C(6)-C(7)-C(8)	119.57(14)
C(6)-C(7)-H(7)	120.2
C(8)-C(7)-H(7)	120.2
C(9)-C(8)-C(7)	119.95(15)
C(9)-C(8)-H(8)	120.0
C(7)-C(8)-H(8)	120.0
C(8)-C(9)-C(4)	121.39(15)
C(8)-C(9)-H(9)	119.3
C(4)-C(9)-H(9)	119.3
C(11)-C(10)-C(15)	118.03(13)
C(11)-C(10)-C(1)	123.22(13)
C(15)-C(10)-C(1)	118.47(13)
C(10)-C(11)-C(12)	120.55(14)
C(10)-C(11)-H(11)	119.7
C(12)-C(11)-H(11)	119.7
C(13)-C(12)-C(11)	121.54(14)
C(13)-C(12)-H(12)	119.2
C(11)-C(12)-H(12)	119.2
C(12)-C(13)-C(14)	117.57(14)
C(12)-C(13)-C(16)	120.16(14)
C(14)-C(13)-C(16)	122.13(14)
C(15)-C(14)-C(13)	121.00(14)
C(15)-C(14)-H(14)	119.5
C(13)-C(14)-H(14)	119.5
C(14)-C(15)-C(10)	121.25(14)
C(14)-C(15)-H(15)	119.4
C(10)-C(15)-H(15)	119.4
C(13)-C(16)-C(17)	113.73(14)
C(13)-C(16)-C(18)	109.27(12)
C(17)-C(16)-C(18)	110.72(14)
C(13)-C(16)-H(16)	107.6
C(17)-C(16)-H(16)	107.6
C(18)-C(16)-H(16)	107.6
C(16)-C(17)-H(17A)	109.5
C(16)-C(17)-H(17B)	109.5
H(17A)-C(17)-H(17B)	109.5
C(16)-C(17)-H(17C)	109.5

H(17A)-C(17)-H(17C)	109.5
H(17B)-C(17)-H(17C)	109.5
C(16)-C(18)-H(18A)	109.5
C(16)-C(18)-H(18B)	109.5
H(18A)-C(18)-H(18B)	109.5
C(16)-C(18)-H(18C)	109.5
H(18A)-C(18)-H(18C)	109.5
H(18B)-C(18)-H(18C)	109.5
C(20)-C(19)-C(1)	125.17(14)
C(20)-C(19)-H(19)	117.4
C(1)-C(19)-H(19)	117.4
C(19)-C(20)-C(21)	126.09(14)
C(19)-C(20)-H(20)	117.0
C(21)-C(20)-H(20)	117.0
C(26)-C(21)-C(22)	117.18(13)
C(26)-C(21)-C(20)	120.34(14)
C(22)-C(21)-C(20)	122.48(14)
C(23)-C(22)-C(21)	121.23(14)
C(23)-C(22)-H(22)	119.4
C(21)-C(22)-H(22)	119.4
C(22)-C(23)-C(24)	121.73(14)
C(22)-C(23)-H(23)	119.1
C(24)-C(23)-H(23)	119.1
C(25)-C(24)-C(23)	116.79(13)
C(25)-C(24)-C(27)	122.23(13)
C(23)-C(24)-C(27)	120.98(13)
C(26)-C(25)-C(24)	121.33(14)
C(26)-C(25)-H(25)	119.3
C(24)-C(25)-H(25)	119.3
C(25)-C(26)-C(21)	121.74(14)
C(25)-C(26)-H(26)	119.1
C(21)-C(26)-H(26)	119.1
C(32)-C(27)-C(28)	117.46(13)
C(32)-C(27)-C(24)	121.35(13)
C(28)-C(27)-C(24)	121.19(13)
C(29)-C(28)-C(27)	121.31(14)
C(29)-C(28)-H(28)	119.3
C(27)-C(28)-H(28)	119.3
C(30)-C(29)-C(28)	120.40(15)
C(30)-C(29)-H(29)	119.8
C(28)-C(29)-H(29)	119.8
C(31)-C(30)-C(29)	119.30(14)
C(31)-C(30)-H(30)	120.3
C(29)-C(30)-H(30)	120.3
C(30)-C(31)-C(32)	120.42(15)
C(30)-C(31)-H(31)	119.8
C(32)-C(31)-H(31)	119.8
C(31)-C(32)-C(27)	121.10(14)
C(31)-C(32)-H(32)	119.4
C(27)-C(32)-H(32)	119.4

Table SI 24: Anisotropic displacement parameters (Å^{2}x 10^3) for 1,4-diene **9**. The anisotropic displacement factor exponent takes the form: -2p^2[h^2 a*^{2}U^{11} + ... + 2 h k a* b* U^{12}]

	U^{11}	U^{22}	U^{33}	U^{23}	U^{13}	U^{12}
C(1)	17(1)	14(1)	13(1)	-1(1)	1(1)	1(1)
C(2)	15(1)	16(1)	14(1)	-2(1)	4(1)	2(1)
C(3)	19(1)	23(1)	17(1)	-1(1)	1(1)	-1(1)
C(4)	15(1)	17(1)	15(1)	-1(1)	4(1)	2(1)
C(5)	19(1)	18(1)	17(1)	0(1)	3(1)	-1(1)
C(6)	19(1)	21(1)	18(1)	-3(1)	1(1)	-1(1)
C(7)	20(1)	27(1)	14(1)	-1(1)	2(1)	4(1)
C(8)	21(1)	22(1)	17(1)	6(1)	4(1)	2(1)
C(9)	16(1)	18(1)	20(1)	0(1)	5(1)	-1(1)
C(10)	15(1)	19(1)	11(1)	-2(1)	-1(1)	-2(1)
C(11)	18(1)	17(1)	13(1)	1(1)	1(1)	-2(1)
C(12)	16(1)	19(1)	14(1)	0(1)	-2(1)	2(1)
C(13)	14(1)	24(1)	14(1)	0(1)	-1(1)	-3(1)
C(14)	20(1)	19(1)	17(1)	3(1)	2(1)	-4(1)
C(15)	19(1)	16(1)	19(1)	0(1)	0(1)	0(1)
C(16)	16(1)	27(1)	20(1)	4(1)	3(1)	1(1)
C(17)	21(1)	35(1)	33(1)	7(1)	8(1)	-2(1)
C(18)	22(1)	40(1)	21(1)	-1(1)	6(1)	5(1)
C(19)	16(1)	18(1)	14(1)	-2(1)	2(1)	-1(1)
C(20)	16(1)	18(1)	14(1)	-2(1)	2(1)	-2(1)
C(21)	13(1)	18(1)	14(1)	1(1)	2(1)	-2(1)
C(22)	18(1)	19(1)	19(1)	-1(1)	1(1)	6(1)
C(23)	18(1)	20(1)	16(1)	-4(1)	1(1)	4(1)
C(24)	12(1)	16(1)	13(1)	2(1)	4(1)	-1(1)
C(25)	19(1)	17(1)	21(1)	-1(1)	-1(1)	5(1)
C(26)	23(1)	17(1)	19(1)	-4(1)	0(1)	2(1)
C(27)	13(1)	16(1)	14(1)	2(1)	4(1)	-2(1)
C(28)	18(1)	18(1)	20(1)	1(1)	1(1)	1(1)
C(29)	20(1)	22(1)	17(1)	-3(1)	0(1)	-2(1)
C(30)	14(1)	26(1)	15(1)	3(1)	1(1)	-2(1)
C(31)	16(1)	24(1)	19(1)	4(1)	2(1)	4(1)
C(32)	18(1)	19(1)	16(1)	-1(1)	3(1)	1(1)

Table SI 25: Hydrogen coordinates (x 10^4) and isotropic displacement parameters (Å^{2}x 10^3) for 1,4-diene **9**.

	x	y	z	U(eq)
H(1)	7078	8963	5379	18
H(3A)	6225	3191	5946	23
H(3B)	5970	4270	5295	23
H(5)	8037	8671	6093	21
H(6)	8873	8592	6943	23
H(7)	8874	5378	7576	24
H(8)	8020	2229	7357	24
H(9)	7189	2288	6507	22
H(11)	8154	3769	5267	19
H(12)	9237	3291	4770	20
H(14)	8878	9746	4044	23
H(15)	7810	10247	4549	22
H(16)	10160	4722	4178	25
H(17A)	10774	7653	3706	44
H(17B)	10484	8834	4272	44
H(17C)	10023	9289	3650	44
H(18A)	9145	6092	3183	41
H(18B)	9215	3473	3446	41
H(18C)	9919	4604	3151	41
H(19)	6435	5861	4469	19
H(20)	5982	10324	4796	19
H(22)	5715	5940	3659	22
H(23)	4813	6061	2855	22
H(25)	3985	12235	3420	23
H(26)	4881	12085	4229	24
H(28)	4009	6067	2175	22
H(29)	3117	6163	1359	23
H(30)	2288	9395	1191	22
H(31)	2365	12541	1848	24
H(32)	3245	12435	2676	21

11 Comparison between computed and experimental NMR shifts of 1,4-diene 3

Table SI 26 shows the computed and experimental NMR shifts of selected atoms for bispentafluoro-(*E*)-(3,5-diphenylpenta-1,4-dien-2-yl)borane. For the computational details see chapter 12.1.

Table SI 26: Selected computed and experimental NMR shifts of bispentafluoro-(*E*)-(3,5-diphenylpenta-1,4-dien-2-yl)borane.

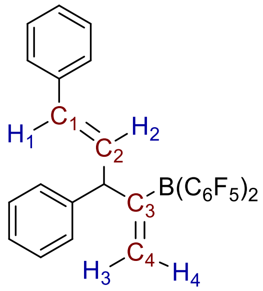

	H_1	H_2	H_3	H_4	C_1	C_2	C_3	C_4	B
computed NMR shifts [ppm]	6.34	6.16	6.30	6.02	137.2	137.1	168.7	148.2	67.9
experimental NMR shifts [ppm]	6.27	6.27	6.08	5.87	132.9	131.3	158.9	141.2	65.2

12 Computational details

Preoptimizations and initial modeling was done using Avogadro (ver. 1.2.0)[20] and the GFN2-xTB method[21] provided by the xTB-program (ver. 6.4.0).[22] All subsequentially computations were performed with Orca 5 (Versions 5.0.1 and 5.0.2).[23] Geometry optimizations were performed with PBEh-3c.[24] Frequency computations were also performed at this level of theory. Stationary points were identified by having either no (minima) or one (transition states) imaginary frequency. These computations were also used to acquire all used thermal corrections. The computed free energies were corrected regarding the standard state by adding RT ln(c0s/c0g) (i.e., about 1.89 kcal mol^{-1}) to energies of all structures.

Single point computations were performed with the revDSD-PBEP86 double-Hybrid Functional[25] with Grimmes D4 dispersion correction[26] and the def2-QZVPP basis set.[27] This refitted version of DSD-PBEP86 is one of the most accurate DFT methods for ground-state thermochemistry and kinetics as shown by benchmark computations using the GMTKN55 database.[28] The RIJCOS-X approximation was used during the single point calculations.[29] For these, the respective def2-QZVPP/C[30] and def2/J[31]

auxiliary basis sets were used. Solvation was considered implicitly with the C-PCM model for dichloromethane.[32]

Against the default ORCA setting, the larger defgrid3 was used throughout all computations. This was done to eliminate convergence problems in the optimizations of larger structures. Overall, the reduced performance introduced by the usage of the large grid was counterbalanced by a more robust convergence in fewer optimization steps.

12.1 NMR computations

A conformer analysis of bispentafluoro-(*E*)-(3,5-diphenylpenta-1,4-dien-2-yl)borane **3** was performed using the GFN2-xTB method provided by the xTB program.[21,33] The nine lowest lying conformers were reoptimized using the PBEh-3c composite method.[24] Frequency computations were used to ensure that the optimized structure is a minimum structure.

The electronic energy of the optimized structures was refined using the DLPNO-CCSD(T)[34] method with the def2-TZVP basis set.[27]

The NMR computations were done with the hybrid version of the Perdew-Burke-Ernzerhof functional containing 25 % exact HF exchange (PBE0)[35] using the GIAO method[36] in combination with the pcS-2 basis set.[37]

For obtaining the NMR shifts all nine conformers were weighed to their respective Boltzmann contribution calculated from the free energy difference where the most stable conformer was used as a reference point. The computed NMR shifts of the nine conformers were multiplied with their respective Boltzmann contribution, summed, and referenced to benzene for ^{1}H and ^{13}C or $H_3B\ NMe_3$ for ^{11}B shifts computed at the same level.

12.2 Cartesian coordinates and energies.

Coordinates are given in angstrom. All Energies are given in Hartree.

1 / Piers' borane / $HB(C_6F_5)_2$

5	3.271604000	-3.041249000	-2.162774000
6	4.175105000	-3.741676000	-1.114331000
6	3.973880000	-3.506886000	0.243406000
6	5.142580000	-4.688997000	-1.438510000
6	4.701087000	-4.147165000	1.226559000
6	5.875043000	-5.359905000	-0.480709000
6	5.652544000	-5.080844000	0.856913000
9	3.068406000	-2.617495000	0.631480000
9	5.362437000	-5.008538000	-2.708624000
9	4.496973000	-3.884826000	2.510924000
9	6.780603000	-6.267635000	-0.822328000
9	6.349750000	-5.711065000	1.784579000
6	3.693320000	-2.712164000	-3.619925000
6	4.984581000	-2.341415000	-3.981267000
6	2.740845000	-2.701803000	-4.634460000
6	5.322267000	-1.994737000	-5.273338000
6	3.047987000	-2.378881000	-5.941176000
6	4.346912000	-2.021607000	-6.255697000
9	5.938685000	-2.274980000	-3.059328000
9	1.486413000	-3.041256000	-4.362801000
9	6.561082000	-1.635427000	-5.584396000
9	2.120635000	-2.403131000	-6.889912000
9	4.658232000	-1.701921000	-7.498855000
1	2.172586000	-2.740082000	-1.828727000

E PCM(DCM)-PBEh-3c	-1478.127101
ZPVE	0.11754515
H	0.13733796
G	0.07194221
E PCM(DCM)-revDSD-PBEP86-D4/def2-QZVPP	-1479.663252

phenylacetylene

6	0.340928000	0.000000000	-5.198486000
6	-0.808521000	-0.352764000	-4.502780000
6	1.490377000	0.352764000	-4.502779000
1	-1.705953000	-0.627974000	-5.040997000
1	2.387809000	0.627974000	-5.040997000
6	-0.812590000	-0.353966000	-3.117297000
6	1.494446000	0.353966000	-3.117297000
1	-1.707782000	-0.628492000	-2.575032000
1	2.389638000	0.628492000	-2.575032000
6	0.340928000	0.000000000	-2.412477000
1	0.340928000	0.000000000	-6.280596000
6	0.340928000	0.000000000	-0.985454000
6	0.340928000	0.000001000	0.218139000

1	0.340928000	0.000000000	1.284027000	
E PCM(DCM)-PBEh-3c				-307.7030531
ZPVE				0.11298843
H				0.12015226
G				0.08338492
E PCM(DCM)-revDSD-PBEP86-D4/def2-QZVPP				-307.9558132

$TS_{1/2}$

6	0.435855000	3.817458000	-0.696901000	
5	0.243390000	2.131449000	0.248322000	
6	1.499155000	1.151981000	0.465024000	
6	2.620669000	1.475785000	1.207058000	
6	1.491408000	-0.111635000	-0.100342000	
6	3.673230000	0.597226000	1.394400000	
6	2.517847000	-1.021454000	0.066647000	
6	3.616560000	-0.660301000	0.824009000	
6	-1.191868000	1.551383000	0.688502000	
6	-1.409424000	1.274810000	2.029330000	
6	-2.255547000	1.286177000	-0.155309000	
6	-2.598340000	0.768428000	2.517873000	
6	-3.465213000	0.784382000	0.292513000	
6	-3.635350000	0.522356000	1.637746000	
9	0.450500000	-0.485160000	-0.849685000	
9	2.464190000	-2.227741000	-0.491007000	
9	4.614281000	-1.517854000	0.999028000	
9	4.732829000	0.946685000	2.118829000	
9	2.724633000	2.679038000	1.777200000	
9	-0.433864000	1.503768000	2.912694000	
9	-2.757004000	0.520918000	3.815106000	
9	-4.787171000	0.036833000	2.083666000	
9	-4.459446000	0.545330000	-0.558699000	
9	-2.149212000	1.508597000	-1.468585000	
1	0.268288000	2.274485000	-2.268600000	
6	0.296542000	2.729083000	-1.295654000	
1	0.410467000	3.128682000	0.954431000	
6	0.558534000	5.181723000	-0.292796000	
6	-0.584895000	5.982169000	-0.242328000	
6	1.811130000	5.713408000	0.022479000	
6	-0.469701000	7.314497000	0.115494000	
6	1.914189000	7.046033000	0.379499000	
6	0.776523000	7.843963000	0.426878000	
1	-1.549993000	5.557346000	-0.484578000	
1	2.690075000	5.084148000	-0.017005000	
1	-1.351373000	7.939717000	0.153055000	
1	2.881392000	7.464735000	0.622040000	
1	0.862398000	8.885315000	0.708148000	
E PCM(DCM)-PBEh-3c				-1785.843695
ZPVE				0.23173917
H				0.25831854
G				0.17849113
E PCM(DCM)-revDSD-PBEP86-D4/def2-QZVPP				-1787.624823

phenylallene

6	9.928090000	-4.545804000	-2.224308000
6	8.713219000	-4.981049000	-2.378717000
1	8.303061000	-5.175122000	-3.363651000
1	8.064466000	-5.163083000	-1.529062000
6	11.151361000	-4.106737000	-2.074276000
6	12.345603000	-4.946774000	-1.916070000
6	12.279794000	-6.342171000	-1.900199000
6	13.428837000	-7.099617000	-1.749691000
6	14.666879000	-6.480567000	-1.612049000
6	14.743318000	-5.095496000	-1.626216000
6	13.591725000	-4.335548000	-1.776738000
1	13.659524000	-3.254044000	-1.786507000
1	15.701140000	-4.602793000	-1.519625000
1	15.563126000	-7.075534000	-1.494428000
1	13.360124000	-8.179858000	-1.739357000
1	11.321401000	-6.836165000	-2.006572000
1	11.308142000	-3.031276000	-2.061821000

E PCM(DCM)-PBEh-3c	-346.9392812
ZPVE	0.14155009
H	0.15005911
G	0.13441665
E PCM(DCM)-revDSD-PBEP86-D4/def2-QZVPP	-347.2156095

$TS_{2/5}$

5	0.416655000	-0.074946000	-0.183685000
6	0.249935000	-0.528119000	-2.064891000
6	0.788332000	0.203478000	-3.020294000
1	0.846002000	-0.188115000	-4.029699000
1	1.237094000	1.170735000	-2.847058000
6	-0.490357000	-1.662630000	-1.907692000
6	-1.881065000	-1.811714000	-2.214803000
6	-2.597656000	-0.831963000	-2.918488000
6	-3.933313000	-1.029819000	-3.204023000
6	-4.570360000	-2.195542000	-2.785664000
6	-3.871743000	-3.173553000	-2.086355000
6	-2.533558000	-2.983310000	-1.800789000
1	-1.979661000	-3.736392000	-1.253479000
1	-4.373233000	-4.076160000	-1.765231000
1	-5.620019000	-2.340237000	-3.005640000
1	-4.487687000	-0.276252000	-3.746481000
1	-2.102333000	0.075447000	-3.234038000
1	-0.029559000	-2.497595000	-1.382310000
6	0.663056000	1.522125000	-0.232082000
6	-0.215622000	2.375921000	-0.887003000
6	-0.082710000	3.751850000	-0.892114000
6	0.956546000	4.335154000	-0.194188000
6	1.833243000	3.532213000	0.506334000
6	1.667409000	2.158080000	0.482862000

9	2.518777000	1.454988000	1.233655000
9	2.821388000	4.085026000	1.205132000
9	1.100567000	5.654270000	-0.185007000
9	-0.948433000	4.517261000	-1.551696000
9	-1.267340000	1.884036000	-1.545628000
6	1.702088000	-1.000540000	0.176304000
6	1.671477000	-2.034906000	1.103331000
6	2.765520000	-2.832186000	1.398876000
6	3.964348000	-2.612713000	0.755279000
6	4.051780000	-1.592500000	-0.171883000
6	2.939455000	-0.817841000	-0.434963000
9	3.115028000	0.168389000	-1.314949000
9	5.205941000	-1.360985000	-0.792587000
9	5.022214000	-3.365525000	1.026552000
9	2.667669000	-3.804824000	2.301912000
9	0.561653000	-2.318433000	1.787795000
6	-0.938790000	-0.410464000	0.582197000
1	-1.352355000	-1.413183000	0.508009000
6	-1.640589000	0.442729000	1.336598000
1	-1.267765000	1.450570000	1.501677000
6	-2.904425000	0.143634000	2.022101000
6	-3.727731000	-0.925465000	1.653720000
6	-3.323033000	0.962235000	3.073697000
6	-4.909808000	-1.178279000	2.329951000
6	-4.505683000	0.709454000	3.753772000
6	-5.303972000	-0.364734000	3.386366000
1	-3.453476000	-1.557226000	0.817359000
1	-2.707297000	1.805011000	3.366078000
1	-5.533559000	-2.008896000	2.024839000
1	-4.804901000	1.354636000	4.569874000
1	-6.229481000	-0.562600000	3.911316000

E PCM(DCM)-PBEh-3c	-2132.849436
ZPVE	0.37993078
H	0.41460442
G	0.31949364
E PCM(DCM)-revDSD-PBEP86-D4/def2-QZVPP	-2134.905799

5

5	7.595327000	-2.350058000	0.032597000
6	8.654932000	-3.315588000	-0.819868000
6	8.312828000	-4.262064000	-1.728166000
1	9.034706000	-4.692302000	-2.413527000
1	7.283069000	-4.551507000	-1.892424000
6	10.017915000	-2.979376000	-0.720778000
6	11.119394000	-3.861345000	-0.782616000
6	10.979363000	-5.259987000	-0.651576000
6	12.079493000	-6.077482000	-0.803476000
6	13.326450000	-5.519222000	-1.070502000
6	13.490561000	-4.137825000	-1.165508000
6	12.401853000	-3.312047000	-1.001936000
1	12.512943000	-2.237147000	-1.070451000

1	14.469048000	-3.719959000	-1.356871000
1	14.186821000	-6.166219000	-1.181693000
1	11.978961000	-7.148036000	-0.691895000
1	10.023070000	-5.683054000	-0.375764000
1	10.264210000	-1.931867000	-0.555690000
6	6.110308000	-3.026653000	0.090280000
6	5.964815000	-4.346953000	0.494905000
6	4.744513000	-4.979356000	0.642091000
6	3.583967000	-4.271888000	0.396519000
6	3.671109000	-2.947337000	0.021243000
6	4.916164000	-2.355474000	-0.115962000
9	4.906959000	-1.057519000	-0.438544000
9	2.558085000	-2.248747000	-0.196846000
9	2.398276000	-4.856176000	0.533026000
9	4.675758000	-6.253340000	1.024856000
9	7.046069000	-5.078794000	0.792505000
6	7.649507000	-0.938670000	-0.813998000
6	8.131510000	0.270048000	-0.337432000
6	8.217940000	1.417284000	-1.112223000
6	7.809857000	1.385969000	-2.428510000
6	7.318214000	0.205500000	-2.952542000
6	7.249446000	-0.911433000	-2.144411000
9	6.743719000	-2.016126000	-2.704822000
9	6.914916000	0.161894000	-4.221202000
9	7.885514000	2.476040000	-3.184434000
9	8.693214000	2.549793000	-0.596279000
9	8.549250000	0.400174000	0.925717000
6	8.159647000	-2.216616000	1.537830000
1	9.231984000	-2.045898000	1.655687000
6	7.457800000	-2.332206000	2.669560000
1	6.388832000	-2.524673000	2.610129000
6	7.967557000	-2.237736000	4.043924000
6	9.268794000	-1.829329000	4.356241000
6	7.118484000	-2.569152000	5.103571000
6	9.702209000	-1.770236000	5.670508000
6	7.549543000	-2.510290000	6.421119000
6	8.846490000	-2.111929000	6.712087000
1	9.952313000	-1.545718000	3.565580000
1	6.103244000	-2.882132000	4.887633000
1	10.713798000	-1.449626000	5.885564000
1	6.870056000	-2.775693000	7.221101000
1	9.187552000	-2.062661000	7.738030000

E PCM(DCM)-PBEh-3c	-2132.856547
ZPVE	0.38128303
H	0.4161447
G	0.32051828
E PCM(DCM)-revDSD-PBEP86-D4/def2-QZVPP	-2134.909912

TS$_{5/3}$

5	7.380363000	-2.085629000	-0.278466000
6	8.373512000	-3.240641000	-0.911662000

6	8.030088000	-4.329071000	-1.621426000
1	8.759737000	-5.038692000	-1.995243000
1	7.003486000	-4.490958000	-1.926824000
6	9.735183000	-3.025660000	-0.558016000
6	10.637050000	-3.965092000	-0.016925000
6	10.216991000	-5.228681000	0.450850000
6	11.147434000	-6.128677000	0.922313000
6	12.496721000	-5.781418000	0.948991000
6	12.926551000	-4.527218000	0.520102000
6	12.003551000	-3.617735000	0.054586000
1	12.319148000	-2.636791000	-0.278475000
1	13.975569000	-4.269522000	0.561436000
1	13.220995000	-6.491277000	1.326962000
1	10.830905000	-7.095031000	1.289262000
1	9.163623000	-5.470622000	0.476265000
1	10.135200000	-2.026533000	-0.710831000
6	5.886573000	-2.758934000	-0.187129000
6	5.620823000	-3.809681000	0.682427000
6	4.385340000	-4.416384000	0.812569000
6	3.336363000	-3.992179000	0.021974000
6	3.554664000	-2.983515000	-0.892669000
6	4.808561000	-2.401781000	-0.985548000
9	4.931623000	-1.463873000	-1.931083000
9	2.560286000	-2.582460000	-1.682570000
9	2.140090000	-4.559590000	0.128869000
9	4.200135000	-5.406959000	1.683247000
9	6.597861000	-4.323792000	1.442027000
6	7.382426000	-0.677586000	-1.103692000
6	6.717605000	0.409201000	-0.552772000
6	6.671633000	1.658484000	-1.137245000
6	7.314756000	1.862237000	-2.344687000
6	7.984707000	0.812651000	-2.936419000
6	8.005128000	-0.424927000	-2.310978000
9	8.670061000	-1.393282000	-2.955135000
9	8.602533000	0.995646000	-4.103009000
9	7.283030000	3.056529000	-2.928266000
9	6.015327000	2.663668000	-0.559963000
9	6.054310000	0.260428000	0.599257000
6	8.064384000	-1.813408000	1.178325000
1	7.875537000	-2.521376000	1.980407000
6	8.967339000	-0.858118000	1.450603000
1	9.183374000	-0.097704000	0.701969000
6	9.729978000	-0.703003000	2.694038000
6	9.823774000	-1.708669000	3.662405000
6	10.410567000	0.495364000	2.927541000
6	10.551598000	-1.512233000	4.824240000
6	11.139205000	0.694596000	4.091004000
6	11.211807000	-0.308824000	5.047157000
1	9.333242000	-2.661024000	3.503818000
1	10.360065000	1.284350000	2.185911000
1	10.610628000	-2.305418000	5.558717000
1	11.651800000	1.634738000	4.249963000

1	11.781165000	-0.158942000	5.955242000	
E PCM(DCM)-PBEh-3c				-2132.855741
ZPVE				0.38080935
H				0.41502237
G				0.3208142
E PCM(DCM)-revDSD-PBEP86-D4/def2-QZVPP				-2134.907772

3

5	-0.705062000	-0.589033000	-0.662365000
6	0.334645000	-1.726445000	-0.769264000
6	-0.105910000	-2.928286000	-1.164849000
1	0.560793000	-3.755045000	-1.381239000
1	-1.160361000	-3.122118000	-1.318779000
6	1.807099000	-1.410173000	-0.538552000
6	2.703656000	-2.626243000	-0.558047000
6	2.644247000	-3.584788000	0.453031000
6	3.470371000	-4.698182000	0.422524000
6	4.370414000	-4.873335000	-0.621432000
6	4.436032000	-3.926809000	-1.633297000
6	3.607069000	-2.812407000	-1.598921000
1	3.664364000	-2.077248000	-2.393268000
1	5.133358000	-4.052930000	-2.451587000
1	5.015455000	-5.742108000	-0.644357000
1	3.411040000	-5.432164000	1.215966000
1	1.942304000	-3.469255000	1.270377000
1	2.135214000	-0.747671000	-1.346675000
6	-2.180835000	-0.898291000	-0.236305000
6	-2.477400000	-1.621554000	0.908837000
6	-3.775514000	-1.900364000	1.290284000
6	-4.820937000	-1.456586000	0.499956000
6	-4.565776000	-0.733133000	-0.651353000
6	-3.256013000	-0.458156000	-0.992124000
9	-3.031033000	0.224410000	-2.113052000
9	-5.573161000	-0.319187000	-1.410089000
9	-6.069275000	-1.725217000	0.846400000
9	-4.030599000	-2.583367000	2.399617000
9	-1.493326000	-2.037062000	1.701950000
6	-0.329241000	0.897395000	-1.003374000
6	-0.526669000	1.905915000	-0.076654000
6	-0.156980000	3.214123000	-0.316054000
6	0.411161000	3.538569000	-1.535590000
6	0.609211000	2.560239000	-2.493668000
6	0.241893000	1.259001000	-2.209412000
9	0.439501000	0.329937000	-3.144991000
9	1.144593000	2.879534000	-3.666197000
9	0.766142000	4.789702000	-1.786393000
9	-0.331405000	4.154713000	0.604114000
9	-1.045014000	1.603968000	1.113013000
6	1.943611000	-0.648143000	0.752552000
1	1.625316000	-1.167949000	1.651695000
6	2.380646000	0.610135000	0.814242000

1	2.663219000	1.099163000	-0.115322000
6	2.485216000	1.449122000	2.009939000
6	2.366814000	0.947569000	3.309730000
6	2.702723000	2.819176000	1.848015000
6	2.453236000	1.791541000	4.403912000
6	2.786809000	3.666239000	2.943570000
6	2.661139000	3.155385000	4.227130000
1	2.216384000	-0.112158000	3.473125000
1	2.810158000	3.224686000	0.848402000
1	2.362842000	1.383528000	5.402415000
1	2.953227000	4.725029000	2.792956000
1	2.729366000	3.811350000	5.085135000

E PCM(DCM)-PBEh-3c	-2172.15067
ZPVE	0.41128057
H	0.44807166
G	0.34748751
E PCM(DCM)-revDSD-PBEP86-D4/def2-QZVPP	-2134.962976

TS$_{\text{indene}}$

5	7.812959000	-2.653282000	0.352650000
6	9.070075000	-3.220508000	-0.544038000
6	8.953793000	-4.226067000	-1.524323000
1	9.273925000	-4.024719000	-2.539905000
1	8.213563000	-5.014749000	-1.450589000
6	10.336528000	-2.670315000	-0.488556000
6	11.341606000	-3.469053000	-1.066898000
6	11.011638000	-4.847346000	-1.266039000
6	11.755441000	-5.607926000	-2.195341000
6	12.836511000	-5.041699000	-2.815381000
6	13.235594000	-3.718608000	-2.515646000
6	12.521740000	-2.947566000	-1.638153000
1	12.795504000	-1.917175000	-1.452398000
1	14.105019000	-3.305765000	-3.009574000
1	13.429799000	-5.625655000	-3.506399000
1	11.507775000	-6.648294000	-2.355043000
1	10.510527000	-5.391719000	-0.472157000
1	10.553321000	-1.714066000	-0.027620000
6	6.498163000	-3.621118000	0.192701000
6	6.555615000	-4.921351000	0.673066000
6	5.490062000	-5.800767000	0.663769000
6	4.279302000	-5.378777000	0.149253000
6	4.167700000	-4.095219000	-0.344255000
6	5.263620000	-3.247792000	-0.312106000
9	5.059159000	-2.021543000	-0.800614000
9	3.003924000	-3.679803000	-0.841123000
9	3.234401000	-6.199288000	0.127916000
9	5.615260000	-7.037379000	1.141329000
9	7.708632000	-5.383632000	1.177042000
6	8.108298000	-2.546542000	1.962259000
6	7.163145000	-1.913239000	2.759637000
6	9.185055000	-3.070923000	2.658110000

6	7.278703000	-1.769959000	4.129245000
6	9.342922000	-2.949753000	4.029528000
6	8.385076000	-2.291143000	4.771412000
9	10.150887000	-3.753729000	2.029667000
9	6.055199000	-1.410960000	2.206076000
9	6.337559000	-1.143278000	4.832988000
9	8.519777000	-2.167348000	6.087739000
9	10.406179000	-3.472836000	4.639128000
6	7.686852000	-1.200006000	-0.331931000
1	7.348272000	-1.178828000	-1.366403000
6	8.092997000	-0.039157000	0.196346000
1	8.462803000	-0.025976000	1.219571000
6	8.091978000	1.279352000	-0.448561000
6	7.715760000	1.486292000	-1.780443000
6	8.480396000	2.395294000	0.298311000
6	7.725476000	2.754990000	-2.335414000
6	8.491201000	3.667572000	-0.255198000
6	8.112573000	3.854735000	-1.576833000
1	7.411623000	0.647915000	-2.394562000
1	8.776286000	2.260701000	1.332541000
1	7.429064000	2.888457000	-3.368174000
1	8.795029000	4.513422000	0.348373000
1	8.118180000	4.844901000	-2.013575000

E PCM(DCM)-PBEh-3c	-2132.841733
ZPVE	0.38059876
H	0.4146427
G	0.32069716
E PCM(DCM)-revDSD-PBEP86-D4/def2-QZVPP	-2134.896322

TS’

5	8.369381000	-1.446212000	-0.184572000
6	9.371903000	-1.995104000	-1.311339000
6	9.607910000	-1.807378000	-2.606428000
1	10.322840000	-2.407579000	-3.158490000
1	9.127299000	-1.011073000	-3.159176000
6	10.035944000	-3.004020000	-0.500964000
6	10.177284000	-4.410643000	-0.806942000
6	9.492065000	-5.007200000	-1.871828000
6	9.684149000	-6.348626000	-2.149148000
6	10.543715000	-7.106800000	-1.362556000
6	11.223485000	-6.525260000	-0.296537000
6	11.039488000	-5.185297000	-0.018192000
1	11.563631000	-4.723889000	0.810534000
1	11.892852000	-7.119208000	0.310665000
1	10.685720000	-8.157163000	-1.580944000
1	9.160293000	-6.808346000	-2.976149000
1	8.821805000	-4.418791000	-2.482742000
1	10.794541000	-2.611691000	0.172833000
6	8.748463000	-0.060055000	0.577630000
6	9.635981000	0.887836000	0.094235000
6	9.856008000	2.108714000	0.712110000

6	9.169617000	2.425460000	1.865507000
6	8.274138000	1.512344000	2.389032000
6	8.086995000	0.307054000	1.742576000
9	7.217819000	-0.541140000	2.307828000
9	7.604975000	1.802998000	3.502165000
9	9.365286000	3.593606000	2.465908000
9	10.721373000	2.980505000	0.198612000
9	10.333184000	0.669355000	-1.021974000
6	8.580156000	-2.802395000	0.957928000
6	7.696658000	-3.885153000	0.862296000
6	7.450219000	-4.760506000	1.894449000
6	8.115823000	-4.584494000	3.097068000
6	9.018593000	-3.545596000	3.248470000
6	9.234656000	-2.690786000	2.190193000
9	10.148635000	-1.742092000	2.364884000
9	9.662028000	-3.396535000	4.399812000
9	7.898684000	-5.417721000	4.095604000
9	6.597703000	-5.768157000	1.756773000
9	7.086793000	-4.127897000	-0.287470000
6	6.826019000	-1.383065000	-0.590993000
1	6.111936000	-1.317499000	0.228824000
6	6.328865000	-1.324715000	-1.830775000
1	7.004523000	-1.405914000	-2.676628000
6	4.917490000	-1.169939000	-2.205394000
6	3.886424000	-1.010245000	-1.272978000
6	4.575237000	-1.180821000	-3.560146000
6	2.570809000	-0.873711000	-1.682161000
6	3.257517000	-1.044189000	-3.972999000
6	2.247244000	-0.890211000	-3.034748000
1	4.109151000	-0.988940000	-0.213589000
1	5.357423000	-1.300078000	-4.301105000
1	1.790940000	-0.751206000	-0.941266000
1	3.021093000	-1.057198000	-5.029317000
1	1.217888000	-0.782063000	-3.350921000

E PCM(DCM)-PBEh-3c	-2132.837348
ZPVE	0.38036457
H	0.41477779
G	0.31989827
E PCM(DCM)-revDSD-PBEP86-D4/def2-QZVPP	-2134.889493

diene 3, conformer 1

6	-1.052156000	-4.942609000	1.789361000
6	-0.736661000	-5.439351000	0.534926000
6	-0.354822000	-4.573598000	-0.479617000
6	-0.281161000	-3.202551000	-0.263146000
6	-0.598770000	-2.716988000	1.001557000
6	-0.979970000	-3.576446000	2.019326000
1	-1.223929000	-3.175833000	2.994413000
1	-0.539255000	-1.657623000	1.216200000
6	0.151446000	-2.291346000	-1.389695000

6	1.537208000	-1.732758000	-1.228454000
6	2.311957000	-1.807547000	-0.148760000
6	3.582513000	-1.099867000	0.023521000
6	4.324020000	-0.588852000	-1.043489000
6	5.458227000	0.171586000	-0.818485000
6	5.882094000	0.433275000	0.477529000
6	5.169967000	-0.087636000	1.547038000
6	4.036737000	-0.851693000	1.319589000
1	3.479721000	-1.246960000	2.160900000
1	5.494144000	0.104498000	2.561255000
1	6.764013000	1.035150000	0.650863000
1	6.015346000	0.562924000	-1.659339000
1	4.018506000	-0.791272000	-2.062238000
1	1.959915000	-2.353712000	0.720091000
1	1.882890000	-1.178253000	-2.095012000
6	-0.868267000	-1.180692000	-1.627812000
6	-1.674197000	-1.250090000	-2.694209000
1	-2.436322000	-0.507332000	-2.895517000
1	-1.585598000	-2.042443000	-3.430582000
5	-0.970823000	0.126924000	-0.798670000
6	-2.375744000	0.739020000	-0.473106000
6	-3.443661000	-0.046675000	-0.050298000
6	-4.678528000	0.489890000	0.265609000
6	-4.874721000	1.854293000	0.142683000
6	-3.841453000	2.671484000	-0.283502000
6	-2.614764000	2.105721000	-0.572116000
9	-1.652258000	2.916087000	-0.999356000
9	-4.041142000	3.975676000	-0.412010000
9	-6.052105000	2.379171000	0.430792000
9	-5.670493000	-0.283210000	0.684609000
9	-3.290679000	-1.353591000	0.104818000
6	0.299941000	0.925188000	-0.323457000
6	0.533121000	1.189516000	1.013056000
6	1.657127000	1.862531000	1.454495000
6	2.568547000	2.331224000	0.525592000
6	2.355075000	2.111740000	-0.824561000
6	1.231902000	1.413812000	-1.222127000
9	1.039064000	1.219491000	-2.527938000
9	3.221946000	2.571844000	-1.715830000
9	3.640171000	2.993062000	0.927328000
9	1.866687000	2.063938000	2.748844000
9	-0.338737000	0.750166000	1.926521000
1	0.161033000	-2.899556000	-2.302392000
1	-0.108805000	-4.972854000	-1.456486000
1	-0.789355000	-6.502792000	0.342001000
1	-1.353740000	-5.613675000	2.582584000

E-PBEh-3c	-2132.899613
ZPVE	0.384235
H	0.418781
G	0.324065
E DLPNO-CCSD(T)/def2-TZVP	-2134.064888

diene 3, conformer 2

6	0.389261000	-6.436635000	-0.230574000
6	0.928954000	-5.417932000	-1.004854000
6	0.698925000	-4.093377000	-0.673368000
6	-0.078278000	-3.762825000	0.435145000
6	-0.612386000	-4.789209000	1.203260000
6	-0.380936000	-6.118552000	0.875856000
1	-0.804100000	-6.904282000	1.487876000
1	-1.218416000	-4.548980000	2.068902000
6	-0.364210000	-2.317258000	0.779692000
6	-1.234787000	-1.691636000	-0.272083000
6	-2.489998000	-1.303491000	-0.059647000
6	-3.330365000	-0.509810000	-0.955751000
6	-4.553422000	-0.036489000	-0.479003000
6	-5.341795000	0.810739000	-1.241065000
6	-4.923499000	1.197937000	-2.504296000
6	-3.715425000	0.723351000	-2.998978000
6	-2.928532000	-0.119871000	-2.235804000
1	-1.989601000	-0.471117000	-2.643988000
1	-3.381578000	1.018978000	-3.984784000
1	-5.531478000	1.865232000	-3.100230000
1	-6.280974000	1.172631000	-0.844217000
1	-4.883677000	-0.324890000	0.512205000
1	-2.925864000	-1.509965000	0.914962000
1	-0.755880000	-1.512767000	-1.231887000
6	0.909045000	-1.494796000	0.978677000
6	2.007097000	-2.057517000	1.502009000
1	2.916300000	-1.492134000	1.663052000
1	2.035641000	-3.095843000	1.813559000
5	0.969867000	0.025337000	0.695754000
6	2.338466000	0.673446000	0.281575000
6	2.861914000	1.754733000	0.971046000
6	4.061486000	2.346282000	0.622135000
6	4.756464000	1.857264000	-0.471364000
6	4.257356000	0.787554000	-1.195727000
6	3.063467000	0.210969000	-0.805019000
9	2.586739000	-0.795063000	-1.535447000
9	4.923092000	0.335838000	-2.249629000
9	5.902164000	2.414228000	-0.825696000
9	4.551147000	3.364481000	1.315315000
9	2.210710000	2.225983000	2.031450000
6	-0.280451000	0.964825000	0.786796000
6	-0.622592000	1.798812000	-0.264595000
6	-1.770128000	2.570474000	-0.260871000
6	-2.597802000	2.530146000	0.845397000
6	-2.279617000	1.721823000	1.925693000

6	-1.135206000	0.953348000	1.875793000	
9	-0.858669000	0.161644000	2.911541000	
9	-3.083356000	1.687144000	2.981696000	
9	-3.703067000	3.254616000	0.872444000	
9	-2.084061000	3.331009000	-1.298943000	
9	0.141020000	1.821470000	-1.357041000	
1	-0.920977000	-2.302515000	1.722982000	
1	1.139832000	-3.308459000	-1.275924000	
1	1.533828000	-5.657319000	-1.869654000	
1	0.571048000	-7.471397000	-0.488994000	
E-PBEh-3c				-2132.901356
ZPVE				0.383754
H				0.418576
G				0.322426
E DLPNO-CCSD(T)/def2-TZVP				-2134.064442

diene 3, conformer 3

6	0.239119000	-2.562775000	3.169933000
6	0.731230000	-3.241994000	2.063792000
6	1.222629000	-2.537296000	0.976138000
6	1.220127000	-1.147009000	0.969927000
6	0.749488000	-0.474634000	2.093399000
6	0.258211000	-1.176709000	3.184362000
1	-0.118281000	-0.637507000	4.043503000
1	0.748594000	0.608645000	2.115006000
6	1.703131000	-0.381016000	-0.244726000
6	3.079892000	0.151608000	0.017178000
6	4.151019000	-0.156705000	-0.712238000
6	5.520298000	0.314628000	-0.492241000
6	5.941565000	0.874868000	0.715663000
6	7.239693000	1.326614000	0.872542000
6	8.150775000	1.222072000	-0.170521000
6	7.751470000	0.654628000	-1.370351000
6	6.450658000	0.202756000	-1.525563000
1	6.147395000	-0.239101000	-2.467376000
1	8.454291000	0.560749000	-2.187699000
1	9.166332000	1.572741000	-0.043721000
1	7.546904000	1.754096000	1.818014000
1	5.254532000	0.941136000	1.549514000
1	4.014962000	-0.807561000	-1.572250000
1	3.167484000	0.824397000	0.865978000
6	0.713672000	0.708117000	-0.644478000
6	1.130893000	1.851622000	-1.202991000
1	0.434635000	2.619347000	-1.515789000
1	2.178499000	2.052810000	-1.398623000
5	-0.809943000	0.463453000	-0.533956000
6	-1.819915000	1.662614000	-0.458476000
6	-1.696085000	2.694875000	0.460009000
6	-2.612603000	3.726851000	0.541884000
6	-3.687326000	3.746651000	-0.330367000
6	-3.843084000	2.738695000	-1.266763000
6	-2.916493000	1.714340000	-1.307034000

9	-3.075787000	0.764147000	-2.224400000	
9	-4.868162000	2.768592000	-2.106514000	
9	-4.565728000	4.732579000	-0.270681000	
9	-2.475089000	4.690784000	1.441887000	
9	-0.687919000	2.698844000	1.327636000	
6	-1.396896000	-0.991888000	-0.511314000	
6	-1.088706000	-1.931276000	-1.478687000	
6	-1.539272000	-3.234779000	-1.414434000	
6	-2.319342000	-3.623164000	-0.337894000	
6	-2.652210000	-2.710742000	0.647420000	
6	-2.201821000	-1.409074000	0.534713000	
9	-2.525611000	-0.545467000	1.494246000	
9	-3.394230000	-3.091020000	1.678198000	
9	-2.748404000	-4.872527000	-0.252455000	
9	-1.232750000	-4.113514000	-2.360431000	
9	-0.331914000	-1.574807000	-2.517659000	
1	1.767370000	-1.088215000	-1.078743000	
1	1.598071000	-3.074467000	0.112939000	
1	0.725753000	-4.324076000	2.045534000	
1	-0.155072000	-3.111597000	4.014675000	
E-PBEh-3c				-2132.904339
ZPVE				0.383918
H				0.418639
G				0.322934
E DLPNO-CCSD(T)/def2-TZVP				-2134.067044

diene 3, conformer 4

6	-1.867037000	-4.871515000	1.945384000
6	-1.472623000	-5.376170000	0.716910000
6	-0.817935000	-4.558733000	-0.193617000
6	-0.547550000	-3.229990000	0.106720000
6	-0.947622000	-2.734009000	1.343625000
6	-1.601384000	-3.545372000	2.256041000
1	-1.906914000	-3.139938000	3.211574000
1	-0.753251000	-1.701811000	1.612566000
6	0.168972000	-2.349740000	-0.898630000
6	1.478889000	-1.862195000	-0.351007000
6	2.594093000	-1.772680000	-1.072049000
6	3.861145000	-1.193704000	-0.623250000
6	4.812327000	-0.830916000	-1.576085000
6	5.993358000	-0.206307000	-1.207975000
6	6.247112000	0.068110000	0.125889000
6	5.316080000	-0.300878000	1.088996000
6	4.141596000	-0.931051000	0.720331000
1	3.440575000	-1.229712000	1.489456000
1	5.512275000	-0.102939000	2.134579000
1	7.164752000	0.561385000	0.417066000
1	6.712864000	0.072583000	-1.966413000
1	4.615142000	-1.027475000	-2.623121000
1	2.562426000	-2.082256000	-2.113255000
1	1.479907000	-1.544727000	0.686283000
6	-0.753078000	-1.222528000	-1.351076000

6	-1.394363000	-1.338601000	-2.520679000
1	-2.089883000	-0.589818000	-2.878518000
1	-1.225920000	-2.178820000	-3.186580000
5	-0.920530000	0.138247000	-0.632995000
6	-2.321372000	0.836258000	-0.587336000
6	-2.466653000	2.192660000	-0.852134000
6	-3.690727000	2.831758000	-0.813467000
6	-4.818327000	2.102020000	-0.476563000
6	-4.716594000	0.751016000	-0.193015000
6	-3.478579000	0.138446000	-0.259741000
9	-3.414277000	-1.149945000	0.043863000
9	-5.800736000	0.064500000	0.139837000
9	-5.996338000	2.698158000	-0.426689000
9	-3.798923000	4.122629000	-1.095338000
9	-1.404572000	2.913505000	-1.197567000
6	0.298290000	0.895417000	0.009268000
6	0.295351000	1.258307000	1.343769000
6	1.368281000	1.891408000	1.943832000
6	2.475070000	2.205510000	1.174866000
6	2.503269000	1.885314000	-0.172398000
6	1.420360000	1.233976000	-0.729598000
9	1.458209000	0.941384000	-2.025357000
9	3.559905000	2.203558000	-0.902421000
9	3.513116000	2.808179000	1.730801000
9	1.348210000	2.195155000	3.235540000
9	-0.761397000	0.948165000	2.100648000
1	0.382313000	-2.963304000	-1.779647000
1	-0.511001000	-4.963205000	-1.150846000
1	-1.673413000	-6.408957000	0.463900000
1	-2.378688000	-5.505607000	2.657100000

E-PBEh-3c	-2132.898245
ZPVE	0.384075
H	0.418685
G	0.323839
E DLPNO-CCSD(T)/def2-TZVP	-2134.061835

diene 3, conformer 5

6	-2.774621000	5.937568000	0.617125000
6	-2.250560000	5.819491000	-0.659542000
6	-1.625460000	4.642260000	-1.048558000
6	-1.513234000	3.571864000	-0.171234000
6	-2.040081000	3.702145000	1.112515000
6	-2.666983000	4.873453000	1.503065000
1	-3.071236000	4.958187000	2.503289000
1	-1.947178000	2.884787000	1.818212000
6	-0.857741000	2.282864000	-0.610297000
6	-1.856237000	1.165298000	-0.636259000
6	-2.026272000	0.334592000	-1.661953000
6	-2.848676000	-0.875956000	-1.631910000
6	-3.874668000	-1.067116000	-0.703488000
6	-4.539688000	-2.277542000	-0.623804000
6	-4.204185000	-3.320500000	-1.478034000

6	-3.210670000	-3.133795000	-2.426205000
6	-2.543947000	-1.921255000	-2.503992000
1	-1.758262000	-1.789690000	-3.238613000
1	-2.946219000	-3.936278000	-3.101728000
1	-4.720535000	-4.268393000	-1.408287000
1	-5.327224000	-2.409024000	0.106506000
1	-4.161041000	-0.260571000	-0.040470000
1	-1.437121000	0.488001000	-2.562679000
1	-2.418217000	1.012828000	0.279460000
6	0.391520000	1.914345000	0.187735000
6	1.088579000	2.833369000	0.867190000
1	2.023970000	2.589888000	1.356148000
1	0.744451000	3.853487000	0.992429000
5	0.933866000	0.463743000	0.291945000
6	0.039723000	-0.785988000	0.607696000
6	-0.845840000	-0.818837000	1.671815000
6	-1.634062000	-1.918562000	1.946721000
6	-1.545692000	-3.034844000	1.131083000
6	-0.674255000	-3.040747000	0.057605000
6	0.103624000	-1.923021000	-0.179443000
9	0.906625000	-1.931571000	-1.244460000
9	-0.596928000	-4.103606000	-0.730144000
9	-2.298099000	-4.092961000	1.381904000
9	-2.473448000	-1.917222000	2.974644000
9	-0.947417000	0.245118000	2.470566000
6	2.474787000	0.226845000	0.128905000
6	3.167986000	0.759797000	-0.948555000
6	4.522669000	0.556358000	-1.130313000
6	5.220750000	-0.192680000	-0.197699000
6	4.565952000	-0.737380000	0.894247000
6	3.206463000	-0.529506000	1.032348000
9	2.600354000	-1.048341000	2.096174000
9	5.245494000	-1.443488000	1.786969000
9	6.518767000	-0.389268000	-0.351071000
9	5.156396000	1.061164000	-2.179750000
9	2.512660000	1.454836000	-1.873246000
1	-0.501369000	2.421829000	-1.638613000
1	-1.220787000	4.556319000	-2.050062000
1	-2.327260000	6.643250000	-1.356995000
1	-3.263273000	6.853048000	0.923197000

E-PBEh-3c	-2132.900687
ZPVE	0.383991
H	0.418607
G	0.323360
E DLPNO-CCSD(T)/def2-TZVP	-2134.063731

diene 3, conformer 6

6	1.316814000	-6.259569000	-0.108871000
6	1.621184000	-5.212296000	-0.968541000
6	1.223465000	-3.921498000	-0.663248000
6	0.511496000	-3.654583000	0.504605000
6	0.213754000	-4.708996000	1.358115000

6	0.613093000	-6.004098000	1.056561000
1	0.372718000	-6.812382000	1.734708000
1	-0.338897000	-4.517795000	2.270414000
6	0.048420000	-2.251175000	0.826720000
6	-0.964130000	-1.791065000	-0.184792000
6	-2.248309000	-1.583435000	0.098587000
6	-3.264892000	-0.995738000	-0.773476000
6	-4.515573000	-0.695261000	-0.232062000
6	-5.492392000	-0.066072000	-0.986536000
6	-5.238336000	0.270504000	-2.306772000
6	-4.002036000	-0.031044000	-2.863191000
6	-3.026434000	-0.654906000	-2.106761000
1	-2.070751000	-0.877797000	-2.563026000
1	-3.796168000	0.226506000	-3.893731000
1	-5.996086000	0.764949000	-2.899628000
1	-6.451271000	0.163137000	-0.540993000
1	-4.720521000	-0.951316000	0.801045000
1	-2.585720000	-1.804312000	1.108608000
1	-0.578004000	-1.587866000	-1.180645000
6	1.198693000	-1.251294000	0.926020000
6	2.428643000	-1.641827000	1.286661000
1	3.245946000	-0.937020000	1.374269000
1	2.664095000	-2.670575000	1.535104000
5	0.971346000	0.266044000	0.733943000
6	-0.409285000	0.944754000	1.031325000
6	-1.084330000	0.759949000	2.224216000
6	-2.351182000	1.263869000	2.440199000
6	-2.976488000	1.972425000	1.426667000
6	-2.331284000	2.183526000	0.221663000
6	-1.056148000	1.678933000	0.051958000
9	-0.464937000	1.851136000	-1.130344000
9	-2.940297000	2.845655000	-0.749591000
9	-4.202107000	2.434691000	1.609475000
9	-2.979759000	1.068555000	3.593058000
9	-0.506126000	0.057328000	3.198662000
6	2.136911000	1.170981000	0.203135000
6	2.509142000	2.326956000	0.870815000
6	3.521458000	3.151692000	0.418280000
6	4.174824000	2.825961000	-0.758518000
6	3.821615000	1.686778000	-1.462459000
6	2.815928000	0.876676000	-0.969175000
9	2.472963000	-0.195001000	-1.680094000
9	4.444701000	1.391496000	-2.595001000
9	5.140968000	3.606223000	-1.212022000
9	3.872091000	4.239088000	1.090343000
9	1.898664000	2.645037000	2.009073000
1	-0.449019000	-2.280218000	1.802688000
1	1.481275000	-3.110916000	-1.334703000
1	2.172161000	-5.402649000	-1.880280000
1	1.628966000	-7.267657000	-0.347335000

E-PBEh-3c -2132.901489
ZPVE 0.383747

H	0.418590
G	0.322182
E DLPNO-CCSD(T)/def2-TZVP	-2134.064355

diene 3, conformer 7

6	3.462262000	-5.216134000	0.879763000
6	2.944634000	-4.210023000	1.682869000
6	2.076414000	-3.268016000	1.152707000
6	1.712430000	-3.314013000	-0.190613000
6	2.238618000	-4.326246000	-0.985460000
6	3.105508000	-5.272185000	-0.458293000
1	3.499668000	-6.053983000	-1.094164000
1	1.968357000	-4.374787000	-2.033849000
6	0.811836000	-2.273422000	-0.813747000
6	1.538072000	-1.018882000	-1.207327000
6	2.736962000	-0.617004000	-0.794806000
6	3.301653000	0.705360000	-1.075652000
6	2.895176000	1.491705000	-2.155666000
6	3.363607000	2.785470000	-2.306836000
6	4.257906000	3.319202000	-1.388390000
6	4.694577000	2.539334000	-0.328325000
6	4.225677000	1.244008000	-0.179406000
1	4.563118000	0.645693000	0.658499000
1	5.397049000	2.941997000	0.389234000
1	4.616468000	4.333212000	-1.503518000
1	3.032344000	3.380057000	-3.148006000
1	2.211745000	1.088459000	-2.892256000
1	3.311082000	-1.249582000	-0.125722000
1	0.966563000	-0.366966000	-1.862406000
6	-0.455117000	-1.952085000	-0.018547000
6	-1.094231000	-2.921471000	0.651262000
1	-2.028470000	-2.739442000	1.167520000
1	-0.693981000	-3.924995000	0.744035000
5	-1.068504000	-0.535930000	0.132945000
6	-2.630212000	-0.387495000	0.121716000
6	-3.395707000	-0.933245000	-0.898357000
6	-4.769915000	-0.793551000	-0.947058000
6	-5.411185000	-0.096763000	0.063472000
6	-4.682211000	0.459385000	1.101338000
6	-3.307676000	0.315483000	1.106294000
9	-2.626778000	0.843339000	2.119500000
9	-5.306268000	1.115090000	2.069823000
9	-6.725842000	0.037972000	0.037532000
9	-5.475850000	-1.309940000	-1.943338000
9	-2.798674000	-1.578541000	-1.895826000
6	-0.232777000	0.779443000	0.333821000
6	0.725942000	0.913275000	1.322831000
6	1.449029000	2.075081000	1.504379000
6	1.222606000	3.150425000	0.662022000
6	0.284468000	3.051823000	-0.349243000
6	-0.433671000	1.878497000	-0.484864000
9	-1.318286000	1.792988000	-1.480955000

9	0.088676000	4.070939000	-1.174975000
9	1.905192000	4.270529000	0.824395000
9	2.354779000	2.171028000	2.468424000
9	0.958947000	-0.105779000	2.148218000
1	0.434013000	-2.703511000	-1.751640000
1	1.675290000	-2.494105000	1.792509000
1	3.216177000	-4.157886000	2.729180000
1	4.136562000	-5.952954000	1.295779000

E-PBEh-3c	-2132.899220
ZPVE	0.384060
H	0.418661
G	0.323356
E DLPNO-CCSD(T)/def2-TZVP	-2134.062384

diene 3, conformer 8

6	4.166281000	2.640774000	-0.778820000
6	3.163477000	2.951149000	0.126304000
6	1.839882000	2.649273000	-0.163645000
6	1.500573000	2.019992000	-1.356133000
6	2.513184000	1.733437000	-2.267503000
6	3.833976000	2.039262000	-1.984537000
1	4.606889000	1.800302000	-2.703033000
1	2.266872000	1.248737000	-3.204813000
6	0.069901000	1.642789000	-1.702712000
6	-0.934916000	2.071330000	-0.674556000
6	-1.908027000	2.950497000	-0.900326000
6	-2.938024000	3.358846000	0.057894000
6	-3.305933000	2.565720000	1.146634000
6	-4.269466000	2.996401000	2.041648000
6	-4.894780000	4.223364000	1.863787000
6	-4.552465000	5.012958000	0.776941000
6	-3.587375000	4.580665000	-0.118317000
1	-3.324609000	5.204834000	-0.964164000
1	-5.039146000	5.967181000	0.623917000
1	-5.650827000	4.555371000	2.562811000
1	-4.543648000	2.365381000	2.876865000
1	-2.854450000	1.591288000	1.283305000
1	-1.949405000	3.436773000	-1.871521000
1	-0.855837000	1.606460000	0.304670000
6	-0.094837000	0.145927000	-1.919894000
6	-0.431746000	-0.336728000	-3.120923000
1	-0.590208000	-1.394218000	-3.293833000
1	-0.537455000	0.304701000	-3.989752000
5	0.176054000	-0.881627000	-0.791497000
6	-0.754433000	-2.125417000	-0.616307000
6	-0.244604000	-3.397168000	-0.388070000
6	-1.055283000	-4.503134000	-0.220214000
6	-2.430048000	-4.338338000	-0.252231000
6	-2.979677000	-3.084115000	-0.456784000
6	-2.138790000	-2.002429000	-0.641868000
9	-2.695450000	-0.808132000	-0.797817000
9	-4.296558000	-2.935054000	-0.466717000

9	-3.220962000	-5.382961000	-0.084612000
9	-0.538723000	-5.709137000	-0.032033000
9	1.070676000	-3.582310000	-0.366219000
6	1.380386000	-0.688875000	0.199339000
6	1.167893000	-0.381584000	1.527698000
6	2.205500000	-0.110983000	2.399803000
6	3.505250000	-0.173836000	1.929657000
6	3.756902000	-0.509419000	0.609520000
6	2.692301000	-0.766148000	-0.230197000
9	2.937897000	-1.115972000	-1.490222000
9	5.006191000	-0.580482000	0.173271000
9	4.514501000	0.086931000	2.746237000
9	1.971008000	0.210506000	3.665609000
9	-0.088447000	-0.296289000	1.979807000
1	-0.181304000	2.142988000	-2.645176000
1	1.069400000	2.916379000	0.548147000
1	3.408728000	3.433938000	1.063448000
1	5.198304000	2.871465000	-0.550573000

E-PBEh-3c	-2132.900616
ZPVE	0.383912
H	0.418588
G	0.323275
E DLPNO-CCSD(T)/def2-TZVP	-2134.063850

diene 3, conformer 9

6	4.164398000	2.633138000	-0.861216000
6	3.824405000	2.004613000	-2.050911000
6	2.501823000	1.692776000	-2.318595000
6	1.494855000	2.000223000	-1.407741000
6	1.841993000	2.656121000	-0.231906000
6	3.167356000	2.964010000	0.042980000
1	3.418604000	3.467630000	0.967480000
1	1.076085000	2.939389000	0.478503000
6	0.061748000	1.616410000	-1.736786000
6	-0.935158000	2.065423000	-0.709942000
6	-1.916898000	2.931690000	-0.947820000
6	-2.939399000	3.360204000	0.009551000
6	-3.617405000	4.559273000	-0.209471000
6	-4.577535000	5.011230000	0.681494000
6	-4.885786000	4.264302000	1.807820000
6	-4.231028000	3.059986000	2.029773000
6	-3.272665000	2.609752000	1.139031000
1	-2.796841000	1.653197000	1.314001000
1	-4.477768000	2.461878000	2.897146000
1	-5.637716000	4.611462000	2.503910000
1	-5.086827000	5.947354000	0.494147000
1	-3.381617000	5.150358000	-1.086469000
1	-1.972785000	3.390349000	-1.931619000
1	-0.842094000	1.626109000	0.279683000
6	-0.104515000	0.115514000	-1.923284000
6	-0.445582000	-0.391349000	-3.112995000
1	-0.604652000	-1.452143000	-3.263790000

1	-0.554361000	0.232352000	-3.994274000
5	0.169627000	-0.887414000	-0.773824000
6	-0.760074000	-2.126703000	-0.566980000
6	-2.144628000	-2.004861000	-0.591194000
6	-2.984554000	-3.081992000	-0.376799000
6	-2.433954000	-4.330680000	-0.143305000
6	-1.058996000	-4.494300000	-0.111955000
6	-0.249279000	-3.392590000	-0.309479000
9	1.066041000	-3.577461000	-0.287452000
9	-0.541373000	-5.695141000	0.104126000
9	-3.223875000	-5.371005000	0.053089000
9	-4.301480000	-2.933441000	-0.385801000
9	-2.702708000	-0.815259000	-0.773961000
6	1.377763000	-0.673195000	0.208073000
6	2.687901000	-0.760520000	-0.224847000
6	3.756074000	-0.486330000	0.604777000
6	3.509942000	-0.121592000	1.918219000
6	2.212086000	-0.047469000	2.391990000
6	1.170851000	-0.336635000	1.530169000
9	-0.083550000	-0.240795000	1.985866000
9	1.982822000	0.302035000	3.651286000
9	4.522579000	0.156462000	2.724840000
9	5.003566000	-0.568182000	0.165360000
9	2.928442000	-1.138192000	-1.477785000
1	-0.196220000	2.097626000	-2.687314000
1	2.249754000	1.186905000	-3.243103000
1	4.592761000	1.749132000	-2.768610000
1	5.197873000	2.868709000	-0.644822000

E-PBEh-3c	-2132.900622
ZPVE	0.383889
H	0.418590
G	0.323117
E DLPNO-CCSD(T)/def2-TZVP	-2134.063781

C_6H_6

6	-4.289446000	1.199629000	0.000000000
6	-4.404933000	-0.183237000	0.000000000
6	-3.034064000	1.791016000	0.000000000
6	-1.894207000	0.999543000	0.000000000
6	-2.009694000	-0.383322000	0.000000000
6	-3.265076000	-0.974710000	0.000000000
1	-1.120786000	-1.000545000	0.000000000
1	-3.355184000	-2.053131000	0.000000000
1	-2.943956000	2.869438000	0.000000000
1	-0.915220000	1.460724000	0.000000000
1	-5.178354000	1.816852000	0.000000000
1	-5.383920000	-0.644418000	0.000000000

BH_3NMe_3

1	-1.036429000	1.556347000	1.247698000
1	-1.039907000	-0.189877000	2.257259000

1	0.608575000	0.389038000	1.250306000
5	-0.601035000	0.427496000	1.311495000
7	-1.151625000	-0.350955000	-0.037292000
6	-0.658321000	-1.736676000	-0.029270000
1	-1.010294000	-2.233622000	0.871107000
1	-1.005723000	-2.282842000	-0.909606000
1	0.428418000	-1.727255000	-0.010415000
6	-2.622512000	-0.346738000	-0.031542000
1	-2.975429000	0.681138000	-0.011601000
1	-3.020063000	-0.854641000	-0.913575000
1	-2.975917000	-0.845649000	0.867182000
6	-0.656546000	0.346665000	-1.233909000
1	-1.005964000	1.375780000	-1.215641000
1	0.430288000	0.355857000	-1.215969000
1	-1.004646000	-0.143007000	-2.146693000

13 Reference

[1] Y. Soltani, L. C. Wilkins, R. L. Melen,*Angew. Chem. Int. Ed.* **2017**, *56*, 11995-11999.

[2] L. E. Longobardi, T. C. Johnstone, R. L. Falconer, C. A. Russell, D. W. Stephan, *Chem. Eur. J.***2016**, *22*, 12665-12669.

[3] a) D. Müller, A. Alexakis, *Chem. Eur. J.***2013**, *19*, 15226-15239; b) Z.-F. Xu, L. Shan, W. Zhang, M. Cen, C.-Y. Li, *Org. Chem. Front.* **2019**, *6*, 1391-1396.

[4] a) Y. Thummala, G. V. Karunakar, V. R. Doddi, *Adv. Synth. Catal.* **2019**, *361*, 611-616; b) E.-i. Negishi, M. Kotora, C. Xu, *J. Org. Chem* **1997**, *62*, 8957-8960.

[5] M. W. Lee, Y. V. Sevryugina, A. Khan, S. Q. Ye, *J. Med. Chem.* **2012**, *55*, 7290-7294.

[6] B. N. Bhawal, J. C. Reisenbauer, C. Ehinger, B. Morandi, *J. Am. Chem. Soc.* **2020**, *142*, 10914-10920.

[7] a) M. Tang, S. Han, S. Huang, S. Huang, L.-G. Xie, *Org. Lett.* **2020**, *22*, 9729-9734; b) C. A. Faler, M. M. Joullié, *Org. Lett.* **2007**, *9*, 1987-1990.

[8] a) M. Yonehara, S. Nakamura, A. Muranaka, M. Uchiyama, *Chem. Asian. J.* **2010**, *5*, 452-455; b) Z. Zhao, L. Racicot, G. K. Murphy,*Angew. Chem. Int. Ed.* **2017**, *56*, 11620-11623.

[9] R. F. Snead, J. Nekvinda, W. L. Santos, *New J. Chem.* **2021**, *45*, 14925-14931.

[10] L. Cui, M. Chen, C. Chen, D. Liu, Z. Jian, *Macromolecules* **2019**, *52*, 7197-7206.

[11] X. Tao, C. G. Daniliuc, K. Soloviova, C. A. Strassert, G. Kehr, G. Erker, *Chem. Commun.* **2019**, *55*, 10166-10169.

[12] D. J. Parks, W. E. Piers, G. P. A. Yap, *Organometallics* **1998**, *17*, 5492-5503.

[13] L. Krause, R. Herbst-Irmer, G. M. Sheldrick, D. Stalke, *J. Appl. Crystallogr.* **2015**, *48*, 3-10.

[14] G. Sheldrick, *Acta Crystallogr. Sect. A* **2015**, *71*, 3-8.

[15] G. Sheldrick, *Acta Crystallogr. Sect. C* **2015**, *71*, 3-8.

[16] P. Müller, *Crystallogr. Rev.* **2009**, *15*, 57-83.

[17] A. Thorn, B. Dittrich, G. M. Sheldrick, *Acta Crystallogr. Sect. A* **2012**, *68*, 448-451.

[18] S. Parsons, H. D. Flack, T. Wagner, *Acta Crystallogr. Sect. B* **2013**, *69*, 249-259.

[19] R. W. W. Hooft, L. H. Straver, A. L. Spek, *J. Appl. Crystallogr.* **2010**, *43*, 665-668.

[20] M. D. Hanwell, D. E. Curtis, D. C. Lonie, T. Vandermeersch, E. Zurek, G. R. Hutchison, *J. Cheminf.* **2012**, *4*, 17.

[21] C. Bannwarth, S. Ehlert, S. Grimme, *J. Chem. Theory Comput.* **2019**, *15*, 1652-1671.

[22] C. Bannwarth, E. Caldeweyher, S. Ehlert, A. Hansen, P. Pracht, J. Seibert, S. Spicher, S. Grimme, *WIREs Comput. Mol. Sci.***2021**, *11*, e1493.

[23] a) F. Neese, *WIREs Comput. Mol. Sci.***2012**, *2*, 73-78; b) F. Neese, *WIREs Comput. Mol. Sci.***2018**, *8*, e1327.

[24] S. Grimme, J. G. Brandenburg, C. Bannwarth, A. Hansen, *J. Chem. Phys.* **2015**, *143*, 054107.

[25] G. Santra, N. Sylvetsky, J. M. L. Martin, *J. Phys. Chem. A* **2019**, *123*, 5129-5143.

[26] E. Caldeweyher, S. Ehlert, A. Hansen, H. Neugebauer, S. Spicher, C. Bannwarth, S. Grimme, *J. Chem. Phys.* **2019**, *150*, 154122.

[27] F. Weigend, R. Ahlrichs, *Phys. Chem. Chem. Phys.* **2005**, *7*, 3297-3305.

[28] L. Goerigk, A. Hansen, C. Bauer, S. Ehrlich, A. Najibi, S. Grimme, *Phys. Chem. Chem. Phys.* **2017**, *19*, 32184-32215.

[29] a) F. Neese, *J. Comput. Chem.* **2003**, *24*, 1740-1747; b) F. Neese, F. Wennmohs, A. Hansen, U. Becker, *Chem. Phys.* **2009**, *356*, 98-109.

[30] A. Hellweg, C. Hättig, S. Höfener, W. Klopper, *Theor. Chem. Acc.* **2007**, *117*, 587-597.

[31] F. Weigend, *Phys. Chem. Chem. Phys.* **2006**, *8*, 1057-1065.

[32] V. Barone, M. Cossi, *J. Phys. Chem. A* **1998**, *102*, 1995-2001.

[33] P. Pracht, F. Bohle, S. Grimme, *Phys. Chem. Chem. Phys.* **2020**, *22*, 7169-7192.

[34] C. Riplinger, B. Sandhoefer, A. Hansen, F. Neese, *J. Chem. Phys.* **2013**, *139*, 134101.

[35] aJ. P. Perdew, K. Burke, M. Ernzerhof, *Phys. Rev. Lett.* **1996**, *77*, 3865-3868; bC. Adamo, V. Barone, *J. Chem. Phys.* **1999**, *110*, 6158-6170.

[36] R. Ditchfield, *Mol. Phys.* **1974**, *27*, 789-807.

[37] F. Jensen, *J. Chem. Theory Comput.* **2008**, *4*, 719-727.
